# Cobalt-catalyzed diastereo- and enantioselective reductive coupling of cyclobutenes and aldehydes through umpolung reactivity

**DOI:** 10.1039/d5sc03755g

**Published:** 2025-08-04

**Authors:** Chuiyi Lin, Jiwu Zhang, Zhihan Zhang, Qinglei Chong, Fanke Meng

**Affiliations:** a State Key Laboratory of Organometallic Chemistry, Center for Excellence in Molecular Synthesis, Shanghai Institute of Organic Chemistry, University of Chinese Academy of Sciences 345 Lingling Road Shanghai 200032 China mengf@sioc.ac.cn chongql@sioc.ac.cn; b College of Chemistry, Central China Normal University 152 Louyu Road Wuhan Hubei 430079 China zhihanzhang@ccnu.edu.cn; c School of Chemistry and Materials Science, Hangzhou Institute for Advanced Study, University of Chinese Academy of Sciences 1 Sub-lane Xiangshan Hangzhou 310024 China; d Beijing National Laboratory for Molecular Sciences China

## Abstract

Catalytic diastereo- and enantioselective functionalization of cyclobutenes represents a general and modular strategy for the construction of enantioenriched complex cyclobutanes. However, all precedents focused on reactions of cyclobutenes with nucleophilic organometallic intermediates, whereas transformations of cyclobutenes with electrophiles remained unknown. Herein, we report an unprecedented cobalt-catalyzed protocol for diastereo- and enantioselective reductive coupling of unactivated cyclobutenes and aldehydes. This process enabled access to a broad range of densely functionalized enantioenriched cyclobutanes and the introduction of a chiral functionalized alkyl group with high efficiency and stereoselectivity. Mechanistic studies revealed that diastereo- and enantioselective oxidative cyclization of cyclobutenes and aldehydes followed by stereoselective protonation might be involved. DFT (Density Functional Theory) calculations elucidated the origin of stereoselectivity. This study provides a new platform for modular synthesis of enantioenriched cyclobutanes and reveals new reactivity for cobalt catalysis, pushing forward the advancement in organocobalt chemistry.

## Abstract 2

A novel approach for highly diastereo- and enantioselective reductive coupling of cyclobutenes and aldehydes through an oxidative cyclization pathway promoted by a readily available cobalt-based catalyst was developed.

## Graphical abstract

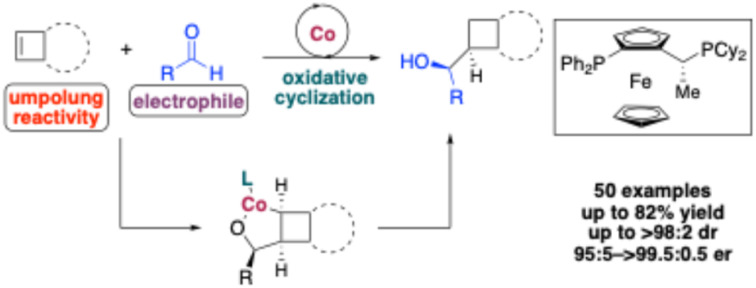

## Introduction

**Scheme 1:**
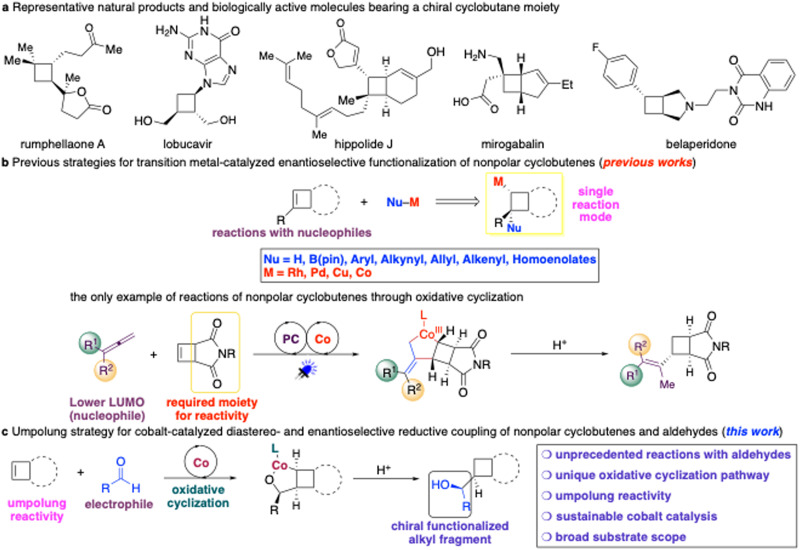
Background and reaction design. Pin, pinacolato; LUMO, lowest unoccupied molecular orbital.

Enantioenriched cyclobutanes widely exist in natural products and pharmaceutical important molecules^1^ and are crucial intermediates in organic synthesis (Scheme 1a).^2^ Therefore, development of a general and modular strategy for the catalytic enantioselective synthesis of a broad range of functionalized cyclobutanes is in high demand. Catalytic approaches for the preparation of enantioenriched cyclobutanes can be divided into two categories: ring formation reactions and functionalization of preformed four-membered rings. Catalytic enantioselective ring formation reactions involve [2 + 2] addition,^3^ cyclization of acyclic precursors,^4^ multi-step ring contraction, and expansion reactions,^5^ albeit with significant limitation to specific substrate patterns. An alternative strategy is catalytic enantioselective functionalization of preformed four-membered rings, including directing group-controlled C–H functionalization of cyclobutanes as well as transformations of cyclobutanones and cyclobutenes.^6^ The advantage of this method is that diversified functional groups can be installed directly onto the four-membered carbocycles through a single set of reactions and starting materials without the requirement of multistep transformations of specific substituents.^7^ Although progress has been made in this field, significant limitations remained unaddressed.

In the context of catalytic enantioselective C–H functionalization of cyclobutanes, an aryl, alkenyl or boryl can be incorporated with the need of a directing group.^8^ Only reduction and reactions involving ketone enolates have been reported for catalytic enantioselective reactions of cyclobutanones.^9^ Moreover, few protocols for catalytic enantioselective conjugate addition of activated cyclobutenes have been developed, including Cu-catalyzed hydride, boryl, and simple alkyl addition with dialkylzinc reagents,^10^ organocatalyzed Diels–Alder reactions,^11^ and Rh-catalyzed arylation with aryl boronic acids.^12^ A more challenging class of substrates is nonpolar cyclobutenes. The sluggish reactivity arose from relatively lower olefinic strain (1.9 kcal mol^−1^) compared with that in cyclopropenes (27.7 kcal mol^−1^) and bicyclic olefins (norbornene: 4.8 kcal mol^−1^).^13^ Although metal-catalyzed enantioselective hydroboration,^14^ borylallylation,^15^ hydroamination,^16^ and diboration^17^ of cyclobutenes have been revealed, transformations that enabled the construction of carbon–carbon bonds remained scarce (Scheme 1b). Fletcher and co-workers reported a series of Rh-catalyzed processes for enantioselective cascade arylation of cyclobutenes with aryl boronic acids, enantioselective hydroacylation of cyclobutenes with salicylaldehydes, as well as enantioselective arylation of cyclobutanone ketals (Scheme 1b).^18^ More recently, a Pd-catalyzed enantioselective process for cascade arylation of cyclobutenes with aryl iodides has been described by Lu and co-workers (Scheme 1b).^19^ We have disclosed a set of Co-catalyzed protocols for enantioselective carbon–carbon bond forming transformations of ester-substituted and unactivated cyclobutenes with Co homoenolates generated from cyclopropanols, alkynes, and potassium allyl trifluoroborate triggered by carbometallation (Scheme 1b).^20^ All of the precedent reactions mentioned above proceeded through the addition of a nucleophilic organometallic intermediate to cyclobutenes, whereas transformations of cyclobutenes with an electrophile such as aldehydes remained unknown. In addition, only achiral functional groups were able to be incorporated onto the four-membered carbocycles. Simultaneous generation of a stereogenic center on the newly introduced substituents remained elusive. Herein, we disclosed a catalytic protocol for enantioselective reductive coupling of cyclobutenes with electrophilic aldehydes promoted by an easily accessible chiral bisphosphine–Co complex to afford a broad range of enantioenriched cyclobutanes with high efficiency, diastereo- and enantioselectivity, enabling the introduction of chiral densely functionalized alkyl groups onto the four-membered ring scaffolds (Scheme 1c).

All of the catalytic enantioselective functionalization of cyclobutenes that has been reported proceeded through a single reaction mode, involving the addition of a nucleophile–metal species to cyclobutenes. However, transformations through other elementary processes remained far less-developed. Oxidative cyclization of two π-bonds promoted by low-valent transition metal complexes represents a classical elementary step in organometallic chemistry. Transition metal-catalyzed transformations of unsaturated hydrocarbons and aldehydes through oxidative cyclization constitute one of the most important strategies for carbon–carbon bond forming reactions in organic synthesis because pre-functionalization of the substrates and pre-formation of the stoichiometric sensitive organometallic reagents were not required.^21^ Therefore, catalytic enantioselective coupling of unsaturated hydrocarbons with aldehydes is an attractive approach for rapid construction of chiral alcohols with high atom- and step-economy.^22^ In this area, most research studies focused on catalytic enantioselective coupling of alkynes^23^ or 1,3-dienes^24^ and aldehydes promoted by Ni- and Co-based catalysts. Recently, we reported the only example of cobalt-catalyzed enantioselective coupling of 1,1-disubstituted allenes with aldehydes through divergent pathways.^25^ It is far more challenging for transformations of simple alkenes with electrophilic aldehydes or imines through oxidative cyclization due to higher-lying LUMO (lowest unoccupied molecular orbital) of the alkenes. A regio- and enantioselective Ni-catalyzed protocol for the reductive coupling of monosubstituted aliphatic alkenes with imines has been reported by Zhou and co-workers.^26^ Xiao and co-workers described a synergistic photoredox/Co-catalyzed coupling of cyclopropenes with imines, although a catalytic cycle involving Co–H addition to cyclopropenes followed by addition to imines instead of oxidative cyclization was proposed.^27^ We recently reported the first example of regio-, diastereo- and enantioselective reductive coupling of cyclobutenes with 1,1-disubstituted allenes.^28^ However, enantioselective reactions of less strained cyclobutenes with aldehydes remained undisclosed. We imagined that taking advantage of the unique reaction mode of cobalt catalysis and a proper choice of a chiral phosphine ligand might facilitate the oxidative cyclization of the inert cyclobutenes with aldehydes. In particular, we anticipated that an electron-rich ligand with stronger σ-donation might enhance the reactivity. We also expected that the chiral ligand was able to accurately control the diastereo- and enantioselectivity for the stereogenic centers newly generated at both the four-membered cyclic core and the side chain.

## Results and discussion

We commenced our studies by treatment of cyclobutene 1a with benzaldehyde 2a in the presence of Co complexes derived from a variety of chiral bisphosphines (Table 1). Zn powder was required for reduction of Co^II^ complexes to Co^I^ complexes to initiate the oxidative cyclization. Chiral bisphosphines bearing axial stereogenicity (4a), two stereogenic centers on the alkyl backbones (4c–d), and P-stereogenic centers (4i) were not able to promote the reaction (entries 1, 3, 4, and 9). No transformation was induced by phosphinooxazoline 4b (entry 2). Reaction of cyclobutene 1a with benzaldehyde 2a in the presence of bisphosphine containing a stereogenic center on the linker afforded the desired product 3a in 63% yield with 45 : 55 dr and 59 : 41 er (entry 5). Although no transformation occurred in the presence of bisphosphine 4g bearing chiral phospholane fragments and a phenyl tether (entry 7), reactions induced by the Co complex formed from bisphosphine 4h with an ethyl linker provided 3a in 48% yield with 68 : 32 dr and 27 : 73 er (entry 8). Highest efficiency and stereoselectivity were obtained with an electron-rich bisphosphine 4f containing a ferrocene backbone (entry 6). Other cobalt halides, such as CoCl_2_ and CoBr_2_, were much less reactive (entries 10 and 11). Lowering and elevating reaction temperature led to significant erosion of efficiency (entries 12 and 13). Further investigations on solvents revealed that the highest yield of desired product 3a could be obtained with reaction performed in DMA (*N*,*N*-dimethylacetamide) (SI). Increasing the amount of Zn improved the efficiency and the diastereoselectivity (entry 14). Switching the reductant from Zn to a photoredox catalytic system resulted in no reaction (entry 15).

**Table 1:** Optimization of reaction conditions

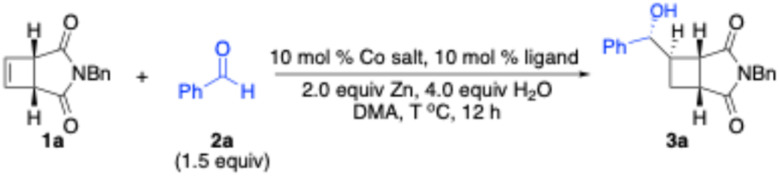						
Entry	Ligand	Co salt	*T* (°C)	Yield^a^ (%)	dr^b^	er^c^
1	4a	CoI_2_	70	<5	NA^d^	NA^d^
2	4b	CoI_2_	70	<5	NA^d^	NA^d^
3	4c	CoI_2_	70	<5	NA^d^	NA^d^
4	4d	CoI_2_	70	<5	NA^d^	NA^d^
5	4e	CoI_2_	70	63	45 : 55	59 : 41
6	4f	CoI_2_	70	68	87.5 : 12.5	>99.5 : 0.5
7	4g	CoI_2_	70	<5	NA^d^	NA^d^
8	4h	CoI_2_	70	48	68 : 32	27 : 73
9	4i	CoI_2_	70	<5	NA^d^	NA^d^
10	4f	CoCl_2_	70	20	>98 : 2	>99.5 : 0.5
11	4f	CoBr_2_	70	5	>98 : 2	>99.5 : 0.5
12	4f	CoI_2_	60	8	>98 : 2	>99.5 : 0.5
13	4f	CoI_2_	80	27	>98 : 2	>99.5 : 0.5
14^e^	4f	CoI_2_	70	79	>98 : 2	>99.5 : 0.5
15^f^	4f	CoI_2_	70	<5	NA^d^	NA^d^
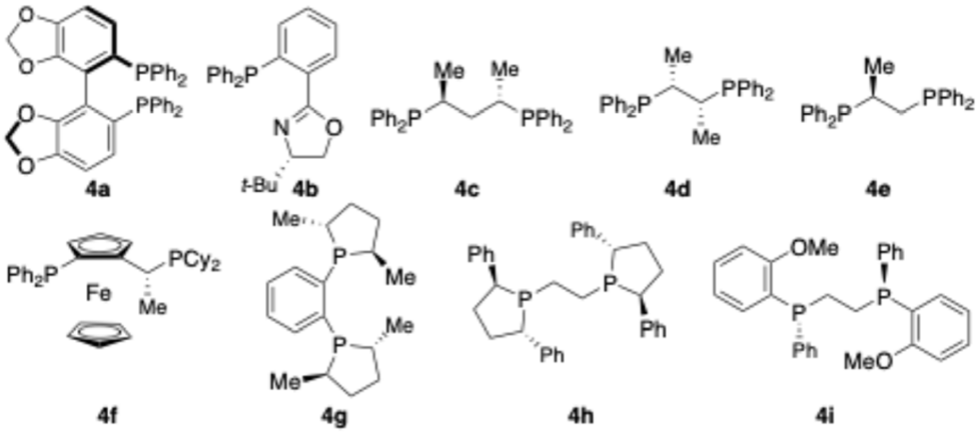						

a Yield of a mixture of diastereomers isolated.

b Determined by analysis of ^1^H NMR spectra of unpurified mixtures.

c Determined by analysis of HPLC spectra.

d Not available.

e Reaction was performed with 6.0 equiv. of Zn.

f Reaction was conducted with 2.0 mol% 4-CzIPN, 2.0 equiv. i-Pr_2_NEt and 3.0 equiv. Hantzsch ester instead of CoI_2_ irradiated with 40 W blue LEDs. DMA, *N*,*N*-dimethylacetamide.

**Scheme 2:**
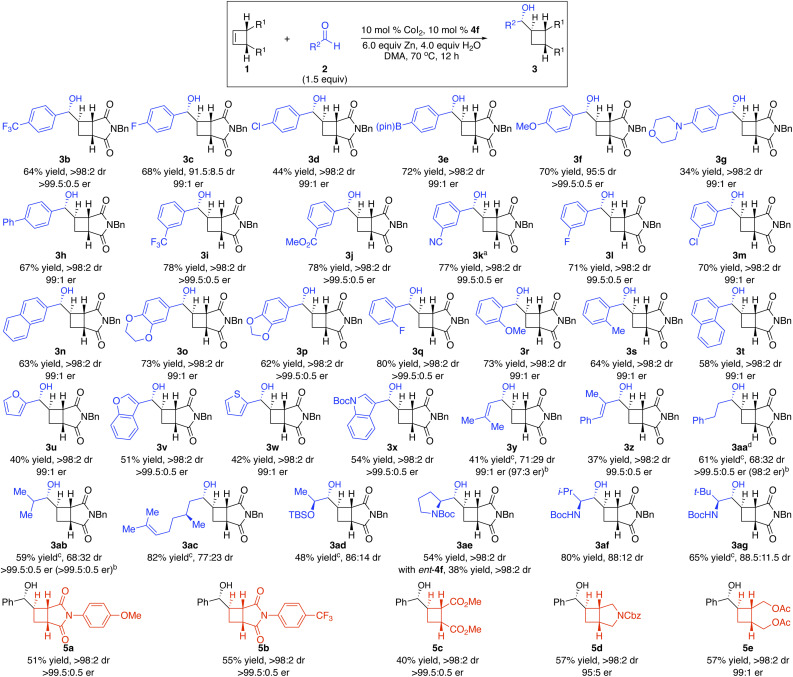
Scope for Co-catalyzed diastereo- and enantioselective reductive coupling of cyclobutenes and aldehydes. ^*a*^The reaction was performed at 90 °C. ^*b*^Enantioselectivity of the minor diastereomer. ^*c*^Yield of a mixture of diastereomers isolated. ^*d*^The reaction was performed in the presence of 7.5 mol% CoI_2_ and 7.5 mol% 4f.

Under the optimal conditions, we investigated the scope of Co-catalyzed diastereo- and enantioselective reductive coupling of cyclobutenes and aldehydes (Scheme 2). Aldehydes that contain electron-deficient (3b–c, 3i–l, and 3q), electron-rich (3f–h, 3n–p, and 3r), halogenated (3c–d, 3l–m, and 3q), and sterically congested (3q–t) aryls underwent the diastereo- and enantioselective reductive coupling, producing enantioenriched cyclobutanes bearing four stereogenic centers in 34–80% yield with 95 : 5–>98 : 2 dr and 99 : 1–>99.5 : 0.5 er. Functional groups, such as boronate (3e), cyano (3k) and ester (3j), are compatible with the reaction. Heteroaryl aldehydes (3u–x) served as suitable substrates that furnished desired products in 40–54% yield with 99 : 1–>99.5 : 0.5 er as a single diastereomer. α,β-Unsaturated aldehyde can be transformed into enantioenriched cyclobutane 3y with 71 : 29 dr, and high enantioselectivity was obtained for each diastereomer. Reaction of a more sizable α,β-unsaturated aldehyde afforded 3z with higher diastereoselectivity. Aliphatic aldehydes (3aa–3ab) were able to participate in the transformation, albeit with lower diastereoselectivity (68 : 32 dr). Enantioenriched aldehydes could be converted into densely functionalized cyclobutanes in high diastereoselectivity (3ac–3ag). In particular, the stereochemistry can be solely controlled by the chiral catalyst (3af). Transformations of cyclobutenes containing aryl-substituted succinimide delivered enantioenriched cyclobutanes (5a–b) in 51–55% yield with >99.5 : 0.5 er as a single diastereomer. Unlike our previous Co-catalyzed enantioselective functionalization of cyclobutenes and the Pd-catalyzed protocol that the succinimide moiety was necessary for the reactivity,^19,20,28^ a variety of cyclobutenes bearing diester (5c), fused cyclic amide (5d), and protected diol (5e) were able to undergo the reductive coupling reactions in 40–57% yield with 95 : 5–>99.5 : 0.5 er as a single diastereomer.

**Scheme 3:**
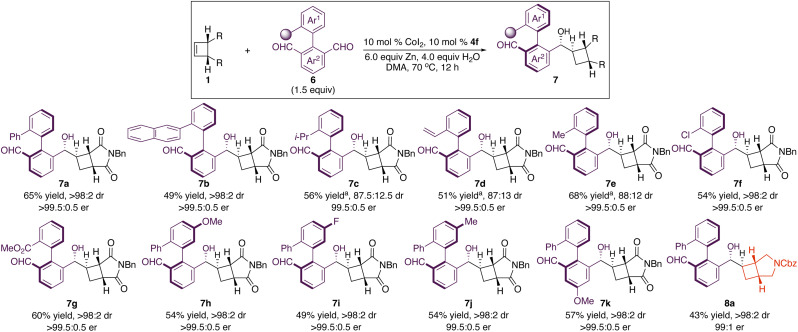
Application to the simultaneous construction of central and axial stereogenicity. ^*a*^Yield of a mixture of diastereomers isolated.

Molecules with axial stereogenicity have attracted increasing attention due to their importance in catalysis, complex molecule synthesis and materials science.^29^ Development of new catalytic approaches for enantioselective access to atropisomers is sought after. In particular, it is challenging for simultaneous establishment of central and axial stereogenicity in a single step and accurate control of diastereo- and enantioselectivity in the process that generates multiple stereogenic elements. We applied our cobalt-catalyzed approach to desymmetrization of dialdehydes for simultaneous construction of central and axial stereogenicity (Scheme 3).^30^ A broad range of dialdehydes containing various substituents and functional groups underwent the reductive coupling reactions, producing enantioenriched cyclobutanes (7a–k) bearing four central and one axial stereogenic centers in 49–68% yield with 87 : 13–>98 : 2 dr and 99.5 : 0.5–>99.5 : 0.5 er. Cyclobutenes in the absence of the succinimide moiety were able to participate in the transformation, delivering the desired product 8a in 43% yield with 99 : 1 er as a single diastereomer. It is worth mentioning that the styrene moiety remained intact in the reaction (7d).

**Scheme 4:**
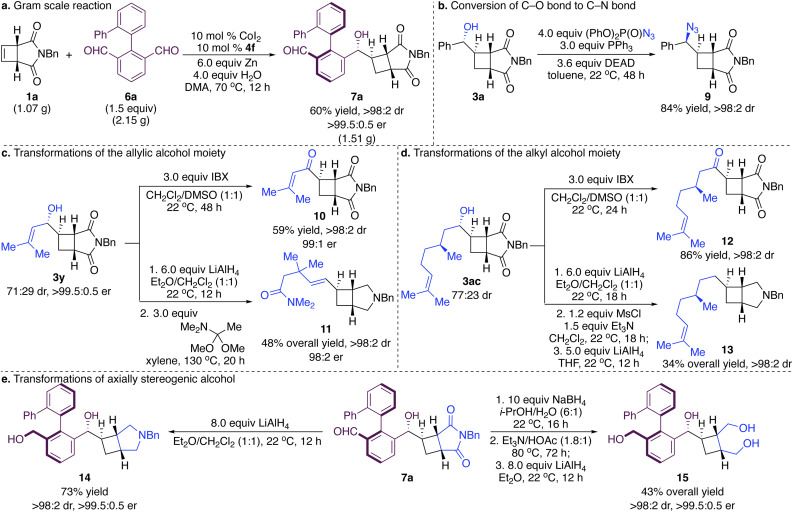
Gram scale reaction and functionalization.

The reaction can be performed on a gram scale (Scheme 4a). Reaction of cyclobutene 1a (1.07 g) with dialdehyde 6a (2.15 g) promoted by the Co complex derived from 4f afforded cyclobutane 7a in 60% yield as a single diastereo- and enantiomer. The stereogenic C–O bond was transformed into C–N bonds by the Mitsunobu reaction with complete inversion of the stereochemistry in 84% yield (Scheme 4b).^31^ Oxidation of the allylic alcohol side chain provided ketone 10 that cannot be accessed through hydroacylation in 59% yield with 99 : 1 er as a single diastereomer (Scheme 4c).^32^ Reduction of the succinimide followed by Eschenmoser–Claisen rearrangement of the allylic alcohol side chain delivered densely functionalized alkenyl cyclobutene 11 in 48% overall yield with 98 : 2 er as a single diastereomer (Scheme 4c).^33^ Similarly, treatment of 3ac generated from reaction with enantioenriched aldehyde with IBX furnished hydroacylation product 12 in 86% yield as a single diastereomer (Scheme 4d).^32^ Reduction of the succinimide followed by mesylation of the alcohol and hydride substitution supplied hydroalkylation product 13 that is otherwise difficult to access in 34% yield over three steps as a single diastereomer (Scheme 4d). The succinimide moiety can be reduced by LiAlH_4_ with simultaneous reduction of the aldehyde to form diol 14 in 73% yield as a single diastereo- and enantiomer (Scheme 4e). Because the cyclobutene bearing two free alcohol side chains cannot participate in the reaction, a multi-step sequence was conducted to convert the succinimide into diol, generating multifunctional cyclobutane 15 in 43% overall yield as a single diastereo- and enantiomer (Scheme 4e).^34^

**Scheme 5:**
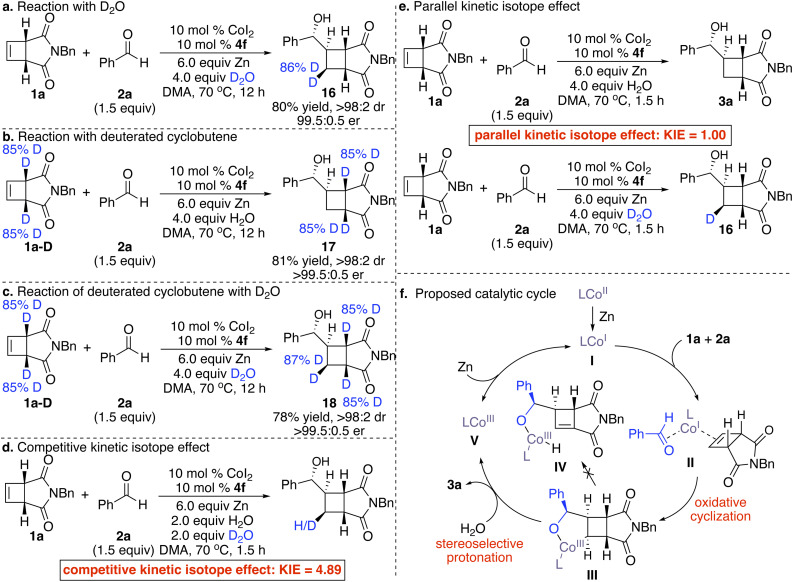
Mechanistic studies and the proposed catalytic cycle.

To gain some preliminary insight into the reaction mechanism, a series of experiments were performed (Scheme 5). Treatment of cyclobutene 1a and benzaldehyde 2a with D_2_O afforded deuterated cyclobutane 16 in 80% yield with 99.5 : 0.5 er as a single diastereomer, suggesting that protonation of the stereogenic C–Co bond in the metallacycle (III → V, Scheme 5f) formed from oxidative cyclization was stereoretentive (Scheme 5a). Reaction of deuterated cyclobutene 1a-D with benzaldehyde 2a promoted by the Co complex derived from 4f delivered deuterated cyclobutane 17 in 81% yield as a single diastereo- and enantiomer, implying that unlike previous Rh- and Pd-catalyzed processes^18*a*,19^ and similar to Co-catalyzed reactions of cyclobutenes with allenes developed by our group,^28^ β-hydride elimination and chain walking on the four-membered scaffold (III → IV, Scheme 5f) didn't occur (Scheme 5b). Transformation of deuterated cyclobutene 1a-D in the presence of D_2_O furnished cyclobutane 18 bearing three deuterated stereogenic centers in 78% yield as a single diastereomer and enantiomer (Scheme 5c). A variety of enantioenriched cyclobutanes containing deuterated stereogenic centers at different sites could be accessed by various combinations of deuterated substrates and reagents. Competitive kinetic isotope effect experiments suggested that protonation was irreversible (Scheme 5d). Parallel kinetic isotope effect experiments indicated that protonation of the C–Co bond might not be the rate-determining step (Scheme 5e).

Based on all the observations above, we proposed the possible catalytic cycle (Scheme 5f). Reduction of the Co^II^ precursor by Zn generated Co^I^ complex I, which coordinated with cyclobutene 1a and benzaldehyde 2a and underwent diastereo- and enantioselective oxidative cyclization to form metallacycle III. Further protonation of metallacycle III with H_2_O released the reductive coupling product 3a. Possible β-hydride elimination was not observed. Reduction of Co^III^ species V by Zn regenerated Co^I^ complex I.

**Scheme 6:**
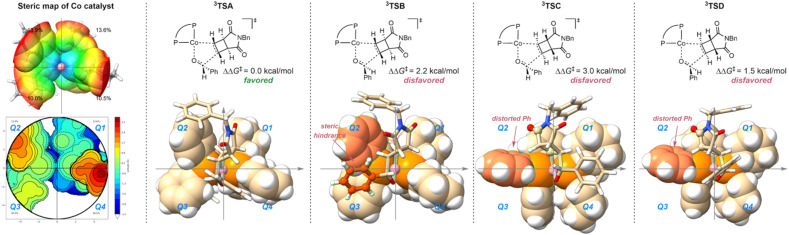
DFT calculations for stereo-determining transition states. A topographic steric map of the Co catalyst as well as transition states with different configurations is depicted. 4f used in experiments was employed in our calculations.

To elucidate the origin of stereoselectivity, we performed density functional theory (DFT) calculations for the oxidative cyclization step (Scheme 6). Considering the well-documented multi-state reactivity characteristic of first-row transition metal complexes,^28,35,36^ we systematically examined both triplet and singlet spin states for all possible transition state configurations (Fig. S1). Our calculations revealed that the triplet pathways consistently exhibited lower energy barriers compared to their singlet counterparts (Fig. S2), prompting us to focus on the structure of triplet TSs to investigate the stereo-induction mode. The topographic steric map analysis (Scheme 6) demonstrates that quadrants Q1 and Q3 of the cobalt catalyst are less congested.^37^ In the energetically most favorable transition state ^3^TSA, consistently reported by experiments and computations, the substrate adopts an optimal orientation where the cyclobutene moiety occupies Q1, while the aldehyde's phenyl group avoids steric clashes by extending away from the catalyst cavity. This contrasts with ^3^TSB, where destabilizing steric repulsions arise between the aldehyde's phenyl group and the congested Q2 region of the ligand framework. Furthermore, in both ^3^TSC and ^3^TSD, the cyclobutene fragments orient towards steric demanding Q2, enforcing significant geometric distortion of the ligand's phenyl group, resulting in higher energy barriers.

## Conclusions

In conclusion, we have developed the first cobalt-catalyzed protocol for diastereo- and enantioselective reductive coupling of nonpolar cyclobutenes and aldehydes through an oxidative cyclization pathway. Such a process enabled umpolung reactivity of cyclobutenes and incorporation of a functionalized chiral alkyl fragment as well as simultaneous establishment of multiple stereogenic centers at both the four-membered cyclic core and side chain. A broad range of enantioenriched densely functionalized cyclobutanes that are otherwise difficult to access can be furnished from easily accessible starting materials and catalysts based on an earth-abundant sustainable transition metal and commercially available ligand with high efficiency and stereoselectivity. Functionalization and application to the simultaneous construction of central and axial stereogenicity as well as deuterated stereogenic centers delivered a variety of useful chiral building blocks. Mechanistic studies revealed that the reaction proceeded through diastereo- and enantioselective oxidative cyclization followed by stereoselective protonation of the stereogenic C–Co bond. Possible β-hydride elimination didn't occur. DFT calculations elucidated the origin of the stereoselectivity. Such discoveries unveiled a novel reaction pathway for cobalt catalysis, opening up new opportunities for designing new cobalt-catalyzed reactions and pushing forward the frontier of organocobalt chemistry.

## Author contributions

ZZ, QC and FM directed the project and prepared the manuscript. CL and JZ performed the experiments. ZZ conducted the DFT calculations.

## Conflicts of interest

There are no conflicts to declare.

## Supplementary Material

SC-OLF-D5SC03755G-s001

SC-OLF-D5SC03755G-s002

## Data Availability

The data supporting this article have been included as part of the SI. CCDC 2373832 and 2373833 contain the supplementary crystallographic data for this paper.^38,39^ The experimental procedures, characterization data, NMR spetra and HPLC traces. See DOI: https://doi.org/10.1039/d5sc03755g.
