# Supplementary material for: Cobalt-catalyzed diastereo- and enantioselective reductive coupling of cyclobutenes and aldehydes through umpolung reactivity
Source: Chem Sci. 2025 Aug 4;16(35):16250–8. doi: 10.1039/d5sc03755g (PMC12352666; doi:10.1039/d5sc03755g)
Supplement: SC-016-D5SC03755G-s001 [file SC-016-D5SC03755G-s001.pdf]

Supplementary Information (SI) for Chemical Science.
This journal is © The Royal Society of Chemistry 2025

Cobalt-Catalyzed Diastereo- and Enantioselective Reductive Coupling of Cyclobutenes and Aldehydes through Umpolung Reactivity

Chuiyi Lin,[1] Jiwu Zhang,[1] Zhihan Zhang,[2,*] Qinglei Chong,[1,*] and Fanke Meng[1,3,4,*]

[1]State Key Laboratory of Organometallic Chemistry, Center for Excellence in Molecular Synthesis, Shanghai Institute of Organic Chemistry, University of Chinese Academy of Sciences, 345 Lingling Road, Shanghai, 200032, China.
[2]College of Chemistry, Central China Normal University, 152 Louyu Road, Wuhan, Hubei, 430079, China.
[3]School of Chemistry and Materials Science, Hangzhou Institute for Advanced Study, University of Chinese Academy of Sciences, 1 Sub-lane Xiangshan, Hangzhou, 310024, China.
[4]Beijing National Laboratory for Molecular Sciences.

*E-mail: zhihanzhang@ccnu.edu.cn, chongql@sioc.ac.cn, mengf@sioc.ac.cn

Supporting Information

Table of Contents

General Information…………………………….……………………………………....S3

Reagents and Starting Substrates ……………………………………………….. …..S4

Additional Optimization of The Reaction Conditions………………...………… S4-S8

Representative Experimental Procedure for synthesis of **3a**..………………………… S9

Characterization of Products…………………………………….………………S10-S61

Experimental Procedure and Characterization of Functionalization……..........S62-S72

Mechanistic Studies...……... …………………………………………… S73-S100

Proof of Stereochemistry: X-ray Characterization Data…………………. S101- S125

Supplementary Reference………………..………………..……………….... S126-S127

NMR Spectra……………………………………………………….……….....S128–S195

■ General Information

Infrared (IR) spectra were recorded on a BRUKER TENSOR 27 FT-IR spectrometer, λ_{max} in cm^{-1}. Bands are characterized as broad (br), strong (s), medium (m), and weak (w). ^{1}H NMR spectra were recorded on a Bruker 400 MHz, Agilent 400 MHz or Varian 400 MHz spectrometers. Chemical shifts are reported in ppm with the solvent resonance as the internal standard ($CDCl_3$: δ 7.26 ppm), or tetramethylsilane as internal standard (TMS: δ 0.00 ppm). Data are reported as follows: chemical shift, integration, multiplicity (s = singlet, d = doublet, t = triplet, q = quartet, m = multiplet), and coupling constant (Hz). ^{13}C NMR spectra were recorded on a Bruker 100 MHz Agilent 100 MHz or Varian 100 MHz spectrometers with complete proton decoupling. Chemical shifts are reported in ppm from tetramethylsilane with the solvent resonance as the internal standard ($CDCl_3$: δ 77.16 ppm). EI-HRMS and ESI-HRMS spectra were obtained on a Waters Premier GC-TOF MS and a Thermo Scientific Q Exactive HF Orbitrap-FTMS, respectively. FI-HRMS and DART-HRMS spectra were obtained on a JEOL-AccuTOF-GCv4G-GCT MS and a Thermo Fisher Scientific LTQ FTICR-MS, respectively. Enantiomer ratios were determined by high-performance liquid chromatography (HPLC) or supercritical fluid chromatography (SFC) from Shimadzu Corporation. (Chiralpak IA (4.6 x 250 mm), Chiralpak IB (4.6 x 250 mm), Chiralpak IC (4.6 x 250 mm), Chiralpak ID (4.6 x 250 mm), Chiralpak IE (4.6 x 250 mm), Chiralpak IF (4.6 x 250 mm), Chiralpak IG (4.6 x 250 mm), Chiralpak IB N-5 (4.6 x 250 mm), Chiralpak AS-H (4.6 x 250 mm), Chiralpak AZ-H (4.6 x 250 mm), Chiralpak AD-H (4.6 x 250 mm)) in comparison with authentic racemic materials. Specific rotations were measured on a Rudolph Research Analytical Autopol VI Polarimeter and Autopol I Polarimeter. Melting points were measured on a RY-I apparatus and uncorrected. Unless otherwise noted, all reactions were carried out with distilled and degassed solvents under an atmosphere of dry N_2 in oven- (135 °C) or flame-dried glassware with standard dry box or vacuum-line techniques. Anhydrous THF (J&K Chemicals Inc. and used as received.) were used without further purification. All work-up and purification procedures were carried out with reagent grade solvents (purchased from Adamas Reagent, Ltd.) in air.

■ Reagents and Starting Substrates

Cobalt salts, ligands: purchased from Strem Chemicals Inc. and used as received.

Aldehydes: amino aldehydes prepared according to a previous reported procedure[1], other monoaldehydes purchased from TCI Chemicals Inc. and purified by flash column chromatography or distillation before used, dialdehydes prepared according to a previous reported procedure[2].

Cyclobutenes: prepared according to a previous reported procedure[3].

■ Additional Optimization of the Reaction Conditions

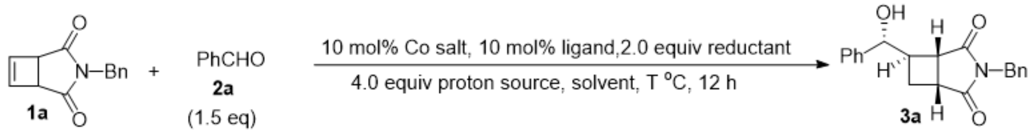

In a N_2-filled glove-box, an oven-dried vial (8 mL) equipped with a magnetic stir bar was charged with Co salt (0.01 mmol, 10 mol %), ligand (0.01 mmol, 10 mol %) and reductant (0.2 mmol, 2.0 equiv.). Solvent (1 mL) was added, then the mixture was allowed to stir at room temperature for 30 min. **1a** (21.3 mg, 0.1 mmol, 1.0 equiv.), **2a** (15.9 mg, 0.15 mmol, 1.5 equiv.) and proton source (0.4 mmol, 4.0 equiv.) were added to the solution. The vial was sealed with a cap (phenolic open top cap with red PTFE/white silicone septum), twined with adhesive tape and taken out of the glove box. It was allowed to stir at correspond temperature for 12 h.

Workup: Upon completion, the reaction was quenched by 50 mL H_2O and extracted with diethyl ether (3*20 mL). The combined organic layer was washed with brine (10 mL) and dried over Na_2SO_4. After the solids were filtered off, the solvent was removed under reduced pressure and the residue was purified by silica-gel column chromatography (eluent: Petroleum ether/ EtOAc = 3:1) to afford **3a**.

Table S1. Screen of chiral ligands for reaction of 1a and 2a

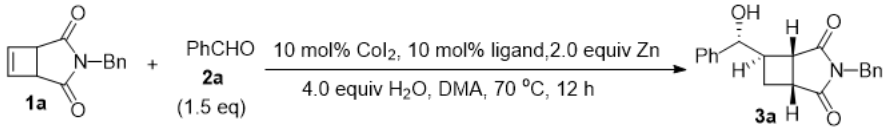

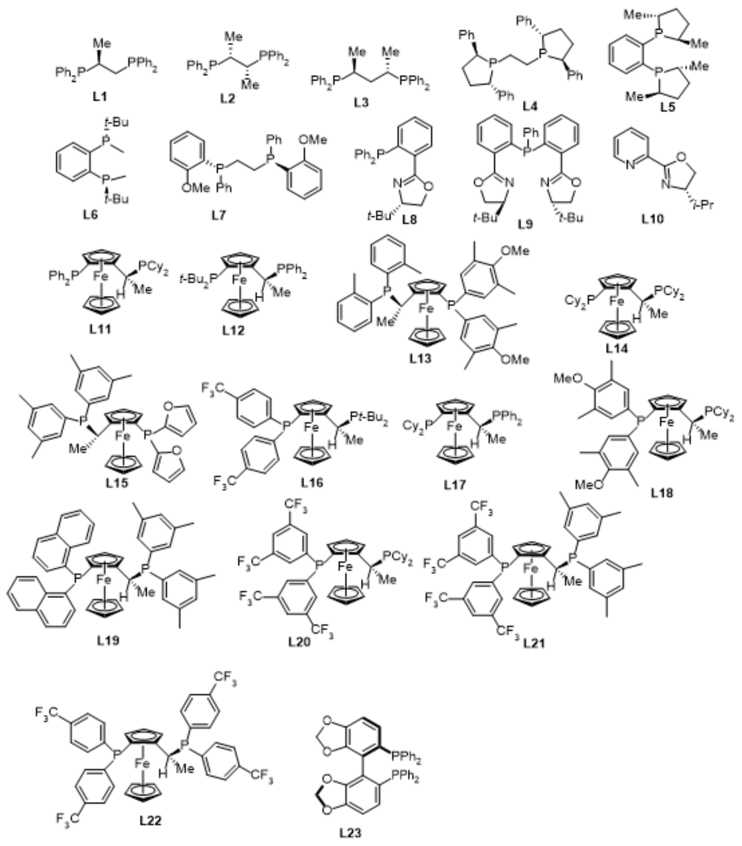

entry	ligand	yield(%)[a]	dr[b]	er(major)[c]	er(minor)[d]
1	**L1**	63	1:1.2	14:86	59:41
2	**L2**	<5	NA[e]	NA	NA
3	**L3**	<5	NA	NA	NA
4	**L4**	48	2.1:1	27:73	>99.5:0.5
5	**L5**	<5	NA	NA	NA
6	**L6**	<5	NA	NA	NA
7	**L7**	<5	NA	NA	NA
8	**L8**	<5	NA	NA	NA
9	**L9**	<5	NA	NA	NA
10	**L10**	<5	NA	NA	NA
11	**L11**	68	7.0:1	>99.5:0.5	NA
12	**L12**	<5	NA	NA	NA
13	**L13**	<5	NA	NA	NA
14	**L14**	<5	NA	NA	NA
15	**L15**	<5	NA	NA	NA
16	**L16**	<5	NA	NA	NA
17	**L17**	<5	NA	NA	NA
18	**L18**	<5	NA	NA	NA
19	**L19**	<5	NA	NA	NA
20	**L20**	<5	NA	NA	NA
21	**L21**	<5	NA	NA	NA
22	**L22**	<5	NA	NA	NA
23	**L23**	**<5**	NA	NA	NA

[a] Isolated yield, total yield. [b] Determined by analysis of ^{1}H NMR spectra of unpurified mixture. [c,d] The enantiomeric excess of **3a** and its diastereoisomer were determined by HPLC with a chiral column. [e] NA: not available.

Table S2. Screen of temperature for reaction of 1a and 2a

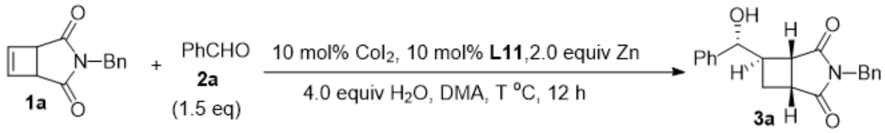

entry	T/ °C	yield(%)[a]	dr[b]	er(major)[c]
1	50	8%	8.7:1	>99.5:0.5
2	60	8%	>20:1	>99.5:0.5
3	80	27%	>20:1	>99.5:0.5
4	90	53%	>20:1	>99.5:0.5

[a] Isolated yield, total yield. [b] Determined by analysis of ^{1}H NMR spectra of unpurified mixture. [c] The enantiomeric excess of **3a** was determined by HPLC with a chiral column.

Table S3. Screen of cobalt salts for reaction of 1a and 2a

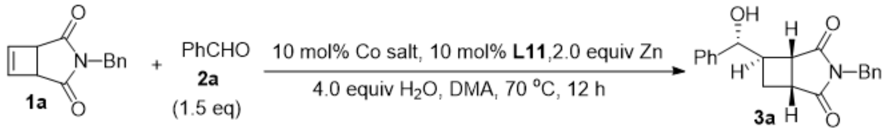

entry	Co-salt	yield(%)[a]	dr[b]	er[c]
1	$CoCl_2$	20%	>20:1	>99.5:0.5
2	$CoBr_2$	5%	>20:1	>99.5:0.5
3	$Co(hfac)_2$	<5%	NA[d]	NA
4	$Co(OAc)_2$	<5%	NA	NA
5	$Co(BF_4)_2$ ·	<5%	NA	NA
6	$Co(acac)_2$	<5%	NA	NA
7	$Co(OTf)_2$	<5%	NA	NA

[a] Isolated yield. [b] Determined by analysis of ^{1}H NMR spectra of unpurified mixture.

[c] The enantiomeric excess of **3a** was determined by HPLC with a chiral column. [d] NA: not available.

Table S4. Screen of solvents for reaction of 1a and 2a

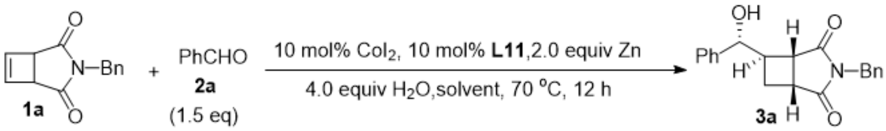

entry	proton source	yield(%)[a]	dr[b]	er[c]
1	DCE	<5%	NA[d]	NA
2	THF	<5%	NA	NA
3	DME	<5%	NA	NA
4	DMF	<5%	NA	NA
5	DMSO	<5%	NA	NA
6	CH_3CN	<5%	NA	NA
7	NMP	36%	>20:1	>99.5:0.5

[a] Isolated yield. [b] Determined by analysis of ^{1}H NMR spectra of unpurified mixture.

[c] The enantiomeric excess of **3a** was determined by HPLC with a chiral column. [d] NA: not available.

Table S5. Screen of proton source for reaction of 1a and 2a

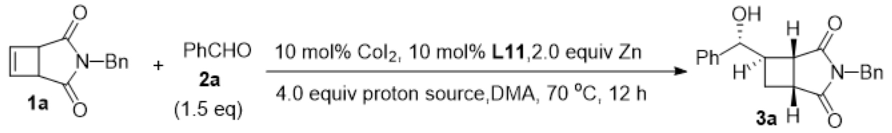

entry	proton source	yield(%)[a]	dr[b]	er[c]
1	MeOH	<5%	NA[d]	NA
2	EtOH	25%	14.0:1	>99.5:0.5
3	*i*-PrOH	9%	>20:1	>99.5:0.5

[a] Isolated yield. [b] Determined by analysis of ^{1}H NMR spectra of unpurified mixture.

[c] The enantiomeric excess of **3a** was determined by HPLC with a chiral column. [d]NA: not available.

Table S6. Screen of reductant for reaction of 1a and 2a

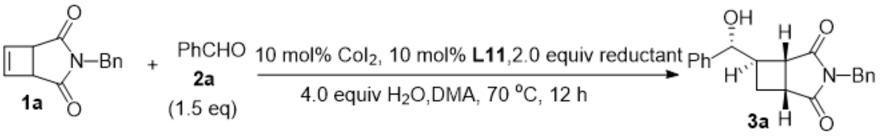

entry	reductant	yield(%)[a]	dr[b]	er[c]
1	Mn	<5%	NA[d]	NA
2	In	<5%	NA[d]	NA

[a] Isolated yield. [b] Determined by analysis of ^{1}H NMR spectra of unpurified mixture.

[c] The enantiomeric excess of **3a** was determined by HPLC with a chiral column. [d] NA: not available.

Table S7. Screen of the equiv. of Zn for reaction of 1a and 2a

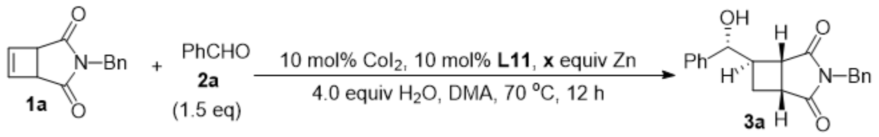

entry	x	yield(%)[a]	dr[b]	er(major)[c]
1	1.5	33%	8.1:1	>99.5:0.5
2	4	73%	>20:1	>99.5:0.5
3	6	79%	>20:1	>99.5:0.5

[a] Isolated yield. [b] Determined by analysis of ^{1}H NMR spectra of unpurified mixture.

[c] The enantiomeric excess of **3a** was determined by HPLC with a chiral column.

Table S8. Screen of the loading of the catalyst for reaction of 1a and 2a

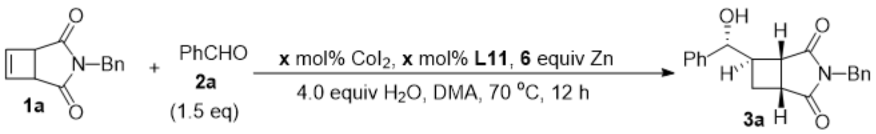

entry	x	yield(%)[a]	dr[b]	er(major)[c]
1	7.5	74%	>20:1	>99.5:0.5
2	5	40%	>20:1	>99.5:0.5
3	2.5	18%	>20:1	>99.5:0.5

[a] Isolated yield. [b] Determined by analysis of ^{1}H NMR spectra of unpurified mixture.

[c] The enantiomeric excess of **3a** was determined by HPLC with a chiral column.

■ Representative Experimental Procedure for synthesis of 3a

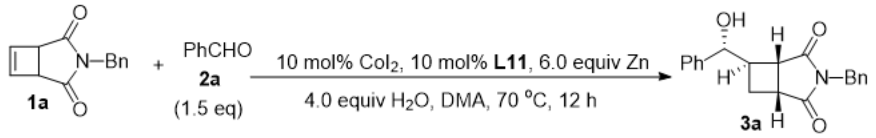

In a N_2-filled glove-box, an oven-dried vial (8 mL) equipped with a magnetic stir bar was charged with CoI_2 (0.02 mmol, 10 mol %), **L11** (0.02 mmol, 10 mol %) and Zn (1.2 mmol, 6.0 equiv.). DMA (2 mL) was added, then the mixture was allowed to stir at room temperature for 30 min. **1a** (42.6 mg, 0.2 mmol, 1.0 equiv.), **2a** (31.8 mg, 0.3 mmol, 1.5 equiv.) and H_2O (14.4 mg, 0.8 mmol, 4.0 equiv.) were added to the solution. The vial was sealed with a cap (phenolic open top cap with red PTFE/white silicone septum), twined with adhesive tape and taken out of the glove box. It was allowed to stir at 70 °C for 12 h.

Workup: Upon completion, the reaction was quenched by 50 mL H_2O and extracted with diethyl ether (3*20 mL). The combined organic layer was washed with brine (10 mL) and dried over Na_2SO_4. After the solids were filtered off, the solvent was removed under reduced pressure and the residue was purified by silica-gel column chromatography (eluent: Petroleum ether/ EtOAc = 3:1) to afford **3a**.

■ Characterization of Products

(1*R*,5*S*,6*R*)-3-benzyl-6-((*R*)-hydroxy(phenyl)methyl)-3-azabicyclo[3.2.0]heptane-2,4-dione (3a)

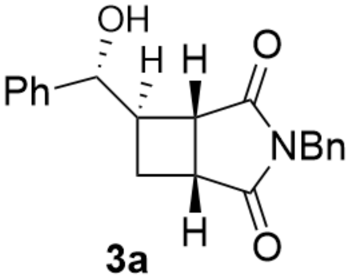

3a

milky oil; **IR (neat):** 3505.40 (br), 1759.18 (w), 1682.03 (s), 1546.34 (w), 1493.52 (w), 1452.02 (w), 1399.66 (m), 1362.41 (w), 1333.88 (w), 1300.79 (w), 1268.15 (m), 1194.08 (w), 1176.01 (w), 1163.65 (w), 1099.02 (w), 1066.97 (w), 1023.77 (w), 990.53 (m), 942.96 (w), 925.55 (w), 908.64 (w), 884.37 (w), 867.26 (w), 835.91 (m), 805.14 (m), 754.02 (w), 725.71 (w), 690.21 (m), 653.85 (m), 636.72 (m), 608.35 (w), 576.73 (w), 520.43 (w), 478.81 (w), 457.63 (m), 447.21 (w), 411.34 (w) cm^{-1}

^{1}H NMR (400 MHz, CDCl$_3$) δ 7.28-7.15 (m, 10 H), 4.59 (d, J = 8.0 Hz, 1 H), 4.56 (s, 2 H), 3.30 (dd, J = 6.8, 4.0 Hz, 1 H), 3.09-3.03 (m, 2 H), 2.61-2.54 (m, 1 H), 2.35-2.28 (m, 1 H), 1.99-1.92 (m, 1 H). **^{13}C NMR (100 MHz, CDCl$_3$)** δ 179.5, 178.9, 141.3, 136.0, 128.8, 128.7, 128.3, 128.0, 126.4, 76.1, 43.7, 42.6, 41.4, 35.7, 25.5.

HRMS (ESI$^+$) [M+Na]$^+$ Calcd for $C_{20}H_{19}NO_3Na$: 344.1257 m/z, Found: 344.1264 m/z;

Specific rotation: $[\alpha]_D^{28.6}$2.0 (c 0.50, $CHCl_3$) for an enantiomerically enriched sample of >99.5:0.5 e.r.

Enantiomeric purity of **3a** was determined by HPLC analysis in comparison with authentic racemic material (>99.5:0.5 e.r. shown; Chiralpak IF column, 90:10 hexane / ^{i}PrOH, 1.0 mL/min, 220 nm).

<Sample Information>

Sample Name : cyl-09012-5RAC
Sample ID :
Data Filename : cyl-09012-5-RACx-IF-90-10-1.0.lcd
Method Filename : cyl-6H-90-10-1.0ml-50min.lcm
Batch Filename : WWLL.lcb
Vial # : 1-91
Injection Volume : 20 uL
Date Acquired : 7/24/2024 21:10:49
Date Processed : 8/12/2024 10:43:30
Sample Type : Unknown
Acquired by : System Administrator
Processed by : System Administrator

<Chromatogram>

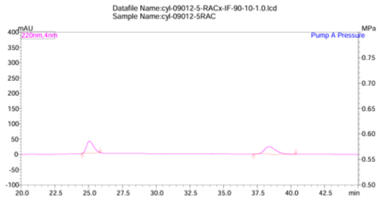

<Peak Table>

PDA Ch2 220nm

Peak#	Ret. Time	Area	Area%
1	25.052	1440307	50.421
2	38.388	1416278	49.579
Total		2856584	100.000

<Sample Information>

Sample Name : cyl-10066
Sample ID :
Data Filename : cyl-10066-IF-90-10-1.0.lcd
Method Filename : cyl-6H-90-10-1.0ml-50min.lcm
Batch Filename : WWLL.lcb
Vial # : 1-95
Injection Volume : 20 uL
Date Acquired : 7/24/2024 22:01:36
Date Processed : 7/24/2024 22:51:39
Sample Type : Unknown
Acquired by : System Administrator
Processed by : System Administrator

<Chromatogram>

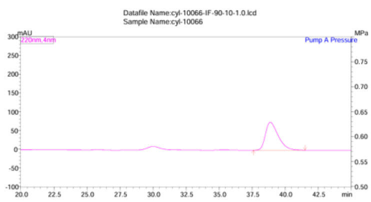

<Peak Table>

PDA Ch2 220nm

Peak#	Ret. Time	Area	Area%
1	38.882	4953922	100.000
Total		4953922	100.000

(1*R*,5*S*,6*R*)-3-benzyl-6-((*R*)-hydroxy(4-(trifluoromethyl)phenyl)methyl)-3-azabicyclo[3.2.0]heptane-2,4-dione (3b)

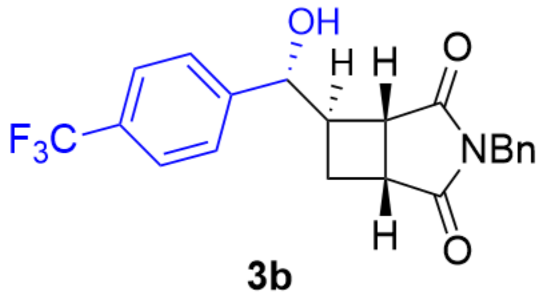

3b

white solid, m.p.175-176 °C; **IR (neat):** 3491.59 (br), 1758.69 (w), 1684.78 (s), 1618.71 (w), 1402.56 (m), 1365.26 (w), 1328.21 (s), 1274.11 (w), 1197.04 (w), 1163.81 (s), 1120.14 (m), 1107.11 (s), 1091.14 (s), 1068.53 (s), 1018.78 (s), 992.43 (s), 944.37 (m), 927.07 (m), 886.61 (w), 854.50 (w), 825.28 (w), 805.61 (w), 784.31 (w), 761.32 (w), 730.52 (w), 703.47 (m), 690.54 (m), 634.48 (s), 520.52 (w), 500.51 (w), 458.99 (w), 448.03 (w), 433.90 (w), 412.26 (w) cm^{-1};

^{1}H NMR (400 MHz, CDCl$_3$) δ 7.51 (d, *J* = 8.0 Hz, 2 H), 7.34 (d, *J* = 8.0 Hz, 2 H), 7.27-7.18 (m, 5 H), 4.68 (d, *J* = 7.2 Hz, 1 H), 4.56 (s, 2 H), 3.32-3.30 (m, 2 H), 3.13-3.08 (m, 1 H), 2.59-2.53 (m, 1 H), 2.39-2.31 (m, 1H), 2.03-1.96 (m, 1 H). **^{13}C NMR (125 MHz, CDCl$_3$)** δ 179.3, 178.7, 145.3, 135.9, 130.5 (t, *J* = 32.5 Hz), 128.8, 128.8, 128.1, 126.7, 125.8 (t, *J* = 3.8 Hz), 124.1 (t, *J* = 270.0 Hz), 75.4, 43.7, 42.7, 41.1, 35.7, 25.5. **^{19}F NMR (376 MHz, CDCl$_3$)** δ -62.55.

HRMS (ESI$^+$) [M+Na]$^+$ Calcd for $C_{21}H_{18}NO_3F_3Na$: 412.1131 m/z, Found: 412.1128 m/z;

Specific rotation: $[\alpha]_D^{27.7}$ -2.7 (*c* 0.50, CHCl$_3$) for an enantiomerically enriched sample of >99.5:0.5 e.r.

Enantiomeric purity of **3b** was determined by HPLC analysis in comparison with authentic racemic material (>99.5:0.5 e.r. shown; Chiralpak IB N-5 column, 90:10 hexane / ^{i}PrOH, 1.0 mL/min, 254 nm).

<Sample Information>

Sample Name : CYL-09035-2RAC
Sample ID :
Data Filename : CYL-09035-2RACX-IBN-5-90-10-1.0.lcd
Method Filename : ZJW-4-9010-1.0-50-1min.lcm
Batch Filename : ZJW.lcb
Vial # : 1-27 Sample Type : Unknown
Injection Volume : 20 uL
Date Acquired : 4/16/2024 8:27:41 PM Acquired by : System Administrator
Date Processed : 4/16/2024 9:07:51 PM Processed by : System Administrator

<Chromatogram>

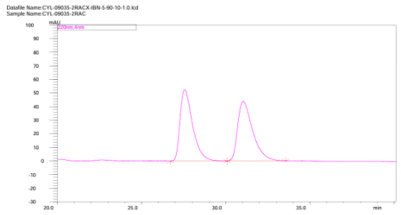

<Peak Table>

PDA Ch2 220nm

Peak#	Ret. Time	Area	Area%
1	27.559	2737340	50.096
2	31.027	2726879	49.904
Total		5464219	100.000

<Sample Information>

Sample Name : CYL-09038
Sample ID :
Data Filename : CYL-09038-IBN-5-90-10-1.0.lcd
Method Filename : ZJW-4-9010-1.0-50-1min.lcm
Batch Filename : ZJW.lcb
Vial # : 1-30 Sample Type : Unknown
Injection Volume : 20 uL
Date Acquired : 4/16/2024 7:37:07 PM Acquired by : System Administrator
Date Processed : 7/27/2024 9:00:16 AM Processed by : System Administrator

<Chromatogram>

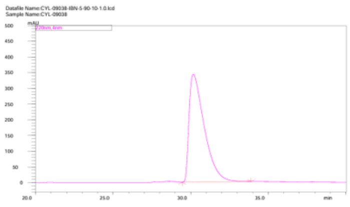

<Peak Table>

PDA Ch2 220nm

Peak#	Ret. Time	Area	Area%
1	30.170	23420315	100.000
Total		23420315	100.000

(1*R*,5*S*,6*R*)-3-benzyl-6-((*R*)-(4-fluorophenyl)(hydroxy)methyl)-3-azabicyclo[3.2.0]heptane-2,4-dione (3c)

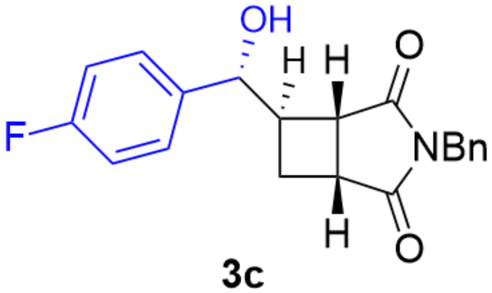

3c

white solid, m.p.188-189 °C; **IR (neat):** 3498.75 (br),1759.41 (w), 1684.01 (s), 1601.71 (w),1504.90 (w), 1401.71 (m), 1363.77 (m), 1336.05 (w), 1300.62 (w), 1216.91 (m), 1194.28 (m), 1177.10 (m), 1159.45 (m), 1100.18 (w), 1015.05 (m), 990.46 (m), 943.14 (w), 927.01 (w), 885.72 (w), 852.72 (w), 831.52 (w), 803.50 (m), 762.27 (m), 729.19 (m), 698.47 (m), 647.69 (m), 570.98 (w), 525.14 (w), 492.69 (w), 462.41 (w), 447.60 (w), 423.94 (w) cm^{-1};

^{1}H NMR (500 MHz, CD_3COCD_3) δ 7.48-7.43 (m, 2 H),7.32-7.23 (m, 5 H), 7.11-7.06 (m, 2 H), 4.85 (d, *J* = 5.0 Hz, 1 H), 4.83-4.81 (m, 1 H), 4.60 (s, 2 H), 3.44-3.42 (m, 1 H), 3.25-3.20 (m, 1 H), 2.88 (br, 1 H), 2.69-2.64 (m, 1 H), 2.56-2.50 (m, 1 H). **^{13}C NMR (125 MHz, CD_3COCD_3)** δ 180.0, 179.2, 162.9 (d, *J* =242.5 Hz), 140.2 (d, *J* =3.8 Hz), 137.8, 129.1 (d, *J* =7.5 Hz), 129.0 (d, *J* =71.3 Hz), 128.3, 115.7, 115.5, 74.8, 44.7, 42.6, 41.7, 36.6, 25.9. **^{19}F NMR (376 MHz, $CDCl_3$)** δ -113.67.

HRMS (ESI^{+}) [M+Na]$^{+}$ Calcd for $C_{20}H_{18}NO_3FNa$: 362.1163 m/z, Found: 362.1170 m/z;

Specific rotation: $[\alpha]_D^{23.8}$6.1 (*c* 0.50, $CHCl_3$) for an enantiomerically enriched sample of 99:1 e.r.

Enantiomeric purity of **3c** was determined by HPLC analysis in comparison with authentic racemic material (99:1 e.r. shown; Chiralpak IB N-5 column, 90:10 hexane / ^{i}PrOH, 1.0 mL/min, 254 nm).

<Sample Information>

Sample Name : cyl-09049-3
Sample ID :
Data Filename : cyl-09049-3NEWNEW-IBN-5-90-10-1.0.lcd
Method Filename : cyl-6hao-90-10-1.0ml-50min.lcm
Batch Filename : WWLL.lcb
Vial # : 1-76 Sample Type : Unknown
Injection Volume : 10 uL
Date Acquired : 9/9/2024 17:38:12 Acquired by : System Administrator
Date Processed : 9/9/2024 18:25:40 Processed by : System Administrator

<Chromatogram>

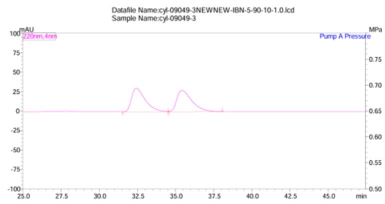

<Peak Table>

PDA Ch2 220nm

Peak#	Ret. Time	Area	Area%
1	32.431	1905951	50.140
2	35.421	1895304	49.860
Total		3801254	100.000

<Sample Information>

Sample Name : cyl-09041NEW
Sample ID :
Data Filename : cyl-09041NEW-IBN-5-90-10-1.0.lcd
Method Filename : cyl-6hao-90-10-1.0ml-150min.lcm
Batch Filename : WWLL.lcb
Vial # : 1-101 Sample Type : Unknown
Injection Volume : 10 uL
Date Acquired : 9/9/2024 16:44:33 Acquired by : System Administrator
Date Processed : 9/9/2024 18:29:40 Processed by : System Administrator

<Chromatogram>

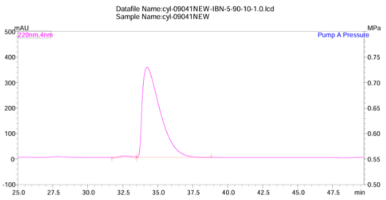

<Peak Table>

PDA Ch2 220nm

Peak#	Ret. Time	Area	Area%
1	32.635	313143	0.998
2	34.231	31050656	99.002
Total		31363798	100.000

(1*R*,5*S*,6*R*)-3-benzyl-6-((*R*)-(4-chlorophenyl)(hydroxy)methyl)-3-azabicyclo[3.2.0]heptane-2,4-dione (3d)

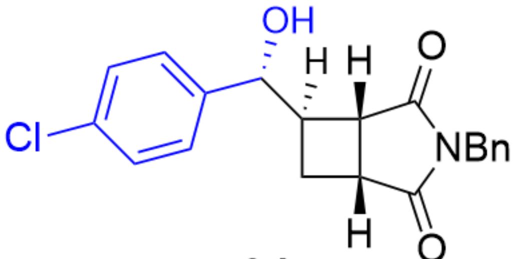

3d

white solid, m.p.183-184 °C; **IR (neat):** 3853.83 (w), 3734.24 (w), 3684.04 (br), 3667.63 (w), 2987.21 (m), 2972.85 (m), 2901.50 (m), 2336.49 (w), 2182.49 (w), 2164.93 (w), 1688.18 (w), 1451.57 (m), 1406.30 (w), 1252.01 (w), 1228.57 (w), 1066.98 (s), 1053.97 (s), 889.10 (w), 460.73 (w), 439.67 (w) cm^{-1};
^{1}H NMR (400 MHz, $CDCl_3$) δ 7.37-7.25 (m, 9 H), 4.71 (d, *J* = 6.8 Hz, 1 H), 4.66 (s, 2 H), 3.38 (dd, *J* = 6.8, 4.8 Hz, 1 H), 3.21-3.16 (m, 1 H), 2.65-2.59 (m, 1 H), 2.48-2.40 (m, 1 H), 2.12-2.04 (m, 1 H). **^{13}C NMR (125 MHz, CD_2Cl_2)** δ 179.5, 178.8, 140.6, 136.6, 134.1, 129.1, 129.0, 128.6, 128.13, 128.11, 75.5, 44.0, 42.7, 41.6, 36.0, 25.6.
HRMS (ESI$^+$) [M+Na]$^+$ Calcd for $C_{20}H_{18}NO_3NaCl$: 378.0867 m/z, Found: 378.0874 m/z;
Specific rotation: $[\alpha]_D^{27.5}$ -3.5 (*c* 0.50, $CHCl_3$) for an enantiomerically enriched sample of 99:1 e.r.
Enantiomeric purity of **3d** was determined by HPLC analysis in comparison with authentic racemic material (99:1 e.r. shown; Chiralpak IB N-5 column, 90:10 hexane / ^{i}PrOH, 1.0 mL/min, 254 nm).

<Sample Information>

Sample Name : cyl-09049-5
Sample ID :
Data Filename : cyl-09049-5NEWNEW-IBN-5-90-10-1.0.lcd
Method Filename : cyl-6hao-90-10-1.0ml-70min.lcm
Batch Filename : WWLL.lcb
Vial # : 1-64 Sample Type : Unknown
Injection Volume : 10 uL
Date Acquired : 9/11/2024 15:53:28 Acquired by : System Administrator
Date Processed : 9/11/2024 16:39:00 Processed by : System Administrator

<Chromatogram>

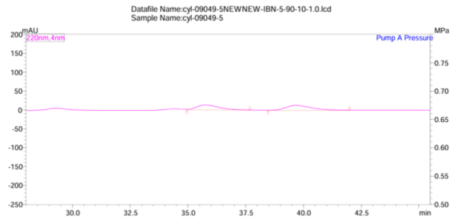

<Peak Table>

PDA Ch2 220nm

Peak#	Ret. Time	Area	Area%
1	35.762	986522	49.711
2	39.680	997999	50.289
Total		1984521	100.000

<Sample Information>

Sample Name : cyl-09042
Sample ID :
Data Filename : cyl-09042NEW-IBN-5-90-10-1.0.lcd
Method Filename : cyl-6hao-90-10-1.0ml-70min.lcm
Batch Filename : WWLL.lcb
Vial # : 1-64 Sample Type : Unknown
Injection Volume : 10 uL
Date Acquired : 9/11/2024 15:02:58 Acquired by : System Administrator
Date Processed : 9/11/2024 15:52:22 Processed by : System Administrator

<Chromatogram>

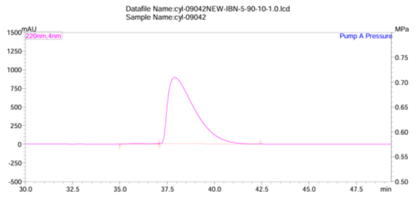

<Peak Table>

PDA Ch2 220nm

Peak#	Ret. Time	Area	Area%
1	35.834	745102	0.841
2	37.901	87812306	99.159
Total		88557408	100.000

(1*R*,5*S*,6*R*)-3-benzyl-6-((*R*)-hydroxy (4-(4,4,5,5-tetramethyl-1,3,2-dioxaborolan-2-yl)phenyl)methyl)-3-azabicyclo[3.2.0]heptane-2,4-dione (3e)

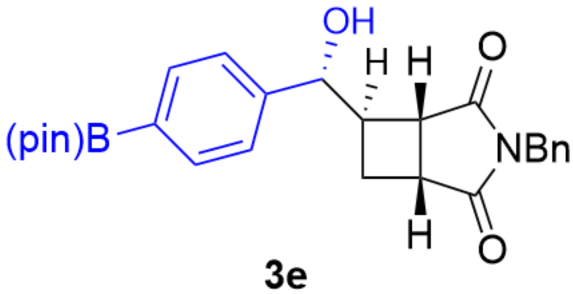

3e

milky oil; **IR (neat):** 3485.95 (br), 2976.70 (w), 2924.54 (w), 1759.94 (w), 1691.44 (s), 1610.54 (w), 1421.47 (s), 1391.99 (s), 1357.49 (s), 1327.55 (m), 1287.48 (m), 1262.07 (m), 1175.29 (m), 1164.03 (m), 1140.90 (m), 1090.36 (m), 1068.94 (m), 1048.55 (m), 1015.40 (m), 990.13 (w), 962.31 (w), 931.00 (m), 908.29 (m), 855.71 (m), 824.03 (m), 804.73 (m), 733.96 (m), 717.48 (m), 690.62 (m), 674.44 (s), 656.41 (m), 624.48 (w), 573.40 (w), 536.44 (w), 458.15 (w), 425.76 (w) cm^{-1};
^{1}H NMR (400 MHz, $CDCl_3$) δ7.80-7.78 (m, 2 H), 7.36-7.23 (m, 7 H), 4.71 (d, *J* = 7.2 Hz, 1 H), 4.64 (s, 2 H), 3.39 (dd, *J* = 7.2, 4.8 Hz, 1 H), 3.18-3.13 (m, 1 H), 2.83 (br, 1 H), 2.69-2.62 (m, 1 H), 2.46-2.38 (m, 1 H), 2.07-2.01 (m, 1 H), 1.33 (s, 1 H). **^{13}C NMR (100 MHz, $CDCl_3$)** δ 179.5, 178.7, 144.4, 136.0, 135.3, 128.78, 128.76, 128.0, 125.7, 84.0, 76.1, 43.7, 42.6, 41.4, 35.7, 25.5, 24.99, 24.97.
HRMS (ESI$^+$) [M+Na]$^+$ Calcd for $C_{26}H_{30}BNO_5Na$: 470.2109 m/z, Found: 470.2109 m/z; **Specific rotation:** $[\alpha]_D^{28.1}$ -4.3 (*c* 0.50, $CHCl_3$) for an enantiomerically enriched sample of 99:1 e.r.
Enantiomeric purity of **3e** was determined by HPLC analysis in comparison with authentic racemic material (99:1 e.r. shown; Chiralpak IB N-5 column, 90:10 hexane / ^{i}PrOH, 1.0 mL/min, 220 nm).

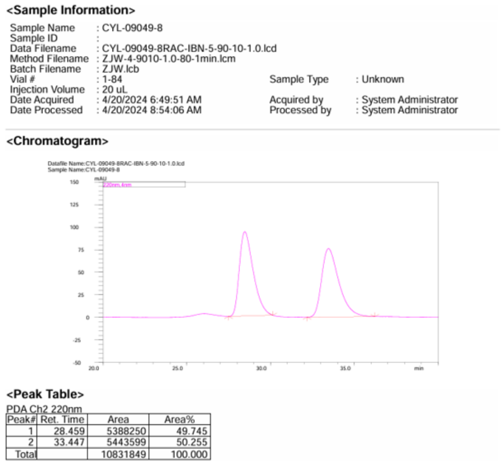
<Sample Information>

Sample Name : CYL-09049-8
Sample ID :
Data Filename : CYL-09049-8RAC-IBN-5-90-10-1.0.lcd
Method Filename : ZJW-4-9010-1.0-80-1min.lcm
Batch Filename : ZJW.lcb
Vial # : 1-84
Injection Volume : 20 uL
Date Acquired : 4/20/2024 6:49:51 AM
Date Processed : 4/20/2024 8:54:06 AM
Sample Type : Unknown
Acquired by : System Administrator
Processed by : System Administrator

<Chromatogram>

<Peak Table>

PDA Ch2 220nm

Peak#	Ret. Time	Area	Area%
1	28.459	5388250	49.745
2	33.447	5443599	50.255
Total		10831849	100.000

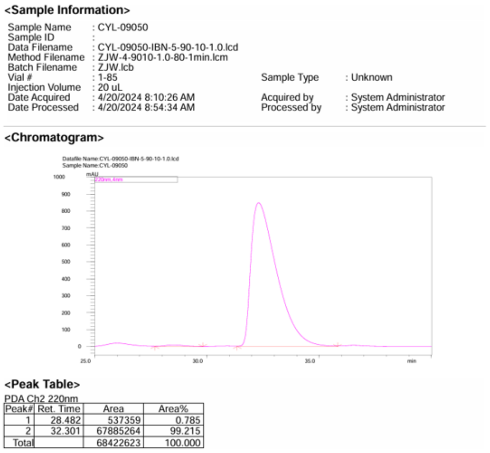
<Sample Information>

Sample Name : CYL-09050
Sample ID :
Data Filename : CYL-09050-IBN-5-90-10-1.0.lcd
Method Filename : ZJW-4-9010-1.0-80-1min.lcm
Batch Filename : ZJW.lcb
Vial # : 1-85
Injection Volume : 20 uL
Date Acquired : 4/20/2024 8:10:26 AM
Date Processed : 4/20/2024 8:54:34 AM
Sample Type : Unknown
Acquired by : System Administrator
Processed by : System Administrator

<Chromatogram>

<Peak Table>

PDA Ch2 220nm

Peak#	Ret. Time	Area	Area%
1	28.482	537359	0.785
2	32.301	67885264	99.215
Total		68422623	100.000

(1*R*,5*S*,6*R*)-3-benzyl-6((*R*)-hydroxy(4-methoxyphenyl)methyl)-3-azabicyclo[3.2.0]heptane-2,4-dione (3f)

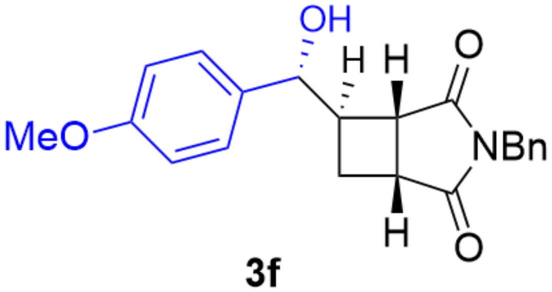

3f

yellow oil; **IR (neat):** 3452.24 (br), 1767.16 (w), 1694.01 (s), 1610.47 (w), 1512.43 (m), 1429.67 (w), 1391.27 (m), 1341.91 (m), 1306.90 (w), 1247.00 (m), 1170.29 (m), 1067.35 (w), 1030.72 (w), 935.74 (w), 833.81 (w), 749.08 (w), 701.94 (w), 651.69 (w), 620.69 (w) cm^{-1};
^{1}H NMR (400 MHz, $CDCl_3$) δ 7.29-7.13 (m, 7H), 6.79-6.76 (m, 2H), 4.59-4.54 (m, 3H), 3.70 (s, 3H), 3.32-3.29 (m, 1H), 3.10-3.05 (m,1 H), 2.80 (br, 1 H), 2.60-2.53 (m, 1H), 2.32-2.24 (m, 1H), 1.99-1.93 (m,1 H). **^{13}CNMR (100MHz, $CDCl_3$)** δ179.5, 179.0, 136.0, 133.3, 128.8, 128.7, 128.0, 127.7, 127.3, 114.1, 75.8, 55.3, 43.6, 42.6, 41.6, 35.7, 25.6.
HRMS (ESI$^+$) [M+Na]$^+$ Calcd for $C_{21}H_{21}NO_4Na$: 374.1363 m/z, Found: 374.1358 m/z;
Specific rotation: $[\alpha]_D^{29.6}$ 1.7 (*c* 0.50, $CHCl_3$) for an enantiomerically enriched sample of >99.5:0.5 e.r.
Enantiomeric purity of **3f** was determined by HPLC analysis in comparison with authentic racemic material (>99.5:0.5 e.r. shown; Chiralpak IB N-5 column, 90:10 hexane / ^{i}PrOH, 1.0 mL/min, 220nm).

<Sample Information>

Sample Name : CYL-09035-1
Sample ID :
Data Filename : CYL-09035-1NEW-IBN-5-90-10-1.0.lcd
Method Filename : ZJW-6-9010-1.0-80-1min.lcm
Batch Filename : ZJW.lcb
Vial # : 1-46 Sample Type : Unknown
Injection Volume : 10 uL
Date Acquired : 8/26/2024 6:15:21 PM Acquired by : System Administrator
Date Processed : 8/26/2024 7:22:57 PM Processed by : System Administrator

<Chromatogram>

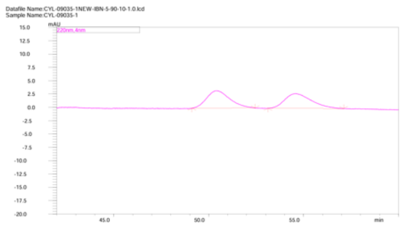

<Peak Table>

PDA Ch2 220nm

Peak#	Ret. Time	Area	Area%
1	50.430	274450	50.491
2	54.570	269108	49.509
Total		543558	100.000

<Sample Information>

Sample Name : CYL-09036
Sample ID :
Data Filename : CYL-09036NEW2-IBN-5-90-10-1.0.lcd
Method Filename : ZJW-6-9010-1.0-80-1min.lcm
Batch Filename : ZJW.lcb
Vial # : 1-47 Sample Type : Unknown
Injection Volume : 10 uL
Date Acquired : 8/26/2024 7:24:23 PM Acquired by : System Administrator
Date Processed : 8/26/2024 8:27:46 PM Processed by : System Administrator

<Chromatogram>

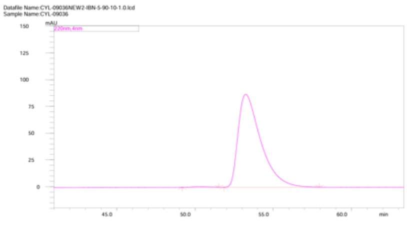

<Peak Table>

PDA Ch2 220nm

Peak#	Ret. Time	Area	Area%
1	50.360	36813	0.421
2	53.245	8713727	99.579
Total		8750540	100.000

(1*R*,5*S*,6*R*)-3-benzyl-6-((*R*)-hydroxy(4-morpholinophenyl)methyl)-3-azabicyclo[3.2.0]heptane-2,4-dione (3g)

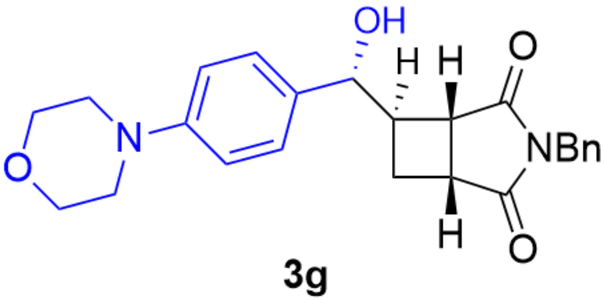

3g

yellow oil; **IR (neat):** 3497.05 (br), 1759.67 (w), 1685.39 (s), 1611.53 (w), 1516.73 (w), 1495.10 (w), 1450.53 (w), 1427.76 (m), 1400.65 (w), 1363.63 (w), 1335.14 (w), 1302.36 (w), 1264.04 (w), 1237.59 (w), 1214.94 (w), 1192.27 (w), 1174.61 (w), 1118.35 (w), 1069.93 (m), 1050.51 (w), 1021.25 (w), 989.12 (w), 925.66 (w), 844.43 (w), 811.41 (w), 779.40 (w), 726.04 (w), 696.43 (m), 653.40 (m), 629.04 (w), 561.45 (w), 484.65 (w), 455.29 (w), 438.22 (w) cm^{-1};
^{1}H NMR (400 MHz, $CDCl_3$) δ 7.38-7.20 (m, 7 H), 6.88-6.86 (m, 2 H), 4.67 (s, 2 H), 4.63 (d, J = 7.2 Hz, 1 H), 3.85-3.82 (m, 4 H), 3.39 (dd, J = 6.8, 4.0 Hz, 1 H), 3.19-3.12 (m, 5 H), 2.69-2.63 (m, 1 H), 2.54 (br, 1 H), 2.41-2.34 (m, 1 H), 2.09-2.02 (m, 1 H).
^{13}C NMR (125 MHz, $CDCl_3$) δ 179.5, 178.9, 151.3, 136.0, 132.5, 128.78, 128.76, 128.0, 127.5, 115.6, 75.9, 66.9, 49.1, 43.5, 42.6, 41.6, 35.7, 25.7.
HRMS (ESI^{+}) [M+Na]$^{+}$ Calcd for $C_{24}H_{26}N_2O_4Na$: 429.1785 m/z, Found: 429.1784 m/z;
Specific rotation: $[\alpha]_D^{25.7}$0.9 (c 0.50, $CHCl_3$) for an enantiomerically enriched sample of 99:1 e.r.
Enantiomeric purity of **3g** was determined by HPLC analysis in comparison with authentic racemic material (99:1 e.r. shown; Chiralpak IG column, 90:10 hexane / ^{i}PrOH, 1.0 mL/min, 254 nm).

<Sample Information>

Sample Name : CYL-09081-4
Sample ID :
Data Filename : CYL-09081-4-RACX-IG-80-20-1.0.lcd
Method Filename : ZJW-1-8020-1.0-120-1min.lcm
Batch Filename : ZJW.lcb
Vial # : 1-94 Sample Type : Unknown
Injection Volume : 20 uL
Date Acquired : 4/29/2024 10:52:59 AM Acquired by : System Administrator
Date Processed : 4/29/2024 2:10:19 PM Processed by : System Administrator

<Chromatogram>

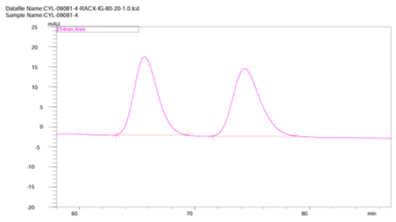

<Peak Table>

PDA Ch1 254nm

Peak#	Ret. Time	Area	Area%
1	65.634	2792905	49.977
2	74.363	2795488	50.023
Total		5588394	100.000

<Sample Information>

Sample Name : CYL-09060
Sample ID :
Data Filename : CYL-09060x-IG-80-20-1.0.lcd
Method Filename : ZJW-1-8020-1.0-120-1min.lcm
Batch Filename : ZJW.lcb
Vial # : 1-78 Sample Type : Unknown
Injection Volume : 20 uL
Date Acquired : 4/29/2024 12:20:55 PM Acquired by : System Administrator
Date Processed : 4/29/2024 2:20:59 PM Processed by : System Administrator

<Chromatogram>

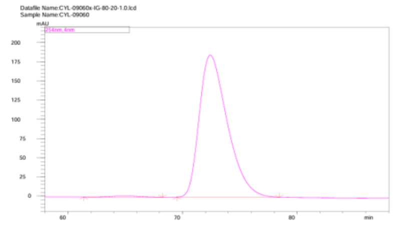

<Peak Table>

PDA Ch1 254nm

Peak#	Ret. Time	Area	Area%
1	64.972	238549	0.752
2	72.429	31469446	99.248
Total		31707995	100.000

(1*R*,5*S*,6*R*)-6-((*R*)-[1,1'-biphenyl]-4-yl(hydroxy)methyl)-3-benzyl-3-azabicyclo[3.2.0]heptane-2,4-dione (3h)

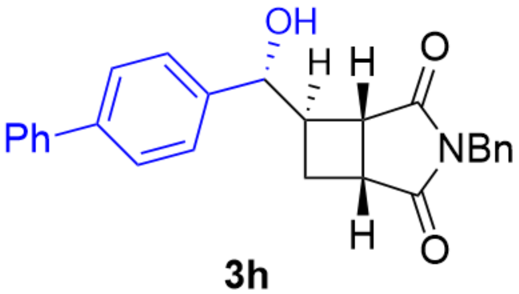

3h

white solid, m.p.183-184 °C; **IR (neat):** 3496.48 (br), 1759.21 (w), 1686.54 (s), 1486.99 (w), 1401.08 (m), 1362.26 (w), 1336.07 (w), 1175.95 (m), 1092.55 (w), 989.59 (m), 943.90 (w), 927.51 (w), 851.20 (w), 761.99 (m), 723.28 (m), 690.62 (m), 649.42 (w), 610.15 (w), 508.77 (w), 457.38 (w) cm^{-1};
^{1}H NMR (400 MHz, $CDCl_3$) δ 7.58-7.55 (m, 4 H), 7.45-7.24 (m, 10 H), 4.75 (d, *J* = 7.2 Hz, 1 H), 4.67 (s, 2 H), 3.44 (dd, *J* = 7.2, 3.6 Hz, 1 H), 3.23-3.17 (m, 1 H), 2.75-2.68 (m, 1 H), 2.50-2.42 (m, 1 H), 2.14-2.07 (m, 1 H). **^{13}C NMR (100 MHz, $CDCl_3$)** δ 179.5, 178.8, 141.3, 140.6, 140.3, 136.0, 128.9, 128.8, 128.1, 127.6, 127.2, 126.9, 76.0, 43.6, 42.7, 41.5, 35.8, 25.7.
HRMS (ESI$^+$) [M+Na]$^+$ Calcd for $C_{26}H_{23}NO_3Na$: 420.1570 m/z, Found: 420.1579 m/z;
Specific rotation: $[\alpha]_D^{27.8}$ 1.9 (*c* 0.50, $CHCl_3$) for an enantiomerically enriched sample of 99:1 e.r.
Enantiomeric purity of **3h** was determined by HPLC analysis in comparison with authentic racemic material (99:1 e.r. shown; Chiralpak IB N-5 column, 80:20 hexane / ^{i}PrOH, 1.0 mL/min, 220 nm).

<Sample Information>

Sample Name : CYL-09081-10rac
Sample ID :
Data Filename : CYL-09081-10rac-IBN-5-80-20-1.0.lcd
Method Filename : ZJW-4-8020-1.0-60-1min.lcm
Batch Filename : ZJW.lcb
Vial # : 1-100
Sample Type : Unknown
Injection Volume : 20 uL
Date Acquired : 4/28/2024 3:19:19 PM
Acquired by : System Administrator
Date Processed : 8/18/2024 5:01:59 PM
Processed by : System Administrator

<Chromatogram>

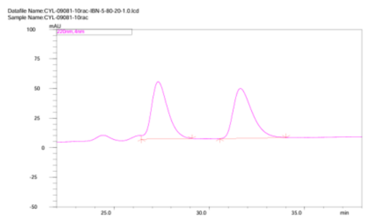

<Peak Table>

PDA Ch2 220nm

Peak#	Ret. Time	Area	Area%
1	27.316	2857333	49.516
2	31.647	2913146	50.484
Total		5770479	100.000

<Sample Information>

Sample Name : CYL-09068
Sample ID :
Data Filename : CYL-09068-IBN-5-80-20-1.0.lcd
Method Filename : ZJW-4-8020-1.0-60-1min.lcm
Batch Filename : ZJW.lcb
Vial # : 1-84
Sample Type : Unknown
Injection Volume : 20 uL
Date Acquired : 4/28/2024 4:17:14 PM
Acquired by : System Administrator
Date Processed : 4/28/2024 5:02:13 PM
Processed by : System Administrator

<Chromatogram>

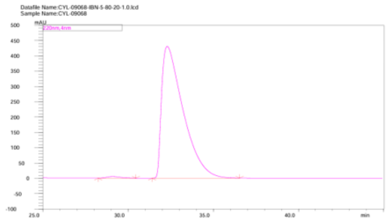

<Peak Table>

PDA Ch2 220nm

Peak#	Ret. Time	Area	Area%
1	29.098	289520	0.760
2	32.298	37780429	99.240
Total		38069949	100.000

(1*R*,5*S*,6*R*)-3-benzyl-6-((*R*)-hydroxy(3-(trifluoromethyl)phenyl)methyl)-3-azabicyclo[3.2.0]heptane-2,4-dione (3i)

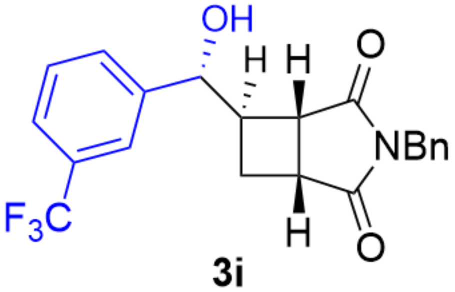

3i

yellow oil; **IR (neat):** 3507.70 (br), 2925.20 (w), 1762.26 (w), 1684.45 (s), 1496.28 (w), 1450.91 (w), 1430.55 (w), 1398.97 (m), 1365.99 (m), 1329.45 (s), 1262.58 (m), 1162.87 (s), 1114.29 (s), 1092.32 (s), 1072.73 (s), 1025.08 (s), 995.28 (w), 943.97 (m), 926.43 (w), 900.33 (s), 803.48 (s), 758.37 (s), 730.43 (s), 700.94 (s), 672.44 (s), 650.50 (s), 637.24 (m), 608.39 (w), 597.18 (w), 578.52 (w), 457.51 (w) cm^{-1};

^{1}H NMR (400 MHz, $CDCl_3$) δ7.53-7.38 (m, 4 H), 7.29-7.17 (m, 5 H), 4.71 (d, *J* = 7.2 Hz, 1 H), 4.57 (s, 2 H), 3.32 (dd, *J* = 6.8, 4.0 Hz, 1 H), 3.15-3.10 (m,1 H), 2.99 (br, 1 H), 2.62-2.55 (m, 1 H), 2.41-2.33 (m, 1H), 2.05-1.99 (m, 1 H). **^{13}C NMR (125 MHz, $CDCl_3$)** δ 179.3, 178.6, 142.4, 135.9, 131.1 (t, *J* = 32.5 Hz), 129.7, 129.4, 128.8, 128.8, 128.1, 125.2 (t, *J* = 3.8 Hz), 124.1 (t, *J* = 270.0 Hz), 123.2 (t, *J* = 3.8 Hz), 75.5, 43.7, 42.7, 41.2, 35.6, 25.5.**^{19}F NMR (376 MHz, $CDCl_3$)** δ -62.61.

HRMS (ESI$^+$) [M+Na]$^+$ Calcd for $C_{21}H_{18}NO_3F_3Na$: 412.1131 m/z, Found: 412.1134 m/z;

Specific rotation: $[\alpha]_D^{29.7}$ -0.2 (*c* 0.50, $CHCl_3$) for an enantiomerically enriched sample of >99.5:0.5 e.r.

Enantiomeric purity of **3i** was determined by HPLC analysis in comparison with authentic racemic material (>99.5:0.5 e.r. shown; Chiralpak IG column, 90:10 hexane / ^{i}PrOH, 1.0 mL/min, 254 nm).

<Sample Information>

Sample Name : cyl-09049-2
Sample ID :
Data Filename : cyl-09049-2NEWNEW-IG-90-10-1.0.lcd
Method Filename : cyl-1hao-90-10-1.0ml-40min.lcm
Batch Filename : WWLL.lcb
Vial # : 1-62 Sample Type : Unknown
Injection Volume : 10 uL
Date Acquired : 9/11/2024 2:59:11 Acquired by : System Administrator
Date Processed : 9/11/2024 8:28:50 Processed by : System Administrator

<Chromatogram>

Datafile Name:cyl-09049-2NEWNEW-IG-90-10-1.0.lcd
Sample Name:cyl-09049-2

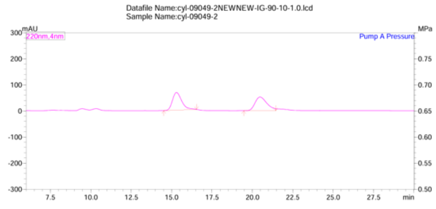

<Peak Table>

PDA Ch2 220nm

Peak#	Ret. Time	Area	Area%
1	15.307	2704538	49.949
2	20.475	2710006	50.051
Total		5414544	100.000

<Sample Information>

Sample Name : cyl-09037
Sample ID :
Data Filename : cyl-09037NEW-IG-90-10-1.0.lcd
Method Filename : cyl-1hao-90-10-1.0ml-40min.lcm
Batch Filename : WWLL.lcb
Vial # : 1-61 Sample Type : Unknown
Injection Volume : 10 uL
Date Acquired : 9/11/2024 2:18:39 Acquired by : System Administrator
Date Processed : 9/11/2024 8:22:29 Processed by : System Administrator

<Chromatogram>

Datafile Name:cyl-09037NEW-IG-90-10-1.0.lcd
Sample Name:cyl-09037

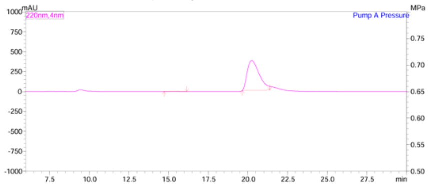

<Peak Table>

PDA Ch2 220nm

Peak#	Ret. Time	Area	Area%
1	15.359	74658	0.375
2	20.237	19836008	99.625
Total		19910665	100.000

Methyl 3-((*R*)-((1*R*,5*S*,6*R*)-3-benzyl-2,4-dioxo-3-azabicyclo[3.2.0]heptan-6-yl)(hydroxy)methyl)benzoate (3j)

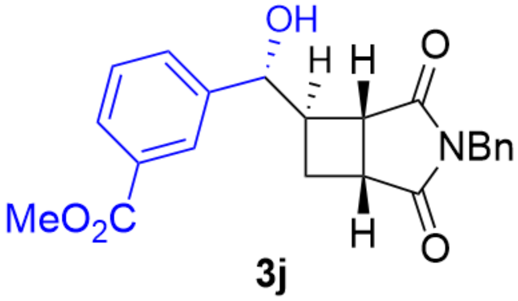

3j

pale yellow oil; **IR (neat):** 3459.96 (br), 2988.92 (w), 2951.82 (w), 1768.52 (w), 1690.05 (s), 1604.46 (w), 1587.78 (w), 1493.57 (w), 1432.17 (m), 1390.06 (m), 1340.10 (m), 1285.03 (s), 1198.54 (m), 1166.32 (m), 1106.40 (w), 1078.57 (w), 998.86 (w), 979.48 (m), 939.43 (m), 909.79 (m), 819.75 (s), 801.93 (s), 728.42 (m), 698.10 (w), 648.35 (w), 618.36 (w), 588.90 (w), 474.72 (w) cm^{-1};
^{1}H NMR (400 MHz, CDCl$_3$) δ 7.97 (s, 1 H), 7.92 (d, *J* = 8.0 Hz, 1 H), 7.52-7.18 (m, 7 H), 4.75 (d, *J* = 7.2 Hz, 1 H), 4.61 (s, 2 H), 3.86 (s, 3 H), 3.54 (br, 1 H), 3.38 (dd, *J* = 6.8, 4.4 Hz, 1 H), 3.18-3.13 (m, 1 H), 2.67-2.62 (m, 1 H), 2.45-2.38 (m, 1 H), 2.07-2.00 (m, 1 H). **^{13}C NMR (100 MHz, CDCl$_3$)** δ 179.4, 178.7, 167.0, 141.9, 135.9, 130.8, 130.6, 129.5, 129.0, 128.8, 128.8, 128.0, 127.5, 75.5, 52.3, 43.7, 42.6, 41.2, 35.7, 25.5.
HRMS (ESI$^+$) [M+Na]$^+$ Calcd for $C_{22}H_{21}NO_5Na$: 402.1312 m/z, Found: 402.1318 m/z;
Specific rotation: $[\alpha]_D^{29.5}$ 4.9 (*c* 0.50, $CHCl_3$) for an enantiomerically enriched sample of >99.5:0.5 e.r.

Enantiomeric purity of **3j** was determined by HPLC analysis in comparison with authentic racemic material (>99.5:0.5 e.r. shown; Chiralpak IB N-5 column, 90:10 hexane / ^{i}PrOH, 1.0 mL/min, 220 nm).

<Sample Information>

Sample Name : cyl-09081-3
Sample ID :
Data Filename : cyl-09081-3NEW2-IBN-5-90-10-1.0.lcd
Method Filename : cyl-6hao-90-10-1.0ml-100min.lcm
Batch Filename : WWLL.lcb
Vial # : 1-70 Sample Type : Unknown
Injection Volume : 10 uL
Date Acquired : 9/11/2024 18:15:57 Acquired by : System Administrator
Date Processed : 9/11/2024 20:03:15 Processed by : System Administrator

<Chromatogram>

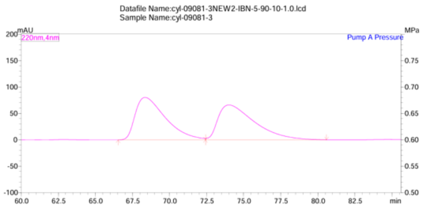

<Peak Table>

PDA Ch2 220nm

Peak#	Ret. Time	Area	Area%
1	68.349	11410238	50.252
2	74.011	11295754	49.748
Total		22705992	100.000

<Sample Information>

Sample Name : cyl-09058
Sample ID :
Data Filename : cyl-09058NEW3-IBN-5-90-10-1.0.lcd
Method Filename : cyl-6hao-90-10-1.0ml-100min.lcm
Batch Filename : WWLL.lcb
Vial # : 1-97 Sample Type : Unknown
Injection Volume : 10 uL
Date Acquired : 9/11/2024 21:00:46 Acquired by : System Administrator
Date Processed : 9/11/2024 22:40:49 Processed by : System Administrator

<Chromatogram>

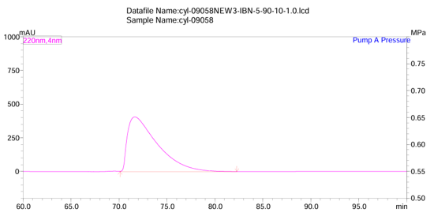

<Peak Table>

PDA Ch2 220nm

Peak#	Ret. Time	Area	Area%
1	71.652	87932215	100.000
Total		87932215	100.000

3-((*R*)-((1*R*,5*S*,6*R*)-3-benzyl-2,4-dioxo-3-azabicyclo[3.2.0]heptan-6-yl)(hydroxy)methyl)benzonitrile (3k)

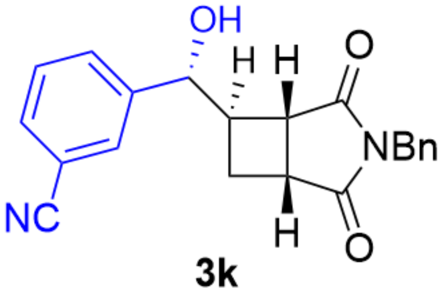

3k

yellow oil; **IR (neat):** 3447.19 (br), 2229.28 (w), 1767.23 (w), 1689.33 (s), 1584.43 (w), 1495.50 (w), 1430.31 (w), 1390.95 (m), 1338.02 (w), 1311.21 (w), 1292.39 (w), 1241.26 (w), 1161.98 (m), 1051.29 (w), 998.90 (w), 909.84 (w), 803.13 (w), 731.38 (m), 694.93 (m), 621.43 (w), 576.90 (w), 471.40 (w) cm^{-1};
^{1}H NMR (400 MHz, $CDCl_3$) δ 7.65 (s, 1 H), 7.60-7.57 (m, 2 H), 7.49-7.45 (m, 1 H), 7.35-7.24 (m, 5 H), 4.79 (d, *J* = 7.2 Hz, 1 H), 4.64 (s, 2 H), 3.38 (dd, *J* = 7.6, 4.8 Hz, 1 H), 3.24-3.18 (m, 1 H), 2.67-2.60 (m, 1 H), 2.53-2.45 (m, 1 H), 2.15-2.09 (m, 1 H), 1.89 (br, 1 H). **^{13}C NMR (100 MHz, $CDCl_3$)** δ 179.2, 178.6, 143.1, 135.9, 131.9, 130.7, 130.0, 129.7, 128.84, 128.75, 128.1, 118.7, 112.8, 74.6, 43.8, 42.7, 40.7, 35.6, 25.4.
HRMS (ESI$^+$) [M+Na]$^+$ Calcd for $C_{21}H_{18}N_2O_3Na$: 369.1210 m/z, Found: 369.1217 m/z;
Specific rotation: $[\alpha]_D^{28.2}$ -8.9 (*c* 0.50, $CHCl_3$) for an enantiomerically enriched sample of 99.5:0.5 e.r.
Enantiomeric purity of **3k** was determined by HPLC analysis in comparison with authentic racemic material (99.5:0.5 e.r. shown; Chiralpak IB N-5 column, 80:20 hexane / ^{i}PrOH, 1.0 mL/min, 254 nm).

<Sample Information>

Sample Name : CYL-09081-15
Sample ID :
Data Filename : CYL-09081-15RACX-IBN-5-80-20-1.0.lcd
Method Filename : ZJW-4-8020-1.0-60-1min.lcm
Batch Filename : ZJW.lcb
Vial # : 1-99
Sample Type : Unknown
Injection Volume : 20 uL
Date Acquired : 5/8/2024 9:30:57 AM
Acquired by : System Administrator
Date Processed : 8/26/2024 9:30:00 PM
Processed by : System Administrator

<Chromatogram>

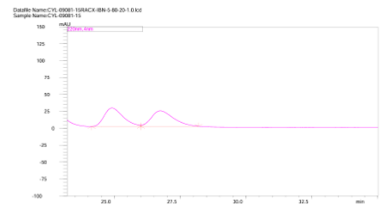

<Peak Table>

PDA Ch2 220nm

Peak#	Ret. Time	Area	Area%
1	24.907	1351387	50.384
2	26.734	1330806	49.616
Total		2682193	100.000

<Sample Information>

Sample Name : CYL-09096
Sample ID :
Data Filename : CYL-09096X-IBN-5-80-20-1.0.lcd
Method Filename : ZJW-4-8020-1.0-60-1min.lcm
Batch Filename : ZJW.lcb
Vial # : 1-100
Sample Type : Unknown
Injection Volume : 20 uL
Date Acquired : 5/8/2024 8:58:50 AM
Acquired by : System Administrator
Date Processed : 8/18/2024 7:57:50 PM
Processed by : System Administrator

<Chromatogram>

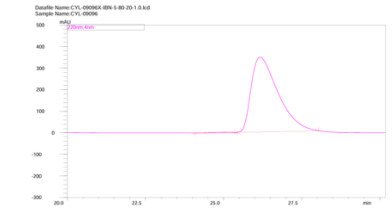

<Peak Table>

PDA Ch2 220nm

Peak#	Ret. Time	Area	Area%
1	25.013	110677	0.532
2	26.171	20710509	99.468
Total		20821186	100.000

(1*R*,5*S*,6*R*)-3-benzyl-6-((*R*)-(3-fluorophenyl)(hydroxy)methyl)-3-azabicyclo[3.2.0]heptane-2,4-dione (3l)

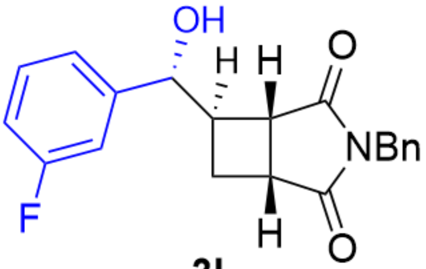

3l

yellow oil; **IR (neat):** 3498.23 (br), 1759.79 (w), 1681.64 (s), 1612.90 (w), 1588.50 (w), 1487.40 (w), 1450.12 (m), 1399.23 (m), 1363.42 (w), 1335.70 (m), 1299.18 (w), 1270.23 (w), 1247.92 (w), 1194.97 (m), 1166.19 (w), 1096.23 (w), 1067.89 (w), 1020.12 (m), 992.86 (w), 936.25 (w), 908.70 (w), 867.76 (w), 791.95 (m), 754.47 (m), 725.86 (w), 699.78 (w), 688.77 (w), 653.97 (w), 634.16 (w), 577.63 (w), 491.19 (w), 452.20 (w), 429.82 (w) cm^{-1};
^{1}H NMR (400 MHz, $CDCl_3$) δ 7.38-7.24 (m, 5 H), 7.09-7.03 (m, 2 H), 7.00-6.95 (m, 1 H), 4.70 (d, *J* = 6.8 Hz, 1 H), 4.64 (s, 2 H), 3.38 (dd, *J* = 4.8, 4.0 Hz, 1 H), 3.20-3.15 (m, 1 H), 2.66-2.59 (m, 1 H), 2.47-2.39 (m, 1 H), 2.11-2.04 (m, 1 H). **^{13}C NMR (125 MHz, $CDCl_3$)** δ 179.3, 178.7, 162.9 (d, *J* =245.0 Hz), 143.9 (d, *J* =6.3 Hz), 135.8, 130.3 (d, *J* =8.8 Hz), 128.7, 128.7, 128.0, 121.8 (d, *J* =2.5 Hz), 115.1 (d, *J* =21.3 Hz), 113.2 (d, *J* =21.3 Hz), 75.3, 43.6, 42.6, 41.1, 35.6, 25.4. **^{19}F NMR (376 MHz, $CDCl_3$)** δ -112.16.
HRMS (ESI$^+$) [M+Na]$^+$ Calcd for $C_{20}H_{18}NO_3FNa$: 362.1163 m/z, Found: 362.1171 m/z;
Specific rotation: $[\alpha]_D^{28.8}$-10.9 (*c* 0.50, $CHCl_3$) for an enantiomerically enriched sample of 99.5:0.5 e.r.
Enantiomeric purity of **3l** was determined by HPLC analysis in comparison with authentic racemic material (99.5:0.5 e.r. shown; Chiralpak IG column, 90:10 hexane / ^{i}PrOH, 1.0 mL/min, 220 nm).

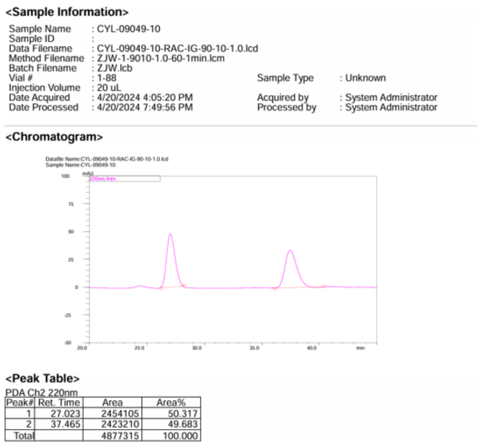

<Sample Information>

Sample Name : CYL-09049-10
Sample ID :
Data Filename : CYL-09049-10-RAC-IG-90-10-1.0.lcd
Method Filename : ZJW-1-9010-1.0-60-1min.lcm
Batch Filename : ZJW.lcb
Vial # : 1-88
Sample Type : Unknown
Injection Volume : 20 uL
Date Acquired : 4/20/2024 4:05:20 PM
Acquired by : System Administrator
Date Processed : 4/20/2024 7:49:56 PM
Processed by : System Administrator

<Chromatogram>

<Peak Table>

PDA Ch2 220nm

Peak#	Ret. Time	Area	Area%
1	27.023	2454105	50.317
2	37.465	2423210	49.683
Total		4877315	100.000

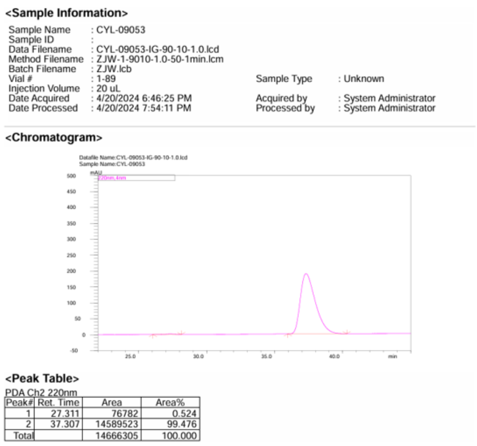

<Sample Information>

Sample Name : CYL-09053
Sample ID :
Data Filename : CYL-09053-IG-90-10-1.0.lcd
Method Filename : ZJW-1-9010-1.0-50-1min.lcm
Batch Filename : ZJW.lcb
Vial # : 1-89
Sample Type : Unknown
Injection Volume : 20 uL
Date Acquired : 4/20/2024 6:46:25 PM
Acquired by : System Administrator
Date Processed : 4/20/2024 7:54:11 PM
Processed by : System Administrator

<Chromatogram>

<Peak Table>

PDA Ch2 220nm

Peak#	Ret. Time	Area	Area%
1	27.311	76782	0.524
2	37.307	14589523	99.476
Total		14666305	100.000

(1*R*,5*S*,6*R*)-3-benzyl-6-((*R*)-(3-chlorophenyl)(hydroxy)methyl)-3-azabicyclo[3.2.0]heptane-2,4-dione (3m)

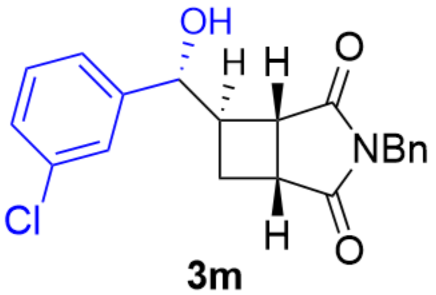

3m

yellow oil; **IR (neat):** 3514.75 (br), 1760.48 (w), 1682.78 (s), 1597.47 (w), 1574.11 (w), 1431.65 (m), 1398.35 (w), 1363.41 (m), 1334.24 (w), 1299.10 (w), 1194.55 (w), 1175.16 (w), 1163.51 (w), 1093.65 (w), 1071.16 (w), 1026.60 (w), 995.75 (w), 944.36 (w), 888.40 (w), 789.16 (w), 725.94 (m), 698.35 (m), 651.60 (w), 637.29 (w), 622.77 (w), 575.54 (w), 454.48 (w), 424.16 (w) cm^{-1};
^{1}H NMR (400 MHz, $CDCl_3$) δ7.35-7.23 (m, 8 H), 7.18-7.16 (m, 1 H), 4.67- 4.63 (m, 3 H), 3.37-3.30 (m, 2 H), 3.19-3.13 (m, 1 H), 2.65-2.58 (m, 1 H), 2.44-2.36 (m, 1 H), 2.09-2.02 (m, 1 H). **^{13}C NMR (100 MHz, $CDCl_3$)** δ 179.4, 178.8, 143.5, 135.9, 134.6, 130.1, 128.8, 128.7, 128.4, 128.0, 126.6, 124.5, 75.4, 43.6, 42.6, 41.2, 35.6, 25.4. **HRMS (ESI^+) $[M+Na]^+$** Calcd for $C_{20}H_{18}NO_3NaCl$: 378.0867 m/z, Found: 378.0870 m/z;
Specific rotation: $[\alpha]_D^{27.0}$ -5.8 (*c* 0.50, $CHCl_3$) for an enantiomerically enriched sample of 99:1 e.r.
Enantiomeric purity of **3m** was determined by HPLC analysis in comparison with authentic racemic material (99:1 e.r. shown; Chiralpak IE column, 90:10 hexane / ^{i}PrOH, 1.0 mL/min, 220 nm).

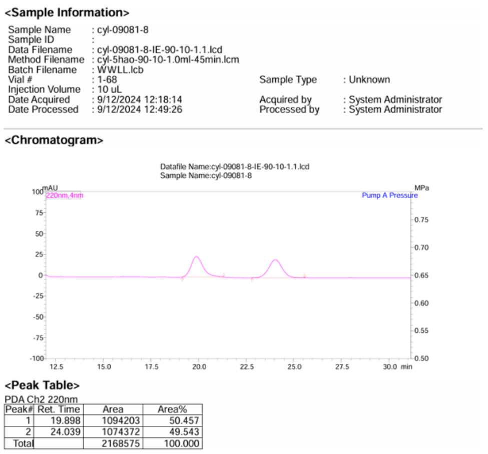

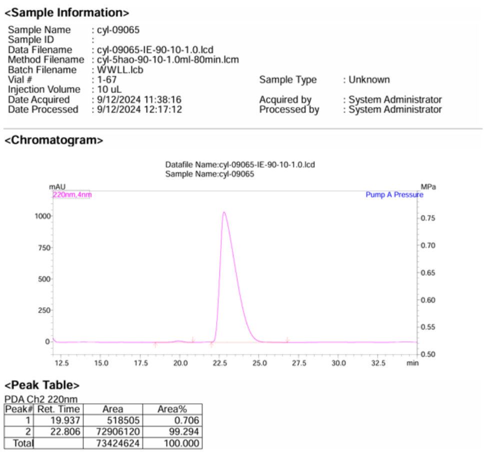

(1*R*,5*S*,6*R*)-3-benzyl-6-((*R*)-hydroxy(naphthalen-2-yl)methyl)-3-azabicyclo[3.2.0]heptane-2,4-dione (3n)

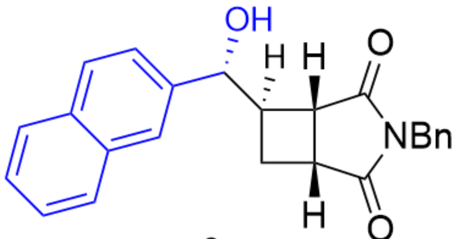

3n

white solid, m.p.173-175 °C;**IR (neat):** 3493.77 (br), 1760.50 (w), 1685.86 (s), 1402.43 (m), 1363.96 (w), 1335.52 (m), 1302.02 (m), 1195.53 (w), 1173.54 (m), 1130.05 (w), 1094.07 (w), 1016.73 (m), 991.12 (w), 943.65 (w), 926.13 (w), 895.59 (m), 857.33 (m), 820.93 (w), 805.89 (w), 776.73 (m), 753.72 (m), 724.76 (m), 699.51 (w), 645.35 (w), 524.36 (w), 500.19 (w), 477.80 (m), 449.00 (m) cm^{-1};
^{1}H NMR (400 MHz, $CDCl_3$) δ7.83-7.78 (m, 4 H), 7.50-7.46 (m, 2 H), 7.44-7.41 (m, 1 H), 7.37-7.34 (m, 2 H), 7.31-7.24 (m, 3 H), 4.86 (d, *J* = 7.2 Hz, 1 H), 4.66 (s, 2 H), 3.44 (dd, *J* = 7.2, 4.4 Hz, 1 H), 3.22-3.16 (m, 1 H), 2.82-2.75 (m, 1 H), 2.51-2.43 (m, 1 H), 2.11-2.04 (m, 1 H), 1.78 (br, 1 H). **^{13}C NMR (125 MHz, $CDCl_3$)** δ 179.5, 178.8,138.6, 136.0, 133.4, 133.3, 128.82, 128.80, 128.1, 128.1, 127.9, 126.5, 126.4, 125.5, 124.1, 76.4, 43.5, 42.6, 41.5, 35.8, 25.7.
HRMS (ESI$^+$) [M+Na]$^+$ Calcd for $C_{24}H_{21}NO_3Na$: 394.1414 m/z, Found: 394.1421 m/z;
Specific rotation: $[\alpha]_D^{29.5}$14.3 (*c* 0.50, $CHCl_3$) for an enantiomerically enriched sample of 99:1 e.r.
Enantiomeric purity of **3n** was determined by HPLC analysis in comparison with authentic racemic material (99:1 e.r. shown; Chiralpak IE column, 90:10 hexane / ^{i}PrOH, 1.0 mL/min, 254 nm).

<Sample Information>

Sample Name : cyl-09081-5
Sample ID :
Data Filename : cyl-09081-5-IE-90-10-1.0.lcd
Method Filename : cyl-5hao-90-10-1.0ml-80min.lcm
Batch Filename : WWLL.lcb
Vial # : 1-72
Sample Type : Unknown
Injection Volume : 10 uL
Date Acquired : 9/12/2024 15:19:11
Acquired by : System Administrator
Date Processed : 9/12/2024 16:07:50
Processed by : System Administrator

<Chromatogram>

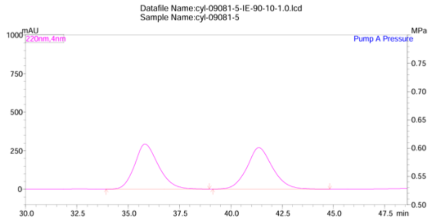

<Peak Table>

PDA Ch2 220nm

Peak#	Ret. Time	Area	Area%
1	35.815	23566661	50.287
2	41.361	23298041	49.713
Total		46864702	100.000

<Sample Information>

Sample Name : cyl-09061
Sample ID :
Data Filename : cyl-09061-IE-90-10-1.0.lcd
Method Filename : cyl-5hao-90-10-1.0ml-80min.lcm
Batch Filename : WWLL.lcb
Vial # : 1-71
Sample Type : Unknown
Injection Volume : 10 uL
Date Acquired : 9/12/2024 14:16:33
Acquired by : System Administrator
Date Processed : 9/12/2024 16:07:20
Processed by : System Administrator

<Chromatogram>

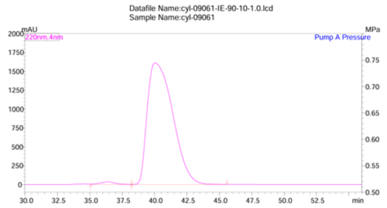

<Peak Table>

PDA Ch2 220nm

Peak#	Ret. Time	Area	Area%
1	36.380	2951706	1.320
2	40.044	220581728	98.680
Total		223533434	100.000

(1*R*,5*S*,6*R*)-3-benzyl-6-((*R*)-(2,3-dihydrobenzo[b][1,4]dioxin-6-yl)(hydroxy)methyl)-3-azabicyclo[3.2.0]heptane-2,4-dione (3o)

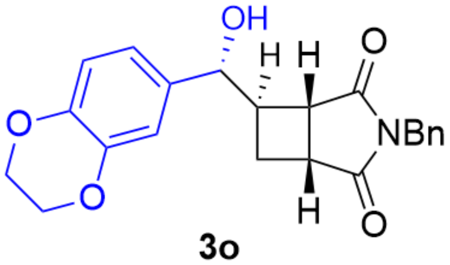

3o

milky oil; **IR (neat):** 3437.43 (br), 3043.64 (w), 1697.58 (s), 1588.93 (w), 1505.55 (w), 1431.02 (w), 1390.75 (w), 1343.00 (m), 1286.06 (w), 1258.98 (w), 1164.93 (w), 1123.54 (w), 1067.28 (w), 918.77 (w), 886.94 (w), 700.88 (w) cm^{-1};

^{1}H NMR (400 MHz, $CDCl_3$) δ7.38-7.24 (m, 5 H), 6.85-6.78 (m, 3 H), 4.66 (s, 2 H), 4.60 (d, J = 7.6 Hz, 1 H), 4.23 (s, 4 H), 3.39-3.37 (m, 1 H), 3.19-3.14 (m, 1 H), 2.66-2.59 (m, 1 H), 2.44-2.35 (m, 2 H), 2.10-2.03 (m, 1 H). **^{13}C NMR (100 MHz, $CDCl_3$)** δ 179.5, 178.7, 143.8, 136.1, 134.7, 128.8, 128.1, 119.4, 117.6, 115.4, 75.9, 64.5, 43.6, 42.6, 41.5, 35.8, 29.8, 25.7.

HRMS (ESI$^+$) [M+Na]$^+$ Calcd for $C_{22}H_{21}NO_5Na$: 271.0117 m/z, Found: 271.0116 m/z;

Specific rotation: $[\alpha]_D^{27.9}$ -1.4 (c 0.50, $CHCl_3$) for an enantiomerically enriched sample of 99:1 e.r.

Enantiomeric purity of **3o** was determined by HPLC analysis in comparison with authentic racemic material (99:1 e.r. shown; Chiralpak IB N-5 column, 80:20 hexane / ^{i}PrOH, 1.0 mL/min, 220 nm).

<Sample Information>

Sample Name : cyl-09049-9
Sample ID :
Data Filename : cyl-09049-9NEW-IBN-5-80-20-1.0.lcd
Method Filename : cyl-6hao-80-20-1.0ml-90min.lcm
Batch Filename : WWLL.lcb
Vial # : 1-85 Sample Type : Unknown
Injection Volume : 10 uL
Date Acquired : 9/12/2024 23:32:05 Acquired by : System Administrator
Date Processed : 9/13/2024 1:02:08 Processed by : System Administrator

<Chromatogram>

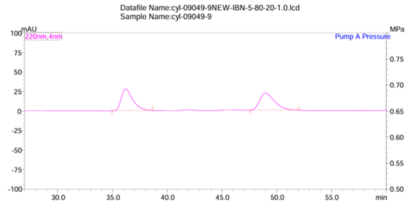

<Peak Table>

PDA Ch2 220nm

Peak#	Ret. Time	Area	Area%
1	36.213	2042558	49.642
2	48.991	2072034	50.358
Total		4114593	100.000

<Sample Information>

Sample Name : cyl-09052
Sample ID :
Data Filename : cyl-09052NEWNEW3-IBN-5-80-20-1.0.lcd
Method Filename : cyl-6hao-80-20-1.0ml-90min.lcm
Batch Filename : WWLL.lcb
Vial # : 1-84 Sample Type : Unknown
Injection Volume : 10 uL
Date Acquired : 9/12/2024 22:01:23 Acquired by : System Administrator
Date Processed : 9/13/2024 8:04:27 Processed by : System Administrator

<Chromatogram>

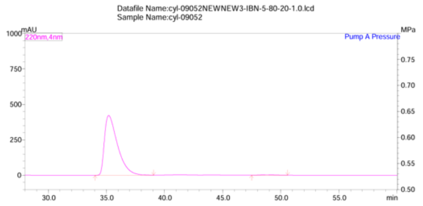

<Peak Table>

PDA Ch2 220nm

Peak#	Ret. Time	Area	Area%
1	35.196	33028643	99.430
2	48.784	189315	0.570
Total		33217958	100.000

(1*R*,5*S*,6*R*)-6-((*R*)-benzo[d][1,3]dioxol-5-yl(hydroxy)methyl)-3-benzyl-3-azabicyclo[3.2.0]heptane-2,4-dione (3p)

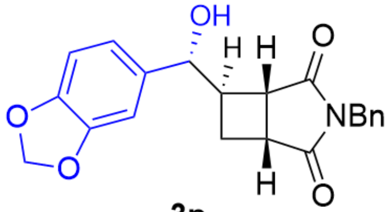

3p

yellow oil; **IR (neat):** 3437.78 (br), 2968.25 (w), 1766.52 (w), 1689.77 (s), 1607.65 (w), 1501.24 (m), 1487.18 (m), 1440.35 (m), 1390.76 (m), 1340.57 (m), 1310.13 (w), 1292.11 (m), 1239.13 (s), 1161.60 (m), 1126.40 (w), 1093.49 (w), 1067.37 (w), 1035.46 (s), 995.78 (w), 928.18 (s), 886.72 (w), 813.65 (w), 747.72 (m), 698.72 (w), 643.96 (w), 620.37 (w), 476.08 (w) cm^{-1};

^{1}H NMR (400 MHz, $CDCl_3$) δ7.36-7.23 (m, 5 H), 6.80 (s, 1 H), 6.74 (s, 2 H), 5.92 (s, 2 H), 4.64 (s, 2 H), 4.59 (d, *J* = 7.6 Hz, 1 H), 3.36 (dd, *J* = 6.8, 4.0 Hz, 1 H), 3.18-3.12 (m, 1 H), 2.63-2.56 (m, 1 H), 2.39-2.31 (m, 1 H), 2.07-2.00 (m, 1 H). **^{13}C NMR (100 MHz, $CDCl_3$)** δ 179.5, 178.8, 148.1, 147.5, 136.0, 135.3, 128.8, 128.7, 128.0, 119.9, 108.3, 106.7, 101.2, 76.1, 43.7, 42.6, 41.5, 35.7, 25.6.

HRMS (ESI$^+$) [M+Na]$^+$ Calcd for $C_{21}H_{19}NO_5Na$: 388.1154 m/z, Found: 388.1161 m/z;

Specific rotation: $[\alpha]_D^{27.3}$ -6.9 (*c* 0.50, $CHCl_3$) for an enantiomerically enriched sample of >99.5:0.5 e.r.

Enantiomeric purity of **3p** was determined by HPLC analysis in comparison with authentic racemic material (>99.5:0.5 e.r. shown; Chiralpak IB N-5 column, 90:10 hexane / ^{i}PrOH, 1.0 mL/min, 220 nm).

<Sample Information>

Sample Name : CYL-09049-6
Sample ID :
Data Filename : CYL-09049-6rac-IBN-5-90-10-1.0.lcd
Method Filename : ZJW-4-9010-1.0-80-1min.lcm
Batch Filename : ZJW.lcb
Vial # : 1-80 Sample Type : Unknown
Injection Volume : 20 uL
Date Acquired : 4/20/2024 1:27:34 AM Acquired by : System Administrator
Date Processed : 4/20/2024 8:26:00 AM Processed by : System Administrator

<Chromatogram>

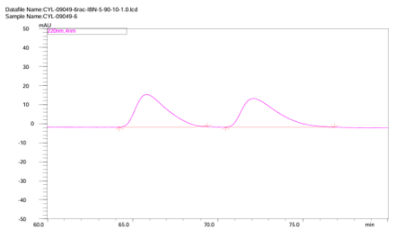

<Peak Table>

PDA Ch2 220nm

Peak#	Ret. Time	Area	Area%
1	65.833	2186807	49.999
2	72.106	2186894	50.001
Total		4373702	100.000

<Sample Information>

Sample Name : CYL-09045
Sample ID :
Data Filename : CYL-09045X-IBN-5-90-10-1.0.lcd
Method Filename : ZJW-4-9010-1.0-90-1min.lcm
Batch Filename : ZJW.lcb
Vial # : 1-81 Sample Type : Unknown
Injection Volume : 20 uL
Date Acquired : 4/21/2024 3:16:49 PM Acquired by : System Administrator
Date Processed : 4/22/2024 8:48:06 AM Processed by : System Administrator

<Chromatogram>

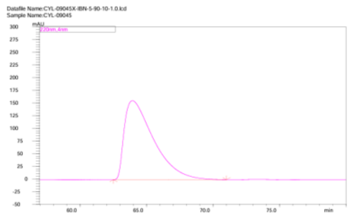

<Peak Table>

PDA Ch2 220nm

Peak#	Ret. Time	Area	Area%
1	63.947	23983598	100.000
Total		23983598	100.000

(1*R*,5*S*,6*R*)-3-benzyl-6-((*R*)-(2-fluorophenyl)(hydroxy)methyl)-3-azabicyclo[3.2.0]heptane-2,4-dione (3q)

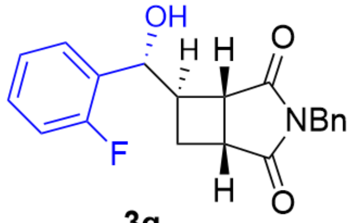

3q

yellow oil; **IR (neat):** 3498.25 (br), 1760.10 (w), 1683.70 (s), 1613.66 (w), 1488.46 (w), 1453.38 (w), 1400.70 (m). 1363.19 (w), 1335.57 (m), 1275.24 (w), 1226.77 (w), 1195.08 (w), 1174.14 (m), 1102.53 (w), 1079.46 (w), 993.05 (m), 946.15 (m), 885.75 (w), 798.94 (m), 749.99 (s), 725.68 (m), 700.49 (w), 649.88 (w), 610.61 (m), 487.78 (w), 456.48 (w), 442.31 (w) cm^{-1};
^{1}H NMR (400 MHz, $CDCl_3$) δ7.47-7.43 (m, 1 H), 7.35-7.22 (m, 6 H), 7.15-7.12 (m, 1 H), 7.03-6.97 (m, 1 H), 5.06 (d, *J* = 6.8 Hz, 1 H), 4.63 (d, *J* = 2.8 Hz, 2 H), 3.39 (dd, *J* = 6.4, 4.4 Hz, 1 H), 3.28 (br, 1 H), 3.20-3.15 (m, 1 H), 2.73-2.67 (m, 1 H), 2.52-2.44 (m, 1 H), 2.09-2.02 (m, 1 H). **^{13}C NMR (125 MHz, $CDCl_3$)** δ179.5, 178.9, 159.9 (d, *J* =243.8 Hz), 136.0, 129.7 (d, *J* =7.5 Hz), 128.78, 128.76, 128.5 (d, *J* =13.8 Hz), 128.0, 127.6 (d, *J* =5.0 Hz), 124.7 (d, *J* =3.8 Hz), 115.5 (d, *J* =21.3 Hz), 69.3 (d, *J* =2.5 Hz), 43.0, 42.6, 41.0, 35.8, 25.2. **^{19}F NMR (376 MHz, $CDCl_3$)** δ -118.86.
HRMS (ESI$^+$) [M+Na]$^+$ Calcd for $C_{20}H_{18}NO_3FNa$: 362.1163 m/z, Found: 362.1164 m/z;
Specific rotation: $[\alpha]_D^{24.3}$ -3.6 (*c* 0.50, $CHCl_3$) for an enantiomerically enriched sample of >99.5:0.5 e.r.
Enantiomeric purity of **3q** was determined by HPLC analysis in comparison with authentic racemic material (>99.5:0.5 e.r. shown; Chiralpak IB N-5 column, 90:10 hexane / ^{i}PrOH, 1.0 mL/min, 220 nm).

<Sample Information>

Sample Name : CYL-09049-7
Sample ID :
Data Filename : CYL-09049-7RAC-IBN-5-90-10-1.0.lcd
Method Filename : ZJW-4-9010-1.0-80-1min.lcm
Batch Filename : ZJW.lcb
Vial # : 1-82
Sample Type : Unknown
Injection Volume : 20 uL
Date Acquired : 4/20/2024 4:08:42 AM
Acquired by : System Administrator
Date Processed : 4/20/2024 8:27:31 AM
Processed by : System Administrator

<Chromatogram>

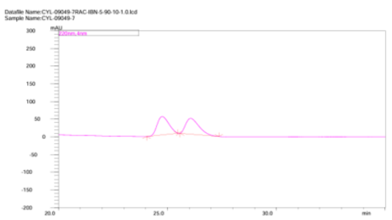

<Peak Table>

PDA Ch2 220nm

Peak#	Ret. Time	Area	Area%
1	24.761	1919681	50.446
2	26.068	1885723	49.554
Total		3805404	100.000

<Sample Information>

Sample Name : CYL-09047
Sample ID :
Data Filename : CYL-09047-IBN-5-90-10-1.0.lcd
Method Filename : ZJW-4-9010-1.0-80-1min.lcm
Batch Filename : ZJW.lcb
Vial # : 1-83
Sample Type : Unknown
Injection Volume : 20 uL
Date Acquired : 4/20/2024 5:29:16 AM
Acquired by : System Administrator
Date Processed : 4/20/2024 6:49:19 AM
Processed by : System Administrator

<Chromatogram>

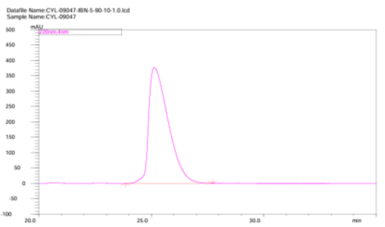

<Peak Table>

PDA Ch2 220nm

Peak#	Ret. Time	Area	Area%
1	25.129	22688448	100.000
Total		22688448	100.000

(1*R*,5*S*,6*R*)-3-benzyl-6-((*R*)-hydroxy(2-methoxyphenyl)methyl)-3-azabicyclo[3.2.0]heptane-2,4-dione (3r)

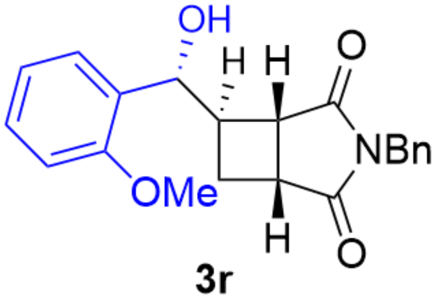

3r

yellow oil; **IR (neat):** 3505.27 (br), 1760.25 (w), 1689.31 (s), 1610.60 (w), 1493.55 (w), 1392.58 (w), 1358.09 (w), 1331.74 (m), 1288.77 (m), 1239.54 (m), 1163.44 (w), 1141.11 (w), 1089.69 (w), 1070.31 (w), 1048.87 (w), 1030.91 (w), 1017.22 (w), 991.30 (w), 962.70 (w), 930.40 (w), 855.33 (w), 751.64 (w), 738.03 (w), 719.39 (w), 700.35 (w), 674.98 (w), 654.50 (w), 625.24 (m), 614.67 (w), 574.00 (w), 534.25 (w), 509.64 (w), 456.73 (w), 424.56 (w) cm^{-1};
^{1}H NMR (400 MHz, $CDCl_3$) δ 7.38-7.23 (m, 7 H), 6.98-6.94 (m, 1 H), 6.87 (d, J = 8.0 Hz, 1 H), 4.93-4.90 (m, 1 H), 4.66 (s, 2 H), 3.81 (s, 3 H), 3.41 (dd, J = 6.8, 4.4 Hz, 1 H), 3.22-3.17 (m, 1 H), 3.11 (d, J = 4.0, 1 H), 2.83-2.76 (m, 1 H), 2.53-2.45 (m, 1 H), 2.11-2.03 (m, 1 H). **^{13}C NMR (100 MHz, $CDCl_3$)** δ 179.4, 179.0, 156.7, 136.1, 129.1, 129.1, 128.7, 128.0, 127.4, 121.0, 110.7, 72.0, 55.4, 42.5, 42.0, 41.2, 36.0, 25.7.
HRMS (ESI$^+$) [M+Na]$^+$ Calcd for $C_{21}H_{21}NO_4Na$: 374.1363 m/z, Found: 374.1368 m/z;
Specific rotation: $[\alpha]_D^{26.7}$ -3.5 (*c* 0.50, $CHCl_3$) for an enantiomerically enriched sample of 99:1 e.r.

Enantiomeric purity of **3r** was determined by HPLC analysis in comparison with authentic racemic material (99:1 e.r. shown; Chiralpak IB N-5 column, 90:10 hexane / ^{i}PrOH, 1.0 mL/min, 254 nm).

<Sample Information>

Sample Name : cyl-09081-2
Sample ID :
Data Filename : cyl-09081-2NEW-IBN-5-90-10-1.0.lcd
Method Filename : cyl-6hao-90-10-1.0ml-90min.lcm
Batch Filename : WWLL.lcb
Vial # : 1-83 Sample Type : Unknown
Injection Volume : 10 uL
Date Acquired : 9/11/2024 10:11:35 Acquired by : System Administrator
Date Processed : 9/11/2024 11:03:19 Processed by : System Administrator

<Chromatogram>

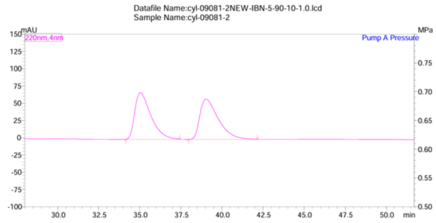

<Peak Table>

PDA Ch2 220nm

Peak#	Ret. Time	Area	Area%
1	35.033	4577233	50.205
2	39.033	4539932	49.795
Total		9117166	100.000

<Sample Information>

Sample Name : cyl-09057
Sample ID :
Data Filename : cyl-09057NEWNEW-IBN-5-90-10-1.0.lcd
Method Filename : cyl-6hao-90-10-1.0ml-90min.lcm
Batch Filename : WWLL.lcb
Vial # : 1-82 Sample Type : Unknown
Injection Volume : 10 uL
Date Acquired : 9/11/2024 9:19:33 Acquired by : System Administrator
Date Processed : 9/11/2024 10:10:45 Processed by : System Administrator

<Chromatogram>

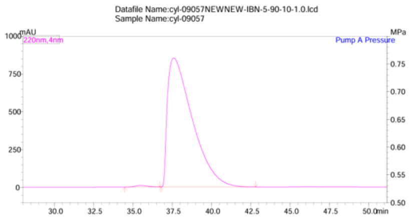

<Peak Table>

PDA Ch2 220nm

Peak#	Ret. Time	Area	Area%
1	35.476	654535	0.691
2	37.583	94105322	99.309
Total		94759858	100.000

(1*R*,5*S*,6*R*)-3-benzyl-6-((*R*)-hydroxy(o-tolyl)methyl)-3-azabicyclo[3.2.0]heptane-2,4-dione (3s)

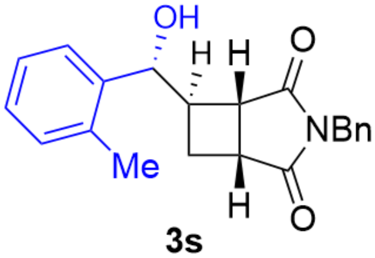

3s

yellow oil; **IR (neat):** 3515.27 (br), 1759.59 (w), 1684.10 (s), 1494.15 (w), 1430.01 (w), 1397.03 (w), 1363.14 (w), 1334.37 (m), 1296.81 (m), 1195.06 (m), 1176.52 (w), 1163.66 (w),1103.35 (w),1086.13 (w), 1066.60 (w), 988.00 (m), 942.18 (m), 926.54 (m), 884.26 (m), 785.34 (w), 751.54 (m), 723.76 (m), 701.51 (m), 652.89 (w), 636.83 (w), 616.66 (w), 454.67 (w), 433.92 (w) cm^{-1};

^{1}H NMR (400 MHz, $CDCl_3$) δ 7.37-7.13 (m, 9 H), 4.95 (d, *J* = 6.8 Hz, 1 H), 4.64 (s, 2 H), 3.43 (dd, *J* = 6.8, 4.4 Hz, 1 H), 3.21-3.16 (m, 1 H), 2.77-2.71 (m, 1 H), 2.64 (br, 1 H), 2.46-2.38 (m, 1H), 2.34 (s, 3 H), 2.15-2.08 (m, 1 H). **^{13}C NMR (100 MHz, $CDCl_3$)** δ 179.6, 179.0, 139.2, 136.0, 135.4, 130.9, 128.77, 128.75, 128.1, 128.0, 126.5, 125.6, 71.8, 42.6, 42.4, 41.0, 35.9, 25.7, 19.3.

HRMS (ESI^{+}) [M+Na]$^{+}$ Calcd for $C_{21}H_{21}NO_3Na$: 358.1414 m/z, Found: 358.1412 m/z;

Specific rotation: $[\alpha]_D^{27.8}$ -6.4 (*c* 0.50, $CHCl_3$) for an enantiomerically enriched sample of 99:1 e.r.

Enantiomeric purity of **3s** was determined by HPLC analysis in comparison with authentic racemic material (99:1 e.r. shown; Chiralpak IB N-5 column, 90:10 hexane / ^{i}PrOH, 1.0 mL/min, 220 nm).

<Sample Information>

Sample Name : CYL-09049-1
Sample ID :
Data Filename : CYL-09049-1rac-IBN-5-90-10-1.0.lcd
Method Filename : ZJW-4-9010-1.0-80-1min.lcm
Batch Filename : ZJW.lcb
Vial # : 1-28 Sample Type : Unknown
Injection Volume : 20 uL
Date Acquired : 4/19/2024 4:56:10 PM Acquired by : System Administrator
Date Processed : 4/19/2024 5:39:25 PM Processed by : System Administrator

<Chromatogram>

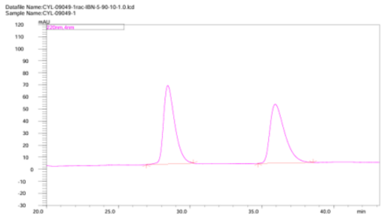

<Peak Table>

PDA Ch2 220nm

Peak#	Ret. Time	Area	Area%
1	28.444	3544540	50.365
2	35.915	3493230	49.635
Total		7037771	100.000

<Sample Information>

Sample Name : CYL-09039
Sample ID :
Data Filename : CYL-09039New-IBN-5-90-10-1.0.lcd
Method Filename : ZJW-4-9010-1.0-50-1min.lcm
Batch Filename : ZJW.lcb
Vial # : 1-28 Sample Type : Unknown
Injection Volume : 20 uL
Date Acquired : 4/19/2024 5:41:48 PM Acquired by : System Administrator
Date Processed : 4/19/2024 6:31:50 PM Processed by : System Administrator

<Chromatogram>

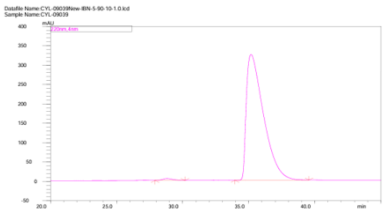

<Peak Table>

PDA Ch2 220nm

Peak#	Ret. Time	Area	Area%
1	28.778	290596	0.984
2	35.167	29253594	99.016
Total		29544190	100.000

(1*R*,5*S*,6*R*)-3-benzyl-6-((*R*)-hydroxy(naphthalen-1-yl)methyl)-3-azabicyclo[3.2.0]heptane-2,4-dione (3t)

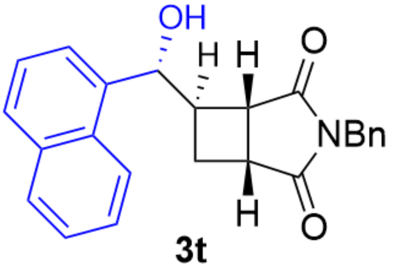

3t

yellow oil; **IR (neat):** 3454.61 (br), 3067.40 (w), 1770.41 (w), 1699.17 (s), 1584.85 (w), 1549.46 (w), 1511.08 (w), 1428.06 (w), 1389.52 (w), 1346.70 (w), 1312.32 (w), 1289.14 (w), 1208.31 (w), 1164.64 (w), 1123.77 (w), 1002.05 (w), 955.91 (w), 935.24 (w), 912.94 (w), 865.39 (w), 781.25 (m), 747.94 (m), 705.73 (w), 661.67 (w), 621.43 (w), 569.00 (w), 468.56 (w) cm^{-1};

^{1}H NMR (400 MHz, $CDCl_3$) δ 8.07-8.04 (m, 1 H), 7.85-7.81 (m, 1 H), 7.77 (d, *J* = 8.0 Hz, 1 H), 7.53-7.40 (m, 4 H), 7.32-7.22 (m, 5 H), 5.41 (d, *J* = 5.6 Hz, 1 H), 4.62-4.58 (m, 2 H), 3.40 (dd, *J* = 6.4, 3.6 Hz, 1 H), 3.16-3.10 (m, 1 H), 2.96-2.89 (m, 1 H), 2.51-2.44 (m, 1 H), 2.14-2.07 (m, 1 H). **^{13}C NMR (100 MHz, $CDCl_3$)** δ 179.6, 178.9, 136.5, 136.0, 134.0, 130.8, 129.2, 128.9, 128.8, 128.0, 126.5, 125.9, 125.4, 123.7, 123.1, 72.0, 42.6, 42.5, 41.0, 35.9, 25.9.

HRMS (ESI$^+$) [M+Na]$^+$ Calcd for $C_{24}H_{21}NO_3Na$: 394.1414 m/z, Found: 394.1422 m/z;

Specific rotation: $[\alpha]_D^{26.4}$ -5.1 (*c* 0.50, $CHCl_3$) for an enantiomerically enriched sample of 99:1 e.r.

Enantiomeric purity of **3t** was determined by HPLC analysis in comparison with authentic racemic material (99:1 e.r. shown; Chiralpak IB N-5 column, 80:20 hexane / ^{i}PrOH, 1.0 mL/min, 254 nm).

<Sample Information>

Sample Name : CYL-09081-11
Sample ID :
Data Filename : CYL-09081-11-IBN-5-80-20-1.0.lcd
Method Filename : ZJW-4-8020-1.0-60-1min.lcm
Batch Filename : ZJW.lcb
Vial # : 1-98
Sample Type : Unknown
Injection Volume : 20 uL
Date Acquired : 5/7/2024 4:23:08 PM
Acquired by : System Administrator
Date Processed : 5/7/2024 5:23:11 PM
Processed by : System Administrator

<Chromatogram>

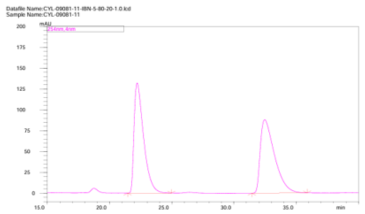

<Peak Table>

PDA Ch1 254nm

Peak#	Ret. Time	Area	Area%
1	22.209	7029238	50.128
2	32.462	6993442	49.872
Total		14022680	100.000

<Sample Information>

Sample Name : CYL-09070
Sample ID :
Data Filename : CYL-09070-IBN-5-80-20-1.0.lcd
Method Filename : ZJW-4-8020-1.0-60-1min.lcm
Batch Filename : ZJW.lcb
Vial # : 1-76
Sample Type : Unknown
Injection Volume : 20 uL
Date Acquired : 5/7/2024 5:53:41 PM
Acquired by : System Administrator
Date Processed : 5/7/2024 6:37:25 PM
Processed by : System Administrator

<Chromatogram>

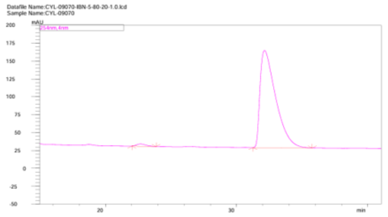

<Peak Table>

PDA Ch1 254nm

Peak#	Ret. Time	Area	Area%
1	22.686	153433	1.343
2	32.128	11273504	98.657
Total		11426937	100.000

(1*R*,5*S*,6*R*)-3-benzyl-6-((*R*)-furan-2-yl(hydroxy)methyl)-3-azabicyclo[3.2.0]heptane-2,4-dione (3u)

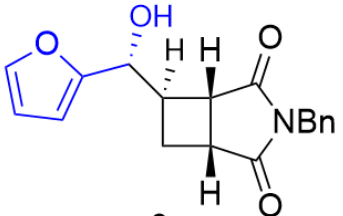

3u

pale yellow oil; **IR (neat):** 3483.34 (br), 2926.54 (w), 1763.10 (w), 1689.90 (s), 1609.69 (w), 1496.48 (w), 1453.06 (w), 1426.91 (w), 1391.99 (m), 1356.89 (w), 1341.37 (w), 1235.97 (w), 1163.89 (m), 1143.59 (m), 1089.33 (w), 1071.09 (w), 1049.78 (w), 1014.95 (w), 994.96 (w), 930.55 (w), 884.26 (w), 854.70 (w), 817.94 (w), 735.78 (w), 697.26 (m), 674.49 (m), 655.01 (w), 626.15 (w), 597.76 (w), 574.42 (w), 548.25 (w), 469.87 (w), 415.86 (w) cm^{-1};
^{1}H NMR (400 MHz, $CDCl_3$) δ7.33-7.19 (m, 6 H), 6.36-6.27 (m, 2 H), 4.71 (d, *J* = 4.0 Hz, 1 H), 4.63 (s, 2 H), 3.36-3.34 (m, 1 H), 3.17-3.11 (m, 1 H), 2.77-2.73 (m, 1 H), 2.55-2.48 (m, 1 H), 2.37 (br, 1 H), 2.17-2.10 (m, 1 H). **^{13}C NMR (100 MHz, $CDCl_3$)** δ 179.5, 179.0, 154.0, 142.6, 136.0, 128.81, 128.76, 128.1, 110.5, 107.2, 68.9, 42.7, 42.6, 40.8, 36.1, 25.4.
HRMS (ESI$^+$) [M+Na]$^+$ Calcd for $C_{18}H_{17}NO_4Na$: 334.1050 m/z, Found: 334.1053 m/z;
Specific rotation: $[\alpha]_D^{26.0}$ -2.6 (*c* 0.50, $CHCl_3$) for an enantiomerically enriched sample of 99:1 e.r.
Enantiomeric purity of **3u** was determined by HPLC analysis in comparison with authentic racemic material (99:1 e.r. shown; Chiralpak IB N-5 column, 90:10 hexane / ^{i}PrOH, 1.0 mL/min, 220 nm).

<Sample Information>

Sample Name : CYL-09084-1rac
Sample ID :
Data Filename : CYL-09084-1rac-IBN-5-90-10-1.0.lcd
Method Filename : ZJW-4-9010-1.0-80-1min.lcm
Batch Filename : ZJW.lcb
Vial # : 1-92 Sample Type : Unknown
Injection Volume : 20 uL
Date Acquired : 4/26/2024 2:53:15 PM Acquired by : System Administrator
Date Processed : 4/26/2024 4:17:16 PM Processed by : System Administrator

<Chromatogram>

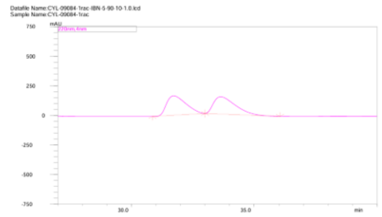

<Peak Table>

PDA Ch2 220nm

Peak#	Ret. Time	Area	Area%
1	31.715	8948898	49.872
2	33.639	8994941	50.128
Total		17943839	100.000

<Sample Information>

Sample Name : CYL-09055
Sample ID :
Data Filename : CYL-09055-IBN-5-90-10-1.0.lcd
Method Filename : ZJW-4-9010-1.0-80-1min.lcm
Batch Filename : ZJW.lcb
Vial # : 1-91 Sample Type : Unknown
Injection Volume : 20 uL
Date Acquired : 4/26/2024 1:32:40 PM Acquired by : System Administrator
Date Processed : 8/12/2024 8:57:28 PM Processed by : System Administrator

<Chromatogram>

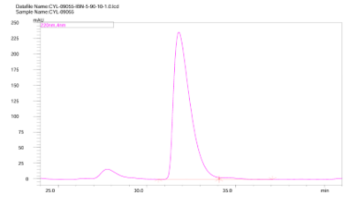

<Peak Table>

PDA Ch2 220nm

Peak#	Ret. Time	Area	Area%
1	31.790	15195278	98.801
2	34.076	184379	1.199
Total		15379657	100.000

(1*R*,5*S*,6*R*)-6-((*R*)-benzofuran-3-yl(hydroxy)methyl)-3-benzyl-3-azabicyclo[3.2.0]heptane-2,4-dione (3v)

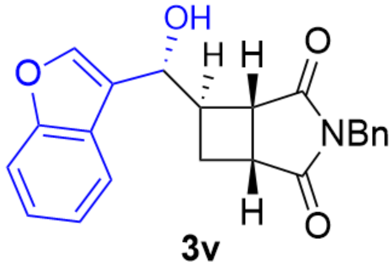

3v

yellow oil; **IR (neat):** 3438.87 (br), 2926.65 (w), 2855.18 (w), 1767.18 (w), 1692.37 (s), 1580.32 (w), 1495.26 (w), 1452.23 (w), 1430.86 (m), 1392.38 (w), 1341.86 (w), 1311.28 (w), 1291.52 (w), 1165.31 (m), 1101.28 (w), 993.23 (w), 935.12 (w), 857.84 (w), 749.01 (m), 701.98 (w), 653.93 (w), 622.23 (w), 476.01 (w), 424.33 (w) cm^{-1};
^{1}H NMR (400 MHz, $CDCl_3$) δ 7.71 (s, 1 H), 7.64-7.62 (m, 1 H), 7.46 (d, *J* = 6.8 Hz, 1 H), 7.38-7.35 (m, 2 H), 7.32-7.25 (m, 4 H), 7.24-7.19 (m, 1 H), 4.95 (d, *J* = 6.4 Hz, 1 H), 4.67 (s, 2 H), 3.38 (dd, *J* = 6.8, 4.4 Hz, 1 H), 3.21-3.15 (m, 1 H), 2.91-2.84 (m, 1 H), 2.56-2.48 (m, 1 H), 2.20-2.13 (m, 1 H). **^{13}C NMR (100 MHz, $CDCl_3$)** δ 179.4, 179.1, 155.7, 142.2, 135.9, 128.8, 128.7, 128.1, 126.2, 124.9, 123.0, 120.8, 120.2, 111.9, 68.2, 42.7, 41.9, 41.0, 36.0, 25.7.
HRMS (ESI$^+$) [M+Na]$^+$ Calcd for $C_{22}H_{19}NO_4Na$: 384.1206 m/z, Found: 384.1208 m/z;
Specific rotation: $[\alpha]_D^{29.6}$ -13.4 (*c* 0.50, $CHCl_3$) for an enantiomerically enriched sample of >99.5:0.5 e.r.
Enantiomeric purity of **3v** was determined by HPLC analysis in comparison with authentic racemic material (>99.5:0.5 e.r. shown; Chiralpak IB N-5 column, 90:10 hexane / ^{i}PrOH, 1.0 mL/min, 254 nm).

<Sample Information>

Sample Name : CYL-09081-13
Sample ID :
Data Filename : CYL-09081-13-RAC-IBN-5-90-10-1.0.lcd
Method Filename : ZJW-4-9010-1.0-80-1min.lcm
Batch Filename : ZJW.lcb
Vial # : 1-97
Injection Volume : 20 uL
Date Acquired : 5/7/2024 10:25:04 PM
Date Processed : 5/7/2024 11:45:06 PM
Sample Type : Unknown
Acquired by : System Administrator
Processed by : System Administrator

<Chromatogram>

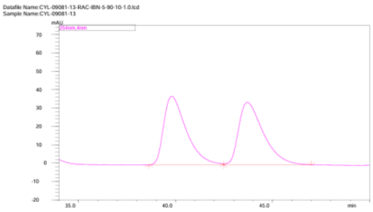

<Peak Table>

PDA Ch1 254nm

Peak#	Ret. Time	Area	Area%
1	39.798	3009986	50.003
2	43.692	3009682	49.997
Total		6019669	100.000

<Sample Information>

Sample Name : CYL-09074
Sample ID :
Data Filename : CYL-09074X-IBN-5-90-10-1.0.lcd
Method Filename : ZJW-4-9010-1.0-80-1min.lcm
Batch Filename : ZJW.lcb
Vial # : 1-98
Injection Volume : 20 uL
Date Acquired : 5/7/2024 11:45:37 PM
Date Processed : 5/8/2024 1:05:40 AM
Sample Type : Unknown
Acquired by : System Administrator
Processed by : System Administrator

<Chromatogram>

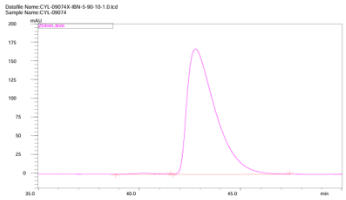

<Peak Table>

PDA Ch1 254nm

Peak#	Ret. Time	Area	Area%
1	40.215	72029	0.444
2	42.768	16154943	99.556
Total		16226972	100.000

(1*R*,5*S*,6*R*)-3-benzyl-6-((*R*)-hydroxy(thiophen-2-yl)methyl)-3-azabicyclo[3.2.0]heptane-2,4-dione (3w)

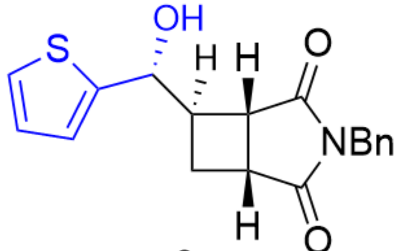

3w

yellow oil; **IR (neat):** 3568.59 (br), 1766.86 (w), 1688.83 (s), 1493.83 (w), 1431.64 (m), 1390.42 (w), 1338.71 (w), 1292.97 (m), 1271.15 (w), 1165.67 (m), 1061.58 (w), 1044.01 (w), 1020.91 (w), 992.04 (w), 922.07 (w), 836.83 (w), 748.28 (w), 729.61 (m), 714.76 (s), 696.46 (w), 648.75 (m), 624.03 (w), 577.64 (w), 522.94 (w), 476.86 (w), 450.99 (w) cm^{-1};

^{1}H NMR (400 MHz, $CDCl_3$) δ 7.39-7.36 (m, 2 H), 7.33-7.25 (m, 4 H), 7.02 (d, *J* = 3.6 Hz, 1 H), 6.98-6.96 (m, 1 H), 4.98 (d, *J* = 7.2 Hz, 1 H), 4.68 (s, 2 H), 3.41 (dd, *J* = 6.8, 3.2 Hz, 1 H), 3.22-3.16 (m, 1 H), 2.78-2.71 (m, 1 H), 2.53-2.45 (m, 1 H), 2.20-2.14 (m, 1 H). **^{13}C NMR (100 MHz, $CDCl_3$)** δ 179.4, 178.7, 144.9, 136.0, 128.81, 128.80, 128.1, 127.0, 125.5, 124.7, 71.8, 43.7, 42.7, 41.3, 35.8, 25.8.

HRMS (ESI$^+$) [M+Na]$^+$ Calcd for $C_{18}H_{17}NO_3NaS$: 350.0821 m/z, Found: 350.0828 m/z;

Specific rotation: $[\alpha]_D^{28.0}$ -10.4 (*c* 0.50, $CHCl_3$) for an enantiomerically enriched sample of 99:1 e.r.

Enantiomeric purity of **3w** was determined by HPLC analysis in comparison with authentic racemic material (99:1 e.r. shown; Chiralpak IE column, 90:10 hexane / ^{i}PrOH, 1.0 mL/min, 220 nm).

<Sample Information>

Sample Name : cyl-09081-9
Sample ID :
Data Filename : cyl-09081-9-IE-90-10-1.0.lcd
Method Filename : cyl-5hao-90-10-1.0ml-80min.lcm
Batch Filename : WWLL.lcb
Vial # : 1-79
Sample Type : Unknown
Injection Volume : 10 uL
Date Acquired : 9/12/2024 16:09:27
Acquired by : System Administrator
Date Processed : 9/13/2024 8:54:53
Processed by : System Administrator

<Chromatogram>

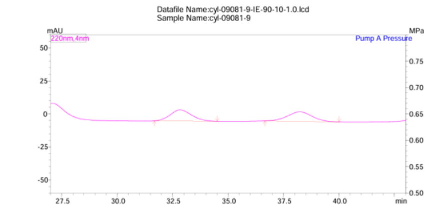

<Peak Table>

PDA Ch2 220nm

Peak#	Ret. Time	Area	Area%
1	32.819	564579	50.251
2	38.217	558948	49.749
Total		1123527	100.000

<Sample Information>

Sample Name : cyl-09066
Sample ID :
Data Filename : cyl-09066NEW2-IE-90-10-1.0.lcd
Method Filename : cyl-5hao-90-10-1.0ml-60min.lcm
Batch Filename : WWLL.lcb
Vial # : 1-79
Sample Type : Unknown
Injection Volume : 20 uL
Date Acquired : 9/12/2024 19:03:25
Acquired by : System Administrator
Date Processed : 9/12/2024 19:49:51
Processed by : System Administrator

<Chromatogram>

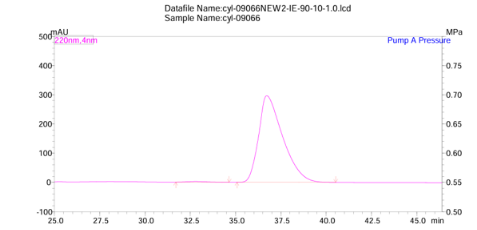

<Peak Table>

PDA Ch2 220nm

Peak#	Ret. Time	Area	Area%
1	32.786	186086	0.672
2	36.719	27517485	99.328
Total		27703572	100.000

tert-butyl 3- ((*R*)- ((1*R*,5*S*,6*R*)-3-benzyl-2,4-dioxo-3-azabicyclo[3.2.0]heptan-6-yl) (hydroxy)methyl)-1H-indole-1-carboxylate (3x)

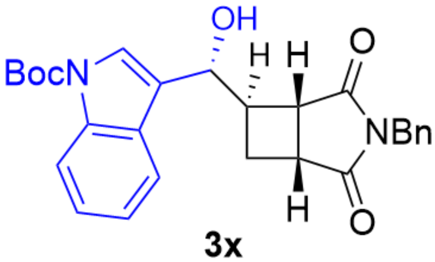

3x

white solid, m.p.85-86 °C; **IR (neat):** 3416.78 (br), 2977.39 (w), 2928.73 (w), 1730.52 (s), 1682.35 (s), 1606.67 (w), 1567.55 (w), 1495.88 (m), 1475.97 (w), 1452.50 (s), 1428.49 (m), 1393.54 (m), 1369.27 (m), 1334.69 (m), 1306.61 (m), 1254.14 (m), 1222.99 (s), 1152.89 (m), 1101.77 (m), 1071.58 (m), 1019.87 (m), 974.01 (w), 935.21 (w), 847.82 (w), 795.69 (w), 748.08 (m), 727.74 (w), 696.81 (w), 616.94 (w), 584.87 (w), 473.33 (w), 424.00 (w) cm^{-1};

^{1}H NMR (400 MHz, $CDCl_3$) δ 8.04 (d, *J* = 9.2 Hz, 1 H), 7.65 (s, 1 H), 7.55 (d, *J* = 7.6 Hz, 1 H) , 7.31-7.12 (m, 7 H), 4.90 (d, *J* = 4.8 Hz, 1 H), 4.61 (s, 2 H), 3.36 (dd, *J* = 6.4, 4.4 Hz, 1 H), 3.16-3.10 (m, 1 H), 2.87-2.81 (m, 1 H), 2.50-2.43 (m, 1 H), 2.33 (br, 1 H), 2.14-2.07 (m, 1 H), 1.59 (s, 9 H). **^{13}C NMR (100 MHz, $CDCl_3$)** δ 179.5, 178.9, 136.1, 128.8, 128.1, 125.0, 123.5, 122.9, 120.5, 119.5, 115.6, 84.2, 69.1, 42.7, 42.0, 41.1, 36.1, 28.3, 25.8.

HRMS (ESI^{+}) [M+Na]$^{+}$ Calcd for $C_{27}H_{28}N_2O_5Na$: 483.1890 m/z, Found: 483.1884 m/z;

Specific rotation: $[\alpha]_D^{28.0}$ -10.6 (*c* 0.50, $CHCl_3$) for an enantiomerically enriched sample of >99.5:0.5 e.r.

Enantiomeric purity of **3x** was determined by HPLC analysis in comparison with authentic racemic material (>99.5:0.5 e.r. shown; Chiralpak IB N-5 column, 90:10 hexane / ^{i}PrOH, 1.0 mL/min, 220 nm).

<Sample Information>

Sample Name : cyl-09081-6
Sample ID :
Data Filename : cyl-09081-6NEW-IBN-5-90-10-1.0.lcd
Method Filename : cyl-6hao-90-10-1.0ml-70min.lcm
Batch Filename : WWLL.lcb
Vial # : 1-85 Sample Type : Unknown
Injection Volume : 10 uL
Date Acquired : 9/11/2024 11:56:21 Acquired by : System Administrator
Date Processed : 9/11/2024 12:37:42 Processed by : System Administrator

<Chromatogram>

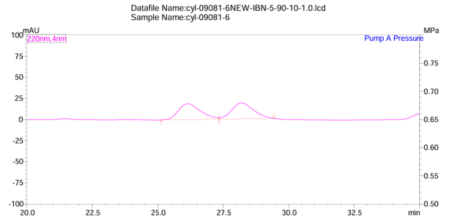

<Peak Table>

PDA Ch2 220nm

Peak#	Ret. Time	Area	Area%
1	26.136	1105283	50.150
2	28.195	1098691	49.850
Total		2203975	100.000

<Sample Information>

Sample Name : cyl-09062
Sample ID :
Data Filename : cyl-09062NEWNEW-IBN-5-90-10-1.0.lcd
Method Filename : cyl-6hao-90-10-1.0ml-90min.lcm
Batch Filename : WWLL.lcb
Vial # : 1-84 Sample Type : Unknown
Injection Volume : 10 uL
Date Acquired : 9/11/2024 11:04:41 Acquired by : System Administrator
Date Processed : 9/11/2024 11:55:09 Processed by : System Administrator

<Chromatogram>

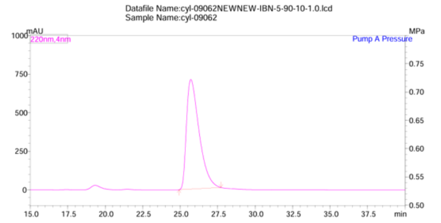

<Peak Table>

PDA Ch2 220nm

Peak#	Ret. Time	Area	Area%
1	25.710	41839992	100.000
Total		41839992	100.000

(1*R*,5*S*,6*R*)-3-benzyl-6-((*R*)-1-hydroxy-3-methylbut-2-en-1-yl)-3-azabicyclo[3.2.0]heptane-2,4-dione (3y)

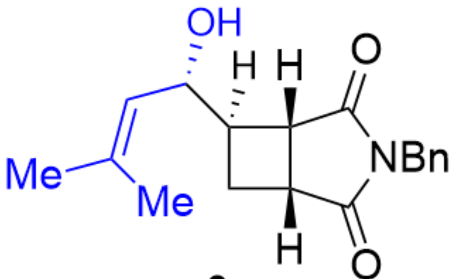

3y

light yellow oil; **IR (neat):** 3451.72 (br), 2930.35 (w), 1767.17 (w), 1696.86 (s), 1431.56 (m), 1391.82 (m), 1342.91 (w), 1311.41 (w), 1292.67 (w), 1166.17 (w), 987.90 (w), 934.81 (w), 700.93 (w), 620.90 (w) cm^{-1};
^{1}H NMR (400 MHz, $CDCl_3$) δ 7.39-7.24 (m, 7 H), 5.17-5.11 (m, 1.4 H), 4.68 (s, 2.8 H), 4.53-4.49 (m, 0.4 H), 4.42-4.38 (m, 1 H), 3.30-3.28 (m, 1 H), 3.19-3.14 (m, 1.8 H), 2.62-2.59 (m, 0.4 H), 2.43-2.35 (m, 2.8 H), 2.15-2.09 (m, 1.4 H), 1.73-1.71 (m, 8.4 H).
^{13}C NMR (100 MHz, $CDCl_3$) δ179.7, 179.2, 138.1, 136.1, 128.8, 128.71, 128.66, 128.0, 124.2, 123.9, 69.8, 69.6, 42.9, 42.6, 42.4, 41.1, 40.9, 36.2, 36.0, 26.0, 25.0, 24.2, 18.7.
HRMS (ESI^+) $[M+Na]^+$ Calcd for $C_{18}H_{21}NO_3Na$: 322.1414 m/z, Found: 322.1412 m/z;
Specific rotation: $[\alpha]_D^{28.2}$ -10.1 (*c* 0.50, $CHCl_3$) for an enantiomerically enriched sample of >99.5:0.5 e.r. shown for major isomer, 97:3 e.r. shown for minor isomer Enantiomeric purity of **3y** was determined by HPLC analysis in comparison with authentic racemic material (>99.5:0.5 e.r. shown for major isomer, 97:3 e.r. shown for minor isomer; Chiralpak IA column, 95:5 hexane / ^{i}PrOH, 1.0 mL/min, 220 nm).

<Sample Information>
Sample Name : CYL-09081-16
Sample ID :
Data Filename : CYL-09081-16RAC-IA-95-5-1.0.lcd
Method Filename : ZJW-1-9505-1.0-60min.lcm
Batch Filename : ZJW.lcb
Vial # : 1-103 Sample Type : Unknown
Injection Volume : 20 uL
Date Acquired : 5/9/2024 2:29:33 PM Acquired by : System Administrator
Date Processed : 5/9/2024 5:37:03 PM Processed by : System Administrator

<Chromatogram>

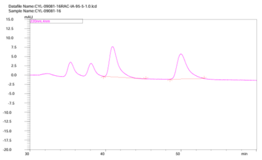

<Peak Table>
PDA Ch2 220nm

Peak#	Ret. Time	Area	Area%
1	40.928	636419	49.554
2	49.934	647862	50.446
Total		1284282	100.000

<Sample Information>
Sample Name : CYL-09040
Sample ID :
Data Filename : CYL-09040-IA-95-5-1.0.lcd
Method Filename : ZJW-1-9505-1.0-60min.lcm
Batch Filename : ZJW.lcb
Vial # : 1-104 Sample Type : Unknown
Injection Volume : 20 uL
Date Acquired : 5/9/2024 4:50:37 PM Acquired by : System Administrator
Date Processed : 7/27/2024 9:15:58 AM Processed by : System Administrator

<Chromatogram>

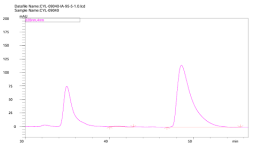

<Peak Table>
PDA Ch2 220nm

Peak#	Ret. Time	Area	Area%
1	40.927	88442	0.646
2	48.577	13607651	99.354
Total		13696093	100.000

Sample Name : [illegible]
Sample ID :
Data Filename : CYL-09081-16RAC-IA-95-5-1.0-MINOR.lcd
Method Filename : ZJW-1-9505-1.0-60min.lcm
Batch Filename : ZJW.lcb
Vial # : 1-103 Sample Type : Unknown
Injection Volume : 20 uL
Date Acquired : 5/9/2024 2:29:33 PM Acquired by : System Administrator
Date Processed : 8/26/2024 3:23:40 PM Processed by : System Administrator

<Chromatogram>

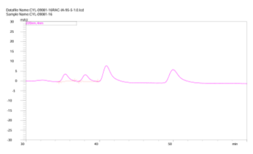

<Peak Table>
PDA Ch2 220nm

Peak#	Ret. Time	Area	Area%
1	35.368	203457	50.460
2	38.089	199751	49.540
Total		403208	100.000

Sample ID : [illegible]
Data Filename : CYL-09040-IA-95-5-1.0-MINOR.lcd
Method Filename : ZJW-1-9505-1.0-60min.lcm
Batch Filename : ZJW.lcb
Vial # : 1-104 Sample Type : Unknown
Injection Volume : 20 uL
Date Acquired : 5/9/2024 4:50:37 PM Acquired by : System Administrator
Date Processed : 8/26/2024 3:27:56 PM Processed by : System Administrator

<Chromatogram>

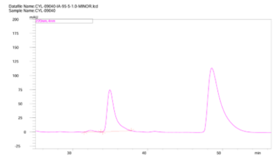

<Peak Table>
PDA Ch2 220nm

Peak#	Ret. Time	Area	Area%
1	32.319	179127	3.283
2	34.946	5276967	96.717
Total		5456094	100.000

(1*R*,5*S*,6*R*)-3-benzyl-6-((*R*,*E*)-1-hydroxy-2-methyl-3-phenylallyl)-3-azabicyclo[3.2.0]heptane-2,4-dione (3z)

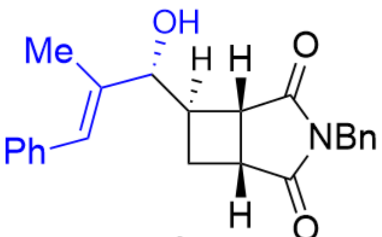

3z

yellow oil; **IR (neat):** 3452.73 (br), 2943.95 (w), 1766.78 (w), 1695.97 (s), 1494.17 (w), 1431.11 (w), 1391.87 (w), 1343.11 (m), 1311.52 (w), 1292.77 (w), 1166.07 (m), 1070.27 (w), 923.24 (w), 750.00 (w), 700.64 (m), 641.25 (w), 621.17 (w) cm^{-1};
^{1}H NMR (400 MHz, $CDCl_3$) δ 7.40-7.19 (m, 10 H), 6.58 (s, 1 H), 4.70 (s, 1 H), 4.22 (d, J = 6.0 Hz, 1 H), 3.36 (dd, J = 6.8, 4.0 Hz, 1 H), 3.23-3.18 (m, 1 H), 2.67-2.60 (m, 1 H), 2.57-2.50 (m, 1 H), 2.21-2.15 (m, 1 H), 1.81 (s, 3 H). **^{13}C NMR (100 MHz, $CDCl_3$)** δ 179.7, 179.1, 137.5, 137.1, 136.1, 129.2, 128.80, 128.77, 128.3, 128.0, 127.1, 126.9, 78.8, 42.6, 40.8, 40.7, 35.9, 25.6, 14.1.
HRMS (ESI$^+$) [M+Na]$^+$ Calcd for $C_{23}H_{23}NO_3Na$: 384.1570 m/z, Found: 384.1574 m/z;
Specific rotation: $[\alpha]_D^{29.5}$ -5.5 (c 0.50, $CHCl_3$) for an enantiomerically enriched sample of >99.5:0.5 e.r.
Enantiomeric purity of **3z** was determined by HPLC analysis in comparison with authentic racemic material (>99.5:0.5 e.r. shown; Chiralpak IB N-5 column, 90:10 hexane / ^{i}PrOH, 1.0 mL/min, 254 nm).

<Sample Information>

Sample Name : CYL-09081-12
Sample ID :
Data Filename : CYL-09081-12-IBN-5-90-10-1.0.lcd
Method Filename : ZJW-4-9010-1.0-60min.lcm
Batch Filename : ZJW.lcb
Vial # : 1-99
Injection Volume : 20 uL
Date Acquired : 5/7/2024 6:59:23 PM
Date Processed : 5/7/2024 7:52:40 PM
Sample Type : Unknown
Acquired by : System Administrator
Processed by : System Administrator

<Chromatogram>

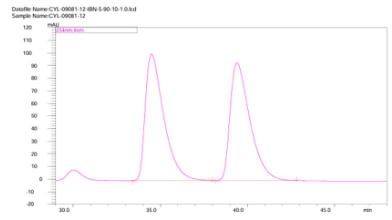

<Peak Table>

PDA Ch1 254nm

Peak#	Ret. Time	Area	Area%
1	34.493	7411588	50.102
2	39.393	7381523	49.898
Total		14793110	100.000

<Sample Information>

Sample Name : CYL-09073
Sample ID :
Data Filename : CYL-09073-IBN-5-90-10-1.0.lcd
Method Filename : ZJW-4-9010-1.0-80-1min.lcm
Batch Filename : ZJW.lcb
Vial # : 1-77
Injection Volume : 20 uL
Date Acquired : 5/6/2024 6:08:55 PM
Date Processed : 8/18/2024 6:37:20 PM
Sample Type : Unknown
Acquired by : System Administrator
Processed by : System Administrator

<Chromatogram>

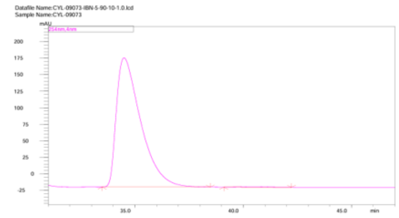

<Peak Table>

PDA Ch1 254nm

Peak#	Ret. Time	Area	Area%
1	34.497	15279365	99.583
2	40.523	63929	0.417
Total		15343294	100.000

(1*R*,5*S*,6*R*)-3-benzyl-6-((*S*)-1-hydroxy-3-phenylpropyl)-3-azabicyclo[3.2.0]heptane-2,4-dione (3aa)

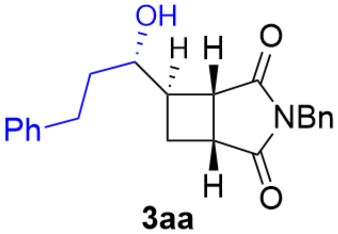

3aa

yellow oil; **IR (neat):** 3516.34 (br), 2926.20 (w), 1762.01 (w), 1683.49 (s), 1603.03 (w), 1494.31 (w), 1452.34 (w), 1427.19 (w), 1396.87 (m), 1364.69 (m), 1337.88 (m), 1296.86 (m), 1274.21 (m), 1196.03 (w), 1175.95 (m), 1126.20 (s), 1095.43 (s), 1079.74 (w), 1058.56 (s), 1037.91 (w), 998.66 (s), 946.85 (w), 925.29 (w), 885.75 (w), 743.34 (m), 730.74 (s), 697.14 (w), 651.09 (m), 637.49 (m), 572.89 (w), 511.92 (w), 492.47 (w), 462.82 (w), 446.64 (w), 416.58 (w) cm^{-1};
^{1}H NMR (400 MHz, $CDCl_3$) δ 7.38-7.16 (m, 13 H), 4.67 (s, 2.8 H), 3.77-3.73 (m, 0.4 H), 3.64-3.59 (m, 1 H), 3.29-3.26 (m, 1 H), 3.13-3.08 (m, 1.8H), 2.81-2.74 (m, 1.4 H), 2.71-2.62 (m, 1.4 H), 2.60-2.51 (m, 1 H), 2.42-2.33 (m, 2.8 H), 2.15-2.04 (m, 1.8 H), 1.76-1.68 (m, 2.8 H) . **^{13}C NMR (100 MHz, $CDCl_3$)** δ 179.6, 179.4, 141.5, 141.4, 136.0, 128.8, 128.67, 128.65, 128.63, 128.60, 128.52, 128.48, 128.0, 126.2, 126.1, 72.2, 72.0, 43.1, 42.59, 42.55, 41.5, 40.5, 36.14, 36.06, 35.9, 35.8, 31.91, 31.87, 25.3, 23.7.
HRMS (ESI^{+}) [M+Na]$^{+}$ Calcd for C_{22}H23NO$_3$Na: 372.1570 m/z, Found: 372.1566 m/z;
Specific rotation:$[\alpha]_D^{28.8}$ -40.0 (*c* 0.50, $CHCl_3$) for an enantiomerically enriched sample of >99.5:0.5 e.r. shown for major isomer, 98:2 e.r. shown for minor isomer Enantiomeric purity of **3aa** was determined by HPLC analysis in comparison with authentic racemic material (>99.5:0.5 e.r. shown for major isomer, 98:2 e.r. shown for minor isomer; Chiralpak IG column, 90:10 hexane / ^{i}PrOH, 1.0 mL/min, 254 nm).

<Sample Information>

Sample Name : cyl-09049-4
Sample ID :
Data Filename : cyl-09049-4NEW-IG-90-10-1.0.lcd
Method Filename : cyl-1hao-90-10-1.0ml-50min.lcm
Batch Filename : WWLL.lcb
Vial # : 1-70
Injection Volume : 10 uL
Date Acquired : 9/9/2024 23:50:40
Date Processed : 9/10/2024 0:40:43
Sample Type : Unknown
Acquired by : System Administrator
Processed by : System Administrator

<Chromatogram>

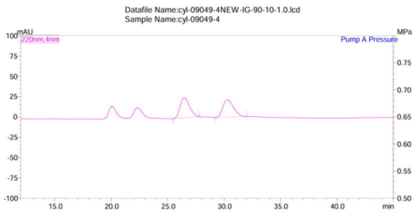

<Peak Table>

PDA Ch2 220nm

Peak#	Ret. Time	Area	Area%
1	26.471	1456340	50.237
2	30.301	1442575	49.763
Total		2898915	100.000

<Sample Information>

Sample Name : cyl-09034
Sample ID :
Data Filename : cyl-09034NEW-IG-90-10-1.0.lcd
Method Filename : cyl-1hao-90-10-1.0ml-50min.lcm
Batch Filename : WWLL.lcb
Vial # : 1-69
Injection Volume : 10 uL
Date Acquired : 9/9/2024 23:00:07
Date Processed : 9/10/2024 14:23:05
Sample Type : Unknown
Acquired by : System Administrator
Processed by : System Administrator

<Chromatogram>

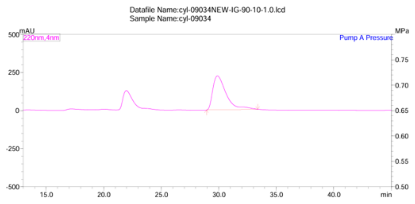

<Peak Table>

PDA Ch2 220nm

Peak#	Ret. Time	Area	Area%
1	29.890	18021892	100.000
Total		18021892	100.000

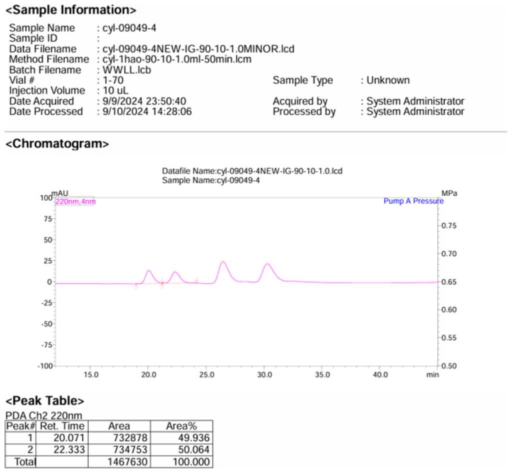

Peak#	Ret. Time	Area	Area%
1	20.071	732878	49.936
2	22.333	734753	50.064
Total		1467630	100.000

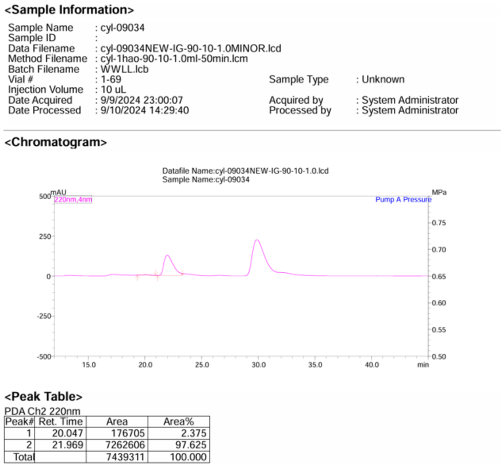

Peak#	Ret. Time	Area	Area%
1	20.047	176705	2.375
2	21.969	7262606	97.625
Total		7439311	100.000

(1*R*,5*S*,6*R*)-3-benzyl-6-((*S*)-1-hydroxy-2-methylpropyl)-3-azabicyclo[3.2.0]heptane-2,4-dione (3ab)

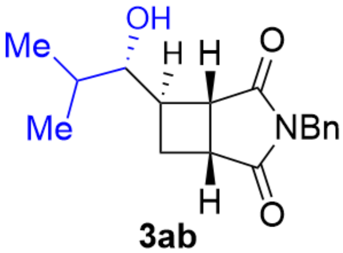

3ab

yellow oil; **IR (neat):** 3496.04 (br), 2959.05 (w), 2873.98 (w), 1766.46 (w), 1690.71 (s), 1495.72 (w), 1430.37 (m), 1390.94 (m), 1340.68 (w), 1311.43 (w), 1293.62 (w), 1261.15 (w), 1165.09 (m), 1067.04 (w), 1013.61 (w), 992.78 (w), 932.52 (w), 803.33 (w), 747.53 (w), 699.54 (m), 652.06 (w), 620.39 (w), 475.18 (w) cm^{-1};
^{1}H NMR (400 MHz, $CDCl_3$) δ 7.32 – 7.18 (m, 7 H), 4.62 (s, 2.9 H), 3.45-3.43 (m, 0.8 H), 3.30-3.23 (m, 1.3 H), 3.14-3.04 (m, 2 H), 2.60-2.37 (m, 2.9 H), 2.13-2.97 (m, 2.9 H), 1.65-1.60 (m, 1.7 H), 0.87-0.84 (m, 6 H), 0.80-0.79 (m, 2.4 H). **^{13}C NMR (100 MHz, $CDCl_3$)** δ 179.8, 179.5, 179.4, 136.1, 128.8, 128.72, 128.68, 128.0, 78.0, 77.7, 42.6, 42.5, 41.8, 40.7, 40.5, 40.4, 36.2, 36.0, 31.73, 31.67, 25.8, 23.8, 19.30, 19.25, 17.7, 17.5.
HRMS (ESI^+) $[M+Na]^+$ Calcd for $C_{17}H_{21}NO_3Na$: 310.1414 m/z, Found: 310.1421 m/z;
Specific rotation: $[\alpha]_D^{27.2}$ -28.5 (*c* 0.50, $CHCl_3$) for an enantiomerically enriched sample of >99.5:0.5 e.r. shown for major isomer, >99.5:0.5 e.r. shown for minor isomer. Enantiomeric purity of **3ab** was determined by HPLC analysis in comparison with authentic racemic material (>99.5:0.5 e.r. shown for major isomer, >99.5:0.5 e.r. shown for minor isomer; Chiralpak IE column, 90:10 hexane / ^{i}PrOH, 1.0 mL/min, 220nm).

<Sample Information>

Sample Name : CYL-09081-14
Sample ID :
Data Filename : CYL-09081-14-RACX-IE-5-90-10-1.0.lcd
Method Filename : ZJW-3-9010-1.0-50-1min.lcm
Batch Filename : ZJW.lcb
Vial # : 1-101 Sample Type : Unknown
Injection Volume : 20 uL
Date Acquired : 5/8/2024 3:12:21 PM Acquired by : System Administrator
Date Processed : 5/8/2024 4:41:39 PM Processed by : System Administrator

<Chromatogram>

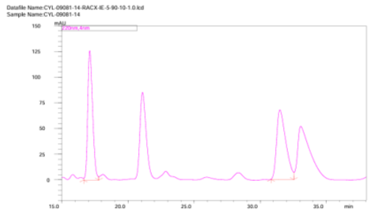

<Peak Table>

PDA Ch2 220nm

Peak#	Ret. Time	Area	Area%
1	17.101	3242915	49.574
2	31.488	3298599	50.426
Total		6541514	100.000

<Sample Information>

Sample Name : CYL-09077
Sample ID :
Data Filename : CYL-09077X-IE-5-90-10-1.0.lcd
Method Filename : ZJW-3-9010-1.0-50-1min.lcm
Batch Filename : ZJW.lcb
Vial # : 1-102 Sample Type : Unknown
Injection Volume : 20 uL
Date Acquired : 5/8/2024 4:02:54 PM Acquired by : System Administrator
Date Processed : 8/18/2024 7:26:30 PM Processed by : System Administrator

<Chromatogram>

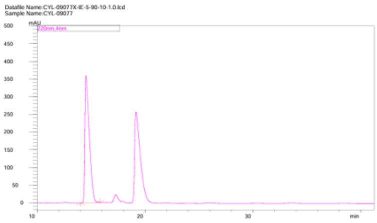

<Peak Table>

PDA Ch2 220nm

Peak#	Ret. Time	Area	Area%
1	14.512	10285988	100.000
Total		10285988	100.000

<Sample Information>

Sample Name : CYL-09081-14
Sample ID :
Data Filename : CYL-09081-14-RACX-IE-5-90-10-1.0-MINOR.lcd
Method Filename : ZJW-3-9010-1.0-50-1min.lcm
Batch Filename : ZJW.lcb
Vial # : 1-101 Sample Type : Unknown
Injection Volume : 20 uL
Date Acquired : 5/8/2024 3:12:21 PM Acquired by : System Administrator
Date Processed : 8/26/2024 8:56:54 PM Processed by : System Administrator

<Chromatogram>

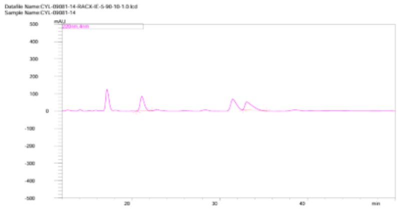

<Peak Table>

PDA Ch2 220nm

Peak#	Ret. Time	Area	Area%
1	21.092	2708458	50.312
2	33.070	2674898	49.688
Total		5383356	100.000

<Sample Information>

Sample Name : CYL-09077
Sample ID :
Data Filename : CYL-09077X-IE-5-90-10-1.0-MINOR.lcd
Method Filename : ZJW-3-9010-1.0-50-1min.lcm
Batch Filename : ZJW.lcb
Vial # : 1-102 Sample Type : Unknown
Injection Volume : 20 uL
Date Acquired : 5/8/2024 4:02:54 PM Acquired by : System Administrator
Date Processed : 8/26/2024 8:58:29 PM Processed by : System Administrator

<Chromatogram>

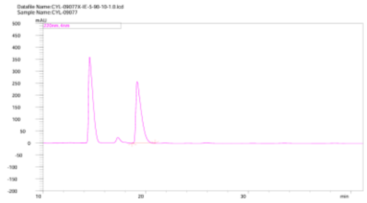

<Peak Table>

PDA Ch2 220nm

Peak#	Ret. Time	Area	Area%
1	19.152	9358024	100.000
Total		9358024	100.000

(1*R*,5*S*,6*R*)-3-benzyl-6-((1*S*,3*S*)-1-hydroxy-3,7-dimethyloct-6-en-1-yl)-3-azabicyclo[3.2.0]heptane-2,4-dione (3ac)

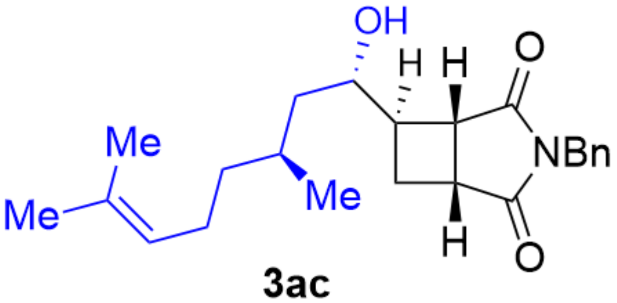

3ac

colorless oil; **IR (neat):** 3835.53 (w), 3821.65 (w), 3796.77 (w), 3779.96 (w), 3765.47 (w), 3756.25 (w), 3745.45 (w), 3735.90 (w), 3723.18 (w), 3701.33 (w), 3680.11 (w), 3670.25 (w), 3660.66 (w), 3644.87 (w), 3633.56 (w), 3594.36 (w), 3583.92 (w), 3570.90 (w), 3551.91 (w), 3522.76 (w), 3503.84 (w), 3477.09 (w), 3441.92 (w), 2917.85 (w), 1698.68 (s), 1392.02 (m), 1341.39 (m), 1166.10 (m) cm^{-1};
^{1}H NMR (400 MHz, $CDCl_3$) δ 7.32-7.18 (m, 7 H), 5.05-4.99 (m, 1.3 H), 4.62-4.61 (m, 2.6 H), 3.82-3.78 (m, 0.3 H), 3.68-3.64 (m, 1 H), 3.23-3.20 (m, 1 H), 3.10-3.05 (m, 1.7 H), 2.55-2.48 (m, 0.4 H), 2.41-2.33 (m, 1 H), 2.30-2.22 (m, 1.3 H), 2.19 (br, 1.3 H), 2.11-2.02 (m, 1.4 H), 2.00-1.82 (m, 2.8 H), 1.62 (s, 4 H), 1.54 (s, 4 H), 1.40-1.30 (m, 1.4 H), 1.28-1.14 (m, 2.9 H), 1.09-0.99 (m, 1.4 H), 0.86 (d, *J* = 6.8 Hz, 3 H), 0.84 (d, *J* = 6.8 Hz, 1 H) . **^{13}C NMR (100 MHz, $CDCl_3$)** δ 179.8, 179.7, 179.4, 179.3, 136.0, 131.5, 128.77, 128.76, 128.7, 128.6, 128.0, 124.7, 124.6, 71.0, 70.5, 43.3, 43.1, 42.6, 42.5, 42.2, 41.4, 40.4, 38.5, 37.9, 36.4, 36.1, 35.9, 29.2, 28.8, 25.8, 25.5, 25.4, 23.7, 23.0, 20.4, 19.1, 17.8.
HRMS (ESI^{+}) [M+Na]$^{+}$ Calcd for $C_{23}H_{31}NO_3Na$:392.2196 m/z, Found: 392.2195 m/z;
Specific rotation: $[\alpha]_D^{26.5}$-125.4 (*c* 0.25, $CHCl_3$)

(1*R*,5*S*,6*R*)-3-benzyl-6-((1*R*,2*S*)-2-((tert-butyldimethylsilyl)oxy)-1-hydroxypropyl)-3-azabicyclo[3.2.0]heptane-2,4-dione (3ad)

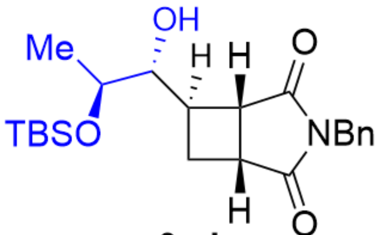

3ad

pale yellow oil; **IR (neat):** 3534.44 (br), 2949.47 (w), 2928.28 (w), 2883.38 (w), 2855.80 (w), 1753.95 (w), 1684.43 (s), 1456.69 (m), 1429.86 (m), 1392.46 (m), 1340.74 (m), 1308.11 (m), 1254.42 (m), 1160.87 (m), 1144.24 (m), 1108.72 (m), 1071.74 (s), 1006.24 (w), 980.05 (w), 965.56 (w), 939.82 (w), 900.37 (w), 829.98 (s), 811.04 (m), 774.16 (m), 748.06 (m), 712.57 (m), 696.85 (m), 648.14 (m), 620.68 (m), 567.65 (w), 473.55 (m) cm^{-1};
^{1}H NMR (400 MHz, $CDCl_3$) δ 7.38 – 7.24 (m, 6 H), 4.68 (s, 2.4 H), 3.85-3.79 (m, 1 H), 3.69-3.65 (m, 1.2 H), 3.35-3.30 (m, 0.4 H), 3.27-3.25 (m, 0.2 H), 3.19-3.13 (m, 2 H), 2.76-2.65 (m, 1.2 H), 2.56-2.47 (m, 2.4 H), 2.13-2.07 (m, 1.2 H), 1.12 (d, *J* = 6.8 Hz, 0.6 H), 1.02 (d, *J* = 6.8 Hz, 3 H), 0.86 (s, 11 H), 0.05-0.04 (m, 7 H). **^{13}C NMR (100 MHz, $CDCl_3$)** δ 179.6, 179.1, 136.1, 128.8, 128.7, 128.0, 76.1, 69.9, 69.2, 42.6, 41.6, 40.1, 39.3, 38.5, 36.4, 36.1, 25.89, 25.86, 25.4, 24.1, 23.0, 20.4, 18.04, 18.00, -4.3, -4.8.
HRMS (ESI$^+$) [M+Na]$^+$ Calcd for $C_{22}H_{33}NO_4NaSi$: 426.2071 m/z, Found: 426.2073 m/z;
Specific rotation: $[\alpha]_D^{28.1}$ -51.8 (*c* 0.50, $CHCl_3$)

tert-butyl (*S*)-2- ((*R*)- ((1*R*,5*S*,6*R*)-3-benzyl-2,4-dioxo-3-azabicyclo[3.2.0]heptan-6-yl) (hydroxy)methyl)pyrrolidine-1-carboxylate (3ae)

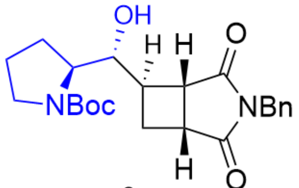

3ae

pale yellow oil; **IR (neat):** 3528.64 (br), 2972.67 (w), 2929.23 (w), 1762.46 (w), 1685.52 (s), 1452.18 (w), 1427.99 (s), 1392.55 (m), 1363.96 (m), 1339.35 (w), 1299.10 (w), 1254.63 (w), 1208.32 (w), 1163.64 (w), 1125.39 (m), 1095.30 (m), 970.67 (w), 940.85 (w), 911.42 (w), 827.54 (w), 805.94 (w), 775.95 (w), 728.17 (w), 715.84 (w), 694.75 (w), 636.37 (w), 460.92 (w), 446.78 (w) cm^{-1};
^{1}H NMR (500 MHz, $CDCl_3$) δ 7.39-7.26 (m, 5 H), 5.20 (br, 1 H), 4.68 (s, 2 H), 4.01-3.98 (m, 1 H), 3.77 (br, 1 H), 3.48-3.44 (m, 2 H), 3.32-3.29 (m, 1 H), 3.22-3.17 (m, 1 H), 2.68-2.61 (m, 1 H), 2.43 (br, 1 H), 2.18-2.14 (m, 1 H), 2.00-1.94 (m, 1 H), 1.79-1.70 (m, 2 H), 1.64-1.61 (m, 1 H), 1.46 (s, 9 H). **^{13}C NMR (125 MHz, $CDCl_3$)** δ 179.8, 179.2, 157.3, 136.2, 128.7, 128.6, 127.9, 80.6, 74.7, 61.6, 48.2, 42.4, 39.9, 38.5, 36.7, 28.5, 28.0, 26.1, 24.3, 14.3.
HRMS (ESI^{+}) [M+Na]$^{+}$ Calcd for $C_{23}H_{30}N_2O_5Na$: 437.2047 m/z, Found: 437.2039 m/z;
Specific rotation: $[\alpha]_D^{28.7}$ -39.4 (*c* 0.20, $CHCl_3$)

tert-butyl (*S*)-2- ((*S*)- ((1*S*,5*R*,6*S*)-3-benzyl-2,4-dioxo-3-azabicyclo[3.2.0]heptan-6-yl) (hydroxy)methyl)pyrrolidine-1-carboxylate (dia-3ae)

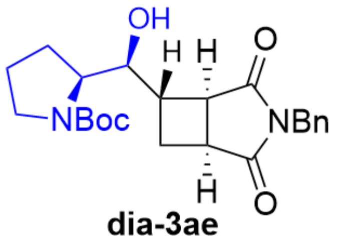

dia-3ae

pale yellow oil; **IR (neat):** 3456.32 (br), 2974.83 (w), 2931.33 (w), 1768.69 (w), 1695.96 (s), 1477.68 (w), 1452.92 (w), 1391.24 (m), 1366.41 (m), 1342.69 (m), 1311.75 (w), 1251.67 (w), 1164.99 (m), 1107.98 (w), 1001.48 (w), 932.32 (w), 773.10 (w), 699.54 (w), 619.61 (w) cm^{-1};

^{1}H NMR (400 MHz, $CDCl_3$) δ 7.33-7.27 (m, 2 H), 7.24-7.18 (m, 3 H) 5.01 (br, 1 H), 4.62 (s, 2 H), 4.01-3.97 (m, 1 H), 3.81 (br, 1 H), 3.40-3.37 (m, 1 H), 3.25-3.22 (m, 1 H), 3.12-3.08 (m, 1 H), 3.07-2.98 (m, 1 H),2.66-2.58 (m, 1 H), 2.34 (br, 1 H), 1.98-1.95 (m, 2 H), 1.69-1.62 (m, 2 H), 1.57-1.52 (m, 1 H), 1.38 (s, 9 H). **^{13}C NMR (125 MHz, $CDCl_3$)** δ 179.9, 179.2, 157.2, 136.1, 128.8, 128.7, 128.0, 80.8, 74.9, 61.6, 48.0, 42.5, 42.0, 39.1, 36.7, 28.5, 28.2, 24.3, 23.2.

HRMS (ESI^+) $[M+Na]^+$ Calcd for $C_{23}H_{30}N_2O_5Na$: 437.2047 m/z, Found: 437.2049 m/z;

Specific rotation: $[\alpha]_D^{28.7}$ -37.6 (*c* 0.20, $CHCl_3$)

tert-butyl ((1*R*,2*S*)-1- ((1*R*,5*S*,6*R*)-3-benzyl-2,4-dioxo-3-azabicyclo[3.2.0]heptan-6-yl)-1-hydroxy-3-methylbutan-2-yl)carbamate (3af)

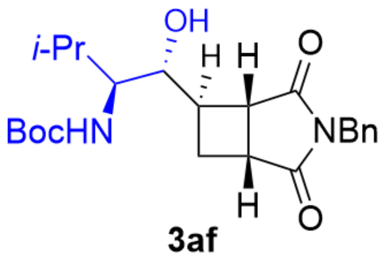

3af

yellow oil; **IR (neat):** 3419.95 (w), 3381.17 (w), 2961.88 (w), 2936.78 (w), 1702.38 (w), 1660.23 (s), 1534.57 (s), 1454.63 (m), 1433.02 (w), 1387.69 (w), 1366.89 (m), 1350.72 (m), 1334.62 (m), 1310.21 (m), 1293.53 (w), 1245.19 (w), 1161.53 (w), 1126.65 (s), 1077.66 (m), 1045.13 (w), 1023.12 (m), 942.87 (m), 773.50 (w), 735.74 (w), 697.62 (w), 641.40 (w), 622.27 (m), 573.04 (m), 465.81 (w), 429.66 (w) cm^{-1};
^{1}H NMR (400 MHz, $CDCl_3$) δ 7.31-7.28 (m, 2 H), 7.26-7.17 (m, 3 H), 4.85 (d, J = 8.8 Hz, 1 H), 4.60 (s, 2 H), 3.82-3.80 (m, 1H), 3.13-3.03 (m, 3 H), 2.97-2.92 (m, 1 H), 2.70-2.63 (m, 1 H), 2.45-2.41 (m, 1 H), 2.08-2.02 (m, 1 H), 1.94-1.87 (m, 1 H), 1.33 (s, 9 H), 0.88 (d, J = 6.8 Hz, 3 H), 0.82 (d, J = 6.8 Hz, 3 H). **^{13}C NMR (100 MHz, $CDCl_3$)** δ 179.6, 179.3, 157.1, 136.0, 128.8, 128.6, 128.0, 79.9, 72.2, 59.9, 42.5, 41.7, 41.7, 36.3, 29.0, 28.4, 23.5, 20.1, 18.9.
HRMS (ESI$^+$) [M+Na]$^+$ Calcd for $C_{23}H_{32}N_2O_5Na$: 439.2203 m/z, Found: 439.2198 m/z;
Specific rotation: $[\alpha]_D^{28.7}$-68.1 (c 0.20, $CHCl_3$)

tert-butyl ((1*R*,2*S*)-1- ((1*R*,5*S*,6*R*)-3-benzyl-2,4-dioxo-3-azabicyclo[3.2.0]heptan-6-yl)-1-hydroxy-3,3-dimethylbutan-2-yl)carbamate (3ag)

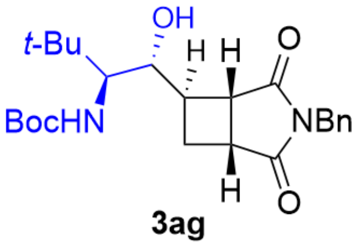

3ag

pale yellow oil; **IR (neat):** 3443.89 (br), 2962.95 (w), 1769.11 (w), 1700.15 (s), 1497.94 (m), 1453.81 (w), 1431.34 (w), 1391.56 (w), 1366.09 (w), 1341.67 (w), 1312.50 (w), 1240.66 (w), 1165.65 (m), 1057.18 (w), 699.07 (w) cm^{-1};
^{1}H NMR (400 MHz, $CDCl_3$) δ 7.35-7.22 (m, 5 H), 5.00 (d, *J* = 9.6 Hz, 1 H), 4.64 (s, 2 H), 4.07-4.02 (m, 1 H), 3.22 (d, *J* = 9.6 Hz, 1 H), 3.15-3.05 (m, 2 H), 2.78 (br, 1 H), 2.74-2.66 (m, 1 H), 2.48-2.45 (m, 1 H), 2.11-2.04 (m, 1 H), 1.39 (m, 9 H), 0.92 (m, 9 H). **^{13}C NMR (100 MHz, $CDCl_3$)** δ 179.6, 179.1, 156.1, 136.0, 128.8, 128.7, 128.0, 79.5, 69.5, 60.3, 43.4, 42.6, 41.7, 36.2, 35.5, 28.5, 27.1, 22.9.
HRMS (ESI$^+$) [M+Na]$^+$ Calcd for $C_{24}H_{34}N_2O_5Na$: 453.2360 m/z, Found: 453.2365 m/z;
Specific rotation: $[\alpha]_D^{28.7}$-77.6 (*c* 0.20, $CHCl_3$)

(1*R*,5*S*,6*R*)-6-((*R*)-hydroxy(phenyl)methyl)-3-(4-methoxyphenyl)-3-azabicyclo[3.2.0]heptane-2,4-dione (5a)

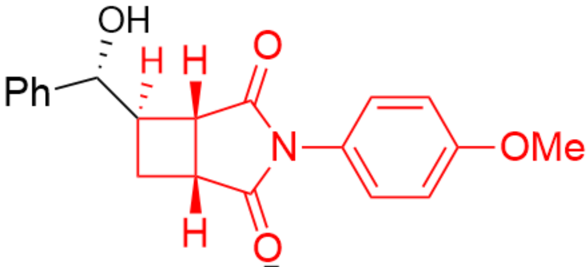

5a

pale yellow oil; **IR (neat):** 3463.91 (br), 2931.40 (w), 2152.35 (w), 1770.47 (w), 1703.23 (s), 1609.61 (w), 1512.37 (m), 1452.42 (w), 1380.19 (w), 1298.08 (w), 1249.84 (m), 1170.22 (m), 1109.27 (w), 1074.35 (w), 1028.56 (w), 828.77 (w), 803.62 (w), 759.36 (w), 740.87 (w), 701.37 (w), 674.65 (w), 611.62 (w), 588.43 (w), 524.88 (w), 432.17 (w) cm^{-1};
^{1}H NMR (400 MHz, $CDCl_3$) δ 7.36-7.28 (m, 5 H), 7.21-7.17 (m, 2 H), 6.98-6.94 (m, 2 H), 4.74 (d, J = 7.2 Hz, 1 H), 3.81 (s, 3 H), 3.54 (dd, J = 7.2, 3.2 Hz, 1 H), 3.33-3.27 (m, 1 H), 2.98 (br, 1 H), 2.88-2.83 (m, 1 H), 2.52-2.45 (m, 1 H), 2.24-2.17 (m, 1 H).
^{13}C NMR (100 MHz, $CDCl_3$) δ 179.2, 178.4, 159.6, 141.3, 128.8, 128.4, 127.7, 126.5, 124.8, 114.6, 76.3, 55.6, 44.1, 41.6, 35.7, 25.8.
HRMS (ESI$^+$) [M+Na]$^+$ Calcd for $C_{20}H_{19}NO_4Na$: 360.1206 m/z, Found: 360.1208 m/z;
Specific rotation: $[\alpha]_D^{29.5}$7.7 (c 0.50, $CHCl_3$) for an enantiomerically enriched sample of >99.5:0.5 e.r.
Enantiomeric purity of **5a** was determined by HPLC analysis in comparison with authentic racemic material (>99.5:0.5 e.r. shown; Chiralpak IF column, 80:20 hexane / ^{i}PrOH, 1.0 mL/min, 220 nm).

<Sample Information>

Sample Name : cyl-09106-1
Sample ID :
Data Filename : cyl-09106-1NEW2-IF-80-20-1.0.lcd
Method Filename : cyl-3hao-80-20-1.0ml-120min.lcm
Batch Filename : WWLL.lcb
Vial # : 1-102 Sample Type : Unknown
Injection Volume : 10 uL
Date Acquired : 9/13/2024 10:12:41 Acquired by : System Administrator
Date Processed : 9/13/2024 12:01:57 Processed by : System Administrator

<Chromatogram>

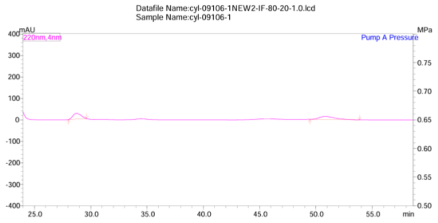

<Peak Table>

PDA Ch2 220nm

Peak#	Ret. Time	Area	Area%
1	28.730	1360449	50.098
2	50.810	1355106	49.902
Total		2715556	100.000

<Sample Information>

Sample Name : cyl-09100
Sample ID :
Data Filename : cyl-09100NEW3-IF-80-20-1.0.lcd
Method Filename : cyl-3hao-80-20-1.0ml-80min.lcm
Batch Filename : WWLL.lcb
Vial # : 1-101 Sample Type : Unknown
Injection Volume : 5 uL
Date Acquired : 9/13/2024 12:11:27 Acquired by : System Administrator
Date Processed : 9/13/2024 13:51:53 Processed by : System Administrator

<Chromatogram>

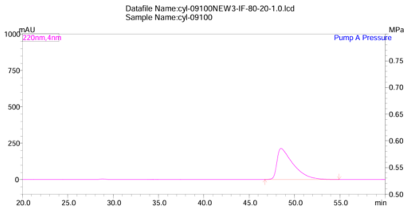

<Peak Table>

PDA Ch2 220nm

Peak#	Ret. Time	Area	Area%
1	48.510	26272436	100.000
Total		26272436	100.000

(1*R*,5*S*,6*R*)-6-((*R*)-hydroxy(phenyl)methyl)-3-(4-(trifluoromethyl)phenyl)-3-azabicyclo[3.2.0]heptane-2,4-dione (5b)

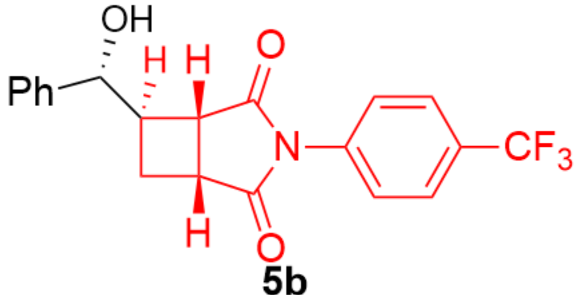

5b

yellow oil; **IR (neat):** 3468.48 (br), 1775.39 (w), 1705.10 (s), 1615.43 (w), 1519.13 (w), 1452.06 (w), 1416.68 (w), 1374.40 (m), 1324.27 (s), 1166.46 (m), 1124.37 (m), 1067.47 (m), 1021.66 (m), 1001.55 (m), 953.31 (w), 838.08 (w), 760.53 (w), 702.35 (w), 660.09 (w), 613.46 (w), 596.87 (w) cm^{-1};

^{1}H NMR (400 MHz, $CDCl_3$) δ 7.72 (d, *J* = 7.6 Hz, 2 H), 7.47 (d, *J* = 7.6 Hz, 2 H), 7.39-7.25 (m, 5 H), 4.76 (d, *J* = 8.4 Hz, 1 H), 3.59 (dd, *J* = 7.2, 4.8 Hz, 1 H), 3.38-3.32 (m, 1 H), 2.92-2.85 (m, 1 H), 2.58-2.50 (m, 1 H), 2.27-2.21 (m, 1 H). **^{13}C NMR (125 MHz, $CDCl_3$)** δ 178.4, 177.6, 141.1, 135.3, 130.5 (t, *J* = 32.5 Hz), 128.9, 128.5, 126.7, 126.4, 126.3 (t, *J* = 3.8 Hz), 123.8 (t, *J* = 271.3 Hz), 76.1, 44.2, 41.5, 35.8, 25.8. **^{19}F NMR (376 MHz, $CDCl_3$)** δ -62.72.

HRMS (FI^+) $[M]^+$ Calcd for $C_{20}H_{16}O_3NF_3$: 375.1077 m/z, Found: 375.1080 m/z;

Specific rotation: $[\alpha]_D^{28.5}$2.4 (*c* 0.50, $CHCl_3$) for an enantiomerically enriched sample of >99.5:0.5 e.r.

Enantiomeric purity of was determined by HPLC analysis in comparison with authentic racemic material (>99.5:0.5 e.r. shown; Chiralpak IA column, 90:10 hexane / ^{i}PrOH, 1.0 mL/min, 254 nm).

<Sample Information>

Sample Name : cyl-09106-2
Sample ID :
Data Filename : cyl-09106-2NEW-IA-90-10-1.0.lcd
Method Filename : cyl-4hao-90-10-1.0ml-80min.lcm
Batch Filename : WWLL.lcb
Vial # : 1-62
Injection Volume : 10 uL
Date Acquired : 9/10/2024 6:25:02
Date Processed : 9/10/2024 7:45:05
Sample Type : Unknown
Acquired by : System Administrator
Processed by : System Administrator

<Chromatogram>

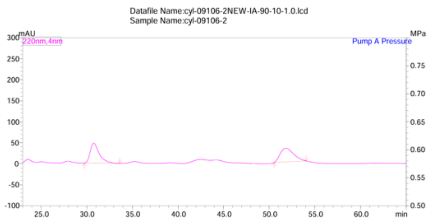

<Peak Table>

PDA Ch2 220nm

Peak#	Ret. Time	Area	Area%
1	30.785	3283535	49.548
2	51.813	3343434	50.452

<Sample Information>

Sample Name : cyl-09101
Sample ID :
Data Filename : cyl-09101NEW-IA-90-10-1.0.lcd
Method Filename : cyl-4hao-90-10-1.0ml-80min.lcm
Batch Filename : WWLL.lcb
Vial # : 1-61
Injection Volume : 10 uL
Date Acquired : 9/10/2024 5:04:30
Date Processed : 9/10/2024 10:39:22
Sample Type : Unknown
Acquired by : System Administrator
Processed by : System Administrator

<Chromatogram>

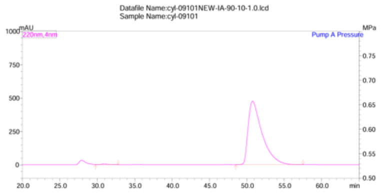

<Peak Table>

PDA Ch2 220nm

Peak#	Ret. Time	Area	Area%
1	30.925	298509	0.478
2	50.758	62202476	99.522
Total		62500985	100.000

Dimethyl-(1*R*,2*S*,3*R*)-3-((*R*)-hydroxy(phenyl)methyl)cyclobutane-1,2-dicarboxylate (5c)

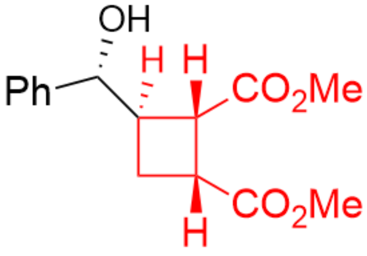

5c

pale yellow oil; **IR (neat):** 3466.05 (br), 2949.62 (w), 2923.48 (w), 1776.20 (w), 1732.87 (s), 1598.58 (w), 1492.26 (m), 1437.94 (w), 1375.56 (w), 1351.55 (w), 1326.79 (w), 1284.99 (w), 1262.63 (w), 1236.04 (w), 1203.15 (m), 1186.27 (m), 1165.33 (m), 1116.61 (m), 1073.14 (m), 1044.29 (m), 1024.24 (m), 1000.01 (m), 953.91 (m), 934.79 (w), 908.57 (m), 873.60 (w), 846.36 (m), 814.00 (m), 768.17 (s), 733.44 (w), 698.25 (w), 677.98 (w), 630.52 (w), 592.00 (w), 549.21 (w), 488.22 (w) cm^{-1};
^{1}H NMR (500 MHz, $CDCl_3$) δ 7.29-7.18 (m, 5 H), 4.51 (d, *J* = 8.8 Hz, 1 H), 3.60 (s, 3 H), 3.60 (s, 3 H), 3.31 (dd, *J* = 9.2, 8.8 Hz, 1 H), 3.25-3.20 (m, 1 H), 3.15-3.06 (m, 1 H), 2.94 (br, 1 H), 2.00-1.89 (m, 2 H). **^{13}C NMR (100 MHz, $CDCl_3$)** δ 174.0, 173.5, 141.8, 128.6, 128.0, 126.3, 77.8, 52.3, 52.1, 44.0, 43.9, 37.8, 24.6. **HRMS (ESI^+) $[M+Na]^+$** Calcd for $C_{15}H_{18}O_5Na$: 301.1046 m/z, Found: 301.1053 m/z;
Specific rotation: $[\alpha]_D^{29.4}$2.3 (*c* 0.50, $CHCl_3$) for an enantiomerically enriched sample of >99.5:0.5 e.r.
Enantiomeric purity of **5c** was determined by HPLC analysis in comparison with authentic racemic material (>99.5:0.5 e.r. shown; Chiralpak ID column, 90:10 hexane / ^{i}PrOH, 1.0 mL/min, 254 nm).

<Sample Information>

Sample Name : CYL-09106-4
Sample ID :
Data Filename : CYL-09106-4RACXX-ID-90-10-1.0.lcd
Method Filename : ZJW-2-9010-1.0-80-1min.lcm
Batch Filename : ZJW.lcb
Vial # : 1-101 Sample Type : Unknown
Injection Volume : 20 uL
Date Acquired : 5/15/2024 2:59:10 PM Acquired by : System Administrator
Date Processed : 5/15/2024 3:47:11 PM Processed by : System Administrator

<Chromatogram>

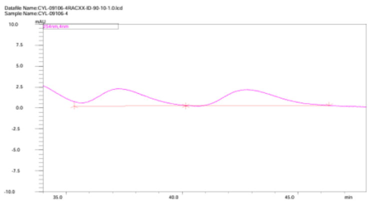

<Peak Table>

PDA Ch1 254nm

Peak#	Ret. Time	Area	Area%
1	37.218	294722	50.336
2	42.809	290789	49.664
Total		585511	100.000

<Sample Information>

Sample Name : cyl-09103
Sample ID :
Data Filename : cyl-09103NEW-ID-90-10-1.0.lcd
Method Filename : cyl-2hao-90-10-1.0ml-80min.lcm
Batch Filename : WWLL.lcb
Vial # : 1-75 Sample Type : Unknown
Injection Volume : 10 uL
Date Acquired : 9/10/2024 14:37:33 Acquired by : System Administrator
Date Processed : 9/10/2024 15:29:18 Processed by : System Administrator

<Chromatogram>

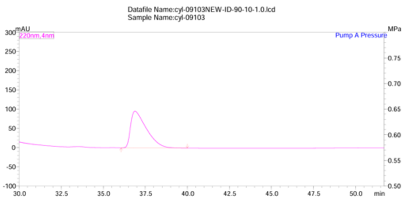

<Peak Table>

PDA Ch2 220nm

Peak#	Ret. Time	Area	Area%
1	36.888	6263041	100.000
Total		6263041	100.000

benzyl (1*R*,5*R*,6*R*)-6- ((*R*)-hydroxy (phenyl)methyl)-3-azabicyclo[3.2.0]heptane-3-carboxylate (5d)

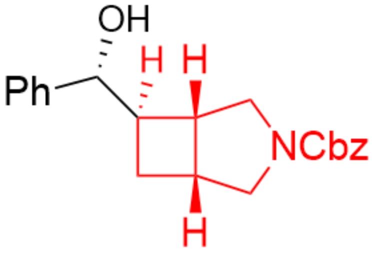

5d

pale yellow oil; **IR (neat):** 2958.98 (w), 2929.81 (w), 2862.87 (w), 1677.22 (s), 1494.75 (w), 1450.67 (m), 1416.25 (s), 1357.18 (m), 1227.75 (m), 1147.56 (m), 1097.23 (m), 1055.79 (m), 1026.91 (w), 740.35 (m), 697.43 (s), 599.20 (w), 543.35 (w) cm^{-1};
^{1}H NMR (400 MHz, $CDCl_3$) δ 7.27-7.15 (m, 10 H), 5.04 (s, 2 H), 4.49 (d, *J* = 8.0 Hz, 1H), 3.52-3.47 (m, 2 H), 3.30-3.25 (m, 1 H), 3.17 (dd, *J* = 11.2, 6.4 Hz, 1H), 2.88 (dd, *J* = 14.4, 4.0 Hz, 1H), 2.74-2.70 (m, 1 H),2.25 (br, 2 H), 1.88-1.85 (m, 1 H), 1.54-1.48 (m, 1 H). **^{13}C NMR (100 MHz, $CDCl_3$)** δ 155.5, 142.7, 137.1, 128.5, 128.0, 127.9, 127.8, 126.3, 66.8, 52.8 (m), 44.8, 41.3 (m), 40.1 (m), 34.3 (m), 33.3 (m), 27.2.
HRMS (ESI^+) $[M+Na]^+$ Calcd for $C_{21}H_{23}NO_3Na$: 360.1570 m/z, Found: 360.1563 m/z;
Specific rotation: $[\alpha]_D^{26.8}$42.1 (*c* 0.50, $CHCl_3$) for an enantiomerically enriched sample of 95:5 e.r.
Enantiomeric purity of **5d** was determined by HPLC analysis in comparison with authentic racemic material (95:5 e.r. shown; Chiralpak ID column, 94:6 hexane / ^{i}PrOH, 1.5mL/min, 220 nm).

<Sample Information>

Sample Name : CYL-09106-3
Sample ID :
Data Filename : CYL-09106-3RAC-ID-94-6-1.5.lcd
Method Filename : ZJW-2-9406-1.0-120-1.5min.lcm
Batch Filename : ZJW.lcb
Vial # : 1-100
Injection Volume : 20 uL
Date Acquired : 5/15/2024 8:11:16 PM
Date Processed : 5/16/2024 8:35:27 AM
Sample Type : Unknown
Acquired by : System Administrator
Processed by : System Administrator

<Chromatogram>

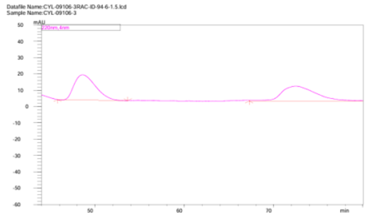

<Peak Table>

PDA Ch2 220nm

Peak#	Ret. Time	Area	Area%
1	48.566	2537720	50.307
2	72.469	2506748	49.693
Total		5044468	100.000

<Sample Information>

Sample Name : cyl-10024
Sample ID :
Data Filename : cyl-10024NEW-ID-94-6-1.5.lcd
Method Filename : cyl-2hao-94-6-1.5ml-120min.lcm
Batch Filename : WWLL.lcb
Vial # : 1-80
Injection Volume : 10 uL
Date Acquired : 9/10/2024 20:24:18
Date Processed : 9/10/2024 21:53:58
Sample Type : Unknown
Acquired by : System Administrator
Processed by : System Administrator

<Chromatogram>

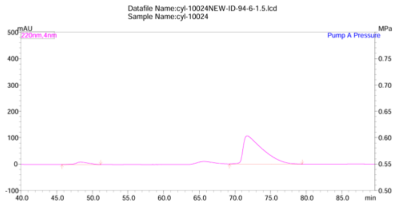

<Peak Table>

PDA Ch2 220nm

Peak#	Ret. Time	Area	Area%
1	48.352	1099532	5.161
2	71.730	20205741	94.839
Total		21305273	100.000

((1*R*,2*R*,3*R*)-3-((*R*)-hydroxy (phenyl)methyl)cyclobutane-1,2-diyl)bis (methylene) diacetate (5e)

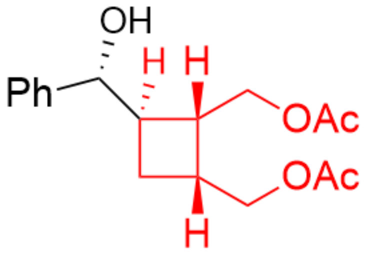

5e

yellow oil; **IR (neat):** 3505.78 (br), 2937.27 (w), 1735.49 (s), 1493.27 (w), 1452.32 (w), 1388.60 (w), 1366.48 (m), 1232.31 (s), 1028.66 (s), 974.85 (w), 803.17 (w), 760.63 (w), 702.29 (m), 631.26 (w), 605.34 (w), 538.89 (w) cm^{-1};
^{1}H NMR (400 MHz, $CDCl_3$) δ 7.28-7.17 (m, 5 H), 4.51 (d, J = 8.4 Hz, 1 H), 4.15-3.99 (m, 4 H), 2.72-2.64 (m, 1 H), 2.62-2.53 (m, 2 H), 2.44-2.36 (m, 1 H), 1.97 (s, 3 H), 1.95 (s, 3 H), 1.86-1.79 (m, 1 H), 1.51-1.45 (m, 1 H). **^{13}C NMR (100 MHz, $CDCl_3$)** δ 171.2, 171.0, 142.4, 128.6, 127.9, 126.2, 78.2, 64.8, 64.7, 43.7, 38.9, 31.5, 24.0, 21.1.
HRMS (ESI$^+$) [M+Na]$^+$ Calcd for $C_{17}H_{22}O_5Na$: 329.1359 m/z, Found: 329.1364 m/z;
Specific rotation: $[\alpha]_D^{29.6}$20.8 (c 0.50, $CHCl_3$) for an enantiomerically enriched sample of 99:1 e.r.
Enantiomeric purity of **5e** was determined by HPLC analysis in comparison with authentic racemic material (99:1 e.r. shown; Chiralpak IG column, 90:10 hexane / ^{i}PrOH, 1.0 mL/min, 220 nm).

<Sample Information>

Sample Name : CYL-10059-1cru
Sample ID :
Data Filename : CYL-10059-1rac-ig-90-10-1.0.lcd
Method Filename : ZJW-1-9010-1.0-150min.lcm
Batch Filename : ZJW.lcb
Vial # : 1-91
Sample Type : Unknown
Injection Volume : 20 uL
Date Acquired : 5/31/2024 2:39:10 PM
Acquired by : System Administrator
Date Processed : 5/31/2024 7:20:40 PM
Processed by : System Administrator

<Chromatogram>

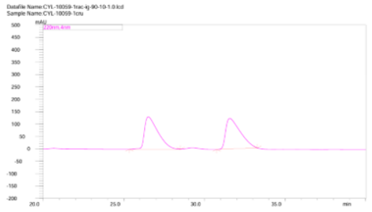

<Peak Table>

PDA Ch2 220nm

Peak#	Ret. Time	Area	Area%
1	26.515	7612575	49.622
2	31.571	7728441	50.378
Total		15341016	100.000

<Sample Information>

Sample Name : CYL-10044
Sample ID :
Data Filename : CYL-10044-IG-90-10-1.0.lcd
Method Filename : ZJW-1-9010-1.0-50-1min.lcm
Batch Filename : ZJW.lcb
Vial # : 1-92
Sample Type : Unknown
Injection Volume : 20 uL
Date Acquired : 5/31/2024 6:36:41 PM
Acquired by : System Administrator
Date Processed : 8/12/2024 11:44:02 AM
Processed by : System Administrator

<Chromatogram>

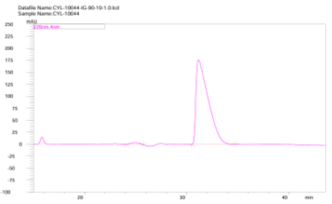

<Peak Table>

PDA Ch2 220nm

Peak#	Ret. Time	Area	Area%
1	24.961	214110	1.433
2	31.152	14722610	98.567
Total		14936720	100.000

(*R*)-6-((*R*)-((1*R*,5*S*,6*R*)-3-benzyl-2,4-dioxo-3-azabicyclo[3.2.0]heptan-6-yl)(hydroxy)methyl)-[1,1':2',1''-terphenyl]-2-carbaldehyde (7a)

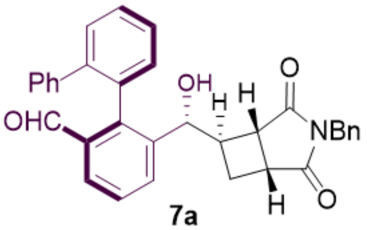

7a

pale yellow solid, m.p.161-162 °C; **IR (neat):** 3649.62 (w), 3615.90 (w), 3583.40 (w), 3564.41 (w), 3536.76 (w), 3503.14 (w), 3467.47 (w), 3423.74 (w), 3401.91 (w), 3133.31 (w), 3084.66 (w), 3065.57 (w), 2153.91 (w), 1699.37 (s), 1430.27 (m), 1390.88 (w), 1342.65 (w), 1311.66 (w), 1238.39 (w), 1160.52 (m), 1124.86 (w), 998.54 (w), 937.77 (w), 749.18 (m), 701.60 (m) cm^{-1}

^{1}H NMR (400 MHz, $CDCl_3$) δ 9.89 (s, 1 H), 7.96-7.94 (m, 1 H), 7.59-7.54 (m, 3 H), 7.47-7.41 (m, 2 H), 7.34-7.25 (m, 5 H), 7.21-7.16 (m, 4 H), 7.06-7.03 (m, 2 H), 4.60 (s, 2 H), 4.32 (d, *J* = 7.2 Hz, 1 H), 3.23 (dd, *J* = 7.2, 4.0 Hz, 1 H), 2.96-2.91 (m, 1 H), 2.39-2.33 (m, 1 H), 2.06-1.99 (m, 1 H), 1.84-1.77 (m, 1 H). **^{13}C NMR (100 MHz, $CDCl_3$)** δ 192.4, 179.2, 178.2, 142.6, 141.2, 140.5, 140.1, 136.0, 135.0, 133.5, 132.3, 130.4, 129.5, 129.3, 128.8, 128.7, 128.5, 128.0, 127.8, 127.7, 127.5, 71.1, 43.2, 42.5, 41.2, 35.5, 25.0.
HRMS (ESI$^+$) [M+Na]$^+$ Calcd for $C_{33}H_{27}NO_4Na$: 524.1832 m/z, Found: 524.1831 m/z;
Specific rotation: $[\alpha]_D^{29.5}$26.2 (*c* 0.50, $CHCl_3$) for an enantiomerically enriched sample of >99.5:0.5 e.r.
Enantiomeric purity of **7a** was determined by HPLC analysis in comparison with authentic racemic material (>99.5:0.5 e.r. shown; Chiralpak IB N-5 column, 80:20 hexane / ^{i}PrOH, 1.0 mL/min, 254 nm).

<Sample Information>

Sample Name : CYL-09081-17
Sample ID :
Data Filename : CYL-09081-17RAC-IBN-5-80-20-1.0.lcd
Method Filename : ZJW-4-8020-1.0-60-1min.lcm
Batch Filename : ZJW.lcb
Vial # : 1-97 Sample Type : Unknown
Injection Volume : 20 uL
Date Acquired : 5/7/2024 1:12:34 PM Acquired by : System Administrator
Date Processed : 7/25/2024 3:49:42 PM Processed by : System Administrator

<Chromatogram>

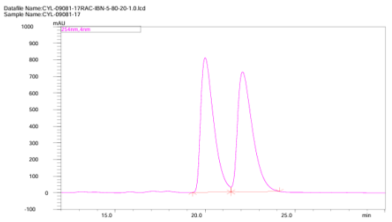

<Peak Table>

PDA Ch1 254nm

Peak#	Ret. Time	Area	Area%
1	19.996	40892947	49.545
2	22.078	41643900	50.455
Total		82536846	100.000

<Sample Information>

Sample Name : CYL-09098
Sample ID :
Data Filename : CYL-09098-IBN-5-80-20-1.0.lcd
Method Filename : ZJW-4-8020-1.0-60-1min.lcm
Batch Filename : ZJW.lcb
Vial # : 1-80 Sample Type : Unknown
Injection Volume : 20 uL
Date Acquired : 5/7/2024 9:56:01 AM Acquired by : System Administrator
Date Processed : 5/7/2024 2:46:54 PM Processed by : System Administrator

<Chromatogram>

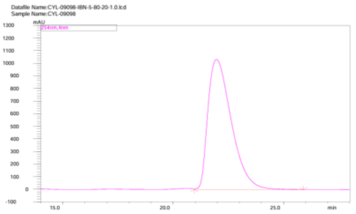

<Peak Table>

PDA Ch1 254nm

Peak#	Ret. Time	Area	Area%
1	21.956	76015557	100.000
Total		76015557	100.000

(*R*)-6-((*R*)-((1*R*,5*S*,6*R*)-3-benzyl-2,4-dioxo-3-azabicyclo[3.2.0]heptan-6-yl) (hydroxy)methyl)-2'- (naphthalen-2-yl)-[1,1'-biphenyl]-2-carbaldehyde (7b)

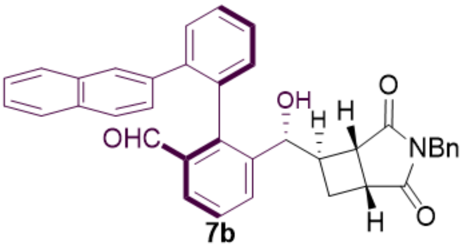

7b

colorless oil; **IR (neat):** 3470.24 (br), 3056.11 (w), 2941.94 (w), 2853.54 (w), 1767.07 (w), 1688.01 (s), 1588.36 (w), 1492.48 (w), 1429.17 (w), 1388.47 (w), 1339.96 (m), 1310.13 (w), 1291.03 (w), 1238.43 (w), 1157.01 (m), 1125.91 (w), 1068.42 (w), 1027.84 (w), 999.01 (w), 938.61 (w), 908.05 (w), 858.49 (w), 818.20 (w), 757.37 (m), 728.33 (m), 697.38 (m), 643.46 (w), 619.75 (w), 597.35 (w), 572.21 (w), 537.61 (w), 476.87 (m), 426.02 (w) cm^{-1}

^{1}H NMR (400 MHz, $CDCl_3$) δ 9.87 (s, 1 H), 7.88-7.86 (m, 1 H), 7.64-7.62 (m, 1 H), 7.57-7.43 (m, 6 H), 7.39-7.31 (m, 4 H), 7.26-7.16 (m, 5 H), 7.11 (d, *J* = 7.6 Hz, 1 H), 7.06-7.03 (m, 1 H), 4.51 (s, 2 H), 4.34 (d, *J* = 8.0 Hz, 1 H), 3.13 (dd, *J* = 6.4, 4.8 Hz, 1H), 2.89-2.83 (m, 1 H), 2.29-2.22 (m, 1 H), 2.06-1.98 (m, 1 H), 1.77-1.71 (m, 1 H), 1.01 (br, 1 H). **^{13}C NMR (100 MHz, $CDCl_3$)** δ 192.4, 179.3, 178.3, 142.6, 141.2, 140.6, 137.6, 136.0, 135.0, 133.8, 133.0, 132.2, 132.1, 129.4, 128.8, 128.5, 128.11, 128.07, 128.0, 127.8, 127.7, 127.0, 126.8, 126.7, 70.9, 43.2, 42.5, 40.9, 35.5, 25.0.

HRMS (ESI$^+$) [M+Na]$^+$ Calcd for $C_{37}H_{29}NO_4Na$: 574.1989 m/z, Found: 574.1981 m/z;

Specific rotation: $[\alpha]_D^{24.1}$19.8 (*c* 0.50, $CHCl_3$) for an enantiomerically enriched sample of >99.5:0.5 e.r.

Enantiomeric purity of **7b** was determined by HPLC analysis in comparison with authentic racemic material (>99.5:0.5 e.r. shown; ID column, 90:10 hexane / ^{i}PrOH, 1.0 mL/min, 220 nm).

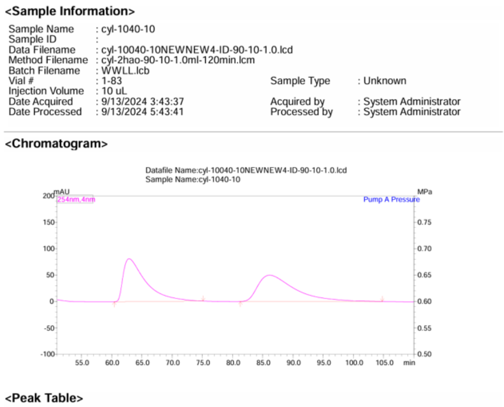

Peak#	Ret. Time	Area	Area%
1	62.882	21156640	50.034
2	86.172	21127533	49.966
Total		42284173	100.000

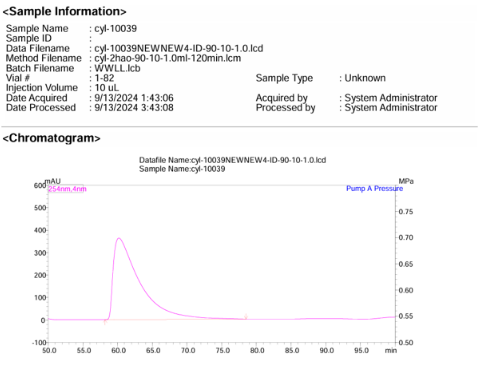

Peak#	Ret. Time	Area	Area%
1	60.179	96092843	100.000
Total		96092843	100.000

(*R*)-6-((*R*)-((1*R*,5*S*,6*R*)-3-benzyl-2,4-dioxo-3-azabicyclo[3.2.0]heptan-6-yl) (hydroxy)methyl)-2'-isopropyl-[1,1'-biphenyl]-2-carbaldehyde (7c)

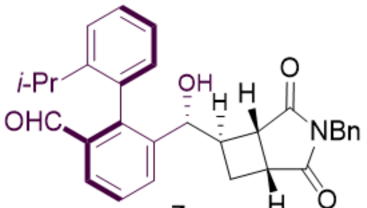

7c

colorless oil; **IR (neat):** 3485.57 (br), 3059.15 (w), 2958.81 (w), 2926.42 (w), 2853.98 (w), 1695.22 (s), 1458.87 (w), 1433.50 (w), 1389.91 (m), 1341.34 (w), 1310.87 (w), 1290.82 (w), 1240.98 (w), 1161.28 (m), 1125.20 (w), 999.77 (w), 938.74 (w), 761.96 (w), 701.08 (w), 620.88 (w) cm^{-1}

^{1}H NMR (400 MHz, $CDCl_3$) δ 9.48 (s, 1 H), 7.93-7.91 (m, 1 H), 7.81-7.79 (m, 1 H), 7.53-7.49 (m, 1 H), 7.40-7.35 (m, 2 H), 7.29-7.21 (m, 5 H), 7.15-7.11 (m, 1 H), 6.86 (d, *J* = 7.6 Hz, 1 H), 4.57 (s, 2 H), 4.42 (d, *J* = 6.0 Hz, 1 H), 3.30-3.27 (m, 1 H), 3.00-2.95 (m, 1 H), 2.54-2.45 (m, 1 H), 2.25-2.16 (m, 1 H), 1.88-1.82 (m, 1 H), 1.14 (d, *J* = 6.4 Hz, 3 H), 0.89 (d, *J* = 6.4 Hz, 3 H). **^{13}C NMR (100 MHz, $CDCl_3$)** δ 192.2, 179.3, 178.7, 147.2, 143.2, 140.8, 136.0, 134.6, 133.1, 131.7, 130.8, 129.4, 128.80, 128.78, 128.6, 128.1, 127.3, 126.2, 125.8, 43.5, 42.6, 40.6, 35.5, 30.3, 25.1, 24.5, 23.3.

HRMS (ESI$^+$) [M+Na]$^+$ Calcd for $C_{30}H_{29}NO_4Na$:490.1989 m/z, Found: 490.1987 m/z;

Specific rotation: $[\alpha]_D^{24.7}$32.2 (*c* 0.15, $CHCl_3$) for an enantiomerically enriched sample of 99.5:0.5 e.r.

Enantiomeric purity of **7c** was determined by HPLC analysis in comparison with authentic racemic material (99.5:0.5 e.r. shown; Chiralpak IC column, 80:20 hexane / ^{i}PrOH, 1.0 mL/min, 254 nm).

<Sample Information>

Sample Name : CYL-10040-7
Sample ID :
Data Filename : CYL-10040-7RAC-IC-80-20-1.0.lcd
Method Filename : ZJW-5-8020-1.0-90min.lcm
Batch Filename : ZJW.lcb
Vial # : 1-102
Injection Volume : 20 uL
Date Acquired : 5/20/2024 12:50:04 PM
Date Processed : 8/18/2024 11:10:13 PM
Sample Type : Unknown
Acquired by : System Administrator
Processed by : System Administrator

<Chromatogram>

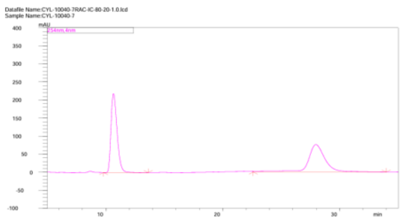

<Peak Table>

PDA Ch1 254nm

Peak#	Ret. Time	Area	Area%
1	10.659	7854012	50.327
2	27.968	7751802	49.673
Total		15605814	100.000

<Sample Information>

Sample Name : CYL-10016
Sample ID :
Data Filename : CYL-10016-IC-80-20-1.0.lcd
Method Filename : ZJW-5-8020-1.0-90min.lcm
Batch Filename : ZJW.lcb
Vial # : 1-103
Injection Volume : 20 uL
Date Acquired : 5/20/2024 2:19:03 PM
Date Processed : 5/20/2024 6:35:25 PM
Sample Type : Unknown
Acquired by : System Administrator
Processed by : System Administrator

<Chromatogram>

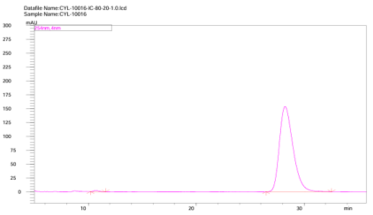

<Peak Table>

PDA Ch1 254nm

Peak#	Ret. Time	Area	Area%
1	10.680	70891	0.524
2	28.226	13445777	99.476
Total		13516668	100.000

(*R*)-6-((*R*)-((1*R*,5*S*,6*R*)-3-benzyl-2,4-dioxo-3-azabicyclo[3.2.0]heptan-6-yl) (hydroxy)methyl)-2'-vinyl-[1,1'-biphenyl]-2-carbaldehyde (7d)

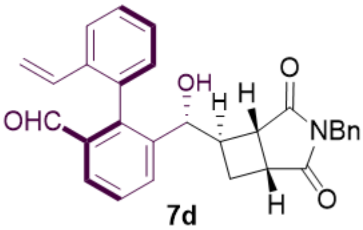

7d

colorless oil; **IR (neat):** 3486.62 (br), 2929.14 (w), 2855.23 (w), 1767.82 (w), 1693.30 (s), 1587.78 (w), 1494.92 (w), 1431.41 (w), 1390.78 (m), 1342.68 (w), 1310.89 (w), 1291.62 (w), 1241.22 (w), 1161.36 (w), 1125.50 (m), 996.42 (w), 920.71 (w), 760.71 (w), 700.93 (m), 621.87 (w) cm^{-1}

^{1}H NMR (400 MHz, $CDCl_3$) δ 9.48 (s, 1 H), 7.94-7.92 (m, 1 H), 7.83-7.81 (m, 1 H), 7.62 (d, *J* = 8.0 Hz, 1 H), 7.55-7.51 (m, 1 H), 7.40-7.36 (m, 1 H), 7.29-7.19 (m, 6 H), 6.95 (d, *J* = 7.6 Hz, 1 H), 6.24 (dd, *J* = 16.0, 11.2 Hz, 1H), 5.60 (d, *J* = 16.0 Hz, 1 H), 5.12 (d, *J* = 11.2 Hz, 1 H), 4.56 (s, 2 H), 4.37 (d, *J* = 6.0 Hz, 1 H), 3.31-3.28 (m, 1 H), 3.00-2.94 (m, 1 H), 2.48-2.42 (m, 1 H), 2.22-2.14 (m, 1 H), 1.94-1.82 (m, 1 H). **^{13}C NMR (125 MHz, $CDCl_3$)** δ 191.9, 179.3, 178.5, 142.3, 141.2, 136.6, 136.0, 134.44, 134.38, 133.7, 132.0, 131.3, 129.2, 129.0, 128.83, 128.80, 128.1, 128.0, 127.5, 125.8, 117.1, 70.8, 42.9, 42.6, 40.4, 35.7, 25.3.

HRMS (ESI$^+$) [M+Na]$^+$ Calcd for $C_{29}H_{25}NO_4Na$: 474.1676 m/z, Found: 474.1672 m/z;

Specific rotation: $[\alpha]_D^{25.4}$40.4 (*c* 0.15, $CHCl_3$) for an enantiomerically enriched sample of >99.5:0.5 e.r.

Enantiomeric purity of **7d** was determined by HPLC analysis in comparison with authentic racemic material (>99.5:0.5 e.r. shown; Chiralpak IC column, 90:10 hexane / ^{i}PrOH, 1.0 mL/min, 254 nm).

<Sample Information>

Sample Name : CYL-10040-9
Sample ID :
Data Filename : CYL-10040-9RAC-IC-90-10-1.0.lcd
Method Filename : ZJW-5-9010-1.0-100-1min.lcm
Batch Filename : ZJW.lcb
Vial # : 1-82 Sample Type : Unknown
Injection Volume : 20 uL
Date Acquired : 5/27/2024 3:54:17 PM Acquired by : System Administrator
Date Processed : 8/18/2024 11:20:18 PM Processed by : System Administrator

<Chromatogram>

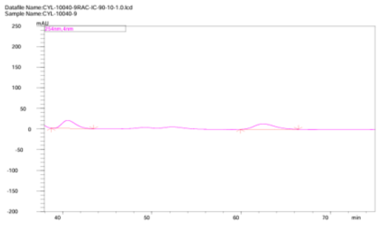

<Peak Table>

PDA Ch1 254nm

Peak#	Ret. Time	Area	Area%
1	40.566	2092593	49.615
2	62.428	2125038	50.385
Total		4217630	100.000

<Sample Information>

Sample Name : CYL-10035
Sample ID :
Data Filename : CYL-10035-IC-90-10-1.0.lcd
Method Filename : ZJW-5-9010-1.0-100-1min.lcm
Batch Filename : ZJW.lcb
Vial # : 1-83 Sample Type : Unknown
Injection Volume : 20 uL
Date Acquired : 5/27/2024 5:30:32 PM Acquired by : System Administrator
Date Processed : 8/18/2024 11:24:18 PM Processed by : System Administrator

<Chromatogram>

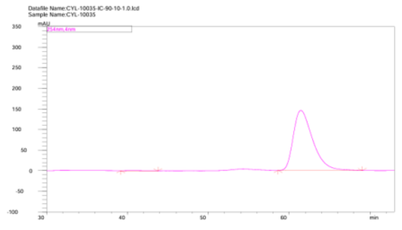

<Peak Table>

PDA Ch1 254nm

Peak#	Ret. Time	Area	Area%
1	40.652	57126	0.235
2	61.429	24269189	99.765
Total		24326315	100.000

(*R*)-6-((*R*)-((1*R*,5*S*,6*R*)-3-benzyl-2,4-dioxo-3-azabicyclo[3.2.0]heptan-6-yl) (hydroxy)methyl)-2'-methyl-[1,1'-biphenyl]-2-carbaldehyde (7e)

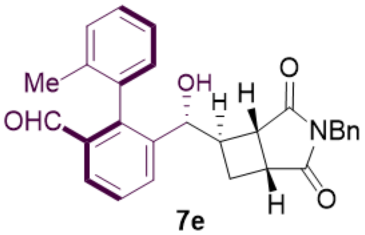

7e

yellow oil; **IR (neat):** 3463.26 (br), 2928.84 (w), 2855.02 (w), 1768.10 (w), 1691.82 (s), 1588.50 (w), 1493.92 (w), 1453.58 (w), 1431.68 (w), 1390.83 (m), 1342.05 (w), 1311.26 (w), 1291.90 (w), 1241.79 (w), 1161.47 (w), 1124.33 (m), 1000.88 (w), 937.84 (w), 757.99 (w), 737.04 (w), 701.44 (w), 622.83 (w) cm^{-1}
^{1}H NMR (400 MHz, $CDCl_3$) δ 9.45 (s, 1 H), 7.92-7.90 (m, 1 H), 7.81-7.78 (m, 1 H), 7.52-7.48 (m, 1 H), 7.29-7.11 (m, 8 H), 6.91 (d, *J* = 7.6 Hz, 1 H), 4.56 (s, 2 H), 4.38 (d, *J* = 6.0 Hz, 1 H), 3.29-3.27 (m, 1 H), 2.99-2.95 (m, 1 H), 2.53-2.47 (m, 1 H), 2.26-2.18 (m, 1 H), 1.96 (s, 3 H), 1.89-1.84 (m, 1 H), 1.62 (br, 1 H). **^{13}C NMR (100 MHz, $CDCl_3$)** δ 192.2, 179.3, 178.7, 143.3, 140.6, 136.4, 136.0, 134.7, 134.2, 131.9, 130.7, 130.6, 128.9, 128.8, 128.7, 128.1, 127.4, 126.0, 70.9, 43.5, 42.6, 40.4, 35.6, 29.8, 25.2, 20.4, 20.3.
HRMS (ESI$^+$) [M+Na]$^+$ Calcd for $C_{28}H_{25}NO_4Na$: 462.1676 m/z, Found: 462.1675 m/z;
Specific rotation: $[\alpha]_D^{28.6}$ −74.8 (*c* 0.20, $CHCl_3$) for an enantiomerically enriched sample of >99.5:0.5 e.r.
Enantiomeric purity of **7e** was determined by HPLC analysis in comparison with authentic racemic material (>99.5:0.5 e.r. shown; Chiralpak IB N-5 column, 80:20 hexane / ^{i}PrOH, 1.0 mL/min, 220 nm).

<Sample Information>

Sample Name : CYL-10040-3
Sample ID :
Data Filename : CYL-10040-3RAC-IBN-5-80-20-1.0.lcd
Method Filename : ZJW-4-8020-1.0-60-1min.lcm
Batch Filename : ZJW.lcb
Vial # : 1-96
Sample Type : Unknown
Injection Volume : 20 uL
Date Acquired : 5/19/2024 3:58:25 PM
Acquired by : System Administrator
Date Processed : 5/20/2024 8:36:24 AM
Processed by : System Administrator

<Chromatogram>

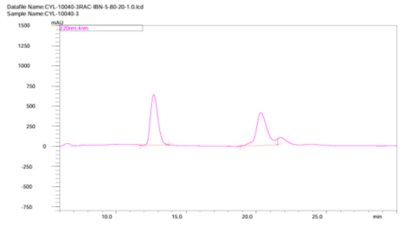

<Peak Table>

PDA Ch2 220nm

Peak#	Ret. Time	Area	Area%
1	12.690	22677965	50.324
2	20.321	22386324	49.676
Total		45064288	100.000

<Sample Information>

Sample Name : CYL-10007
Sample ID :
Data Filename : CYL-10007-IBN-5-80-20-1.0.lcd
Method Filename : ZJW-4-8020-1.0-60-1min.lcm
Batch Filename : ZJW.lcb
Vial # : 1-97
Sample Type : Unknown
Injection Volume : 20 uL
Date Acquired : 5/19/2024 4:58:59 PM
Acquired by : System Administrator
Date Processed : 5/19/2024 5:59:02 PM
Processed by : System Administrator

<Chromatogram>

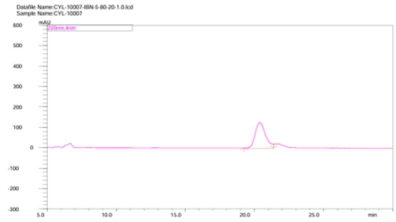

<Peak Table>

PDA Ch2 220nm

Peak#	Ret. Time	Area	Area%
1	20.374	6490841	100.000
Total		6490841	100.000

(*R*)-6-((*R*)-((1*R*,5*S*,6*R*)-3-benzyl-2,4-dioxo-3-azabicyclo[3.2.0]heptan-6-yl) (hydroxy)methyl)-2'-chloro-[1,1'-biphenyl]-2-carbaldehyde (7f)

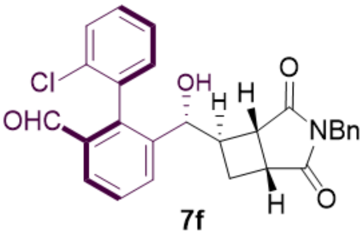

7f

colorless oil; **IR (neat):** 3464.86 (br), 1767.62 (w), 1690.04 (s), 1586.73 (w), 1494.25 (w), 1429.81 (w), 1389.77 (m), 1339.39 (w), 1310.49 (w), 1290.84 (w), 1241.67 (w), 1157.28 (m), 1125.72 (w), 1063.28 (w), 1032.69 (w), 1001.10 (w), 936.69 (w), 886.08 (w), 810.89 (m), 756.03 (m), 690.60 (m), 640.10 (w), 618.83 (w), 596.59 (w), 561.04 (w), 471.49 (w), 435.00 (w) cm^{-1}

^{1}H NMR (400 MHz, CDCl$_3$) δ 9.52 (s, 1 H), 7.99-7.93 (m, 2 H), 7.63-7.59 (m, 1 H), 7.54-7.50 (m, 1 H), 7.43-7.26 (m, 7 H), 7.09-7.07 (m, 1 H), 4.61 (s, 2 H), 4.51 (d, *J* = 5.6 Hz, 1 H), 3.41 (dd, *J* =6.8, 4.4 Hz, 1 H), 3.08-3.02 (m, 1 H), 2.49-2.44 (m, 1 H), 2.35-2.28 (m, 1 H), 1.94-1.88 (m, 1 H), 1.89-1.84 (m, 1 H), 1.62 (br, 1 H). **^{13}C NMR (100 MHz, CDCl$_3$)** δ 179.4, 179.3, 178.5, 141.1, 140.3, 136.0, 134.2, 134.1, 133.5, 132.4, 132.1, 130.3, 130.1, 129.4, 128.79, 128.76, 128.0, 127.9, 127.1, 70.7, 42.9, 42.6, 40.1, 35.7, 25.2.

HRMS (ESI$^+$) [M+Na]$^+$ Calcd for $C_{27}H_{22}NO_4NaCl$: 482.1130 m/z, Found: 482.1127 m/z;

Specific rotation: $[\alpha]_D^{23.7}$128.1 (*c* 0.50, $CHCl_3$) for an enantiomerically enriched sample of >99.5:0.5 e.r.

Enantiomeric purity of **7f** was determined by HPLC analysis in comparison with authentic racemic material (>99.5:0.5 e.r. shown; Chiralpak IC column, 80:20 hexane / ^{i}PrOH, 1.0 mL/min, 254 nm).

<Sample Information>

Sample Name : CYL-10040-4
Sample ID :
Data Filename : CYL-10040-4RAC-IC-80-20-1.0.lcd
Method Filename : ZJW-5-8020-1.0-90min.lcm
Batch Filename : ZJW.lcb
Vial # : 1-98 Sample Type : Unknown
Injection Volume : 20 uL
Date Acquired : 5/20/2024 11:34:35 AM Acquired by : System Administrator
Date Processed : 5/20/2024 2:55:31 PM Processed by : System Administrator

<Chromatogram>

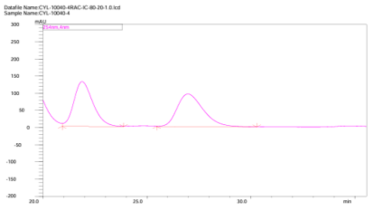

<Peak Table>

PDA Ch1 254nm

Peak#	Ret. Time	Area	Area%
1	21.879	8354128	50.157
2	26.968	8301772	49.843
Total		16655900	100.000

<Sample Information>

Sample Name : CYL-10010
Sample ID :
Data Filename : CYL-10010-IC-80-20-1.0.lcd
Method Filename : ZJW-5-8020-1.0-90min.lcm
Batch Filename : ZJW.lcb
Vial # : 1-99 Sample Type : Unknown
Injection Volume : 20 uL
Date Acquired : 5/20/2024 12:11:21 PM Acquired by : System Administrator
Date Processed : 8/18/2024 10:50:27 PM Processed by : System Administrator

<Chromatogram>

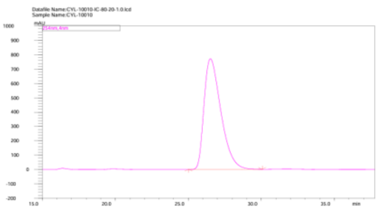

<Peak Table>

PDA Ch1 254nm

Peak#	Ret. Time	Area	Area%
1	26.512	61819553	100.000
Total		61819553	100.000

methyl(*R*)-2'- ((*R*)- ((1*R*,5*S*,6*R*)-3-benzyl-2,4-dioxo-3-azabicyclo[3.2.0]heptan-6-yl)(hydroxy)methyl)-6'-formyl-[1,1'-biphenyl]-2-carboxylate (7g)

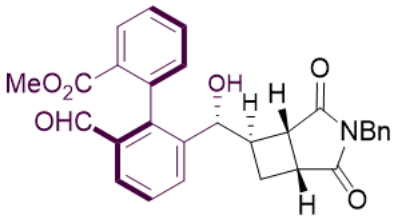

7g

colorless oil; **IR (neat):** 3454.17 (br), 2948.64 (w), 2854.57 (w), 1767.69 (w), 1689.95 (s), 1590.54 (w), 1494.08 (w), 1432.44 (w), 1390.10 (m), 1340.62 (w), 1279.33 (m), 1258.29 (m), 1191.52 (w), 1160.25 (m), 1127.95 (w), 1079.21 (w), 1046.50 (w), 999.68 (w), 960.68 (w), 910.16 (m), 802.27 (w), 723.89 (s), 643.68 (w), 619.97 (w), 597.12 (w), 570.86 (w), 470.04 (w) cm^{-1}

^{1}H NMR (400 MHz, $CDCl_3$) δ 9.45 (s, 1 H), 8.07-8.04 (m, 1 H), 7.94-7.89 (m, 2 H), 7.61-7.54 (m, 3 H), 7.39-7.37 (m, 2 H), 7.33-7.26 (m, 3 H), 7.16-7.14 (m, 1 H), 4.67 (s, 2 H), 4.44 (d, J = 7.2 Hz, 1 H), 3.69 (s, 3 H), 3.48 (dd, J =7.2, 4.0 Hz, 1 H), 3.34 (br, 1 H), 3.09-3.04 (m, 1 H), 2.70-2.67 (m, 1 H), 2.26-2.18 (m, 1 H), 2.04-1.97 (m, 1 H). **^{13}C NMR (100 MHz, $CDCl_3$)** δ 191.3, 179.4, 178.7, 168.1, 143.0, 141.1, 136.9, 136.1, 133.8, 132.1, 132.0, 131.8, 130.7, 128.9, 128.79, 128.77, 128.0, 127.8, 71.2, 52.8, 42.6, 42.1, 41.0, 35.9, 25.6.

HRMS (ESI$^+$) [M+Na]$^+$ Calcd for $C_{29}H_{25}NO_6Na$: 506.1574 m/z, Found: 506.1567 m/z; **Specific rotation:** $[\alpha]_D^{28.4}$61.3 (c 0.50, $CHCl_3$) for an enantiomerically enriched sample of >99.5:0.5 e.r.

Enantiomeric purity of **7g** was determined by HPLC analysis in comparison with authentic racemic material (>99.5:0.5 e.r. shown; Chiralpak IF column, 90:10 hexane / ^{i}PrOH, 1.0 mL/min, 254 nm).

<Sample Information>

Sample Name : cyl-10040-2
Sample ID :
Data Filename : cyl-10040-2NEW-IF-90-10-1.0.lcd
Method Filename : cyl-3hao-90-10-1.0ml-80min.lcm
Batch Filename : WWLL.lcb
Vial # : 1-64
Sample Type : Unknown
Injection Volume : 10 uL
Date Acquired : 9/10/2024 9:26:46
Acquired by : System Administrator
Date Processed : 9/10/2024 14:38:03
Processed by : System Administrator

<Chromatogram>

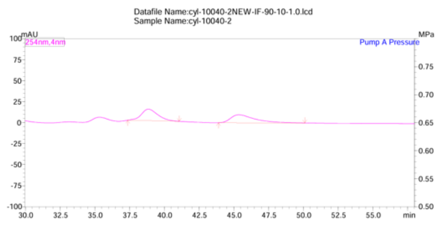

<Peak Table>

PDA Ch1 254nm

Peak#	Ret. Time	Area	Area%
1	38.862	1127503	49.454
2	45.398	1152386	50.546
Total		2279889	100.000

<Sample Information>

Sample Name : cyl-10003
Sample ID :
Data Filename : cyl-10003NEW-IF-90-10-1.0.lcd
Method Filename : cyl-3hao-90-10-1.0ml-80min.lcm
Batch Filename : WWLL.lcb
Vial # : 1-63
Sample Type : Unknown
Injection Volume : 10 uL
Date Acquired : 9/10/2024 8:06:07
Acquired by : System Administrator
Date Processed : 9/10/2024 14:38:28
Processed by : System Administrator

<Chromatogram>

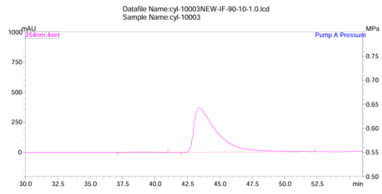

<Peak Table>

PDA Ch1 254nm

Peak#	Ret. Time	Area	Area%
1	39.052	211959	0.471
2	43.423	44813204	99.529
Total		45025163	100.000

(*R*)-6-((*R*)-((1*R*,5*S*,6*R*)-3-benzyl-2,4-dioxo-3-azabicyclo[3.2.0]heptan-6-yl) (hydroxy)methyl)-5'-methoxy-[1,1':2',1''-terphenyl]-2-carbaldehyde (7h)

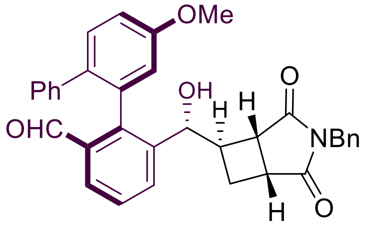

7h

colorless oil; **IR (neat):** 3452.26 (br), 2928.31 (w), 2850.98 (w), 1767.51 (w), 1687.50 (s), 1602.87 (w), 1561.35 (w), 1480.29 (w), 1459.44 (w), 1438.21 (m), 1388.94 (w), 1339.66 (w), 1309.42 (w), 1288.53 (w), 1263.17 (w), 1226.39 (m), 1206.28 (w), 1160.11 (m), 1123.25 (w), 1071.79 (w), 1049.76 (w), 1017.68 (w), 910.10 (w), 879.33 (w), 816.90 (w), 771.27 (w), 728.26 (m), 698.97 (s), 647.46 (w), 619.72 (w), 591.47 (w), 567.16 (w), 516.76 (w), 469.29 (w) cm^{-1}

^{1}H NMR (400 MHz, $CDCl_3$) δ 9.93 (s, 1 H), 7.96-7.94 (m, 1 H), 7.59-7.57 (m, 1 H), 7.49-7.43 (m, 2 H), 7.34-7.23 (m, 5 H), 7.20-7.15 (m, 3 H), 7.11-7.08 (m, 1 H), 7.01-6.99 (m, 2 H), 6.73 (d, *J* = 2.8 Hz, 1 H), 4.61 (s, 2 H), 4.36 (d, *J* = 7.6 Hz, 1 H), 3.83 (s, 3 H), 3.27 (dd, *J* = 6.8, 4.0 Hz, 1 H), 3.01-2.95 (m, 1 H), 2.43-2.36 (m, 1 H), 2.11-2.03 (m, 1 H), 1.88-1.81 (m, 1 H). **^{13}C NMR (100 MHz, $CDCl_3$)** δ 192.4, 179.3, 178.3, 158.9, 142.6, 140.5, 139.8, 136.0, 134.9, 134.7, 133.7, 132.4, 131.5, 129.5, 128.8, 128.7, 128.5, 128.0, 127.6, 127.1, 117.8, 114.5, 71.1, 55.6, 43.1, 42.5, 41.2, 35.6, 25.1.

HRMS (ESI$^+$) [M+Na]$^+$ Calcd for $C_{34}H_{29}NO_5Na$: 554.1938 m/z, Found: 554.1930 m/z; **Specific rotation:** $[\alpha]_D^{26.4}$-11.8 (*c* 0.50, $CHCl_3$) for an enantiomerically enriched sample of >99.5:0.5 e.r.

Enantiomeric purity of **7h** was determined by HPLC analysis in comparison with authentic racemic material (>99.5:0.5 e.r. shown; Chiralpak IB N-5 column, 90:10 hexane / ^{i}PrOH, 1.0 mL/min, 254 nm).

<Sample Information>

Sample Name : CYL-10040-1
Sample ID :
Data Filename : CYL-10040-1X-IBN-5-90-10-1.0.lcd
Method Filename : ZJW-6-9010-1.0-120-1min.lcm
Batch Filename : ZJW.lcb
Vial # : 1-46
Sample Type : Unknown
Injection Volume : 10 uL
Date Acquired : 8/26/2024 10:32:05 PM
Acquired by : System Administrator
Date Processed : 8/27/2024 11:50:21 AM
Processed by : System Administrator

<Chromatogram>

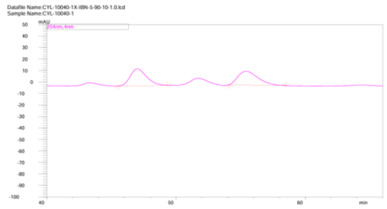

<Peak Table>

PDA Ch1 254nm

Peak#	Ret. Time	Area	Area%
1	47.008	1305400	49.930
2	55.420	1309069	50.070
Total		2614468	100.000

<Sample Information>

Sample Name : cyl-10002
Sample ID :
Data Filename : cyl-10002NEWNEW-IBN-5-90-10-1.0.lcd
Method Filename : cyl-6hao-90-10-1.0ml-90min.lcm
Batch Filename : WWLL.lcb
Vial # : 1-86
Sample Type : Unknown
Injection Volume : 10 uL
Date Acquired : 9/11/2024 12:38:44
Acquired by : System Administrator
Date Processed : 9/11/2024 15:17:02
Processed by : System Administrator

<Chromatogram>

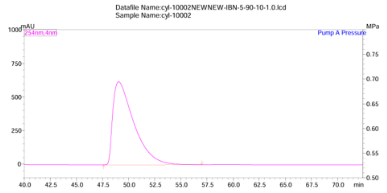

<Peak Table>

PDA Ch1 254nm

Peak#	Ret. Time	Area	Area%
1	49.029	86471778	100.000
Total		86471778	100.000

(*R*)-6-((*R*)-((1*R*,5*S*,6*R*)-3-benzyl-2,4-dioxo-3-azabicyclo[3.2.0]heptan-6-yl)(hydroxy)methyl)-5'-fluoro-[1,1':2',1''-terphenyl]-2-carbaldehyde (7i)

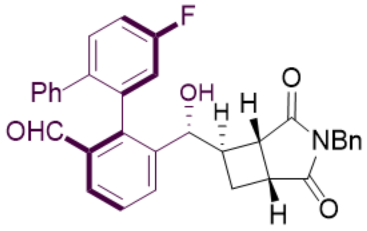

7i

colorless oil; **IR (neat):** 3455.51 (br), 1768.61 (w), 1693.72 (s), 1604.52 (w), 1584.53 (w), 1478.70 (w), 1454.82 (w), 1390.25 (m), 1342.12 (w), 1311.09 (w), 1291.39 (w), 1237.06 (w), 1164.51 (m), 1123.49 (w), 1073.37 (w), 998.22 (w), 937.82 (w), 889.33 (w), 827.46 (w), 768.38 (m), 747.93 (m), 701.80 (m), 614.86 (w), 579.49 (w), 555.26 (w) cm^{-1}

^{1}H NMR (400 MHz, $CDCl_3$) δ 9.88 (s, 1 H), 7.92 (d, *J* = 7.6 Hz, 1 H), 7.59 (d, *J* = 7.6 Hz, 1 H), 7.53-7.44 (m, 2 H), 7.32-7.22 (m, 6 H), 7.18-7.16 (m, 3 H), 7.01-6.98 (m, 2 H), 6.94-6.91 (m, 1 H), 4.60 (s, 2 H), 4.32 (d, *J* = 7.2 Hz, 1 H), 3.27 (dd, *J* =7.6, 4.8 Hz, 1 H), 3.01-2.96 (m, 1 H), 2.41-2.34 (m, 1 H), 2.12-2.04 (m, 1 H), 1.89-1.83 (m, 1 H). **^{13}C NMR (125 MHz, $CDCl_3$)** δ 191.8, 179.1, 178.2, 161.7 (d, *J* =248.8 Hz), 141.0, 140.4, 139.1, 137.4, 135.9, 135.7 (d, *J* =7.5 Hz), 134.7, 132.4, 132.1 (d, *J* =8.8 Hz), 129.4, 129.2, 128.72, 128.67, 128.5, 128.2, 128.0, 127.6, 118.7 (d, *J* =22.5 Hz), 116.3 (d, *J* =20.0 Hz), 70.8, 43.1, 42.5, 40.9, 35.5, 25.0 .**^{19}F NMR (376 MHz, $CDCl_3$)** δ -113.57.

HRMS (ESI^+) $[M+Na]^+$ Calcd for $C_{33}H_{26}NO_4FNa$: 542.1738 m/z, Found: 542.1729 m/z;

Specific rotation: $[\alpha]_D^{24.4}$32.1 (*c* 0.50, $CHCl_3$) for an enantiomerically enriched sample of >99.5:0.5 e.r.

Enantiomeric purity of **7i** was determined by HPLC analysis in comparison with authentic racemic material (>99.5:0.5 e.r. shown; Chiralpak IB N-5 column, 80:20 hexane / ^{i}PrOH, 1.0 mL/min, 254 nm).

<Sample Information>

Sample Name : CYL-10040-6
Sample ID :
Data Filename : CYL-10040-6RAC-IBN-5-80-20-1.0.lcd
Method Filename : ZJW-4-8020-1.0-60-1min.lcm
Batch Filename : ZJW.lcb
Vial # : 1-100 Sample Type : Unknown
Injection Volume : 20 uL
Date Acquired : 5/19/2024 8:00:41 PM Acquired by : System Administrator
Date Processed : 5/20/2024 8:41:49 AM Processed by : System Administrator

<Chromatogram>

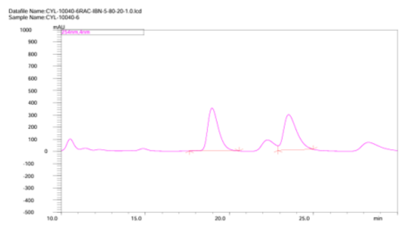

<Peak Table>

PDA Ch1 254nm

Peak#	Ret. Time	Area	Area%
1	18.969	15709568	49.997
2	23.530	15711527	50.003
Total		31421095	100.000

<Sample Information>

Sample Name : CYL-10015
Sample ID :
Data Filename : CYL-10015-IBN-5-80-20-1.0.lcd
Method Filename : ZJW-4-8020-1.0-60-1min.lcm
Batch Filename : ZJW.lcb
Vial # : 1-101 Sample Type : Unknown
Injection Volume : 20 uL
Date Acquired : 5/19/2024 9:01:14 PM Acquired by : System Administrator
Date Processed : 5/20/2024 8:40:22 AM Processed by : System Administrator

<Chromatogram>

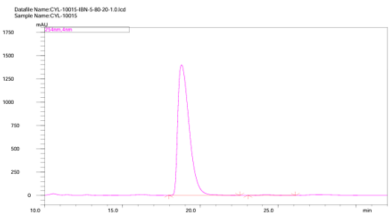

<Peak Table>

PDA Ch1 254nm

Peak#	Ret. Time	Area	Area%
1	18.784	70575027	99.581
2	24.202	296713	0.419
Total		70871741	100.000

(*R*)-6-((*R*)-((1*R*,5*S*,6*R*)-3-benzyl-2,4-dioxo-3-azabicyclo[3.2.0]heptan-6-yl)(hydroxy)methyl)-5'-methyl-[1,1':2',1''-terphenyl]-2-carbaldehyde (7j)

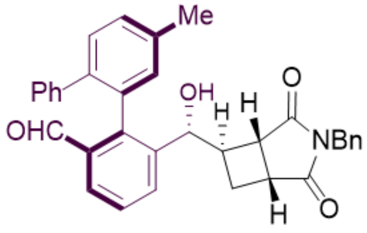

7j

colorless oil; **IR (neat):** 3406.39 (br), 1767.76 (w), 1694.44 (s), 1587.97 (w), 1480.03 (w), 1430.85 (w), 1388.58 (m), 1343.15 (m), 1310.09 (w), 1289.67 (w), 1265.23 (w), 1238.70 (w), 1155.37 (m), 1124.59 (w), 1100.62 (w), 1071.26 (w), 1001.37 (w), 965.72 (w), 910.01 (m), 827.78 (m), 809.82 (m), 774.16 (s), 727.36 (s), 699.19 (w), 647.73 (w), 616.97 (m), 590.20 (w), 574.13 (w), 532.94 (w), 513.22 (w), 469.68 (w) cm^{-1}

^{1}H NMR (400 MHz, $CDCl_3$) δ 9.83 (s, 1 H), 7.89-7.87 (m, 1 H), 7.50-7.48 (m, 1 H), 7.39-7.35 (m, 2 H), 7.30-7.09 (m, 9 H), 6.96-6.93 (m, 3 H), 4.54 (s, 2 H), 4.25 (d, J = 8.0 Hz, 1 H), 3.17 (dd, J =7.2, 4.8 Hz, 1 H), 2.91-2.85 (m, 1 H), 2.34 (s, 3 H), 2.31-2.26 (m, 1 H), 1.97-1.89 (m, 1 H), 1.77-1.71 (m, 1 H). **^{13}C NMR (100 MHz, $CDCl_3$)** δ 192.6, 179.3, 178.2, 142.9, 140.5, 140.1, 138.3, 137.7, 136.0, 135.1, 133.4, 132.9, 132.4, 130.2, 130.1, 129.5, 128.77, 128.75, 128.7, 128.5, 128.0, 127.6, 127.4, 71.2, 43.3, 42.6, 41.4, 35.6, 25.0, 21.3.

HRMS (ESI$^+$) [M+Na]$^+$ Calcd for $C_{34}H_{29}NO_4Na$: 538.1989 m/z, Found: 538.1987 m/z;

Specific rotation: $[\alpha]_D^{28.4}$18.1 (c 0.50, $CHCl_3$) for an enantiomerically enriched sample of 99.5:0.5 e.r.

Enantiomeric purity of **7j** was determined by HPLC analysis in comparison with authentic racemic material (99.5:0.5 e.r. shown; Chiralpak IB N-5 column, 80:20 hexane / ^{i}PrOH, 1.0 mL/min, 254 nm).

<Sample Information>

Sample Name : CYL-10040-5
Sample ID :
Data Filename : CYL-10040-5RAC-IBN-5-80-20-1.0.lcd
Method Filename : ZJW-4-8020-1.0-80-1min.lcm
Batch Filename : ZJW.lcb
Vial # : 1-80 Sample Type : Unknown
Injection Volume : 20 uL
Date Acquired : 5/19/2024 3:09:52 AM Acquired by : System Administrator
Date Processed : 5/19/2024 12:45:11 PM Processed by : System Administrator

<Chromatogram>

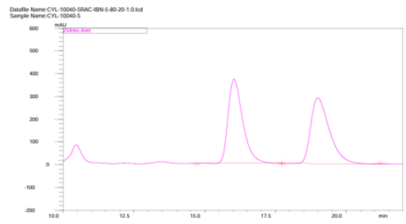

<Peak Table>

PDA Ch1 254nm

Peak#	Ret. Time	Area	Area%
1	16.032	13277541	50.262
2	18.996	13139158	49.738
Total		26416699	100.000

<Sample Information>

Sample Name : CYL-10014
Sample ID :
Data Filename : CYL-10014-IBN-5-80-20-1.0.lcd
Method Filename : ZJW-4-8020-1.0-80-1min.lcm
Batch Filename : ZJW.lcb
Vial # : 1-81 Sample Type : Unknown
Injection Volume : 20 uL
Date Acquired : 5/19/2024 4:30:25 AM Acquired by : System Administrator
Date Processed : 8/18/2024 10:56:59 PM Processed by : System Administrator

<Chromatogram>

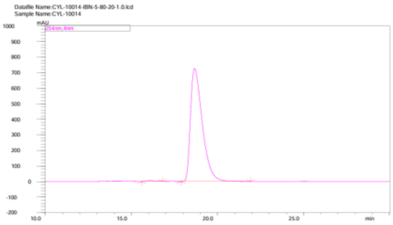

<Peak Table>

PDA Ch1 254nm

Peak#	Ret. Time	Area	Area%
1	16.085	170719	0.489
2	18.673	34706654	99.511
Total		34877373	100.000

(*R*)-6-((*R*)-((1*R*,5*S*,6*R*)-3-benzyl-2,4-dioxo-3-azabicyclo[3.2.0]heptan-6-yl) (hydroxy)methyl)-4-methoxy-[1,1':2',1''-terphenyl]-2-carbaldehyde (7k)

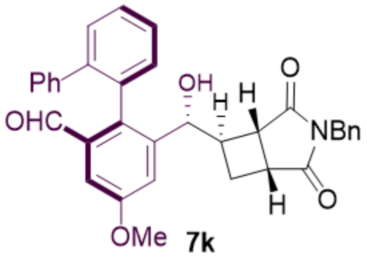

7k

colorless oil; **IR (neat):** 3502.27 (br), 3062.62 (w), 2938.93 (w), 2850.25 (w), 1769.04 (w), 1699.20 (s), 1600.70 (w), 1462.94 (w), 1430.88 (w), 1391.54 (m), 1341.57 (w), 1308.29 (m), 1162.18 (m), 1123.52 (w), 999.67 (w), 946.15 (w), 748.62 (m), 725.79 (w), 701.51 (m), 616.40 (w), 473.09 (w), 419.27 (w) cm^{-1}

^{1}H NMR (400 MHz, $CDCl_3$) δ 9.79 (s, 1 H), 7.48 (d, *J* =4.8 Hz, 2 H), 7.39-7.33 (m, 2 H), 7.26-7.15 (m, 8 H), 7.09 (d, *J* = 6.8 Hz, 1 H), 7.04 (d, *J* = 2.0 Hz, 1 H), 6.98-6.96 (m, 2 H), 4.54 (s, 2 H), 4.21 (d, *J* = 6.8 Hz, 1 H), 3.78 (s, 3 H),3.15-3.13 (m, 1 H), 2.88-2.83 (m, 1 H), 2.30-2.24 (m, 1 H), 1.97-1.90 (m, 1 H), 1.77-1.70 (m, 1 H). **^{13}C NMR (100 MHz, $CDCl_3$)** δ 192.2, 179.3, 178.2, 159.7, 142.3, 141.6, 140.4, 136.2, 136.0, 135.7, 133.5, 132.9, 130.4, 129.5, 129.3, 128.8, 128.6, 128.0, 127.7, 127.5, 119.2, 111.1, 71.1, 55.7, 43.2, 42.6, 41.3, 35.6, 25.0.

HRMS (ESI$^+$) [M+Na]$^+$ Calcd for $C_{34}H_{29}NO_5Na$: 554.1938 m/z, Found: 554.1938 m/z; **Specific rotation:** $[\alpha]_D^{28.7}$-25.6 (*c* 0.20, $CHCl_3$) for an enantiomerically enriched sample of >99.5:0.5 e.r.

Enantiomeric purity of **7k** was determined by HPLC analysis in comparison with authentic racemic material (>99.5:0.5 e.r. shown; Chiralpak IB N-5 column, 80:20 hexane / ^{i}PrOH, 1.0 mL/min, 254 nm).

<Sample Information>

Sample Name : cyl-10040-8
Sample ID :
Data Filename : cyl-10040-8NEW-IBN-5-80-20-1.0.lcd
Method Filename : cyl-6hao-80-20-1.0ml-60min.lcm
Batch Filename : WWLL.lcb
Vial # : 1-89 Sample Type : Unknown
Injection Volume : 10 uL
Date Acquired : 9/12/2024 7:15:27 Acquired by : System Administrator
Date Processed : 9/12/2024 8:15:30 Processed by : System Administrator

<Chromatogram>

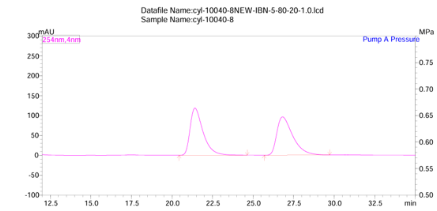

<Peak Table>

PDA Ch1 254nm

Peak#	Ret. Time	Area	Area%
1	21.415	6607241	50.111
2	26.819	6578032	49.889
Total		13185273	100.000

<Sample Information>

Sample Name : cyl-10017
Sample ID :
Data Filename : cyl-10017NEW-IBN-5-80-120-1.0.lcd
Method Filename : cyl-6hao-80-20-1.0ml-60min.lcm
Batch Filename : WWLL.lcb
Vial # : 1-88 Sample Type : Unknown
Injection Volume : 10 uL
Date Acquired : 9/12/2024 6:14:44 Acquired by : System Administrator
Date Processed : 9/12/2024 7:14:47 Processed by : System Administrator

<Chromatogram>

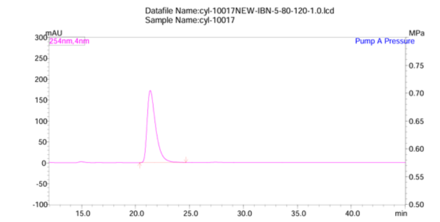

<Peak Table>

PDA Ch1 254nm

Peak#	Ret. Time	Area	Area%
1	21.367	9556717	100.000
Total		9556717	100.000

benzyl(1*R*,5*R*,6*R*)-6-((*R*)-((*R*)-6-formyl-[1,1':2',1''-terphenyl]-2-yl)(hydroxy)methyl)-3-azabicyclo[3.2.0]heptane-3-carboxylate (8a)

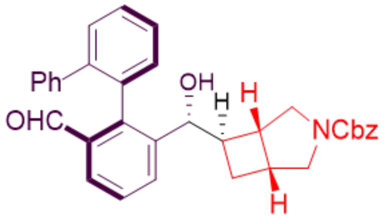

8a

colorless oil; **IR (neat):** 3448.69 (br), 3060.93 (w), 2927.91 (w), 2857.14 (w), 1688.48 (s), 1588.65 (w), 1449.25 (w), 1418.61 (m), 1358.10 (m), 1237.00 (w), 1146.68 (w), 1098.75 (w), 1028.27 (w), 1007.61 (w), 913.86 (w), 802.19 (w), 748.08 (m), 699.75 (m) cm^{-1}

^{1}H NMR (400 MHz, $CDCl_3$) δ 9.84 (s, 1 H), 7.87-7.85 (m, 1 H), 7.50-7.11 (m, 14 H), 7.00-6.97 (m, 2 H), 5.05 (s, 2 H), 4.04 (d, J = 8.0 Hz, 1 H), 3.41 (br, 2 H), 3.22-3.10 (m, 2 H), 2.65-2.61 (m, 1 H), 2.48-2.44 (m, 1 H), 2.08-2.00 (m, 1 H), 1.41-1.36 (m, 1 H), 1.29-1.18 (m, 1 H), 0.46 (br, 1 H). **^{13}C NMR (100 MHz, $CDCl_3$)** δ 192.6, 155.5, 142.7, 141.8, 141.3, 140.3, 137.1, 134.9, 133.9, 132.5, 130.2, 129.6, 129.12, 129.10, 128.6, 128.4, 128.0, 127.88, 127.86, 127.5, 127.3, 72.7, 66.8, 52.7 (m), 44.2, 41.3 (m), 33.7 (m), 29.8, 26.6.

HRMS (ESI^{+}) [M+Na]$^{+}$ Calcd for $C_{34}H_{31}NO_4Na$: 540.2145 m/z, Found: 540.2149 m/z;

Specific rotation: $[\alpha]_D^{26.6}$12.4 (c 0.50, $CHCl_3$) for an enantiomerically enriched sample of 99:1 e.r.

Enantiomeric purity of **8a** was determined by HPLC analysis in comparison with authentic racemic material (99:1 e.r. shown; Chiralpak IB N-5 column, 90:10 hexane / ^{i}PrOH, 1.0 mL/min, 220 nm).

<Sample Information>

Sample Name : CYL-10059-2
Sample ID :
Data Filename : CYL-10059-2RAC-IBN-5-90-10-1.0.lcd
Method Filename : ZJW-4-9010-1.0-120-1min.lcm
Batch Filename : ZJW.lcb
Vial # : 1-91 Sample Type : Unknown
Injection Volume : 20 uL
Date Acquired : 6/3/2024 3:39:52 PM Acquired by : System Administrator
Date Processed : 6/3/2024 5:21:20 PM Processed by : System Administrator

<Chromatogram>

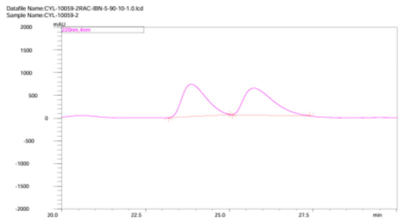

<Peak Table>

PDA Ch2 220nm

Peak#	Ret. Time	Area	Area%
1	23.865	35553444	49.601
2	25.731	36125828	50.399
Total		71679272	100.000

<Sample Information>

Sample Name : CYL-10057
Sample ID :
Data Filename : CYL-10057-IBN-5-90-10-1.0.lcd
Method Filename : ZJW-4-9010-1.0-50min.lcm
Batch Filename : ZJW.lcb
Vial # : 1-92 Sample Type : Unknown
Injection Volume : 20 uL
Date Acquired : 6/3/2024 5:22:40 PM Acquired by : System Administrator
Date Processed : 6/3/2024 6:12:43 PM Processed by : System Administrator

<Chromatogram>

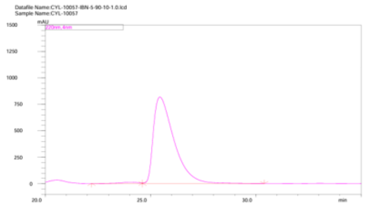

<Peak Table>

PDA Ch2 220nm

Peak#	Ret. Time	Area	Area%
1	24.031	757178	1.344
2	25.441	55588362	98.656
Total		56345540	100.000

■ Experimental Procedure and Characterization for Gram-Scale Reaction and Functionalization

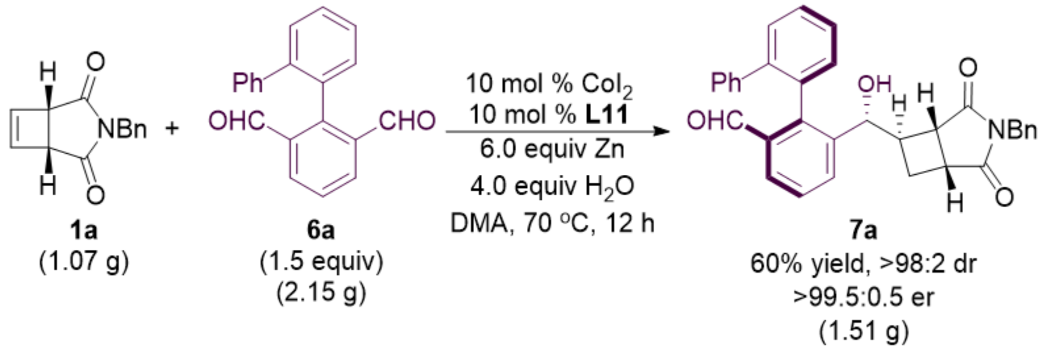

In a N_2-filled glove-box, a 250-mL round-bottomed flask equipped with a magnetic stir bar was charged with CoI_2 (156.4 mg, 0.50 mmol, 10 mol %), **L11** (297.3 mg, 0.50 mmol, 10 mol %) and Zn (1.96 g, 30.0 mmol, 6.0 equiv.). DMA (50 mL) was added, then the mixture was allowed to stir at room temperature for 30 min. Then **1a (**1.07 g, 5.0 mmol, 1.0 equiv.) and **6a** (2.15 g, 15.0 mmol, 1.5 equiv.) were added to the solution. It was allowed to stir at 70 °C for 12 h.

Workup: Upon completion, the reaction was quenched by 250 mL H_2O and extracted with diethyl ether (3*150 mL). The combined organic layer was washed with brine (100 mL) and dried over Na_2SO_4. After the solids were filtered off, the solvent was removed under reduced pressure and the residue was purified by silica-gel column chromatography (eluent: Petroleum ether/ EtOAc =7:3) to afford **7a** as a yellow solid (1.51 g, 60% yield).

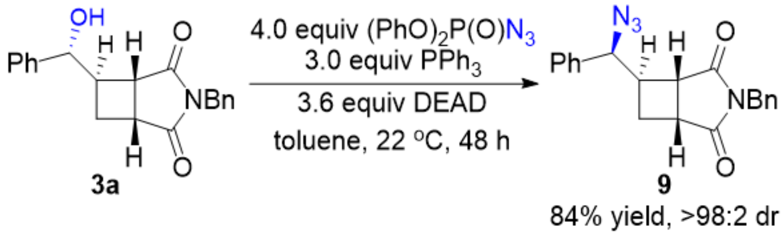

Following a known procedure[4]: In a N_2-filled glove-box, an oven-dried vial (8 mL) equipped with a magnetic stir bar was charged with $(PhO)_2P$ $(O)N_3$ (55.5 mg, 0.2 mmol, 4.0 equiv.), PPh_3 (39.4 mg, 0.15 mmol, 3.0 equiv.), DEAD (31.4 mg, 0.18 mmol, 3.6 equiv.), toluene (1 mL) and **3a** (16.1 mg, 0.05 mmol, 1.0 equiv.). The vial was sealed with a cap (phenolic open top cap with red PTFE/white silicone septum) and the solution was allowed to stir at 22 °C for 48 h. After completion, the mixture was purified by preparative TLC (petroleum ether/ethyl acetate = 3:1) to afford the **9** as colorless oil (14.5 mg, 84% yield).

(1*R*,5*S*,6*R*)-6-((*S*)-azido(phenyl)methyl)-3-benzyl-3-azabicyclo[3.2.0]heptane-2,4-dione (9)

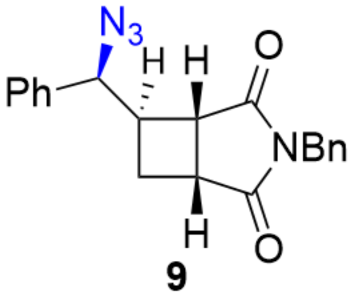

colorless oil; **IR (neat):** 2098.68 (s), 1770.40 (w), 1699.03 (s), 1494.44 (w), 1452.94 (w), 1428.02 (w), 1388.21 (m), 1341.63 (m), 1310.85 (w), 1289.82 (w), 1259.41 (w), 1164.01 (m), 1077.40 (w), 1060.40 (w), 1028.20 (w), 930.28 (w), 802.79 (m), 741.73 (w), 699.03 (s), 647.08 (w), 619.60 (w) cm^{-1}

^{1}H NMR (400 MHz, $CDCl_3$) δ 7.34-7.15 (m, 10 H),4.64 (d, J = 5.6 Hz, 1 H), 4.57 (s, 2 H), 3.14-3.04 (m, 2 H), 2.67-2.55 (m, 2 H), 2.11-2.04 (m, 1 H), 2.23-2.16 (m, 1 H), 2.14 (s, 3 H), 1.86 (s, 3 H). **^{13}C NMR (100 MHz, $CDCl_3$)** δ 178.8, 178.1, 136.6, 135.9, 129.3, 129.1, 128.8, 128.1, 127.2, 68.5, 42.7, 42.0, 41.8, 35.6, 25.2.

HRMS (DART^{+}) [M+H]$^{+}$ Calcd for $C_{20}H_{19}O_2N_4$: 347.1503 m/z, Found: 347.1502 m/z;

Specific rotation: $[\alpha]_D^{24.9}$-22.6 (c 0.50, $CHCl_3$)

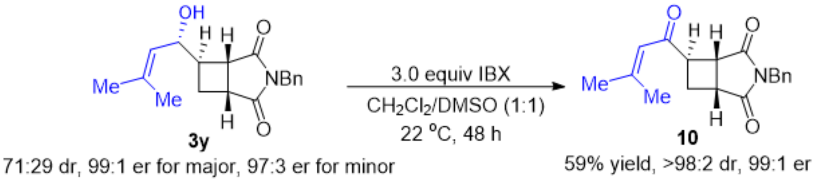

Following a known procedure[5]: A an oven-dried vial (8 mL) equipped with a magnetic stir bar was charged with **3y** (29.9 mg, 0.1 mmol, 1.0 equiv.), IBX (84.0 mg, 0.3 mmol, 3.0 equiv.) 2 mL DCM and 2 mL DMSO. The resulting reaction mixture was stirred for 48 h at room temperature and quenched with H_2O (20 mL). The resulting aqueous phase was extracted with DCM (10 mL×3). The combined organic phase was dried over Na_2SO_4. After removal of the solvent, the resulting crude mixture was purified by silica gel column chromatography (petroleum ether/ethyl acetate = 3:1) to afford **10** as colorless oil (17.5 mg, 59%).

(1*R*,5*S*,6*R*)-3-benzyl-6-(3-methylbut-2-enoyl)-3-azabicyclo[3.2.0]heptane-2,4-dione (10)

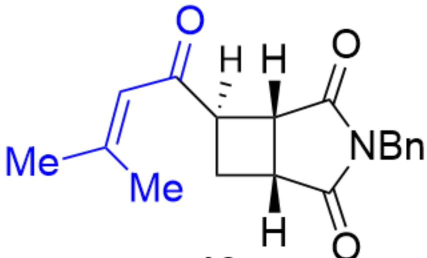

10

colorless oil; **IR (neat):** 21766.14 (w), 1685.85 (s), 1618.00 (m), 1496.52 (w), 1432.63 (m), 1392.62 (m), 1363.30 (m), 1334.91 (m), 1302.65 (w), 1250.29 (w), 1207.26 (w), 1175.48 (m), 1157.25 (m),, 1133.14 (m), 1105.17 (w), 1048.98 (w), 1004.63 (w), 938.24 (m), 855.67 (w), 841.38 (w), 799.71 (w), 732.56 (m), 715.19 (w), 692.54 (m), 649.21 (m), 630.86 (m), 598.03 (w), 577.15 (w), 457.65 (w), 431.73 (w) cm^{-1}

^{1}H NMR (400 MHz, $CDCl_3$) δ 7.34-7.31 (m, 2 H), 7.28-7.19 (m, 3 H), 5.94 (s, 1 H), 4.64 (s, 2 H), 3.45 (dd, *J* = 6.4, 4.8 Hz, 1 H), 3.17-3.09 (m, 2 H), 2.76-2.69 (m, 1 H), 2.35-2.28 (m, 1 H), 2.23-2.16 (m, 1 H), 2.14 (s, 3 H), 1.86 (s, 3 H). **^{13}C NMR (150 MHz, $CDCl_3$)** δ 196.5, 178.9, 159.8, 136.0, 128.9, 128.8, 128.2, 121.2, 46.8, 42.7, 40.7, 35.8, 28.1, 25.2, 21.3.

HRMS (ESI$^+$) [M+Na]$^+$ Calcd for $C_{18}H_{19}NO_3Na$: 320.1257 m/z, Found: 320.1257 m/z;

Specific rotation: $[\alpha]_D^{24.6}$-64.4 (*c* 0.50, $CHCl_3$) for an enantiomerically enriched sample of 99:1 e.r.

Enantiomeric purity of **10** was determined by HPLC analysis in comparison with authentic racemic material (99:1 e.r. shown; Chiralpak IG column, 90:10 hexane / ^{i}PrOH, 1.0 mL/min, 254 nm).

<Sample Information>

Sample Name : cyl-10076RAC
Sample ID :
Data Filename : cyl-10076RACNEW-IG-90-10-1.0.lcd
Method Filename : cyl-1hao-90-10-1.0ml-60min.lcm
Batch Filename : WWLL.lcb
Vial # : 1-72
Sample Type : Unknown
Injection Volume : 10 uL
Date Acquired : 9/10/2024 1:41:56
Acquired by : System Administrator
Date Processed : 9/10/2024 2:41:59
Processed by : System Administrator

<Chromatogram>

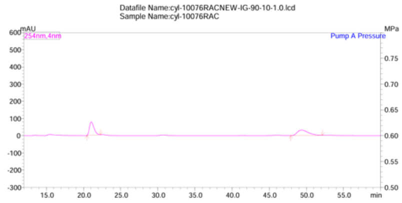

<Peak Table>

PDA Ch1 254nm

Peak#	Ret. Time	Area	Area%
1	21.048	3297088	50.256
2	49.353	3263489	49.744
Total		6560577	100.000

<Sample Information>

Sample Name : cyl-10076
Sample ID :
Data Filename : cyl-10076NEW-IG-90-10-1.0.lcd
Method Filename : cyl-1hao-90-10-1.0ml-60min.lcm
Batch Filename : WWLL.lcb
Vial # : 1-71
Sample Type : Unknown
Injection Volume : 10 uL
Date Acquired : 9/10/2024 0:41:13
Acquired by : System Administrator
Date Processed : 9/10/2024 1:41:16
Processed by : System Administrator

<Chromatogram>

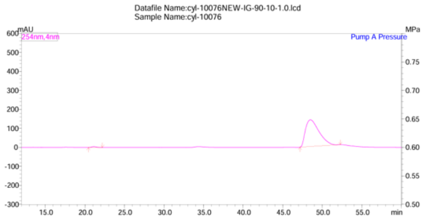

<Peak Table>

PDA Ch1 254nm

Peak#	Ret. Time	Area	Area%
1	21.120	175223	1.030
2	48.512	16829993	98.970
Total		17005215	100.000

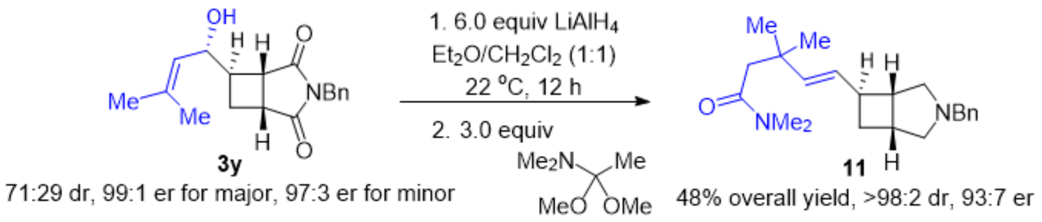

Following a known procedure[6]: In a N_2-filled glove-box, a 100-mL round-bottomed flask equipped with a magnetic stir bar was charged with **3y** (239.5 mg, 0.8 mmol, 1.0 equiv.), $LiAlH_4$ (182.4 mg, 4.8 mmol, 6.0 equiv.), 5 mL DCM and 5 mL Et_2O. The vial was sealed with a cap (phenolic open top cap with red PTFE/white silicone septum) and the solution was allowed to stir at 22 °C for 12 h. Upon completion, the reaction was quenched by 5 mL saturated aqueous solution of NH_4Cl and extracted with DCM (3×30 mL). The combined organic layer was washed with brine (30 mL) and dried over Na_2SO_4. After the solids were filtered off, the solvent was removed under reduced pressure and the residue was purified by silica-gel column chromatography (eluent: hexanes/ethyl acetate = 3:1) to afford the intermediate as yellow oil (166.6 mg, 76% yield).

Following a known procedure[7]: A 25-mL round-bottomed flask equipped with a magnetic stir bar was charged with intermediate (27.1 mg, 0.1 mmol, 1.0 equiv.), 1,1-dimethoxy-N,N-dimethylethan-1-amine (40.0 mg, 0.3 mmol, 3.0 equiv.) and 2 mL xylene. The solution was allowed to stir at 130 °C for 20 h. Upon completion, the solvent was removed under reduced pressure and the residue was purified by silica-gel column chromatography (eluent: hexanes/ethyl acetate = 1:1) to afford the **11** as colorless oil (21.5 mg, 63% yield).

(*E*)-5-((1*R*,5*S*,6*S*)-3-benzyl-3-azabicyclo[3.2.0]heptan-6-yl)-N,N,3,3-tetramethylpent-4-enamide (11)

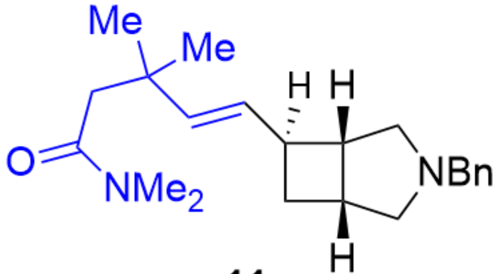

11

colorless oil; **IR (neat):** 2926.83 (m), 2779.05 (w), 1640.97 (s), 1493.38 (w), 1467.72 (w), 1453.02 (w), 1393.32 (w), 1378.33 (w), 1343.39 (w), 1261.07 (w), 1176.70 (w), 1107.53 (m), 1026.74 (w), 972.58 (m), 740.60 (m), 699.32 (m) cm^{-1}

^{1}H NMR (400 MHz, CDCl$_3$) δ 7.34-7.15 (m, 5 H), 5.46 (dd, *J* = 15.6, 7.2 Hz, 1 H), 5.35 (d, *J* = 15.6 Hz, 1H), 3.66-3.57 (m, 2 H), 2.88 (s, 3 H), 2.84 (s, 3 H), 2.77-2.73 (m, 2 H), 2.64-2.58 (m, 2 H), 2.45-2.40 (m, 1 H), 2.23 (s, 2 H), 2.07-1.98 (m, 2 H), 1.95-1.88 (m, 1 H), 1.86-1.79 (m, 1 H), 1.08 (s, 6 H). **^{13}C NMR (100 MHz, CDCl$_3$)** δ 171.7, 136.8, 131.4, 128.8, 128.3, 126.8, 60.5, 60.0, 59.8, 44.9, 44.3, 40.3, 38.7, 36.0, 35.5, 33.9, 31.0, 28.04, 27.97.

HRMS (ESI$^+$) [M+Na]$^+$ Calcd for $C_{22}H_{33}N_2O$:341.2587 m/z, Found: 341.2588 m/z; **Specific rotation:** $[\alpha]_D^{25.5}$-44.5 (*c* 0.50, CHCl$_3$) for an enantiomerically enriched sample of 98:2 e.r.

Enantiomeric purity of **11** was determined by HPLC analysis in comparison with authentic racemic material (98:2 e.r. shown; Chiralpak IG column, 90:10 hexane / ^{i}PrOH, 1.0 mL/min, 220 nm).

<Sample Information>

Sample Name : cyl-11007rac
Sample ID :
Data Filename : cyl-11007rac-ID-90-10-1.0.lcd
Method Filename : cyl-5hao-90-10-1.0ml-90min.lcm
Batch Filename : WWLL.lcb
Vial # : 1-80 Sample Type : Unknown
Injection Volume : 10 uL
Date Acquired : 8/23/2024 20:15:59 Acquired by : System Administrator
Date Processed : 9/14/2024 9:06:20 Processed by : System Administrator

<Chromatogram>

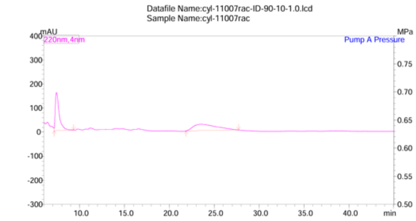

<Peak Table>

PDA Ch2 220nm

Peak#	Ret. Time	Area	Area%
1	7.416	4896028	49.870
2	23.534	4921525	50.130
Total		9817554	100.000

<Sample Information>

Sample Name : cyl-11007
Sample ID :
Data Filename : cyl-11007-ID-90-10-1.0.lcd
Method Filename : cyl-5hao-90-10-1.0ml-90min.lcm
Batch Filename : WWLL.lcb
Vial # : 1-79 Sample Type : Unknown
Injection Volume : 10 uL
Date Acquired : 8/23/2024 21:17:27 Acquired by : System Administrator
Date Processed : 9/14/2024 9:07:06 Processed by : System Administrator

<Chromatogram>

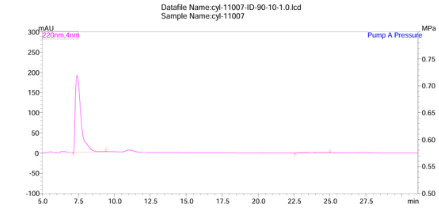

<Peak Table>

PDA Ch2 220nm

Peak#	Ret. Time	Area	Area%
1	7.413	5312747	98.448
2	23.674	83762	1.552
Total		5396509	100.000

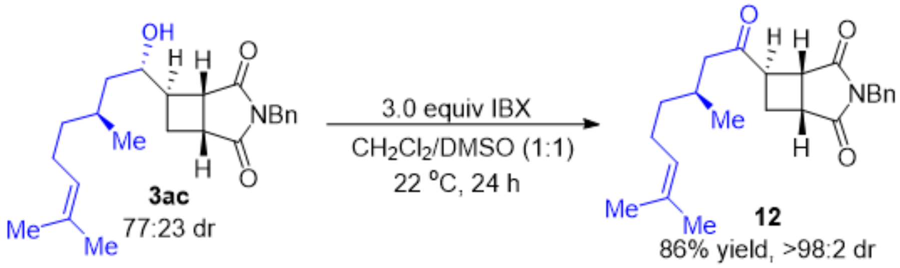

Following a known procedure[5]: An oven-dried vial (8 mL) equipped with a magnetic stir bar was charged with **3ac** (27.0 mg, 0.07 mmol, 1.0 equiv.), IBX (58.8 mg, 0.21 mmol, 3.0 equiv.), 1 mL DCM and 1 mL DMSO. The resulting reaction mixture was stirred for 24 h at room temperature and quenched with H_2O (10 mL). The resulting aqueous phase was extracted with DCM (10 mL × 3). The combined organic phase was dried over Na_2SO_4. After removal of the solvent, the resulting crude mixture was purified by silica gel column chromatography (petroleum ether/ethyl acetate = 4:1) to afford **12** as colorless oil (22.2 mg, 86%).

(1*R*,5*S*,6*R*)-3-benzyl-6-((*S*)-3,7-dimethyloct-6-enoyl)-3-azabicyclo[3.2.0]heptane-2,4-dione (12)

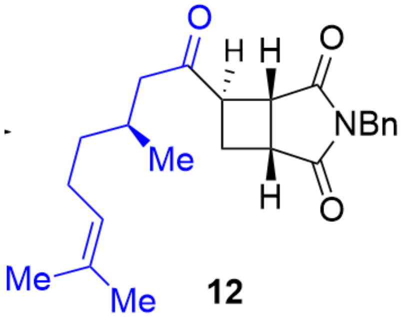

12

colorless oil; **IR (neat):** 2970.07 (w), 2907.79 (w), 2873.86 (w), 2851.90 (w), 1775.03 (w), 1696.57 (s), 1494.36 (w), 1451.54 (w), 1422.44 (w), 1390.89 (m), 1354.24 (m), 1325.54 (m), 1163.04 (s), 1129.28 (w), 1115.74 (w), 1083.53 (w), 1063.40 (w), 987.76 (w), 934.95 (m), 902.16 (w), 848.42 (w), 782.89 (m), 732.46 (m), 698.07 (w), 653.98 (m), 630.97 (m), 579.13 (m), 463.54 (m), 435.57 (w) cm^{-1}
^{1}H NMR (400 MHz, $CDCl_3$) δ 7.32-7.18 (m, 5 H), 5.01-4.96 (m, 1 H), 4.62 (s, 2 H), 3.40-3.37 (m, 1 H), 3.15-3.07 (m, 2 H), 2.71-2.63 (m, 1 H), 2.35 (dd, J = 16.0, 6.8 Hz, 1 H), 2.22-2.13 (m, 2 H), 1.98-2.13 (m, 3 H), 1.59 (s, 3 H), 1.50 (s, 3 H), 1.26-1.17 (m, 1 H), 1.15-1.06 (m, 1 H), 0.82 (d, J = 6.8 Hz, 3 H). **^{13}C NMR (100 MHz, $CDCl_3$)** δ 207.1, 178.6, 177.7, 135.9, 131.8, 128.9, 128.8, 128.2, 124.2, 48.0, 45.9, 42.8, 40.5, 37.0, 35.8, 28.7, 25.8, 25.6, 25.1, 19.9, 17.8.
HRMS (ESI$^+$) [M+Na]$^+$ Calcd for $C_{23}H_{29}NO_3Na$:390.2040 m/z, Found: 390.2043 m/z;
Specific rotation: $[\alpha]_D^{29.5}$-57.6 (*c* 0.50, $CHCl_3$)

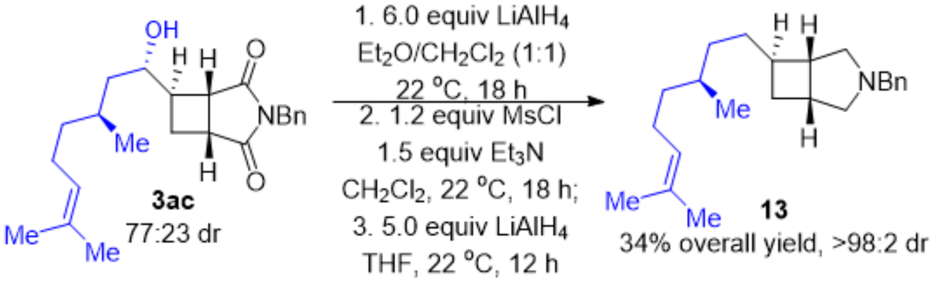

Following a known procedure[6]: In a N_2-filled glove-box, a 100-mL round-bottomed flask equipped with a magnetic stir bar was charged with **3ac (**622.6 mg, 1.7 mmol, 1.0 equiv.), $LiAlH_4$ (385.3 mg, 10.1 mmol, 6.0 equiv.), 10 mL DCM and 10 mL Et_2O. The vial was sealed with a cap (phenolic open top cap with red PTFE/white silicone septum) and the solution was allowed to stir at 22 °C for 18 h. Upon completion, the reaction was quenched by 10 mL saturated aqueous solution of NH_4Cl and extracted with DCM (3×30 mL). The combined organic layer was washed with brine (30 mL) and dried over Na_2SO_4. After the solids were filtered off, the solvent was removed under reduced pressure and the residue was purified by silica-gel column chromatography (eluent: hexanes/ethyl acetate = 3:1) to afford the intermediate as colorless oil (426.3 mg, 74% yield).

Following a known procedure[8]: In a N_2-filled glove-box, an oven-dried vial (8 mL) equipped with a magnetic stir bar was charged with the intermediate (68.2 mg, 0.2 mmol, 1.0 equiv.), 1 mL DCM, Et_3N (30.4 mg, 0.3 mmol, 1.5 equiv.) and MsCl (27.5 mg, 0.24 mmol, 1.2 equiv.). The solution was allowed to stir at 22 °C for 6 h. Upon completion, the reaction was quenched by 2 mL saturated aqueous solution of $NaHCO_3$ and extracted with DCM (3×10 mL). The combined organic layer was washed with brine (10 mL) and dried over Na_2SO_4. After the solids were filtered off, the solvent was removed under reduced pressure and the residue was purified by silica-gel column chromatography (eluent: hexanes/ethyl acetate =5:1) to afford the intermediate-2 as yellow oil (77.5 mg, 92% yield).

In a N_2-filled glove-box, a 100-mL round-bottomed flask equipped with a magnetic stir bar was charged with intermediate-2 (25.2 mg, 0.06 mmol, 1.0 equiv.), 2mL THF and $LiAlH_4$ (11.4 mg, 0.3 mmol, 5.0 equiv.). The solution was allowed to stir at 22 °C for 12 h. Upon completion, the reaction was quenched by 1 mL saturated aqueous solution of NH_4Cl and extracted with DCM (3×10 mL). The combined organic layer was washed with brine (10 mL) and dried over Na_2SO_4. After the solids were filtered off, the solvent was removed under reduced pressure and the residue was purified by silica-gel column chromatography (eluent: hexanes/ethyl acetate =10:1) to afford the **13** as colorless oil (9.8 mg, 50% yield).

(1*R*,5*S*,6*R*)-3-benzyl-6-((*S*)-3,7-dimethyloct-6-en-1-yl)-3-azabicyclo[3.2.0]heptane (13)

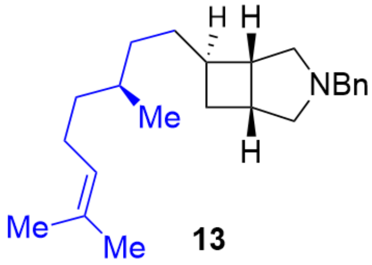

13

colorless oil; **IR (neat):** 2920.75 (s), 2852.13 (m), 2779.37 (m), 1454.58 (m), 1375.13 (w), 1343.63 (w), 1177.59 (w), 1117.25 (w), 968.05 (w), 852.81 (w), 738.28 (w), 698.10 (m) cm^{-1}
^{1}H NMR (400 MHz, $CDCl_3$) δ 7.34-7.32 (m, 2 H), 7.26-7.23 (m, 2 H), 7.19-7.17 (m, 1 H), 5.04-5.00 (m, 1 H), 3.61 (d, *J* = 3.2 Hz, 2 H),2.74-2.68 (m, 2 H), 2.62-2.55 (m, 1 H), 2.25-2.20 (m, 1 H), 2.06-1.99 (m, 2 H), 1.94-1.78 (m, 4 H), 1.60 (s, 3 H), 1.60-1.54 (m, 1 H), 1.53 (s, 3 H), 1.35-1.20 (m, 4 H), 1.16-1.00 (m, 2 H), 1.97-1.87 (m, 1 H), 0.78 (d, *J* = 7.2 Hz, 3 H). **^{13}C NMR (100 MHz, $CDCl_3$)** δ 131.1, 128.8, 128.3, 126.8, 125.2, 60.6, 60.5, 60.0, 43.8, 38.2, 37.3, 34.6, 34.2, 34.1, 32.6, 30.9, 25.9, 25.7, 19.8, 17.8.
HRMS (ESI^+) $[M+Na]^+$ Calcd for $C_{23}H_{36}N$:326.2842 m/z, Found: 326.2835 m/z; **Specific rotation:** $[\alpha]_D^{27.1}$-23.7 (*c* 0.50, $CHCl_3$)

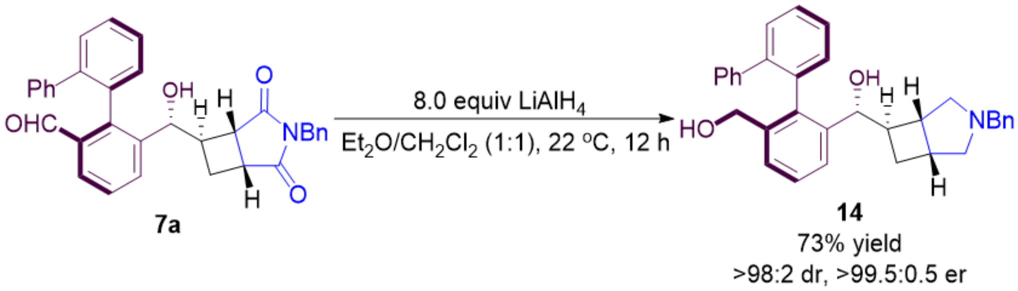

Following a known procedure[6]: In a N_2-filled glove-box, an oven-dried vial (8 mL) equipped with a magnetic stir bar was charged with **9a** (50.2 mg, 0.1 mmol, 1.0 equiv.), $LiAlH_4$ (30.4 mg, 0.8 mmol, 8.0 equiv.), 1 mL DCM and 1 mL Et_2O. The vial was sealed with a cap (phenolic open top cap with red PTFE/white silicone septum) and the solution was allowed to stir at 22 °C for 12 h. Upon completion, the reaction was quenched by 3 mL saturated aqueous solution of NH_4Cl and extracted with DCM (3×10 mL). The combined organic layer was washed with brine (10 mL) and dried over Na_2SO_4. After the solids were filtered off, the solvent was removed under reduced pressure and the residue was purified by silica-gel column chromatography (eluent: hexanes/ethyl acetate = 1:1) to afford the intermediate as colorless oil (34.7 mg, 73% yield).

(*R*)-((1*R*,5*R*,6*R*)-3-benzyl-3-azabicyclo[3.2.0]heptan-6-yl)((*S*)-6- (hydroxymethyl)-[1,1':2',1''-terphenyl]-2-yl)methanol (14)

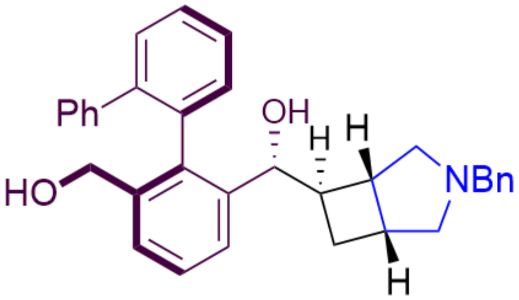

14

colorless oil; **IR (neat):** 3577.37 (w), 3443.69 (br), 3059.83 (w), 2926.09 (w), 2781.19 (w), 1468.29 (m), 1452.04 (w), 1431.53 (w), 1375.23 (w), 1343.58 (w), 1261.52 (w), 1232.72 (w), 1176.74 (w), 1094.07 (w), 1056.21 (w), 1024.21 (m), 1011.44 (m), 745.49 (s), 700.26 (s) cm^{-1}

^{1}H NMR (400 MHz, $CDCl_3$) δ 7.47-7.34 (m, 4 H), 7.27-7.04 (m, 13 H), 4.41 (s, 2 H), 4.06 (d, *J* = 8.4 Hz, 1 H), 3.59-3.50 (m, 2 H), 2.68 (d, *J* = 9.6 Hz, 1 H), 2.61 (d, *J* = 9.6 Hz, 1 H), 2.47-2.43 (m, 1 H), 2.33-2.27 (m, 1 H), 2.26-2.19 (m, 1 H), 2.00-1.91 (m, 2 H), 1.68 (br, 1 H), 1.41-1.30 (m, 2 H), 0.36 (br, 2 H). **^{13}C NMR (100 MHz, $CDCl_3$)** δ 141.0, 140.8, 140.7, 138.7, 138.3, 136.6, 131.5, 130.1, 129.5, 128.8, 128.42, 128.38, 128.3, 128.24, 128.21, 127.5, 127.2, 127.0, 126.8, 126.2, 74.1, 63.9, 60.3, 60.2, 59.8, 43.9, 41.3, 33.6, 26.5.

HRMS (ESI$^+$) [M+Na]$^+$ Calcd for $C_{33}H_{34}NO_2$:476.2584 m/z, Found: 476.2580 m/z; **Specific rotation:** $[\alpha]_D^{26.0}$2.4 (*c* 0.50, $CHCl_3$) for an enantiomerically enriched sample of >99.5:0.5 e.r.

Enantiomeric purity of **14** was determined by HPLC analysis in comparison with authentic racemic material (>99.5:0.5 e.r. shown; Chiralpak IB N-5 column, 95:5 hexane / ^{i}PrOH, 1.0 mL/min, 254 nm).

<Sample Information>

Sample Name : CYL-11014RAC
Sample ID :
Data Filename : CYL-11014RAC-IBN-5-95-5-1.0.lcd
Method Filename : ZJW-4-9505-1.0-90min.lcm
Batch Filename : ZJW.lcb
Vial # : 1-93 Sample Type : Unknown
Injection Volume : 10 uL
Date Acquired : 7/11/2024 4:41:12 AM Acquired by : System Administrator
Date Processed : 7/11/2024 9:02:43 AM Processed by : System Administrator

<Chromatogram>

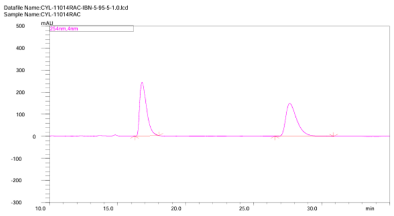

<Peak Table>

PDA Ch1 254nm

Peak#	Ret. Time	Area	Area%
1	16.782	8548020	50.400
2	27.650	8412316	49.600
Total		16960336	100.000

<Sample Information>

Sample Name : CYL-11014
Sample ID :
Data Filename : CYL-11014-IBN-5-95-5-1.0.lcd
Method Filename : ZJW-4-9505-1.0-90min.lcm
Batch Filename : ZJW.lcb
Vial # : 1-98 Sample Type : Unknown
Injection Volume : 10 uL
Date Acquired : 7/11/2024 10:16:45 AM Acquired by : System Administrator
Date Processed : 7/11/2024 2:15:25 PM Processed by : System Administrator

<Chromatogram>

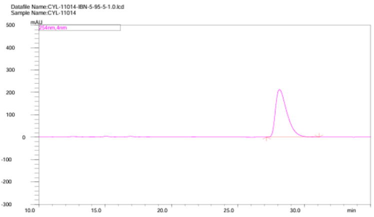

<Peak Table>

PDA Ch1 254nm

Peak#	Ret. Time	Area	Area%
1	28.132	12797514	100.000
Total		12797514	100.000

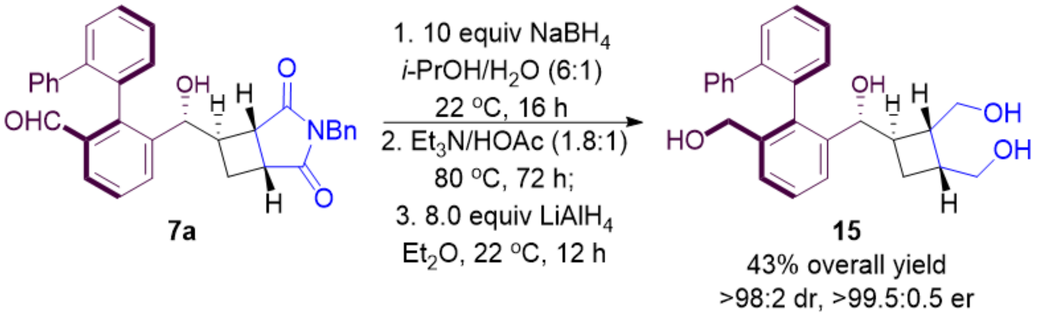

Following a known procedure[6]: In a N_2-filled glove-box, an oven-dried vial (8 mL) equipped with a magnetic stir bar was charged with **9a** (100.3 mg, 0.2 mmol, 1.0 equiv.) 1.8 mL *i*-PrOH, 0.3 mL H_2O and $NaBH_4$ (76.0 mg, 2 mmol, 10.0 equiv.) was added. The solution was allowed to stir at 22 °C for 16 h. Upon completion, the reaction was cooled to 0 °C and quenched by 1 mL saturated aqueous solution of Na_2CO_3 and extracted with DCM (3×30 mL). The combined organic layer was washed with brine (10 mL) and dried over Na_2SO_4. After the solids were filtered off, the solvent was removed under reduced pressure and the residue was used to next step without purified.

A 100-mL round-bottomed flask equipped with a magnetic stir bar was charged with the crude product, 13 mL Et_3N, 7 mL AcOH and heated to 80 °C for 72 h. The resulting mixture was cooled, poured onto ice cold saturated sodium bicarbonate, extrated with DCM (3×50 mL). The combined organic layer was washed with brine (20 mL) and dried over Na_2SO_4. After the solids were filtered off, the solvent was removed under reduced pressure and the residue was purified by silica-gel column chromatography (eluent: hexanes/ethyl acetate = 1:1) to afford the lactone as red-brown oil (2 steps yield 59%).

In a N_2-filled glove-box, an oven-dried vial (8 mL) equipped with a magnetic stir bar was charged with the lactone (48.0 mg, 0.12 mmol, 1.0 equiv.), 2 mL Et_2O and lithium aluminum hydride (36.5 mg, 0.96 mmol, 8.0 equiv.). The reaction was stirred at 22 °C for 12 h then was quenched with water and extracted with DCM (3×30 mL). The combined organic layer was washed with brine (10 mL) and dried over Na_2SO_4. After the solids were filtered off, the solvent was removed under reduced pressure and the residue was purified by silica-gel column chromatography (eluent: hexanes/ethyl acetate = 1:3) to afford the **15** as milky oil (34.5 mg, 79% yield).

((1*R*,2*R*,3*R*)-3-((*R*)-hydroxy((*S*)-6-(hydroxymethyl)-[1,1':2',1''-terphenyl]-2-yl)methyl)cyclobutane-1,2-diyl)dimethanol (15)

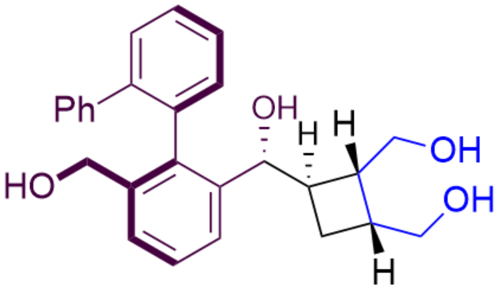

15

milky white oil; **IR (neat):** 3330.64 (br), 2926.51 (w), 2868.32 (w), 1479.86 (w), 1447.95 (w), 1426.81 (m), 1345.37 (w), 1259.16 (w), 1217.21 (w), 1013.47 (s), 908.90 (m), 743.86 (s), 730.41 (s), 700.14 (s), 643.89 (w), 615.44 (w), 569.94 (w), 546.82 (w), 509.69 (w) cm^{-1}

^{1}H NMR (400 MHz, $CDCl_3$) δ 7.46-7.33 (m, 4 H), 7.26-7.22 (m, 1 H), 7.14-7.07 (m, 5 H), 7.02-7.00 (m, 2 H), 4.43 (s, 2 H), 3.98 (d, *J* = 8.4 Hz, 1 H), 3.62-3.51 (m, 2 H), 3.45-3.41 (m, 2 H), 2.26-2.08 (m, 4 H), 1.28-1.08 (m, 3 H). **^{13}C NMR (100 MHz, $CDCl_3$)** δ 140.8, 140.7, 140.5, 138.9, 138.2, 136.3, 131.5, 130.2, 129.5, 128.6, 128.5, 128.2, 127.6, 127.3, 127.2, 125.9, 74.3, 63.8, 63.1, 62.7, 43.9, 43.2, 34.7, 22.8.

HRMS (ESI^+) $[M+Na]^+$ Calcd for $C_{26}H_{28}O_4Na$:427.1880 m/z, Found: 427.1874 m/z; **Specific rotation:** $[\alpha]_D^{25.2}$-24.7 (*c* 0.50, $CHCl_3$) for an enantiomerically enriched sample of >99.5:0.5 e.r.

Enantiomeric purity of **15** was determined by HPLC analysis in comparison with authentic racemic material (>99.5:0.5 e.r. shown; Chiralpak IE column, 85:15 hexane / ^{i}PrOH, 1.0 mL/min, 254 nm).

<Sample Information>

Sample Name : CYL-11008RAC
Sample ID :
Data Filename : CYL-11008RAC-IE-85-15-1.0.lcd
Method Filename : ZJW-3-8515-1.0-120-1min.lcm
Batch Filename : ZJW.lcb
Vial # : 1-91
Injection Volume : 10 uL
Date Acquired : 7/10/2024 9:06:47 AM
Date Processed : 7/10/2024 9:47:57 AM
Sample Type : Unknown
Acquired by : System Administrator
Processed by : System Administrator

<Chromatogram>

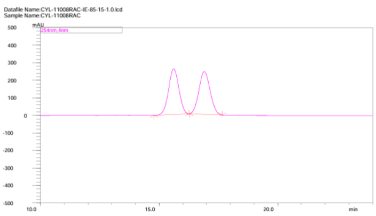

<Peak Table>

PDA Ch1 254nm

Peak#	Ret. Time	Area	Area%
1	15.612	7888335	49.571
2	16.903	8024955	50.429
Total		15913290	100.000

<Sample Information>

Sample Name : CYL-11006
Sample ID :
Data Filename : CYL-11006-IE-85-15-1.0.lcd
Method Filename : ZJW-3-8515-1.0-120-1min.lcm
Batch Filename : ZJW.lcb
Vial # : 1-92
Injection Volume : 10 uL
Date Acquired : 7/10/2024 9:49:22 AM
Date Processed : 7/10/2024 10:21:03 AM
Sample Type : Unknown
Acquired by : System Administrator
Processed by : System Administrator

<Chromatogram>

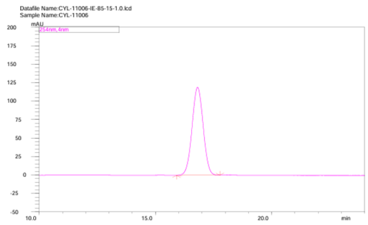

<Peak Table>

PDA Ch1 254nm

Peak#	Ret. Time	Area	Area%
1	16.811	3959944	100.000
Total		3959944	100.000

■ Mechanistic Studies

Deuterated experiments

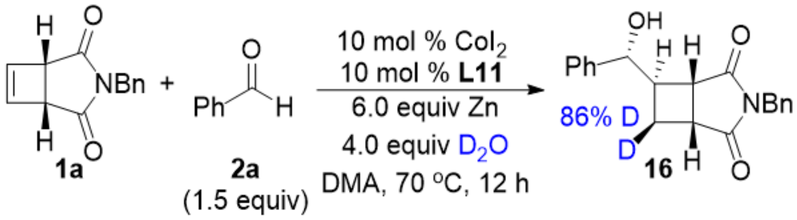

In a N_2-filled glove-box, an oven-dried vial (8 mL) equipped with a magnetic stir bar was charged with CoI_2 (0.02 mmol, 10 mol %), **L11** (0.02 mmol, 10 mol %) and Zn (1.2 mmol, 6.0 equiv.). DMA (2 mL) was added, then the mixture was allowed to stir at room temperature for 30 min. **1a** (42.6 mg, 0.2 mmol, 1.0 equiv.), **2a** (31.8 mg, 0.3 mmol, 1.5 equiv.) and D_2O (16.0 mg, 0.8 mmol, 4.0 equiv.) were added to the solution. The vial was sealed with a cap (phenolic open top cap with red PTFE/white silicone septum), twined with adhesive tape and taken out of the glove box. It was allowed to stir at 70 °C for 12 h.

Workup: Upon completion, the reaction was quenched by 50 mL H_2O and extracted with diethyl ether (3*20 mL). The combined organic layer was washed with brine (10 mL) and dried over Na_2SO_4. After the solids were filtered off, the solvent was removed under reduced pressure and the residue was purified by silica-gel column chromatography (eluent: Petroleum ether/ EtOAc = 3:1) to afford **16** (51.2 mg, 80% yield, >20:1 dr, 99.5:0.5 er).

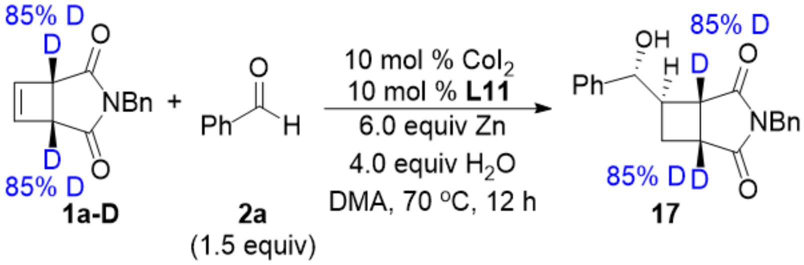

In a N_2-filled glove-box, an oven-dried vial (8 mL) equipped with a magnetic stir bar was charged with CoI_2 (0.02 mmol, 10 mol %), **L11** (0.02 mmol, 10 mol %) and Zn (1.2 mmol, 6.0 equiv.). DMA (2 mL) was added, then the mixture was allowed to stir at room temperature for 30 min. **1a-D** (42.6 mg, 0.2 mmol, 1.0 equiv.), **2a** (31.8 mg, 0.3 mmol, 1.5 equiv.) and H_2O (14.4 mg, 0.8 mmol, 4.0 equiv.) were added to the solution. The vial was sealed with a cap (phenolic open top cap with red PTFE/white silicone septum), twined with adhesive tape and taken out of the glove box. It was allowed to stir at 70 °C for 12 h.

Workup: Upon completion, the reaction was quenched by 50 mL H_2O and extracted with diethyl ether (3*20 mL). The combined organic layer was washed with brine (10 mL) and dried over Na_2SO_4. After the solids were filtered off, the solvent was removed under reduced pressure and the residue was purified by silica-gel column chromatography (eluent: Petroleum ether/ EtOAc = 3:1) to afford **17** (52.4 mg, 81% yield, >20:1 dr, >99.5:0.5 er).

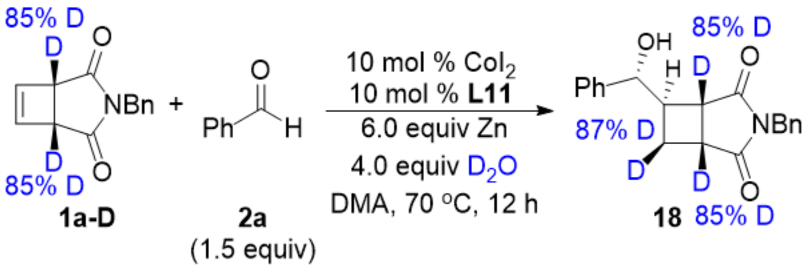

In a N_2-filled glove-box, an oven-dried vial (8 mL) equipped with a magnetic stir bar was charged with CoI_2 (0.02 mmol, 10 mol %), **L11** (0.02 mmol, 10 mol %) and Zn (1.2 mmol, 6.0 equiv.). DMA (2 mL) was added, then the mixture was allowed to stir at room temperature for 30 min. **1a-D** (42.6 mg, 0.2 mmol, 1.0 equiv.), **2a** (31.8 mg, 0.3 mmol, 1.5 equiv.) and D_2O (16.0 mg, 0.8 mmol, 4.0 equiv.) were added to the solution. The vial was sealed with a cap (phenolic open top cap with red PTFE/white silicone septum), twined with adhesive tape and taken out of the glove box. It was allowed to stir at 70 °C for 12 h.

Workup: Upon completion, the reaction was quenched by 50 mL H_2O and extracted with diethyl ether (3*20 mL). The combined organic layer was washed with brine (10 mL) and dried over Na_2SO_4. After the solids were filtered off, the solvent was removed under reduced pressure and the residue was purified by silica-gel column chromatography (eluent: Petroleum ether/ EtOAc = 3:1) to afford **18** (50.7 mg, 78% yield, >20:1 dr, >99.5:0.5 er).

Characterization for Deuterated experiments

(1*R*,5*S*,6*R*,7*S*)-3-benzyl-6-((*R*)-hydroxy(phenyl)methyl)-3-azabicyclo[3.2.0]heptane-2,4-dione-7-d (16)

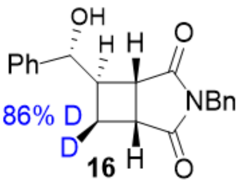

16

yellow oil; **IR (neat):** 3505.62 (br), 1759.61 (w), 1684.34 (s), 1493.60 (w), 1452.83 (w), 1433.38 (w), 1400.19 (m), 1363.01 (w), 1334.81 (m), 1301.56 (w), 1259.44 (w), 1194.98 (w), 1175.32 (w), 1161.82 (w), 1087.74 (w), 1068.81 (w), 1026.04 (w), 997.75 (w), 967.69 (w), 929.31 (w), 882.66 (w), 820.58 (w), 754.62 (m), 725.67 (m), 708.78 (m), 691.37 (w), 637.13 (w), 602.06 (w), 482.33 (w), 458.37 (w), 445.91 (w) cm^{-1}

^{1}H NMR (400 MHz, $CDCl_3$) δ 7.30-7.15 (m, 10 H), 4.59 (d, *J* = 8.0 Hz, 1 H), 4.56 (s, 2 H), 3.29 (dd, *J* = 6.8, 4.8 Hz, 1 H), 3.07-3.04 (m, 1 H), 2.96 (br, 1 H), 2.59-2.54 (m, 1 H), 2.36-2.28 (m, 0.14 H), 1.95-1.92 (m, 1 H). **^{13}C NMR (100 MHz, $CDCl_3$)** δ 179.5, 178.9, 141.3, 136.0, 128.8, 128.7, 128.3, 128.0, 126.4, 76.1, 43.6, 42.6, 41.4, 35.6, 25.53-25.01 (m). **^{2}H NMR (61 MHz, $CHCl_3$)** δ 2.33.
HRMS (ESI$^+$) [M+Na]$^+$ Calcd for $C_{20}H_{18}NO_3NaD$: 345.1320 m/z, Found: 345.1327 m/z;
Specific rotation: $[\alpha]_D^{28.7}$ -1.6 (*c* 0.50, $CHCl_3$) for an enantiomerically enriched sample of 99.5:0.5 e.r.

Enantiomeric purity of **16** was determined by HPLC analysis in comparison with authentic racemic material (99.5:0.5 e.r. shown; Chiralpak IF column, 90:10 hexane / ^{i}PrOH, 1.0 mL/min, 220 nm).

<Sample Information>

Sample Name : cyl-10063rac
Sample ID :
Data Filename : cyl-10063-RAC-IF-90-10-1.0.lcd
Method Filename : cyl-6H-90-10-1.0ml-50min.lcm
Batch Filename : WWLL.lcb
Vial # : 1-91 Sample Type : Unknown
Injection Volume : 20 uL
Date Acquired : 7/24/2024 22:52:09 Acquired by : System Administrator
Date Processed : 7/27/2024 10:03:39 Processed by : System Administrator

<Chromatogram>

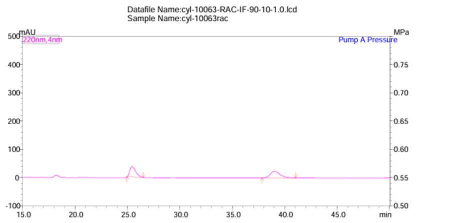

<Peak Table>

PDA Ch2 220nm

Peak#	Ret. Time	Area	Area%
1	25.432	1449431	49.951
2	39.006	1452268	50.049
Total		2901699	100.000

<Sample Information>

Sample Name : cyl-10063
Sample ID :
Data Filename : cyl-10063-IF-90-10-1.0.lcd
Method Filename : cyl-6H-90-10-1.0ml-50min.lcm
Batch Filename : WWLL.lcb
Vial # : 1-92 Sample Type : Unknown
Injection Volume : 20 uL
Date Acquired : 7/24/2024 23:42:42 Acquired by : System Administrator
Date Processed : 7/25/2024 7:44:47 Processed by : System Administrator

<Chromatogram>

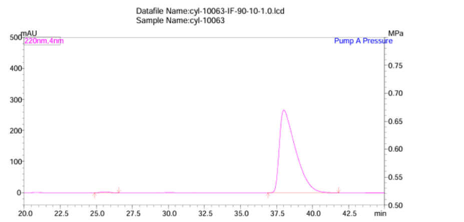

<Peak Table>

PDA Ch2 220nm

Peak#	Ret. Time	Area	Area%
1	25.570	99422	0.475
2	38.014	20812458	99.525
Total		20911880	100.000

(1*R*,5*S*,6*R*)-3-benzyl-6-((*R*)-hydroxy(phenyl)methyl)-3-azabicyclo[3.2.0]heptane-2,4-dione-1,5-d2 (17)

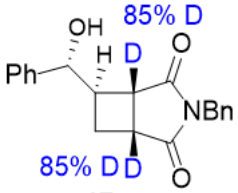

17

yellow oil; **IR (neat):**3505.13 (br), 1759.13 (w), 1684.58 (s), 1493.92 (w), 1452.49 (w), 1432.93 (w), 1397.95 (m), 1362.41 (w), 1334.88 (m), 1259.87 (w), 1216.34 (w), 1201.00 (w), 1150.90 (w), 1136.34 (w), 1085.64 (w), 1013.71 (w), 987.38 (m), 911.29 (w), 864.35 (w), 811.40 (w), 751.76 (w), 723.37 (m), 690.68 (m), 633.66 (m), 603.58 (w), 574.86 (m), 502.36 (w), 478.2 (w), 457.50 (w), 442.79 (w), 409.94 (w) cm^{-1}

^{1}H NMR (400 MHz, $CDCl_3$) δ 7.30-7.15 (m, 10 H), 4.59 (d, J = 8.0 Hz, 1 H), 4.56 (s, 2 H), 3.30 (d, J = 4.8 Hz, 0.15 H), 3.06 (dd, J = 9.2, 6.0 Hz, 0.15 H), 2.93 (br, 1 H), 2.59-2.54 (m, 1 H), 2.35-2.28 (m, 1 H), 1.98-1.92 (m, 1 H). **^{13}C NMR (100 MHz, $CDCl_3$)** δ 179.5, 178.9, 141.3, 136.0, 128.8, 128.7, 128.3, 128.0, 126.4, 76.1, 43.6, 42.6, 41.3, 35.6, 25.4. **^{2}H NMR (61 MHz, $CHCl_3$)** δ 3.28, 3.06.
HRMS (ESI$^+$) [M+Na]$^+$ Calcd for $C_{20}H_{17}NO_3NaD_2$: 346.1383 m/z, Found: 346.1390 m/z;
Specific rotation: $[\alpha]_D^{28.9}$3.1 (c 0.50, $CHCl_3$) for an enantiomerically enriched sample of >99.5:0.5 e.r.
Enantiomeric purity of **17** was determined by HPLC analysis in comparison with authentic racemic material (>99.5:0.5 e.r. shown; Chiralpak IF column, 90:10 hexane / ^{i}PrOH, 1.0 mL/min, 220 nm).

<Sample Information>

Sample Name : cyl-10064rac
Sample ID :
Data Filename : cyl-10064-RAC-IF-90-10-1.0.lcd
Method Filename : cyl-6H-90-10-1.0ml-50min.lcm
Batch Filename : WWLL.lcb
Vial # : 1-91 Sample Type : Unknown
Injection Volume : 20 uL
Date Acquired : 7/25/2024 0:33:22 Acquired by : System Administrator
Date Processed : 7/25/2024 1:23:24 Processed by : System Administrator

<Chromatogram>

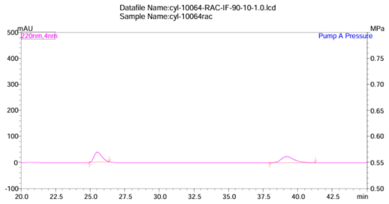

<Peak Table>

PDA Ch2 220nm

Peak#	Ret. Time	Area	Area%
1	25.520	1483524	50.411
2	39.194	1459305	49.589
Total		2942829	100.000

<Sample Information>

Sample Name : cyl-10064
Sample ID :
Data Filename : cyl-10064-IF-90-10-1.0.lcd
Method Filename : cyl-6H-90-10-1.0ml-50min.lcm
Batch Filename : WWLL.lcb
Vial # : 1-93 Sample Type : Unknown
Injection Volume : 20 uL
Date Acquired : 7/25/2024 1:23:55 Acquired by : System Administrator
Date Processed : 7/25/2024 2:13:57 Processed by : System Administrator

<Chromatogram>

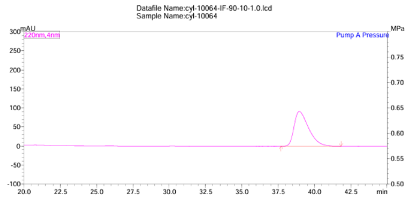

<Peak Table>

PDA Ch2 220nm

Peak#	Ret. Time	Area	Area%
1	38.966	6332725	100.000
Total		6332725	100.000

(1*R*,5*S*,6*R*,7*S*)-3-benzyl-6-((*R*)-hydroxy(phenyl)methyl)-3-azabicyclo[3.2.0]heptane-2,4-dione-1,5,7-d3 (18)

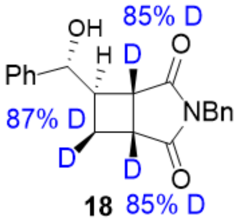

18

yellow oil; **IR (neat):** 3505.13 (br), 1759.70 (w), 1682.93 (s), 1493.66 (w), 1452.56 (w), 1398.36 (m), 1362.26 (w), 1334.62 (m), 1260.76 (w), 1204.63 (w), 1183.32 (w), 1121.20 (m), 1093.32 (w), 1071.27 (w), 1015.65 (m), 988.95 (m), 807.69 (w), 752.27 (w), 720.81 (m), 690.85 (m), 633.17 (w), 596.35 (m), 570.53 (m), 456.90 (w), 441.42 (w) cm^{-1}

^{1}H NMR (400 MHz, $CDCl_3$) δ 7.28-7.14 (m, 10 H), 4.59 (d, *J* = 8.0 Hz, 1 H), 4.55 (s, 2 H), 3.30 (d, *J* = 4.8 Hz, 0.15 H), 3.08 (br, 1 H), 3.04 (d, *J* = 4.4 Hz, 0.15 H), 2.57-2.53 (m, 1 H), 2.33-2.28 (m, 0.13 H), 1.97-1.91 (m, 1 H). **^{13}C NMR (100 MHz, $CDCl_3$)** δ 179.5, 178.9, 141.4, 136.0, 128.7, 128.7, 128.3, 128.0, 126.4, 76.0, 43.4, 42.6, 41.3, 35.5, 25.38-25.07 (m). **^{2}H NMR (61 MHz, $CHCl_3$)** δ 3.29, 3.06, 2.39.

HRMS (ESI$^+$) [M+Na]$^+$ Calcd for $C_{20}H_{16}NO_3NaD_3$: 347.1445 m/z, Found: 347.1444 m/z;

Specific rotation: $[\alpha]_D^{29.0}$1.3 (*c* 0.50, $CHCl_3$) for an enantiomerically enriched sample of >99.5:0.5 e.r.

Enantiomeric purity of **18** was determined by HPLC analysis in comparison with authentic racemic material (>99.5:0.5 e.r. shown; Chiralpak IF column, 90:10 hexane / ^{i}PrOH, 1.0 mL/min, 220 nm).

<Sample Information>

Sample Name : cyl-10064rac
Sample ID :
Data Filename : cyl-10065-RAC-IF-90-10-1.0.lcd
Method Filename : cyl-6H-90-10-1.0ml-50min.lcm
Batch Filename : WWLL.lcb
Vial # : 1-91
Injection Volume : 20 uL
Date Acquired : 7/25/2024 2:14:41
Date Processed : 7/25/2024 3:04:43
Sample Type : Unknown
Acquired by : System Administrator
Processed by : System Administrator

<Chromatogram>

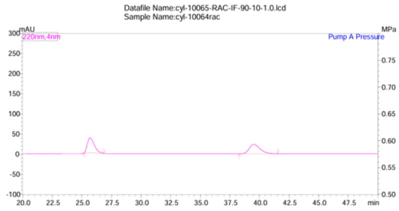

<Peak Table>

PDA Ch2 220nm

Peak#	Ret. Time	Area	Area%
1	25.692	1486118	50.278
2	39.519	1469655	49.722
Total		2955773	100.000

<Sample Information>

Sample Name : cyl-10065
Sample ID :
Data Filename : cyl-10065NEW-IF-90-10-1.0.lcd
Method Filename : cyl-3hao-90-10-1.0ml-60min.lcm
Batch Filename : WWLL.lcb
Vial # : 1-65
Injection Volume : 10 uL
Date Acquired : 9/10/2024 10:47:26
Date Processed : 9/10/2024 11:47:29
Sample Type : Unknown
Acquired by : System Administrator
Processed by : System Administrator

<Chromatogram>

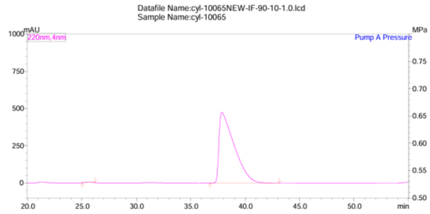

<Peak Table>

PDA Ch2 220nm

Peak#	Ret. Time	Area	Area%
1	25.669	155183	0.334
2	37.857	46303998	99.666
Total		46459181	100.000

Parallel KIE experiments

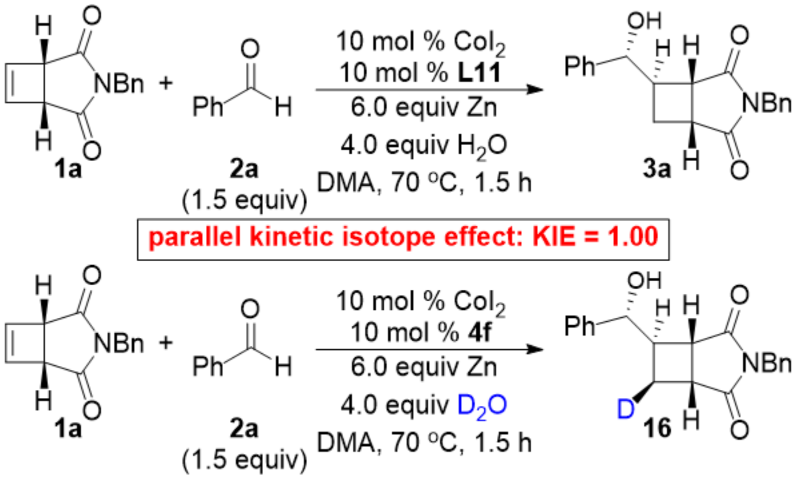

In a N_2-filled glove-box, an oven-dried vial (8 mL) equipped with a magnetic stir bar was charged with CoI_2 (0.02 mmol, 10 mol %), **L11** (0.02 mmol, 10 mol %) and Zn (1.2 mmol, 6.0 equiv.). DMA (2 mL) was added, then the mixture was allowed to stir at room temperature for 30 min. **1a** (42.6 mg, 0.2 mmol, 1.0 equiv.), **2a** (31.8 mg, 0.3 mmol, 1.5 equiv.) and D_2O (16.0 mg, 0.8 mmol, 4.0 equiv.) or H_2O (14.4 mg, 0.8 mmol, 4.0 equiv.) were added to the solution. The vial was sealed with a cap (phenolic open top cap with red PTFE/white silicone septum), twined with adhesive tape and taken out of the glove box. It was allowed to stir at 70 °C for 1.5 h.

Workup: The reaction was quenched by 50 mL H_2O and extracted with diethyl ether (3*20 mL). The combined organic layer was washed with brine (10 mL) and dried over Na_2SO_4. After the solids were filtered off, the solvent was removed under reduced pressure and 7 μL CH_2Br_2 was added. We calculated KIE value according to the analysis of crude ^{1}H NMR. We repeated the experiment for three times. The NMR yield of **16** is 22%, 21%, 24%, respectively. The NMR yield of **3a** is 23%, 21%, 24%, respectively. The KIE value is 1.05, 1.00, 1.00, respectively.

Competitive KIE experiments

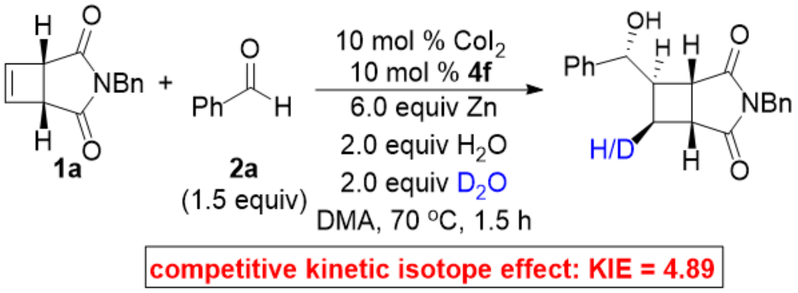

In a N_2-filled glove-box, an oven-dried vial (8 mL) equipped with a magnetic stir bar was charged with CoI_2 (0.02 mmol, 10 mol %), **L11** (0.02 mmol, 10 mol %) and Zn (1.2 mmol, 6.0 equiv.). DMA (2 mL) was added, then the mixture was allowed to stir at room temperature for 30 min. **1a** (42.6 mg, 0.2 mmol, 1.0 equiv.), **2a** (31.8 mg, 0.3 mmol, 1.5 equiv.) and D_2O (0.4 mmol, 2.0 equiv.), H_2O (0.4 mmol, 2.0 equiv.) were added to the solution. The vial was sealed with a cap (phenolic open top cap with red PTFE/white silicone septum), twined with adhesive tape and taken out of the glove box. It was allowed to stir at 70 °C for 1.5 h.

Workup: The reaction was quenched by 50 mL H_2O and extracted with diethyl ether (3*20 mL). The combined organic layer was washed with brine (10 mL) and dried over Na_2SO_4. After the solids were filtered off, the solvent was removed under reduced pressure and 7 μL CH_2Br_2 was added. We calculated KIE value according to the analysis of crude ^1H NMR. We repeated the experiment for three times. The total ^1H NMR yield of **3a** and **3a-D** is 20%, 21%, 23%, respectively. The KIE value is 5.67, 4.25, 4.75, respectively.

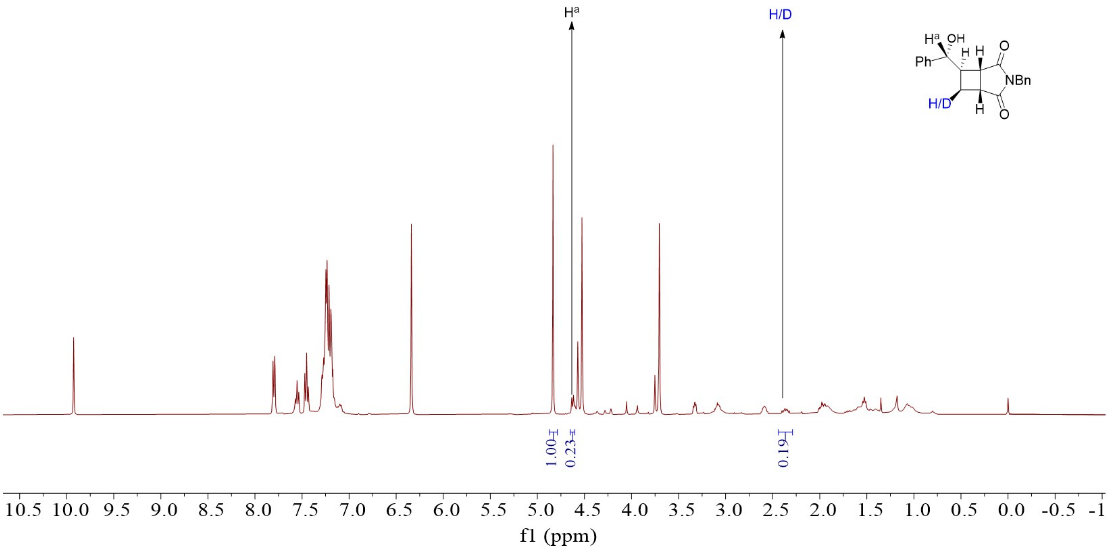

DFT Studies

Computational details

All of the calculations were performed using the Gaussian 16 program[8]. Structures were optimized at the (U)B3LYP level of density functional theory[9] with Grimme's D3(BJ) dispersion correction[10] in gas phase. For optimizations, Ahlrichs's def2SVP basis set was used for all atoms. [11] Frequency calculations have been performed to verify the optimized structures as local minima or transition state and to obtain Gibbs free energy at 298 K. To reduce error caused by the breakdown of the harmonic oscillator approximation, Truhlar's quasi-harmonic correction was used to compute molecular entropies by setting all positive frequencies that are less than 100 cm^{-1} to 100 cm^{-1}. [12] Intrinsic reaction coordinate (IRC) calculations were carried out to make sure that every transition state links relevant intermediates. [13] The electronic energies were further refined by carrying out single-point energy calculations using (U)TPSSh functional[14,15] with Grimme's D3(BJ) dispersion correction, which was reported suitable for calculating first-row transition metal complexes involving multiple spin states[16-18]. The def2TZVP basis set was applied for all atoms. [11] The SMD solvation model with THF as the solvent was employed to account for solvation effect. [19] The three-dimensional (3D) structures were depicted using CYLview software.

Additional Computational Results

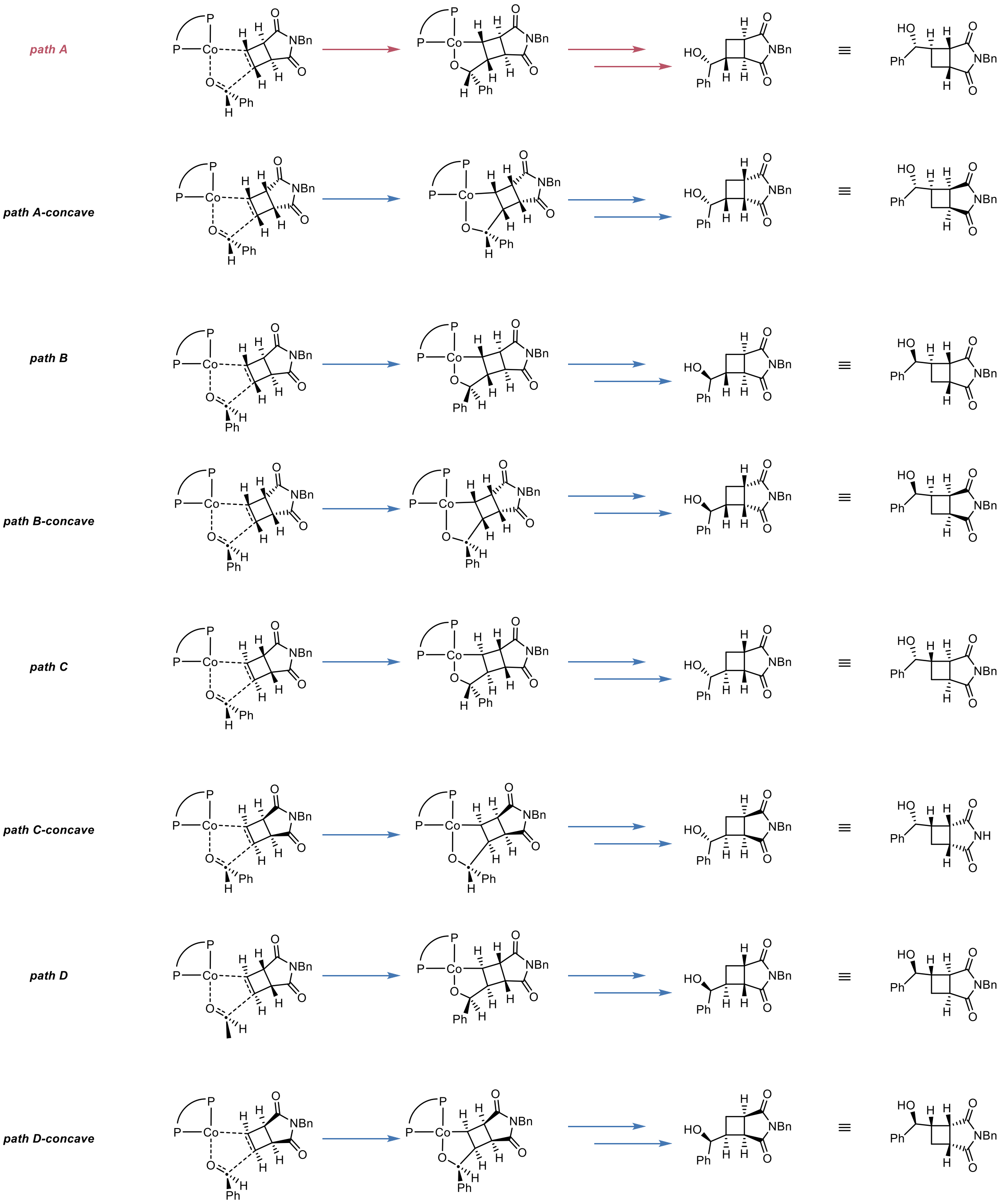

Figure S1. All plausible diastereomeric transition states leading to different diastereomers.

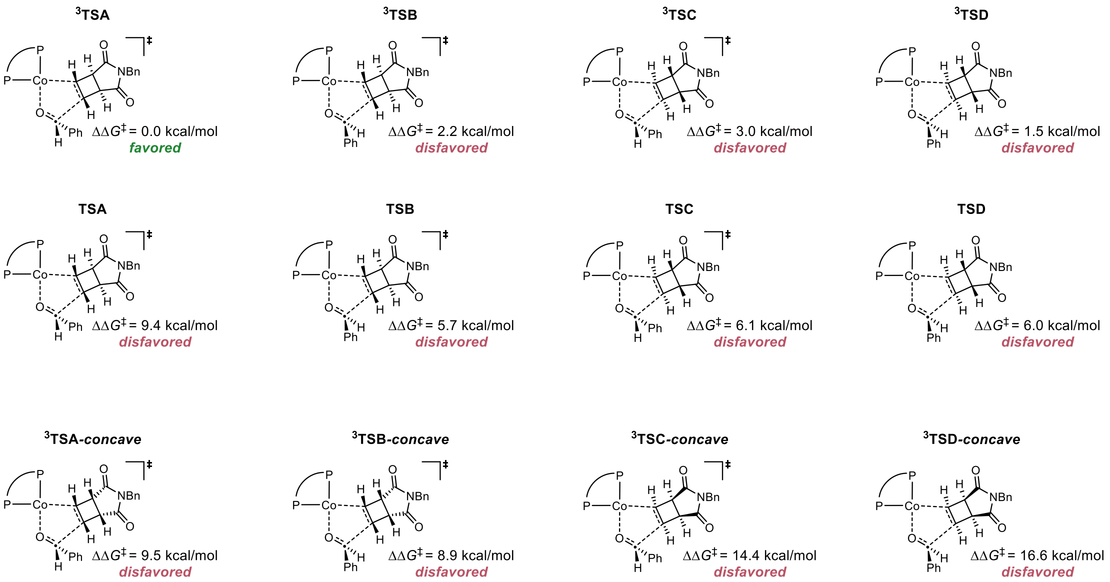

Figure S2. Calculated energy difference for all oxidative cyclization transition states for both singlet state and triplet state. All energies are given in kcal/mol.

XYZ coordinate

[3]TSA

E = -5781.78228903 G = -5780.817825

26	4.61836000	-1.20683100	-0.05682700
15	1.12758300	1.91306000	-0.88292700
15	1.16382700	-1.35741600	0.66138100
6	5.68123400	-0.16329000	1.38850900
6	4.99638900	-1.26133100	1.98641800
6	5.45309900	-2.46027200	1.36142600
6	6.42551700	-2.10227300	0.37903000
6	6.56512700	-0.68167400	0.39312700
6	2.60023600	-1.21398900	-0.42981600
6	3.24890000	-2.28480300	-1.13863500
6	4.20883700	-1.71743100	-2.02083300
6	4.17932400	-0.30090000	-1.86022800
6	3.19834800	0.03180300	-0.87407100
6	2.87435000	1.42530400	-0.39025400
6	3.96634000	2.44944900	-0.71441100
6	1.69038600	-1.99403400	2.28313600
6	2.18160400	-3.30128400	2.43002400
6	2.63365000	-3.73768700	3.67471300
6	2.59366800	-2.87573700	4.77813600
6	2.09279600	-1.57947700	4.63698000
6	1.63747900	-1.13435300	3.39114700
6	0.10596200	-2.66303500	-0.04962800
6	-0.87803700	-3.23059300	0.78084500
6	-1.86613000	-4.05316200	0.23876000
6	-1.88098700	-4.32229300	-1.13350400
6	-0.90208100	-3.76861200	-1.96093000
6	0.08578200	-2.93792800	-1.42588400
1	5.54984400	0.88582200	1.64759100
1	4.24793500	-1.19691200	2.77076100
1	5.10978900	-3.46683400	1.58843600
1	6.95005700	-2.78879100	-0.28305600
1	7.21774800	-0.09771000	-0.25348500
1	3.03090700	-3.34378800	-1.02161300
1	4.87861600	-2.26981000	-2.67687200
1	4.83085400	0.40253300	-2.37248700
1	2.77971500	1.38053000	0.70949500
1	4.05405300	2.63949700	-1.79135400
1	4.93426700	2.06954700	-0.35815300
1	3.77702900	3.40651400	-0.21115600
1	2.20999900	-3.97548800	1.57134300
1	3.01829900	-4.75365700	3.78882500
1	2.05331100	-0.90975300	5.49879800
1	1.24659500	-0.11966400	3.27676600
1	-0.87375100	-3.02116500	1.85342500
1	-2.63564900	-4.48094800	0.88448900
1	-0.91047700	-3.97518700	-3.03330300
1	0.83012900	-2.49402900	-2.08538700
27	-0.02088000	0.67963100	0.63417400
8	-3.40726400	0.14265000	-3.01044900
8	-5.41678000	0.42983200	1.09269200
7	-4.67621400	0.19183200	-1.07869400
6	-3.54635300	0.44365600	-1.84860600
6	-1.35208700	0.83343700	2.56779100
6	-2.19026400	0.22829500	1.05349600
1	-2.59876500	-0.70176800	1.46491000
6	-3.19788500	1.23996700	0.43434100
1	-3.26799200	2.21908000	0.92391000
6	-4.57028100	0.60493600	0.25055300
6	-5.83056300	-0.54918200	-1.58588400
1	-5.95552400	-0.26727100	-2.63903900
1	-6.70064400	-0.20108200	-1.01343600
6	-5.66565800	-2.04733900	-1.45064800
6	-5.85288700	-2.66970200	-0.20629500
1	-6.11494700	-2.06054800	0.66193900
6	-5.72528100	-4.05526400	-0.08549700
1	-5.88966100	-4.53354600	0.88317100
6	-5.41931800	-4.83262800	-1.20745000
1	-5.34382900	-5.91902000	-1.11675200
6	-5.21885700	-4.21636100	-2.44580000
1	-4.98039600	-4.81873500	-3.32573100
6	-5.33176000	-2.82854700	-2.56454500
1	-5.16941900	-2.34438700	-3.53031700
6	-2.52263800	1.10996800	-0.94458400
1	-2.09271700	1.99454900	-1.42844200
6	-1.57103300	0.08953600	-0.32785100
1	-1.42422800	-0.89125300	-0.78676800
6	0.82810500	1.70230200	-2.70079500
6	1.95299600	2.20536600	-3.62602900
6	0.45904000	0.25345900	-3.06862800
1	-0.06628600	2.33192500	-2.86817200
6	1.52109500	2.11166800	-5.09547100
1	2.84218000	1.57375600	-3.47037700
1	2.24172600	3.23600000	-3.38504800
6	0.03289400	0.14828300	-4.53520200
1	1.33560700	-0.38932500	-2.88730900
1	-0.34885600	-0.11371800	-2.42745000

6	1.11266500	0.68648500	-5.47654000
1	2.34016000	2.46644100	-5.74137300
1	0.67092900	2.79685700	-5.26425700
1	-0.20150500	-0.90204200	-4.77165800
1	-0.90928000	0.70740800	-4.66978600
1	0.76363900	0.65539800	-6.52070900
1	2.00086300	0.03087700	-5.42285300
6	0.89732900	3.69783400	-0.40326100
6	1.43155300	4.76443800	-1.37449400
6	-0.58088800	3.98004000	-0.07152900
1	1.46221100	3.76322400	0.54520500
6	1.25471800	6.17191800	-0.78820000
1	0.87107400	4.69773200	-2.32250100
1	2.48828200	4.59102100	-1.61731400
6	-0.76159000	5.38449000	0.51137400
1	-1.18249300	3.89113700	-0.99360100
1	-0.95641000	3.23177100	0.63917500
6	-0.20490500	6.45826800	-0.42645600
1	1.62877000	6.91819100	-1.50693600
1	1.88322700	6.26591300	0.11562400
1	-1.82807000	5.56169800	0.72330900
1	-0.23787600	5.43467200	1.48291700
1	-0.29713100	7.45502000	0.03269300
1	-0.81017300	6.48288200	-1.35068600
8	-0.15976400	1.42867400	2.32997800
6	-2.38781600	1.72193300	3.19787400
6	-3.59295400	1.18335400	3.66722400
6	-2.16392600	3.10151400	3.29407400
6	-4.57000600	2.01550700	4.21242900
1	-3.78394400	0.11075600	3.58697100
6	-3.14128700	3.93388800	3.84521900
1	-1.21116800	3.50552700	2.95246800
6	-4.34767500	3.39361500	4.29985300
1	-5.51149500	1.58782600	4.56244000
1	-2.95774000	5.00793300	3.92501800
1	-5.11383900	4.04479600	4.72642300
1	-2.66416800	-4.95221200	-1.55411300
1	2.94990800	-3.22106200	5.75118600
1	-1.25774700	-0.12153300	3.11818200

TSA

E = -5781.77204464 G = -5780.802922

26	-4.11705300	-2.16485900	0.18040400
15	-1.45828200	1.60529800	1.00267600
15	-0.88836600	-1.07882000	-0.84222900
6	-5.82079200	-1.25266400	-0.56629900
6	-4.97003900	-1.64839500	-1.63878600
6	-4.79248600	-3.06444000	-1.55966200
6	-5.53482000	-3.54020700	-0.43887500
6	-6.16742300	-2.42064200	0.17910800
6	-2.12419900	-1.63131000	0.34248900
6	-2.33242700	-2.97348400	0.81831400
6	-3.25022000	-2.91726500	1.90122700
6	-3.63813700	-1.55948700	2.08696500
6	-2.96501300	-0.74898300	1.11724500
6	-3.12285200	0.74704000	0.94957900
6	-4.18953100	1.28528400	1.91075700
6	-1.69837300	-0.92938000	-2.47927400
6	-2.04429600	-2.09472400	-3.18532200
6	-2.71906200	-2.00170500	-4.40134800
6	-3.06358700	-0.74785400	-4.91974200
6	-2.72036600	0.41164300	-4.22162100
6	-2.03389100	0.32621600	-3.00575300
6	0.32581100	-2.42426600	-1.03418300
6	0.98782200	-2.58156300	-2.26446000
6	2.05790000	-3.47114300	-2.37953400
6	2.48915900	-4.19850300	-1.26792200
6	1.84573700	-4.03742500	-0.03754400
6	0.77221000	-3.15505100	0.08211700
1	-6.13934600	-0.23520300	-0.34584100
1	-4.52742500	-0.98795400	-2.37941300
1	-4.19530500	-3.67410700	-2.23227700
1	-5.58584300	-4.57204600	-0.09598200
1	-6.78542300	-2.44936600	1.07474000
1	-1.86396400	-3.86872100	0.41877900
1	-3.62616000	-3.76968500	2.46348300
1	-4.36089300	-1.21316100	2.81997200
1	-3.44464900	0.94737900	-0.08718100
1	-3.94337300	1.04913300	2.95288300
1	-5.15309200	0.80740400	1.68085400
1	-4.32262100	2.36664600	1.83407200
1	-1.78726000	-3.07544400	-2.78239800
1	-2.98256200	-2.91076600	-4.94639000
1	-2.97612800	1.39263900	-4.62772200
1	-1.73243100	1.23876500	-2.48920200
1	0.67028000	-2.00819700	-3.13667800
1	2.56092300	-3.58891800	-3.34166900

1	2.19984800	-4.58495000	0.83674700
1	0.29745300	-3.01835600	1.05477500
27	0.04283500	0.83827600	-0.42877400
8	3.39110600	0.00863200	3.10916700
8	5.29217900	0.33495600	-1.04113700
7	4.60667300	0.06959000	1.14783600
6	3.50107300	0.33523800	1.94960700
6	1.21032900	1.64310900	-2.27508800
6	2.11673000	0.46524700	-1.03551500
1	2.44439500	-0.31589100	-1.72736000
6	3.17148700	1.29171700	-0.27008800
1	3.35065900	2.32336800	-0.60195000
6	4.48950000	0.53389800	-0.16269800
6	5.74115200	-0.73038900	1.60606800
1	5.88522800	-0.49649300	2.66896300
1	6.61738700	-0.39041300	1.03944500
6	5.52240800	-2.21500100	1.41149700
6	5.95256100	-2.84582200	0.23554900
1	6.43242800	-2.25144800	-0.54541300
6	5.77828800	-4.22193800	0.06942400
1	6.13762600	-4.70817300	-0.84085800
6	5.16372600	-4.97789900	1.07236600
1	5.04352400	-6.05715700	0.94966700
6	4.71179800	-4.35005900	2.23739900
1	4.23572000	-4.93735400	3.02651300
6	4.88851700	-2.97439000	2.40621600
1	4.54326500	-2.48005800	3.31712400
6	2.47927200	1.03537900	1.07810200
1	2.01210600	1.88137200	1.59869700
6	1.55049500	0.04305400	0.34539900
1	1.61579700	-1.00562800	0.64099500
6	-0.76272000	1.52167400	2.72219300
6	-1.45988500	2.37437100	3.79759800
6	-0.57353700	0.07225900	3.20742800
1	0.24374900	1.94951900	2.58159000
6	-0.64906600	2.34158900	5.10009700
1	-2.46920100	1.98114200	3.99102200
1	-1.58554600	3.41217700	3.45806000
6	0.22198600	0.03835800	4.51687600
1	-1.56034800	-0.39052000	3.36363700
1	-0.06573800	-0.52399900	2.43589600
6	-0.43227400	0.90822100	5.59343500
1	-1.16071700	2.94387700	5.86782600
1	0.33040000	2.82231300	4.92564600
1	0.30627800	-1.00388300	4.86486100
1	1.25208600	0.37863700	4.32063300
1	0.18094700	0.90965200	6.50838400
1	-1.40937300	0.47304500	5.87314000
6	-1.63139600	3.39413600	0.49013800
6	-2.87182600	4.20552400	0.89722500
6	-0.32755800	4.13890700	0.84911600
1	-1.64413300	3.30353600	-0.60905200
6	-2.81876300	5.61823400	0.29691400
1	-2.94709800	4.27425200	1.99479000
1	-3.78444200	3.70759200	0.54067600
6	-0.30359600	5.53861800	0.22878800
1	-0.23969400	4.23294400	1.94397400
1	0.54268100	3.56426300	0.49873900
6	-1.52616500	6.35321700	0.65596300
1	-3.69834200	6.19012900	0.63294900
1	-2.89722300	5.54088500	-0.80239600
1	0.62893000	6.05262300	0.51198500
1	-0.29352400	5.44346700	-0.87119000
1	-1.50975400	7.34867000	0.18499300
1	-1.48974700	6.52263300	1.74742200
8	0.13467400	2.21986800	-1.69918900
6	2.31208100	2.55931800	-2.69463800
6	3.39481200	2.06574400	-3.43652100
6	2.28089900	3.91274900	-2.33285800
6	4.44003000	2.91263400	-3.80148000
1	3.43313200	1.00913500	-3.71226700
6	3.32601200	4.76155000	-2.70533800
1	1.42667500	4.28825700	-1.77031100
6	4.40830300	4.26288400	-3.43571900
1	5.28522000	2.51818300	-4.36903000
1	3.29393700	5.81739500	-2.42688500
1	5.22717700	4.92593500	-3.72339000
1	3.33610000	-4.88021300	-1.35101800
1	-3.59505000	-0.67786200	-5.87125300
1	0.96385600	0.91700200	-3.07228000

TSB

E = -5781.77914164 G = -5780.808801

26	2.30896900	-2.07314000	-2.83485300
15	1.92091900	1.62850600	0.06728200
15	0.30830500	-1.24178800	-0.02656500
6	4.32658600	-2.54459400	-2.73631500

6	3.61173100	-3.23346000	-1.71459000
6	2.63074500	-4.05876100	-2.34674200
6	2.74214800	-3.87636500	-3.75653700
6	3.78704900	-2.93588600	-3.99869000
6	0.88461800	-1.08427500	-1.72020300
6	0.31132800	-1.60635500	-2.93241500
6	0.99580000	-1.01295700	-4.02875000
6	2.00408300	-0.14913900	-3.51305900
6	1.95816700	-0.18811600	-2.08376800
6	2.88002500	0.54667000	-1.13589900
6	4.00942800	1.24508300	-1.90184200
6	1.47901800	-2.33068700	0.86432500
6	1.37536500	-3.72652400	0.76034000
6	2.29541800	-4.54793800	1.41198600
6	3.33342000	-3.98586900	2.16326700
6	3.44298500	-2.59674800	2.26784900
6	2.51409500	-1.77370400	1.62746400
6	-1.29434700	-2.10452100	0.01104900
6	-1.65059700	-2.81284000	1.17429800
6	-2.95078600	-3.29465700	1.33295500
6	-3.91446300	-3.05988800	0.34886300
6	-3.57217400	-2.34907900	-0.80439100
6	-2.27185100	-1.87487000	-0.97450100
1	5.13578700	-1.83351500	-2.58071500
1	3.77860000	-3.14166800	-0.64467100
1	1.91782600	-4.70706400	-1.84412500
1	2.11848700	-4.34775200	-4.51397500
1	4.10102700	-2.56531000	-4.97282300
1	-0.48924000	-2.33691000	-2.99854800
1	0.81144400	-1.21965300	-5.08111100
1	2.71482000	0.40955900	-4.11552900
1	3.33010000	-0.19805700	-0.45969200
1	3.61488800	1.92807500	-2.66317500
1	4.61261000	0.48572700	-2.42015900
1	4.67322300	1.81623100	-1.24719700
1	0.57235600	-4.17253900	0.17255800
1	2.20572800	-5.63315900	1.32909700
1	4.24460100	-2.15085200	2.86056300
1	2.57980100	-0.69379200	1.74949300
1	-0.92664300	-2.96899700	1.97359000
1	-3.20935200	-3.83648600	2.24422000
1	-4.32373200	-2.14378900	-1.56630600
1	-2.03575000	-1.30423200	-1.87200600
27	0.18144300	0.65319400	1.04097600
8	-3.10369900	3.12133200	-1.73556400
8	-5.02220300	1.35777900	2.02709500
7	-4.34424600	2.30410800	0.03143200
6	-3.17100300	2.71317500	-0.59980100
6	-0.76516700	0.67568800	3.15157000
6	-1.95120800	0.64374400	1.61098100
1	-2.49793700	-0.28810700	1.76518300
6	-2.68673000	1.99620300	1.67878900
1	-2.57907400	2.59722200	2.59519200
6	-4.16017600	1.83275300	1.32839800
6	-5.64030100	2.27312200	-0.64359900
1	-5.63521200	3.09840200	-1.36763100
1	-6.40245900	2.47027000	0.12116500
6	-5.91655700	0.95795500	-1.34005100
6	-6.67399100	-0.03595800	-0.70491000
1	-7.03403300	0.13402400	0.31224900
6	-6.97473600	-1.22769500	-1.36980600
1	-7.58415700	-1.98727000	-0.87435400
6	-6.50988700	-1.44129100	-2.67125500
1	-6.75824700	-2.36627700	-3.19701100
6	-5.72850100	-0.46529100	-3.29952600
1	-5.36462800	-0.62729000	-4.31698300
6	-5.43248200	0.72891600	-2.63722700
1	-4.83607400	1.50116900	-3.12906100
6	-2.02944300	2.46643100	0.36892700
1	-1.31740500	3.30034700	0.39628600
6	-1.46715800	1.03305500	0.21405500
1	-1.81681200	0.46793900	-0.65216300
6	1.37299800	3.16902300	-0.81785100
6	2.47596100	4.18388400	-1.18029400
6	0.50804700	2.85365800	-2.05276000
1	0.72611400	3.65324500	-0.06499400
6	1.84950400	5.46783100	-1.73927900
1	3.14133000	3.75402900	-1.94163100
1	3.10414100	4.41775200	-0.31068400
6	-0.10277600	4.13438000	-2.63167400
1	1.13721400	2.37183600	-2.81779800
1	-0.28334100	2.13290800	-1.80739800
6	0.96979100	5.17570100	-2.95783800
1	2.64675800	6.18332900	-1.99690600
1	1.24159200	5.94713800	-0.95071300
1	-0.69658200	3.88511500	-3.52415000
1	-0.82226600	4.54986800	-1.90678000
1	0.50332200	6.10370200	-3.32452900

1	1.60681500	4.80108900	-3.77999800
6	3.03053300	2.10927100	1.49329200
6	4.54412100	2.23789000	1.26843900
6	2.46332400	3.34344900	2.22703300
1	2.87744300	1.26194000	2.18253200
6	5.26139800	2.46569700	2.60685900
1	4.75869200	3.07955600	0.58919900
1	4.94427400	1.32933100	0.79366500
6	3.18694500	3.54845800	3.56154000
1	2.59741000	4.24269700	1.60574300
1	1.38734500	3.21363200	2.40268700
6	4.69968100	3.67267500	3.36239400
1	6.34208600	2.58635400	2.42966000
1	5.14548200	1.56052800	3.23043500
1	2.78861100	4.44448900	4.06378000
1	2.96355000	2.69253000	4.22227000
1	5.20842100	3.78360100	4.33306500
1	4.91710600	4.59268800	2.78981200
8	0.48633800	1.05217700	2.83627600
6	-0.93032700	-0.65193600	3.82567700
6	-2.20055600	-1.08474000	4.23713400
6	0.18081600	-1.47527700	4.04077400
6	-2.35760600	-2.34148200	4.82083500
1	-3.07341500	-0.44380000	4.08357900
6	0.02319900	-2.73581000	4.62071200
1	1.16652400	-1.11470400	3.75526500
6	-1.24747500	-3.17549000	5.00343400
1	-3.34944800	-2.67365100	5.13542600
1	0.89630900	-3.37333300	4.77691600
1	-1.37318800	-4.16095800	5.45763600
1	-4.93742100	-3.41797500	0.48209700
1	4.05457000	-4.63187900	2.66848000
1	-1.35245200	1.47322600	3.62957600

^{3}TSB

E = -5781.77898308 G = -5780.81431

26	3.63148200	-0.91718600	-2.22671000
15	1.33011700	2.11213200	0.66307100
15	0.81063100	-1.36394800	-0.11694000
6	5.57476300	-1.12859300	-1.51411400
6	4.77924600	-2.23622700	-1.09993200
6	4.28092500	-2.88200700	-2.27166700
6	4.77023200	-2.17250100	-3.40834600
6	5.56666500	-1.08477500	-2.94073400
6	1.74456600	-0.56757000	-1.45460000
6	1.68333500	-0.81776800	-2.86822000
6	2.46327900	0.17395500	-3.52806000
6	3.03141900	1.02820600	-2.53716000
6	2.59757200	0.58517100	-1.24883200
6	2.89387200	1.24231000	0.07609200
6	4.18763700	2.05977300	0.09672000
6	1.86526200	-2.66471200	0.60110400
6	1.97933600	-3.93853500	0.02674800
6	2.82444000	-4.88991300	0.60029800
6	3.56786400	-4.57347500	1.74266100
6	3.46166700	-3.30245400	2.31526500
6	2.60686300	-2.35359000	1.75018900
6	-0.64459200	-2.17940700	-0.84501100
6	-1.22532500	-3.26744700	-0.16861000
6	-2.47841800	-3.74525400	-0.55712500
6	-3.17271500	-3.13573700	-1.60502400
6	-2.60423700	-2.05042300	-2.27796600
6	-1.34601400	-1.57584300	-1.90480500
1	6.09798900	-0.43693200	-0.85697500
1	4.58202900	-2.53252000	-0.07434000
1	3.63241600	-3.75403100	-2.29307300
1	4.54922500	-2.40083800	-4.44949500
1	6.06252300	-0.34128200	-3.56223100
1	1.14144800	-1.63068000	-3.34524000
1	2.63625200	0.23907000	-4.60047400
1	3.70968600	1.85526000	-2.73190600
1	3.00050700	0.44407000	0.82906300
1	4.13951200	2.94626400	-0.54641300
1	5.01263100	1.42799800	-0.26049600
1	4.43275900	2.38834400	1.11528300
1	1.40393800	-4.18946300	-0.86619300
1	2.90712900	-5.88261700	0.15244200
1	4.03449900	-3.05407800	3.21127400
1	2.49404300	-1.37548700	2.22300100
1	-0.71794700	-3.72866200	0.67908300
1	-2.91738600	-4.58690300	-0.01811700
1	-3.15321300	-1.56258800	-3.08432300
1	-0.92024000	-0.71984600	-2.43072400
27	0.02897700	0.34466700	1.32710700
8	-3.52406900	3.09227400	-0.91182000
8	-5.33083900	-0.21135200	1.69214200
7	-4.70583600	1.48877200	0.25729800

6	-3.60071100	2.29118900	-0.00988500
6	-1.18763500	-0.68459100	3.05894000
6	-2.14328700	-0.19189200	1.53292900
1	-2.49837500	-1.16315400	1.18232900
6	-3.16596500	0.89665700	1.94551900
1	-3.25107900	1.12400300	3.01887100
6	-4.53521500	0.62378100	1.33701600
6	-5.90334300	1.48316400	-0.58324000
1	-6.04421800	2.51337800	-0.93560200
1	-6.74316100	1.20778700	0.06735100
6	-5.80107300	0.52994800	-1.75379500
6	-6.29351200	-0.77805700	-1.64652800
1	-6.72885700	-1.11064700	-0.70133200
6	-6.23886800	-1.64416400	-2.74161700
1	-6.64822000	-2.65368700	-2.65638900
6	-5.68035500	-1.21451800	-3.94915100
1	-5.65057300	-1.88756300	-4.80924100
6	-5.16647800	0.08238900	-4.05492300
1	-4.73283100	0.42381500	-4.99789600
6	-5.22558600	0.95095200	-2.96222900
1	-4.83552100	1.96822500	-3.04297600
6	-2.51928800	1.91738000	0.98839400
1	-2.01664000	2.80140600	1.39862100
6	-1.64064400	0.76229300	0.48812200
1	-1.64627100	0.47004400	-0.56372300
6	0.72769600	3.33878800	-0.60330600
6	1.81958700	4.25269700	-1.19656100
6	-0.09244300	2.70802000	-1.74543400
1	0.03970000	3.96542000	-0.00465800
6	1.19774000	5.34108000	-2.08021300
1	2.49035000	3.63759000	-1.81512500
1	2.43431500	4.71089300	-0.41329800
6	-0.70000700	3.78565100	-2.65027300
1	0.55838400	2.04407900	-2.33509500
1	-0.90017000	2.09327100	-1.34180100
6	0.36225500	4.73337500	-3.20823500
1	1.99666800	5.98162500	-2.48705100
1	0.55998800	5.99335900	-1.45686400
1	-1.25562800	3.29837300	-3.46744600
1	-1.44874000	4.34988100	-2.06963700
1	-0.10839000	5.52787900	-3.80865000
1	1.02941200	4.17659900	-3.89131100
6	1.70334100	3.01887100	2.24936400
6	2.46974900	4.34942900	2.15128000
6	0.40148500	3.21952400	3.05477700
1	2.32530400	2.29011700	2.80262700
6	2.77106300	4.91057100	3.54703500
1	1.84928100	5.07852900	1.60420100
1	3.40192400	4.23409400	1.58273700
6	0.68672200	3.80674800	4.44168500
1	-0.26347200	3.90415300	2.49671600
1	-0.12276900	2.25987300	3.16793300
6	1.48990000	5.10646300	4.36031000
1	3.31773000	5.86194500	3.44916000
1	3.44480800	4.21530000	4.07943400
1	-0.26372800	3.96941000	4.97466500
1	1.24960800	3.05985500	5.02902700
1	1.72822800	5.47321600	5.37107600
1	0.87299800	5.88845000	3.88146500
8	0.05804900	-0.18230000	3.12116900
6	-1.32587500	-2.18201900	3.15164000
6	-2.58714300	-2.79574000	3.07512200
6	-0.19009900	-2.97415400	3.34173300
6	-2.69633900	-4.18440600	3.14123300
1	-3.48995900	-2.19138000	2.94847100
6	-0.29718200	-4.36583300	3.39895000
1	0.77443800	-2.48635800	3.45041100
6	-1.54965000	-4.97517600	3.28822800
1	-3.68065800	-4.65364700	3.07742800
1	0.60160700	-4.97185100	3.53344900
1	-1.63754400	-6.06309700	3.33253900
1	-4.16133800	-3.49417200	-1.89544100
1	4.22996300	-5.31973400	2.18706600
1	-1.87964200	-0.17427000	3.75084600

TSC

E = -5781.77634375 G = -5780.808042

26	2.80204000	-2.63886900	-0.98166100
15	-0.34248700	0.90549400	-1.52081300
15	1.92789500	0.40997300	0.82884400
6	4.51721800	-3.61838200	-0.35953100
6	3.85706900	-4.36675000	-1.38047000
6	3.78812300	-3.55339100	-2.55098100
6	4.40683800	-2.30148200	-2.25499800
6	4.85889100	-2.34340100	-0.89925300
6	1.74334700	-1.19077300	0.01970100
6	1.58494600	-2.45949800	0.67715300

6	1.01303500	-3.36692200	-0.25469500
6	0.79489500	-2.67624000	-1.48339800
6	1.22828900	-1.32629000	-1.32252200
6	1.01547100	-0.16771100	-2.25299300
6	0.85584800	-0.55798800	-3.72052400
6	3.28053200	1.40036500	0.10951700
6	3.49672700	2.66816400	0.67595900
6	4.46793100	3.51306800	0.14043000
6	5.22497100	3.10387900	-0.96426200
6	5.01278200	1.84246400	-1.52550500
6	4.04217500	0.99078900	-0.98964500
6	2.52811300	0.05562100	2.51426700
6	1.99855100	0.72654700	3.62564400
6	2.51035100	0.46680200	4.90086300
6	3.54489500	-0.45581700	5.06980100
6	4.08423700	-1.11363200	3.95856600
6	3.58497800	-0.85618200	2.68238900
1	4.70909700	-3.95638300	0.65716600
1	3.45407900	-5.37240300	-1.27603800
1	3.33137300	-3.83453000	-3.49814200
1	4.52326100	-1.47075700	-2.94874800
1	5.36852800	-1.54157900	-0.36862700
1	1.83159700	-2.68509500	1.71010800
1	0.81237300	-4.41930000	-0.06646200
1	0.38879600	-3.11161500	-2.39316400
1	1.88496900	0.49959800	-2.17345000
1	0.00038900	-1.22712600	-3.87944800
1	1.76145800	-1.08940900	-4.05098100
1	0.72456800	0.32289900	-4.36409700
1	2.89771400	2.99697800	1.52938300
1	4.63529700	4.49683900	0.58431800
1	5.60657900	1.51672600	-2.38269200
1	3.87217500	0.00611000	-1.41703700
1	1.20622000	1.46350500	3.48451400
1	2.09889400	0.99214400	5.76558400
1	4.90087200	-1.82736600	4.08767400
1	4.01138100	-1.36337600	1.81556200
27	-0.08387100	1.27664200	0.70643700
8	-0.73324500	-3.14632700	2.82621500
8	-4.69932700	-0.93022600	2.23813500
7	-2.86954200	-2.30999700	2.53816300
6	-1.48891000	-2.20939500	2.71493200
6	-1.34381200	2.47816100	2.21571800
6	-2.13518900	0.72807800	1.36800700
1	-2.90048600	1.01750200	0.64306400
6	-2.48564400	0.02102200	2.68441600
1	-2.76841500	0.61500100	3.56851500
6	-3.52301100	-1.08006700	2.45629400
6	-3.56184200	-3.58604900	2.37709900
1	-3.01121500	-4.31662100	2.98711000
1	-4.57080800	-3.46321500	2.79050500
6	-3.63246700	-4.05033100	0.93702600
6	-2.45510500	-4.33060700	0.22484500
1	-1.48996200	-4.20093200	0.71639300
6	-2.51848800	-4.79840600	-1.08857900
1	-1.59570000	-5.02092200	-1.63022900
6	-3.75830400	-5.00773300	-1.70209800
1	-3.80859500	-5.38920700	-2.72446500
6	-4.93318900	-4.72682100	-1.00017400
1	-5.90492100	-4.88209100	-1.47420600
6	-4.86927300	-4.23979800	0.30908900
1	-5.78971900	-4.01098900	0.85155400
6	-1.14264600	-0.74027500	2.63815300
1	-0.32897300	-0.45435900	3.31347800
6	-1.04757700	-0.26900500	1.16419400
1	-1.05457900	-1.04469600	0.39904300
6	-2.02107000	0.35966300	-2.13961400
6	-2.33292000	0.72191200	-3.60905100
6	-2.39641700	-1.11518700	-1.89794200
1	-2.69096200	0.97067700	-1.50884600
6	-3.82266300	0.50666000	-3.90745300
1	-1.73513800	0.08436600	-4.27739600
1	-2.06156400	1.75984300	-3.84030400
6	-3.88682200	-1.33447500	-2.17932700
1	-1.79732600	-1.76040000	-2.56046900
1	-2.17799600	-1.44522800	-0.87811300
6	-4.25929800	-0.92843100	-3.60557600
1	-4.02637700	0.76282100	-4.95955400
1	-4.41544800	1.20951600	-3.29437900
1	-4.13863200	-2.38669600	-1.99416600
1	-4.47510100	-0.74313200	-1.45448700
1	-5.34292400	-1.04192700	-3.76779700
1	-3.76397900	-1.61455300	-4.31665800
6	-0.11168100	2.59539800	-2.26773200
6	-1.29242100	3.51001700	-1.89426700
6	1.21396400	3.23222000	-1.81961800
1	-0.08565300	2.45969400	-3.36297900
6	-1.11647200	4.92599300	-2.45178300

1	-1.36070600	3.56219300	-0.79734200
1	-2.24827200	3.09579300	-2.24311100
6	1.38974000	4.63804800	-2.39877700
1	1.22604100	3.29317900	-0.71874100
1	2.07153200	2.60612900	-2.10142700
6	0.21628000	5.54639300	-2.02736700
1	-1.95900600	5.55146300	-2.11746900
1	-1.16924700	4.88959700	-3.55485500
1	2.34174900	5.06097400	-2.04060600
1	1.47092100	4.56943200	-3.49863100
1	0.33870900	6.54114400	-2.48422200
1	0.21310700	5.70117600	-0.93411100
8	-0.03029200	2.57476500	2.03895100
6	-2.20660800	3.59589000	1.74521300
6	-3.60775400	3.49591500	1.78555500
6	-1.61229700	4.78460600	1.29696900
6	-4.39799800	4.56099700	1.35550000
1	-4.08450200	2.58088000	2.14556800
6	-2.40621100	5.85452000	0.88027400
1	-0.52416200	4.85832300	1.29062000
6	-3.79902700	5.74172800	0.89974100
1	-5.48635300	4.47422300	1.38084600
1	-1.93609900	6.78029100	0.54114900
1	-4.42094200	6.57674500	0.56967800
1	3.93908500	-0.65909400	6.06789200
1	5.98292700	3.76908800	-1.38347400
1	-1.63033600	2.07839100	3.19746200

^{3}TSC

E = -5781.77798638 G = -5780.813038

26	2.95761100	-2.06920300	-1.60968000
15	-0.23799700	1.51316200	-1.17491100
15	1.97693100	0.16275700	1.11026600
6	4.72125000	-3.13057600	-1.37751700
6	4.07461000	-3.49891500	-2.59573100
6	3.95209800	-2.32870900	-3.40306100
6	4.52364800	-1.23532600	-2.68531400
6	5.00110900	-1.73334100	-1.43259200
6	1.86226400	-1.06213100	-0.20514900
6	1.78056900	-2.48163600	0.02746400
6	1.22560800	-3.08561600	-1.13155500
6	0.93869400	-2.06044400	-2.07946700
6	1.30657500	-0.80064100	-1.52053200
6	1.07721300	0.55661200	-2.12942300
6	0.89569500	0.53848500	-3.64662100
6	3.16277800	1.49168500	0.73225200
6	3.17778800	2.59618900	1.60202200
6	4.04304900	3.66209100	1.35658700
6	4.88821900	3.63727800	0.24092900
6	4.87110900	2.54040100	-0.62460100
6	4.01071200	1.46719400	-0.38022800
6	2.63429900	-0.63724600	2.59995900
6	2.09100800	-0.27777400	3.84309400
6	2.61628900	-0.82362600	5.01689200
6	3.67863100	-1.72925700	4.95039500
6	4.22738500	-2.08045200	3.71199900
6	3.71461300	-1.53147300	2.53626200
1	4.94488900	-3.79683700	-0.54635000
1	3.71541500	-4.49362600	-2.85293700
1	3.49080800	-2.27782000	-4.38765600
1	4.59200500	-0.20879000	-3.04122400
1	5.48878000	-1.15000900	-0.65376700
1	2.05926800	-3.00140500	0.93915700
1	1.08166800	-4.15371200	-1.27825900
1	0.53280400	-2.21422200	-3.07613600
1	1.96463500	1.16296900	-1.90241500
1	-0.00167300	-0.01877900	-3.94755200
1	1.76523200	0.05838200	-4.12142000
1	0.80826800	1.55736200	-4.04940600
1	2.50785900	2.62855200	2.46546000
1	4.05341500	4.51903100	2.03330100
1	5.53129000	2.51887400	-1.49466200
1	3.98528000	0.60949400	-1.04738700
1	1.26353900	0.43592600	3.89006400
1	2.19512800	-0.54157500	5.98422300
1	5.06302500	-2.78199000	3.66394300
1	4.14945800	-1.79540200	1.57059600
27	-0.09429400	1.05067500	1.18690800
8	-0.32806900	-3.90964100	2.02280000
8	-4.46701800	-1.95703600	2.17248100
7	-2.53226200	-3.21231500	2.05336600
6	-1.15699500	-3.05227900	2.21648100
6	-1.95937300	1.74473000	2.45547000
6	-2.05030600	0.02980000	1.61819000
1	-2.80595000	0.18365200	0.84672400
6	-2.31110700	-0.97205500	2.77087000
1	-2.60883300	-0.56398100	3.74828700

6	-3.27372900	-2.06295100	2.31873900
6	-3.13428800	-4.42073200	1.49263600
1	-2.47522600	-5.25424000	1.77225500
1	-4.11043000	-4.55732900	1.97466800
6	-3.28630700	-4.33485400	-0.01141800
6	-2.14653600	-4.30028700	-0.83043000
1	-1.15786800	-4.34616100	-0.37087100
6	-2.27610300	-4.22994000	-2.21831900
1	-1.38267900	-4.21249100	-2.84722500
6	-3.54595900	-4.20664200	-2.80486600
1	-3.64861400	-4.16611900	-3.89164700
6	-4.68375900	-4.23493000	-1.99440400
1	-5.67810800	-4.21051200	-2.44573800
6	-4.55393400	-4.28804800	-0.60320300
1	-5.44445600	-4.29516300	0.02946000
6	-0.91525800	-1.59719300	2.57313200
1	-0.13900300	-1.47539700	3.33673600
6	-0.81967500	-0.74471200	1.29828100
1	-0.66440300	-1.23248300	0.33345300
6	-1.95912900	1.15052000	-1.80820400
6	-2.40986800	1.86179800	-3.09636000
6	-2.28024800	-0.35213300	-1.90086800
1	-2.57136700	1.56468500	-0.98538300
6	-3.91233300	1.64243600	-3.32599600
1	-1.84079900	1.47126400	-3.95607800
1	-2.20855600	2.94078600	-3.04571300
6	-3.78399200	-0.56413200	-2.09873200
1	-1.73409200	-0.79332700	-2.75066300
1	-1.94350000	-0.90093700	-1.01422000
6	-4.28123100	0.15669100	-3.35320600
1	-4.21826000	2.13551600	-4.26264300
1	-4.46908300	2.14163700	-2.51253900
1	-4.00069900	-1.63948900	-2.15166600
1	-4.32037500	-0.17938100	-1.21208600
1	-5.37102300	0.03593700	-3.46022700
1	-3.82634400	-0.31592900	-4.24299600
6	0.06936500	3.28651600	-1.66024900
6	-0.86691500	4.18695100	-0.83965100
6	1.52918800	3.72838000	-1.48268600
1	-0.18844600	3.37239000	-2.72914400
6	-0.68151100	5.66842100	-1.17530200
1	-0.64538500	4.02030900	0.22575500
1	-1.91862700	3.89763900	-0.97982500
6	1.71163700	5.21305200	-1.81712900
1	1.83663100	3.55349400	-0.44063900
1	2.20187800	3.13106000	-2.11474100
6	0.77518800	6.09952900	-0.99307700
1	-1.34980300	6.27470900	-0.54249500
1	-0.99174000	5.84934900	-2.22022700
1	2.76320000	5.49551900	-1.64828100
1	1.51253100	5.37128500	-2.89245600
1	0.90391900	7.15760200	-1.27111700
1	1.04610700	6.01848400	0.07533600
8	-0.77100500	2.34941300	2.35505400
6	-3.15847000	2.48693400	1.92295800
6	-4.41434300	1.86816900	1.78641000
6	-3.03556000	3.85010200	1.62546600
6	-5.50817400	2.59359600	1.31317100
1	-4.55301400	0.81501600	2.04280400
6	-4.13268600	4.57546700	1.15509800
1	-2.07335300	4.33529300	1.78154500
6	-5.36848600	3.94706500	0.98546700
1	-6.47633300	2.09987800	1.20451900
1	-4.02209400	5.63821100	0.92704200
1	-6.22640400	4.51227700	0.61452700
1	4.08532900	-2.16030500	5.86773600
1	5.56076800	4.47572300	0.04763500
1	-2.14388300	1.33943900	3.46517300

TSD

E = -5781.77668778 G = -5780.80827

26	-3.62706900	-2.08678200	-1.58375000
15	-0.19473900	-1.08024700	1.60015800
15	-1.84116100	0.84182200	-0.39538400
6	-5.24810900	-1.71858800	-2.81852700
6	-5.01097900	-3.12225500	-2.70989300
6	-5.12845000	-3.48584900	-1.33509200
6	-5.44017300	-2.30737400	-0.59289700
6	-5.51522600	-1.21456500	-1.51184900
6	-2.10078100	-0.79044900	-1.09400200
6	-2.00133500	-1.17904500	-2.47355900
6	-1.85896300	-2.59361800	-2.51656900
6	-1.86585200	-3.08857700	-1.17832100
6	-1.99209600	-1.98024300	-0.28983700
6	-1.82760800	-1.93063400	1.20091800
6	-2.06649700	-3.25915600	1.91666400
6	-3.19522500	1.35190100	0.71553100

6	-3.10824500	2.64200500	1.26795800
6	-4.06475400	3.06813500	2.18926800
6	-5.10740900	2.21605000	2.57086500
6	-5.19450000	0.93438400	2.02181100
6	-4.24070000	0.50312000	1.09639600
6	-1.95414600	1.99631500	-1.80034300
6	-0.92796200	2.90882400	-2.07180000
6	-1.04269500	3.79529200	-3.14575800
6	-2.18239100	3.77006400	-3.95235800
6	-3.21399300	2.86318200	-3.67994000
6	-3.10648600	1.98085600	-2.60517700
1	-5.21394400	-1.13436500	-3.73626000
1	-4.75843700	-3.79311800	-3.52891700
1	-4.99052900	-4.48436100	-0.92440600
1	-5.60677500	-2.25974300	0.48170800
1	-5.73471000	-0.17941000	-1.25833300
1	-2.03612500	-0.50893200	-3.32862000
1	-1.79417300	-3.19747800	-3.41937500
1	-1.79830500	-4.13484500	-0.89032600
1	-2.54886000	-1.20519500	1.59944100
1	-1.38470400	-4.04742500	1.57345500
1	-3.09446600	-3.59460000	1.70997100
1	-1.95575200	-3.16177400	3.00565900
1	-2.28091000	3.29855000	0.99612600
1	-3.99392300	4.07095300	2.61606200
1	-6.00983800	0.26724400	2.31108600
1	-4.30575400	-0.48942700	0.65985300
1	-0.04891200	2.94476100	-1.43325000
1	-0.23953700	4.50836600	-3.34447400
1	-4.10904100	2.84922000	-4.30575500
1	-3.91334200	1.27806500	-2.38850300
27	0.12393300	0.85203100	0.56227400
8	2.55058800	-1.20805600	-3.23635900
8	5.01772200	2.01591800	-1.12245700
7	4.03470600	0.30702800	-2.32475500
6	2.76531100	-0.20622400	-2.59847900
6	1.33134700	2.73404600	1.26447400
6	2.24465300	1.23412100	0.20874900
1	3.04925900	1.04238800	0.92593100
6	2.58268600	1.78425200	-1.20003100
1	2.36412000	2.83792000	-1.41423100
6	4.03122300	1.44824600	-1.52914500
6	5.25663000	-0.41517300	-2.67064300
1	5.13608600	-0.80945300	-3.68755400
1	6.06848400	0.32502000	-2.66392200
6	5.53832200	-1.53658900	-1.69207800
6	5.48097200	-2.87452200	-2.09972400
1	5.21659800	-3.11033300	-3.13300800
6	5.75823400	-3.90403600	-1.19528900
1	5.72088900	-4.94365400	-1.52855300
6	6.07612700	-3.60482600	0.13144700
1	6.29836700	-4.40837300	0.83731600
6	6.11431900	-2.27084500	0.55226600
1	6.37262100	-2.03123700	1.58669000
6	5.85325300	-1.24166300	-0.35507900
1	5.91764900	-0.19817600	-0.03700900
6	1.75994500	0.66200600	-1.86606500
1	0.88966900	0.90393900	-2.48795700
6	1.51881800	0.09086300	-0.46102700
1	1.84635700	-0.93082900	-0.29291000
6	1.17269900	-2.34117400	1.51214800
6	1.35967400	-3.27649300	2.72270500
6	1.13979200	-3.17845400	0.21667400
1	2.07462700	-1.70486900	1.47687700
6	2.67637400	-4.05389000	2.56760000
1	0.51757100	-3.98293000	2.78680000
1	1.37493200	-2.71662300	3.66793800
6	2.45921000	-3.93344400	0.04677300
1	0.31399900	-3.90147300	0.28640000
1	0.92979600	-2.56721400	-0.67159000
6	2.73759500	-4.83510700	1.25148900
1	2.80654000	-4.73207400	3.42607300
1	3.51672600	-3.33729200	2.60264900
1	2.43574400	-4.52233500	-0.88321800
1	3.27946400	-3.20694600	-0.07337600
1	3.72079800	-5.31900600	1.14516800
1	1.98912000	-5.64789000	1.27763300
6	-0.32753900	-0.58203600	3.38611400
6	0.99687400	0.03804300	3.87073400
6	-1.49861400	0.38628400	3.63147800
1	-0.51023400	-1.51287900	3.95150300
6	0.91304700	0.46222900	5.34056300
1	1.20983100	0.92618700	3.25550200
1	1.83853000	-0.65508900	3.72333400
6	-1.57397000	0.81057800	5.10154400
1	-1.36000900	1.28016900	3.00379800
1	-2.45463100	-0.06498000	3.32843200
6	-0.25473500	1.42286700	5.57754700

1	1.86570500	0.92374500	5.64606500
1	0.78566000	-0.43730600	5.96927400
1	-2.40261000	1.52501400	5.22942500
1	-1.81753500	-0.06820200	5.72566100
1	-0.31751700	1.69377500	6.64319400
1	-0.07055800	2.36108800	5.02352200
8	0.00937100	2.60331400	1.20784100
6	1.93537700	3.94724500	0.65271600
6	1.12647000	4.88865600	-0.00113400
6	3.31912000	4.16758300	0.73944200
6	1.69889300	6.02382300	-0.57926700
1	0.04855300	4.73295300	-0.02966500
6	3.88918200	5.29675900	0.15534200
1	3.96130300	3.43591300	1.23378900
6	3.08052100	6.22592400	-0.50877500
1	1.06263500	6.75859700	-1.07794500
1	4.96888000	5.44766000	0.21038800
1	3.52819700	7.11170600	-0.96481000
1	-2.27317800	4.46160500	-4.79283100
1	-5.85272300	2.55372000	3.29430900
1	1.79664900	2.47760400	2.23199500

[3]TSD

E = -5781.77906914 G = -5780.815319

26	-2.88669100	-2.74779200	0.14572200
15	-0.44635400	1.00267200	1.94602700
15	-1.68030100	0.37776600	-1.28725100
6	-4.19017100	-3.84785900	-1.02862900
6	-3.87833700	-4.55818900	0.16988200
6	-4.28991000	-3.75886700	1.27797000
6	-4.85795400	-2.55377600	0.76555700
6	-4.79737100	-2.61061100	-0.66189600
6	-1.67755900	-1.19761900	-0.41653700
6	-1.19077600	-2.43027700	-0.97995000
6	-0.89705400	-3.31510600	0.09077500
6	-1.18325300	-2.64795300	1.31713900
6	-1.64213000	-1.32728900	1.02803100
6	-1.88515900	-0.21851100	2.02009200
6	-2.29287500	-0.70507600	3.40975500
6	-3.19543900	1.33987300	-0.97152800
6	-3.22870400	2.65925300	-1.45291000
6	-4.34904000	3.45662000	-1.22184300
6	-5.43746300	2.94674900	-0.50458900
6	-5.40561200	1.63402800	-0.02666400
6	-4.28727600	0.82949000	-0.26008700
6	-1.70629100	0.06542900	-3.07478100
6	-0.97096000	0.91664800	-3.91472400
6	-1.02462000	0.74564300	-5.29995300
6	-1.80738600	-0.27484200	-5.84697000
6	-2.54763500	-1.11799800	-5.01120200
6	-2.50638200	-0.94710200	-3.62697900
1	-3.98462900	-4.18596200	-2.04264200
1	-3.38942300	-5.52884900	0.22817900
1	-4.17710200	-4.01773200	2.32915900
1	-5.27452100	-1.74415300	1.36220800
1	-5.14764300	-1.84318700	-1.34965500
1	-1.03406200	-2.64052500	-2.03385800
1	-0.54609800	-4.33918100	-0.01505100
1	-1.08954600	-3.08326500	2.30887200
1	-2.70120700	0.40484700	1.62971000
1	-1.51988000	-1.33309000	3.87227500
1	-3.21096600	-1.30781500	3.33418400
1	-2.49272500	0.13721900	4.08698500
1	-2.37500900	3.06919000	-1.99915600
1	-4.37141300	4.48210300	-1.59645200
1	-6.25591900	1.23231700	0.52913800
1	-4.25122900	-0.19285800	0.10728600
1	-0.35759900	1.71347700	-3.48438500
1	-0.45236700	1.40844100	-5.95247500
1	-3.16396900	-1.91021300	-5.44162700
1	-3.09169500	-1.59689600	-2.97457500
27	0.13264200	1.35521100	-0.34359600
8	1.58126000	-2.82355300	-2.61869600
8	5.20085800	-0.39667900	-1.21462500
7	3.57258900	-1.85595300	-1.95601900
6	2.22991700	-1.84899300	-2.31341900
6	2.09208300	2.65863200	-0.27232600
6	2.35995000	0.80819600	-0.43621300
1	2.95860400	0.64404800	0.46703100
6	2.97081300	0.42630800	-1.81073000
1	3.28164200	1.24683000	-2.46977400
6	4.08430600	-0.59796300	-1.62277400
6	4.32209000	-3.09389300	-1.74273500
1	3.97095900	-3.81341400	-2.49401400
1	5.37978300	-2.86671900	-1.92466500
6	4.11360200	-3.62548800	-0.34091400
6	2.95205400	-4.34804800	-0.02428600

1	2.21526200	-4.54154000	-0.80667000
6	2.74651900	-4.81747900	1.27487900
1	1.84587200	-5.39001300	1.51043500
6	3.70208500	-4.57769600	2.26758400
1	3.55008900	-4.96149800	3.27919400
6	4.85648400	-3.85203500	1.95966600
1	5.60564700	-3.66147000	2.73154800
6	5.05747300	-3.37082800	0.66318700
1	5.95358500	-2.79401200	0.42312800
6	1.73587100	-0.42516300	-2.15939800
1	1.08261800	-0.12919300	-2.98729200
6	1.25215200	-0.16364500	-0.73096800
1	0.98551700	-0.99366600	-0.07131300
6	0.93387200	0.48873800	3.09208200
6	0.67971300	0.68488000	4.59857600
6	1.47081900	-0.92662400	2.81861500
1	1.73163100	1.19742400	2.80072300
6	1.95872800	0.41873000	5.40309400
1	-0.10901600	-0.00680400	4.93214300
1	0.31694400	1.70058800	4.80986900
6	2.74890800	-1.18865400	3.62177500
1	0.70706200	-1.66834600	3.10057400
1	1.66722500	-1.08185800	1.74961900
6	2.52598400	-0.97435200	5.12004700
1	1.75153600	0.54352300	6.47791700
1	2.71328700	1.18211300	5.14026900
1	3.10470400	-2.20811900	3.41716900
1	3.54285300	-0.50723100	3.26498900
1	3.46620900	-1.12549600	5.67378900
1	1.81830000	-1.73707800	5.49345100
6	-1.09947300	2.58740500	2.66985100
6	0.02691300	3.63888500	2.72874800
6	-2.27990700	3.13295700	1.85081000
1	-1.44759200	2.36988400	3.69456500
6	-0.48133800	4.97083200	3.29246600
1	0.40236300	3.79787200	1.70395700
1	0.87210500	3.27955000	3.33493800
6	-2.78728700	4.46716700	2.40531100
1	-1.94581900	3.28038000	0.81139900
1	-3.10853500	2.41120100	1.81362700
6	-1.66593400	5.50520200	2.48433200
1	0.34183600	5.70287300	3.30239000
1	-0.78701200	4.82844100	4.34479100
1	-3.61345800	4.83135700	1.77375900
1	-3.21106000	4.30405800	3.41275300
1	-2.04004100	6.44315500	2.92444000
1	-1.32518800	5.74915600	1.46209200
8	0.85533400	3.05625700	-0.62153700
6	3.21468300	3.21962800	-1.09131600
6	2.94202500	3.89793800	-2.28591900
6	4.54385100	3.04658800	-0.67968800
6	3.99115900	4.39869800	-3.06103700
1	1.90237800	4.03774800	-2.58527400
6	5.58976300	3.54152600	-1.45727300
1	4.76602700	2.50523400	0.24335600
6	5.31523600	4.21830600	-2.65092400
1	3.77475300	4.93574400	-3.98746800
1	6.62256200	3.39602800	-1.13422600
1	6.13490800	4.60706500	-3.25921600
1	-1.84423700	-0.41240600	-6.92974900
1	-6.31152800	3.57491200	-0.31991400
1	2.30641300	2.76013300	0.80887600

^{3}TSA-concave

E = -5781.76739228 G = -5780.802674

26	4.73537300	-0.98057500	-0.58351700
15	0.02992400	-1.71629800	-0.39654100
15	2.10818300	1.08091500	0.60439600
6	5.04036000	-0.89103200	-2.63579900
6	5.15110000	0.41255000	-2.07082600
6	6.19935900	0.38707700	-1.10245700
6	6.73951600	-0.93414600	-1.07302700
6	6.02088800	-1.72617500	-2.01843800
6	3.11888600	-0.42066200	0.56207600
6	4.25420000	-0.71658000	1.39458900
6	4.62785400	-2.07001300	1.17027700
6	3.74932900	-2.61753300	0.18969600
6	2.81282900	-1.61118900	-0.20646000
6	1.71566700	-1.78027800	-1.23043000
6	1.94654100	-2.98456600	-2.15121300
6	2.91643200	2.35750200	-0.41589900
6	4.11711200	2.96449700	-0.01231600
6	4.74518300	3.88836900	-0.84662900
6	4.18143100	4.21150600	-2.08729700
6	2.98538600	3.61251100	-2.48928700
6	2.34813400	2.68662900	-1.65628700
6	2.20677000	1.69860600	2.32135100
6	2.03183900	3.07202900	2.56590400

6	1.93060400	3.54824800	3.87488500	6	-1.07308800	-1.26162100	-2.95352900
6	1.99459400	2.66145700	4.95375400	6	-1.60465800	-3.55259500	-1.95726400
6	2.16064000	1.29385400	4.71739800	1	-2.17140800	-1.67451000	-1.15694100
6	2.26248100	0.81173800	3.41023700	6	-2.29690000	-1.38278700	-3.86949500
1	4.32925800	-1.19372100	-3.40217900	1	-0.18859700	-1.63494500	-3.49534400
1	4.53792900	1.27144200	-2.32583500	1	-0.88564200	-0.20552100	-2.71462200
1	6.52217100	1.22544600	-0.48989100	6	-2.82492300	-3.65030500	-2.88342400
1	7.54057300	-1.28449400	-0.42463300	1	-0.74247200	-4.04915800	-2.43153400
1	6.18075100	-2.78422700	-2.21831300	1	-1.81494300	-4.08901500	-1.02092400
1	4.73216000	-0.02311100	2.08237000	6	-2.63396600	-2.84542900	-4.17161700
1	5.46536200	-2.58688900	1.63455600	1	-2.11755700	-0.82453500	-4.80217500
1	3.81557100	-3.62219500	-0.21931400	1	-3.16095000	-0.89877200	-3.37945000
1	1.69785300	-0.87345300	-1.85848000	1	-3.02961600	-4.70794000	-3.11386600
1	1.89174600	-3.93758000	-1.61319600	1	-3.71250200	-3.27320800	-2.34360300
1	2.94668100	-2.90816300	-2.60078000	1	-3.53671500	-2.91020300	-4.79952600
1	1.21260500	-3.01508700	-2.96454500	1	-1.81216500	-3.29180700	-4.76073500
1	4.56109500	2.71227100	0.95281200	8	-0.65613900	1.57200600	-1.36673700
1	5.67972100	4.35828500	-0.53207000	6	-2.14339300	3.40578700	-0.95920000
1	2.54155500	3.86720100	-3.45414300	6	-2.45308100	4.60278300	-0.29846300
1	1.41024700	2.21975100	-1.96720500	6	-2.93544600	2.97956000	-2.03275000
1	1.98464600	3.77455100	1.73115300	6	-3.55956500	5.35235600	-0.69040100
1	1.80592800	4.61883500	4.05225100	1	-1.82633000	4.94972800	0.52809100
1	2.21390300	0.59544600	5.55556000	6	-4.03997800	3.73525800	-2.42701500
1	2.38290700	-0.25827400	3.23924500	1	-2.66722800	2.05862100	-2.55133600
27	-0.15415900	0.51760900	0.10189200	6	-4.35864800	4.91607200	-1.75273500
6	-0.93579100	2.61133300	-0.57400600	1	-3.80282900	6.28029600	-0.16853200
6	-0.07075000	-2.96259300	0.97356100	1	-4.65673000	3.40149200	-3.26459900
6	0.51768400	-4.35205100	0.66786300	1	-5.22844600	5.50235200	-2.05678400
6	0.51257400	-2.42383300	2.29275100	1	1.91839100	3.03608200	5.97670300
1	-1.16174100	-3.04947600	1.10850200	1	4.67732000	4.93430400	-2.73888700
6	0.22728800	-5.32215100	1.82026400	1	-0.07165100	3.27124900	-0.36751300
1	1.60806100	-4.26081700	0.54909800	8	-4.79136700	2.48296200	0.40081300
1	0.12188800	-4.75647800	-0.27258700	8	-2.97994700	-1.64885200	0.97479000
6	0.23952700	-3.39331600	3.44737400	7	-3.90151000	0.35448600	0.30682000
1	1.59985000	-2.27991000	2.18023700	6	-3.93149100	1.70477700	0.71371700
1	0.07955700	-1.44196300	2.51968100	6	-0.95159900	0.77369700	1.84916900
6	0.77669300	-4.79483200	3.14785800	1	-0.18984300	0.66796600	2.62518600
1	0.65812800	-6.30929600	1.58801600	6	-2.41061700	0.44384400	2.12199300
1	-0.86448700	-5.46753500	1.90382300	1	-2.65665800	0.11547900	3.14154000
1	0.68378100	-2.99859400	4.37580800	6	-3.10194100	-0.44748700	1.09718300
1	-0.85065400	-3.44600800	3.61719100	6	-4.83576400	-0.17827000	-0.68839400
1	0.52685900	-5.48539000	3.96882700	1	-4.30011800	-0.95228100	-1.25217100
1	1.87993300	-4.75686300	3.09365600	1	-5.06731400	0.65534800	-1.36368100
6	-1.28286100	-2.07832600	-1.66414600	6	-6.09258300	-0.74469600	-0.06959900

6	-6.18729300	-2.11447700	0.21141400
1	-5.34729400	-2.77097300	-0.02630500
6	-7.34075200	-2.63415800	0.80362700
1	-7.40918800	-3.70369700	1.01544600
6	-8.40552300	-1.78718600	1.12311900
1	-9.30880800	-2.19372900	1.58380000
6	-8.31384900	-0.41888400	0.84966900
1	-9.14535000	0.24522800	1.09660400
6	-7.16232400	0.10191400	0.25601500
1	-7.08152200	1.17113400	0.04800300
6	-2.77727800	1.88003200	1.68572900
1	-3.05380600	2.57715800	2.48542200
6	-1.28405100	2.10928500	1.24872000
1	-0.77938100	2.97346800	1.69126100

^{3}TSB-concave

E = -5781.76900980 G = -5780.803574

26	4.05047300	-2.10004100	-0.40141600
15	-0.65864700	-1.49322400	-0.53180400
15	2.01419800	0.62553500	0.66757200
6	4.56433200	-2.16573700	-2.41326500
6	4.94902400	-0.91286500	-1.85429800
6	5.85968300	-1.16811200	-0.78479900
6	6.03826800	-2.58061900	-0.68572300
6	5.23445000	-3.19880400	-1.69005300
6	2.55393000	-1.11204000	0.62899400
6	3.47909100	-1.72923100	1.54051600
6	3.47916700	-3.12973000	1.29554700
6	2.57833600	-3.39227200	0.22253200
6	2.00267400	-2.15485400	-0.20904500
6	0.98001000	-1.99118400	-1.30514300
6	0.93151300	-3.19493600	-2.25328100
6	3.18258100	1.57050500	-0.36491000
6	4.50071500	1.82796300	0.04172300
6	5.36671100	2.52601400	-0.80024000
6	4.92816100	2.95751600	-2.05701700
6	3.62147800	2.68758800	-2.47088600
6	2.74747200	1.99815300	-1.62743700
6	2.31797100	1.11586400	2.40825500
6	2.85748200	2.36546100	2.75362100
6	2.95432500	2.74934200	4.09435900
6	2.51240400	1.89569300	5.10714300
6	1.97522300	0.64841900	4.77315900
6	1.87686400	0.26102000	3.43630200
1	3.87680200	-2.30674700	-3.24502100
1	4.60241400	0.06276100	-2.18087300
1	6.32918600	-0.41869200	-0.15279800
1	6.65680100	-3.09834300	0.04519700
1	5.13497600	-4.26943200	-1.85984800
1	4.06408200	-1.20874900	2.29461600
1	4.09291400	-3.86796100	1.80783800
1	2.39830300	-4.36789400	-0.22080800
1	1.24901400	-1.10006500	-1.89635500
1	0.58910600	-4.10692600	-1.75219900
1	1.94161200	-3.38460900	-2.64287900
1	0.26996300	-3.01123700	-3.10730800
1	4.85481400	1.47742900	1.01243800
1	6.39102500	2.72829900	-0.47932000
1	3.27343700	3.02636000	-3.44841900
1	1.71932500	1.81302400	-1.94126600
1	3.21643100	3.04024400	1.97807400
1	3.38391100	3.72166500	4.34565100
1	1.63404700	-0.03039800	5.55795400
1	1.46287500	-0.71755600	3.19186500
27	-0.34997600	0.72164200	0.09115300
6	-0.80378400	2.86579900	-0.67783400
6	-1.15928800	-2.72465200	0.76505200
6	-0.93616000	-4.21257600	0.43783100
6	-0.56360600	-2.39608600	2.14587400
1	-2.24403000	-2.54438200	0.83624500
6	-1.57595800	-5.09418100	1.51859700
1	0.14411700	-4.41675700	0.40691000
1	-1.34462900	-4.47348100	-0.54658600
6	-1.18872000	-3.27943000	3.23015600
1	0.52786500	-2.55021900	2.11933400
1	-0.73826100	-1.33998600	2.38390500
6	-1.02597900	-4.76693600	2.90896200
1	-1.40582200	-6.15535500	1.27529500
1	-2.66970000	-4.94053600	1.50899700
1	-0.73550200	-3.04396800	4.20714300
1	-2.26050600	-3.02799700	3.30659700
1	-1.52648800	-5.38189800	3.67363300
1	0.04645400	-5.03197700	2.94489800
6	-1.97267900	-1.47031500	-1.85589100
6	-1.49307500	-0.75217500	-3.13314600
6	-2.65942400	-2.80942000	-2.17583700
1	-2.73113800	-0.81755800	-1.39556700

6	-2.65356900	-0.56592600	-4.11716100
1	-0.70846500	-1.35007000	-3.62396800
1	-1.05623700	0.22225800	-2.88164600
6	-3.79587400	-2.61684500	-3.18993400
1	-1.92312200	-3.51790000	-2.58790100
1	-3.06192900	-3.26281500	-1.25815000
6	-3.32328300	-1.89944100	-4.45632000
1	-2.28534700	-0.07241800	-5.03059200
1	-3.39535200	0.12150300	-3.67207300
1	-4.23299000	-3.59717200	-3.43789100
1	-4.60355400	-2.02891200	-2.72064500
1	-4.16970500	-1.74321900	-5.14369700
1	-2.60094600	-2.54206300	-4.99208200
8	-0.64179800	1.78673600	-1.42945600
6	0.30491500	3.88807900	-0.71643800
6	0.44162200	4.57284000	-1.93605200
6	1.20618300	4.16084900	0.31483600
6	1.46776900	5.49925200	-2.11704800
1	-0.25793700	4.36688600	-2.74960000
6	2.23446700	5.08968800	0.13494100
1	1.13378000	3.63461400	1.26526100
6	2.37169600	5.75780900	-1.08150300
1	1.56293800	6.02286700	-3.07095100
1	2.93522000	5.28680400	0.94942400
1	3.17750000	6.48092200	-1.22334800
1	2.59125600	2.19749600	6.15368300
1	5.60873600	3.50359800	-2.71387700
1	-1.80196000	3.32046500	-0.77339400
8	-3.98176300	2.75409000	-0.74611700
8	-3.72308600	-1.14626500	1.62866200
7	-4.06226600	0.68988800	0.27841500
6	-3.66777500	2.02454700	0.16362600
6	-1.14701400	1.09062600	1.85890500
1	-0.40314800	0.85187000	2.62268900
6	-2.65960900	1.01383700	2.12666600
1	-2.91246400	1.00457900	3.19601500
6	-3.52063500	0.01916000	1.37198000
6	-5.05275600	0.08666100	-0.61687500
1	-4.84979400	-0.99089400	-0.62708600
1	-4.87321500	0.50127200	-1.61695300
6	-6.46631900	0.35971700	-0.15865900
6	-7.08220300	-0.49581700	0.76616900
1	-6.54136800	-1.37207100	1.13221900
6	-8.37533700	-0.22701500	1.21955900
1	-8.85166200	-0.90047900	1.93574300
6	-9.06091500	0.89886400	0.75344600
1	-10.07412700	1.10647500	1.10504000
6	-8.44972700	1.75628900	-0.16586500
1	-8.98368100	2.63563500	-0.53316500
6	-7.15596400	1.48947600	-0.61999800
1	-6.67143900	2.16263500	-1.33091400
6	-2.80240400	2.34822600	1.37189400
1	-3.22339700	3.20468400	1.91624800
6	-1.25917400	2.41137700	1.21579100
1	-0.72191800	3.27015200	1.61918100

^{3}TSC-concave

E = -5781.75957065 G = -5780.794948

26	-0.47142000	3.94333600	-1.41201500
15	-2.39493600	-0.36971400	-0.42914700
15	0.40580900	1.22528400	0.83871100
6	0.46291400	5.76453800	-1.09099600
6	-0.38522100	5.83494600	-2.23716900
6	-1.71781500	5.54065600	-1.82014000
6	-1.69552400	5.28921800	-0.41517500
6	-0.34602700	5.43065900	0.03530600
6	0.01798500	2.09811500	-0.68400500
6	1.00132400	2.51761700	-1.64889200
6	0.33519100	2.73534200	-2.88494900
6	-1.04887800	2.43828800	-2.70954900
6	-1.26124800	2.01968800	-1.36231500
6	-2.53201600	1.48441500	-0.76405600
6	-3.81028000	1.93737600	-1.46970500
6	-0.71326100	1.65169000	2.21890800
6	-0.77451800	0.73878400	3.28595300
6	-1.53657000	1.04108300	4.41590300
6	-2.25365100	2.24044200	4.47866100
6	-2.20395200	3.14107600	3.41022200
6	-1.42908000	2.85240600	2.28455600
6	2.00896800	1.75691900	1.50262900
6	2.66035900	0.88122400	2.38309600
6	3.81433000	1.29914600	3.04806900
6	4.33033200	2.57792100	2.81948300
6	3.68288400	3.44674800	1.93542500
6	2.51551500	3.04577000	1.28489200
1	1.53971700	5.92340000	-1.08224800
1	-0.06710800	6.05127000	-3.25531500

1	-2.59414800	5.50058900	-2.46453300
1	-2.55896000	5.04161100	0.19992300
1	0.00830400	5.30230800	1.05628100
1	2.06896200	2.60941500	-1.46972700
1	0.79876800	3.10488700	-3.79761500
1	-1.82046200	2.53942000	-3.46893200
1	-2.57536800	1.85628500	0.26788100
1	-3.86232000	1.59101200	-2.50969000
1	-3.85617100	3.03733700	-1.48174800
1	-4.70551900	1.56680300	-0.95105800
1	-0.23886900	-0.21252000	3.23202700
1	-1.58112600	0.33124700	5.24451400
1	-2.76539900	4.07693900	3.45645200
1	-1.37737600	3.54796800	1.45053100
1	2.26958800	-0.12686400	2.54505300
1	4.31886400	0.61958300	3.73818500
1	4.08510000	4.44660200	1.75840700
1	1.99745200	3.73122200	0.61436500
6	-2.81624000	-1.36808400	-1.97556100
6	-4.27997100	-1.82047900	-2.12554200
6	-2.33827000	-0.73897300	-3.29806500
1	-2.22320100	-2.28361200	-1.81088800
6	-4.42415500	-2.80905900	-3.29075200
1	-4.92337000	-0.94041500	-2.29884600
1	-4.63855400	-2.30544400	-1.20845000
6	-2.45662800	-1.73915100	-4.45324400
1	-2.96067200	0.13966200	-3.52544100
1	-1.30907300	-0.36508600	-3.23208700
6	-3.89498400	-2.23767600	-4.60744400
1	-5.48048100	-3.10437900	-3.39402200
1	-3.86708400	-3.72948100	-3.03860400
1	-2.10533200	-1.27213100	-5.38738300
1	-1.78557400	-2.59630300	-4.25986200
1	-3.95850400	-2.99233300	-5.40736900
1	-4.53578500	-1.39447200	-4.92245500
6	-3.82835000	-0.68306700	0.73091300
6	-3.64688000	-2.06744100	1.36936100
6	-4.02603400	0.39127700	1.80977700
1	-4.73283200	-0.69373800	0.10268500
6	-4.79835900	-2.42099600	2.31177900
1	-2.69869400	-2.06600100	1.92943800
1	-3.53806300	-2.84275600	0.59728200
6	-5.17841200	0.02865700	2.75372500
1	-3.10450600	0.49209900	2.39547900
1	-4.22819800	1.37292000	1.35853200
6	-4.98084600	-1.34947200	3.38804300
1	-4.60944200	-3.40605700	2.76592000
1	-5.73158200	-2.52004400	1.72821900
1	-5.26646600	0.80516900	3.53068700
1	-6.12952300	0.03851100	2.19119500
1	-5.83172700	-1.59728500	4.04236800
1	-4.08300400	-1.32650200	4.03232400
1	5.23856900	2.89961400	3.33362800
1	-2.85511000	2.47143500	5.36041800
27	-0.08689400	-0.90775700	0.31796800
6	0.25285600	-3.18589100	0.57920400
8	0.21748100	-2.27260800	1.55430900
6	-0.94899400	-4.10072900	0.50334800
6	-1.52595400	-4.45061500	1.73669900
6	-1.46245600	-4.68866700	-0.66174100
6	-2.59461800	-5.34350000	1.80062800
1	-1.12145600	-4.00827000	2.64757700
6	-2.55241400	-5.56226600	-0.60132300
1	-1.02445100	-4.48661300	-1.63950900
6	-3.12489400	-5.89128500	0.62916400
1	-3.01956600	-5.60987100	2.77097400
1	-2.94556400	-5.99688800	-1.52316900
1	-3.97040600	-6.58087000	0.67541900
1	1.19779900	-3.75022200	0.54398400
8	3.41839700	-3.53903600	0.46408700
8	3.65943100	0.81298600	-0.93389900
7	3.77109200	-1.32047700	-0.05935400
6	3.17615800	-2.57179100	-0.21807300
6	0.74299700	-0.94215400	-1.45409600
1	0.04767800	-0.42203500	-2.11138000
6	2.23489500	-0.99252500	-1.79421000
1	2.43304300	-0.74978100	-2.84746600
6	3.28228100	-0.33509200	-0.91281500
6	4.91358100	-1.10558600	0.83251400
1	4.90908400	-0.04172200	1.08959400
1	4.73157600	-1.70058400	1.73694000
6	6.21502200	-1.49496800	0.17066500
6	6.90988400	-0.55772200	-0.60778500
1	6.51420000	0.45624700	-0.70707700
6	8.09452600	-0.91831900	-1.25343800
1	8.63340700	-0.18145400	-1.85363300
6	8.59316000	-2.21842300	-1.12687600
1	9.52226900	-2.49927900	-1.62820100

6	7.90314500	-3.15698500	-0.35425900
1	8.29141500	-4.17291800	-0.25150400
6	6.71731000	-2.79814500	0.29121200
1	6.17027300	-3.53198800	0.88734800
6	2.21554100	-2.47759600	-1.39586600
1	2.49585100	-3.21690000	-2.15894000
6	0.68184000	-2.39532000	-1.14512400
1	0.02998400	-3.01913100	-1.75501300

^{3}TSD-concave

E = -5781.75658865 G = -5780.79224

26	-2.60602300	-3.37986500	0.68456700
15	-2.29523100	1.43504700	0.60359500
15	-0.32496300	-1.03807300	-0.86422100
6	-2.43638800	-5.33242900	0.01418000
6	-3.38428000	-5.25038700	1.07871300
6	-4.42541100	-4.36162200	0.67635000
6	-4.12331000	-3.89497400	-0.63826100
6	-2.89331200	-4.49741800	-1.04801200
6	-1.26933300	-1.83851800	0.43950000
6	-0.74193100	-2.80560700	1.36543100
6	-1.64895800	-2.91187900	2.45287600
6	-2.73197600	-2.01380500	2.22364400
6	-2.50278700	-1.31768900	0.99892400
6	-3.29620100	-0.15710600	0.45892300
6	-4.73661100	-0.07894400	0.96467400
6	-1.35813800	-0.69748700	-2.34317400
6	-0.87279900	0.24609200	-3.26509000
6	-1.57533400	0.49106800	-4.44654700
6	-2.76957900	-0.18645200	-4.70912000
6	-3.25831300	-1.11786600	-3.78780400
6	-2.55175600	-1.37844000	-2.61217600
6	0.92029100	-2.15732700	-1.57180000
6	1.93477800	-1.57122300	-2.34322100
6	2.82516700	-2.37874100	-3.05166000
6	2.72096000	-3.77065300	-2.97500100
6	1.71505100	-4.35402800	-2.19855200
6	0.80590200	-3.55257600	-1.50572500
1	-1.52072100	-5.92096200	0.01917800
1	-3.31239300	-5.75772100	2.03894300
1	-5.29043300	-4.07902900	1.27353600
1	-4.73325100	-3.20927600	-1.22367200
1	-2.38686200	-4.34367700	-1.99894000
1	0.20333400	-3.32907600	1.26431400
1	-1.55065000	-3.59367300	3.29531600
1	-3.59851000	-1.89045400	2.86819100
1	-3.33449500	-0.26389200	-0.63213600
1	-4.79547800	0.07324300	2.05036900
1	-5.26117200	-1.01822800	0.73045200
1	-5.28365100	0.74319600	0.48191900
1	0.03656000	0.81119600	-3.05506400
1	-1.19374600	1.22675100	-5.15763600
1	-4.19047000	-1.65084400	-3.98904100
1	-2.91903000	-2.10532800	-1.89340200
1	2.03236900	-0.48371100	-2.38703400
1	3.61207000	-1.91994500	-3.65404500
1	1.63087100	-5.44141400	-2.13993400
1	0.00473700	-4.00819200	-0.92551900
6	-2.43075800	2.11859400	2.35131400
6	-3.61621400	3.06142900	2.62916700
6	-2.37151700	1.03412600	3.44490900
1	-1.50980000	2.72501600	2.43730700
6	-3.50059800	3.68476900	4.02725700
1	-4.56052900	2.49667100	2.55034600
1	-3.66552200	3.86619500	1.88347000
6	-2.22296900	1.66198500	4.83435400
1	-3.30598000	0.45517300	3.41939300
1	-1.57185900	0.30287700	3.26339500
6	-3.37371500	2.62784100	5.12653700
1	-4.37104800	4.33384100	4.21324100
1	-2.61274200	4.34251800	4.04911300
1	-2.17950200	0.86825300	5.59730600
1	-1.26034600	2.20259600	4.88905900
1	-3.23430300	3.10973100	6.10707600
1	-4.31634100	2.05490300	5.19163500
6	-3.20950100	2.66054100	-0.46139300
6	-2.35855600	3.93543200	-0.61467500
6	-3.59029200	2.10354100	-1.84108400
1	-4.14046300	2.91258900	0.07199500
6	-3.08031800	4.99432900	-1.45404900
1	-1.41739100	3.65789200	-1.11571900
1	-2.09173800	4.34919200	0.37209000
6	-4.29680500	3.16392200	-2.69323000
1	-2.68141600	1.76877700	-2.35994600
1	-4.24232800	1.22329100	-1.74446500
6	-3.45846700	4.43711300	-2.82868500
1	-2.43877600	5.88375600	-1.55924700

1	-3.99247500	5.32511200	-0.92531300
1	-4.52185800	2.74049000	-3.68533600
1	-5.26908000	3.41378300	-2.23124400
1	-4.00271600	5.19432100	-3.41502700
1	-2.53534800	4.20606700	-3.39033300
1	3.42410900	-4.40193100	-3.52244300
1	-3.32052000	0.01247800	-5.63089500
27	0.01341300	1.07057600	-0.10075900
6	1.16905300	3.10544000	-0.37596600
8	0.60776300	2.34914200	-1.32425300
6	2.52218500	3.66687100	-0.68356600
6	3.15507200	4.53863000	0.21385700
6	3.13674900	3.36421400	-1.90363300
6	4.39942200	5.08288700	-0.09634500
1	2.67286200	4.79776000	1.16097300
6	4.37831900	3.91739600	-2.21699200
1	2.62562300	2.69939500	-2.60056500
6	5.01543700	4.76908600	-1.31250200
1	4.89073300	5.75750600	0.60821400
1	4.85398800	3.67965200	-3.17107400
1	5.99124700	5.19536800	-1.55540300
1	0.49492800	3.88058200	0.03347900
8	4.69502600	1.59955200	-0.04327200
8	2.29827300	-2.08568700	1.20804800
7	3.62170300	-0.39057900	0.37319300
6	3.81924600	0.99206600	0.51470600
6	0.76868600	0.87894900	1.69085400
1	-0.05596600	0.85821900	2.40102900
6	2.09270500	0.21382700	2.03508500
1	2.18089800	-0.05854100	3.09686600
6	2.63071200	-0.92249200	1.18291900
6	4.56425100	-1.23248100	-0.36964700
1	4.01037200	-2.12286900	-0.68153400
1	4.86986800	-0.66560400	-1.25785200
6	5.75698600	-1.61192600	0.47785100
6	5.71043800	-2.76895200	1.26943600
1	4.81746300	-3.39840000	1.24678200
6	6.79307100	-3.11081000	2.08286200
1	6.75127600	-4.01652700	2.69243800
6	7.93061300	-2.29868000	2.11210900
1	8.77943900	-2.56823900	2.74482500
6	7.98147300	-1.14328100	1.32677200
1	8.86986200	-0.50772400	1.34568200
6	6.89898400	-0.79920700	0.51373100
1	6.92995900	0.10906200	-0.09172900
6	2.82399600	1.48518700	1.56272000
1	3.37873300	2.05012800	2.32335300
6	1.43969400	2.15234300	1.26008200
1	1.20585800	3.02592800	1.87692400

■ Proof of Stereochemistry: X-ray Characterization Data

(1*R*,5*S*,6*R*)-3-benzyl-6-((*R*)-hydroxy(4-(trifluoromethyl)phenyl)methyl)-3-azabicyclo[3.2.0]heptane-2,4-dione (3b)

The absolute configurations of **3a-3ah** and **5a-5e** are assigned as (*R*, *S*, *R*) by analogy to that of **3b (**CCDC number: 2373832), whose absolute configuration was determined to be (1*R*,5*S*,6*R*) by X-ray crystallographic analysis (vide infra)

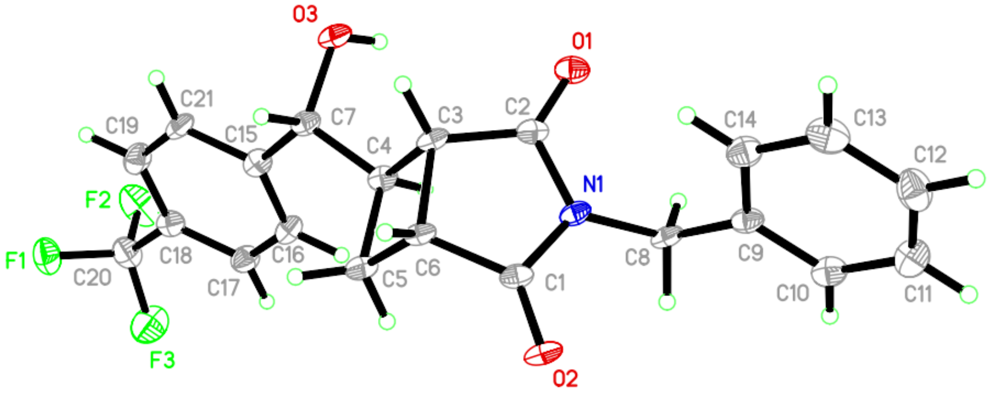

Table S9. Crystal data and structure refinement for t.

Identification code	t	
Empirical formula	C21 H18 F3 N O3	
Formula weight	389.36	
Temperature	100 (2) K	
Wavelength	1.54184 Å	
Crystal system	Monoclinic	
Space group	C2	
Unit cell dimensions	a = 20.1984 (5) Å	α= 90°.
	b = 6.0121 (5) Å	β= 92.317 (3)°.
	c = 29.4619 (10) Å	γ = 90°.
Volume	3574.8 (3) Å^3	
Z	8	
Density (calculated)	1.447 Mg/m^3	
Absorption coefficient	1.002 mm^{-1}	
F (000)	1616	
Crystal size	0.200 x 0.180 x 0.150 mm^3	
Theta range for data collection	3.002 to 75.937°.	
Index ranges	-25<=h<=25, -7<=k<=5, -36<=l<=37	

Reflections collected	29298
Independent reflections	6698 [R (int) = 0.0340]
Completeness to theta = 67.684°	99.8 %
Absorption correction	Semi-empirical from equivalents
Max. and min. transmission	1.00000 and 0.87673
Refinement method	Full-matrix least-squares on F^2
Data / restraints / parameters	6698 / 1 / 513
Goodness-of-fit on F^2	1.136
Final R indices [I>2sigma (I)]	R1 = 0.0551, wR2 = 0.1118
R indices (all data)	R1 = 0.0601, wR2 = 0.1138
Absolute structure parameter	0.03 (6)
Extinction coefficient	n/a
Largest diff. peak and hole	0.304 and -0.282 e.Å^{-3}

Table S10. Atomic coordinates (x 10^4) and equivalent isotropic displacement parameters (Å^{2}x 10^3) for t. U (eq) is defined as one third of the trace of the orthogonalized U^{ij} tensor.

	x	y	z	U (eq)
N (1)	-1370 (2)	5726 (6)	-135 (1)	20 (1)
C (1)	-1802 (2)	6315 (7)	207 (1)	22 (1)
O (1)	-746 (1)	2753 (6)	-351 (1)	26 (1)
F (1)	1073 (1)	2552 (6)	2956 (1)	40 (1)
C (30)	6121 (2)	4761 (8)	5106 (1)	22 (1)
C (29)	5544 (2)	2566 (8)	3985 (1)	23 (1)
C (28)	4760 (2)	5181 (8)	3541 (2)	29 (1)
C (27)	4321 (2)	5588 (9)	3175 (2)	31 (1)
C (26)	4217 (2)	3996 (9)	2844 (2)	33 (1)
C (25)	4541 (2)	1957 (9)	2878 (2)	33 (1)
C (6)	-1774 (2)	4507 (7)	559 (1)	21 (1)
O (6)	5237 (2)	925 (5)	4264 (1)	27 (1)
F (6)	3828 (1)	6497 (6)	2276 (1)	43 (1)
C (15)	-52 (2)	2061 (8)	1433 (1)	23 (1)
C (2)	-1105 (2)	3609 (7)	-77 (1)	22 (1)
N (2)	6387 (2)	6869 (6)	5164 (1)	23 (1)
O (2)	-2107 (1)	8058 (5)	203 (1)	25 (1)
F (2)	1880 (1)	2551 (6)	2512 (1)	51 (1)
C (20)	1281 (2)	3427 (10)	2569 (2)	35 (1)
C (31)	6310 (2)	3917 (7)	4653 (1)	23 (1)

C (35)	6807 (2)	7459 (8)	4820 (1)	22 (1)
C (23)	5087 (2)	3150 (8)	3582 (1)	26 (1)
C (9)	-1622 (2)	7321 (7)	-909 (1)	22 (1)
C (14)	-2026 (2)	5536 (8)	-1044 (2)	28 (1)
C (42)	7088 (2)	6511 (8)	6049 (2)	27 (1)
C (34)	6783 (2)	5696 (7)	4466 (1)	23 (1)
C (21)	96 (2)	430 (8)	1761 (2)	25 (1)
C (19)	523 (2)	893 (8)	2130 (2)	29 (1)
C (18)	812 (2)	2971 (8)	2173 (1)	25 (1)
C (17)	680 (2)	4602 (8)	1846 (1)	28 (1)
C (16)	247 (2)	4133 (8)	1481 (2)	26 (1)
C (8)	-1171 (2)	7248 (7)	-488 (1)	22 (1)
C (7)	-522 (2)	1439 (7)	1037 (1)	22 (1)
C (38)	6685 (2)	10129 (8)	6239 (2)	28 (1)
C (37)	6678 (2)	8315 (7)	5945 (2)	24 (1)
C (10)	-1617 (2)	9226 (8)	-1176 (2)	26 (1)
C (13)	-2413 (2)	5686 (9)	-1442 (2)	34 (1)
C (5)	-1275 (2)	4987 (8)	965 (1)	24 (1)
O (5)	5764 (1)	3889 (5)	5381 (1)	26 (1)
F (5)	3104 (1)	4475 (6)	2584 (1)	50 (1)
C (3)	-1301 (2)	2763 (7)	372 (1)	20 (1)
O (3)	-222 (2)	-194 (5)	754 (1)	26 (1)
F (3)	1371 (2)	5595 (6)	2648 (1)	51 (1)
C (41)	7498 (2)	6534 (9)	6440 (2)	31 (1)
C (24)	4971 (2)	1570 (9)	3244 (2)	30 (1)
C (32)	5779 (2)	4507 (8)	4274 (1)	22 (1)
C (22)	3735 (2)	4463 (11)	2460 (2)	38 (1)
C (36)	6216 (2)	8361 (8)	5531 (1)	24 (1)
C (33)	6273 (2)	6147 (8)	4062 (1)	25 (1)
C (39)	7096 (2)	10150 (8)	6625 (2)	31 (1)
C (11)	-2004 (2)	9355 (9)	-1572 (2)	32 (1)
C (4)	-768 (2)	3391 (7)	753 (1)	20 (1)
O (4)	7115 (1)	9207 (5)	4820 (1)	27 (1)
F (4)	3767 (2)	3030 (6)	2114 (1)	51 (1)
C (12)	-2402 (2)	7598 (10)	-1708 (2)	35 (1)
C (40)	7500 (2)	8350 (9)	6729 (2)	32 (1)

Table S11. Bond lengths [Å] and angles [°] for t.

N (1)-C (2)	1.388 (5)
N (1)-C (1)	1.404 (5)
N (1)-C (8)	1.454 (5)
C (1)-O (2)	1.215 (5)
C (1)-C (6)	1.502 (6)
O (1)-C (2)	1.219 (5)
F (1)-C (20)	1.339 (6)
C (30)-O (5)	1.225 (5)
C (30)-N (2)	1.385 (6)
C (30)-C (31)	1.492 (6)
C (29)-O (6)	1.440 (5)
C (29)-C (32)	1.509 (6)
C (29)-C (23)	1.516 (6)
C (28)-C (27)	1.388 (6)
C (28)-C (23)	1.391 (6)
C (27)-C (26)	1.378 (7)
C (26)-C (25)	1.392 (7)
C (26)-C (22)	1.488 (7)
C (25)-C (24)	1.376 (6)
C (6)-C (3)	1.535 (6)
C (6)-C (5)	1.561 (6)
F (6)-C (22)	1.354 (7)
C (15)-C (16)	1.390 (6)
C (15)-C (21)	1.400 (6)
C (15)-C (7)	1.520 (6)
C (2)-C (3)	1.486 (6)
N (2)-C (35)	1.396 (5)
N (2)-C (36)	1.456 (5)
F (2)-C (20)	1.337 (6)
C (20)-F (3)	1.335 (6)
C (20)-C (18)	1.498 (6)
C (31)-C (34)	1.549 (6)
C (31)-C (32)	1.558 (6)
C (35)-O (4)	1.221 (5)
C (35)-C (34)	1.484 (6)
C (23)-C (24)	1.390 (6)

C (9)-C (10)	1.389 (6)
C (9)-C (14)	1.396 (6)
C (9)-C (8)	1.509 (6)
C (14)-C (13)	1.385 (6)
C (42)-C (41)	1.390 (6)
C (42)-C (37)	1.392 (6)
C (34)-C (33)	1.566 (6)
C (21)-C (19)	1.389 (6)
C (19)-C (18)	1.383 (7)
C (18)-C (17)	1.393 (6)
C (17)-C (16)	1.387 (6)
C (7)-O (3)	1.438 (5)
C (7)-C (4)	1.513 (6)
C (38)-C (39)	1.383 (6)
C (38)-C (37)	1.392 (6)
C (37)-C (36)	1.505 (6)
C (10)-C (11)	1.381 (6)
C (13)-C (12)	1.393 (7)
C (5)-C (4)	1.552 (6)
F (5)-C (22)	1.339 (5)
C (3)-C (4)	1.569 (5)
C (41)-C (40)	1.384 (7)
C (32)-C (33)	1.551 (6)
C (22)-F (4)	1.338 (6)
C (39)-C (40)	1.382 (7)
C (11)-C (12)	1.377 (7)

C (2)-N (1)-C (1)	113.0 (4)
C (2)-N (1)-C (8)	123.2 (3)
C (1)-N (1)-C (8)	123.5 (4)
O (2)-C (1)-N (1)	122.7 (4)
O (2)-C (1)-C (6)	129.4 (4)
N (1)-C (1)-C (6)	107.7 (3)
O (5)-C (30)-N (2)	123.0 (4)
O (5)-C (30)-C (31)	128.8 (4)
N (2)-C (30)-C (31)	108.1 (4)
O (6)-C (29)-C (32)	110.0 (3)
O (6)-C (29)-C (23)	110.0 (3)

C (32)-C (29)-C (23)	115.5 (4)
C (27)-C (28)-C (23)	120.5 (5)
C (26)-C (27)-C (28)	120.2 (5)
C (27)-C (26)-C (25)	120.1 (4)
C (27)-C (26)-C (22)	118.9 (5)
C (25)-C (26)-C (22)	120.9 (5)
C (24)-C (25)-C (26)	119.1 (5)
C (1)-C (6)-C (3)	104.7 (3)
C (1)-C (6)-C (5)	113.7 (4)
C (3)-C (6)-C (5)	90.5 (3)
C (16)-C (15)-C (21)	118.5 (4)
C (16)-C (15)-C (7)	123.6 (4)
C (21)-C (15)-C (7)	117.9 (4)
O (1)-C (2)-N (1)	122.9 (4)
O (1)-C (2)-C (3)	129.1 (4)
N (1)-C (2)-C (3)	107.9 (4)
C (30)-N (2)-C (35)	112.7 (4)
C (30)-N (2)-C (36)	123.6 (3)
C (35)-N (2)-C (36)	123.6 (4)
F (3)-C (20)-F (2)	106.8 (4)
F (3)-C (20)-F (1)	106.3 (4)
F (2)-C (20)-F (1)	105.5 (4)
F (3)-C (20)-C (18)	113.1 (4)
F (2)-C (20)-C (18)	112.2 (4)
F (1)-C (20)-C (18)	112.4 (4)
C (30)-C (31)-C (34)	105.4 (4)
C (30)-C (31)-C (32)	111.7 (3)
C (34)-C (31)-C (32)	90.3 (3)
O (4)-C (35)-N (2)	122.8 (4)
O (4)-C (35)-C (34)	128.2 (4)
N (2)-C (35)-C (34)	108.9 (4)
C (24)-C (23)-C (28)	118.2 (4)
C (24)-C (23)-C (29)	118.9 (4)
C (28)-C (23)-C (29)	123.0 (4)
C (10)-C (9)-C (14)	119.3 (4)
C (10)-C (9)-C (8)	118.1 (4)
C (14)-C (9)-C (8)	122.5 (4)
C (13)-C (14)-C (9)	119.6 (5)

C (41)-C (42)-C (37)	120.3 (4)
C (35)-C (34)-C (31)	104.4 (3)
C (35)-C (34)-C (33)	114.3 (4)
C (31)-C (34)-C (33)	89.6 (3)
C (19)-C (21)-C (15)	120.6 (4)
C (18)-C (19)-C (21)	119.9 (4)
C (19)-C (18)-C (17)	120.4 (4)
C (19)-C (18)-C (20)	119.2 (4)
C (17)-C (18)-C (20)	120.5 (4)
C (16)-C (17)-C (18)	119.3 (4)
C (17)-C (16)-C (15)	121.3 (4)
N (1)-C (8)-C (9)	115.6 (3)
O (3)-C (7)-C (4)	110.1 (3)
O (3)-C (7)-C (15)	110.5 (3)
C (4)-C (7)-C (15)	114.5 (4)
C (39)-C (38)-C (37)	120.7 (4)
C (42)-C (37)-C (38)	118.8 (4)
C (42)-C (37)-C (36)	122.5 (4)
C (38)-C (37)-C (36)	118.7 (4)
C (11)-C (10)-C (9)	120.6 (4)
C (14)-C (13)-C (12)	120.6 (5)
C (4)-C (5)-C (6)	89.6 (3)
C (2)-C (3)-C (6)	106.1 (3)
C (2)-C (3)-C (4)	110.8 (3)
C (6)-C (3)-C (4)	89.9 (3)
C (40)-C (41)-C (42)	120.3 (4)
C (25)-C (24)-C (23)	121.9 (5)
C (29)-C (32)-C (33)	117.3 (3)
C (29)-C (32)-C (31)	114.8 (4)
C (33)-C (32)-C (31)	89.8 (3)
F (4)-C (22)-F (5)	106.7 (4)
F (4)-C (22)-F (6)	105.3 (4)
F (5)-C (22)-F (6)	104.7 (4)
F (4)-C (22)-C (26)	114.0 (5)
F (5)-C (22)-C (26)	113.2 (4)
F (6)-C (22)-C (26)	112.2 (4)
N (2)-C (36)-C (37)	115.7 (3)
C (32)-C (33)-C (34)	89.9 (3)

C (40)-C (39)-C (38)	120.3 (4)
C (12)-C (11)-C (10)	120.4 (5)
C (7)-C (4)-C (5)	117.6 (3)
C (7)-C (4)-C (3)	114.2 (4)
C (5)-C (4)-C (3)	89.6 (3)
C (11)-C (12)-C (13)	119.5 (4)
C (39)-C (40)-C (41)	119.6 (4)

Symmetry transformations used to generate equivalent atoms:

Table S12. Anisotropic displacement parameters ($Å^2$x 10^3) for t. The anisotropic displacement factor exponent takes the form: $-2\pi^2[h^2 a^{*2}U^{11} + ... + 2 h k a^* b^* U^{12}]$

	U^{11}	U^{22}	U^{33}	U^{23}	U^{13}	U^{12}
N (1)	17 (2)	13 (2)	29 (2)	0 (1)	1 (1)	-3 (1)
C (1)	19 (2)	15 (2)	30 (2)	-4 (2)	-1 (2)	-1 (2)
O (1)	20 (1)	24 (2)	33 (2)	-5 (1)	4 (1)	1 (1)
F (1)	43 (2)	51 (2)	27 (1)	6 (1)	2 (1)	3 (1)
C (30)	12 (2)	25 (3)	30 (2)	-2 (2)	-3 (2)	6 (2)
C (29)	22 (2)	18 (2)	30 (2)	-1 (2)	5 (2)	1 (2)
C (28)	28 (2)	28 (3)	30 (2)	-4 (2)	-1 (2)	6 (2)
C (27)	24 (2)	36 (3)	33 (2)	-2 (2)	1 (2)	7 (2)
C (26)	26 (2)	46 (4)	27 (2)	3 (2)	2 (2)	-1 (2)
C (25)	26 (2)	37 (3)	34 (3)	-5 (2)	-2 (2)	1 (2)
C (6)	18 (2)	12 (2)	31 (2)	-2 (2)	2 (2)	0 (2)
O (6)	25 (2)	20 (2)	37 (2)	0 (1)	3 (1)	-1 (1)
F (6)	31 (1)	55 (2)	44 (2)	17 (2)	-2 (1)	1 (1)
C (15)	16 (2)	23 (3)	30 (2)	2 (2)	3 (2)	0 (2)
C (2)	22 (2)	18 (2)	27 (2)	-4 (2)	-2 (2)	2 (2)
N (2)	23 (2)	19 (2)	27 (2)	0 (2)	2 (1)	0 (2)
O (2)	23 (1)	12 (2)	42 (2)	-2 (1)	2 (1)	3 (1)
F (2)	25 (1)	86 (3)	41 (2)	-11 (2)	-5 (1)	8 (2)
C (20)	34 (2)	42 (3)	29 (2)	-1 (2)	2 (2)	4 (2)
C (31)	23 (2)	18 (2)	28 (2)	1 (2)	0 (2)	1 (2)
C (35)	17 (2)	20 (3)	29 (2)	2 (2)	0 (2)	8 (2)
C (23)	18 (2)	29 (3)	29 (2)	-1 (2)	4 (2)	0 (2)
C (9)	18 (2)	16 (2)	30 (2)	-2 (2)	5 (2)	-2 (2)

C (14)	25 (2)	25 (3)	33 (2)	-1 (2)	0 (2)	1 (2)
C (42)	27 (2)	18 (2)	35 (2)	0 (2)	5 (2)	1 (2)
C (34)	19 (2)	19 (2)	32 (2)	0 (2)	3 (2)	1 (2)
C (21)	25 (2)	14 (2)	36 (2)	6 (2)	6 (2)	0 (2)
C (19)	26 (2)	30 (3)	31 (2)	8 (2)	-1 (2)	6 (2)
C (18)	20 (2)	24 (3)	30 (2)	-1 (2)	2 (2)	0 (2)
C (17)	30 (2)	22 (3)	30 (2)	0 (2)	-1 (2)	-2 (2)
C (16)	25 (2)	20 (3)	31 (2)	6 (2)	1 (2)	-1 (2)
C (8)	22 (2)	11 (2)	33 (2)	1 (2)	4 (2)	1 (2)
C (7)	19 (2)	17 (2)	29 (2)	0 (2)	1 (2)	-2 (2)
C (38)	27 (2)	19 (3)	39 (3)	-2 (2)	8 (2)	-1 (2)
C (37)	22 (2)	19 (3)	31 (2)	2 (2)	8 (2)	-8 (2)
C (10)	22 (2)	22 (3)	35 (2)	-1 (2)	5 (2)	-5 (2)
C (13)	27 (2)	35 (3)	41 (3)	-9 (2)	-1 (2)	-6 (2)
C (5)	25 (2)	17 (2)	31 (2)	-2 (2)	-1 (2)	4 (2)
O (5)	26 (2)	20 (2)	33 (2)	3 (1)	5 (1)	-4 (1)
F (5)	21 (1)	80 (3)	48 (2)	20 (2)	-1 (1)	-5 (2)
C (3)	18 (2)	11 (2)	32 (2)	0 (2)	-1 (2)	-1 (2)
O (3)	24 (2)	15 (2)	38 (2)	-1 (1)	4 (1)	-1 (1)
F (3)	67 (2)	41 (2)	45 (2)	-1 (2)	-23 (2)	-14 (2)
C (41)	28 (2)	25 (3)	39 (3)	0 (2)	-2 (2)	5 (2)
C (24)	29 (2)	27 (3)	33 (2)	-4 (2)	1 (2)	1 (2)
C (32)	18 (2)	19 (2)	27 (2)	0 (2)	2 (2)	2 (2)
C (22)	23 (2)	57 (4)	34 (3)	6 (3)	2 (2)	-3 (2)
C (36)	24 (2)	17 (2)	31 (2)	1 (2)	5 (2)	4 (2)
C (33)	24 (2)	25 (3)	27 (2)	4 (2)	0 (2)	-2 (2)
C (39)	29 (2)	30 (3)	35 (3)	-6 (2)	2 (2)	-1 (2)
C (11)	26 (2)	32 (3)	37 (3)	5 (2)	3 (2)	-4 (2)
C (4)	16 (2)	18 (2)	27 (2)	-2 (2)	1 (2)	-2 (2)
O (4)	24 (2)	20 (2)	37 (2)	4 (1)	1 (1)	-4 (1)
F (4)	47 (2)	64 (2)	40 (2)	-2 (2)	-12 (1)	-7 (2)
C (12)	28 (2)	49 (4)	28 (2)	0 (2)	-1 (2)	3 (2)
C (40)	31 (2)	33 (3)	32 (3)	-1 (2)	-5 (2)	0 (2)

Table S13. Hydrogen coordinates (x 10^4) and isotropic displacement parameters ($Å^2$x 10^3) for t.

	x	y	z	U (eq)
H (1)	20 (30)	440 (120)	580 (20)	80 (20)
H (29)	5947	1858	3862	28
H (28)	4837	6299	3764	35
H (27)	4092	6966	3154	37
H (25)	4467	848	2652	39
H (6)	-2214	3919	645	25
H (2)	4970 (30)	1540 (110)	4430 (20)	60 (20)
H (31)	6479	2353	4649	27
H (14)	-2035	4226	-864	33
H (42)	7088	5259	5853	32
H (34)	7224	5126	4377	28
H (21)	-97	-1006	1731	30
H (19)	615	-216	2354	35
H (17)	884	6023	1873	33
H (16)	154	5250	1259	31
H (8A)	-721	6829	-578	26
H (8B)	-1143	8765	-358	26
H (7)	-919	727	1168	26
H (38)	6405	11366	6173	34
H (10)	-1346	10450	-1085	31
H (13)	-2689	4474	-1533	41
H (5A)	-1124	6553	982	29
H (5B)	-1430	4477	1262	29
H (3)	-1462	1191	376	24
H (41)	7777	5298	6509	37
H (24)	5195	182	3266	36
H (32)	5397	5318	4402	26
H (36A)	5765	7983	5626	29
H (36B)	6202	9899	5411	29
H (33A)	6111	7702	4048	30
H (33B)	6429	5661	3764	30
H (39)	7101	11407	6821	38

H (11)	-1996	10666	-1752	38
H (4)	-391	4233	625	25
H (12)	-2666	7688	-1982	42
H (40)	7778	8358	6997	39

Table S14. Torsion angles [°] for t.

C (2)-N (1)-C (1)-O (2)	-176.4 (4)
C (8)-N (1)-C (1)-O (2)	9.1 (6)
C (2)-N (1)-C (1)-C (6)	6.4 (4)
C (8)-N (1)-C (1)-C (6)	-168.1 (3)
C (23)-C (28)-C (27)-C (26)	-1.5 (7)
C (28)-C (27)-C (26)-C (25)	1.3 (7)
C (28)-C (27)-C (26)-C (22)	179.0 (4)
C (27)-C (26)-C (25)-C (24)	-0.8 (7)
C (22)-C (26)-C (25)-C (24)	-178.4 (4)
O (2)-C (1)-C (6)-C (3)	-179.4 (4)
N (1)-C (1)-C (6)-C (3)	-2.4 (4)
O (2)-C (1)-C (6)-C (5)	-82.1 (5)
N (1)-C (1)-C (6)-C (5)	94.8 (4)
C (1)-N (1)-C (2)-O (1)	176.2 (4)
C (8)-N (1)-C (2)-O (1)	-9.3 (6)
C (1)-N (1)-C (2)-C (3)	-7.7 (4)
C (8)-N (1)-C (2)-C (3)	166.8 (3)
O (5)-C (30)-N (2)-C (35)	176.5 (4)
C (31)-C (30)-N (2)-C (35)	-7.1 (4)
O (5)-C (30)-N (2)-C (36)	-6.9 (6)
C (31)-C (30)-N (2)-C (36)	169.5 (4)
O (5)-C (30)-C (31)-C (34)	-178.5 (4)
N (2)-C (30)-C (31)-C (34)	5.4 (4)
O (5)-C (30)-C (31)-C (32)	84.9 (5)
N (2)-C (30)-C (31)-C (32)	-91.2 (4)
C (30)-N (2)-C (35)-O (4)	-176.5 (4)
C (36)-N (2)-C (35)-O (4)	6.9 (6)
C (30)-N (2)-C (35)-C (34)	5.8 (4)
C (36)-N (2)-C (35)-C (34)	-170.8 (4)
C (27)-C (28)-C (23)-C (24)	1.2 (7)
C (27)-C (28)-C (23)-C (29)	-176.8 (4)

O (6)-C (29)-C (23)-C (24)	-69.4 (5)
C (32)-C (29)-C (23)-C (24)	165.4 (4)
O (6)-C (29)-C (23)-C (28)	108.5 (5)
C (32)-C (29)-C (23)-C (28)	-16.7 (6)
C (10)-C (9)-C (14)-C (13)	0.3 (6)
C (8)-C (9)-C (14)-C (13)	-178.1 (4)
O (4)-C (35)-C (34)-C (31)	-179.6 (4)
N (2)-C (35)-C (34)-C (31)	-2.0 (4)
O (4)-C (35)-C (34)-C (33)	-83.4 (5)
N (2)-C (35)-C (34)-C (33)	94.2 (4)
C (30)-C (31)-C (34)-C (35)	-2.0 (4)
C (32)-C (31)-C (34)-C (35)	110.6 (3)
C (30)-C (31)-C (34)-C (33)	-117.0 (3)
C (32)-C (31)-C (34)-C (33)	-4.4 (3)
C (16)-C (15)-C (21)-C (19)	1.3 (6)
C (7)-C (15)-C (21)-C (19)	179.9 (4)
C (15)-C (21)-C (19)-C (18)	-1.0 (6)
C (21)-C (19)-C (18)-C (17)	-0.2 (6)
C (21)-C (19)-C (18)-C (20)	-178.9 (4)
F (3)-C (20)-C (18)-C (19)	-161.3 (4)
F (2)-C (20)-C (18)-C (19)	77.9 (6)
F (1)-C (20)-C (18)-C (19)	-40.9 (6)
F (3)-C (20)-C (18)-C (17)	20.1 (6)
F (2)-C (20)-C (18)-C (17)	-100.8 (5)
F (1)-C (20)-C (18)-C (17)	140.4 (4)
C (19)-C (18)-C (17)-C (16)	0.9 (7)
C (20)-C (18)-C (17)-C (16)	179.6 (4)
C (18)-C (17)-C (16)-C (15)	-0.5 (7)
C (21)-C (15)-C (16)-C (17)	-0.6 (6)
C (7)-C (15)-C (16)-C (17)	-179.0 (4)
C (2)-N (1)-C (8)-C (9)	98.1 (4)
C (1)-N (1)-C (8)-C (9)	-87.9 (5)
C (10)-C (9)-C (8)-N (1)	156.2 (4)
C (14)-C (9)-C (8)-N (1)	-25.3 (6)
C (16)-C (15)-C (7)-O (3)	111.4 (5)
C (21)-C (15)-C (7)-O (3)	-67.0 (5)
C (16)-C (15)-C (7)-C (4)	-13.6 (6)
C (21)-C (15)-C (7)-C (4)	168.0 (4)

C (41)-C (42)-C (37)-C (38)	-0.1 (6)
C (41)-C (42)-C (37)-C (36)	-178.9 (4)
C (39)-C (38)-C (37)-C (42)	0.5 (6)
C (39)-C (38)-C (37)-C (36)	179.4 (4)
C (14)-C (9)-C (10)-C (11)	-0.5 (6)
C (8)-C (9)-C (10)-C (11)	178.0 (4)
C (9)-C (14)-C (13)-C (12)	0.2 (7)
C (1)-C (6)-C (5)-C (4)	-101.3 (4)
C (3)-C (6)-C (5)-C (4)	5.0 (3)
O (1)-C (2)-C (3)-C (6)	-178.5 (4)
N (1)-C (2)-C (3)-C (6)	5.7 (4)
O (1)-C (2)-C (3)-C (4)	85.3 (5)
N (1)-C (2)-C (3)-C (4)	-90.4 (4)
C (1)-C (6)-C (3)-C (2)	-2.0 (4)
C (5)-C (6)-C (3)-C (2)	-116.7 (3)
C (1)-C (6)-C (3)-C (4)	109.7 (3)
C (5)-C (6)-C (3)-C (4)	-5.0 (3)
C (37)-C (42)-C (41)-C (40)	0.1 (7)
C (26)-C (25)-C (24)-C (23)	0.5 (7)
C (28)-C (23)-C (24)-C (25)	-0.7 (7)
C (29)-C (23)-C (24)-C (25)	177.4 (4)
O (6)-C (29)-C (32)-C (33)	165.0 (3)
C (23)-C (29)-C (32)-C (33)	-69.7 (5)
O (6)-C (29)-C (32)-C (31)	61.4 (4)
C (23)-C (29)-C (32)-C (31)	-173.3 (3)
C (30)-C (31)-C (32)-C (29)	-128.5 (4)
C (34)-C (31)-C (32)-C (29)	124.7 (4)
C (30)-C (31)-C (32)-C (33)	111.2 (4)
C (34)-C (31)-C (32)-C (33)	4.4 (3)
C (27)-C (26)-C (22)-F (4)	167.3 (4)
C (25)-C (26)-C (22)-F (4)	-15.0 (7)
C (27)-C (26)-C (22)-F (5)	-70.5 (7)
C (25)-C (26)-C (22)-F (5)	107.2 (6)
C (27)-C (26)-C (22)-F (6)	47.7 (6)
C (25)-C (26)-C (22)-F (6)	-134.6 (5)
C (30)-N (2)-C (36)-C (37)	95.3 (5)
C (35)-N (2)-C (36)-C (37)	-88.5 (5)
C (42)-C (37)-C (36)-N (2)	-22.0 (6)

C (38)-C (37)-C (36)-N (2)	159.1 (4)
C (29)-C (32)-C (33)-C (34)	-122.5 (4)
C (31)-C (32)-C (33)-C (34)	-4.4 (3)
C (35)-C (34)-C (33)-C (32)	-101.2 (4)
C (31)-C (34)-C (33)-C (32)	4.4 (3)
C (37)-C (38)-C (39)-C (40)	-0.9 (7)
C (9)-C (10)-C (11)-C (12)	0.2 (7)
O (3)-C (7)-C (4)-C (5)	163.0 (3)
C (15)-C (7)-C (4)-C (5)	-71.8 (5)
O (3)-C (7)-C (4)-C (3)	60.0 (4)
C (15)-C (7)-C (4)-C (3)	-174.8 (3)
C (6)-C (5)-C (4)-C (7)	-122.2 (4)
C (6)-C (5)-C (4)-C (3)	-4.9 (3)
C (2)-C (3)-C (4)-C (7)	-127.5 (4)
C (6)-C (3)-C (4)-C (7)	125.3 (4)
C (2)-C (3)-C (4)-C (5)	112.2 (4)
C (6)-C (3)-C (4)-C (5)	5.0 (3)
C (10)-C (11)-C (12)-C (13)	0.3 (7)
C (14)-C (13)-C (12)-C (11)	-0.5 (7)
C (38)-C (39)-C (40)-C (41)	0.9 (7)
C (42)-C (41)-C (40)-C (39)	-0.5 (7)

Symmetry transformations used to generate equivalent atoms:

Table S15. Hydrogen bonds for t [Å and °].

D-H...A	d (D-H)	d (H...A)	d (D...A)	< (DHA)

(*R*)-6-((*R*)-((1*R*,5*S*,6*R*)-3-benzyl-2,4-dioxo-3-azabicyclo[3.2.0]heptan-6-yl)(hydroxy)methyl)-[1,1':2',1''-terphenyl]-2-carbaldehyde (7a)

The absolute configurations of **7a-7k** and **8a** are assigned as (*R*, *S*, *R*, *Ra*) by analogy to that of **7a (**CCDC number: 2373833), whose absolute configuration was determined to be (1*R*,5*S*,6*R*, *Ra*) by X-ray crystallographic analysis (vide infra).

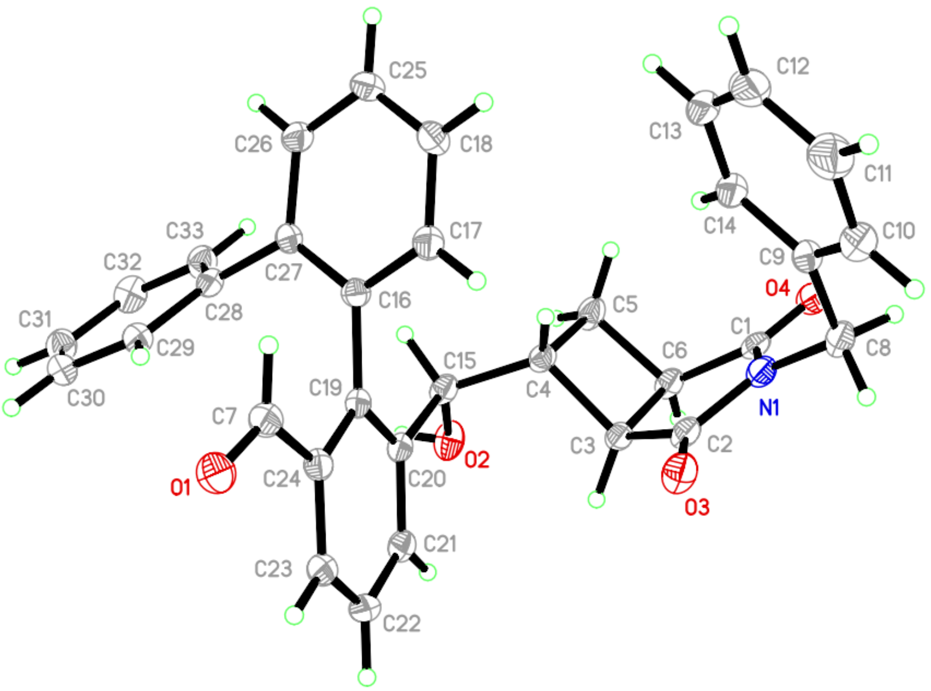

Table S16. Crystal data and structure refinement for t.

Identification code	t	
Empirical formula	C33 H27 N O4	
Formula weight	501.55	
Temperature	100 (2) K	
Wavelength	1.54184 Å	
Crystal system	Orthorhombic	
Space group	$P2_12_12_1$	
Unit cell dimensions	a = 10.5121 (8) Å	α= 90°.
	b = 12.4941 (6) Å	β= 90°.
	c = 20.0428 (5) Å	γ = 90°.
Volume	2632.4 (3) $Å^3$	
Z	4	
Density (calculated)	1.266 Mg/m^3	
Absorption coefficient	0.665 mm^{-1}	
F (000)	1056	
Crystal size	0.180 x 0.160 x 0.140 mm^3	
Theta range for data collection	4.170 to 76.134°.	
Index ranges	-9<=h<=12, -14<=k<=15, -24<=l<=25	
Reflections collected	31952	

Independent reflections	5443 [R (int) = 0.0246]
Completeness to theta = 67.684°	100.0 %
Absorption correction	Semi-empirical from equivalents
Max. and min. transmission	1.00000 and 0.80995
Refinement method	Full-matrix least-squares on F^2
Data / restraints / parameters	5443 / 0 / 345
Goodness-of-fit on F^2	1.029
Final R indices [I>2sigma (I)]	R1 = 0.0275, wR2 = 0.0694
R indices (all data)	R1 = 0.0293, wR2 = 0.0702
Absolute structure parameter	-0.18 (5)
Extinction coefficient	0.00123 (19)
Largest diff. peak and hole	0.174 and -0.131 e.Å^{-3}

Table S17. Atomic coordinates (x 10^4) and equivalent isotropic displacement parameters (Å^{2}x 10^3)
for t. U (eq) is defined as one third of the trace of the orthogonalized U^{ij} tensor.

	x	y	z	U (eq)
C (16)	1457 (1)	3809 (1)	2322 (1)	22 (1)
C (27)	1716 (1)	3391 (1)	1686 (1)	22 (1)
O (1)	-2350 (1)	4541 (1)	2618 (1)	32 (1)
N (1)	4545 (1)	4241 (1)	4713 (1)	25 (1)
C (1)	5509 (1)	3520 (1)	4566 (1)	24 (1)
C (8)	4773 (2)	5324 (1)	4949 (1)	29 (1)
C (7)	-1217 (2)	4336 (1)	2574 (1)	26 (1)
C (6)	4920 (2)	2523 (1)	4289 (1)	25 (1)
C (32)	1355 (2)	673 (2)	882 (1)	33 (1)
C (24)	-588 (2)	3484 (1)	2958 (1)	24 (1)
C (22)	-746 (2)	2115 (2)	3794 (1)	30 (1)
C (4)	3513 (1)	2830 (1)	3497 (1)	23 (1)
O (4)	6635 (1)	3706 (1)	4645 (1)	31 (1)
C (20)	1242 (1)	2372 (1)	3203 (1)	22 (1)
C (29)	-194 (2)	2286 (1)	1374 (1)	26 (1)
C (23)	-1293 (2)	2939 (1)	3438 (1)	28 (1)
C (19)	691 (1)	3206 (1)	2831 (1)	21 (1)
C (18)	2734 (2)	5372 (2)	2065 (1)	30 (1)
C (17)	1963 (2)	4806 (1)	2501 (1)	26 (1)

C (5)	4939 (2)	2501 (2)	3512 (1)	30 (1)
C (13)	4670 (2)	6726 (2)	3262 (1)	42 (1)
C (26)	2544 (2)	3954 (1)	1264 (1)	28 (1)
C (3)	3472 (1)	2747 (1)	4280 (1)	23 (1)
O (3)	2364 (1)	4298 (1)	4751 (1)	34 (1)
C (14)	4801 (2)	5931 (2)	3743 (1)	33 (1)
C (10)	4209 (2)	7191 (2)	4586 (1)	36 (1)
C (33)	1888 (2)	1572 (1)	1174 (1)	29 (1)
C (15)	2644 (2)	2068 (1)	3117 (1)	24 (1)
C (25)	3046 (2)	4938 (2)	1451 (1)	31 (1)
C (28)	1124 (2)	2391 (1)	1428 (1)	23 (1)
C (30)	-729 (2)	1393 (1)	1071 (1)	30 (1)
C (9)	4574 (2)	6160 (2)	4411 (1)	28 (1)
C (12)	4298 (2)	7749 (2)	3437 (1)	47 (1)
C (31)	46 (2)	584 (2)	826 (1)	33 (1)
C (11)	4065 (2)	7979 (2)	4104 (1)	48 (1)
C (21)	514 (2)	1834 (1)	3675 (1)	27 (1)
C (2)	3325 (1)	3822 (1)	4607 (1)	25 (1)
O (2)	2893 (1)	1018 (1)	3358 (1)	33 (1)

Table S18. Bond lengths [Å] and angles [°] for t.

C (16)-C (17)	1.401 (2)
C (16)-C (27)	1.404 (2)
C (16)-C (19)	1.502 (2)
C (27)-C (26)	1.403 (2)
C (27)-C (28)	1.488 (2)
O (1)-C (7)	1.221 (2)
N (1)-C (1)	1.387 (2)
N (1)-C (2)	1.400 (2)
N (1)-C (8)	1.454 (2)
C (1)-O (4)	1.2167 (19)
C (1)-C (6)	1.499 (2)
C (8)-C (9)	1.516 (2)
C (7)-C (24)	1.471 (2)
C (6)-C (3)	1.548 (2)
C (6)-C (5)	1.558 (2)

C (32)-C (31)	1.385 (3)
C (32)-C (33)	1.386 (3)
C (24)-C (23)	1.392 (2)
C (24)-C (19)	1.411 (2)
C (22)-C (23)	1.377 (2)
C (22)-C (21)	1.392 (2)
C (4)-C (15)	1.524 (2)
C (4)-C (5)	1.555 (2)
C (4)-C (3)	1.574 (2)
C (20)-C (21)	1.389 (2)
C (20)-C (19)	1.407 (2)
C (20)-C (15)	1.532 (2)
C (29)-C (30)	1.388 (2)
C (29)-C (28)	1.396 (2)
C (18)-C (25)	1.385 (3)
C (18)-C (17)	1.386 (2)
C (13)-C (12)	1.381 (3)
C (13)-C (14)	1.391 (3)
C (26)-C (25)	1.389 (2)
C (3)-C (2)	1.502 (2)
O (3)-C (2)	1.2074 (19)
C (14)-C (9)	1.390 (2)
C (10)-C (9)	1.389 (3)
C (10)-C (11)	1.389 (3)
C (33)-C (28)	1.397 (2)
C (15)-O (2)	1.422 (2)
C (30)-C (31)	1.388 (3)
C (12)-C (11)	1.388 (3)

C (17)-C (16)-C (27)	119.26 (14)
C (17)-C (16)-C (19)	118.44 (14)
C (27)-C (16)-C (19)	122.27 (14)
C (26)-C (27)-C (16)	118.75 (15)
C (26)-C (27)-C (28)	118.08 (15)
C (16)-C (27)-C (28)	123.14 (14)
C (1)-N (1)-C (2)	113.23 (14)
C (1)-N (1)-C (8)	123.52 (14)
C (2)-N (1)-C (8)	123.24 (14)

O (4)-C (1)-N (1)	124.06 (17)
O (4)-C (1)-C (6)	127.47 (15)
N (1)-C (1)-C (6)	108.45 (13)
N (1)-C (8)-C (9)	112.77 (14)
O (1)-C (7)-C (24)	123.56 (16)
C (1)-C (6)-C (3)	105.03 (13)
C (1)-C (6)-C (5)	112.39 (15)
C (3)-C (6)-C (5)	90.28 (12)
C (31)-C (32)-C (33)	120.09 (17)
C (23)-C (24)-C (19)	120.79 (15)
C (23)-C (24)-C (7)	118.44 (14)
C (19)-C (24)-C (7)	120.75 (14)
C (23)-C (22)-C (21)	119.86 (15)
C (15)-C (4)-C (5)	114.95 (14)
C (15)-C (4)-C (3)	116.21 (13)
C (5)-C (4)-C (3)	89.46 (11)
C (21)-C (20)-C (19)	119.51 (14)
C (21)-C (20)-C (15)	119.14 (14)
C (19)-C (20)-C (15)	121.32 (14)
C (30)-C (29)-C (28)	120.81 (16)
C (22)-C (23)-C (24)	120.05 (15)
C (20)-C (19)-C (24)	118.57 (14)
C (20)-C (19)-C (16)	120.69 (14)
C (24)-C (19)-C (16)	120.72 (14)
C (25)-C (18)-C (17)	119.91 (17)
C (18)-C (17)-C (16)	121.02 (16)
C (4)-C (5)-C (6)	90.10 (12)
C (12)-C (13)-C (14)	120.80 (19)
C (25)-C (26)-C (27)	121.16 (16)
C (2)-C (3)-C (6)	104.95 (13)
C (2)-C (3)-C (4)	112.27 (14)
C (6)-C (3)-C (4)	89.75 (11)
C (9)-C (14)-C (13)	120.21 (19)
C (9)-C (10)-C (11)	120.74 (19)
C (32)-C (33)-C (28)	121.02 (15)
O (2)-C (15)-C (4)	107.19 (12)
O (2)-C (15)-C (20)	111.54 (13)
C (4)-C (15)-C (20)	111.40 (13)

C (18)-C (25)-C (26)	119.73 (16)
C (29)-C (28)-C (33)	118.21 (15)
C (29)-C (28)-C (27)	121.42 (15)
C (33)-C (28)-C (27)	120.08 (14)
C (29)-C (30)-C (31)	120.17 (16)
C (10)-C (9)-C (14)	118.86 (17)
C (10)-C (9)-C (8)	119.78 (15)
C (14)-C (9)-C (8)	121.30 (17)
C (13)-C (12)-C (11)	119.15 (19)
C (32)-C (31)-C (30)	119.68 (17)
C (12)-C (11)-C (10)	120.2 (2)
C (20)-C (21)-C (22)	121.21 (16)
O (3)-C (2)-N (1)	123.06 (16)
O (3)-C (2)-C (3)	129.10 (16)
N (1)-C (2)-C (3)	107.82 (13)

Symmetry transformations used to generate equivalent atoms:

Table S19. Anisotropic displacement parameters ($Å^2$x 10^3) for t. The anisotropic displacement factor exponent takes the form: $-2\pi^2[h^2 a^{*2}U^{11} + ... + 2 h k a^* b^* U^{12}]$

	U^{11}	U^{22}	U^{33}	U^{23}	U^{13}	U^{12}
C (16)	17 (1)	26 (1)	22 (1)	4 (1)	-2 (1)	0 (1)
C (27)	18 (1)	27 (1)	22 (1)	1 (1)	-2 (1)	-1 (1)
O (1)	23 (1)	39 (1)	35 (1)	3 (1)	-2 (1)	5 (1)
N (1)	24 (1)	33 (1)	19 (1)	-1 (1)	0 (1)	1 (1)
C (1)	22 (1)	37 (1)	14 (1)	4 (1)	2 (1)	3 (1)
C (8)	32 (1)	34 (1)	21 (1)	-4 (1)	-2 (1)	1 (1)
C (7)	23 (1)	30 (1)	26 (1)	0 (1)	0 (1)	0 (1)
C (6)	22 (1)	33 (1)	19 (1)	-1 (1)	-1 (1)	6 (1)
C (32)	39 (1)	30 (1)	32 (1)	-2 (1)	-4 (1)	6 (1)
C (24)	21 (1)	28 (1)	22 (1)	-2 (1)	-2 (1)	-1 (1)
C (22)	26 (1)	42 (1)	22 (1)	6 (1)	0 (1)	-8 (1)
C (4)	20 (1)	32 (1)	17 (1)	-1 (1)	1 (1)	2 (1)
O (4)	21 (1)	48 (1)	25 (1)	2 (1)	-2 (1)	-1 (1)
C (20)	22 (1)	27 (1)	17 (1)	-2 (1)	-2 (1)	-1 (1)
C (29)	23 (1)	31 (1)	24 (1)	1 (1)	0 (1)	-1 (1)

C (23)	20 (1)	40 (1)	24 (1)	0 (1)	-1 (1)	-2 (1)
C (19)	21 (1)	25 (1)	18 (1)	-2 (1)	-2 (1)	-3 (1)
C (18)	27 (1)	30 (1)	32 (1)	2 (1)	-4 (1)	-8 (1)
C (17)	26 (1)	28 (1)	25 (1)	-1 (1)	-2 (1)	-1 (1)
C (5)	21 (1)	47 (1)	21 (1)	-5 (1)	0 (1)	5 (1)
C (13)	38 (1)	64 (1)	24 (1)	8 (1)	-7 (1)	-19 (1)
C (26)	24 (1)	35 (1)	23 (1)	2 (1)	1 (1)	-2 (1)
C (3)	20 (1)	31 (1)	19 (1)	0 (1)	0 (1)	1 (1)
O (3)	24 (1)	50 (1)	29 (1)	-9 (1)	0 (1)	10 (1)
C (14)	29 (1)	47 (1)	24 (1)	-1 (1)	-2 (1)	-9 (1)
C (10)	42 (1)	38 (1)	29 (1)	1 (1)	-1 (1)	-6 (1)
C (33)	26 (1)	35 (1)	27 (1)	0 (1)	-3 (1)	3 (1)
C (15)	24 (1)	30 (1)	19 (1)	0 (1)	-2 (1)	4 (1)
C (25)	26 (1)	38 (1)	30 (1)	7 (1)	1 (1)	-9 (1)
C (28)	23 (1)	28 (1)	18 (1)	2 (1)	-2 (1)	-1 (1)
C (30)	27 (1)	37 (1)	27 (1)	1 (1)	-2 (1)	-8 (1)
C (9)	24 (1)	37 (1)	23 (1)	1 (1)	-3 (1)	-7 (1)
C (12)	50 (1)	52 (1)	39 (1)	19 (1)	-14 (1)	-20 (1)
C (31)	40 (1)	30 (1)	30 (1)	0 (1)	-6 (1)	-7 (1)
C (11)	56 (1)	39 (1)	48 (1)	7 (1)	-7 (1)	-7 (1)
C (21)	28 (1)	31 (1)	22 (1)	4 (1)	-6 (1)	-3 (1)
C (2)	22 (1)	38 (1)	16 (1)	1 (1)	0 (1)	4 (1)
O (2)	38 (1)	28 (1)	31 (1)	-3 (1)	-9 (1)	8 (1)

Table S20. Hydrogen coordinates (x 10^4) and isotropic displacement parameters (Å^{2}x 10 ^{3}) for t.

	x	y	z	U (eq)
H (8A)	5657	5376	5117	35
H (8B)	4193	5477	5326	35
H (7)	-715	4749	2276	32
H (6)	5181	1841	4512	30
H (32)	1888	117	719	40
H (22)	-1229	1740	4119	36
H (4)	3403	3586	3343	28

H (29)	-731	2831	1546	31
H (23)	-2151	3135	3521	33
H (18)	3048	6058	2187	36
H (17)	1775	5098	2927	31
H (5A)	5514	3040	3311	36
H (5B)	5103	1782	3322	36
H (13)	4837	6564	2807	50
H (26)	2766	3657	844	33
H (3)	2923	2164	4464	28
H (14)	5047	5229	3614	40
H (10)	4055	7358	5042	44
H (33)	2788	1632	1203	35
H (15)	2864	2099	2632	29
H (25)	3601	5311	1158	37
H (30)	-1627	1337	1032	36
H (12)	4204	8287	3106	56
H (31)	-321	-28	620	40
H (11)	3807	8679	4230	57
H (21)	884	1263	3921	32
H (2)	2769	573	3051	49

Table S21. Torsion angles [°] for t.

C (17)-C (16)-C (27)-C (26)	4.2 (2)
C (19)-C (16)-C (27)-C (26)	-173.66 (14)
C (17)-C (16)-C (27)-C (28)	-174.03 (14)
C (19)-C (16)-C (27)-C (28)	8.1 (2)
C (2)-N (1)-C (1)-O (4)	-177.28 (15)
C (8)-N (1)-C (1)-O (4)	3.0 (2)
C (2)-N (1)-C (1)-C (6)	4.08 (18)
C (8)-N (1)-C (1)-C (6)	-175.67 (14)
C (1)-N (1)-C (8)-C (9)	103.85 (17)
C (2)-N (1)-C (8)-C (9)	-75.9 (2)
O (4)-C (1)-C (6)-C (3)	-177.97 (16)
N (1)-C (1)-C (6)-C (3)	0.61 (17)
O (4)-C (1)-C (6)-C (5)	-81.3 (2)
N (1)-C (1)-C (6)-C (5)	97.28 (16)

O (1)-C (7)-C (24)-C (23)	-5.6 (3)
O (1)-C (7)-C (24)-C (19)	172.78 (16)
C (21)-C (22)-C (23)-C (24)	0.4 (3)
C (19)-C (24)-C (23)-C (22)	-0.3 (3)
C (7)-C (24)-C (23)-C (22)	178.06 (15)
C (21)-C (20)-C (19)-C (24)	1.1 (2)
C (15)-C (20)-C (19)-C (24)	-176.78 (14)
C (21)-C (20)-C (19)-C (16)	179.46 (15)
C (15)-C (20)-C (19)-C (16)	1.5 (2)
C (23)-C (24)-C (19)-C (20)	-0.5 (2)
C (7)-C (24)-C (19)-C (20)	-178.80 (14)
C (23)-C (24)-C (19)-C (16)	-178.82 (15)
C (7)-C (24)-C (19)-C (16)	2.9 (2)
C (17)-C (16)-C (19)-C (20)	-99.45 (18)
C (27)-C (16)-C (19)-C (20)	78.41 (19)
C (17)-C (16)-C (19)-C (24)	78.84 (19)
C (27)-C (16)-C (19)-C (24)	-103.30 (18)
C (25)-C (18)-C (17)-C (16)	-2.0 (3)
C (27)-C (16)-C (17)-C (18)	-1.4 (2)
C (19)-C (16)-C (17)-C (18)	176.55 (15)
C (15)-C (4)-C (5)-C (6)	-123.70 (15)
C (3)-C (4)-C (5)-C (6)	-4.83 (13)
C (1)-C (6)-C (5)-C (4)	-101.50 (15)
C (3)-C (6)-C (5)-C (4)	4.91 (14)
C (16)-C (27)-C (26)-C (25)	-3.8 (2)
C (28)-C (27)-C (26)-C (25)	174.54 (15)
C (1)-C (6)-C (3)-C (2)	-4.57 (16)
C (5)-C (6)-C (3)-C (2)	-117.87 (14)
C (1)-C (6)-C (3)-C (4)	108.45 (13)
C (5)-C (6)-C (3)-C (4)	-4.85 (13)
C (15)-C (4)-C (3)-C (2)	-131.31 (14)
C (5)-C (4)-C (3)-C (2)	110.95 (14)
C (15)-C (4)-C (3)-C (6)	122.61 (15)
C (5)-C (4)-C (3)-C (6)	4.86 (13)
C (12)-C (13)-C (14)-C (9)	-0.8 (3)
C (31)-C (32)-C (33)-C (28)	1.2 (3)
C (5)-C (4)-C (15)-O (2)	50.38 (18)
C (3)-C (4)-C (15)-O (2)	-52.17 (17)

C (5)-C (4)-C (15)-C (20)	172.66 (13)
C (3)-C (4)-C (15)-C (20)	70.10 (17)
C (21)-C (20)-C (15)-O (2)	21.7 (2)
C (19)-C (20)-C (15)-O (2)	-160.42 (14)
C (21)-C (20)-C (15)-C (4)	-98.08 (18)
C (19)-C (20)-C (15)-C (4)	79.84 (18)
C (17)-C (18)-C (25)-C (26)	2.5 (3)
C (27)-C (26)-C (25)-C (18)	0.4 (3)
C (30)-C (29)-C (28)-C (33)	-0.4 (3)
C (30)-C (29)-C (28)-C (27)	173.52 (15)
C (32)-C (33)-C (28)-C (29)	-0.6 (3)
C (32)-C (33)-C (28)-C (27)	-174.65 (16)
C (26)-C (27)-C (28)-C (29)	-119.50 (18)
C (16)-C (27)-C (28)-C (29)	58.7 (2)
C (26)-C (27)-C (28)-C (33)	54.3 (2)
C (16)-C (27)-C (28)-C (33)	-127.43 (17)
C (28)-C (29)-C (30)-C (31)	0.9 (3)
C (11)-C (10)-C (9)-C (14)	0.3 (3)
C (11)-C (10)-C (9)-C (8)	177.70 (17)
C (13)-C (14)-C (9)-C (10)	0.4 (2)
C (13)-C (14)-C (9)-C (8)	-176.96 (16)
N (1)-C (8)-C (9)-C (10)	152.13 (15)
N (1)-C (8)-C (9)-C (14)	-30.6 (2)
C (14)-C (13)-C (12)-C (11)	0.5 (3)
C (33)-C (32)-C (31)-C (30)	-0.7 (3)
C (29)-C (30)-C (31)-C (32)	-0.3 (3)
C (13)-C (12)-C (11)-C (10)	0.2 (3)
C (9)-C (10)-C (11)-C (12)	-0.6 (3)
C (19)-C (20)-C (21)-C (22)	-1.0 (2)
C (15)-C (20)-C (21)-C (22)	176.95 (15)
C (23)-C (22)-C (21)-C (20)	0.2 (3)
C (1)-N (1)-C (2)-O (3)	174.54 (16)
C (8)-N (1)-C (2)-O (3)	-5.7 (2)
C (1)-N (1)-C (2)-C (3)	-7.14 (18)
C (8)-N (1)-C (2)-C (3)	172.61 (14)
C (6)-C (3)-C (2)-O (3)	-174.83 (17)
C (4)-C (3)-C (2)-O (3)	89.2 (2)
C (6)-C (3)-C (2)-N (1)	6.98 (17)

C (4)-C (3)-C (2)-N (1)	-89.02 (15)

Symmetry transformations used to generate equivalent atoms:

Table S22. Hydrogen bonds for t [Å and °].

D-H...A	d (D-H)	d (H...A)	d (D...A)	< (DHA)

■ Supplementary Reference:

【1】 Deng, Z. ; Dickschat, Jeroen S.; Dong, Y.; Lu, J.; Luo, M.; Qi, M.; Shen, K.; Xu, H.; Yin, Z. The Mechanism of Dehydrating Bimodules in *trans*-Acyltransferase Polyketide Biosynthesis: A Showcase Study on Hepatoprotective Hangtaimycin. *Angew. Chem. Int. Ed.* **60***,* e202106250 (2021).

【2】 Jiang, H.; He, X.K.; Jiang, X.; Zhao, W.; Lu, L.Q.; Cheng, Y. and Xiao W.J. Photoinduced Cobalt-Catalyzed Desymmetrization of Dialdehydes to Access Axial Chirality. *J. Am. Chem. Soc.* **145**, 6944−6952 (2023).

【3】 Goetzke F W.; Hell A M L.; van Dijk L.; Fletcher S P. A Catalytic Asymmetric cross-coupling approach to the synthesis of cyclobutanes. *Nat. Chem.* **13**, 880 (2023).

【4】 Smith, III, A. B.; Safonov, I. G.; Corbett, R. M. Total Synthesis of (+)-Zampanolide. *J. Am. Chem. Soc*. **123**, 12426–12427 (2001).

【5】 Fischer, D.; Nguyen, T. X.; Trzoss, L.; Dakanali, M.; Theodorakis, E. A. Intramolecular Cyclization Strategies Towards the Synthesis of Zoanthamine Alkaloids. *Tetrahedron Lett*. **52**, 4920–4923 (2011).

【6】 Liang, Z.; Wang, L.; Wang, Y.; Wang, L.; Chong, Q.; Meng, F. Cobalt-Catalyzed Diastereo- and Enantioselective Carbon–Carbon Bond Forming Reactions of Cyclobutenes. *J. Am. Chem. Soc*. **145**, 3588–3598 (2023).

【7】 Gu, P.; Ding, L.; Fang, X.; Zhu, J.; Kang, S.; Wu, B.; Zhang, J.; Zhao, Y.; Shi, Z. Chromium and Metal-Reductant-Free Asymmetric Nozaki-Hiyama-Kishi (NHK) Reaction Enabled by Metallaphotoredox Catalysis. *Angew. Chem. Int. Ed*. **63**, e202408195 (2024).

【8】 Frisch, M. J.; Trucks, G. W.; Schlegel, H. B.; Scuseria, G. E.; Robb, M. A.; Cheeseman, J. R.; Scalmani, G.; Barone, V.; Petersson, G. A.; Nakatsuji, H.; Li, X.; Caricato, M.; Marenich, A. V.; Bloino, J.; Janesko, B. G.; Gomperts, R.; Mennucci, B.; Hratchian, H. P.; Ortiz, J. V.; Izmaylov, A. F.; Sonnenberg, J. L.; Williams; Ding, F.; Lipparini, F.; Egidi, F.; Goings, J.; Peng, B.; Petrone, A.; Henderson, T.; Ranasinghe, D.; Zakrzewski, V. G.; Gao, J.; Rega, N.; Zheng, G.; Liang, W.; Hada, M.; Ehara, M.; Toyota, K.; Fukuda, R.; Hasegawa, J.; Ishida, M.; Nakajima, T.; Honda, Y.; Kitao, O.; Nakai, H.; Vreven, T.; Throssell, K.; Montgomery Jr., J. A.; Peralta, J. E.; Ogliaro, F.; Bearpark, M. J.; Heyd, J. J.; Brothers, E. N.; Kudin, K. N.; Staroverov, V. N.; Keith, T. A.; Kobayashi, R.; Normand, J.; Raghavachari, K.; Rendell, A. P.; Burant, J. C.; Iyengar, S. S.; Tomasi, J.; Cossi, M.; Millam, J. M.; Klene, M.; Adamo, C.; Cammi, R.; Ochterski, J. W.; Martin, R. L.; Morokuma, K.; Farkas, O.; Foresman, J. B.; Fox, D. J. *Gaussian 16 Rev. A.03*, Wallingford, CT, 2016.

【9】 Becke, A. D., Density-functional thermochemistry. III. The role of exact exchange. *J. Chem. Phys.* **98**, 5648-5652 (1993).

【10】 Grimme, S.; Antony, J.; Ehrlich, S.; Krieg, H., A consistent and accurate ab initio parametrization of density functional dispersion correction (DFT-D) for the 94 elements H-Pu. *J. Chem. Phys.* **132**, 154104 (2010).

【11】 Weigend, F.; Ahlrichs, R., Balanced basis sets of split valence, triple zeta valence and quadruple zeta valence quality for H to Rn: Design and assessment of accuracy. *Phys. Chem. Chem. Phys.* **7**, 3297-3305 (2005).

【12】 Zhao, Y.; Truhlar, D. G., The M06 suite of density functionals for main group thermochemistry, thermochemical kinetics, noncovalent interactions, excited states, and transition elements: two new functionals and systematic testing of four M06-class functionals and 12 other functionals. *Theor. Chem. Acc.* **120**, 215-241 (2008).

【13】 Fukui, K., The path of chemical reactions-the IRC approach. *Acc. Chem. Res.* **14**, 363-368 (1981).

【14】 Staroverov, V. N.; Scuseria, G. E.; Tao, J.; Perdew, J. P., Comparative assessment of a new nonempirical density functional: Molecules and hydrogen-bonded complexes. *J. Chem. Phys.* **119**, 12129-12137 (2003).

【15】 Tao, J.; Perdew, J. P.; Staroverov, V. N.; Scuseria, G. E., Climbing the Density Functional Ladder: Nonempirical Meta--Generalized Gradient Approximation Designed for Molecules and Solids. *Phys. Rev. Lett.* **91**, 146401 (2003).

【16】 Amabilino, S.; Deeth, R. J., DFT Analysis of Spin Crossover in Mn(III) Complexes: Is a Two-Electron S = 2 to S = 0 Spin Transition Feasible? *Inorg. Chem.* **56**, 2602-2613 (2017).

【17】 Kepp, K. P., Theoretical Study of Spin Crossover in 30 Iron Complexes. *Inorg. Chem.* **55**, 2717-2727 (2016).

【18】 Jensen, K. P., Bioinorganic Chemistry Modeled with the TPSSh Density Functional. *Inorg. Chem.* **47**, 10357-10365 (2008).

【19】 Marenich, A. V.; Cramer, C. J.; Truhlar, D. G., Universal solvation model based on solute electron density and on a continuum model of the solvent defined by the bulk dielectric constant and atomic surface tensions. *J. Phys. Chem. B* **113**, 6378-6396 (2009).

■ NMR Spectra

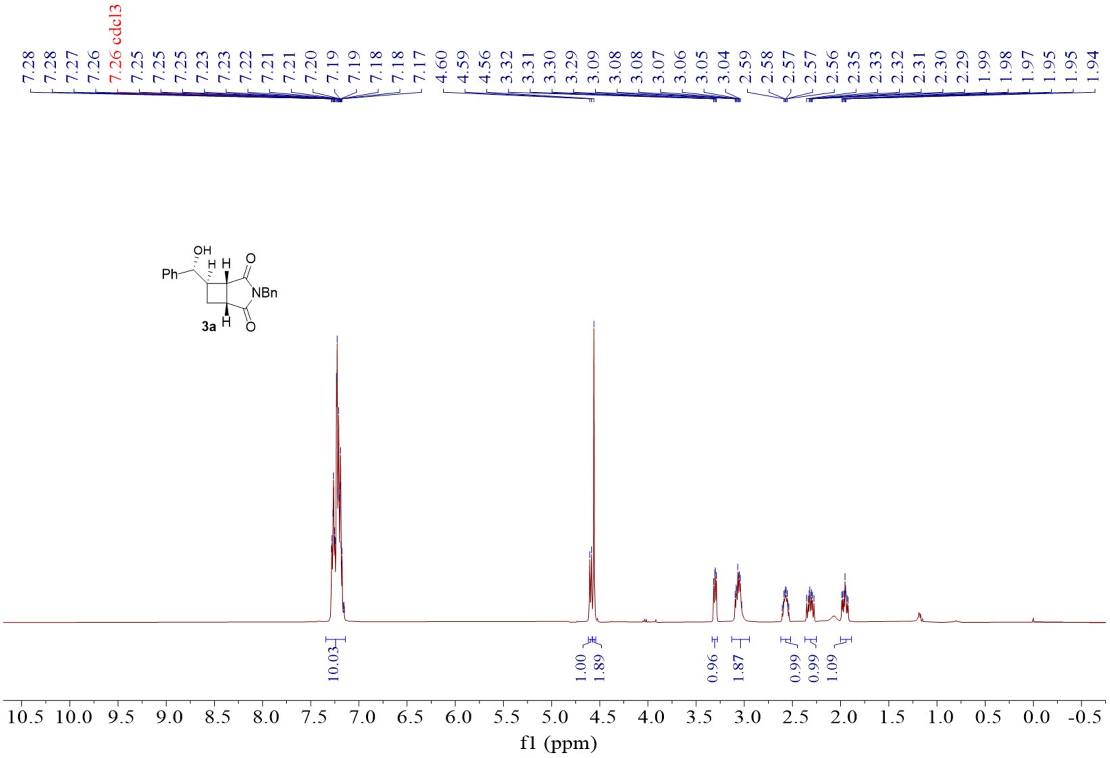

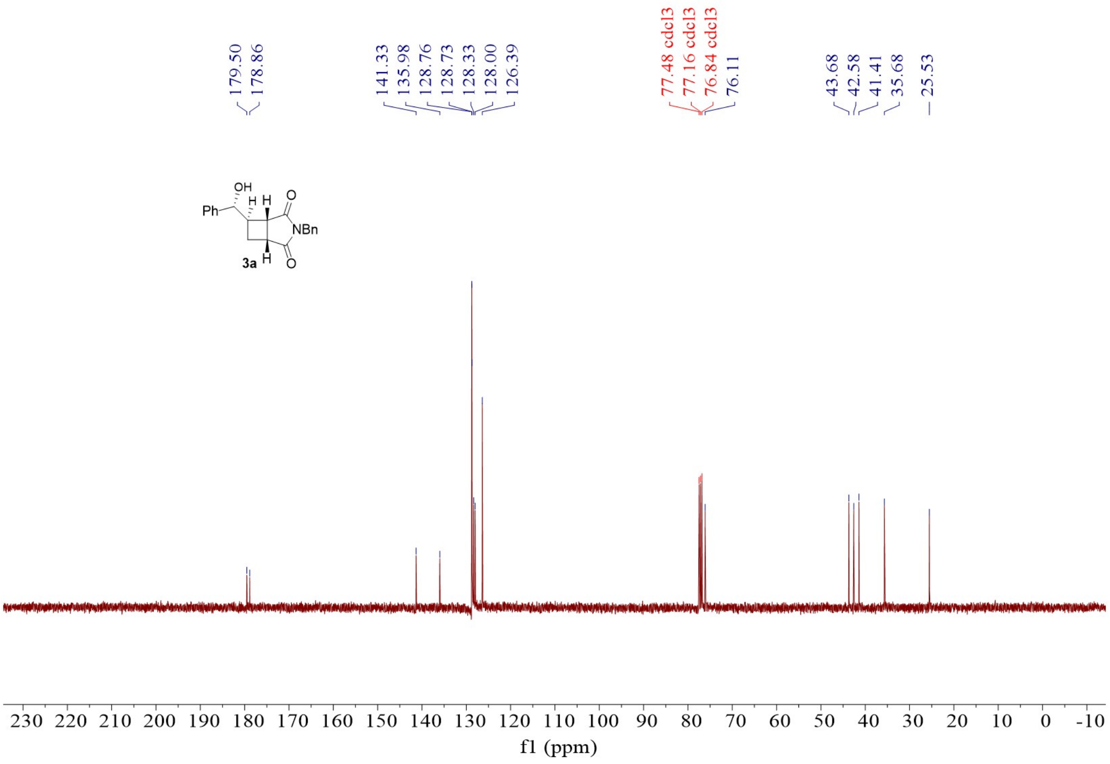

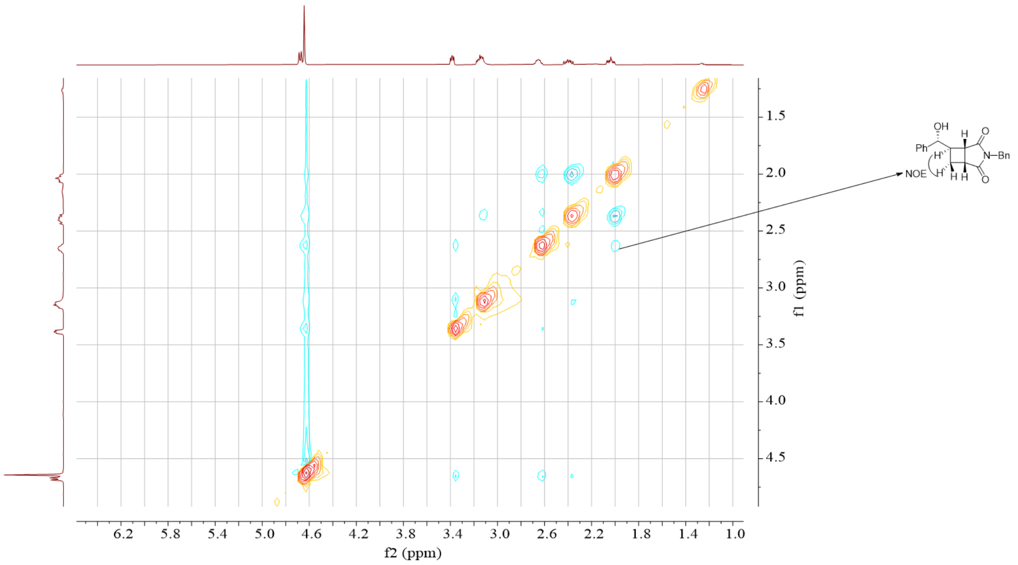
OH
H
O
Ph
H
N–Bn
NOE
H
H H
O
6.2
5.8
5.4
5.0
4.6
4.2
3.8
3.4
3.0
2.6
2.2
1.8
1.4
1.0
f2 (ppm)
1.5
2.0
2.5
3.0
3.5
4.0
4.5
f1 (ppm)

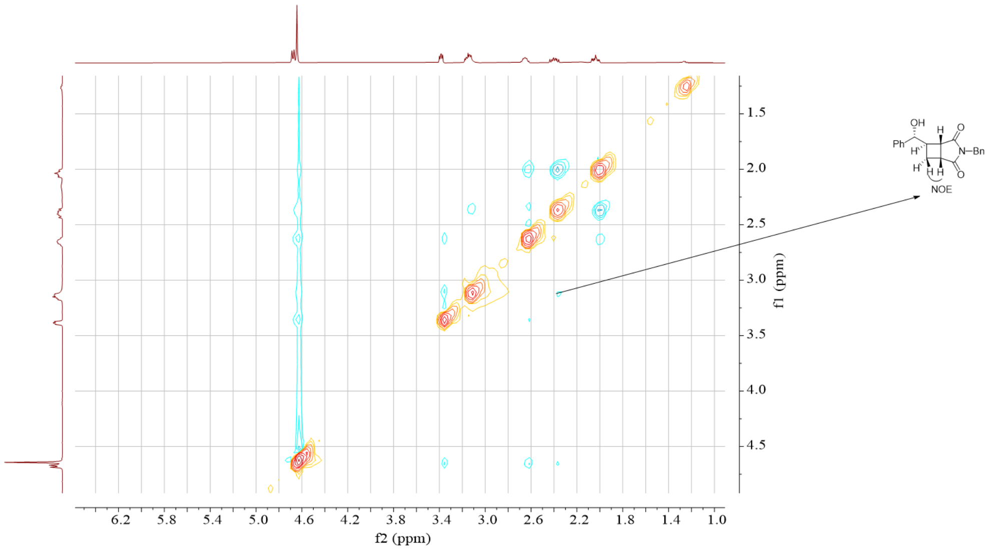
OH
H
O
Ph
H
N–Bn
H
H H
O
NOE
6.2
5.8
5.4
5.0
4.6
4.2
3.8
3.4
3.0
2.6
2.2
1.8
1.4
1.0
f2 (ppm)
1.5
2.0
2.5
3.0
3.5
4.0
4.5
f1 (ppm)

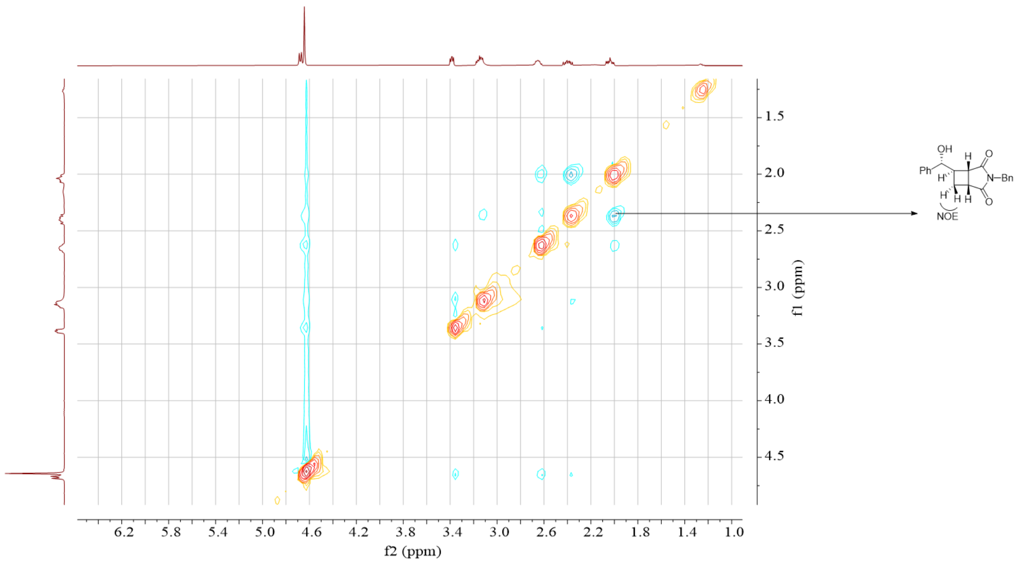
1.5
2.0
2.5
3.0
3.5
4.0
4.5
f1 (ppm)
6.2
5.8
5.4
5.0
4.6
4.2
3.8
3.4
3.0
2.6
2.2
1.8
1.4
1.0
f2 (ppm)
OH
Ph
H
O
N–Bn
NOE

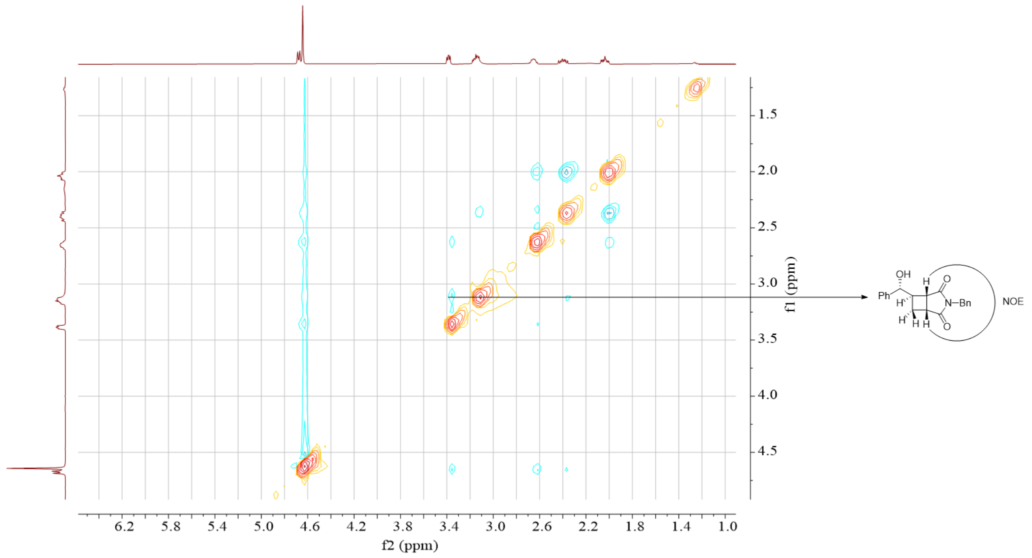
1.5
2.0
2.5
3.0
3.5
4.0
4.5
f1 (ppm)
6.2
5.8
5.4
5.0
4.6
4.2
3.8
3.4
3.0
2.6
2.2
1.8
1.4
1.0
f2 (ppm)
OH
Ph
H
O
N–Bn
NOE

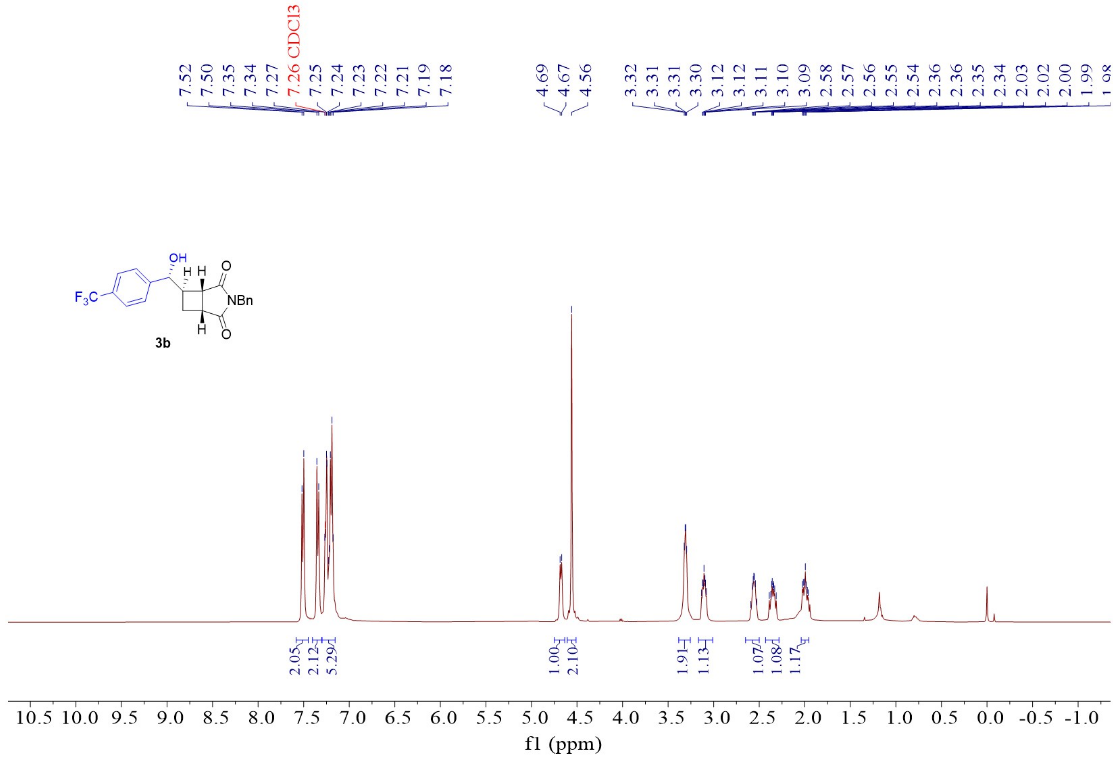
7.52
7.50
7.35
7.34
7.27
7.26 CDCl3
7.25
7.24
7.23
7.22
7.21
7.19
7.18
4.69
4.67
4.56
3.32
3.31
3.31
3.30
3.12
3.12
3.11
3.10
3.09
2.58
2.57
2.56
2.55
2.54
2.36
2.35
2.34
2.03
2.02
2.00
1.99
OH
H
H
O
F3C
NBn
H
O
3b
2.05
2.12
5.29
1.00
2.10
1.91
1.13
1.07
1.08
1.17
10.5 10.0 9.5 9.0 8.5 8.0 7.5 7.0 6.5 6.0 5.5 5.0 4.5 4.0 3.5 3.0 2.5 2.0 1.5 1.0 0.5 0.0 -0.5 -1.0
f1 (ppm)

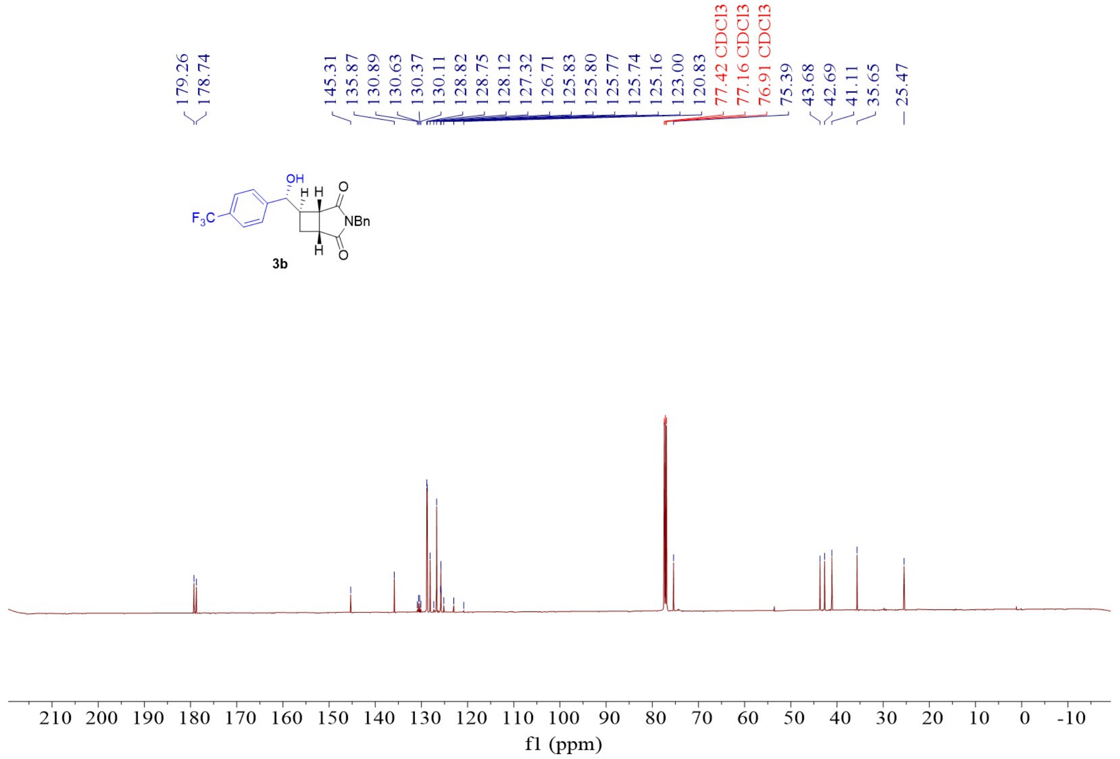
179.26
178.74
145.31
135.87
130.89
130.63
130.37
130.11
128.82
128.75
128.12
127.32
126.71
125.83
125.80
125.77
125.74
125.16
123.00
120.83
77.42 CDCl3
77.16 CDCl3
76.91 CDCl3
75.39
43.68
42.69
41.11
35.65
25.47
OH
H
H
O
F3C
NBn
H
O
3b
210 200 190 180 170 160 150 140 130 120 110 100 90 80 70 60 50 40 30 20 10 0 -10
f1 (ppm)

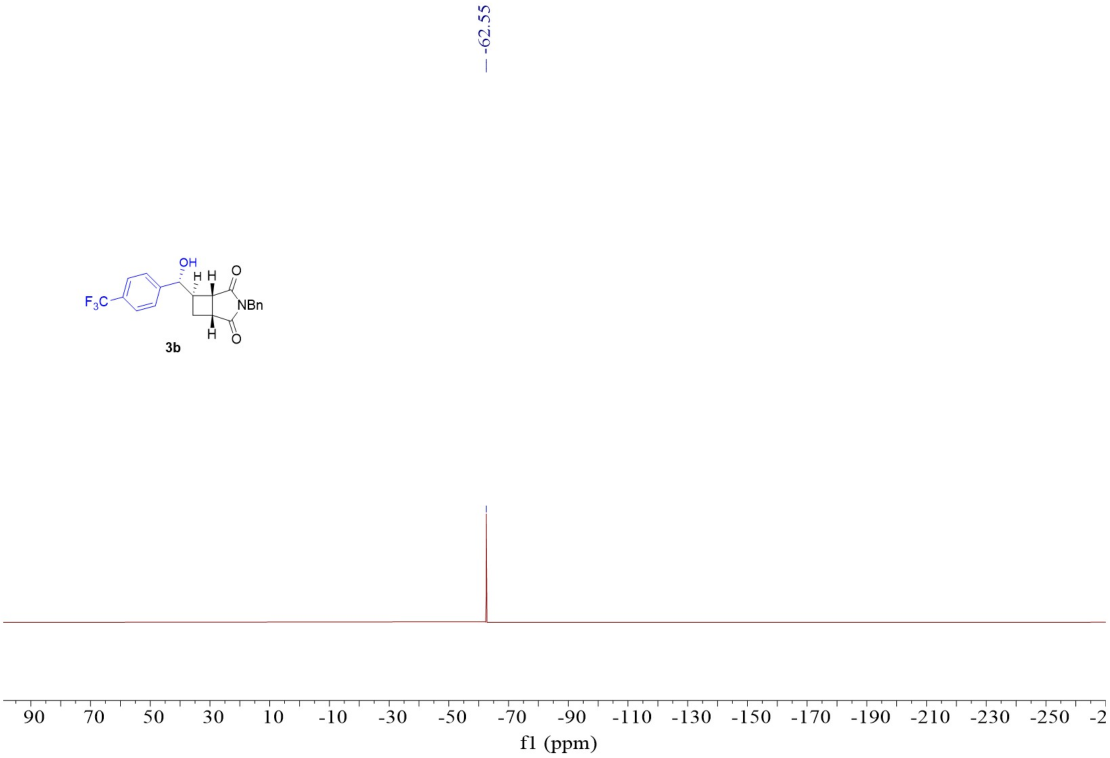
-62.55
3b
90 70 50 30 10 -10 -30 -50 -70 -90 -110 -130 -150 -170 -190 -210 -230 -250
f1 (ppm)

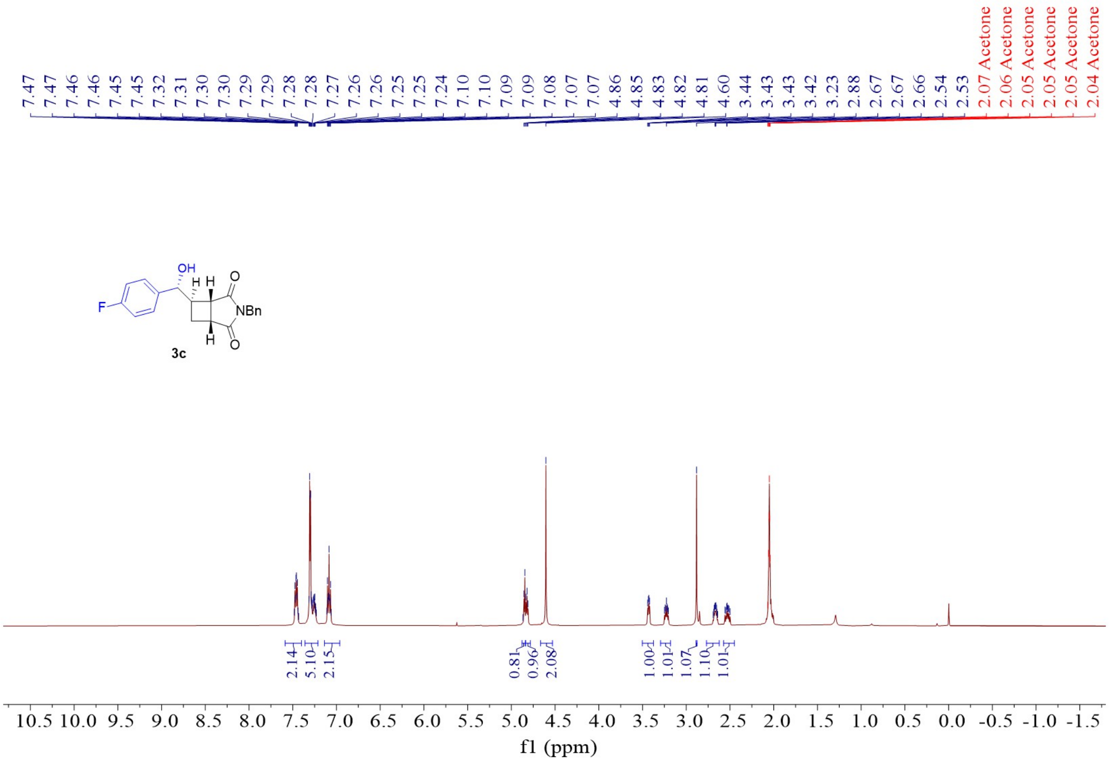
7.47 7.47 7.46 7.46 7.45 7.45 7.32 7.31 7.30 7.30 7.29 7.29 7.28 7.28 7.27 7.26 7.26 7.25 7.25 7.24 7.10 7.10 7.09 7.09 7.08 7.07 7.07 4.86 4.85 4.83 4.82 4.81 4.60 3.44 3.43 3.43 3.42 3.23 2.88 2.67 2.67 2.66 2.54 2.53
2.07 Acetone 2.06 Acetone 2.05 Acetone 2.05 Acetone 2.05 Acetone 2.04 Acetone
3c
2.14 5.10 2.15 0.81 0.96 2.08 1.00 1.01 1.07 1.10 1.01
10.5 10.0 9.5 9.0 8.5 8.0 7.5 7.0 6.5 6.0 5.5 5.0 4.5 4.0 3.5 3.0 2.5 2.0 1.5 1.0 0.5 0.0 -0.5 -1.0 -1.5
f1 (ppm)

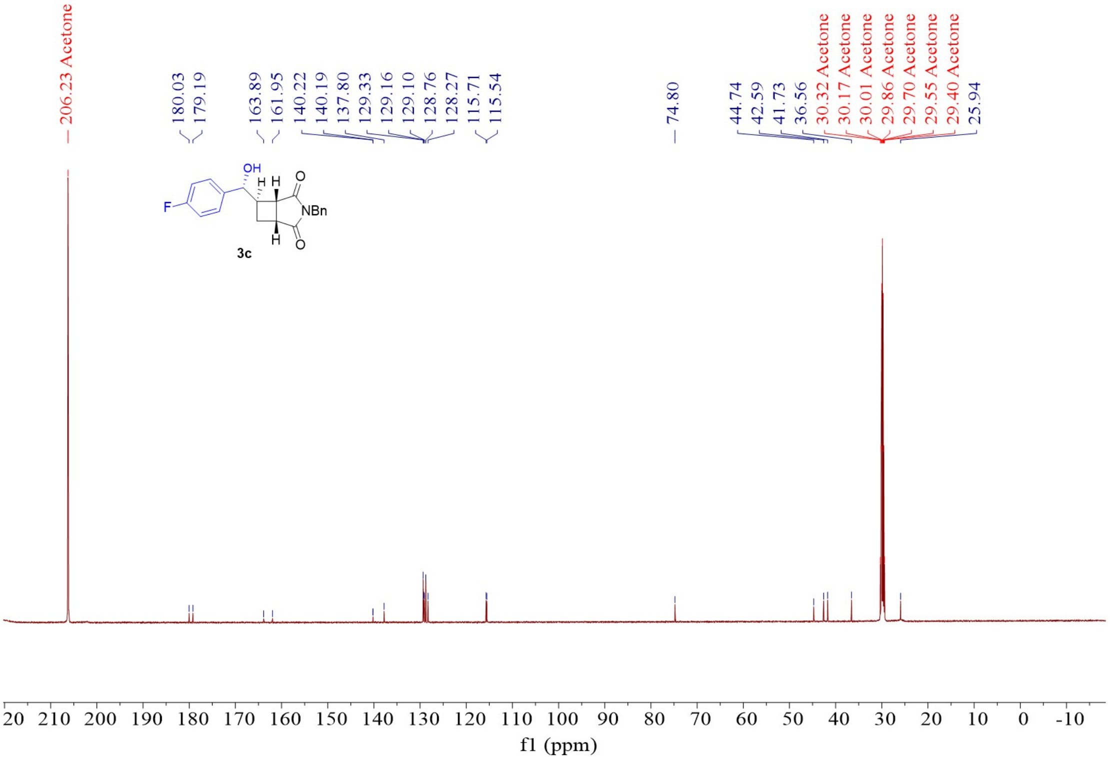
206.23 Acetone
180.03
179.19
163.89
161.95
140.22
140.19
137.80
129.33
129.16
129.10
128.76
128.27
115.71
115.54
74.80
44.74
42.59
41.73
36.56
30.32 Acetone
30.17 Acetone
30.01 Acetone
29.86 Acetone
29.70 Acetone
29.55 Acetone
29.40 Acetone
25.94
OH
H
H
O
F
NBn
H
O
3c
20 210 200 190 180 170 160 150 140 130 120 110 100 90 80 70 60 50 40 30 20 10 0 -10
f1 (ppm)

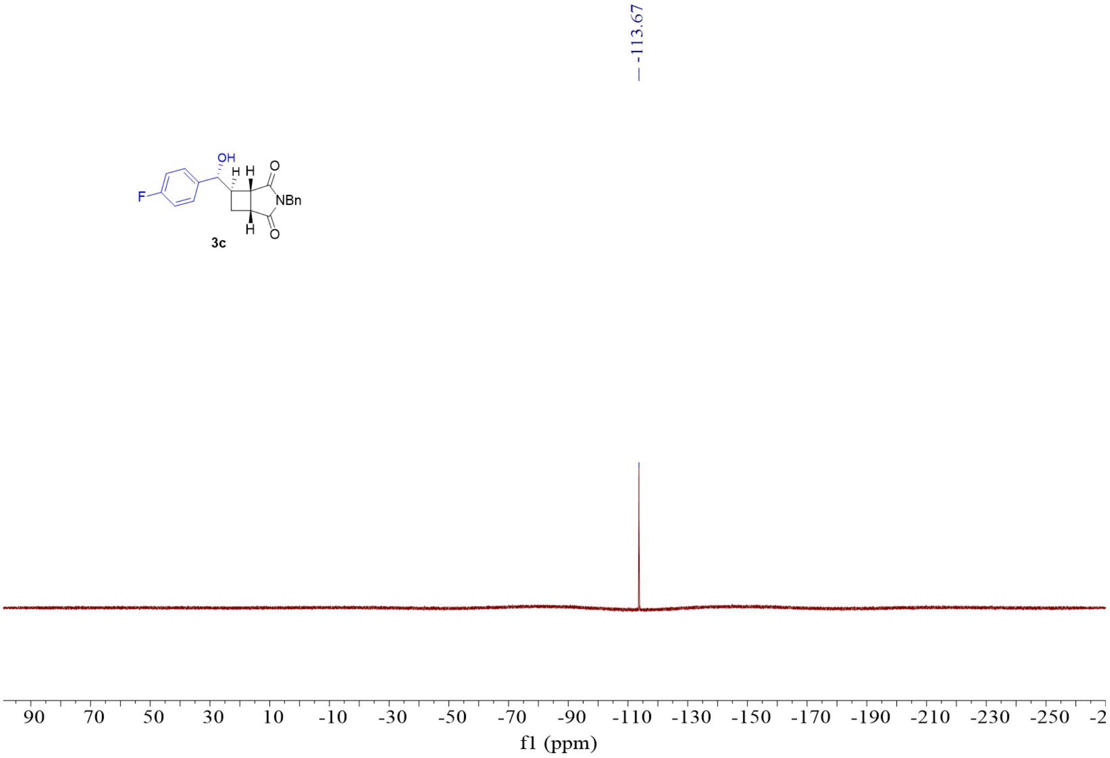
-113.67
OH
H
H
O
F
NBn
H
O
3c
90 70 50 30 10 -10 -30 -50 -70 -90 -110 -130 -150 -170 -190 -210 -230 -250 -2
f1 (ppm)

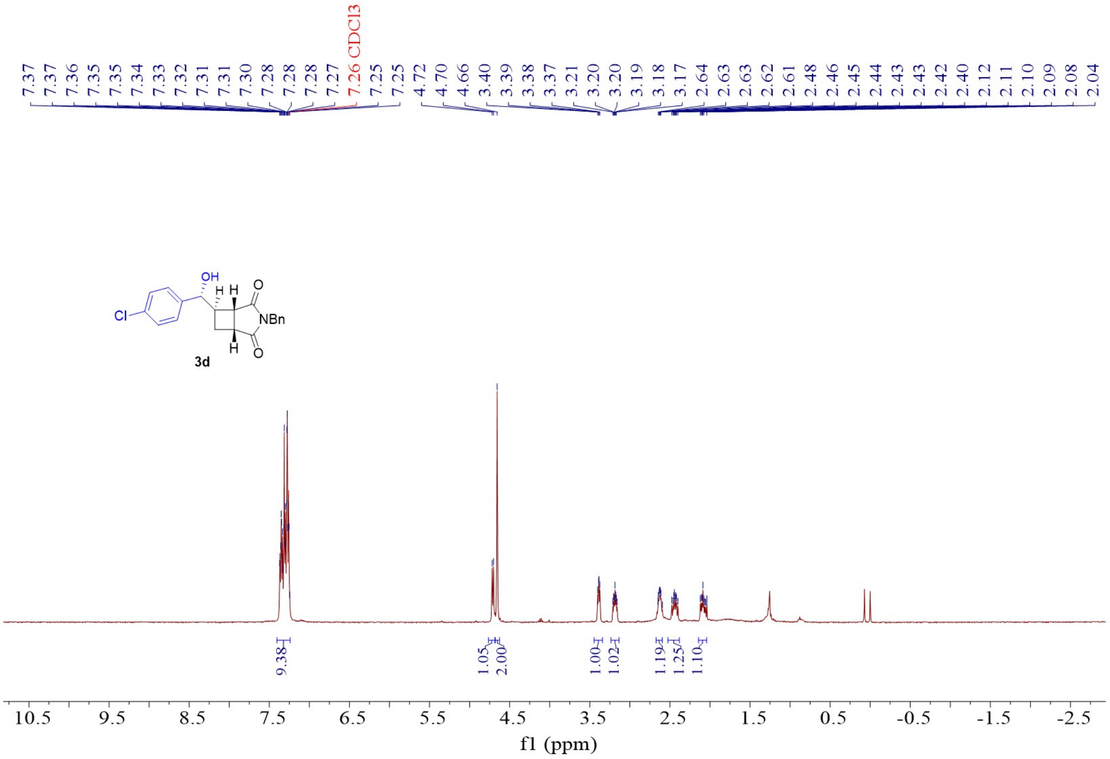
7.37 7.37 7.36 7.35 7.35 7.34 7.33 7.32 7.31 7.31 7.30 7.28 7.28 7.28 7.27 7.26 CDCl3 7.25 7.25 4.72 4.70 4.66 3.40 3.39 3.38 3.37 3.21 3.20 3.20 3.19 3.18 3.17 2.64 2.63 2.63 2.62 2.61 2.48 2.46 2.45 2.44 2.43 2.43 2.42 2.40 2.12 2.11 2.10 2.09 2.08 2.04
OH
H
Cl
NBn
H
3d
9.38
1.05
2.00
1.00
1.02
1.19
1.25
1.10
10.5 9.5 8.5 7.5 6.5 5.5 4.5 3.5 2.5 1.5 0.5 -0.5 -1.5 -2.5
f1 (ppm)

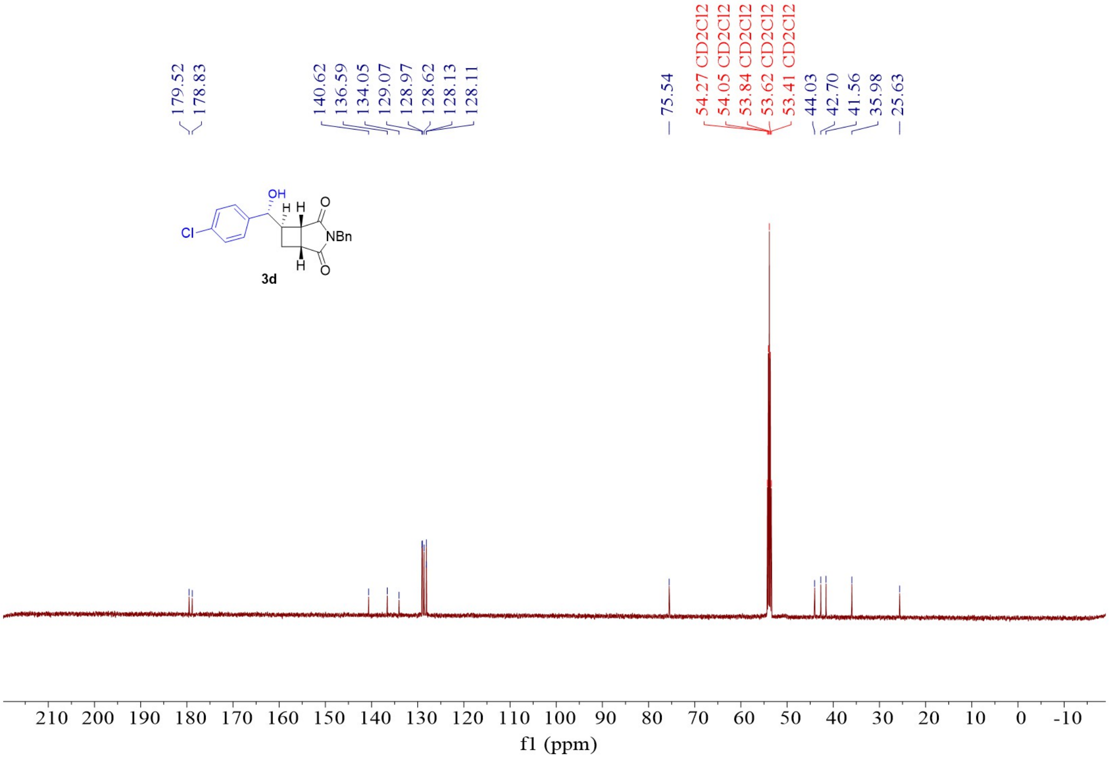
179.52 178.83
140.62 136.59 134.05 129.07 128.97 128.62 128.13 128.11
75.54
54.27 CD2Cl2 54.05 CD2Cl2 53.84 CD2Cl2 53.62 CD2Cl2 53.41 CD2Cl2
44.03 42.70 41.56 35.98 25.63
OH
H
Cl
NBn
H
3d
210 200 190 180 170 160 150 140 130 120 110 100 90 80 70 60 50 40 30 20 10 0 -10
f1 (ppm)

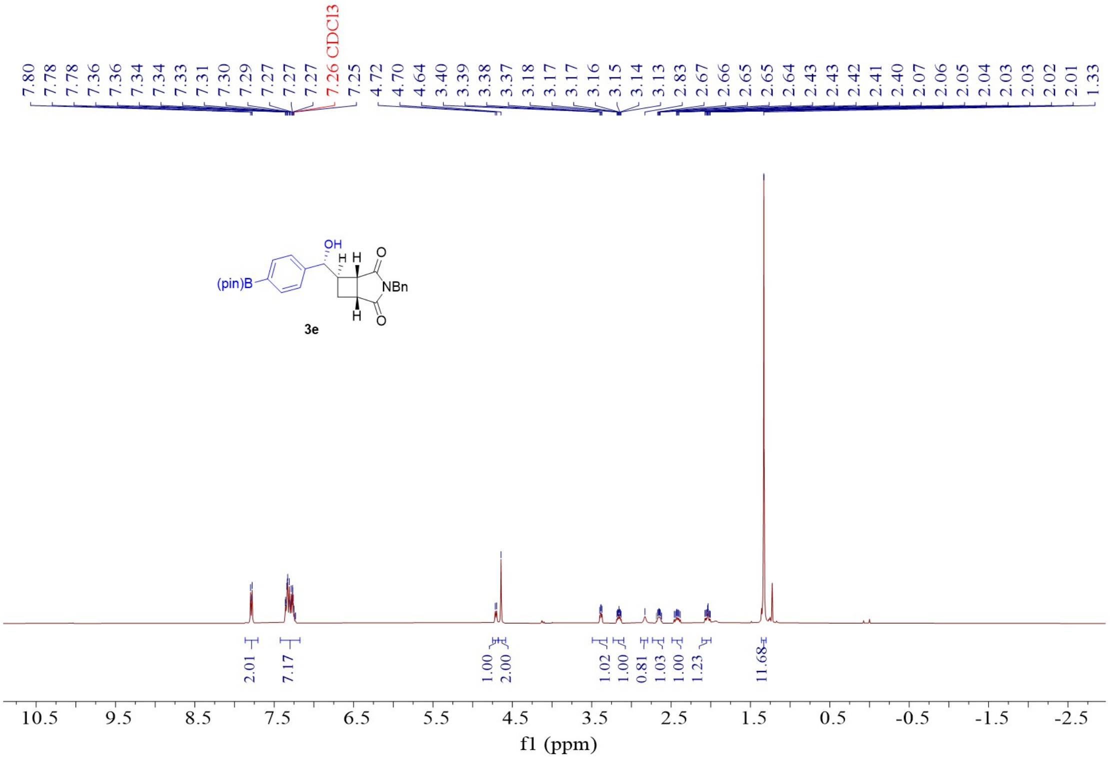

7.80
7.78
7.78
7.36
7.36
7.34
7.34
7.33
7.31
7.30
7.29
7.27
7.27
7.27
7.26 CDCl3
7.25
4.72
4.70
4.64
3.40
3.39
3.38
3.37
3.18
3.17
3.17
3.16
3.15
3.14
3.13
2.83
2.67
2.66
2.65
2.65
2.64
2.43
2.43
2.42
2.41
2.40
2.07
2.06
2.05
2.04
2.03
2.03
2.02
2.01
1.33
OH
H
H
O
(pin)B
NBn
H
O
3e
2.01
7.17
1.00
2.00
1.02
1.00
0.81
1.03
1.00
1.23
11.68
10.5 9.5 8.5 7.5 6.5 5.5 4.5 3.5 2.5 1.5 0.5 -0.5 -1.5 -2.5
f1 (ppm)

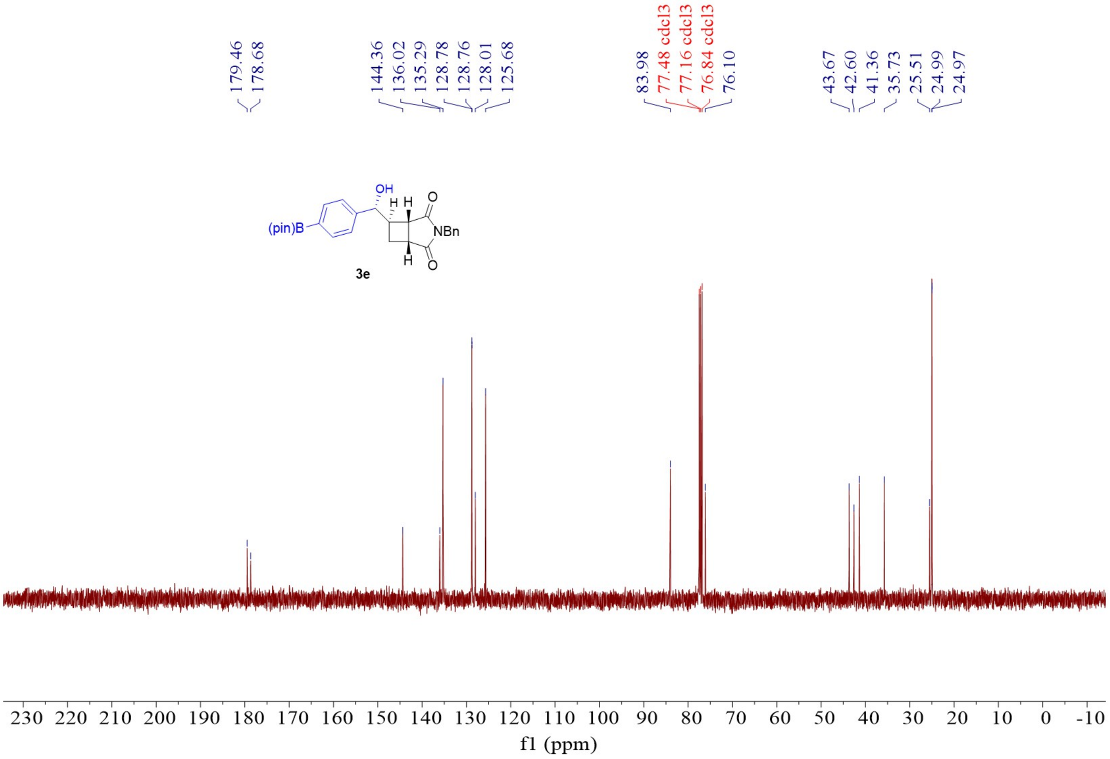

179.46
178.68
144.36
136.02
135.29
128.78
128.76
128.01
125.68
83.98
77.48 cdcl3
77.16 cdcl3
76.84 cdcl3
76.10
43.67
42.60
41.36
35.73
25.51
24.99
24.97
OH
H
H
O
(pin)B
NBn
H
O
3e
230 220 210 200 190 180 170 160 150 140 130 120 110 100 90 80 70 60 50 40 30 20 10 0 -10
f1 (ppm)

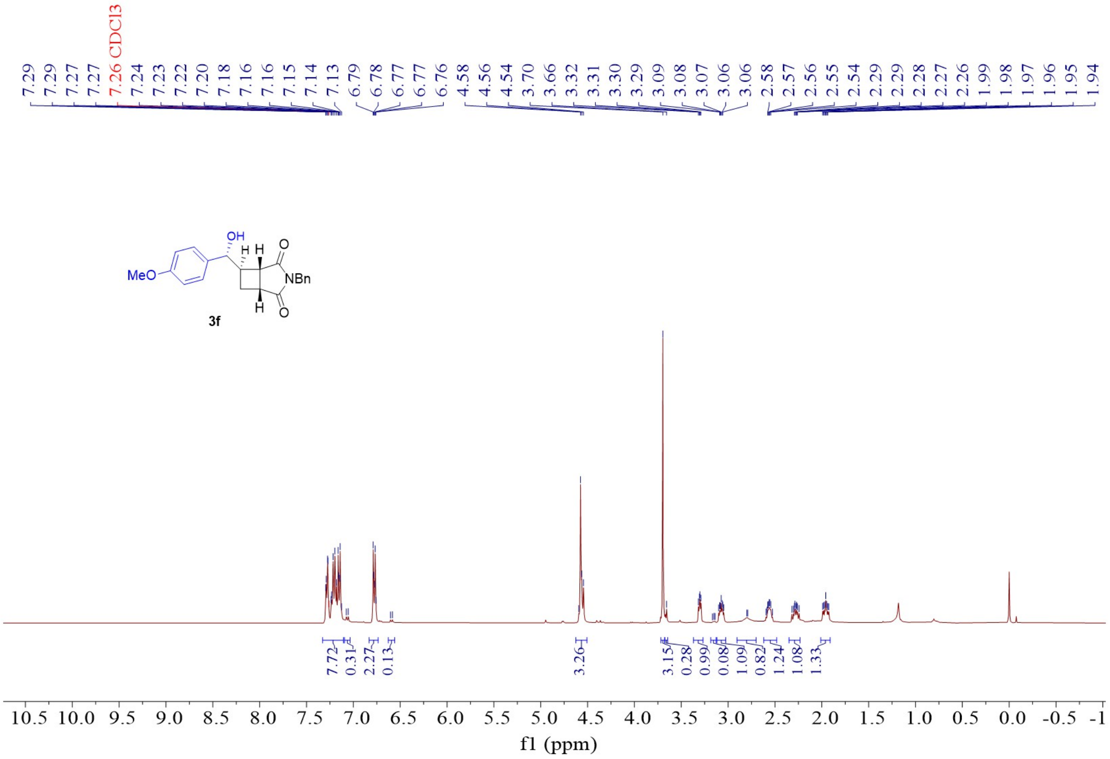

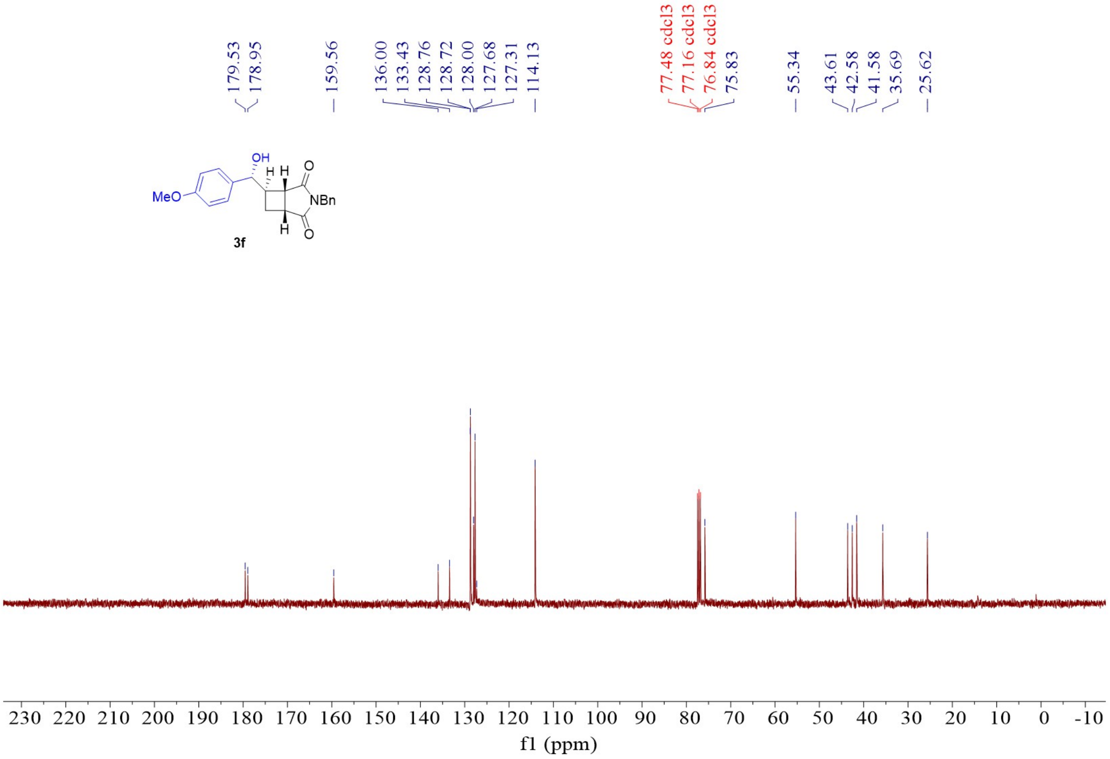

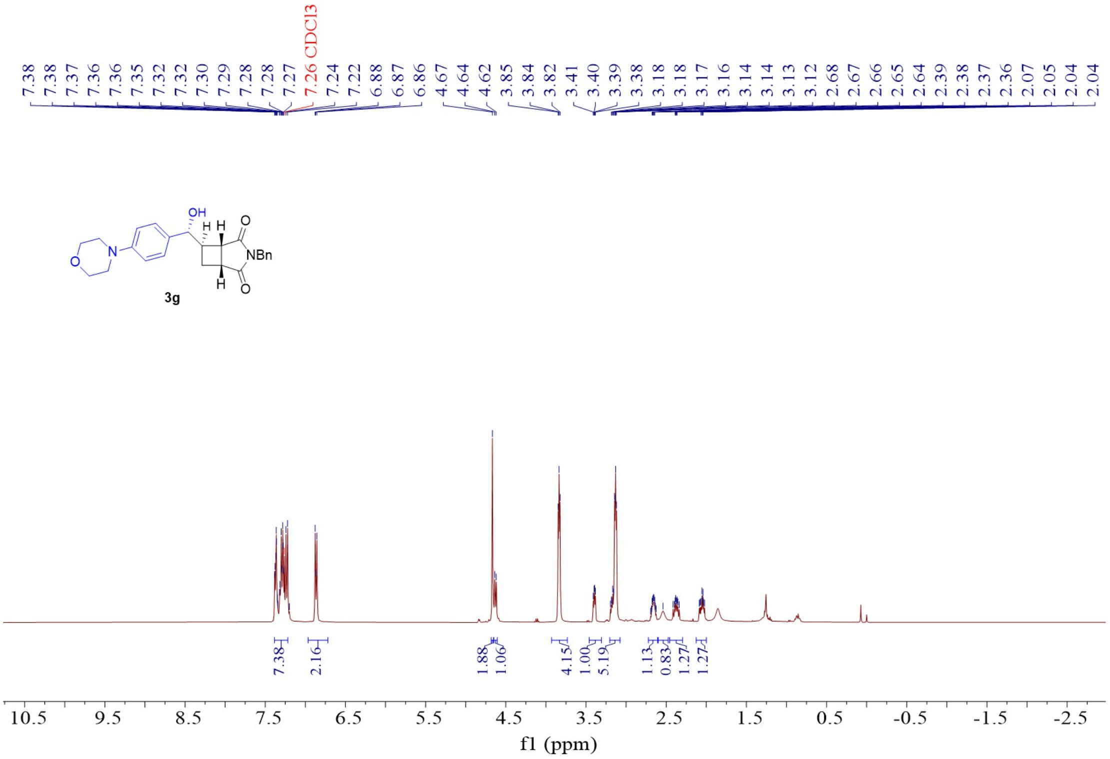

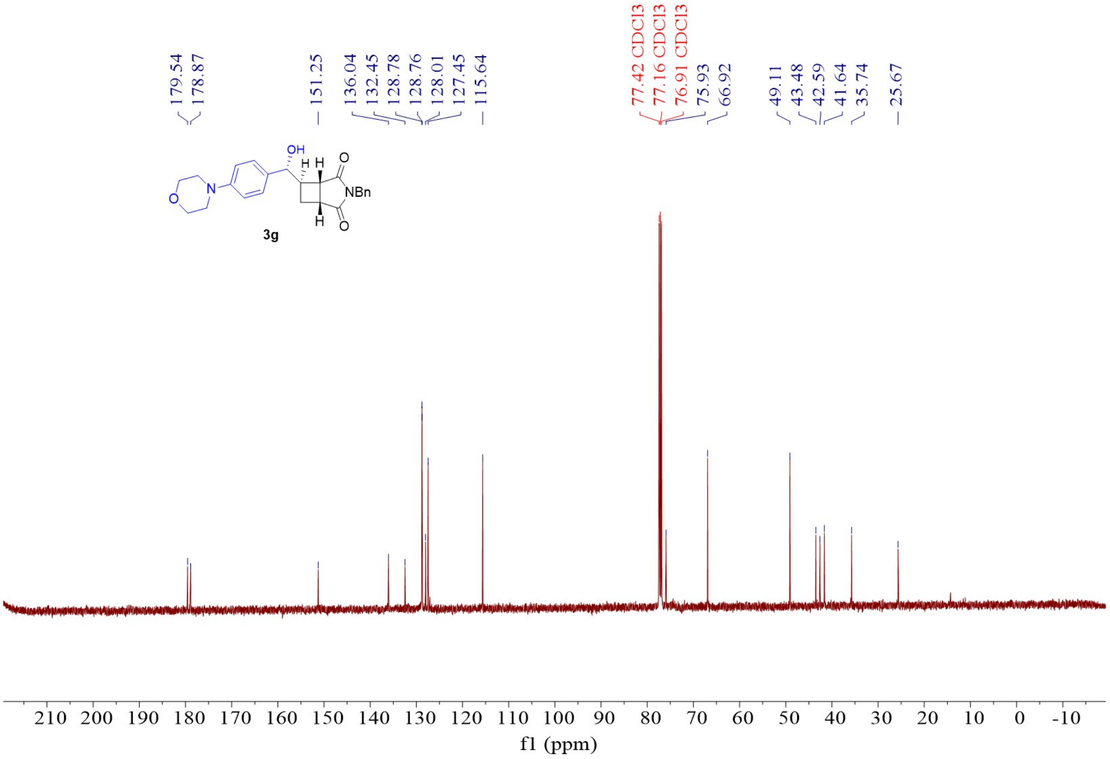

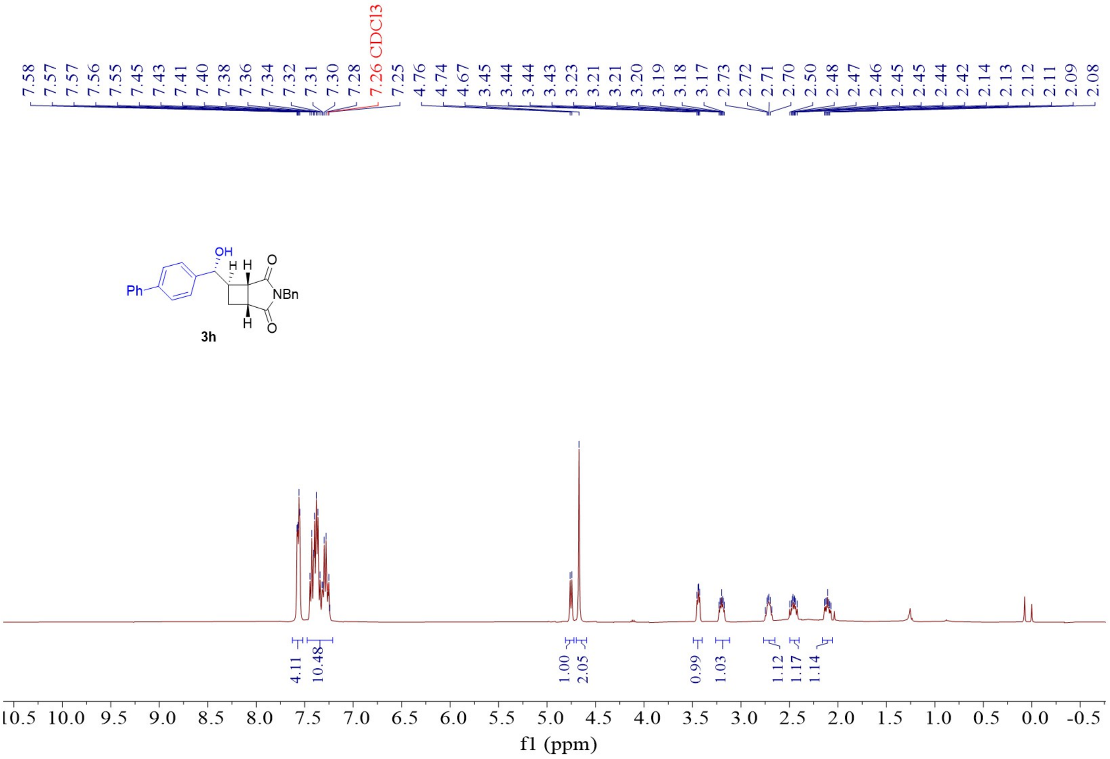
7.58
7.57
7.57
7.56
7.55
7.45
7.43
7.41
7.40
7.38
7.36
7.34
7.32
7.31
7.30
7.28
7.26 CDCl3
7.25
4.76
4.74
4.67
3.45
3.44
3.44
3.43
3.23
3.21
3.21
3.20
3.19
3.18
3.17
2.73
2.72
2.71
2.70
2.50
2.48
2.47
2.46
2.45
2.45
2.44
2.42
2.14
2.13
2.12
2.11
2.09
2.08
OH
H
H
O
Ph
NBn
H
O
3h
4.11
10.48
1.00
2.05
0.99
1.03
1.12
1.17
1.14
10.5 10.0 9.5 9.0 8.5 8.0 7.5 7.0 6.5 6.0 5.5 5.0 4.5 4.0 3.5 3.0 2.5 2.0 1.5 1.0 0.5 0.0 -0.5
f1 (ppm)

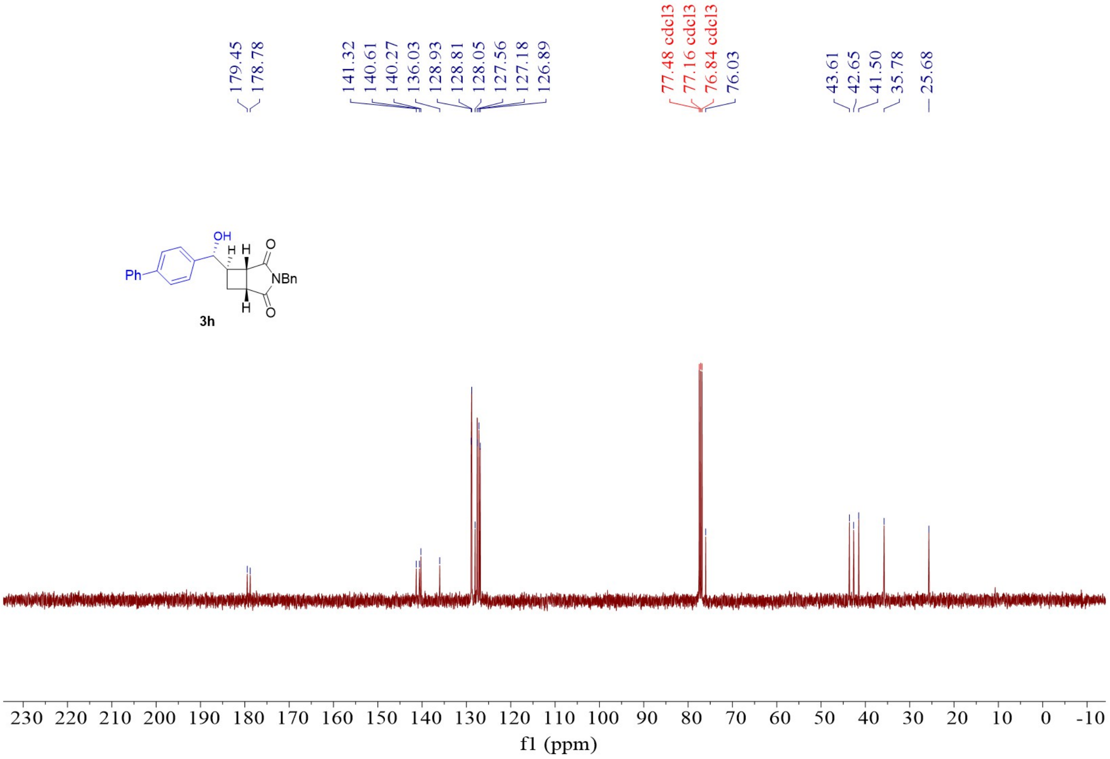
179.45
178.78
141.32
140.61
140.27
136.03
128.93
128.81
128.05
127.56
127.18
126.89
77.48 cdcl3
77.16 cdcl3
76.84 cdcl3
76.03
43.61
42.65
41.50
35.78
25.68
OH
H
H
O
Ph
NBn
H
O
3h
230 220 210 200 190 180 170 160 150 140 130 120 110 100 90 80 70 60 50 40 30 20 10 0 -10
f1 (ppm)

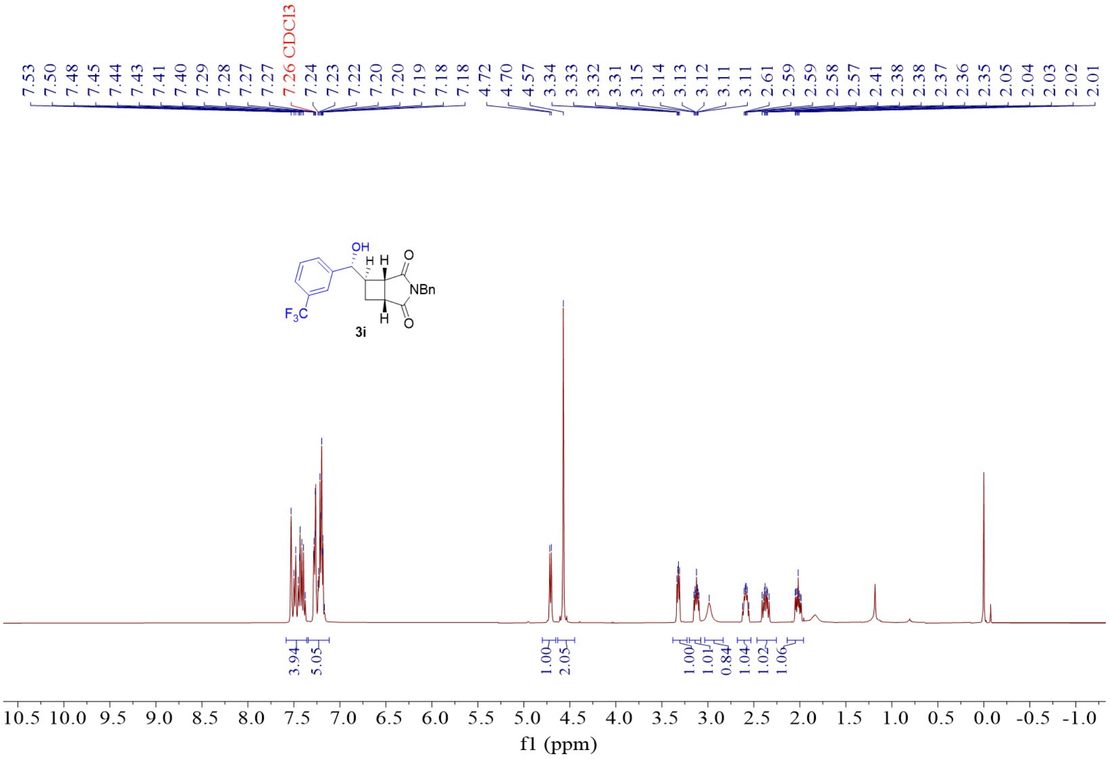

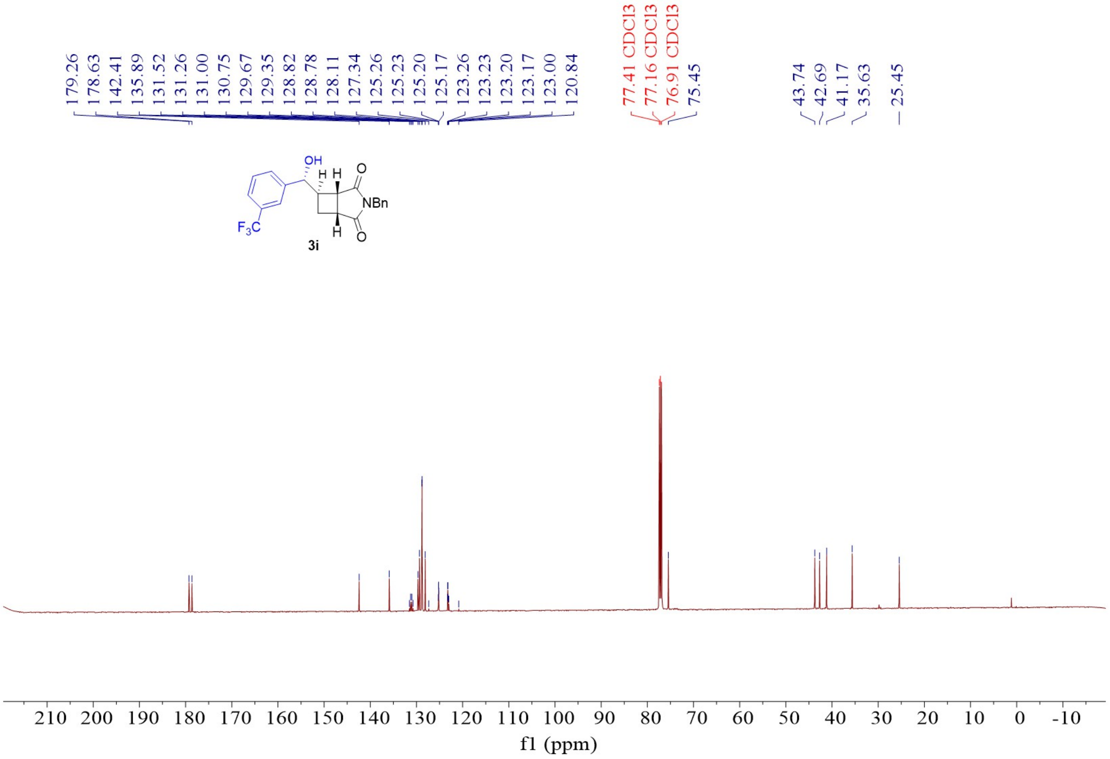

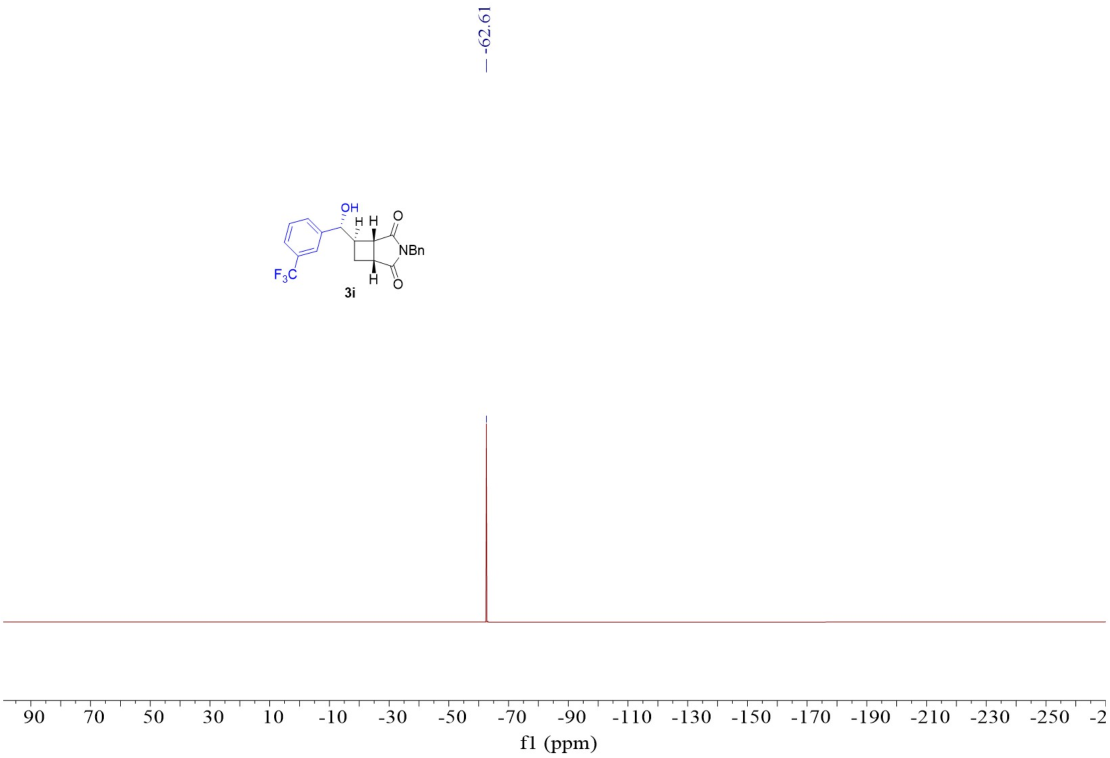
-62.61
OH
H
H
O
NBn
O
F_3C
3i
90 70 50 30 10 -10 -30 -50 -70 -90 -110 -130 -150 -170 -190 -210 -230 -250 -2
f1 (ppm)

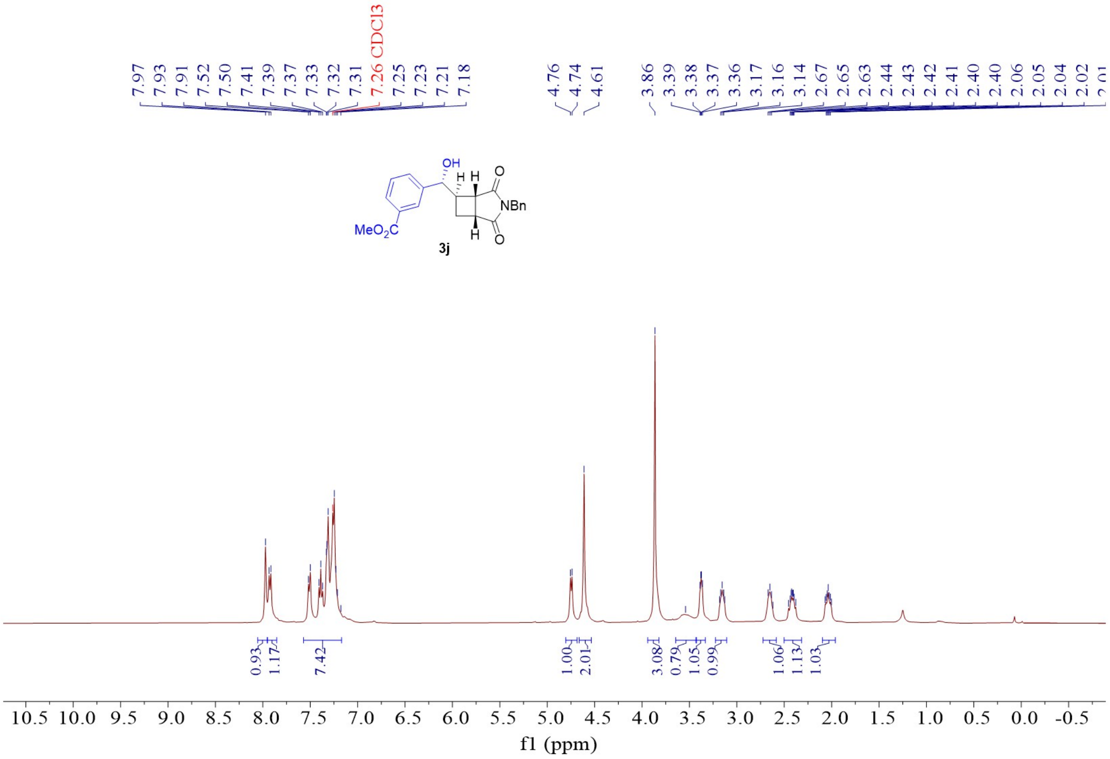
7.97 7.93 7.91 7.52 7.50 7.41 7.39 7.37 7.33 7.32 7.31 7.26 CDCl3 7.25 7.23 7.21 7.18
4.76 4.74 4.61
3.86 3.39 3.38 3.37 3.36 3.17 3.16 3.14 2.67 2.65 2.63 2.44 2.43 2.42 2.41 2.40 2.40 2.06 2.05 2.04 2.02
OH
H
H
O
NBn
O
MeO_2C
3j
0.93 1.17 7.42 1.00 2.01 3.08 0.79 1.05 0.99 1.06 1.13 1.03
10.5 10.0 9.5 9.0 8.5 8.0 7.5 7.0 6.5 6.0 5.5 5.0 4.5 4.0 3.5 3.0 2.5 2.0 1.5 1.0 0.5 0.0 -0.5
f1 (ppm)

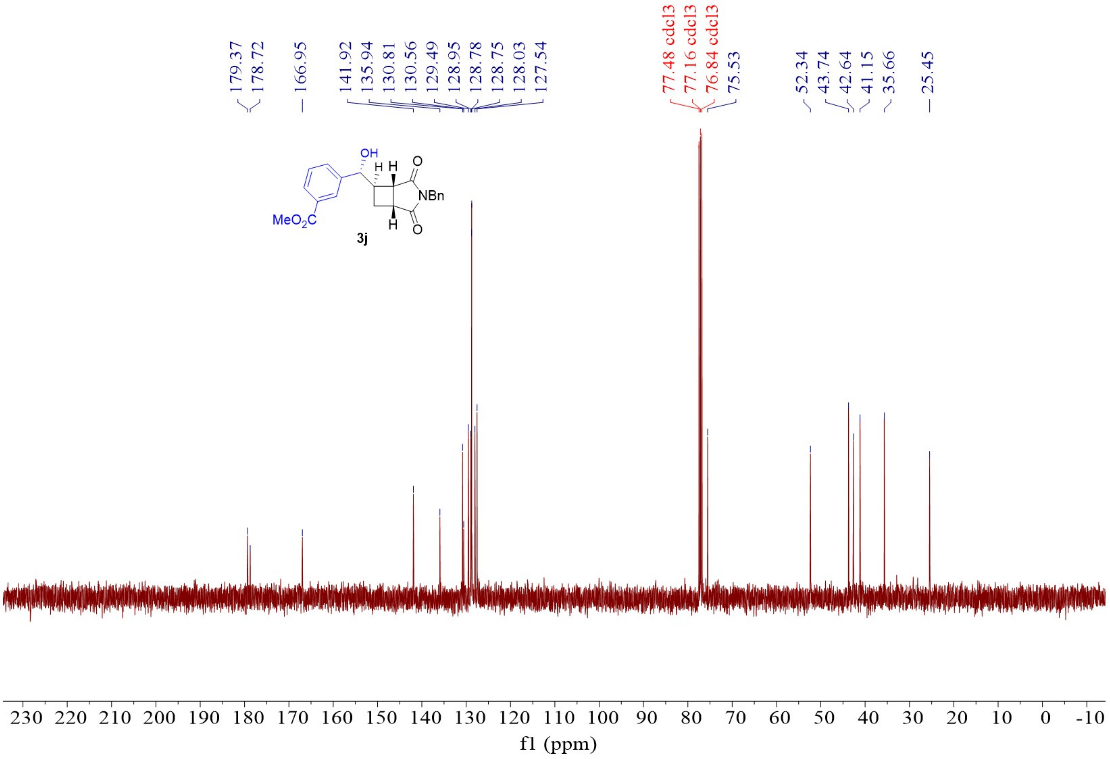
179.37
178.72
166.95
141.92
135.94
130.81
130.56
129.49
128.95
128.78
128.75
128.03
127.54
77.48 cdcl3
77.16 cdcl3
76.84 cdcl3
75.53
52.34
43.74
42.64
41.15
35.66
25.45
OH
H H
O
NBn
MeO2C
H
O
3j
f1 (ppm)

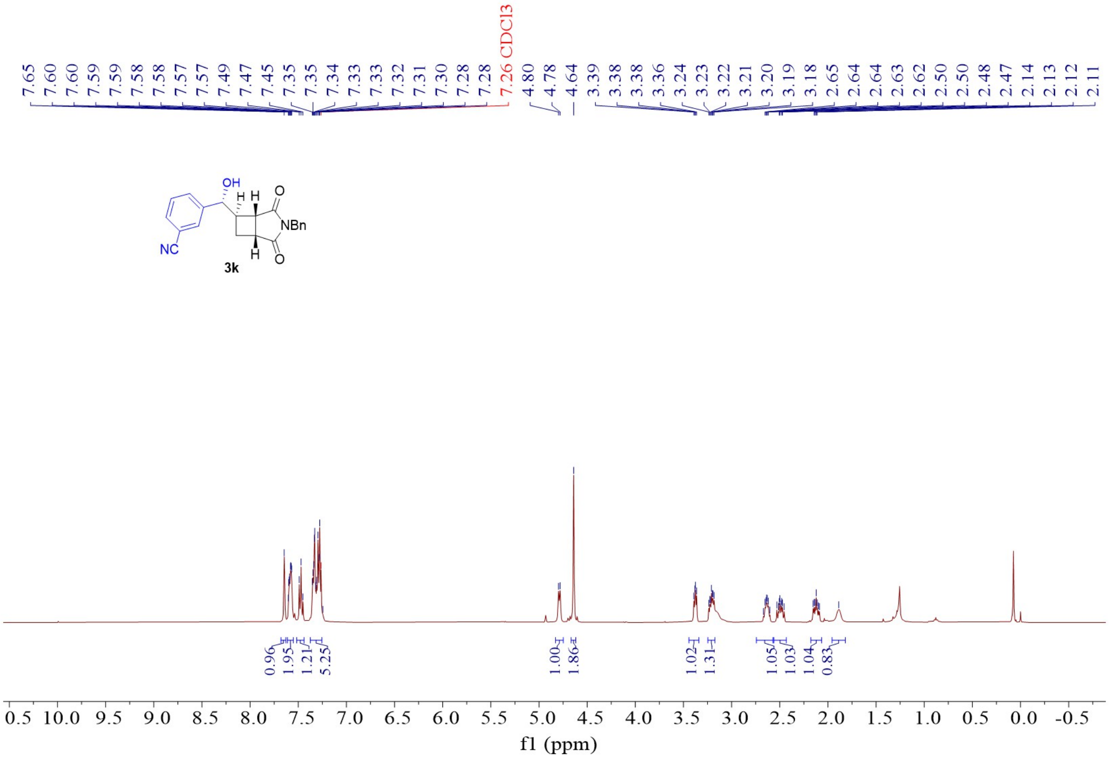
7.65
7.60
7.60
7.59
7.59
7.58
7.58
7.57
7.57
7.49
7.47
7.45
7.35
7.35
7.34
7.33
7.33
7.32
7.31
7.30
7.28
7.28
7.26 CDCl3
4.80
4.78
4.64
3.39
3.38
3.38
3.36
3.24
3.23
3.22
3.21
3.20
3.19
3.18
2.65
2.64
2.64
2.63
2.62
2.50
2.50
2.48
2.47
2.14
2.13
2.12
2.11
OH
H H
O
NBn
NC
H
O
3k
0.96
1.95
1.21
5.25
1.00
1.86
1.02
1.31
1.05
1.03
1.04
0.83
f1 (ppm)

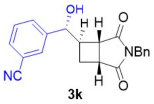
OH
H H
O
NBn
NC
H
O

3k

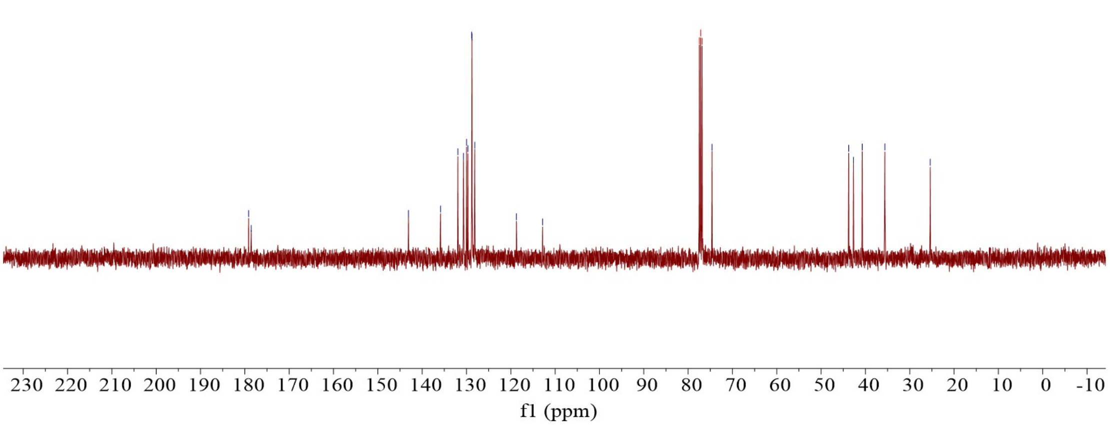
179.16
178.56
143.08
135.85
131.94
130.68
129.97
129.67
128.84
128.75
128.13
118.73
112.84
77.48 cdcl3
77.16 cdcl3
76.84 cdcl3
74.62
43.76
42.71
40.72
35.62
25.37
230 220 210 200 190 180 170 160 150 140 130 120 110 100 90 80 70 60 50 40 30 20 10 0 -10
f1 (ppm)

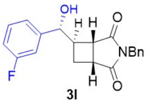
OH
H
O
NBn
F
H
O

3l

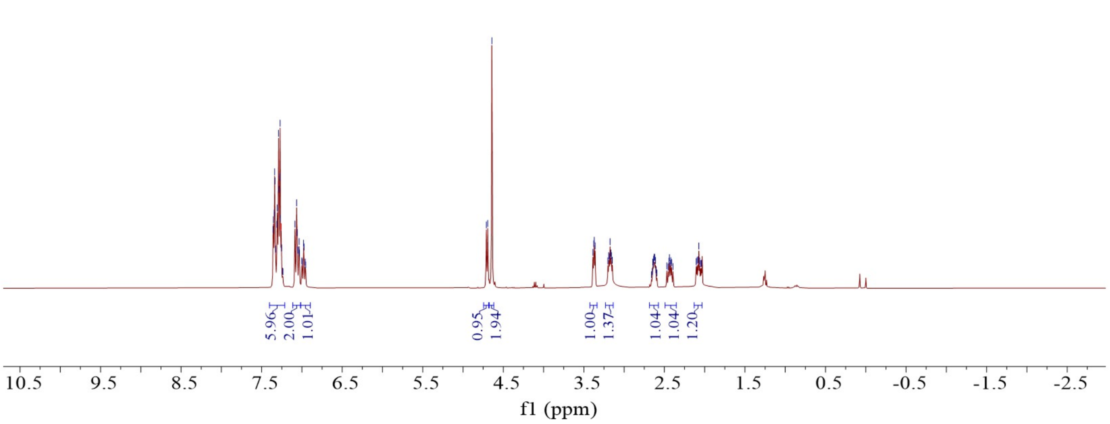
7.36
7.35
7.34
7.34
7.33
7.33
7.31
7.31
7.29
7.29
7.28
7.27
7.27
7.26 CDCl3
7.25
7.09
7.07
7.06
7.06
7.04
7.04
7.03
7.00
6.98
6.97
4.71
4.69
4.64
3.39
3.38
3.37
3.36
3.20
3.19
3.18
3.17
3.16
3.16
3.15
2.63
2.63
2.62
2.61
2.44
2.44
2.42
2.09
2.08
2.07
2.06
5.96
2.00
1.01
0.95
1.94
1.00
1.37
1.04
1.04
1.20
10.5 9.5 8.5 7.5 6.5 5.5 4.5 3.5 2.5 1.5 0.5 -0.5 -1.5 -2.5
f1 (ppm)

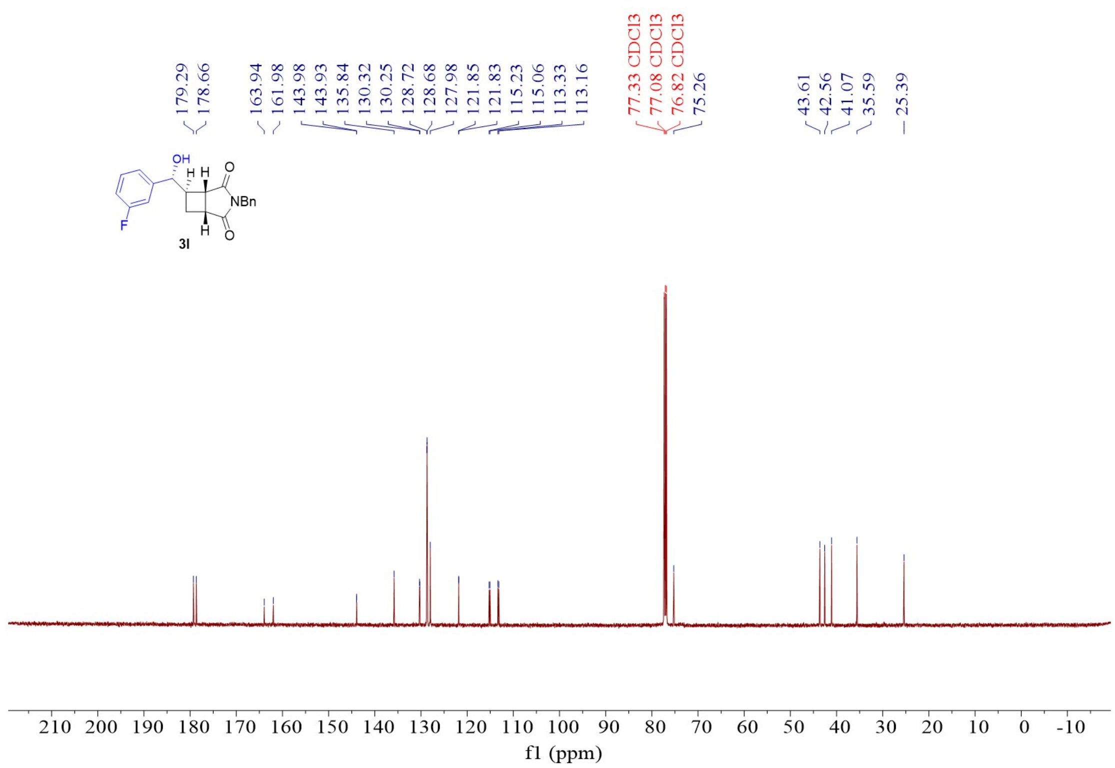
179.29
178.66
163.94
161.98
143.98
143.93
135.84
130.32
130.25
128.72
128.68
127.98
121.85
121.83
115.23
115.06
113.33
113.16
77.33 CDCl3
77.08 CDCl3
76.82 CDCl3
75.26
43.61
42.56
41.07
35.59
25.39
OH
H H
O
NBn
F
H
O
3l
210 200 190 180 170 160 150 140 130 120 110 100 90 80 70 60 50 40 30 20 10 0 -10
f1 (ppm)

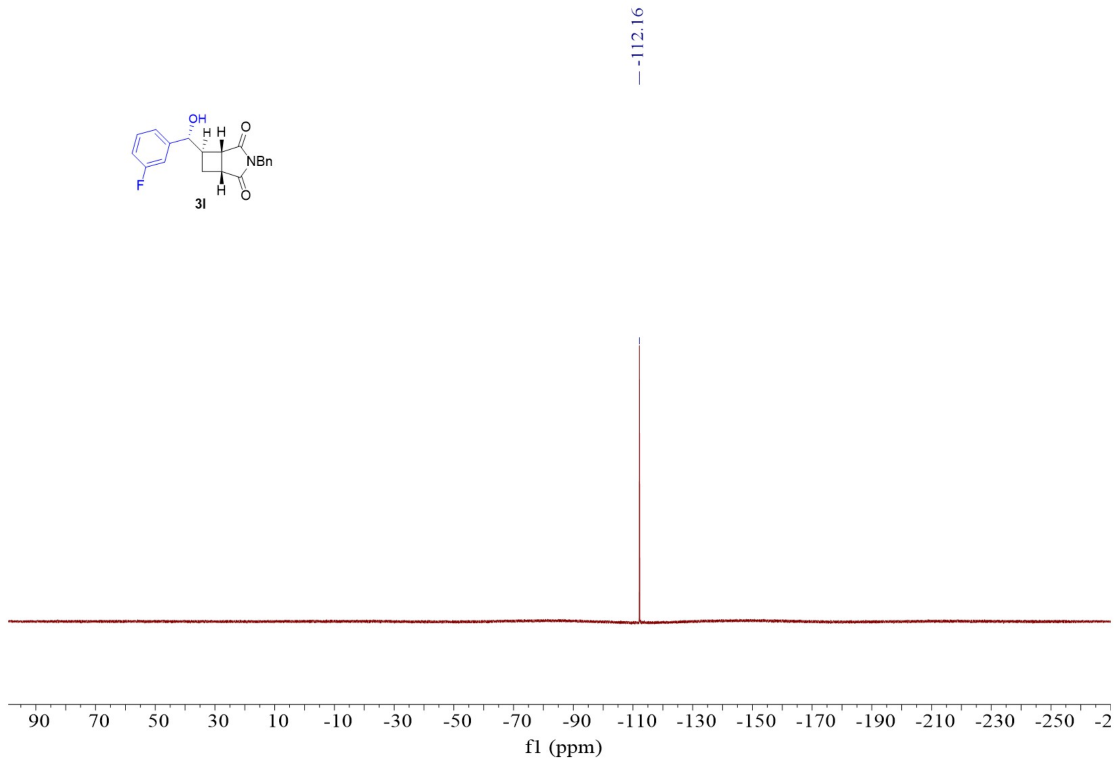
-112.16
OH
H H
O
NBn
F
H
O
3l
90 70 50 30 10 -10 -30 -50 -70 -90 -110 -130 -150 -170 -190 -210 -230 -250 -2
f1 (ppm)

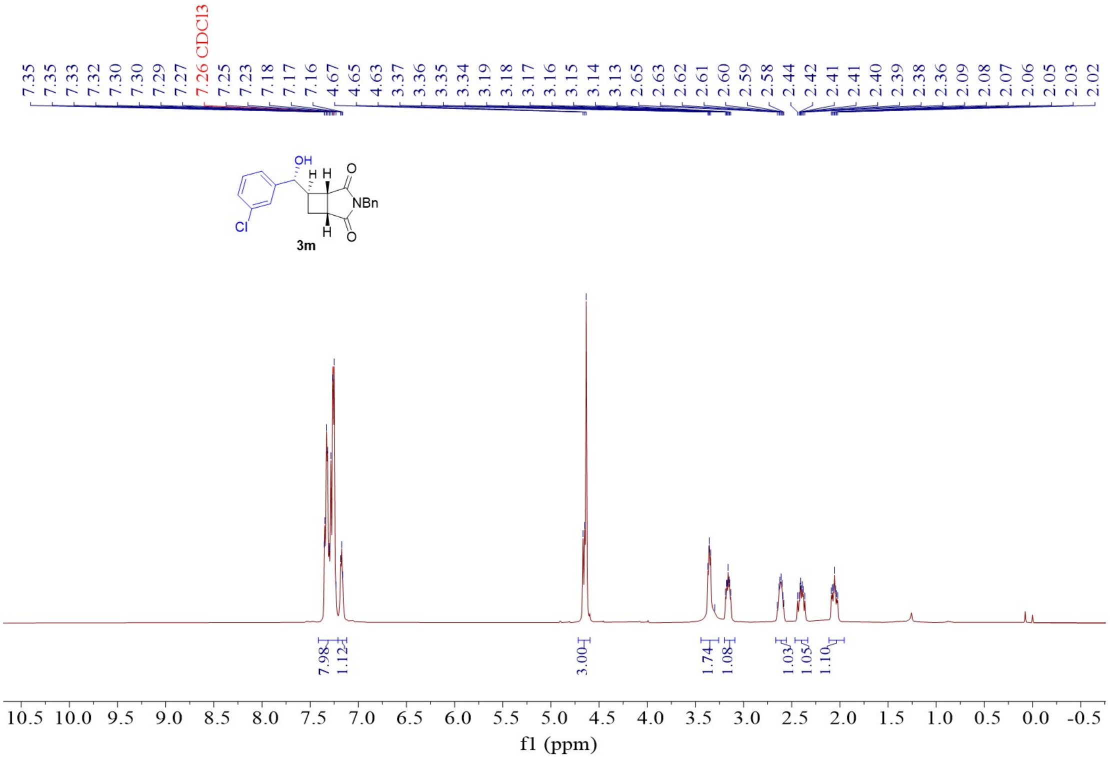
7.35
7.35
7.33
7.32
7.30
7.30
7.29
7.27
7.26 CDCl3
7.25
7.23
7.18
7.17
7.16
4.67
4.65
4.63
3.37
3.36
3.35
3.34
3.19
3.18
3.17
3.16
3.15
3.14
3.13
2.65
2.63
2.62
2.61
2.60
2.59
2.58
2.44
2.42
2.41
2.41
2.40
2.39
2.38
2.36
2.09
2.08
2.07
2.06
2.05
2.03
2.02
OH
H
H
O
NBn
Cl
H
O
3m
7.98
1.12
3.00
1.74
1.08
1.03
1.05
1.10
10.5 10.0 9.5 9.0 8.5 8.0 7.5 7.0 6.5 6.0 5.5 5.0 4.5 4.0 3.5 3.0 2.5 2.0 1.5 1.0 0.5 0.0 -0.5
f1 (ppm)

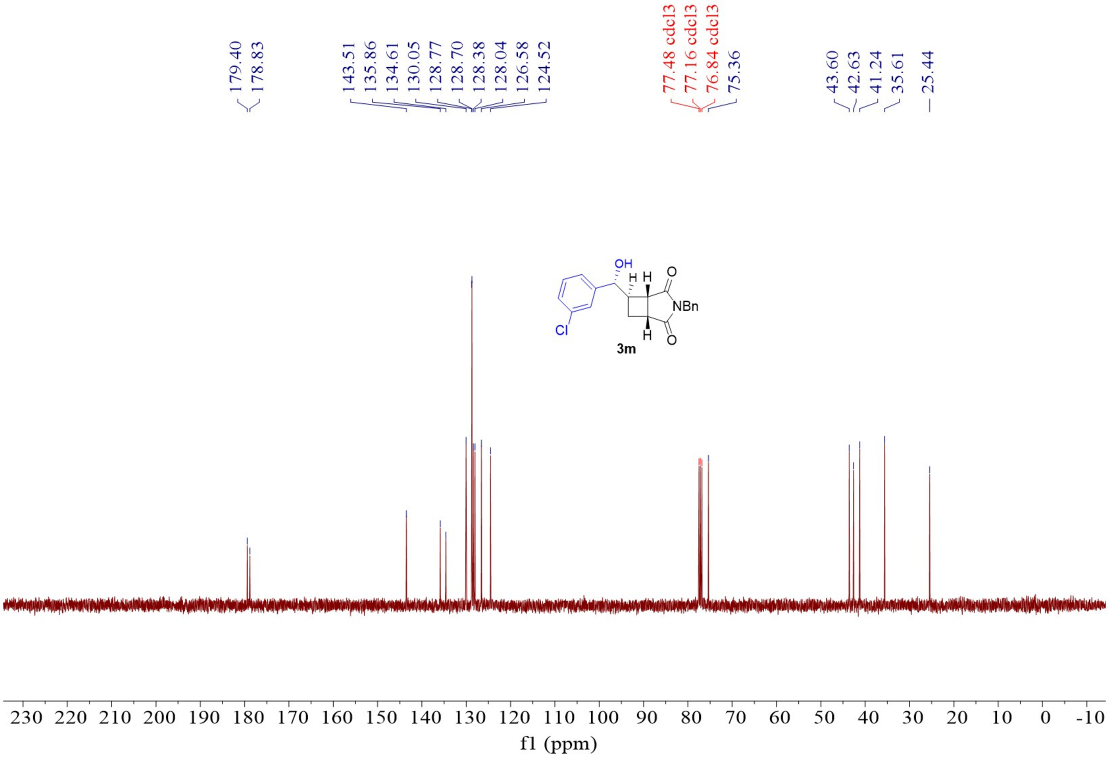
179.40
178.83
143.51
135.86
134.61
130.05
128.77
128.70
128.38
128.04
126.58
124.52
77.48 cdcl3
77.16 cdcl3
76.84 cdcl3
75.36
43.60
42.63
41.24
35.61
25.44
OH
H
H
O
NBn
Cl
H
O
3m
230 220 210 200 190 180 170 160 150 140 130 120 110 100 90 80 70 60 50 40 30 20 10 0 -10
f1 (ppm)

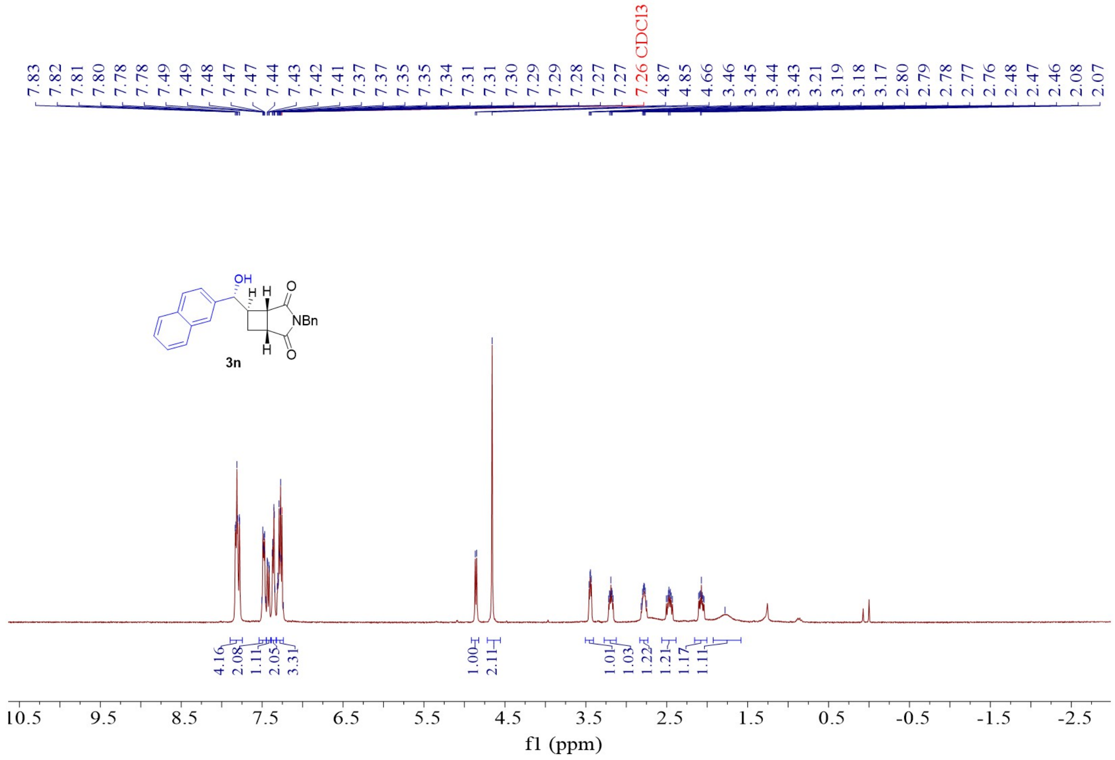

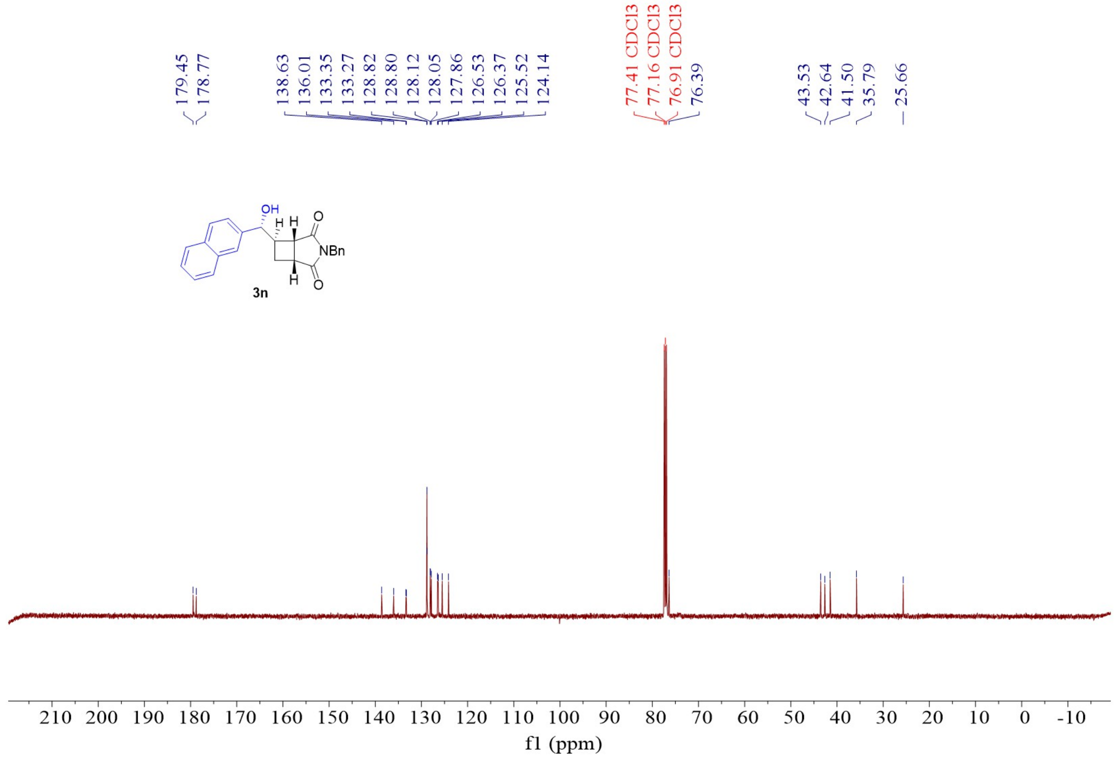

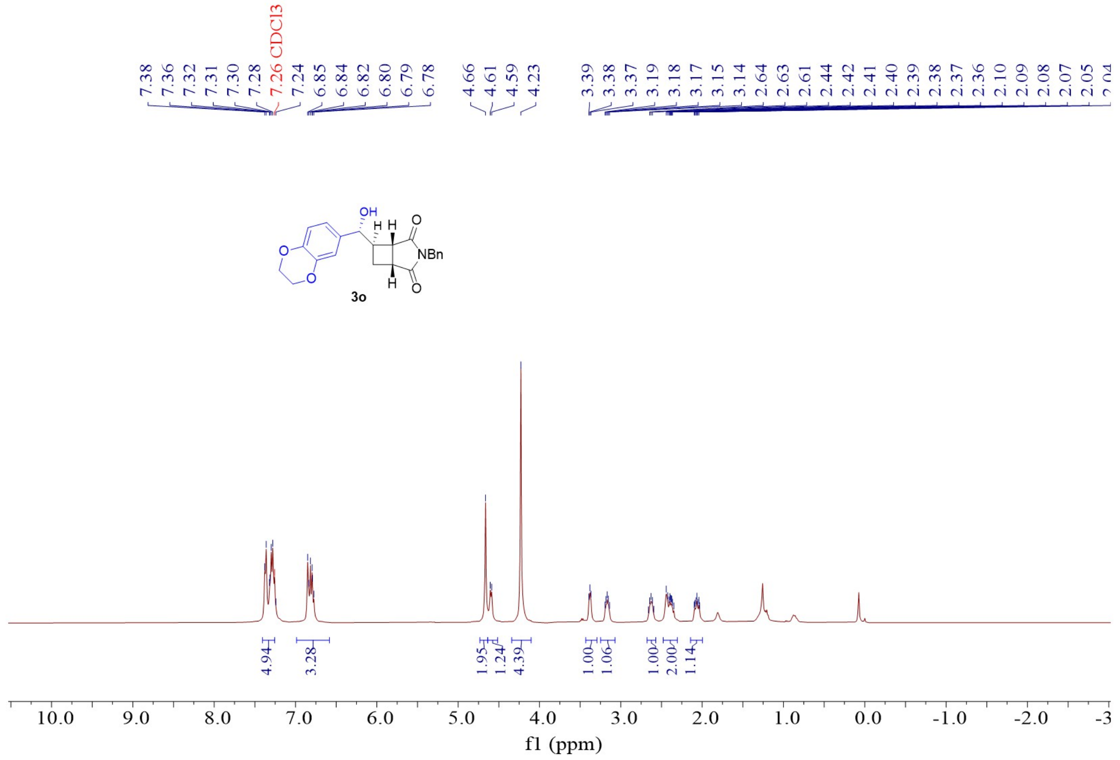

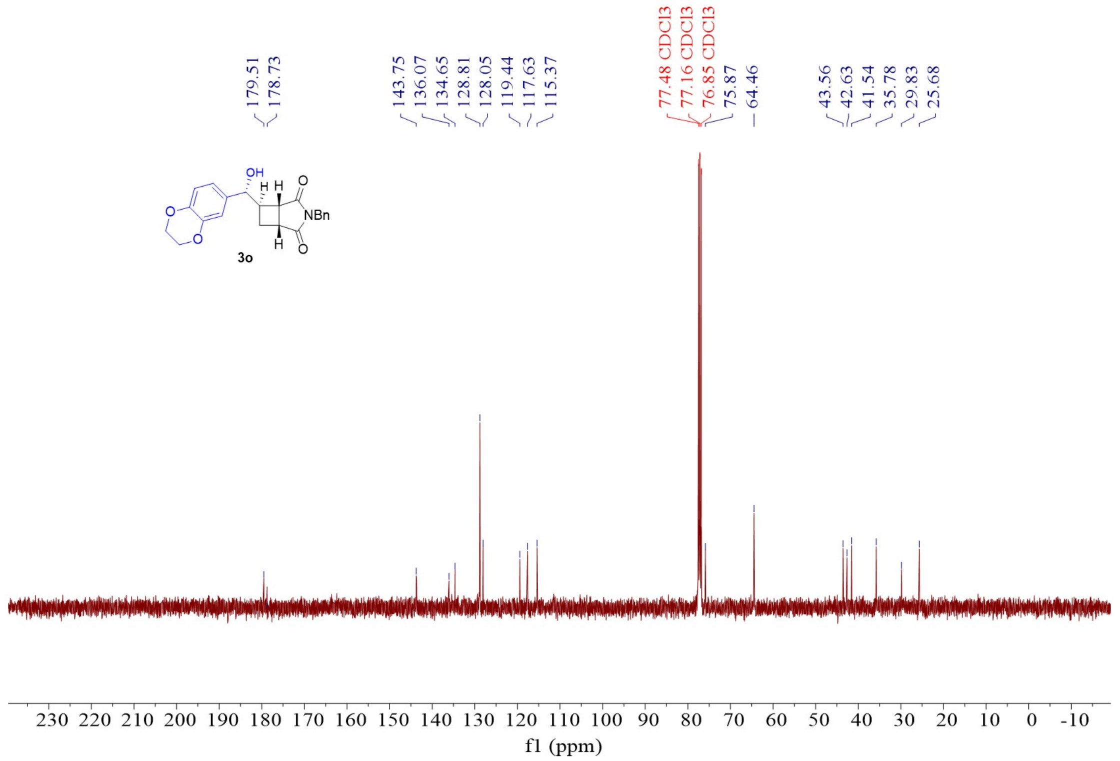

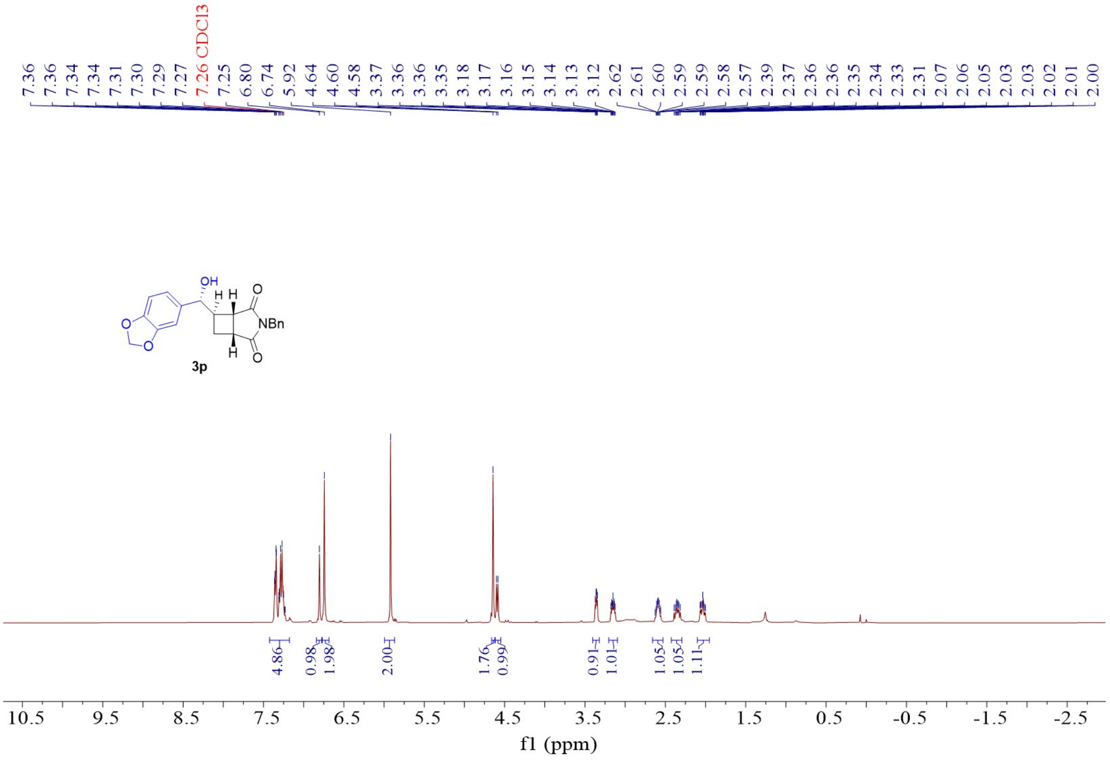
7.36
7.36
7.34
7.34
7.31
7.30
7.29
7.27
7.26 CDCl3
7.25
6.80
6.74
5.92
4.64
4.60
4.58
3.37
3.36
3.36
3.35
3.18
3.17
3.16
3.15
3.14
3.13
3.12
2.62
2.61
2.60
2.59
2.59
2.58
2.57
2.39
2.37
2.36
2.36
2.35
2.34
2.33
2.31
2.07
2.06
2.05
2.03
2.03
2.02
2.01
2.00
OH
H
H
O
NBn
H
O
O
O
3p
4.86
0.98
1.98
2.00
1.76
0.99
0.91
1.01
1.05
1.05
1.11
f1 (ppm)

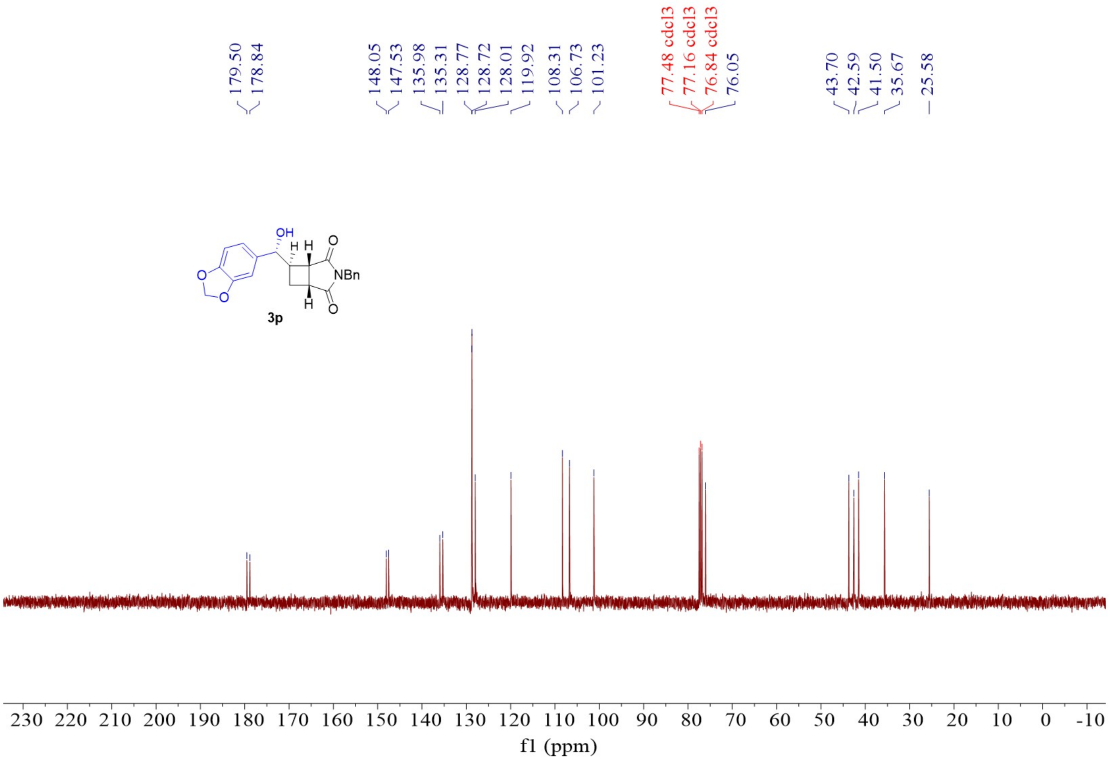
179.50
178.84
148.05
147.53
135.98
135.31
128.77
128.72
128.01
119.92
108.31
106.73
101.23
77.48 cdcl3
77.16 cdcl3
76.84 cdcl3
76.05
43.70
42.59
41.50
35.67
25.58
OH
H
H
O
NBn
H
O
O
O
3p
f1 (ppm)

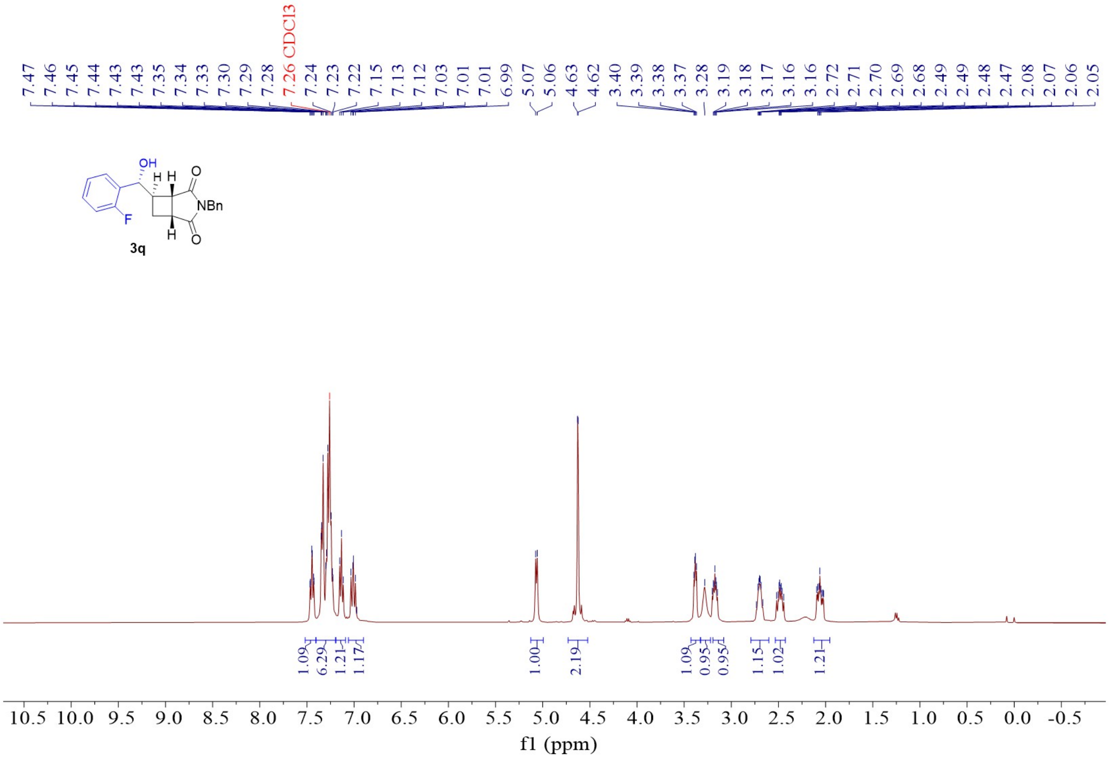

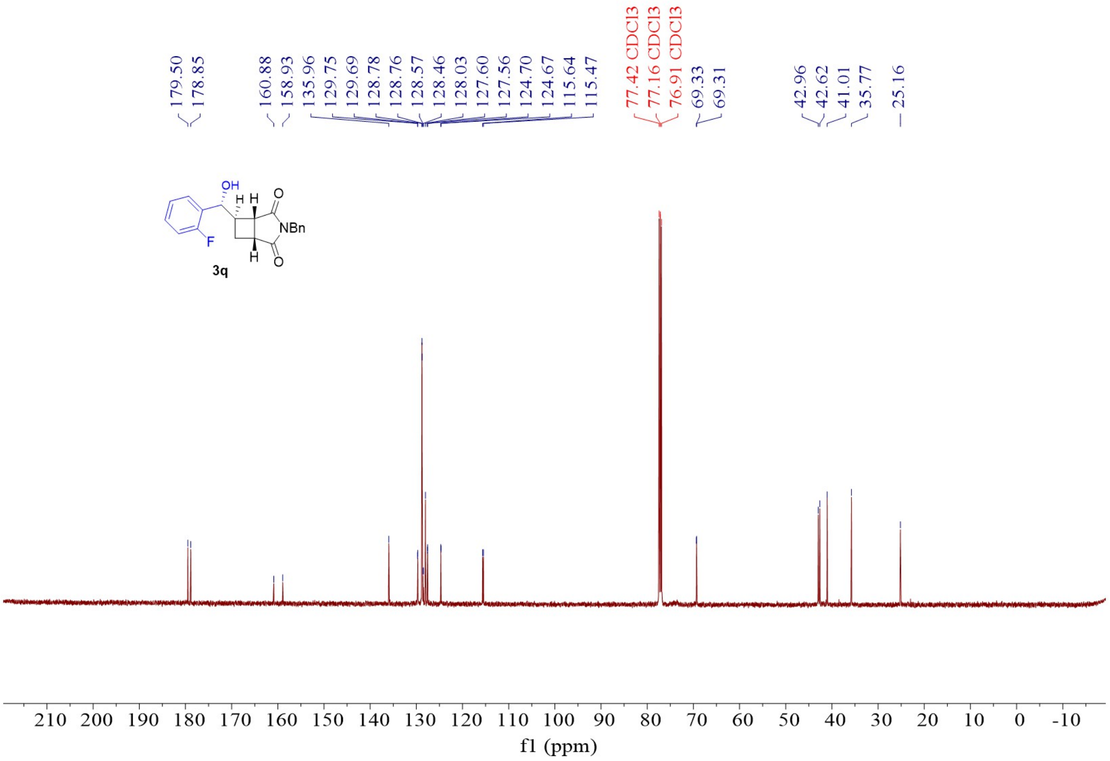

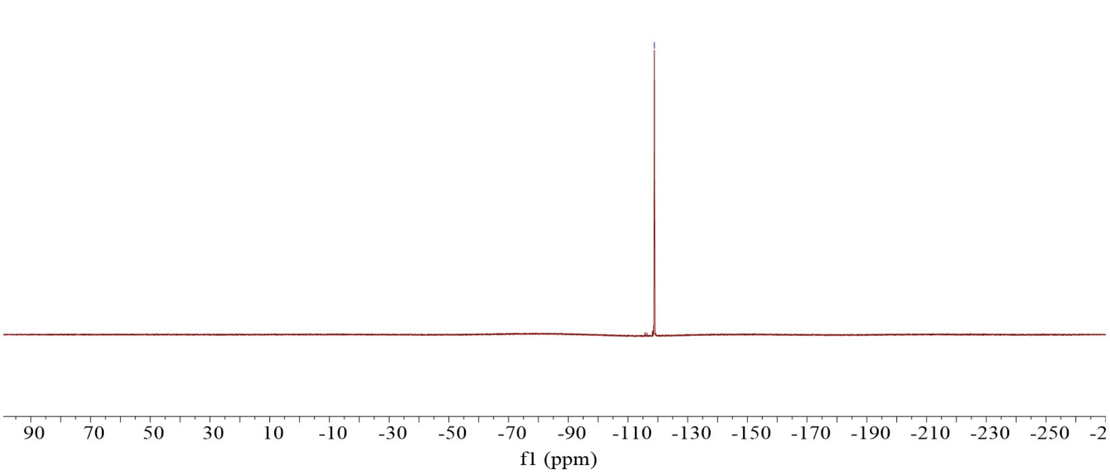
-118.86
90 70 50 30 10 -10 -30 -50 -70 -90 -110 -130 -150 -170 -190 -210 -230 -250
f1 (ppm)

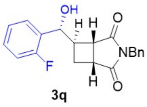
OH
H
H
O
NBn
F
H
O

3q

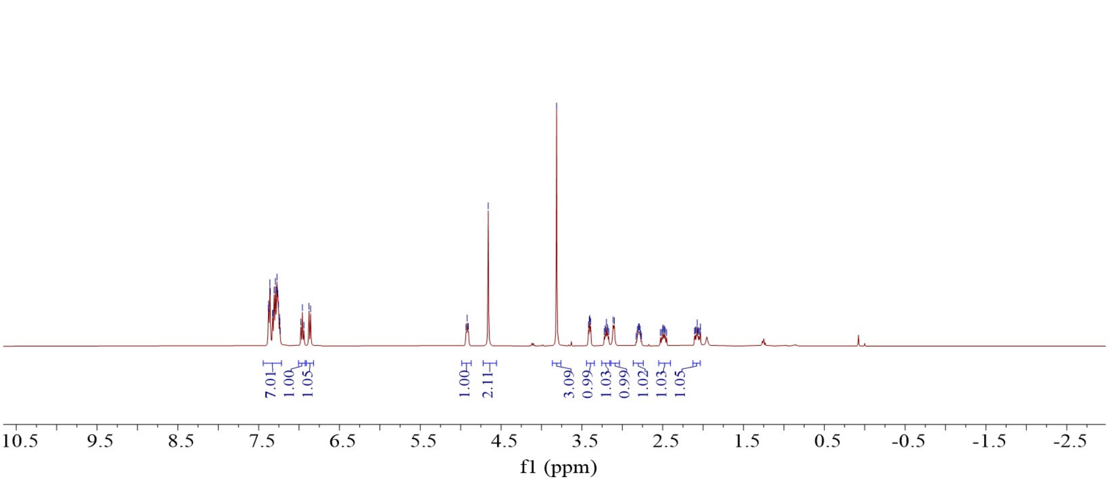
7.38 7.38 7.36 7.36 7.33 7.32 7.31 7.29 7.28 7.27 7.27 7.26 7.26 CDCl3 7.25 7.24 7.24 6.98 6.96 6.94 6.88 6.86 4.93 4.92 4.90 4.66 3.81 3.42 3.41 3.40 3.39 3.21 3.21 3.20 3.19 3.18 3.11 3.10 2.81 2.80 2.80 2.79 2.78 2.50 2.49 2.48 2.48 2.08 2.07 2.06 2.03
7.01 1.00 1.05 1.00 2.11 3.09 0.99 1.03 0.99 1.02 1.03 1.05
10.5 9.5 8.5 7.5 6.5 5.5 4.5 3.5 2.5 1.5 0.5 -0.5 -1.5 -2.5
f1 (ppm)

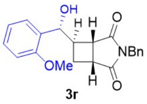
OH
H
H
O
NBn
OMe
H
O

3r

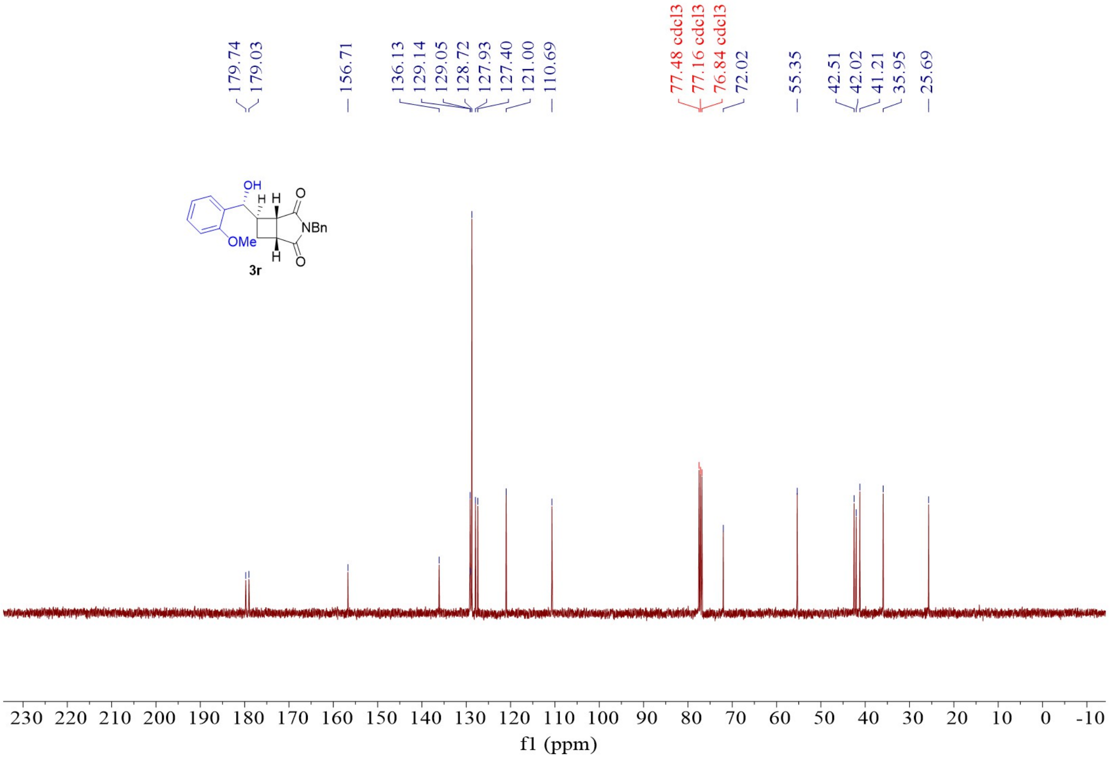

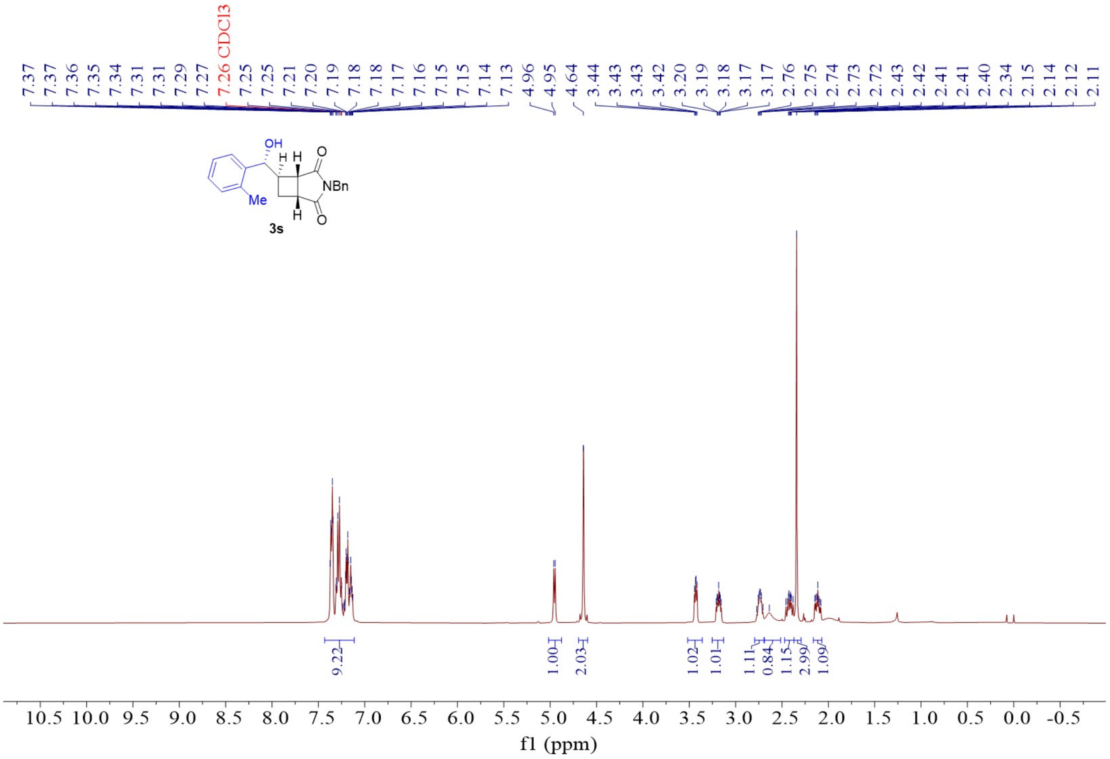

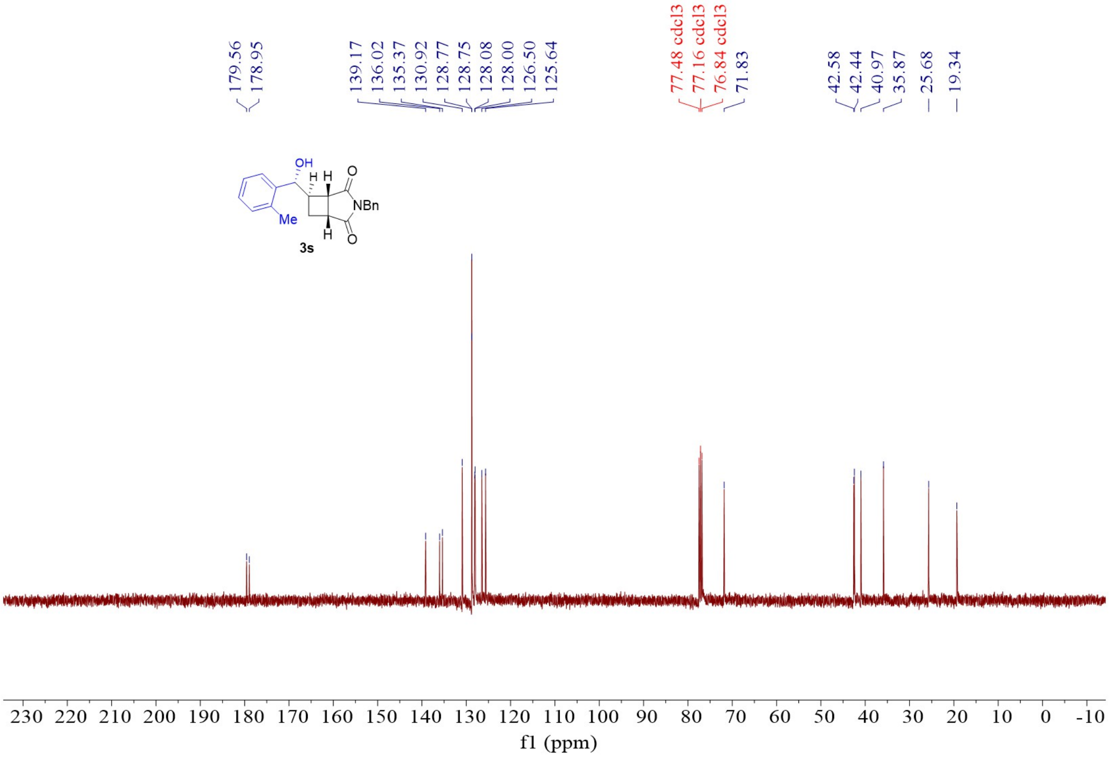
179.56
178.95
139.17
136.02
135.37
130.92
128.77
128.75
128.08
128.00
126.50
125.64
77.48 cdcl3
77.16 cdcl3
76.84 cdcl3
71.83
42.58
42.44
40.97
35.87
25.68
19.34
OH
H
H
O
NBn
Me
H
O
3s
230 220 210 200 190 180 170 160 150 140 130 120 110 100 90 80 70 60 50 40 30 20 10 0 -10
f1 (ppm)

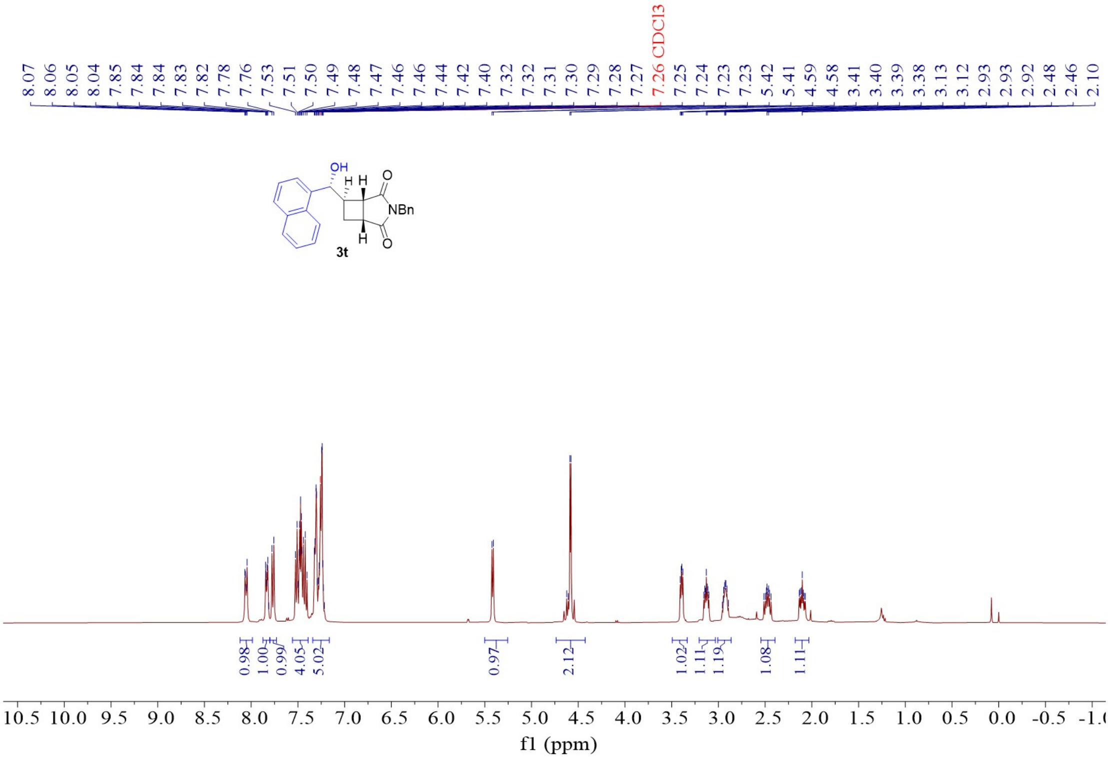
8.07
8.06
8.05
8.04
7.85
7.84
7.84
7.83
7.82
7.78
7.76
7.53
7.51
7.50
7.49
7.48
7.47
7.46
7.46
7.44
7.42
7.40
7.32
7.32
7.31
7.30
7.29
7.28
7.27
7.26 CDCl3
7.25
7.24
7.23
7.23
5.42
5.41
4.59
4.58
3.41
3.40
3.39
3.38
3.13
3.12
2.93
2.93
2.92
2.48
2.46
2.10
OH
H
H
O
NBn
H
O
3t
0.98
1.00
0.99
4.05
5.02
0.97
2.12
1.02
1.11
1.19
1.08
1.11
10.5 10.0 9.5 9.0 8.5 8.0 7.5 7.0 6.5 6.0 5.5 5.0 4.5 4.0 3.5 3.0 2.5 2.0 1.5 1.0 0.5 0.0 -0.5 -1.0
f1 (ppm)

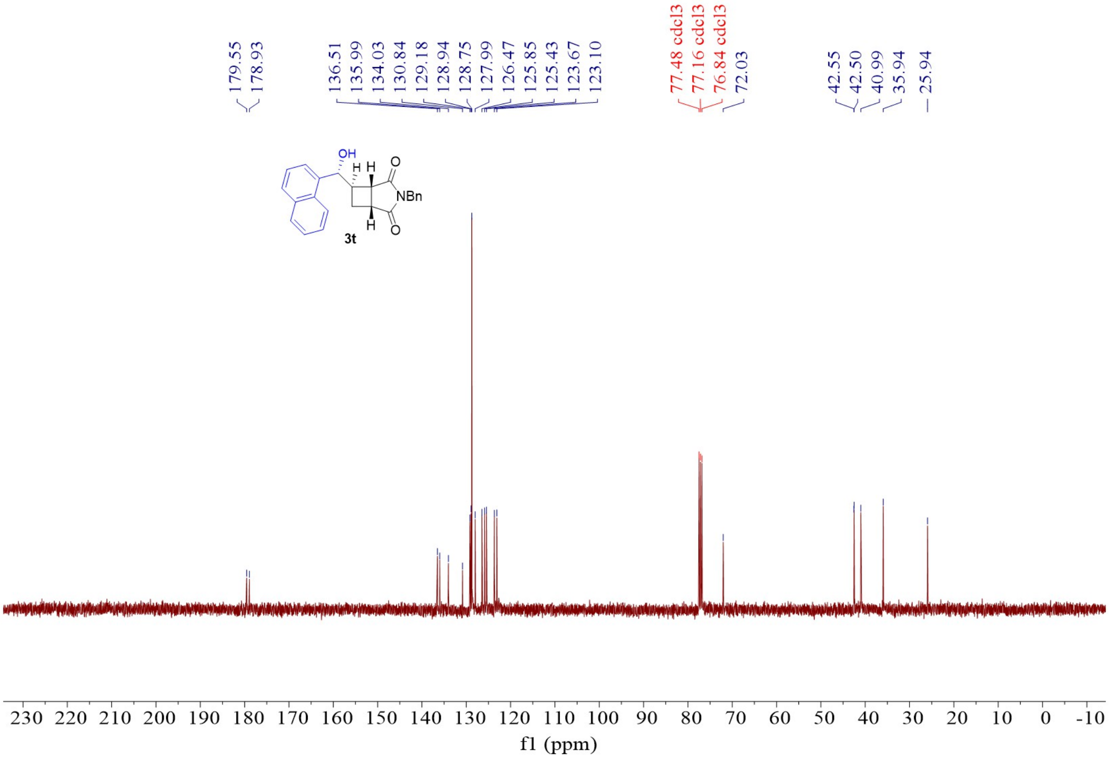
179.55
178.93
136.51
135.99
134.03
130.84
129.18
128.94
128.75
127.99
126.47
125.85
125.43
123.67
123.10
77.48 cdcl3
77.16 cdcl3
76.84 cdcl3
72.03
42.55
42.50
40.99
35.94
25.94
OH
H
H
O
NBn
O
3t
230 220 210 200 190 180 170 160 150 140 130 120 110 100 90 80 70 60 50 40 30 20 10 0 -10
f1 (ppm)

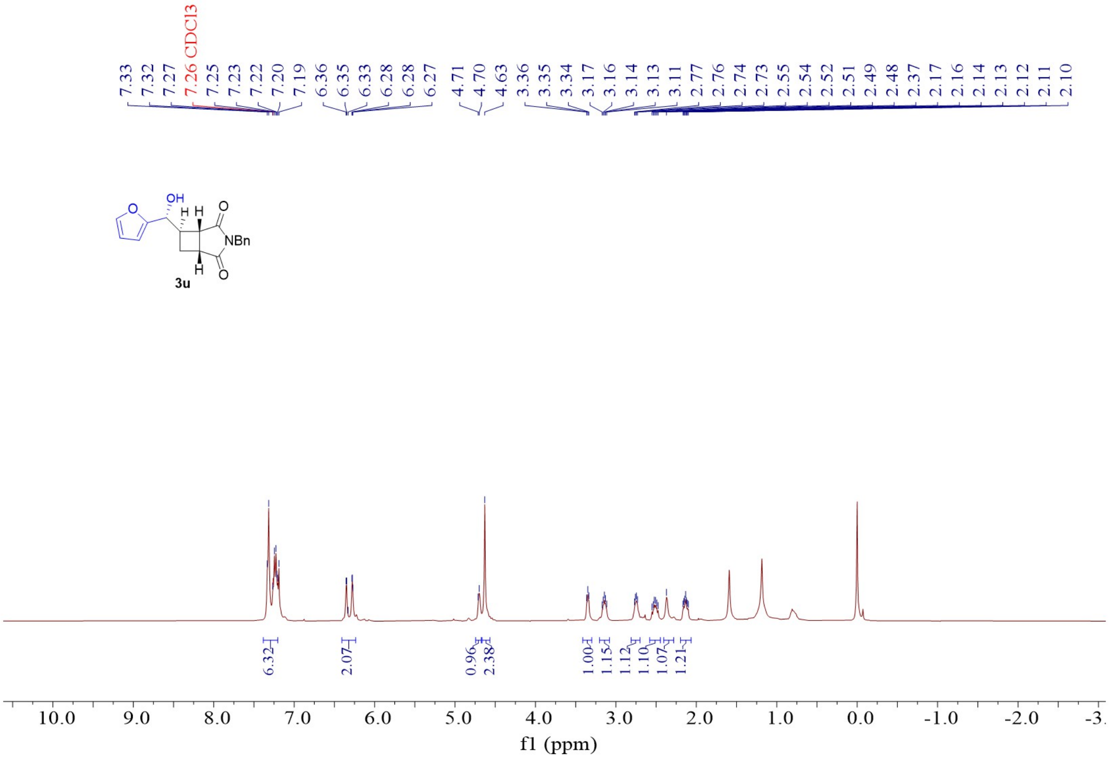
7.33
7.32
7.27
7.26 CDCl3
7.25
7.23
7.22
7.20
7.19
6.36
6.35
6.33
6.28
6.28
6.27
4.71
4.70
4.63
3.36
3.35
3.34
3.17
3.16
3.14
3.13
3.11
2.77
2.76
2.74
2.73
2.55
2.54
2.52
2.51
2.49
2.48
2.37
2.17
2.16
2.14
2.13
2.12
2.11
2.10
O
OH
H
H
O
NBn
H
O
3u
6.32
2.07
0.96
2.38
1.00
1.15
1.12
1.10
1.07
1.21
10.0 9.0 8.0 7.0 6.0 5.0 4.0 3.0 2.0 1.0 0.0 -1.0 -2.0 -3.
f1 (ppm)

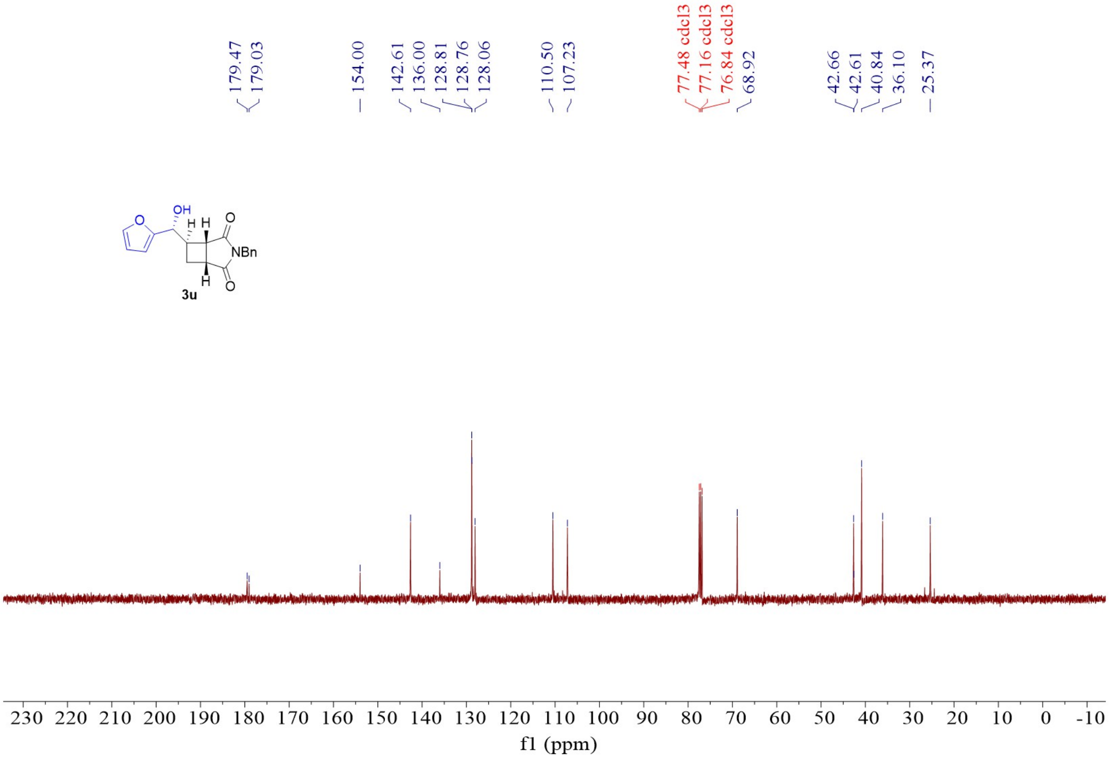
179.47
179.03
154.00
142.61
136.00
128.81
128.76
128.06
110.50
107.23
77.48 cdcl3
77.16 cdcl3
76.84 cdcl3
68.92
42.66
42.61
40.84
36.10
25.37
OH
H
H
O
NBn
O
3u
230 220 210 200 190 180 170 160 150 140 130 120 110 100 90 80 70 60 50 40 30 20 10 0 -10
f1 (ppm)

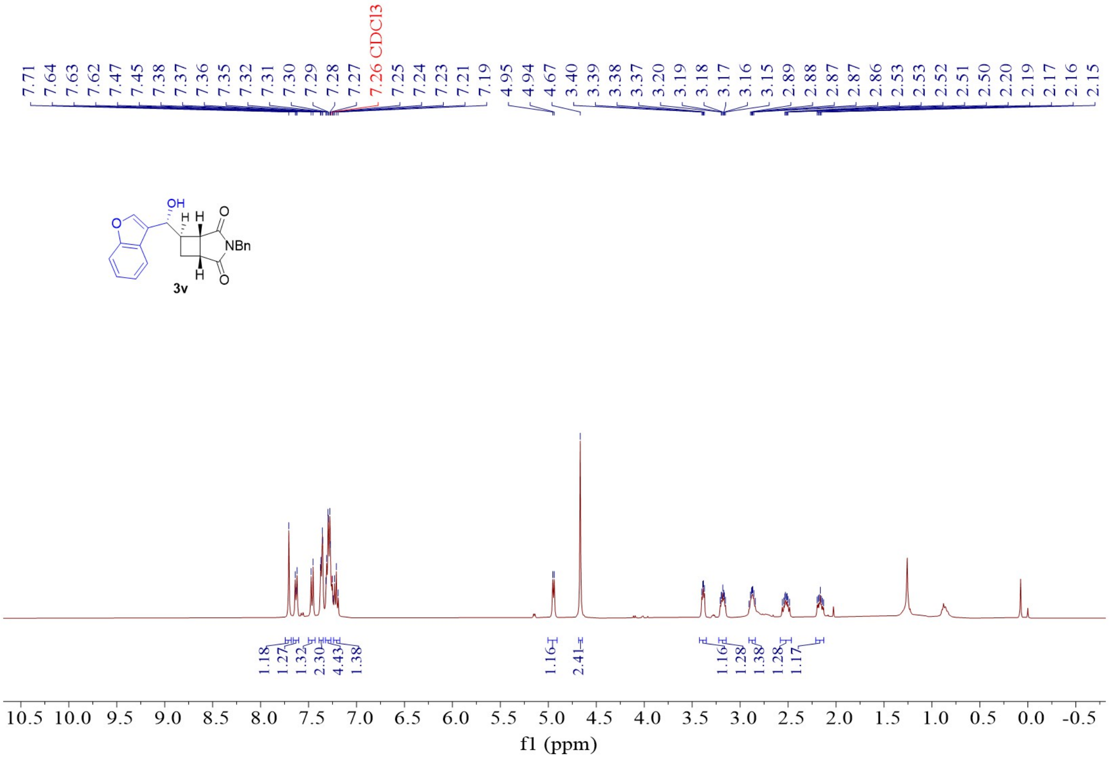
7.71
7.64
7.63
7.62
7.47
7.45
7.38
7.37
7.36
7.35
7.32
7.31
7.30
7.29
7.28
7.27
7.26 CDCl3
7.25
7.24
7.23
7.21
7.19
4.95
4.94
4.67
3.40
3.39
3.38
3.37
3.20
3.19
3.18
3.17
3.16
3.15
2.89
2.88
2.87
2.87
2.86
2.53
2.53
2.52
2.51
2.50
2.20
2.19
2.17
2.16
2.15
OH
H
H
O
NBn
O
3v
1.18
1.27
1.32
2.30
4.43
1.38
1.16
2.41
1.16
1.28
1.38
1.28
1.17
10.5 10.0 9.5 9.0 8.5 8.0 7.5 7.0 6.5 6.0 5.5 5.0 4.5 4.0 3.5 3.0 2.5 2.0 1.5 1.0 0.5 0.0 -0.5
f1 (ppm)

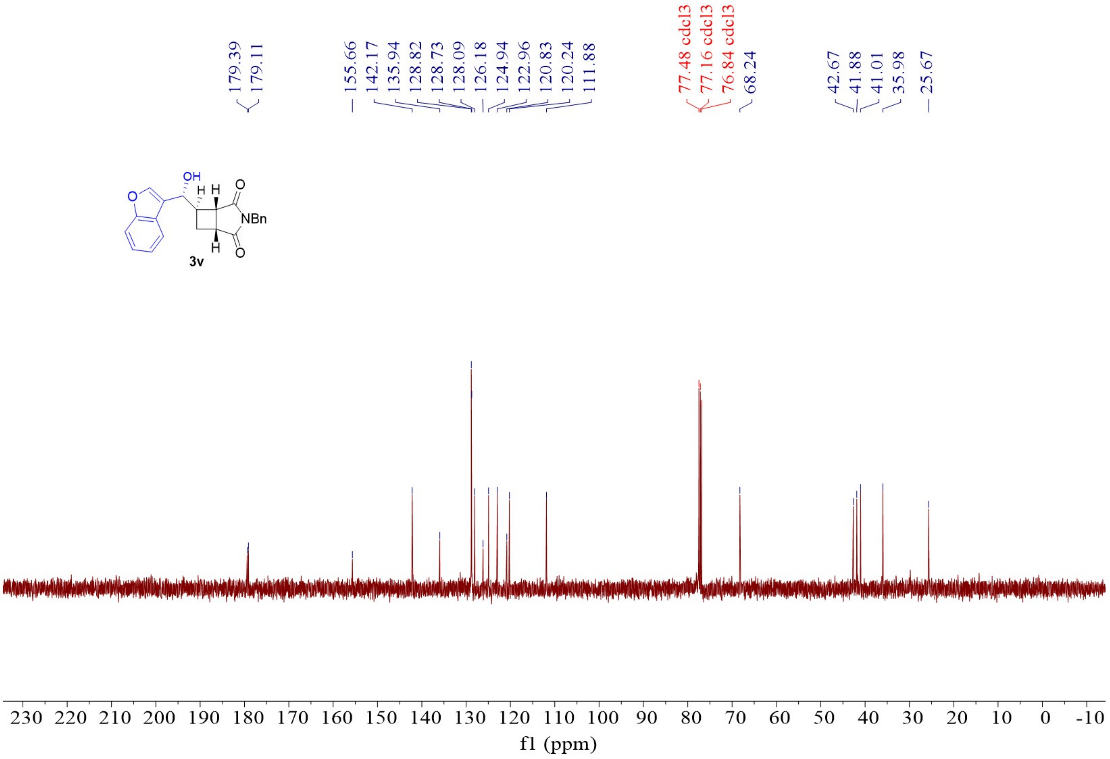

179.39
179.11
155.66
142.17
135.94
128.82
128.73
128.09
126.18
124.94
122.96
120.83
120.24
111.88
77.48 cdcl3
77.16 cdcl3
76.84 cdcl3
68.24
42.67
41.88
41.01
35.98
25.67
OH
H
H
O
NBn
O
3v
230 220 210 200 190 180 170 160 150 140 130 120 110 100 90 80 70 60 50 40 30 20 10 0 -10
f1 (ppm)

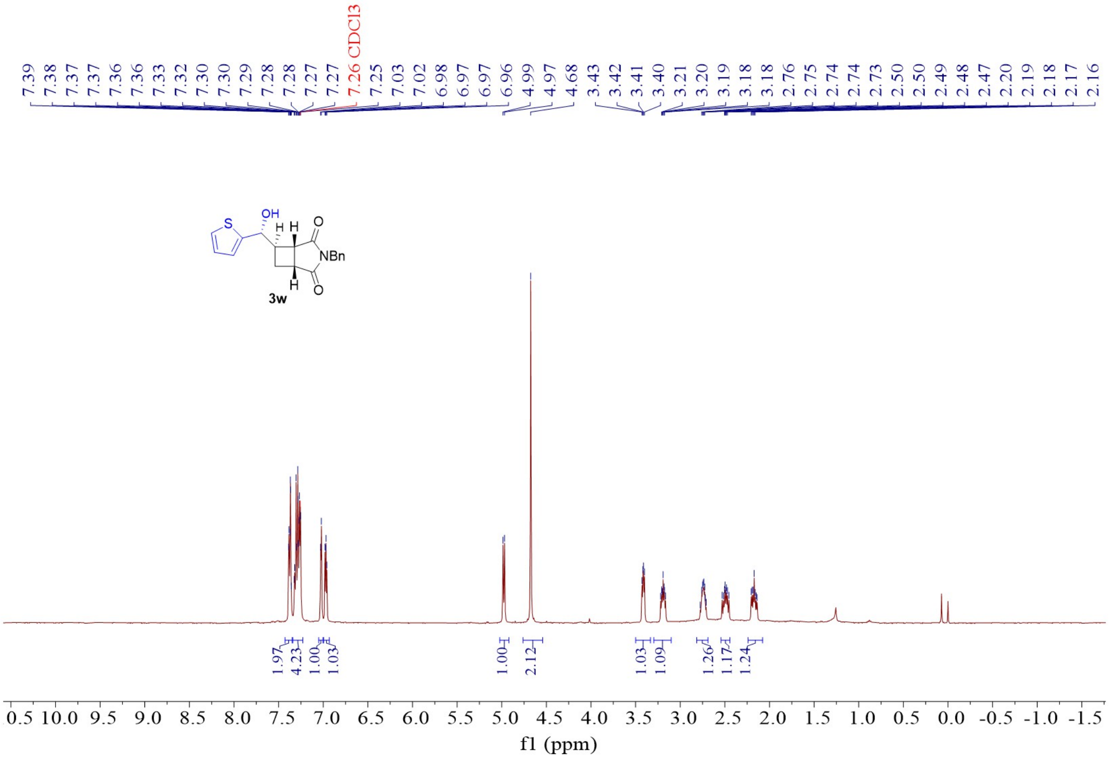

7.39
7.38
7.37
7.37
7.36
7.36
7.33
7.32
7.30
7.30
7.29
7.28
7.28
7.27
7.27
7.26 CDCl3
7.25
7.03
7.02
6.98
6.97
6.97
6.96
4.99
4.97
4.68
3.43
3.42
3.41
3.40
3.21
3.20
3.19
3.18
2.76
2.75
2.74
2.74
2.73
2.50
2.50
2.49
2.48
2.47
2.20
2.19
2.18
2.17
2.16
S
OH
H
H
O
NBn
O
3w
1.97
4.23
1.00
1.03
1.00
2.12
1.03
1.09
1.26
1.17
1.24
0.5 10.0 9.5 9.0 8.5 8.0 7.5 7.0 6.5 6.0 5.5 5.0 4.5 4.0 3.5 3.0 2.5 2.0 1.5 1.0 0.5 0.0 -0.5 -1.0 -1.5
f1 (ppm)

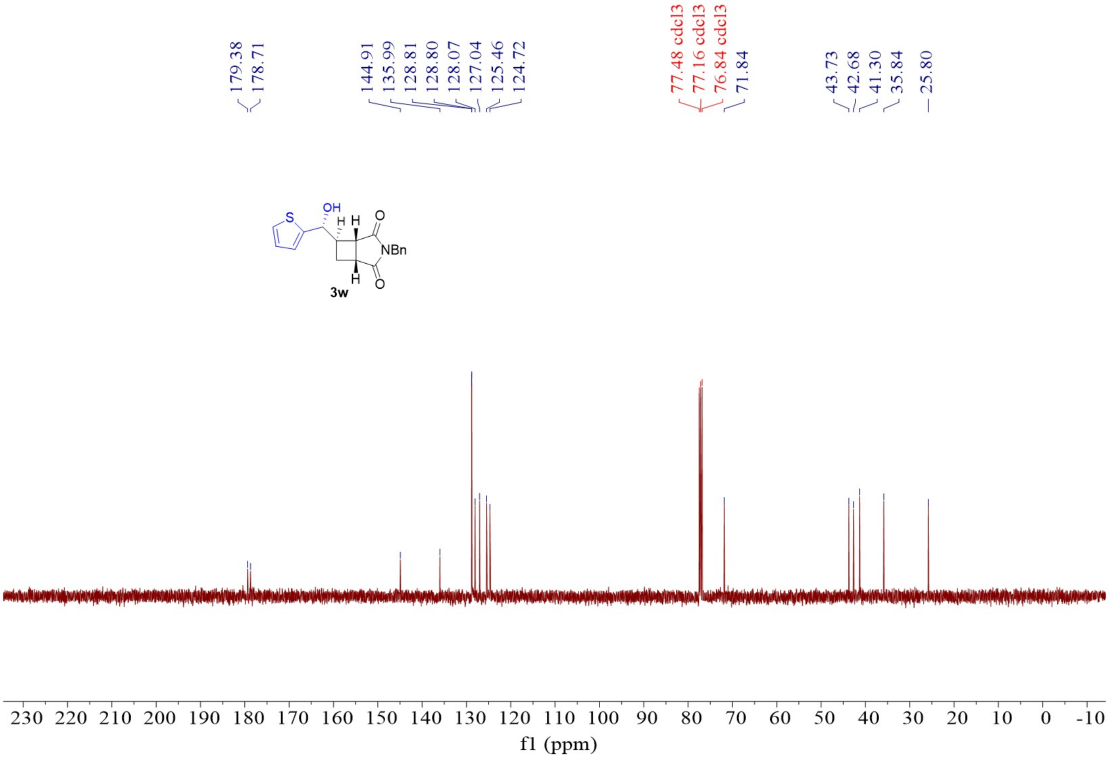
179.38
178.71
144.91
135.99
128.81
128.80
128.07
127.04
125.46
124.72
77.48 cdcl3
77.16 cdcl3
76.84 cdcl3
71.84
43.73
42.68
41.30
35.84
25.80
OH
S
H
H
O
NBn
H
O
3w
230 220 210 200 190 180 170 160 150 140 130 120 110 100 90 80 70 60 50 40 30 20 10 0 -10
f1 (ppm)

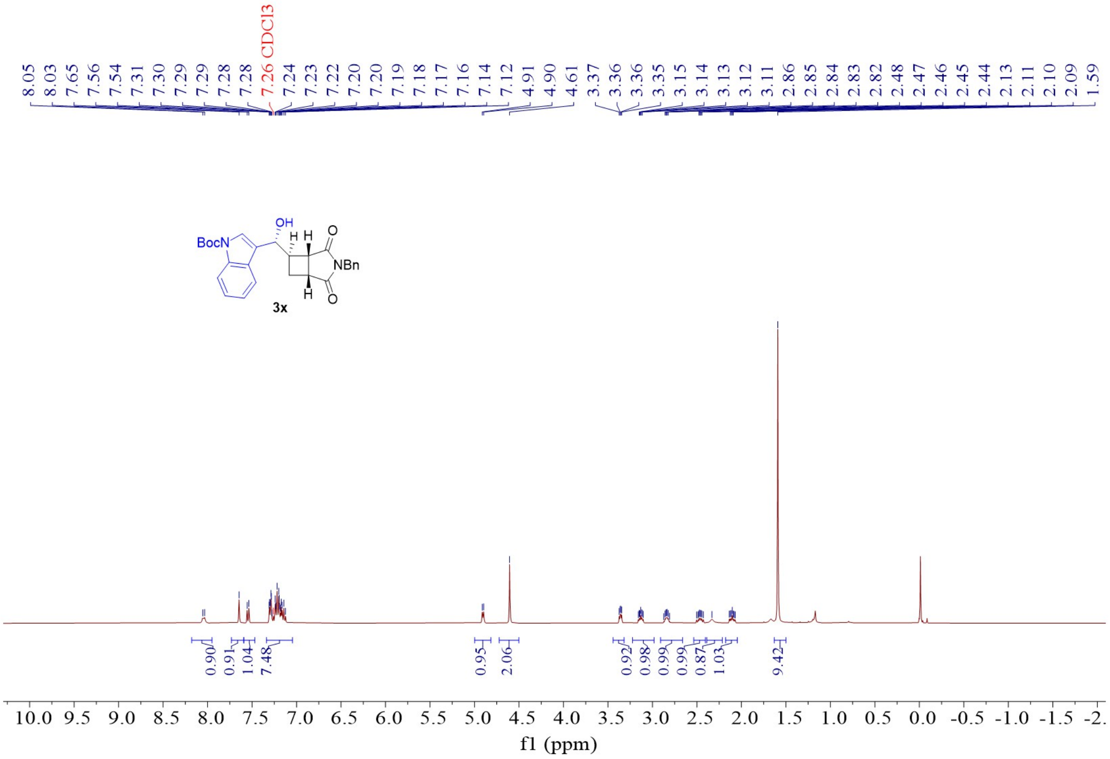
8.05
8.03
7.65
7.56
7.54
7.31
7.30
7.29
7.29
7.28
7.28
7.26 CDCl3
7.24
7.23
7.22
7.20
7.20
7.19
7.18
7.17
7.16
7.14
7.12
4.91
4.90
4.61
3.37
3.36
3.36
3.35
3.15
3.14
3.13
3.12
3.11
2.86
2.85
2.84
2.83
2.82
2.48
2.47
2.46
2.45
2.44
2.13
2.11
2.10
2.09
1.59
OH
BocN
H
H
O
NBn
H
O
3x
0.90
0.91
1.04
7.48
0.95
2.06
0.92
0.98
0.99
0.99
0.87
1.03
9.42
10.0 9.5 9.0 8.5 8.0 7.5 7.0 6.5 6.0 5.5 5.0 4.5 4.0 3.5 3.0 2.5 2.0 1.5 1.0 0.5 0.0 -0.5 -1.0 -1.5 -2.
f1 (ppm)

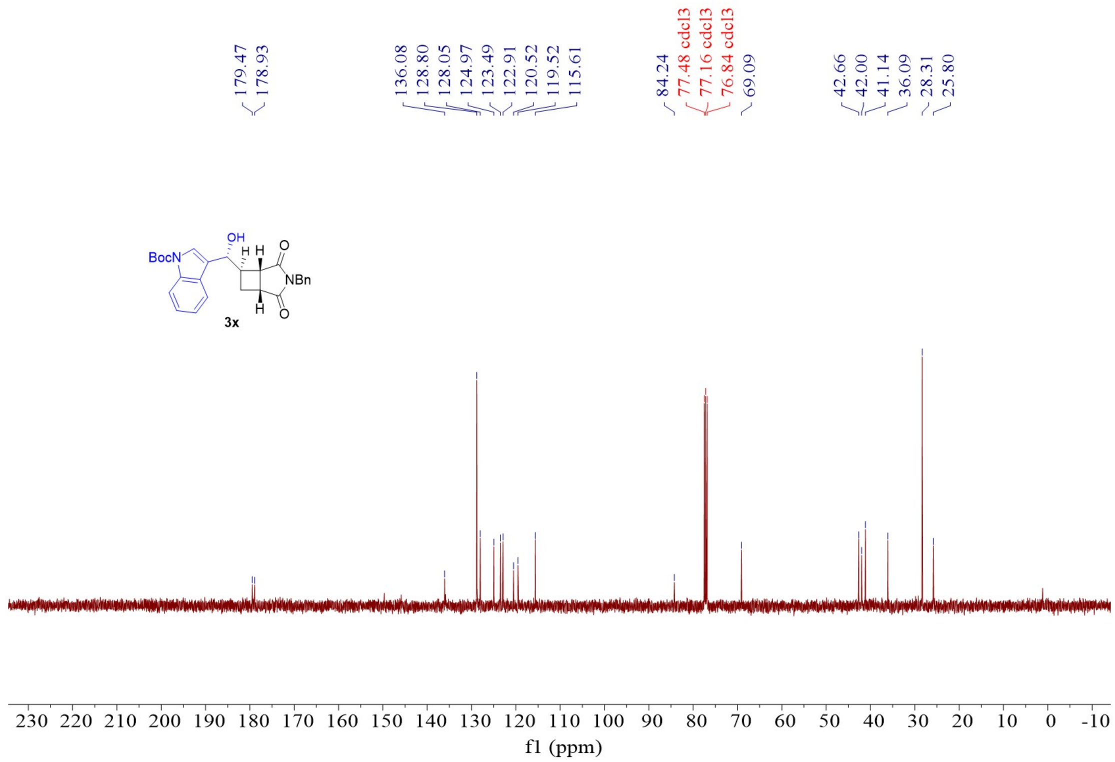
179.47
178.93
136.08
128.80
128.05
124.97
123.49
122.91
120.52
119.52
115.61
84.24
77.48 cdcl3
77.16 cdcl3
76.84 cdcl3
69.09
42.66
42.00
41.14
36.09
28.31
25.80
BocN
OH
H
H
O
NBn
H
O
3x
230 220 210 200 190 180 170 160 150 140 130 120 110 100 90 80 70 60 50 40 30 20 10 0 -10
f1 (ppm)

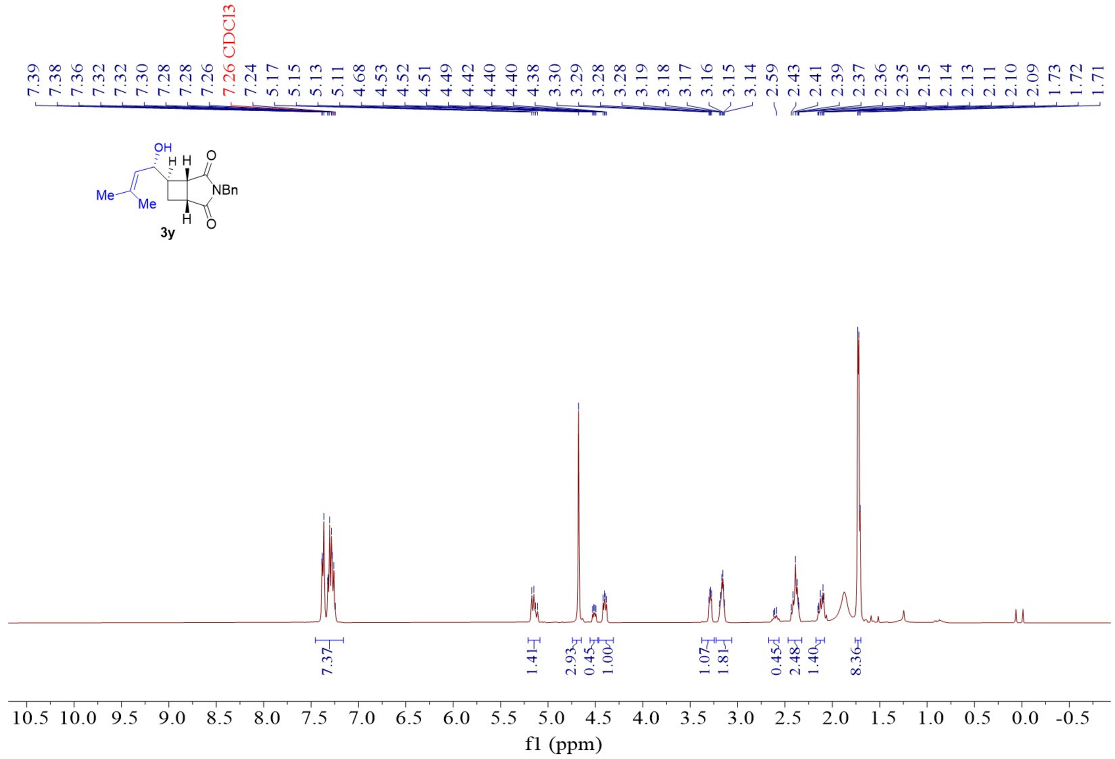
7.39
7.38
7.36
7.32
7.32
7.30
7.28
7.28
7.26
7.26 CDCl3
7.24
5.17
5.15
5.13
5.11
4.68
4.53
4.52
4.51
4.49
4.42
4.40
4.40
4.38
3.30
3.29
3.28
3.28
3.19
3.18
3.17
3.16
3.15
3.14
2.59
2.43
2.41
2.39
2.37
2.36
2.35
2.15
2.14
2.13
2.11
2.10
2.09
1.73
1.72
1.71
OH
H
H
O
Me
Me
NBn
H
O
3y
7.37
1.41
2.93
0.45
1.00
1.07
1.81
0.45
2.48
1.40
8.36
10.5 10.0 9.5 9.0 8.5 8.0 7.5 7.0 6.5 6.0 5.5 5.0 4.5 4.0 3.5 3.0 2.5 2.0 1.5 1.0 0.5 0.0 -0.5
f1 (ppm)

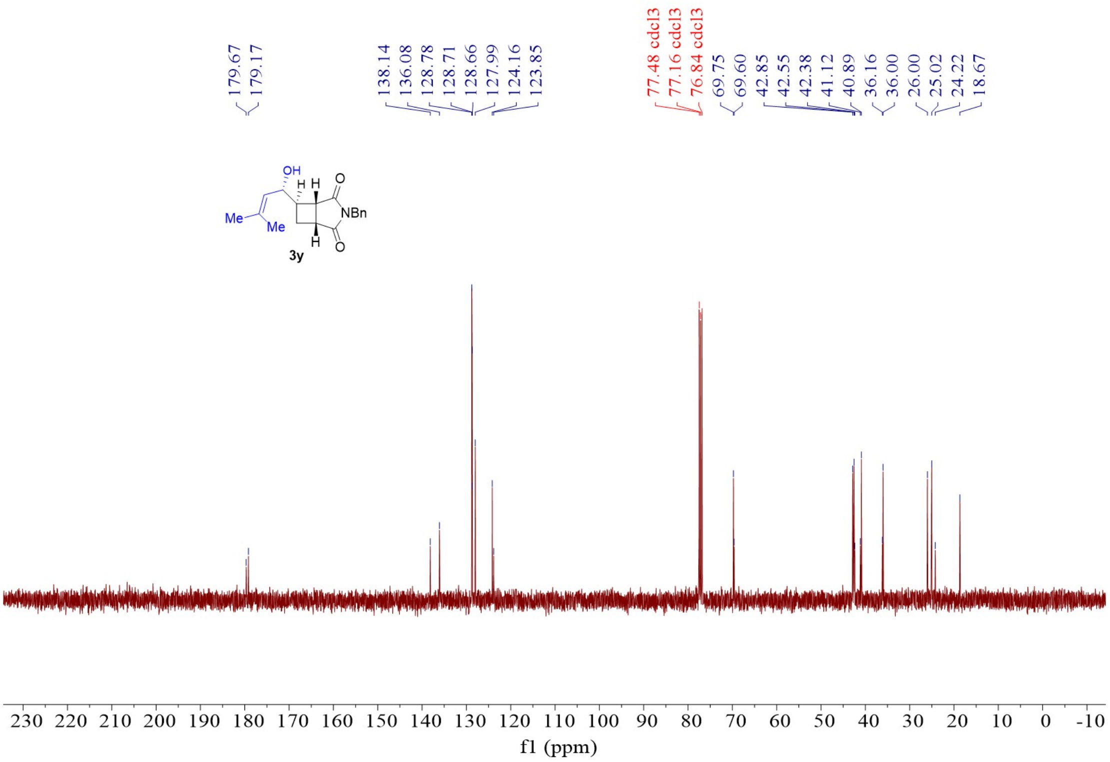
179.67
179.17
138.14
136.08
128.78
128.71
128.66
127.99
124.16
123.85
77.48 cdcl3
77.16 cdcl3
76.84 cdcl3
69.75
69.60
42.85
42.55
42.38
41.12
40.89
36.16
36.00
26.00
25.02
24.22
18.67
OH
H
H
O
NBn
O
Me
Me
3y
230 220 210 200 190 180 170 160 150 140 130 120 110 100 90 80 70 60 50 40 30 20 10 0 -10
f1 (ppm)

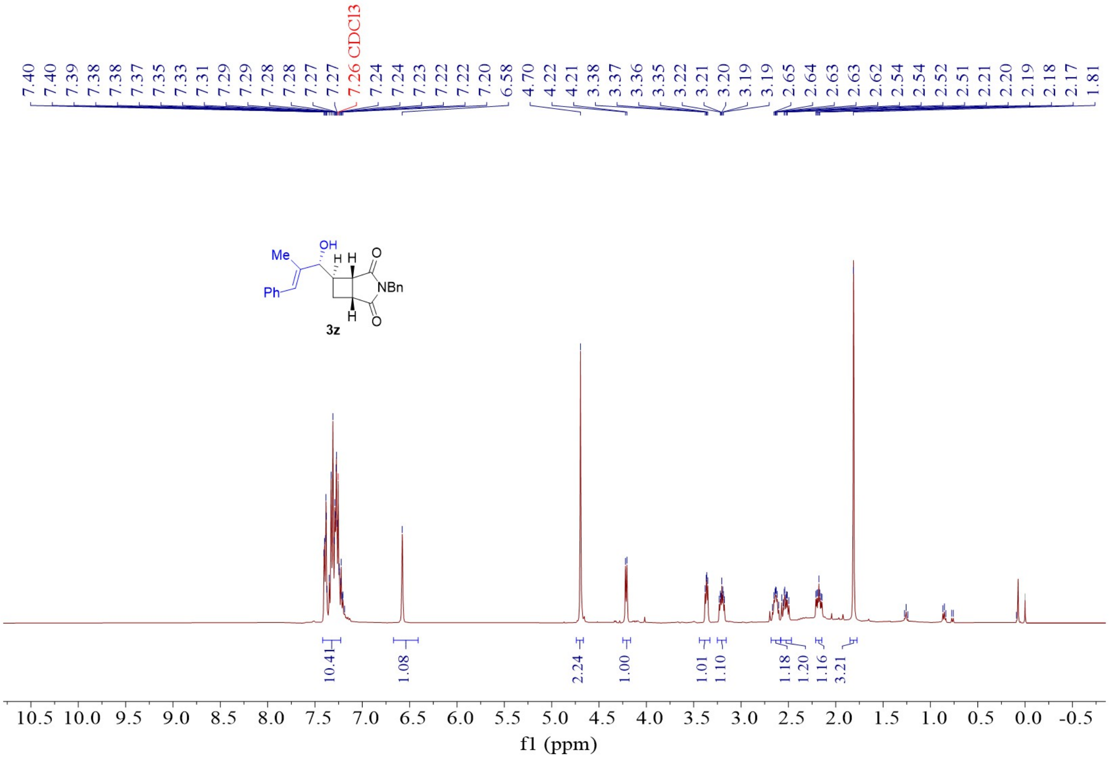
7.40
7.40
7.39
7.38
7.38
7.37
7.35
7.33
7.31
7.29
7.29
7.28
7.28
7.27
7.26 CDCl3
7.24
7.24
7.23
7.22
7.22
7.20
6.58
4.70
4.22
4.21
3.38
3.37
3.36
3.35
3.22
3.21
3.20
3.19
2.65
2.64
2.63
2.63
2.62
2.54
2.54
2.52
2.51
2.21
2.20
2.19
2.18
2.17
1.81
Me
OH
H
H
O
NBn
Ph
H
O
3z
10.41
1.08
2.24
1.00
1.01
1.10
1.18
1.20
1.16
3.21
10.5 10.0 9.5 9.0 8.5 8.0 7.5 7.0 6.5 6.0 5.5 5.0 4.5 4.0 3.5 3.0 2.5 2.0 1.5 1.0 0.5 0.0 -0.5
f1 (ppm)

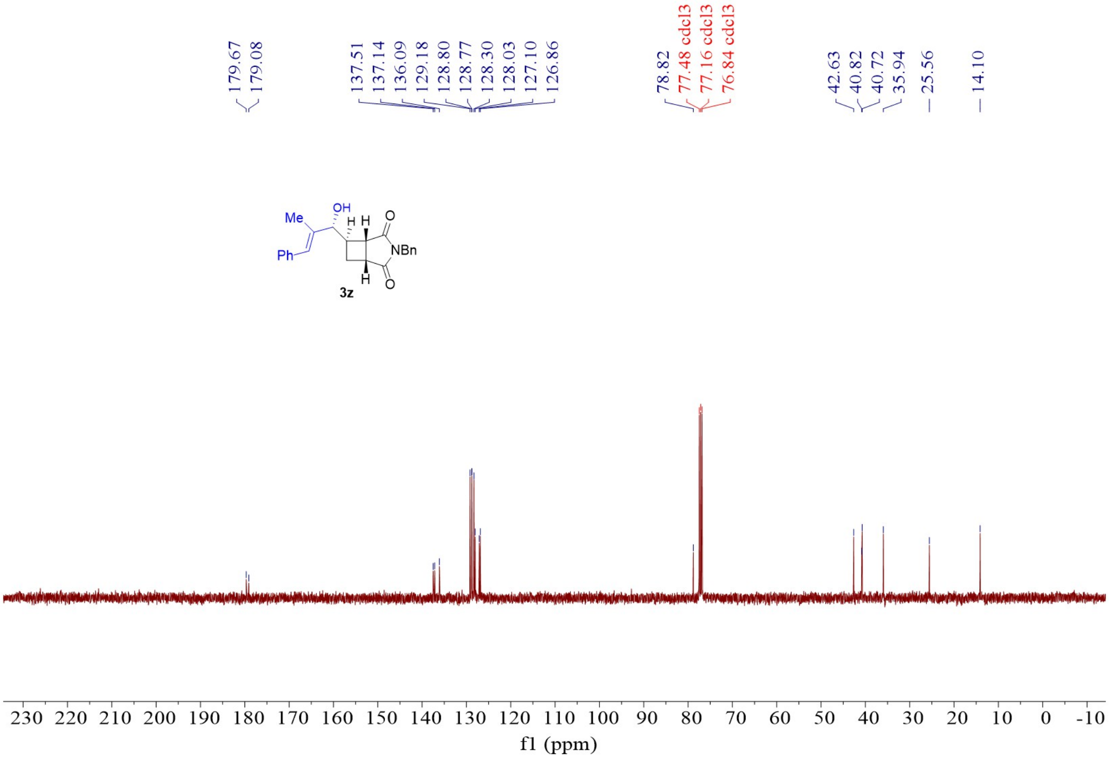
179.67
179.08
137.51
137.14
136.09
129.18
128.80
128.77
128.30
128.03
127.10
126.86
78.82
77.48 cdcl3
77.16 cdcl3
76.84 cdcl3
42.63
40.82
40.72
35.94
25.56
14.10
Me
OH
H H
O
NBn
Ph
H
O
3z
230 220 210 200 190 180 170 160 150 140 130 120 110 100 90 80 70 60 50 40 30 20 10 0 -10
f1 (ppm)

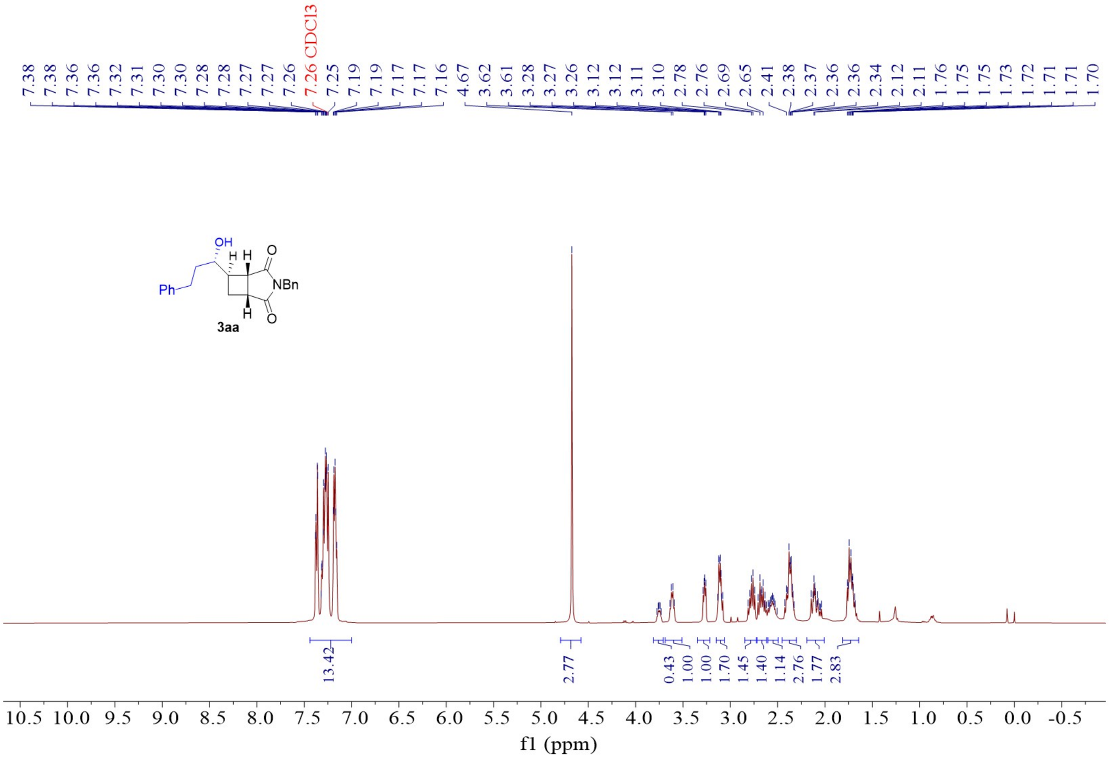
7.38 7.38 7.36 7.36 7.32 7.31 7.30 7.30 7.28 7.28 7.27 7.27 7.26 7.26 CDCl3 7.25 7.19 7.19 7.17 7.17 7.16 4.67 3.62 3.61 3.28 3.27 3.26 3.12 3.12 3.11 3.10 2.78 2.76 2.69 2.65 2.41 2.38 2.37 2.36 2.36 2.34 2.12 2.11 1.76 1.75 1.75 1.73 1.72 1.71 1.71 1.70
OH
H H
O
NBn
Ph
H
O
3aa
13.42
2.77
0.43
1.00
1.00
1.70
1.45
1.40
1.14
2.76
1.77
2.83
10.5 10.0 9.5 9.0 8.5 8.0 7.5 7.0 6.5 6.0 5.5 5.0 4.5 4.0 3.5 3.0 2.5 2.0 1.5 1.0 0.5 0.0 -0.5
f1 (ppm)

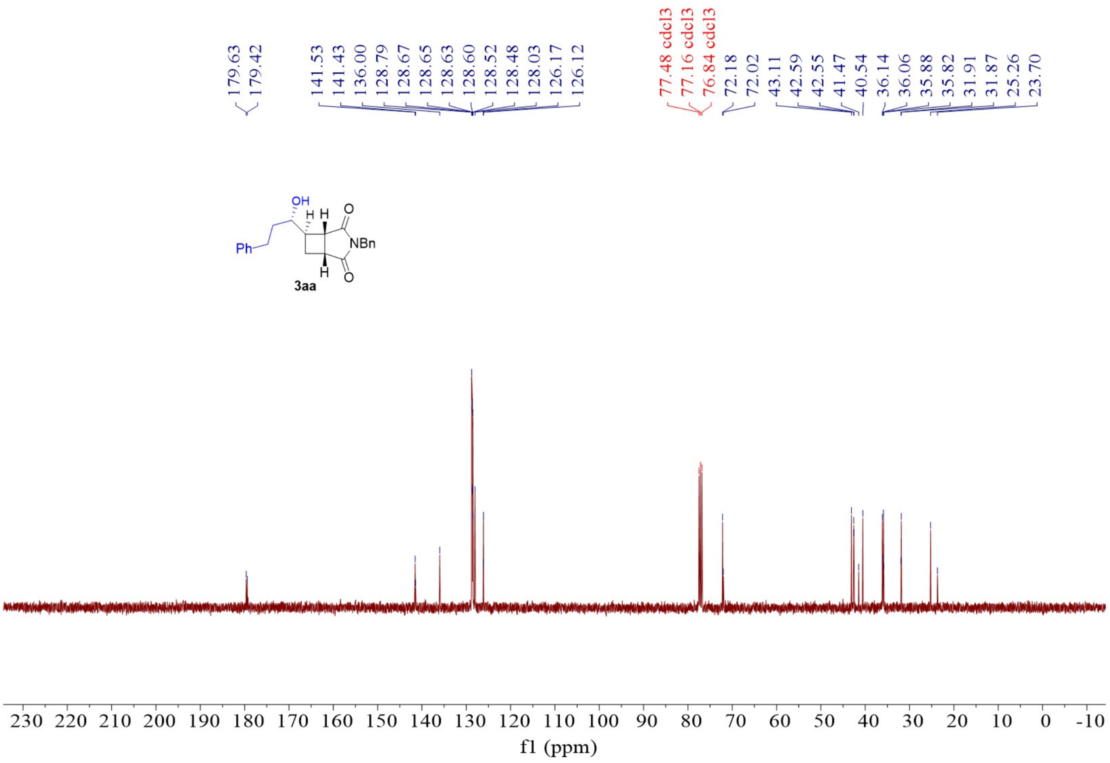
179.63
179.42
141.53
141.43
136.00
128.79
128.67
128.65
128.63
128.60
128.52
128.48
128.03
126.17
126.12
77.48 cdcl3
77.16 cdcl3
76.84 cdcl3
72.18
72.02
43.11
42.59
42.55
41.47
40.54
36.14
36.06
35.88
35.82
31.91
31.87
25.26
23.70
OH
H H
O
Ph
NBn
H
O
3aa
230 220 210 200 190 180 170 160 150 140 130 120 110 100 90 80 70 60 50 40 30 20 10 0 -10
f1 (ppm)

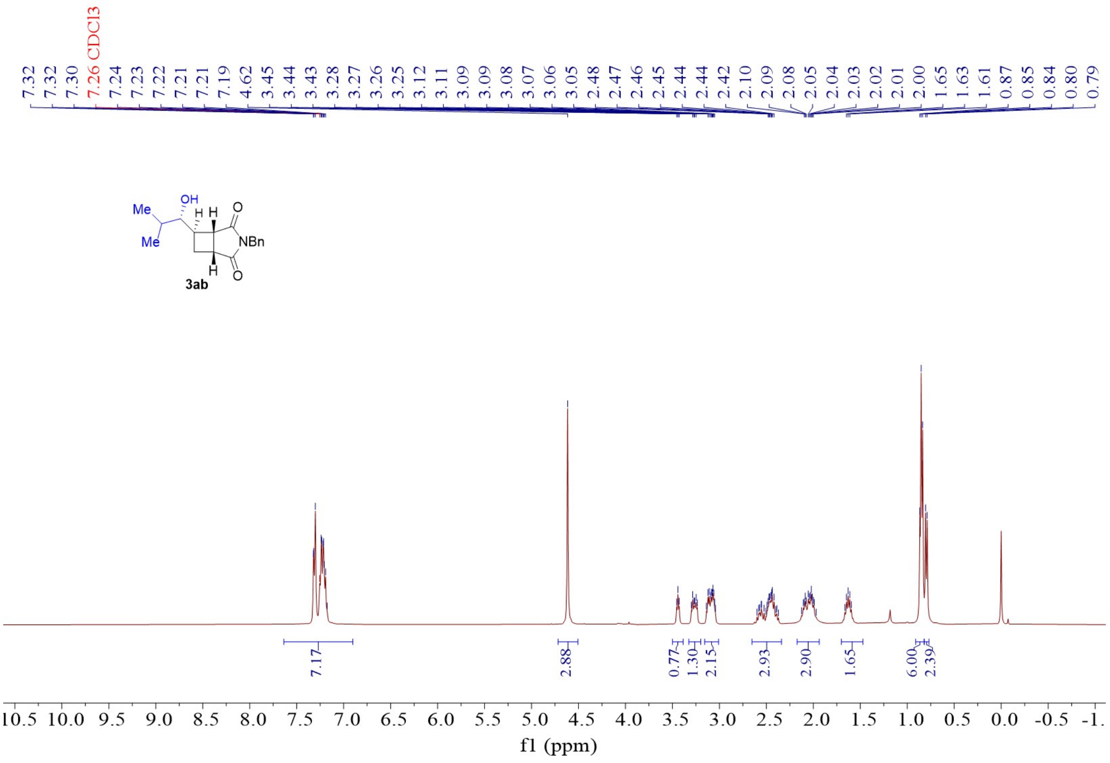
7.32
7.32
7.30
7.26 CDCl3
7.24
7.23
7.22
7.21
7.21
7.19
4.62
3.45
3.44
3.43
3.28
3.27
3.26
3.25
3.12
3.11
3.09
3.09
3.08
3.07
3.06
3.05
2.48
2.47
2.46
2.45
2.44
2.44
2.42
2.10
2.09
2.08
2.05
2.04
2.03
2.02
2.01
2.00
1.65
1.63
1.61
0.87
0.85
0.84
0.80
0.79
Me
OH
H H
O
Me
NBn
H
O
3ab
7.17
2.88
0.77
1.30
2.15
2.93
2.90
1.65
6.00
2.39
10.5 10.0 9.5 9.0 8.5 8.0 7.5 7.0 6.5 6.0 5.5 5.0 4.5 4.0 3.5 3.0 2.5 2.0 1.5 1.0 0.5 0.0 -0.5 -1.
f1 (ppm)

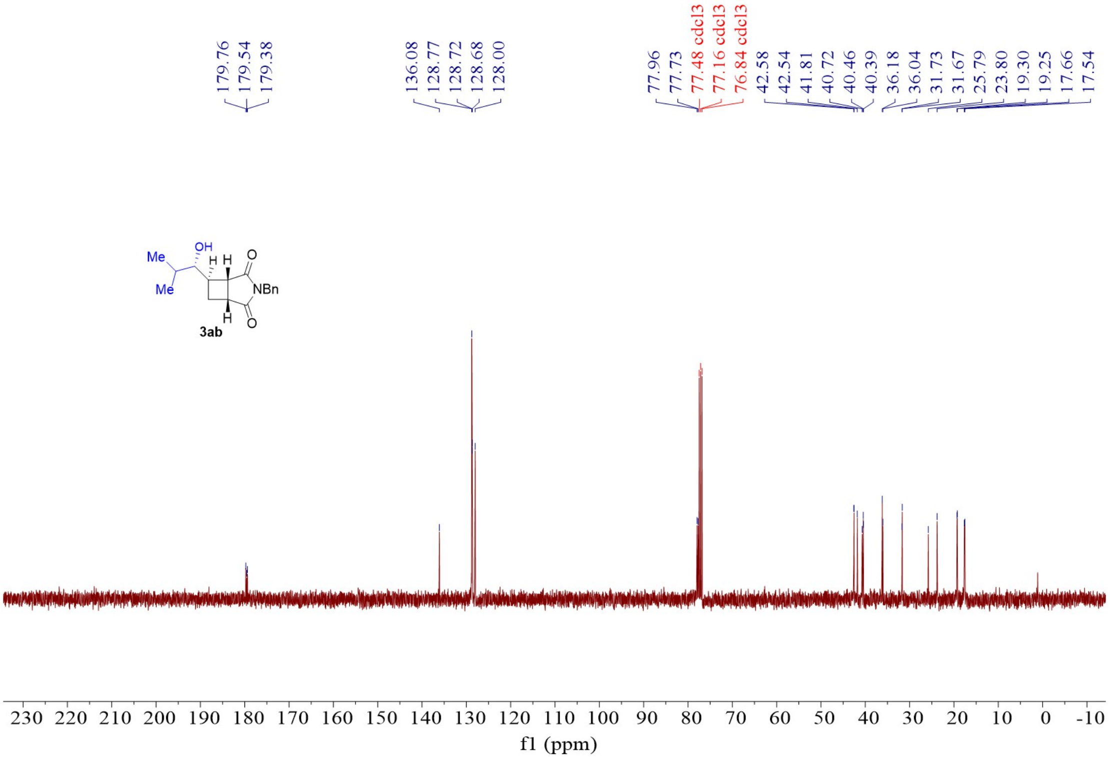
179.76
179.54
179.38
136.08
128.77
128.72
128.68
128.00
77.96
77.73
77.48 cdcl3
77.16 cdcl3
76.84 cdcl3
42.58
42.54
41.81
40.72
40.46
40.39
36.18
36.04
31.73
31.67
25.79
23.80
19.30
19.25
17.66
17.54
OH
Me
Me
H
H
O
NBn
H
O
3ab
230 220 210 200 190 180 170 160 150 140 130 120 110 100 90 80 70 60 50 40 30 20 10 0 -10
f1 (ppm)

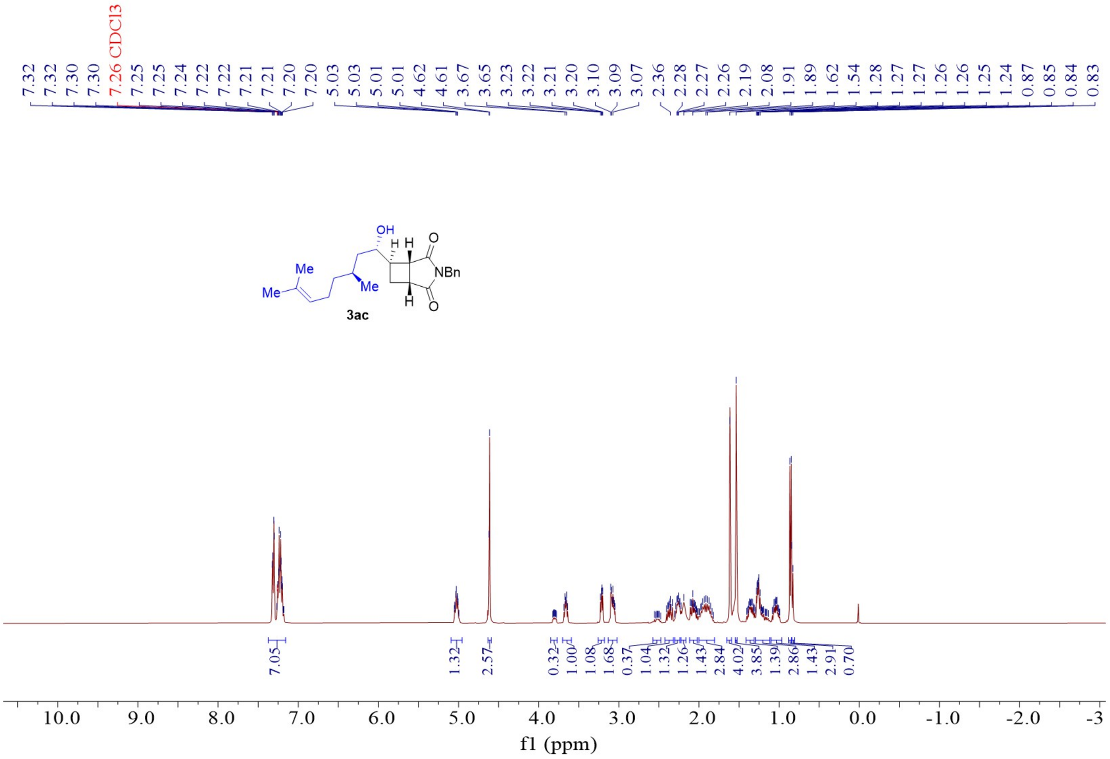
7.32
7.32
7.30
7.30
7.26 CDCl3
7.25
7.25
7.24
7.22
7.22
7.21
7.21
7.20
7.20
5.03
5.01
5.01
4.62
4.61
3.67
3.65
3.23
3.22
3.21
3.20
3.10
3.09
3.07
2.36
2.28
2.27
2.26
2.19
2.08
1.91
1.89
1.62
1.54
1.28
1.27
1.27
1.26
1.26
1.25
1.24
0.87
0.85
0.84
0.83
OH
Me
Me
H
H
O
NBn
Me
H
O
3ac
7.05
1.32
2.57
0.32
1.00
1.08
1.68
0.37
1.04
1.32
1.26
1.43
2.84
4.02
3.85
1.39
2.86
1.43
2.91
0.70
10.0 9.0 8.0 7.0 6.0 5.0 4.0 3.0 2.0 1.0 0.0 -1.0 -2.0 -3
f1 (ppm)

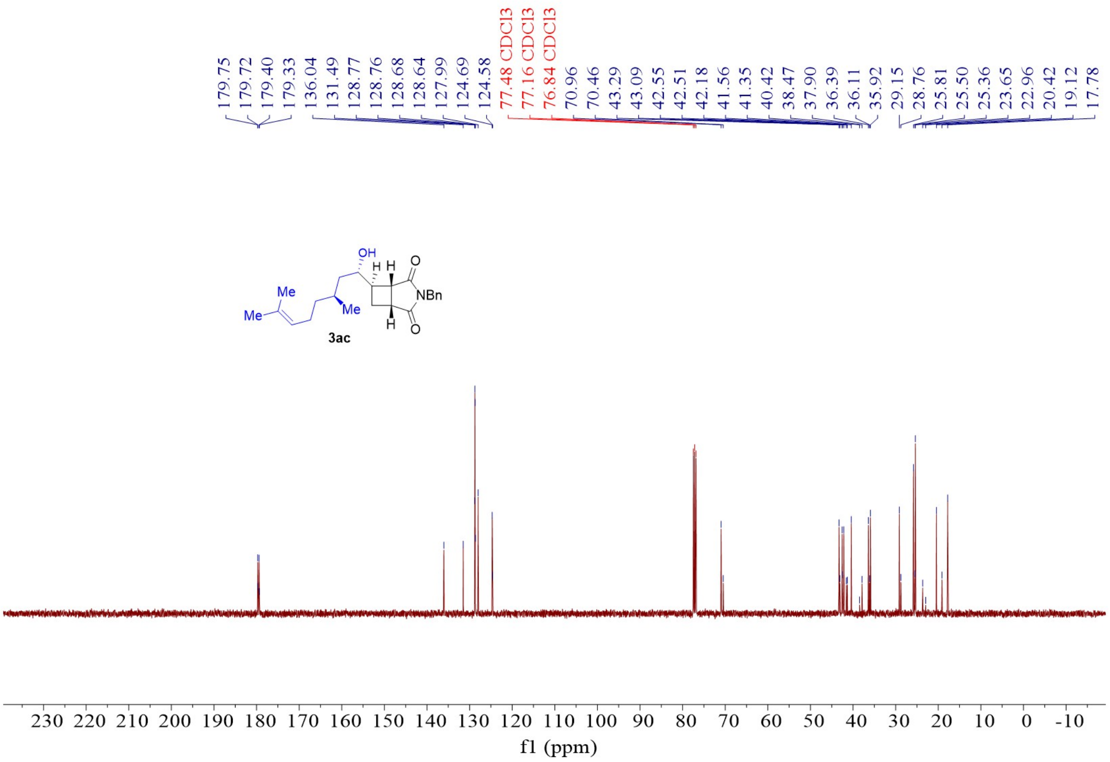
179.75
179.72
179.40
179.33
136.04
131.49
128.77
128.76
128.68
128.64
127.99
124.69
124.58
77.48 CDCl3
77.16 CDCl3
76.84 CDCl3
70.96
70.46
43.29
43.09
42.55
42.51
42.18
41.56
41.35
40.42
38.47
37.90
36.39
36.11
35.92
29.15
28.76
25.81
25.50
25.36
23.65
22.96
20.42
19.12
17.78
OH
H H
O
NBn
Me
Me
Me
H
O
3ac
230 220 210 200 190 180 170 160 150 140 130 120 110 100 90 80 70 60 50 40 30 20 10 0 -10
f1 (ppm)

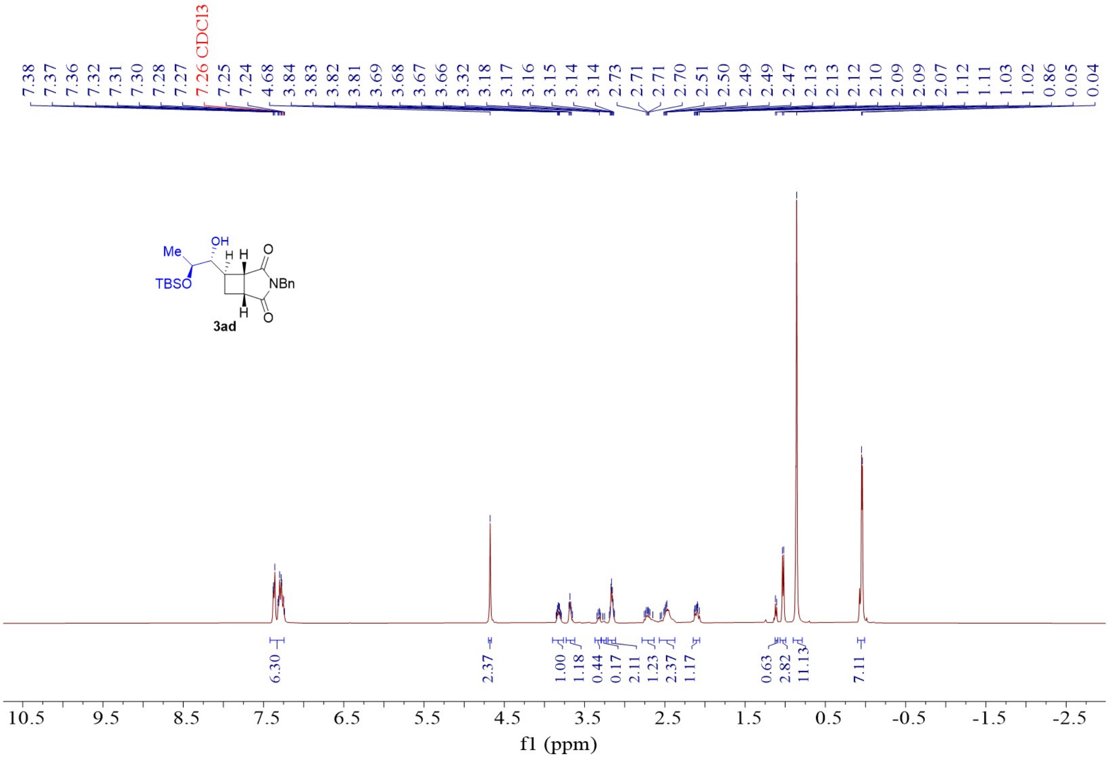
7.38
7.37
7.36
7.32
7.31
7.30
7.28
7.27
7.26 CDCl3
7.25
7.24
4.68
3.84
3.83
3.82
3.81
3.69
3.68
3.67
3.66
3.32
3.18
3.17
3.16
3.15
3.14
3.14
2.73
2.71
2.71
2.70
2.51
2.50
2.49
2.49
2.47
2.13
2.13
2.12
2.10
2.09
2.09
2.07
1.12
1.11
1.03
1.02
0.86
0.05
0.04
Me
OH
H H
O
TBSO
NBn
H
O
3ad
6.30
2.37
1.00
1.18
0.44
0.17
2.11
1.23
2.37
1.17
0.63
2.82
11.13
7.11
10.5 9.5 8.5 7.5 6.5 5.5 4.5 3.5 2.5 1.5 0.5 -0.5 -1.5 -2.5
f1 (ppm)

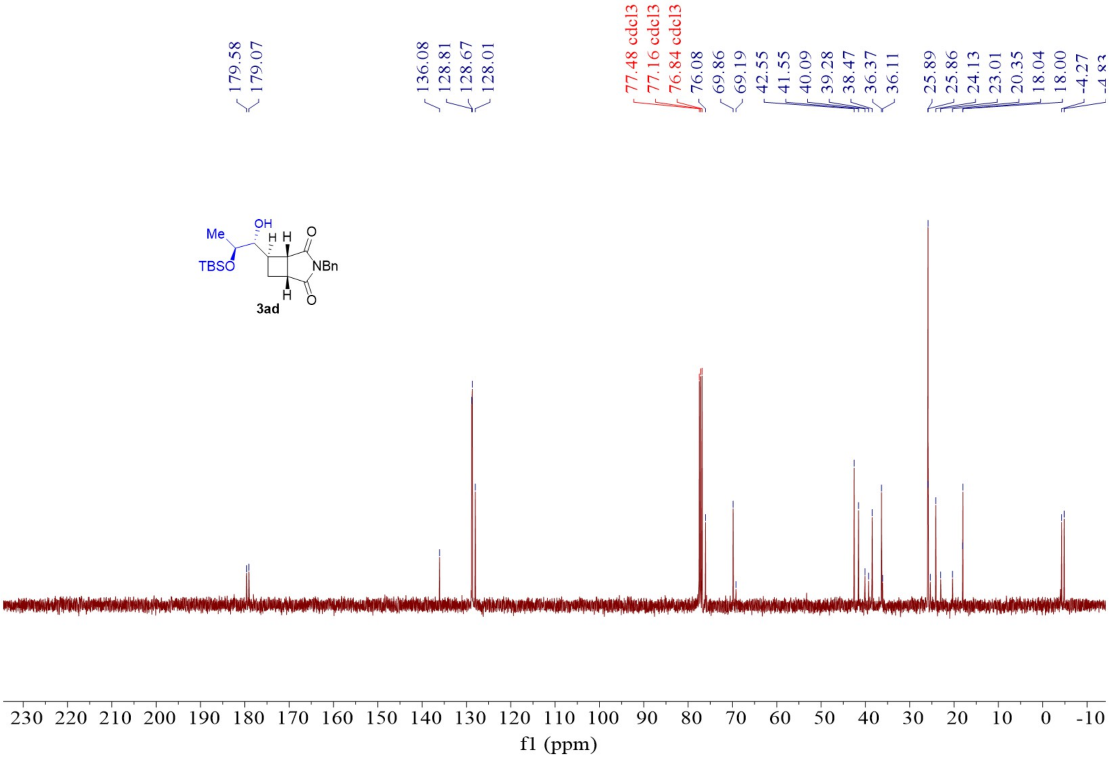
179.58
179.07
136.08
128.81
128.67
128.01
77.48 cdcl3
77.16 cdcl3
76.84 cdcl3
76.08
69.86
69.19
42.55
41.55
40.09
39.28
38.47
36.37
36.11
25.89
25.86
24.13
23.01
20.35
18.04
18.00
-4.27
Me
OH
H
H
O
TBSO
NBn
H
O
3ad
230 220 210 200 190 180 170 160 150 140 130 120 110 100 90 80 70 60 50 40 30 20 10 0 -10
f1 (ppm)

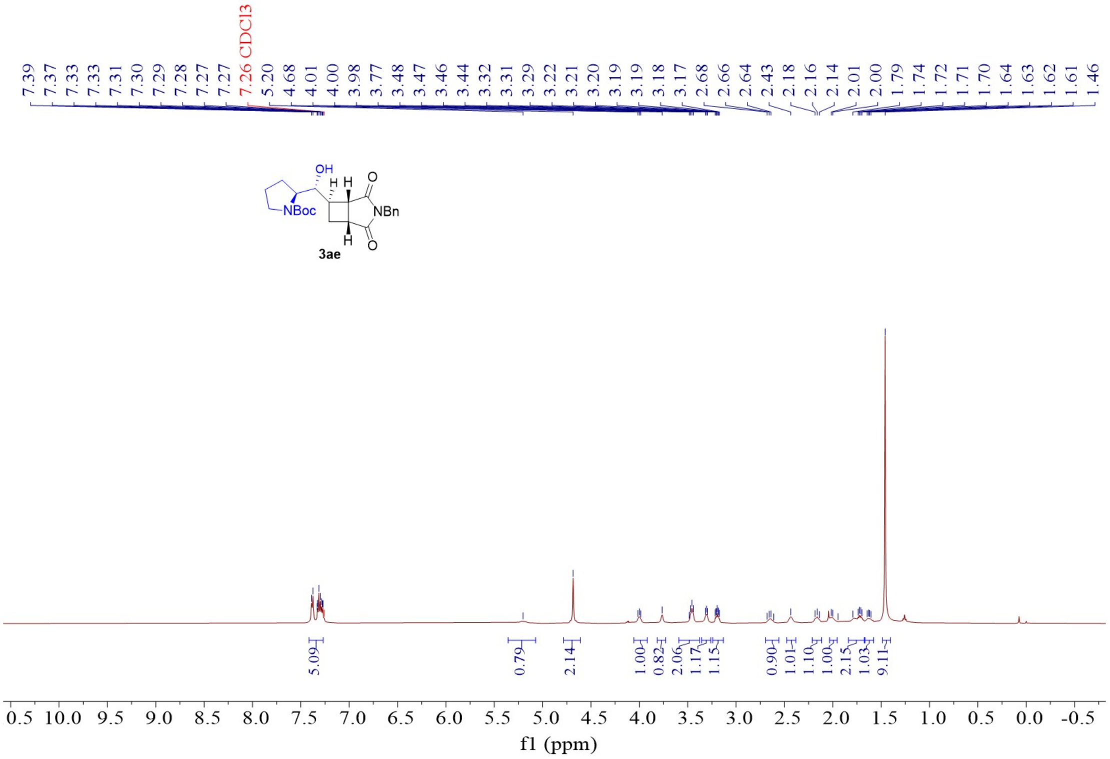
7.39
7.37
7.33
7.33
7.31
7.30
7.29
7.28
7.27
7.27
7.26 CDCl3
5.20
4.68
4.01
4.00
3.98
3.77
3.48
3.47
3.46
3.44
3.32
3.31
3.29
3.22
3.21
3.20
3.19
3.19
3.18
3.17
2.68
2.66
2.64
2.43
2.18
2.16
2.14
2.01
2.00
1.79
1.74
1.72
1.71
1.70
1.64
1.63
1.62
1.61
1.46
OH
H
H
O
NBoc
NBn
H
O
3ae
5.09
0.79
2.14
1.00
0.82
2.06
1.17
1.15
0.90
1.01
1.10
1.00
2.15
1.03
9.11
0.5 10.0 9.5 9.0 8.5 8.0 7.5 7.0 6.5 6.0 5.5 5.0 4.5 4.0 3.5 3.0 2.5 2.0 1.5 1.0 0.5 0.0 -0.5
f1 (ppm)

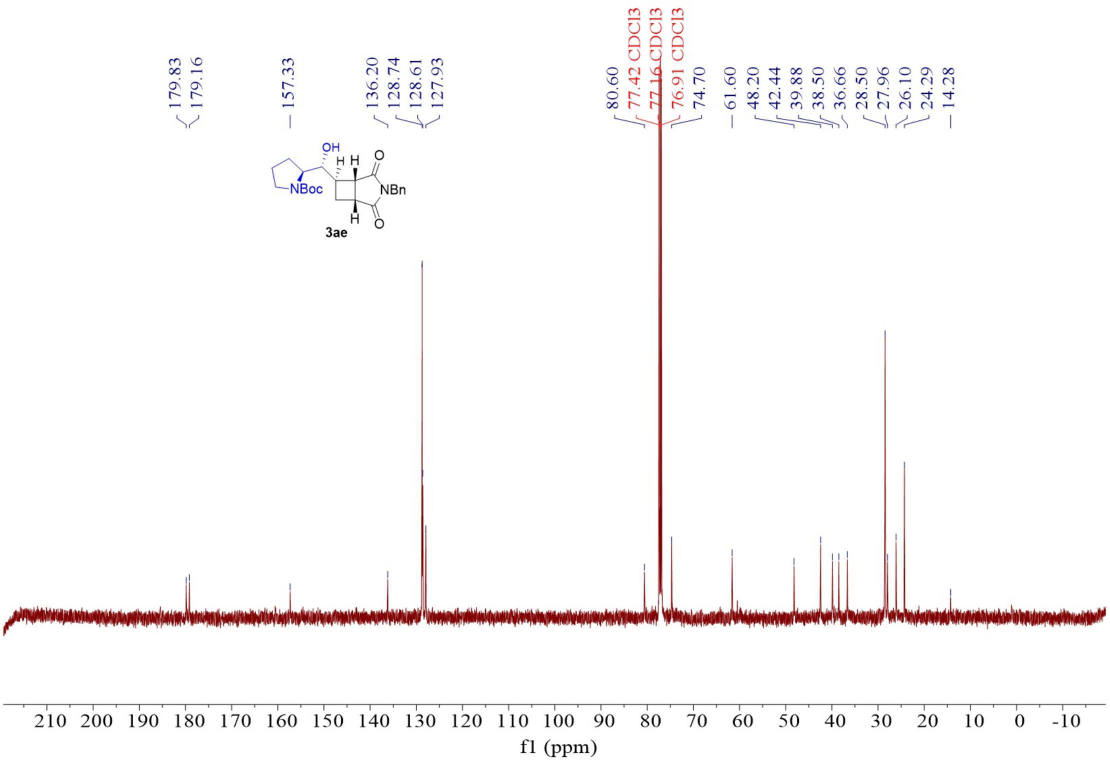
179.83
179.16
157.33
136.20
128.74
128.61
127.93
80.60
77.42 CDCl3
77.16 CDCl3
76.91 CDCl3
74.70
61.60
48.20
42.44
39.88
38.50
36.66
28.50
27.96
26.10
24.29
14.28
OH
H H
O
NBoc
NBn
H
O
3ae
210 200 190 180 170 160 150 140 130 120 110 100 90 80 70 60 50 40 30 20 10 0 -10
f1 (ppm)

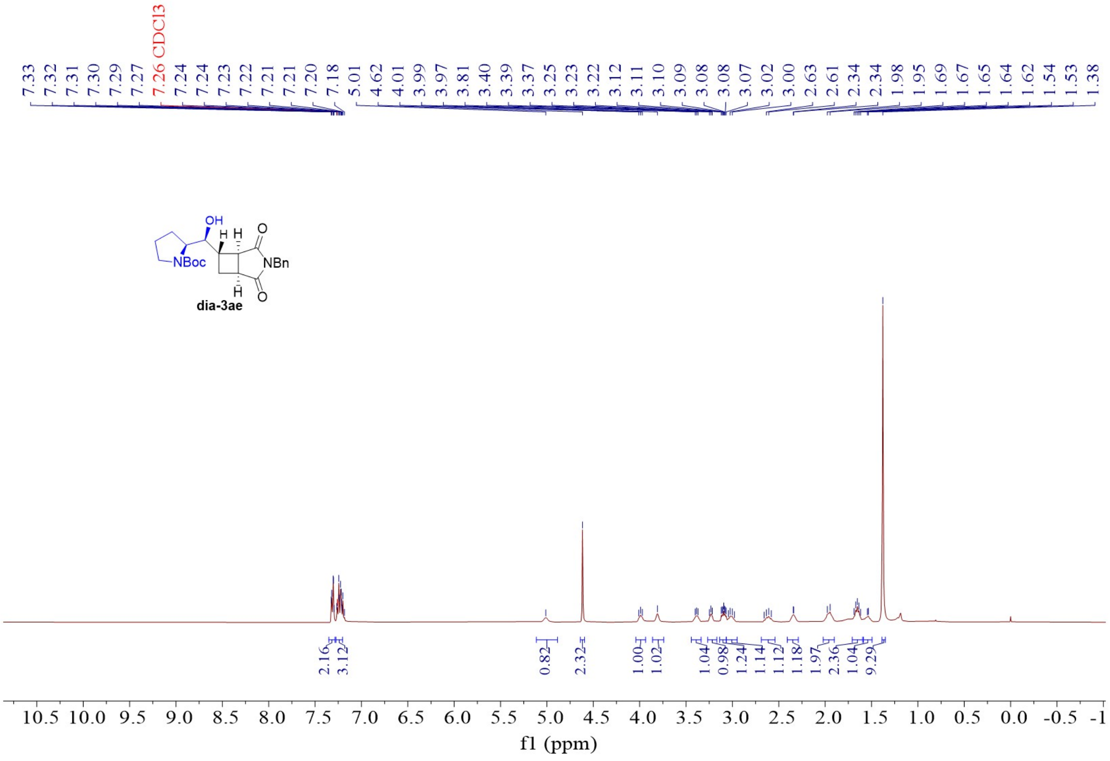
7.33
7.32
7.31
7.30
7.29
7.27
7.26 CDCl3
7.24
7.24
7.23
7.22
7.21
7.21
7.20
7.18
5.01
4.62
4.01
3.99
3.97
3.81
3.40
3.39
3.37
3.25
3.23
3.22
3.12
3.11
3.10
3.09
3.08
3.08
3.07
3.02
3.00
2.63
2.61
2.34
2.34
1.98
1.95
1.69
1.67
1.65
1.64
1.62
1.54
1.53
1.38
OH
H H
O
NBoc
NBn
H
O
dia-3ae
2.16
3.12
0.82
2.32
1.00
1.02
1.04
0.98
1.24
1.14
1.12
1.18
1.97
2.36
1.04
9.29
10.5 10.0 9.5 9.0 8.5 8.0 7.5 7.0 6.5 6.0 5.5 5.0 4.5 4.0 3.5 3.0 2.5 2.0 1.5 1.0 0.5 0.0 -0.5 -1
f1 (ppm)

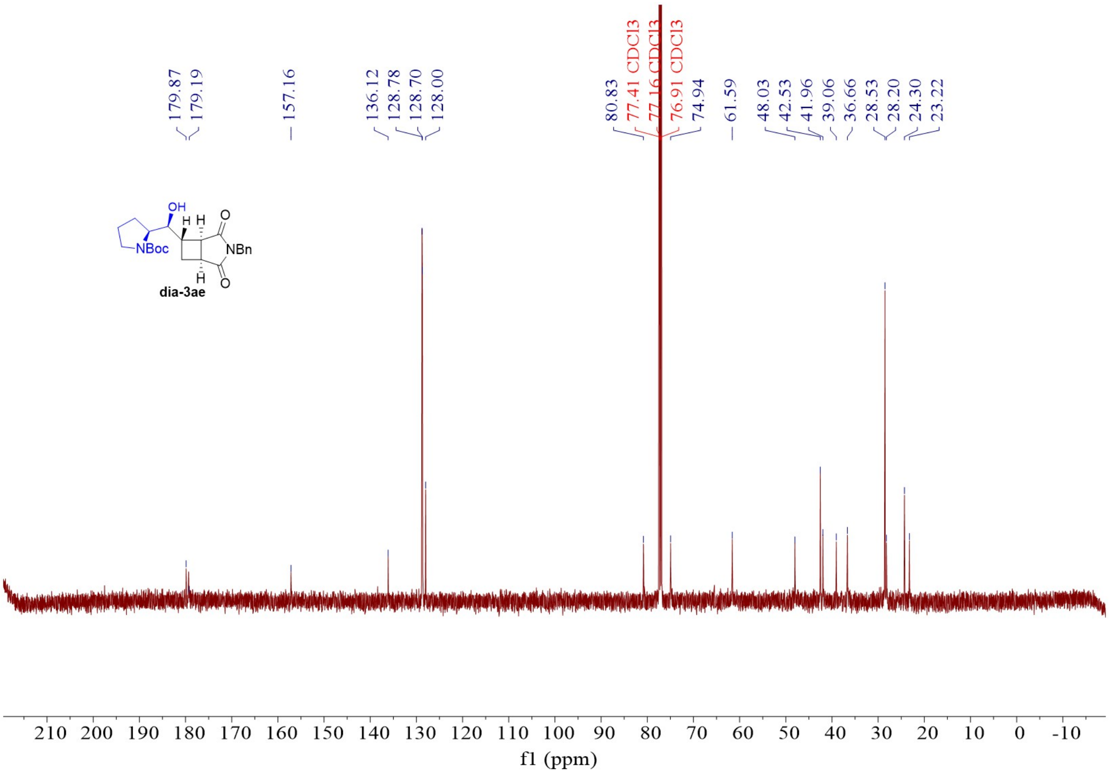
179.87
179.19
157.16
136.12
128.78
128.70
128.00
80.83
77.41 CDCl3
77.16 CDCl3
76.91 CDCl3
74.94
61.59
48.03
42.53
41.96
39.06
36.66
28.53
28.20
24.30
23.22
OH
H H
O
NBoc
NBn
H
O
dia-3ae
210 200 190 180 170 160 150 140 130 120 110 100 90 80 70 60 50 40 30 20 10 0 -10
f1 (ppm)

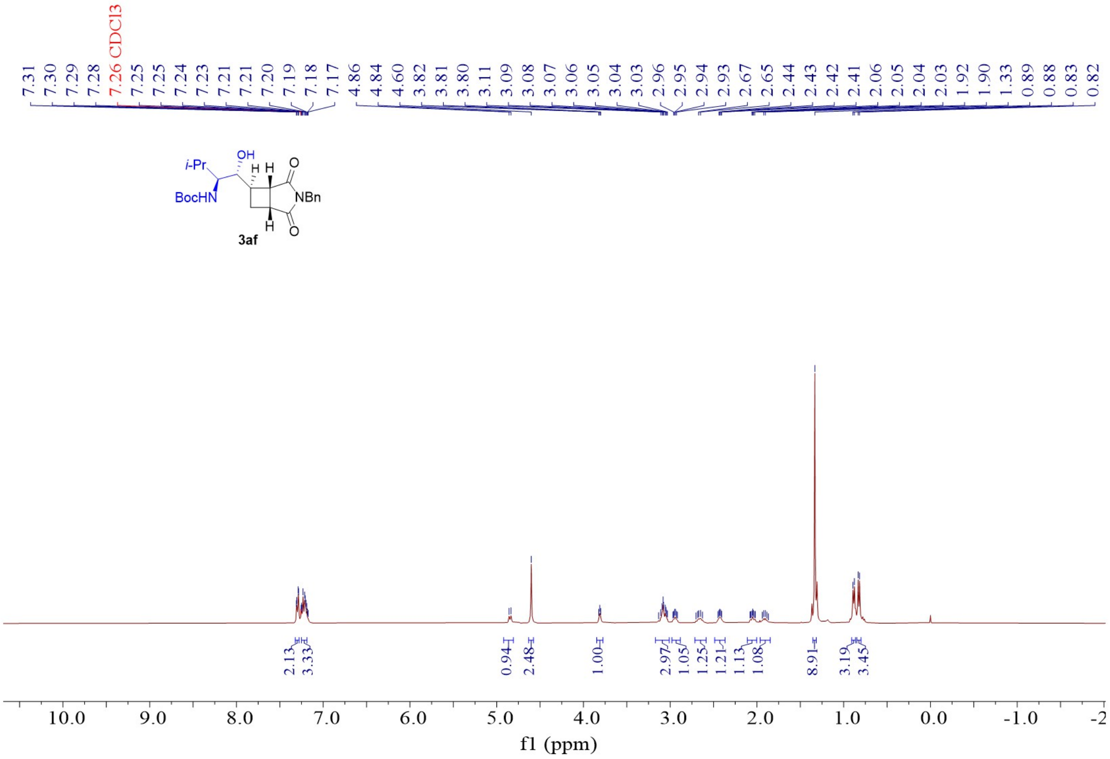
7.31
7.30
7.29
7.28
7.26 CDCl3
7.25
7.24
7.23
7.21
7.21
7.20
7.19
7.18
7.17
4.86
4.84
4.60
3.82
3.81
3.80
3.11
3.09
3.08
3.07
3.06
3.05
3.04
3.03
2.96
2.95
2.94
2.93
2.67
2.65
2.44
2.43
2.42
2.41
2.06
2.05
2.04
2.03
1.92
1.90
1.33
0.89
0.88
0.83
0.82
i-Pr
OH
H H
O
BocHN
NBn
H
O
3af
2.13
3.33
0.94
2.48
1.00
2.97
1.05
1.25
1.21
1.13
1.08
8.91
3.19
3.45
10.0 9.0 8.0 7.0 6.0 5.0 4.0 3.0 2.0 1.0 0.0 -1.0 -2
f1 (ppm)

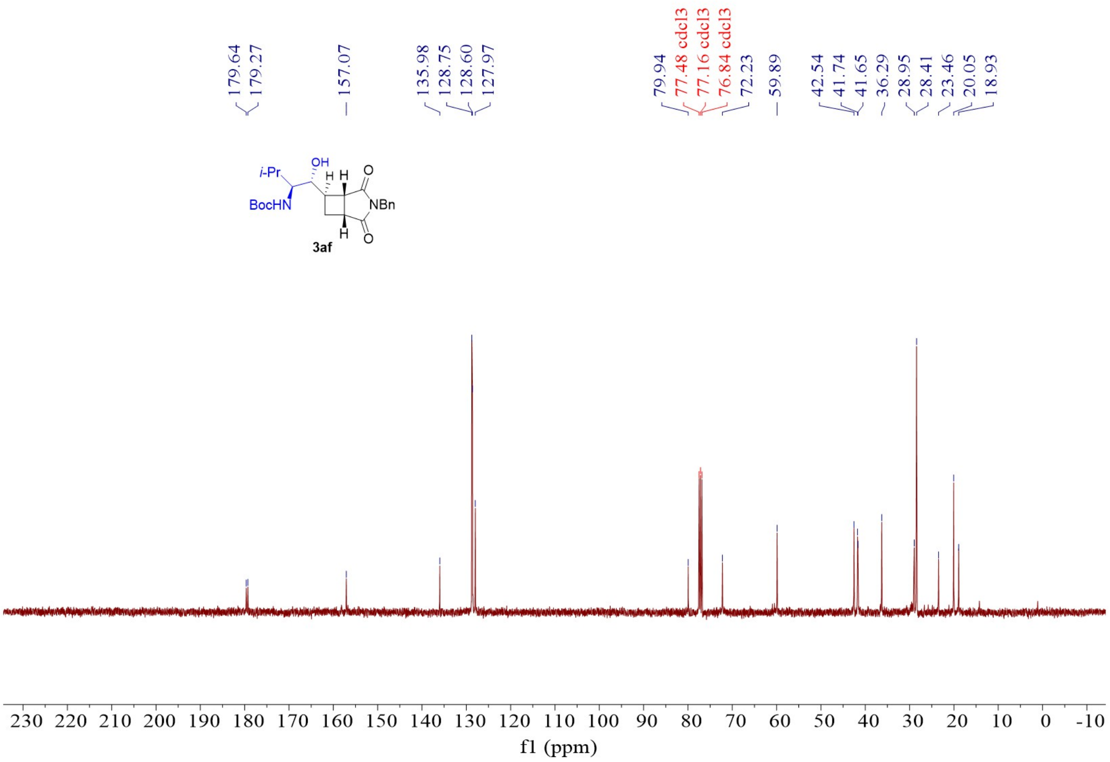
179.64
179.27
157.07
135.98
128.75
128.60
127.97
79.94
77.48 cdcl3
77.16 cdcl3
76.84 cdcl3
72.23
59.89
42.54
41.74
41.65
36.29
28.95
28.41
23.46
20.05
18.93
i-Pr
OH
H
H
O
BocHN
NBn
H
O
3af
230 220 210 200 190 180 170 160 150 140 130 120 110 100 90 80 70 60 50 40 30 20 10 0 -10
f1 (ppm)

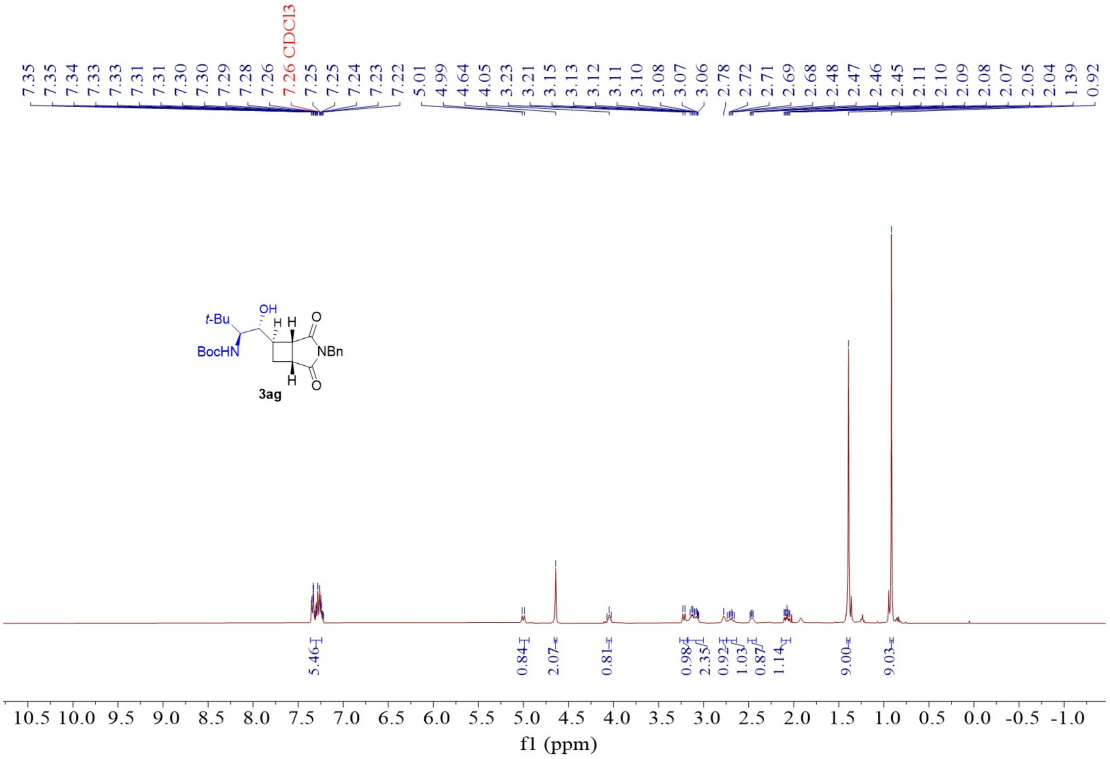
7.35
7.35
7.34
7.33
7.33
7.31
7.31
7.30
7.30
7.29
7.28
7.26
7.26 CDCl3
7.25
7.25
7.24
7.23
7.22
5.01
4.99
4.64
4.05
3.23
3.21
3.15
3.13
3.12
3.11
3.10
3.08
3.07
3.06
2.78
2.72
2.71
2.69
2.68
2.48
2.47
2.46
2.45
2.11
2.10
2.09
2.08
2.07
2.05
2.04
1.39
0.92
t-Bu
OH
H
H
O
BocHN
NBn
H
O
3ag
5.46
0.84
2.07
0.81
0.98
2.35
0.92
1.03
0.87
1.14
9.00
9.03
10.5 10.0 9.5 9.0 8.5 8.0 7.5 7.0 6.5 6.0 5.5 5.0 4.5 4.0 3.5 3.0 2.5 2.0 1.5 1.0 0.5 0.0 -0.5 -1.0
f1 (ppm)

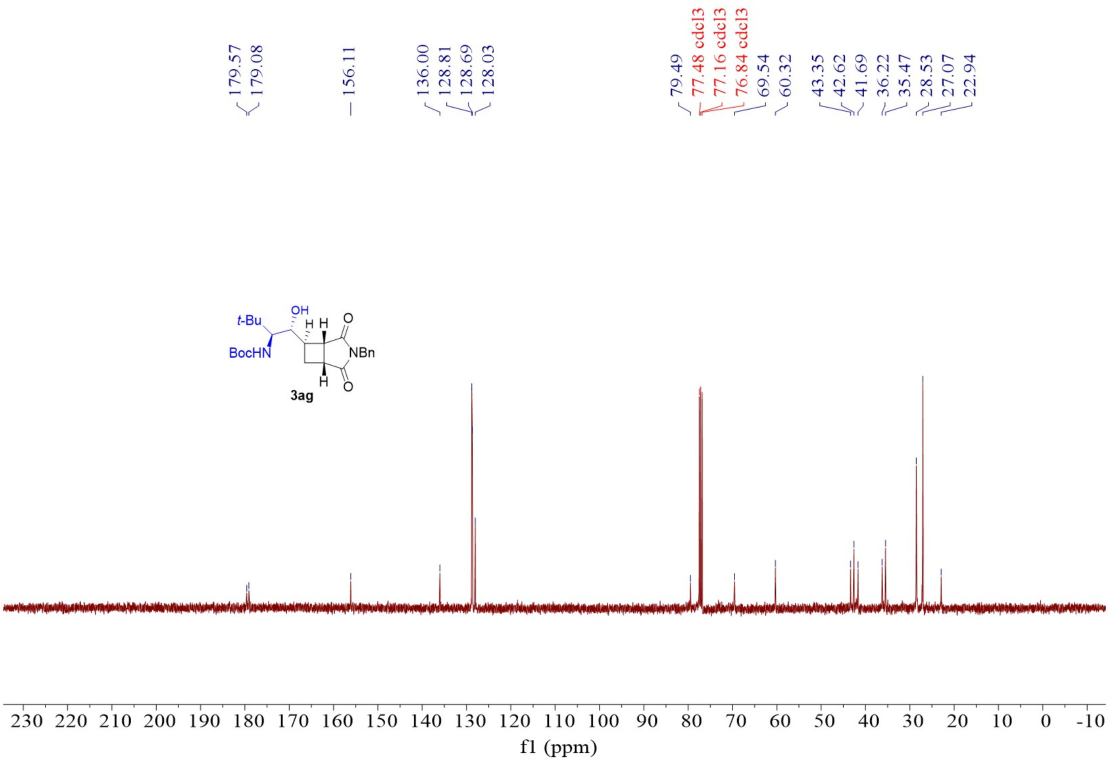
179.57
179.08
156.11
136.00
128.81
128.69
128.03
79.49
77.48 cdcl3
77.16 cdcl3
76.84 cdcl3
69.54
60.32
43.35
42.62
41.69
36.22
35.47
28.53
27.07
22.94
t-Bu
OH
H H
O
BocHN
NBn
H
O
3ag
230 220 210 200 190 180 170 160 150 140 130 120 110 100 90 80 70 60 50 40 30 20 10 0 -10
f1 (ppm)

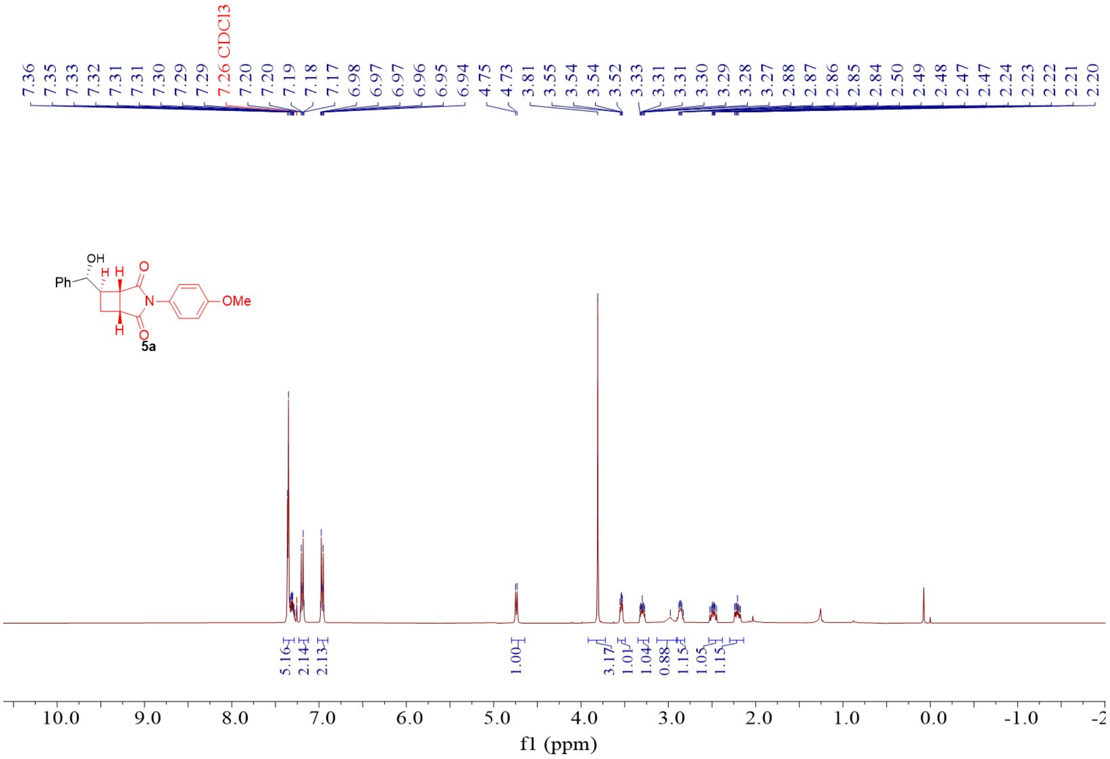
7.36
7.35
7.33
7.32
7.31
7.31
7.30
7.29
7.29
7.26 CDCl3
7.20
7.20
7.19
7.18
7.17
6.98
6.97
6.97
6.96
6.95
6.94
4.75
4.73
3.81
3.55
3.54
3.54
3.52
3.33
3.31
3.31
3.30
3.29
3.28
3.27
2.88
2.87
2.86
2.85
2.84
2.50
2.49
2.48
2.47
2.47
2.24
2.23
2.22
2.21
2.20
OH
Ph
H H
O
N
OMe
H
O
5a
5.16
2.14
2.13
1.00
3.17
1.01
1.04
0.88
1.15
1.05
1.15
10.0 9.0 8.0 7.0 6.0 5.0 4.0 3.0 2.0 1.0 0.0 -1.0
f1 (ppm)

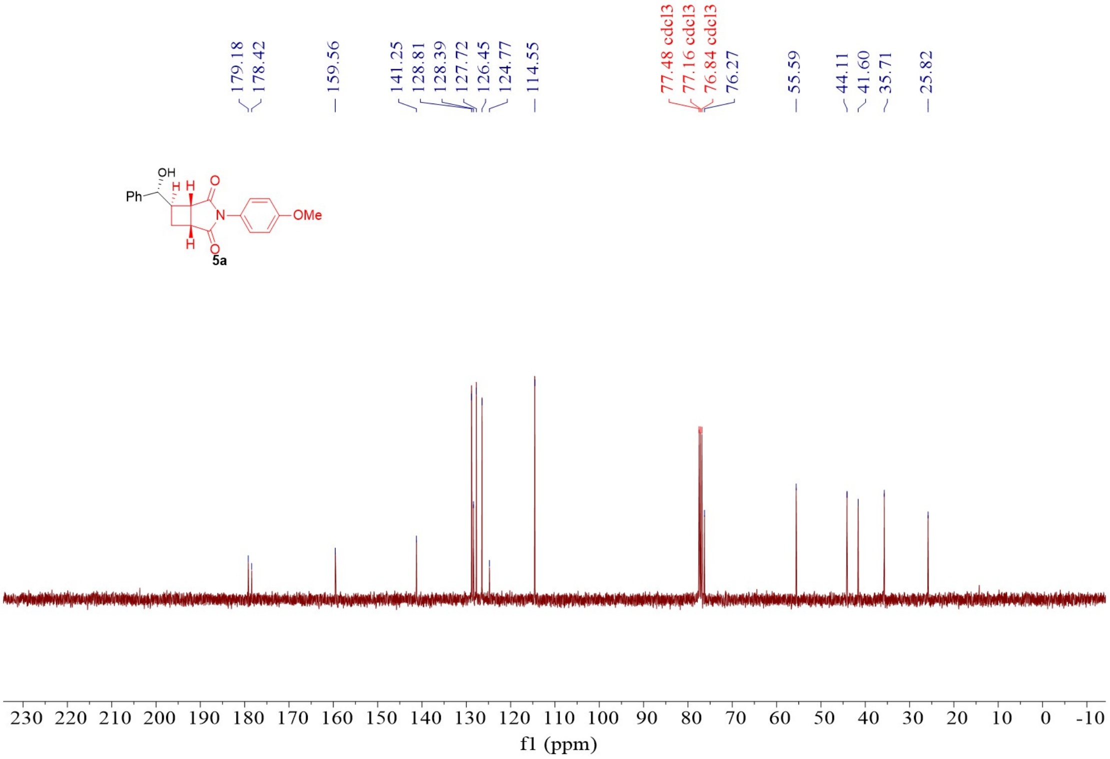
179.18
178.42
159.56
141.25
128.81
128.39
127.72
126.45
124.77
114.55
77.48 cdcl3
77.16 cdcl3
76.84 cdcl3
76.27
55.59
44.11
41.60
35.71
25.82
OH
Ph
H
H
O
N
OMe
O
H
5a
230 220 210 200 190 180 170 160 150 140 130 120 110 100 90 80 70 60 50 40 30 20 10 0 -10
f1 (ppm)

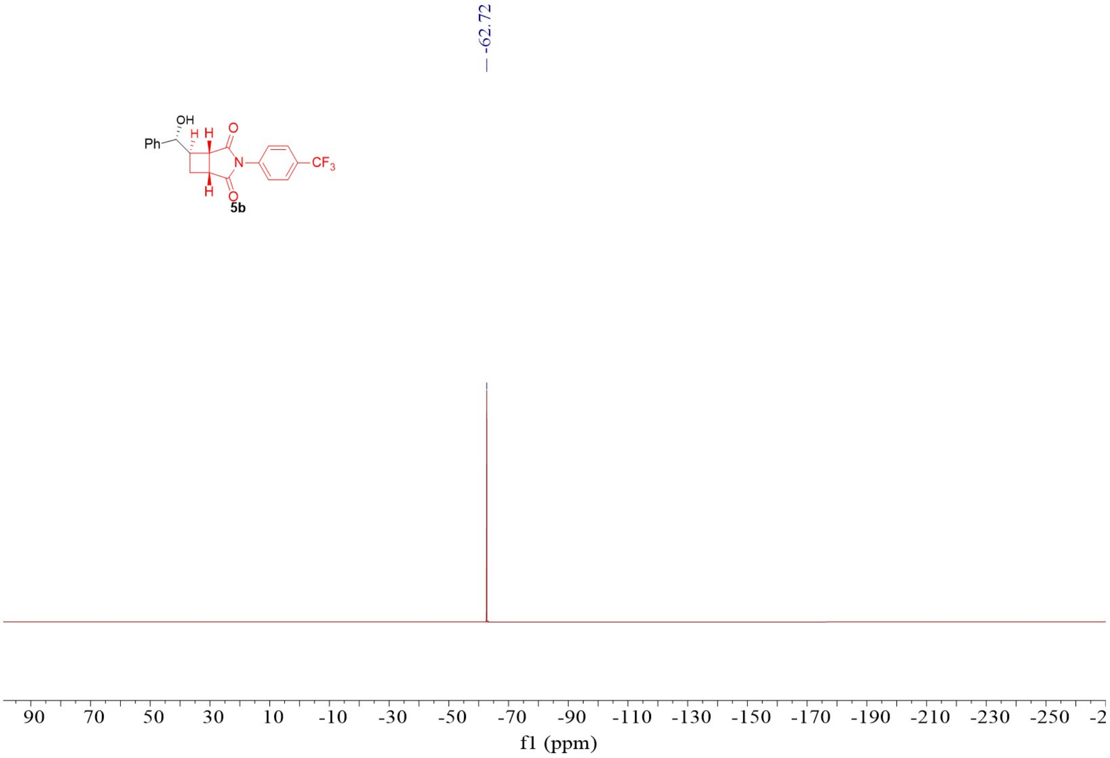
-62.72
OH
Ph
H
H
O
N
CF3
O
H
5b
90 70 50 30 10 -10 -30 -50 -70 -90 -110 -130 -150 -170 -190 -210 -230 -250 -2
f1 (ppm)

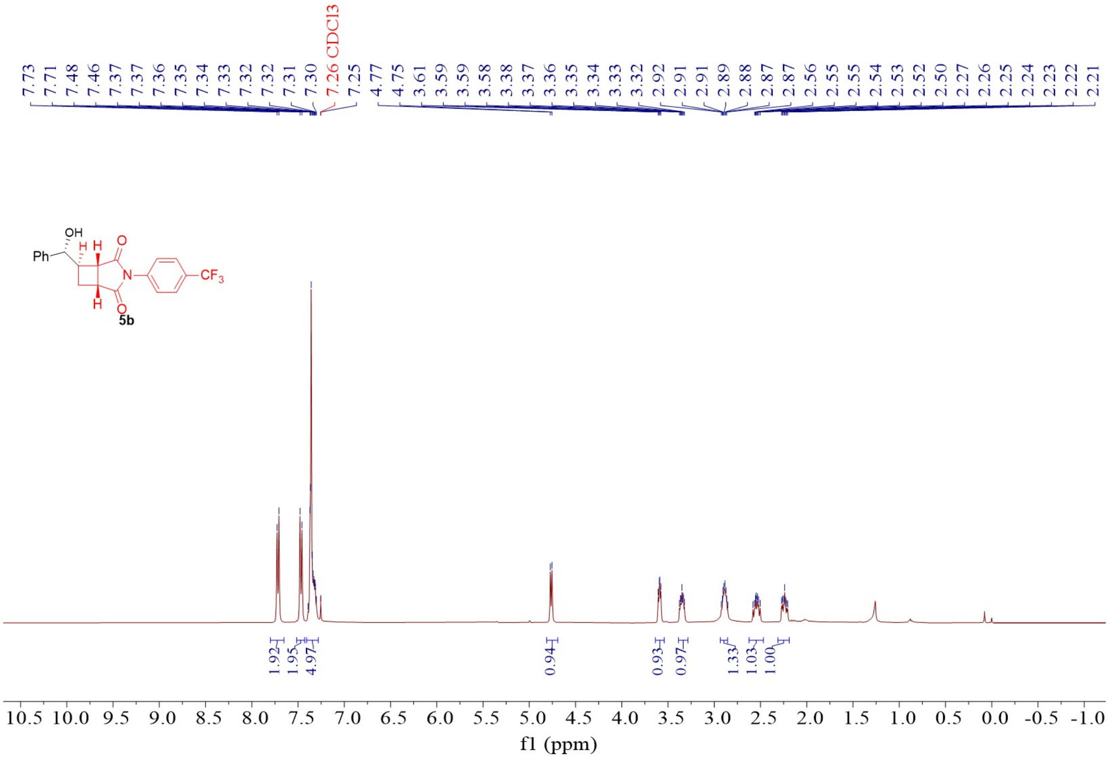
7.73 7.71 7.48 7.46 7.37 7.37 7.36 7.35 7.34 7.33 7.32 7.32 7.31 7.30 7.26 CDCl3 7.25 4.77 4.75 3.61 3.59 3.59 3.58 3.38 3.37 3.36 3.35 3.34 3.33 3.32 2.92 2.91 2.91 2.89 2.88 2.87 2.87 2.56 2.55 2.55 2.54 2.53 2.52 2.50 2.27 2.26 2.25 2.24 2.23 2.22 2.21
Ph
OH
H
H
O
O
N
CF3
5b
1.92
1.95
4.97
0.94
0.93
0.97
1.33
1.03
1.00
10.5 10.0 9.5 9.0 8.5 8.0 7.5 7.0 6.5 6.0 5.5 5.0 4.5 4.0 3.5 3.0 2.5 2.0 1.5 1.0 0.5 0.0 -0.5 -1.0
f1 (ppm)

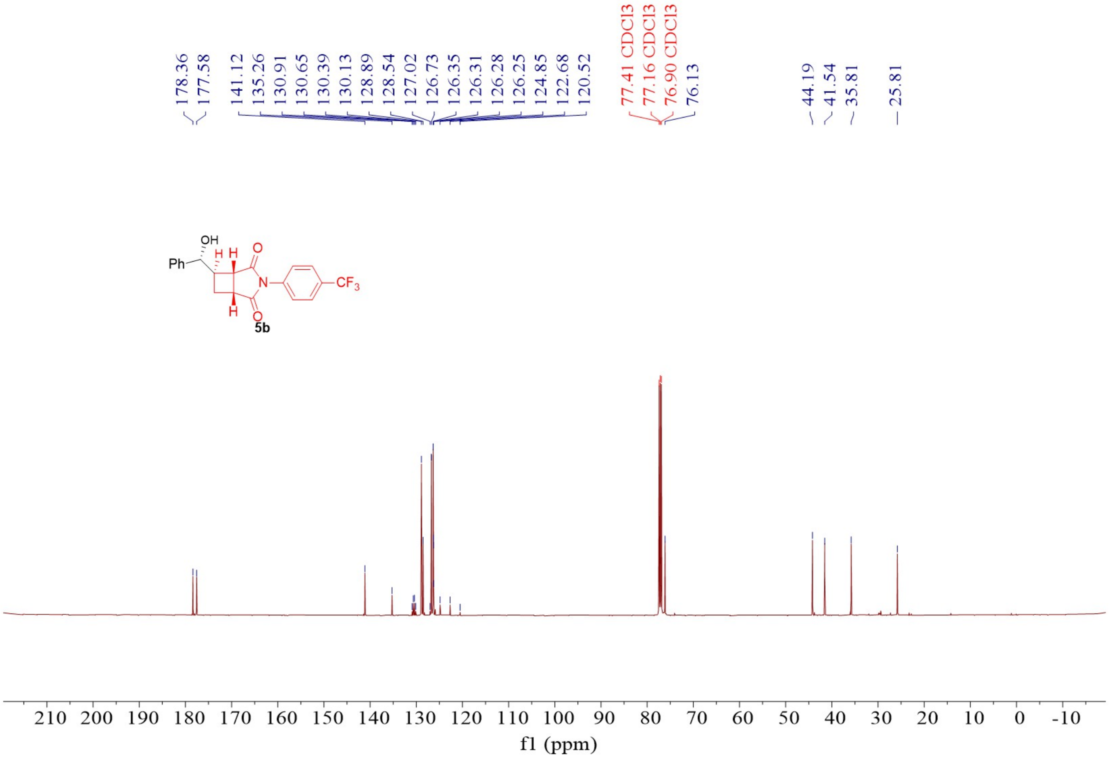
178.36 177.58 141.12 135.26 130.91 130.65 130.39 130.13 128.89 128.54 127.02 126.73 126.35 126.31 126.28 126.25 124.85 122.68 120.52 77.41 CDCl3 77.16 CDCl3 76.90 CDCl3 76.13 44.19 41.54 35.81 25.81
Ph
OH
H
H
O
O
N
CF3
5b
210 200 190 180 170 160 150 140 130 120 110 100 90 80 70 60 50 40 30 20 10 0 -10
f1 (ppm)

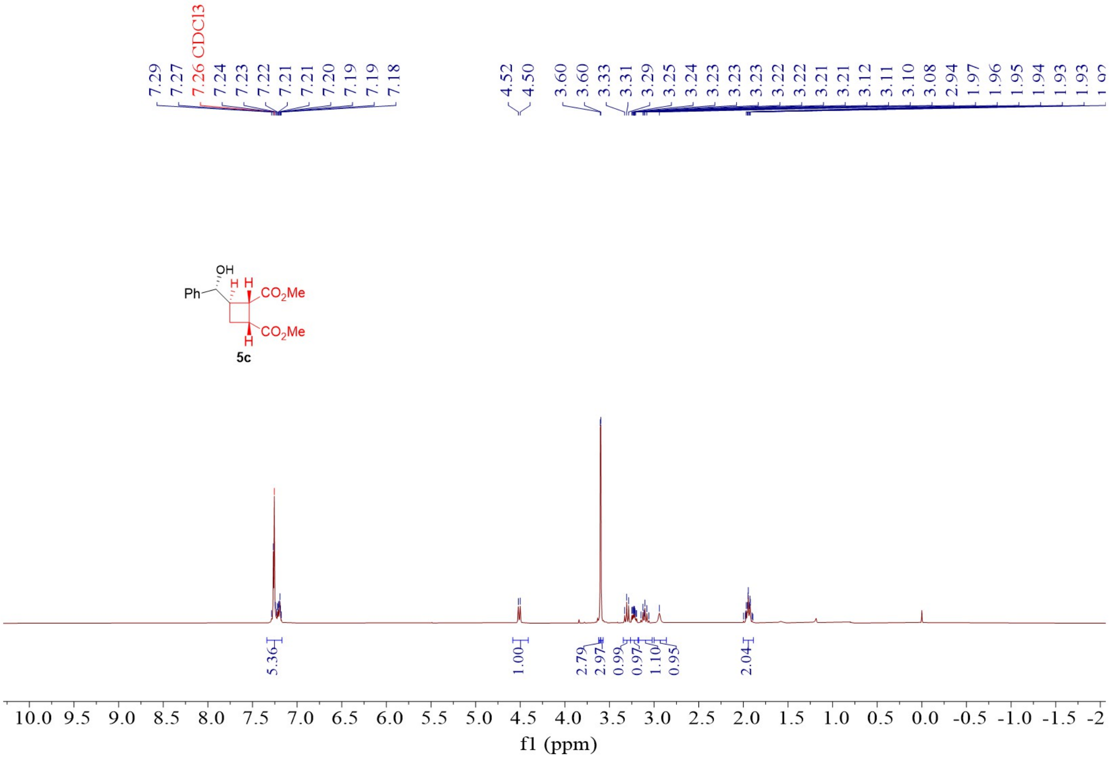

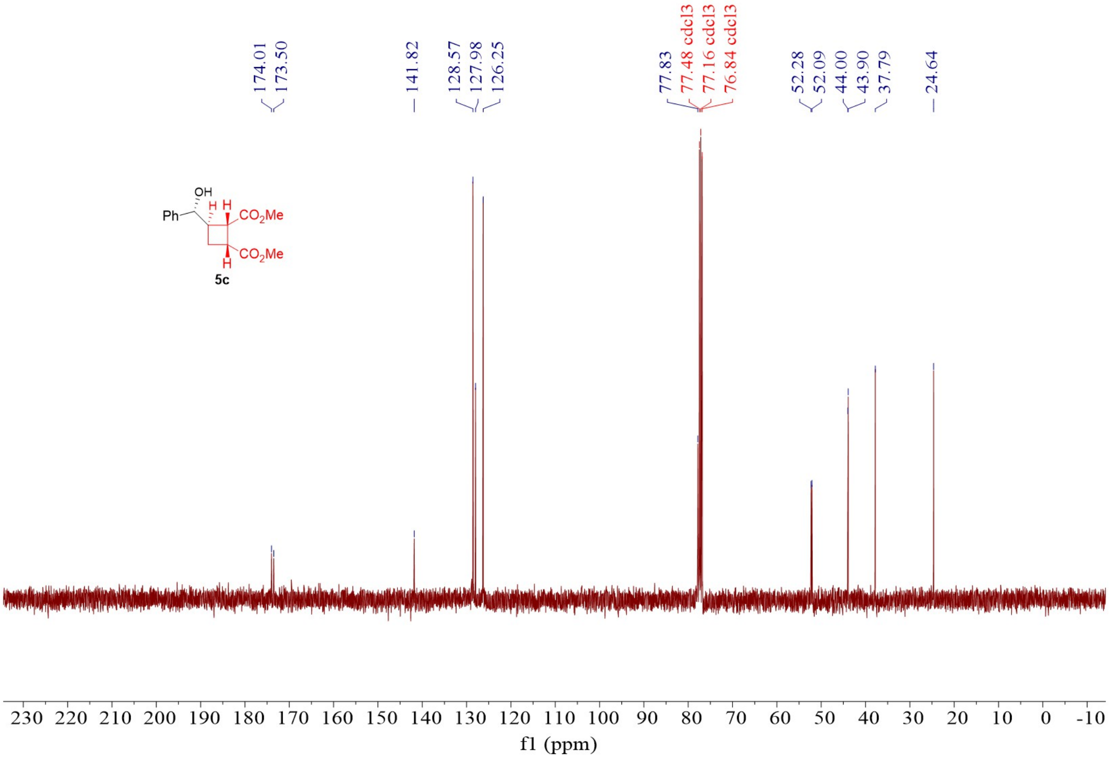

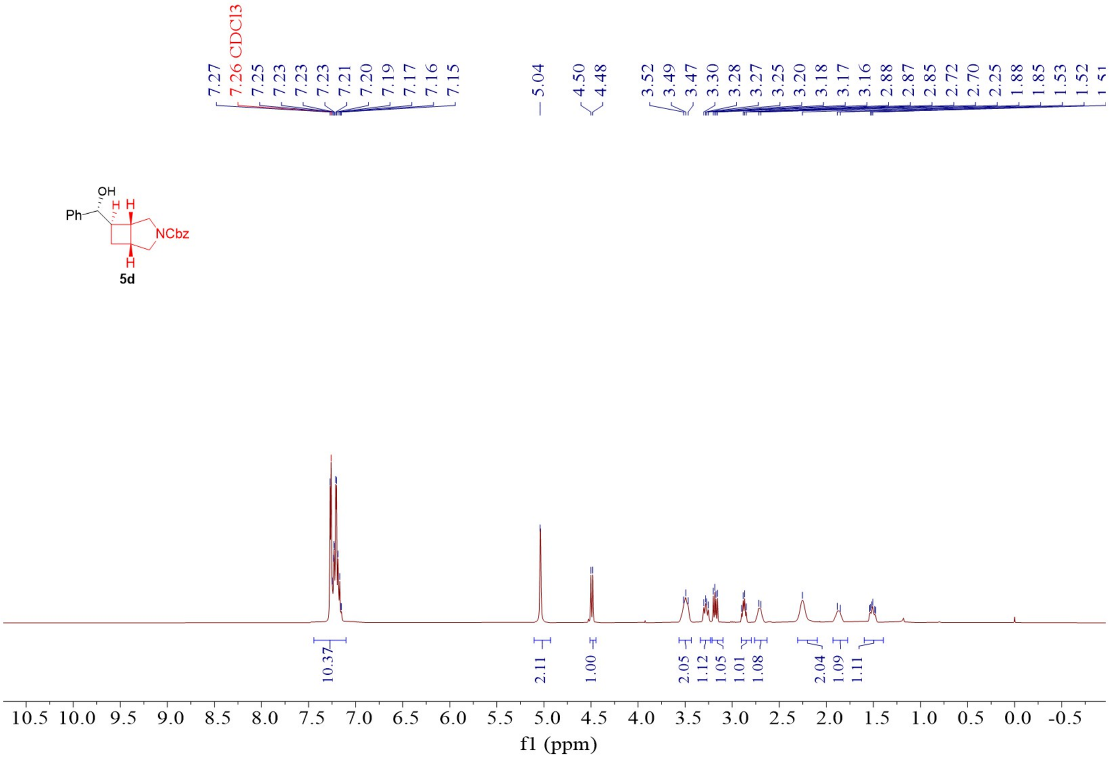
7.27
7.26 CDCl3
7.25
7.23
7.23
7.23
7.21
7.20
7.19
7.17
7.16
7.15
5.04
4.50
4.48
3.52
3.49
3.47
3.30
3.28
3.27
3.25
3.20
3.18
3.17
3.16
2.88
2.87
2.85
2.72
2.70
2.25
1.88
1.85
1.53
1.52
OH
Ph
H H
NCbz
H
5d
10.37
2.11
1.00
2.05
1.12
1.05
1.01
1.08
2.04
1.09
1.11
10.5 10.0 9.5 9.0 8.5 8.0 7.5 7.0 6.5 6.0 5.5 5.0 4.5 4.0 3.5 3.0 2.5 2.0 1.5 1.0 0.5 0.0 -0.5
f1 (ppm)

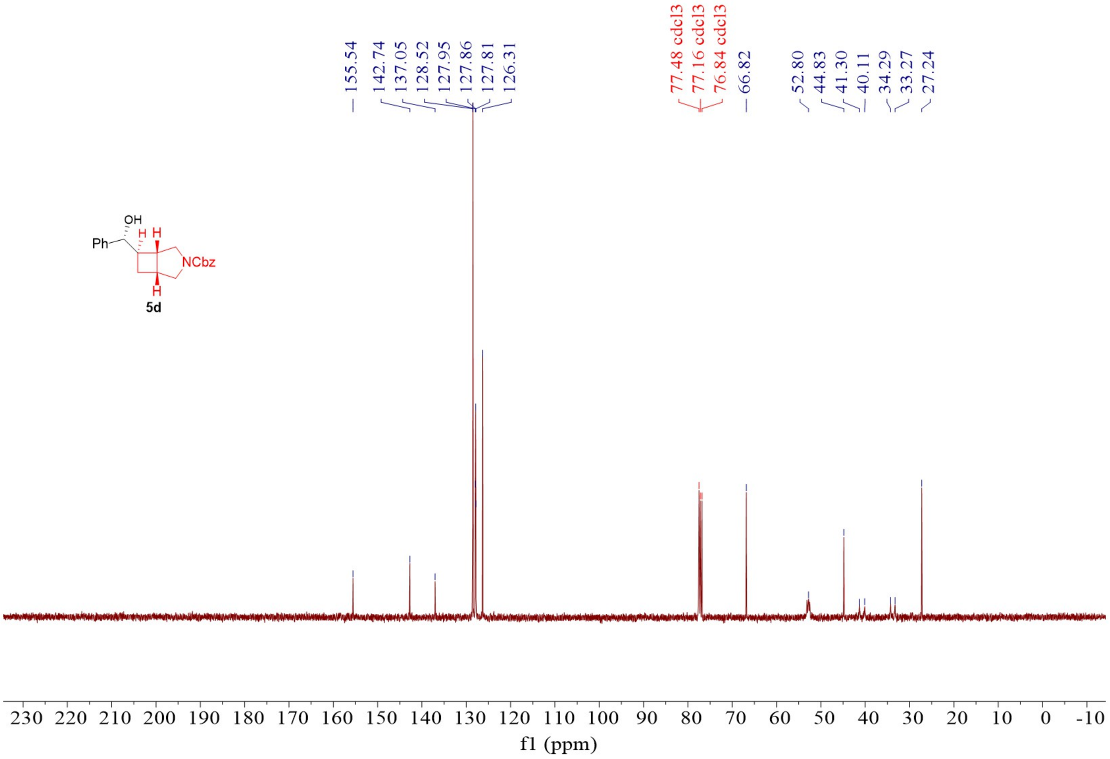
155.54
142.74
137.05
128.52
127.95
127.86
127.81
126.31
77.48 cdcl3
77.16 cdcl3
76.84 cdcl3
66.82
52.80
44.83
41.30
40.11
34.29
33.27
27.24
OH
Ph
H H
NCbz
H
5d
230 220 210 200 190 180 170 160 150 140 130 120 110 100 90 80 70 60 50 40 30 20 10 0 -10
f1 (ppm)

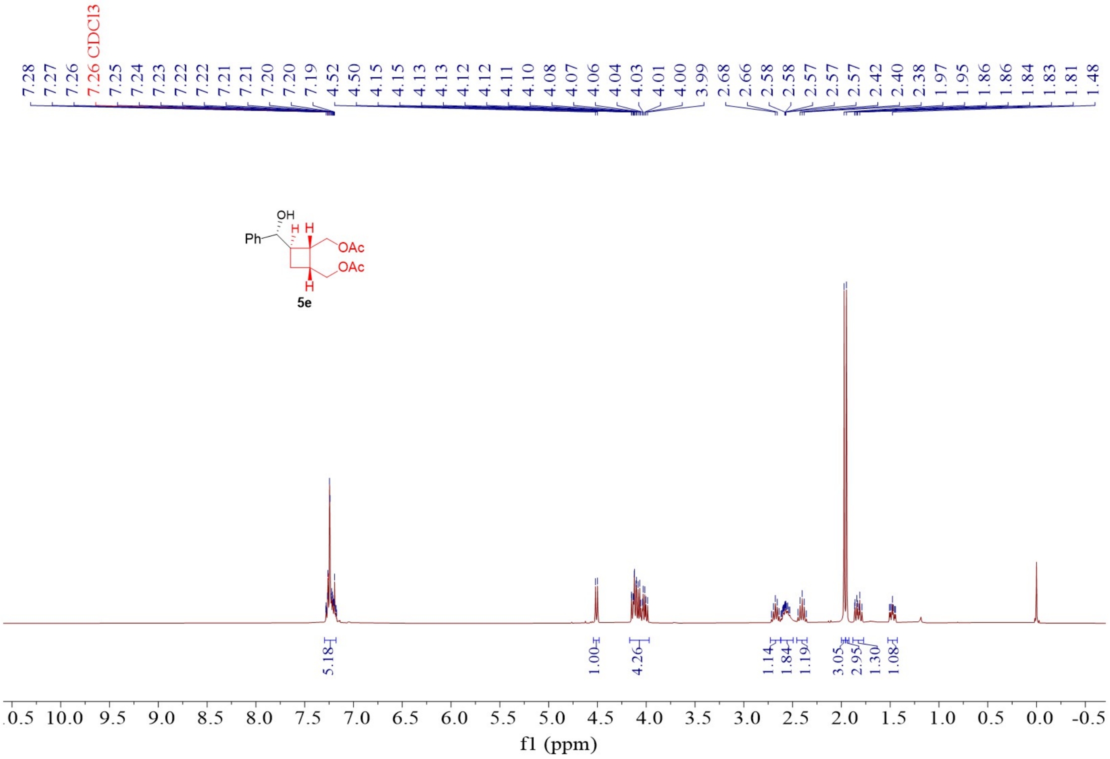
7.28
7.27
7.26
7.26 CDCl3
7.25
7.24
7.23
7.22
7.22
7.21
7.21
7.20
7.20
7.19
4.52
4.50
4.15
4.15
4.13
4.13
4.12
4.12
4.11
4.10
4.08
4.07
4.06
4.04
4.03
4.01
4.00
3.99
2.68
2.66
2.58
2.58
2.57
2.57
2.57
2.42
2.40
2.38
1.97
1.95
1.86
1.86
1.84
1.83
1.81
1.48
OH
Ph
H H
OAc
OAc
H
5e
5.18
1.00
4.26
1.14
1.84
1.19
3.05
2.95
1.30
1.08
.0.5 10.0 9.5 9.0 8.5 8.0 7.5 7.0 6.5 6.0 5.5 5.0 4.5 4.0 3.5 3.0 2.5 2.0 1.5 1.0 0.5 0.0 -0.5
f1 (ppm)

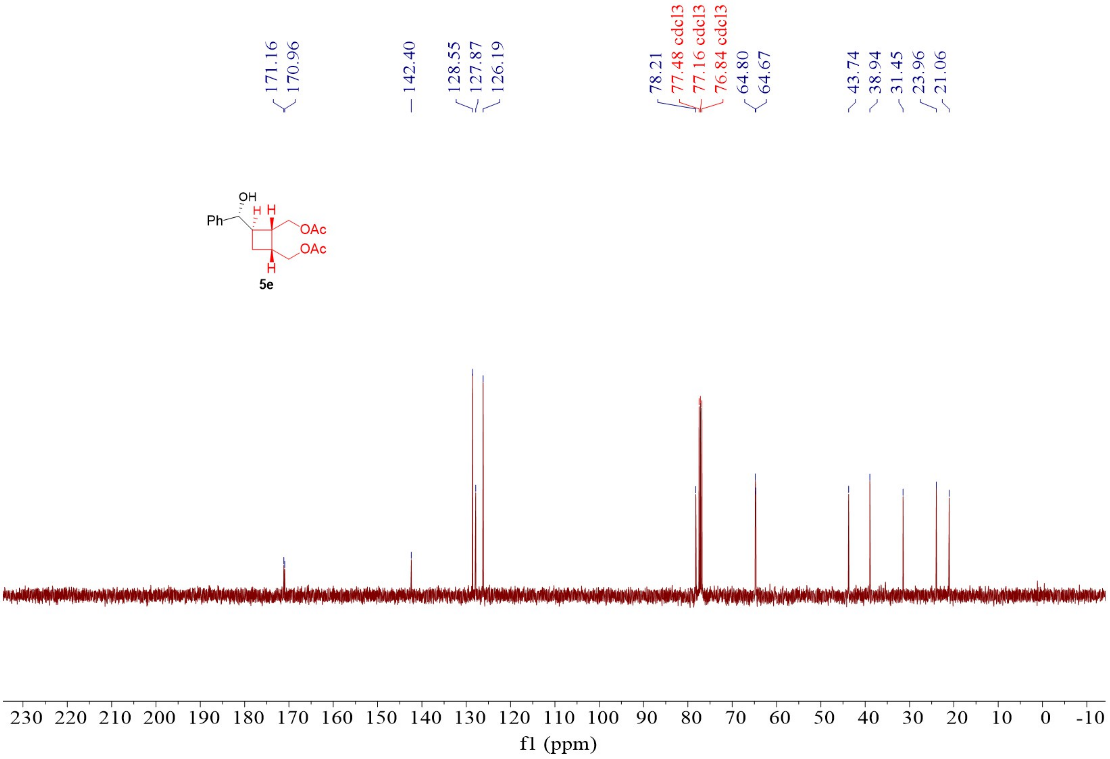
171.16
170.96
142.40
128.55
127.87
126.19
78.21
77.48 cdcl3
77.16 cdcl3
76.84 cdcl3
64.80
64.67
43.74
38.94
31.45
23.96
21.06
OH
Ph
H H
OAc
OAc
H
5e
230 220 210 200 190 180 170 160 150 140 130 120 110 100 90 80 70 60 50 40 30 20 10 0 -10
f1 (ppm)

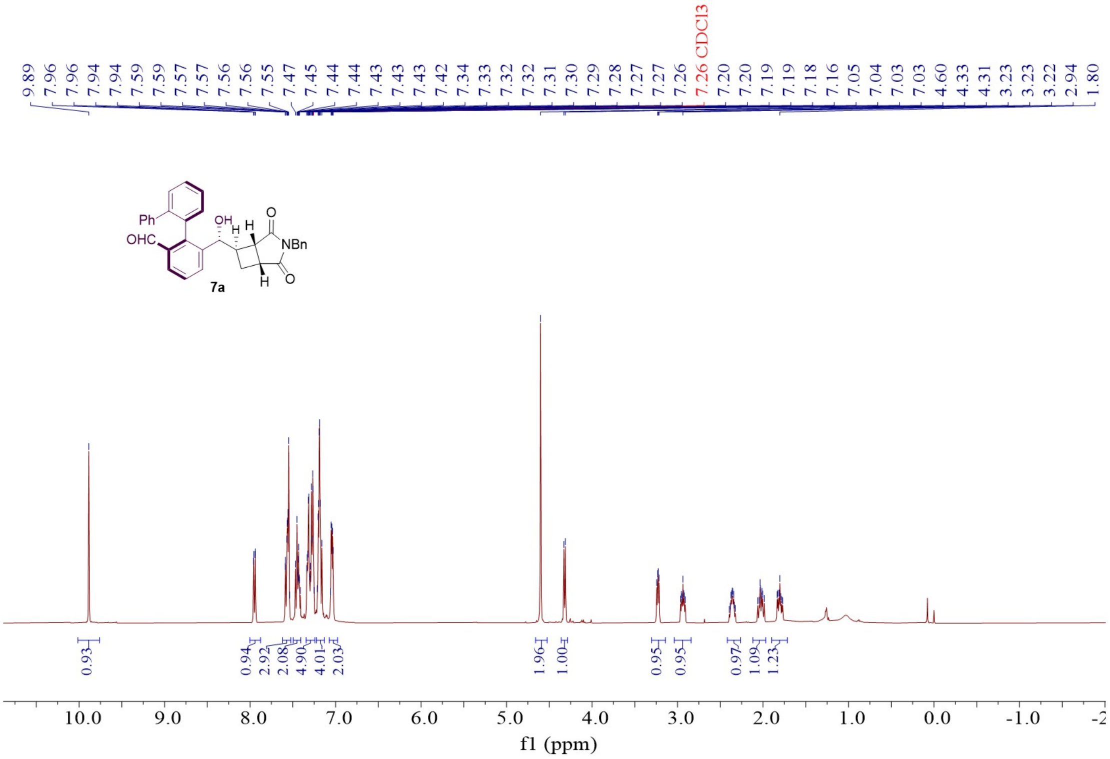
9.89
7.96
7.96
7.94
7.94
7.59
7.59
7.57
7.57
7.56
7.56
7.55
7.47
7.45
7.44
7.44
7.43
7.43
7.43
7.42
7.34
7.33
7.32
7.32
7.31
7.30
7.29
7.28
7.27
7.27
7.26
7.26 CDCl3
7.20
7.20
7.19
7.19
7.18
7.16
7.05
7.04
7.03
7.03
4.60
4.33
4.31
3.23
3.23
3.22
2.94
1.80
Ph
OHC
OH
H
O
NBn
H
O
7a
0.93
0.94
2.92
2.08
4.90
4.01
2.03
1.96
1.00
0.95
0.95
0.97
1.09
1.23
10.0
9.0
8.0
7.0
6.0
5.0
4.0
3.0
2.0
1.0
0.0
-1.0
f1 (ppm)

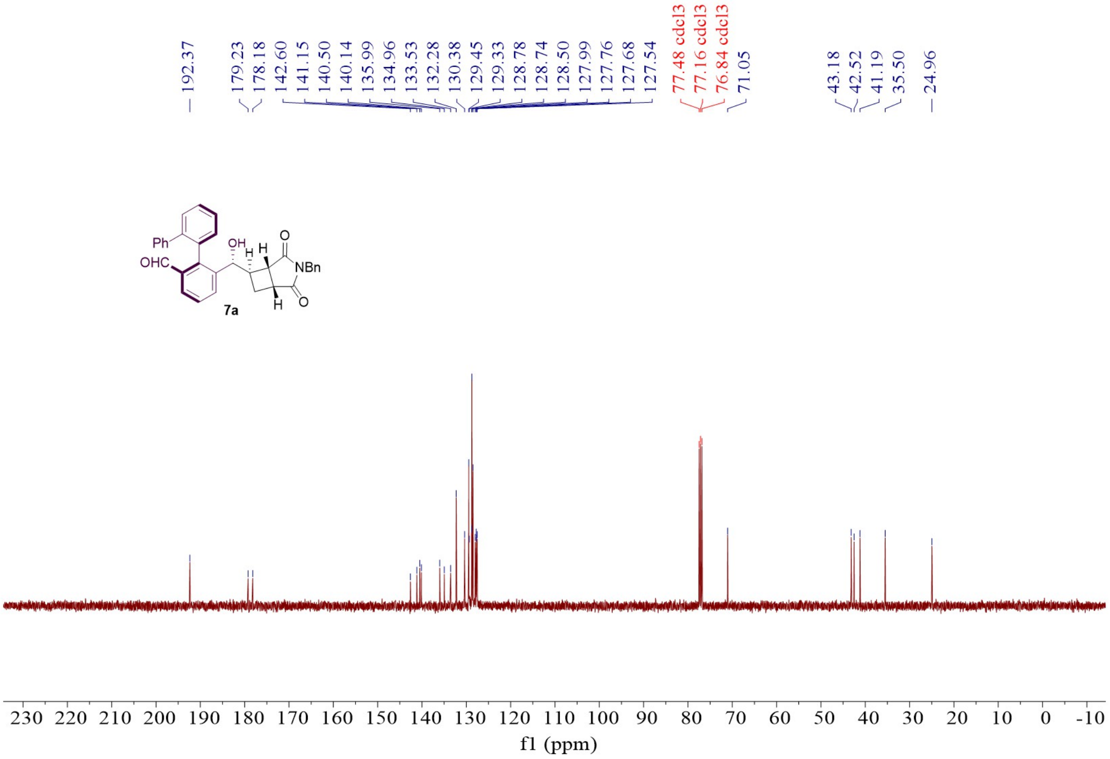
192.37
179.23
178.18
142.60
141.15
140.50
140.14
135.99
134.96
133.53
132.28
130.38
129.45
129.33
128.78
128.74
128.50
127.99
127.76
127.68
127.54
77.48 cdcl3
77.16 cdcl3
76.84 cdcl3
71.05
43.18
42.52
41.19
35.50
24.96
Ph
OHC
OH
H
O
NBn
H
O
7a
230 220 210 200 190 180 170 160 150 140 130 120 110 100 90 80 70 60 50 40 30 20 10 0 -10
f1 (ppm)

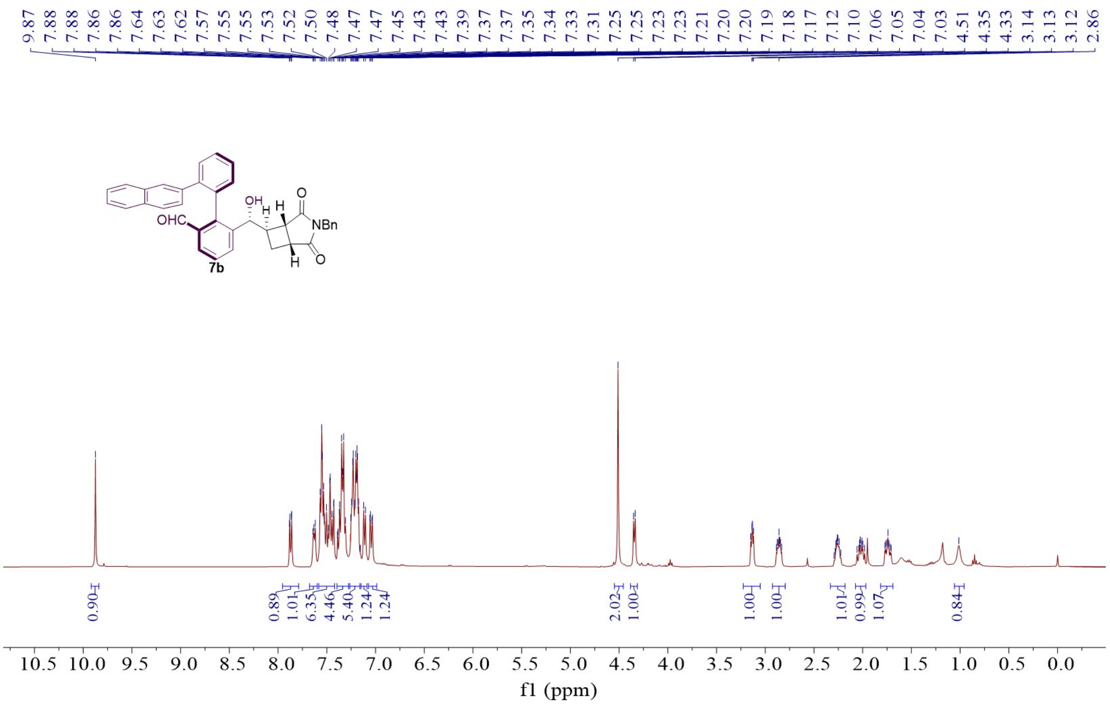

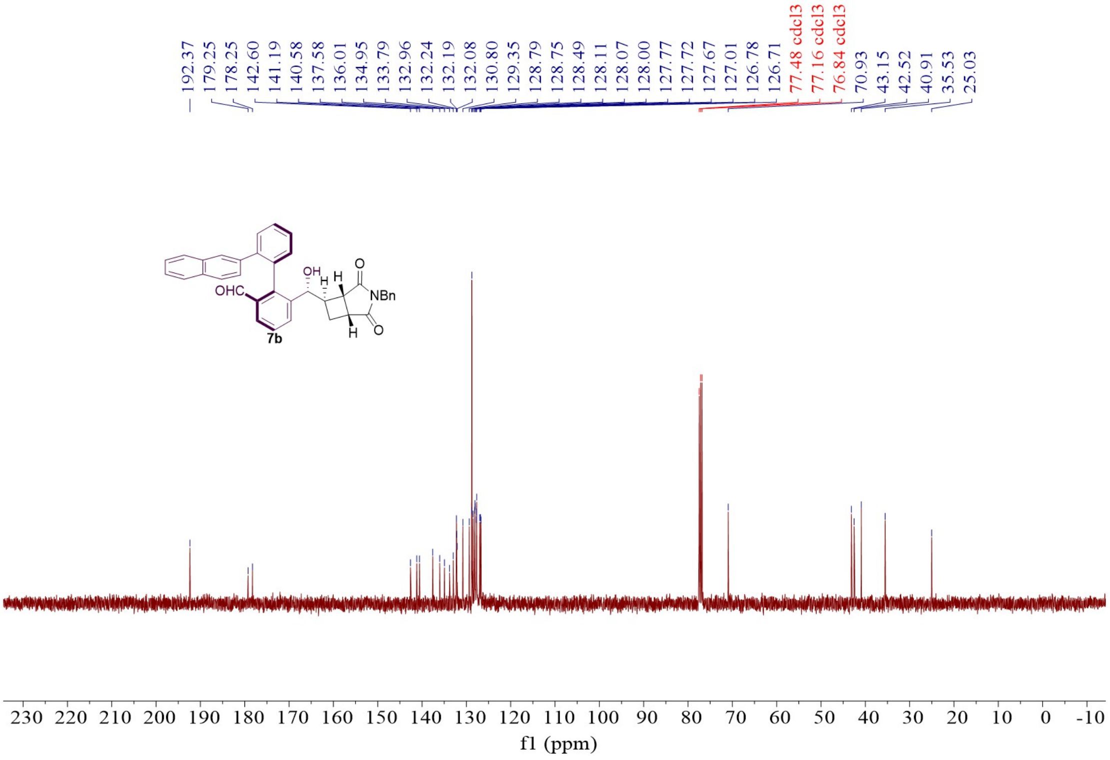

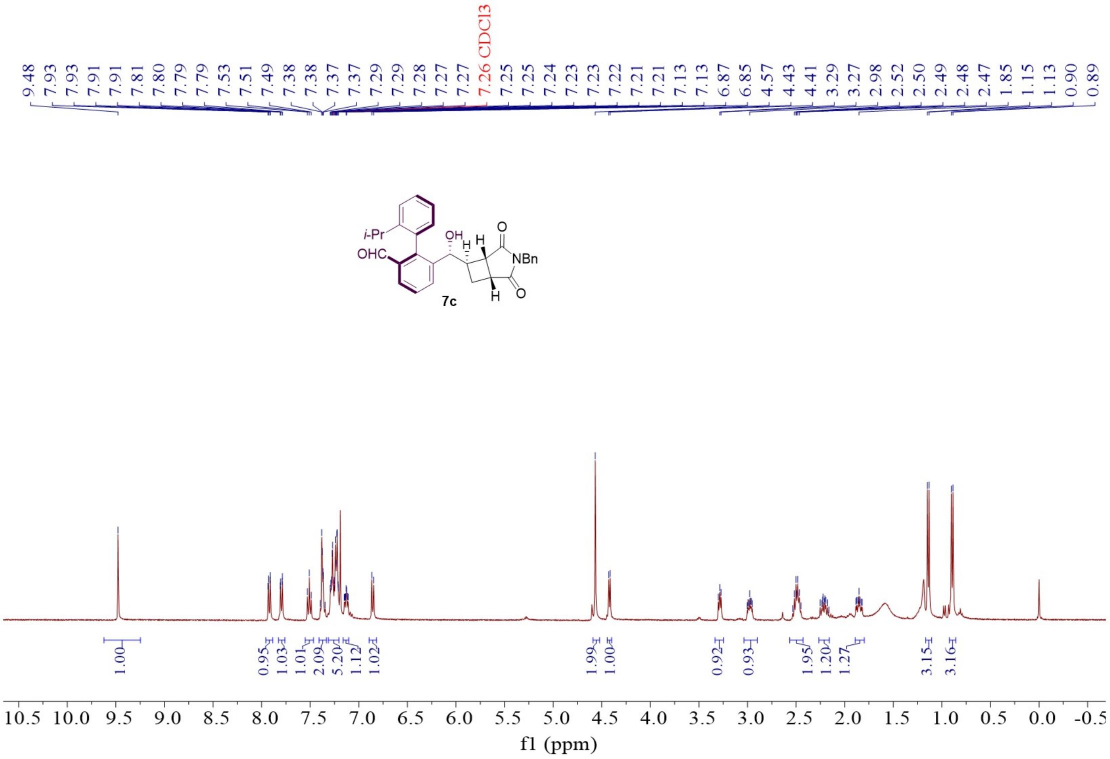
9.48 7.93 7.93 7.91 7.91 7.81 7.80 7.79 7.79 7.53 7.51 7.49 7.38 7.38 7.37 7.37 7.29 7.29 7.28 7.27 7.27 7.26 CDCl3 7.25 7.25 7.24 7.23 7.23 7.22 7.21 7.21 7.13 7.13 6.87 6.85 4.57 4.43 4.41 3.29 3.27 2.98 2.52 2.50 2.49 2.48 2.47 1.85 1.15 1.13 0.90 0.89
i-Pr
OHC
OH
H
O
NBn
H
O
7c
1.00
0.95
1.03
1.01
2.09
5.20
1.12
1.02
1.99
1.00
0.92
0.93
1.95
1.20
1.27
3.15
3.16
10.5 10.0 9.5 9.0 8.5 8.0 7.5 7.0 6.5 6.0 5.5 5.0 4.5 4.0 3.5 3.0 2.5 2.0 1.5 1.0 0.5 0.0 -0.5
f1 (ppm)

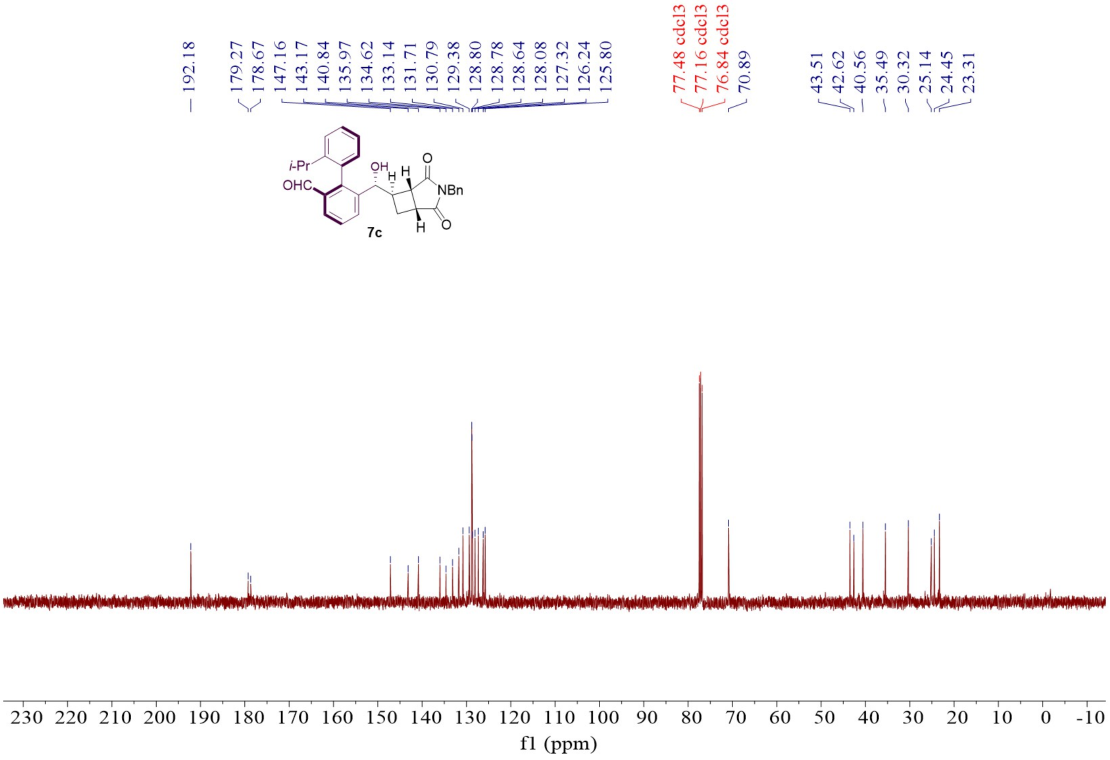
192.18
179.27 178.67
147.16 143.17 140.84 135.97 134.62 133.14 131.71 130.79 129.38 128.80 128.78 128.64 128.08 127.32 126.24 125.80
77.48 cdcl3 77.16 cdcl3 76.84 cdcl3
70.89
43.51 42.62 40.56 35.49 30.32 25.14 24.45 23.31
i-Pr
OHC
OH
H
O
NBn
H
O
7c
230 220 210 200 190 180 170 160 150 140 130 120 110 100 90 80 70 60 50 40 30 20 10 0 -10
f1 (ppm)

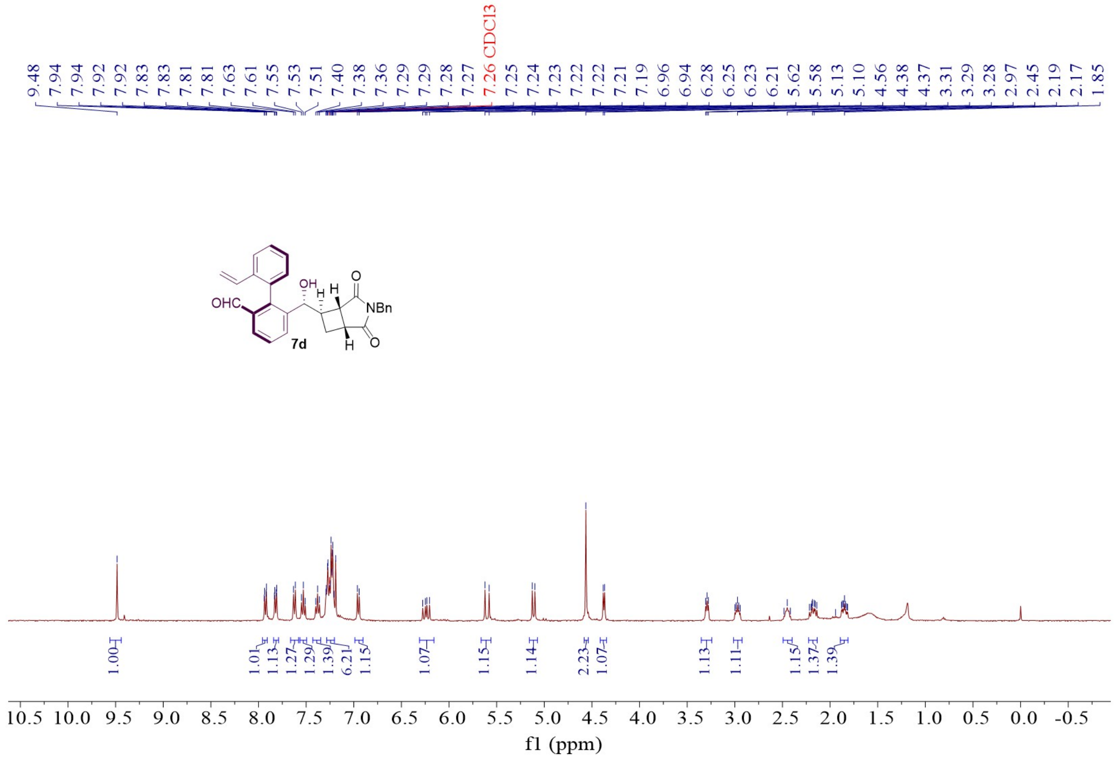
9.48 7.94 7.94 7.92 7.92 7.83 7.83 7.81 7.81 7.63 7.61 7.55 7.53 7.51 7.40 7.38 7.36 7.29 7.29 7.28 7.27 7.26 CDCl3 7.25 7.24 7.23 7.22 7.22 7.21 7.19 6.96 6.94 6.28 6.25 6.23 6.21 5.62 5.58 5.13 5.10 4.56 4.38 4.37 3.31 3.29 3.28 2.97 2.45 2.19 2.17 1.85
OHC
OH
H
NBn
7d
1.00 1.01 1.13 1.27 1.29 1.39 6.21 1.15 1.07 1.15 1.14 2.23 1.07 1.13 1.11 1.15 1.37 1.39
10.5 10.0 9.5 9.0 8.5 8.0 7.5 7.0 6.5 6.0 5.5 5.0 4.5 4.0 3.5 3.0 2.5 2.0 1.5 1.0 0.5 0.0 -0.5
f1 (ppm)

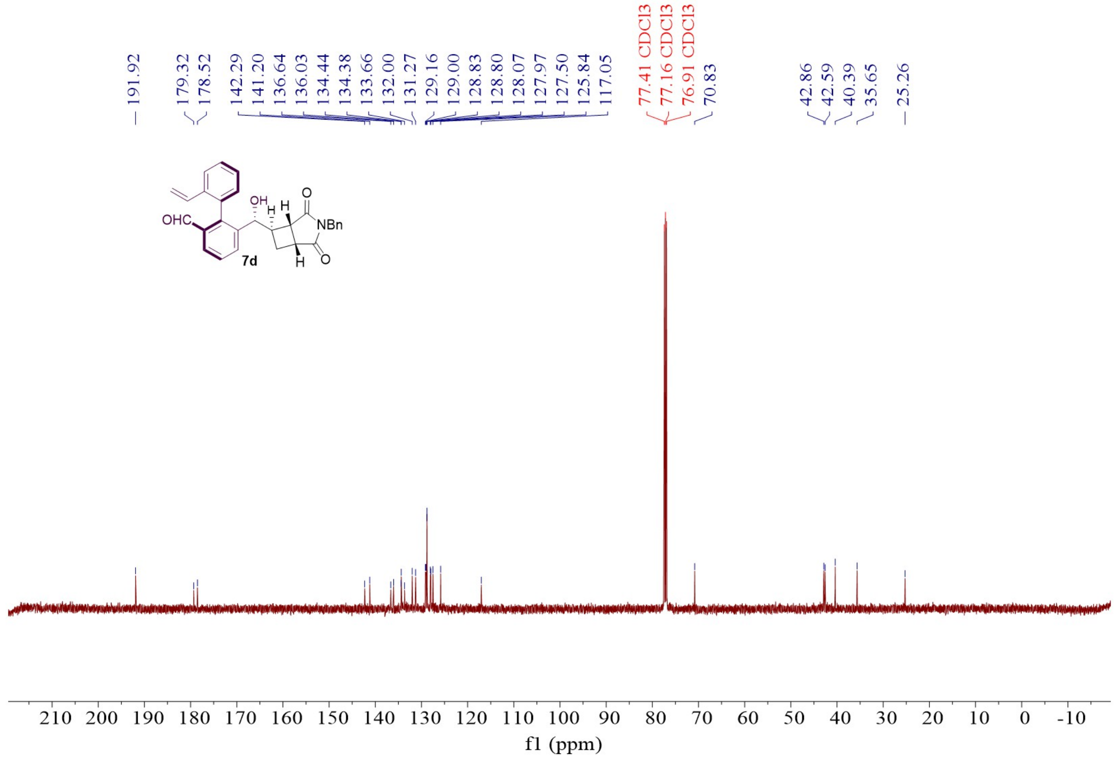
191.92 179.32 178.52 142.29 141.20 136.64 136.03 134.44 134.38 133.66 132.00 131.27 129.16 129.00 128.83 128.80 128.07 127.97 127.50 125.84 117.05 77.41 CDCl3 77.16 CDCl3 76.91 CDCl3 70.83 42.86 42.59 40.39 35.65 25.26
OHC
OH
H
NBn
7d
210 200 190 180 170 160 150 140 130 120 110 100 90 80 70 60 50 40 30 20 10 0 -10
f1 (ppm)

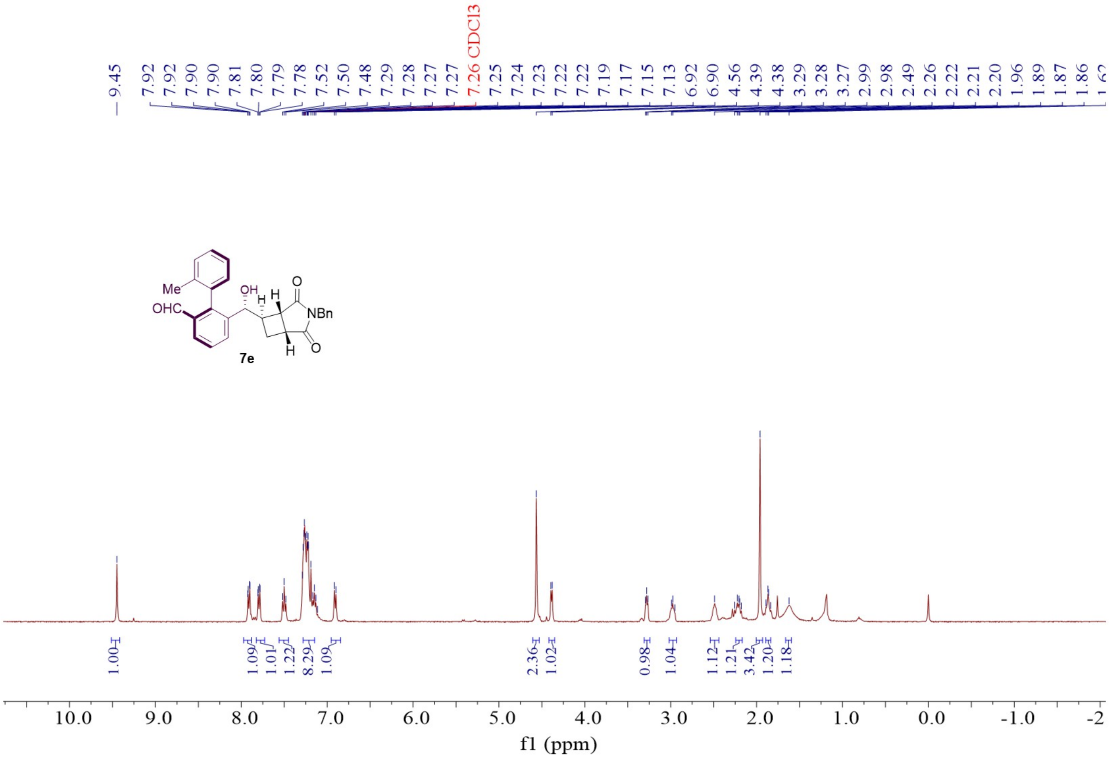
9.45
7.92
7.92
7.90
7.90
7.81
7.80
7.79
7.78
7.52
7.50
7.48
7.29
7.28
7.27
7.27
7.26 CDCl3
7.25
7.24
7.23
7.22
7.22
7.19
7.17
7.15
7.13
6.92
6.90
4.56
4.39
4.38
3.29
3.28
3.27
2.99
2.98
2.49
2.26
2.22
2.21
2.20
1.96
1.89
1.87
1.86
Me
OHC
OH
H
O
NBn
H
O
7e
1.00
1.09
1.01
1.22
8.29
1.09
2.36
1.02
0.98
1.04
1.12
1.21
3.42
1.20
1.18
10.0
9.0
8.0
7.0
6.0
5.0
4.0
3.0
2.0
1.0
0.0
-1.0
-2
f1 (ppm)

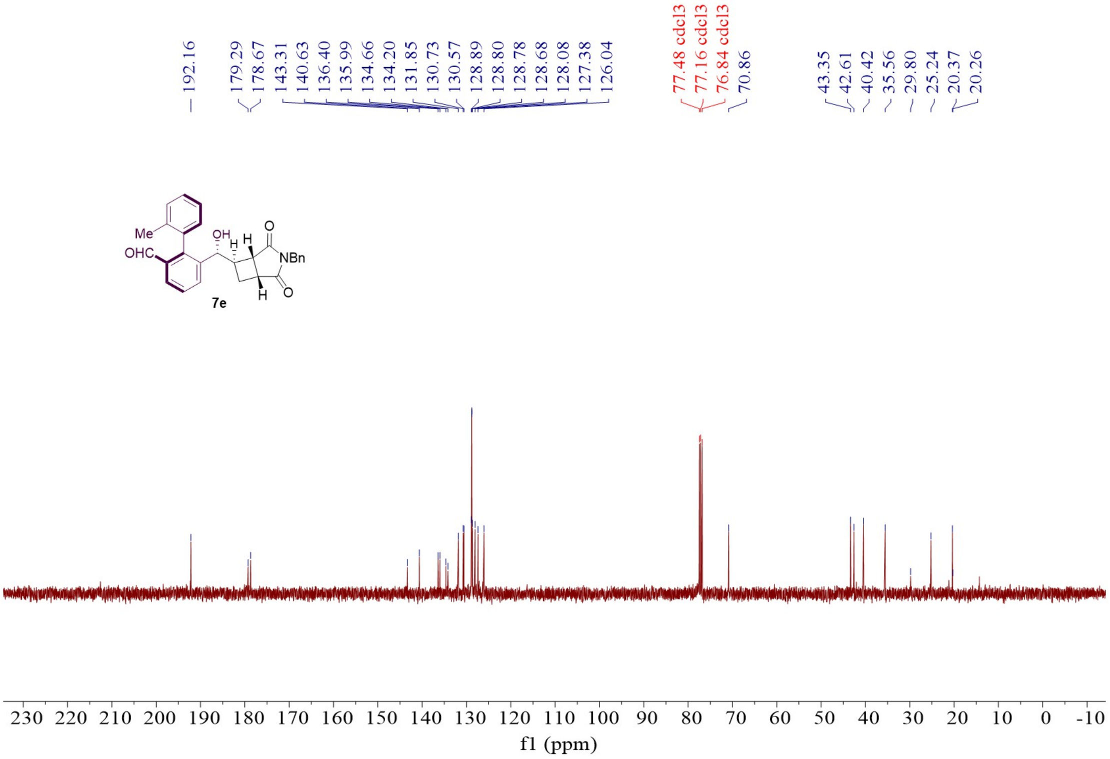
192.16
179.29
178.67
143.31
140.63
136.40
135.99
134.66
134.20
131.85
130.73
130.57
128.89
128.80
128.78
128.68
128.08
127.38
126.04
77.48 cdcl3
77.16 cdcl3
76.84 cdcl3
70.86
43.35
42.61
40.42
35.56
29.80
25.24
20.37
20.26
Me
OHC
OH
H
O
NBn
H
O
7e
230
220
210
200
190
180
170
160
150
140
130
120
110
100
90
80
70
60
50
40
30
20
10
0
-10
f1 (ppm)

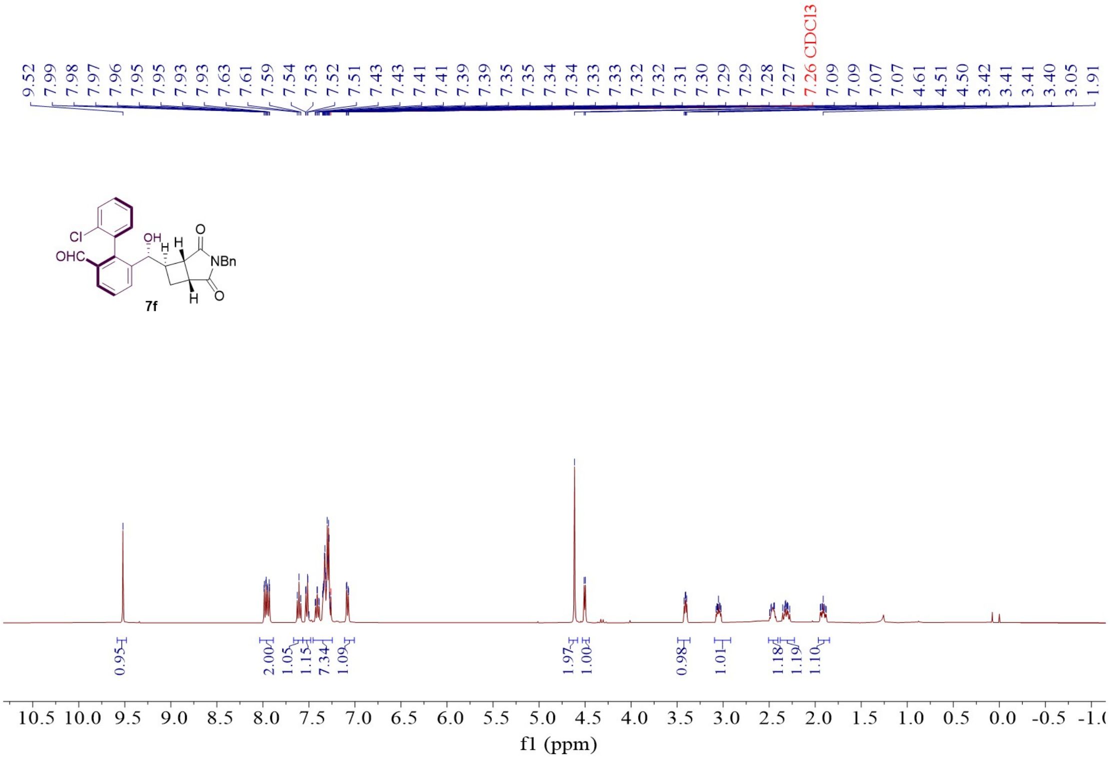
9.52
7.99
7.98
7.97
7.96
7.95
7.95
7.93
7.93
7.63
7.61
7.59
7.54
7.53
7.52
7.51
7.43
7.43
7.41
7.41
7.39
7.39
7.35
7.35
7.34
7.34
7.33
7.33
7.32
7.32
7.31
7.30
7.29
7.29
7.28
7.27
7.26 CDCl3
7.09
7.09
7.07
7.07
4.61
4.51
4.50
3.42
3.41
3.41
3.40
3.05
1.91
Cl
OHC
OH
H
H
O
NBn
H
O
7f
0.95
2.00
1.05
1.15
7.34
1.09
1.97
1.00
0.98
1.01
1.18
1.19
1.10
10.5 10.0 9.5 9.0 8.5 8.0 7.5 7.0 6.5 6.0 5.5 5.0 4.5 4.0 3.5 3.0 2.5 2.0 1.5 1.0 0.5 0.0 -0.5
f1 (ppm)

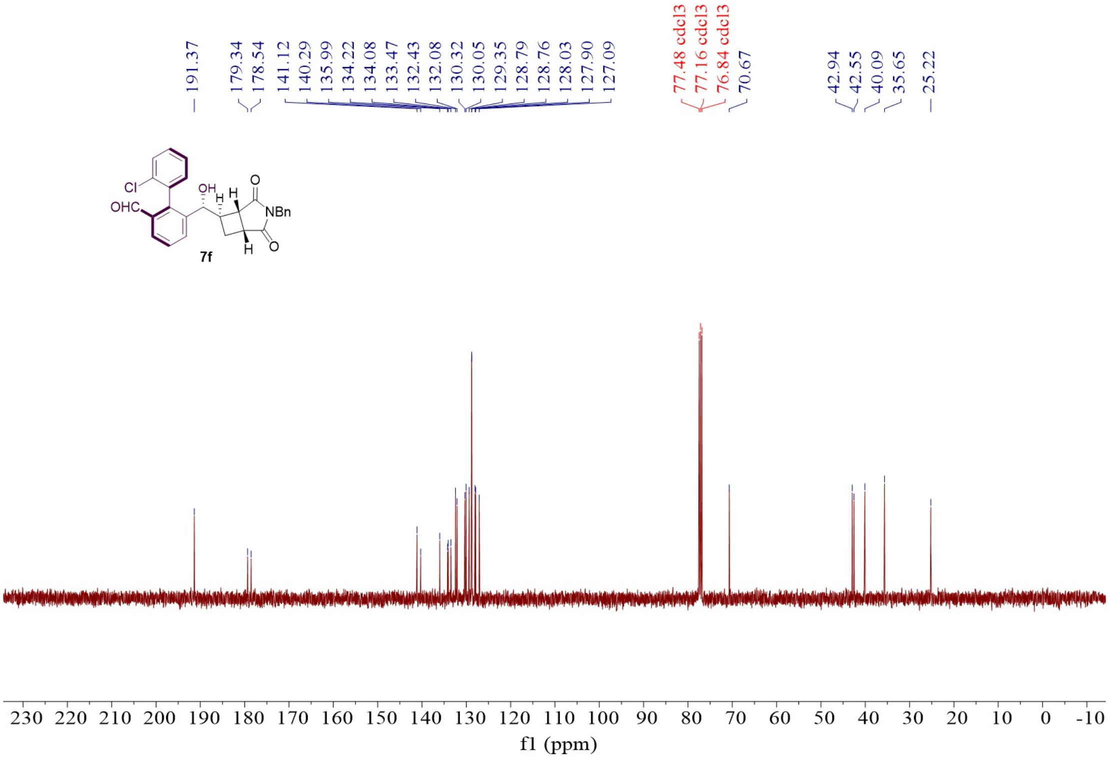
191.37
179.34
178.54
141.12
140.29
135.99
134.22
134.08
133.47
132.43
132.08
130.32
130.05
129.35
128.79
128.76
128.03
127.90
127.09
77.48 cdcl3
77.16 cdcl3
76.84 cdcl3
70.67
42.94
42.55
40.09
35.65
25.22
Cl
OHC
OH
H
H
O
NBn
H
O
7f
230 220 210 200 190 180 170 160 150 140 130 120 110 100 90 80 70 60 50 40 30 20 10 0 -10
f1 (ppm)

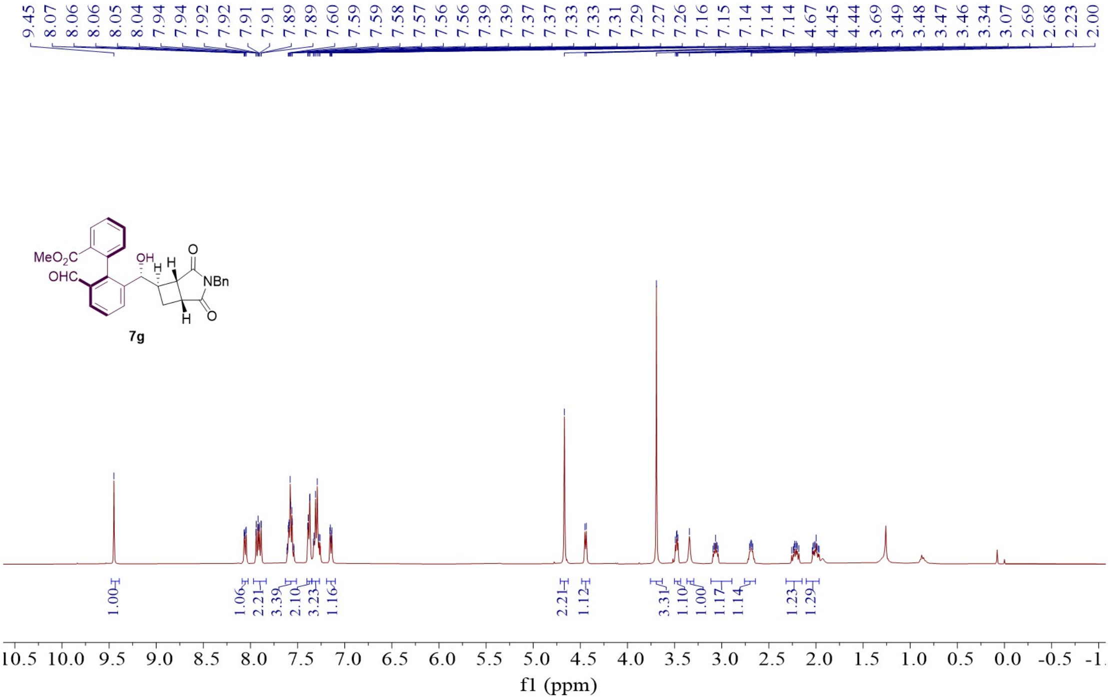
9.45
8.07
8.06
8.06
8.05
8.04
7.94
7.94
7.92
7.92
7.91
7.91
7.89
7.89
7.60
7.59
7.59
7.58
7.57
7.56
7.56
7.39
7.39
7.37
7.37
7.33
7.33
7.31
7.29
7.27
7.26
7.16
7.15
7.14
7.14
7.14
4.67
4.45
4.44
3.69
3.49
3.48
3.47
3.46
3.34
3.07
2.69
2.68
2.23
2.00
MeO2C
OHC
OH
H
H
O
NBn
H
O
7g
1.00
1.06
2.21
3.39
2.10
3.23
1.16
2.21
1.12
3.31
1.10
1.00
1.17
1.14
1.23
1.29
10.5 10.0 9.5 9.0 8.5 8.0 7.5 7.0 6.5 6.0 5.5 5.0 4.5 4.0 3.5 3.0 2.5 2.0 1.5 1.0 0.5 0.0 -0.5 -1.
f1 (ppm)

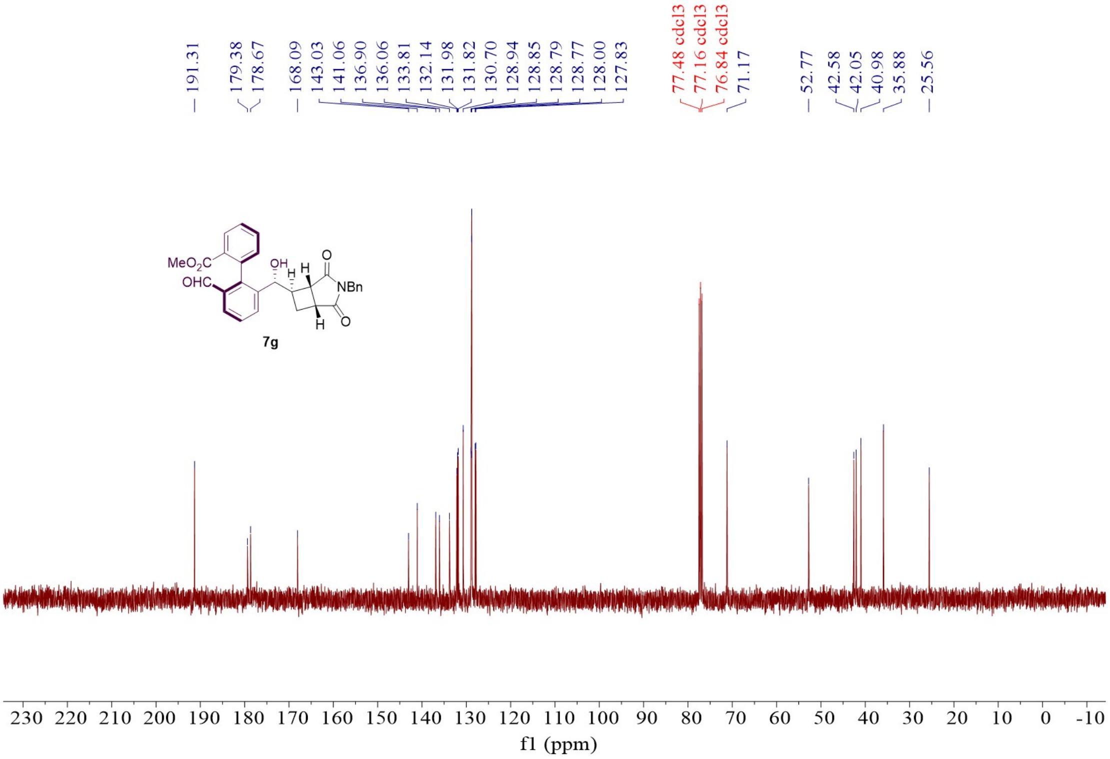
191.31
179.38
178.67
168.09
143.03
141.06
136.90
136.06
133.81
132.14
131.98
131.82
130.70
128.94
128.85
128.79
128.77
128.00
127.83
77.48 cdcl3
77.16 cdcl3
76.84 cdcl3
71.17
52.77
42.58
42.05
40.98
35.88
25.56
MeO2C
OHC
OH
H
H
O
NBn
H
O
7g
230 220 210 200 190 180 170 160 150 140 130 120 110 100 90 80 70 60 50 40 30 20 10 0 -10
f1 (ppm)

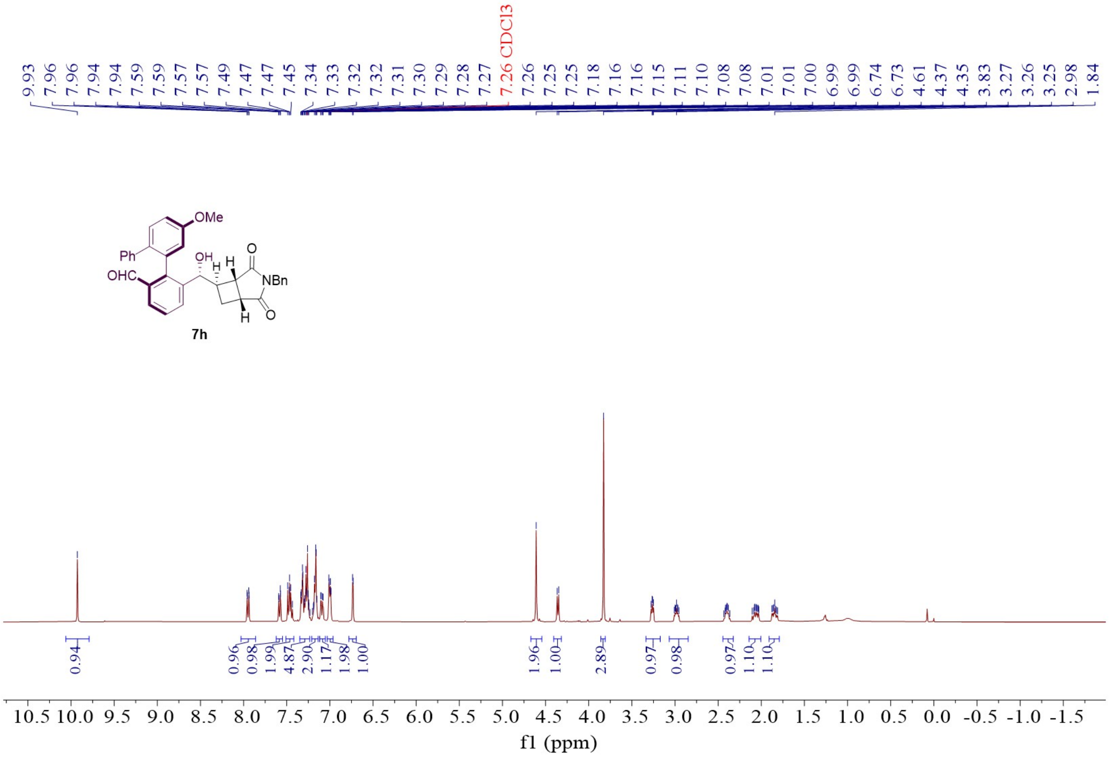

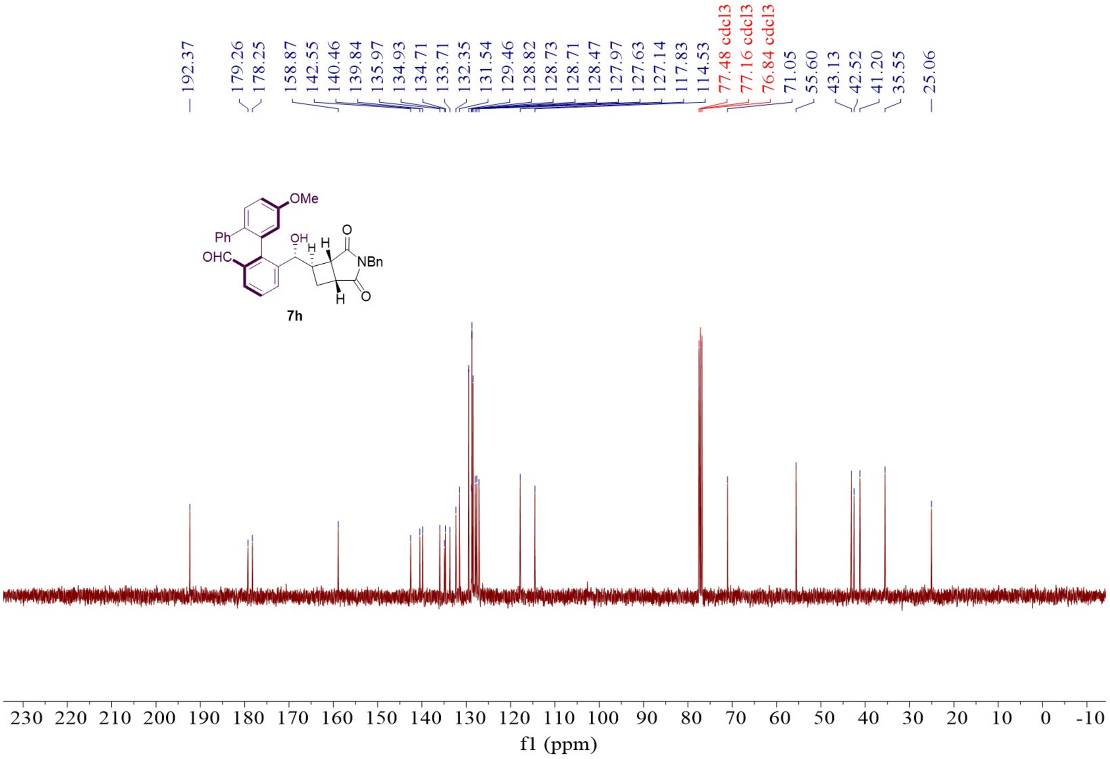

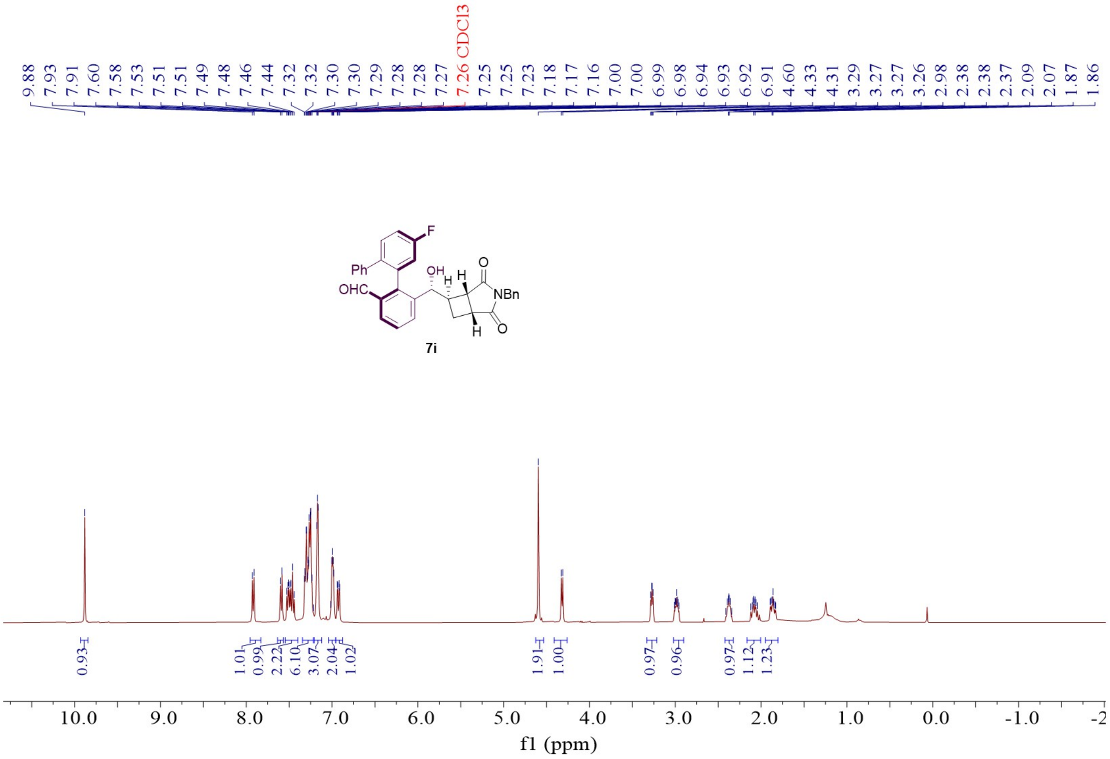
9.88 7.93 7.91 7.60 7.58 7.53 7.51 7.51 7.49 7.48 7.46 7.44 7.32 7.32 7.30 7.29 7.28 7.28 7.27 7.26 CDCl3 7.25 7.25 7.23 7.18 7.17 7.16 7.00 6.99 6.98 6.94 6.93 6.92 6.91 4.60 4.33 4.31 3.29 3.27 3.27 3.26 2.98 2.38 2.38 2.37 2.09 2.07 1.87 1.86
F
Ph
OHC
OH
H
O
NBn
H
O
7i
0.93 1.01 0.99 2.22 6.10 3.07 2.04 1.02 1.91 1.00 0.97 0.96 0.97 1.12 1.23
10.0 9.0 8.0 7.0 6.0 5.0 4.0 3.0 2.0 1.0 0.0 -1.0 -2
f1 (ppm)

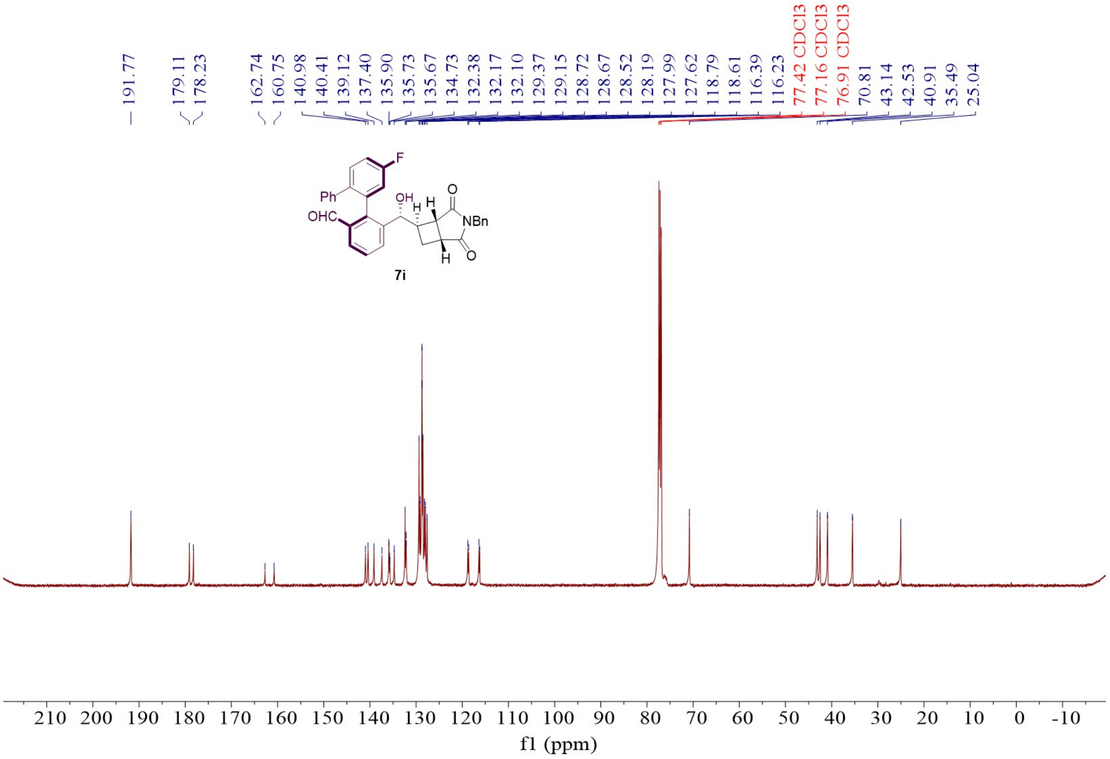
191.77 179.11 178.23 162.74 160.75 140.98 140.41 139.12 137.40 135.90 135.73 135.67 134.73 132.38 132.17 132.10 129.37 129.15 128.72 128.67 128.52 128.19 127.99 127.62 118.79 118.61 116.39 116.23 77.42 CDCl3 77.16 CDCl3 76.91 CDCl3 70.81 43.14 42.53 40.91 35.49 25.04
F
Ph
OHC
OH
H
O
NBn
H
O
7i
210 200 190 180 170 160 150 140 130 120 110 100 90 80 70 60 50 40 30 20 10 0 -10
f1 (ppm)

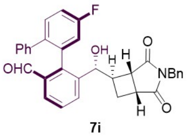
F
Ph
OHC
OH
H
H
O
NBn
H
O

7i

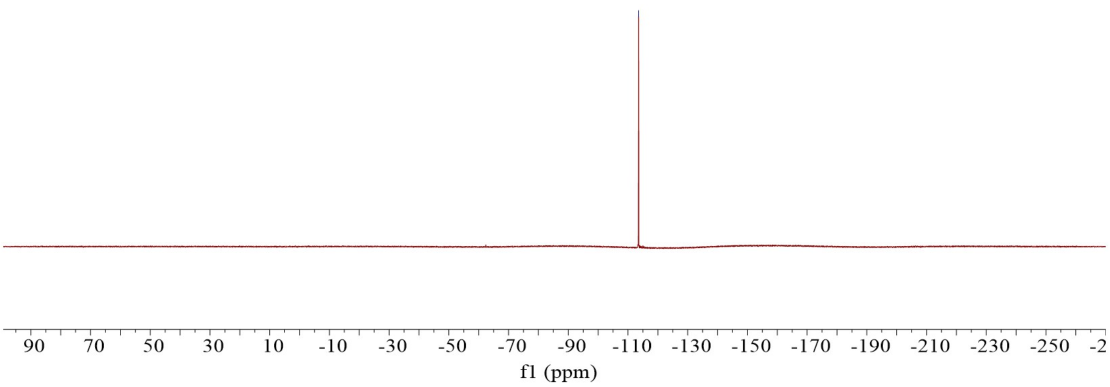
-113.57
90 70 50 30 10 -10 -30 -50 -70 -90 -110 -130 -150 -170 -190 -210 -230 -250
f1 (ppm)

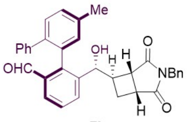
Me
Ph
OHC
OH
H
H
O
NBn
H
O

7j

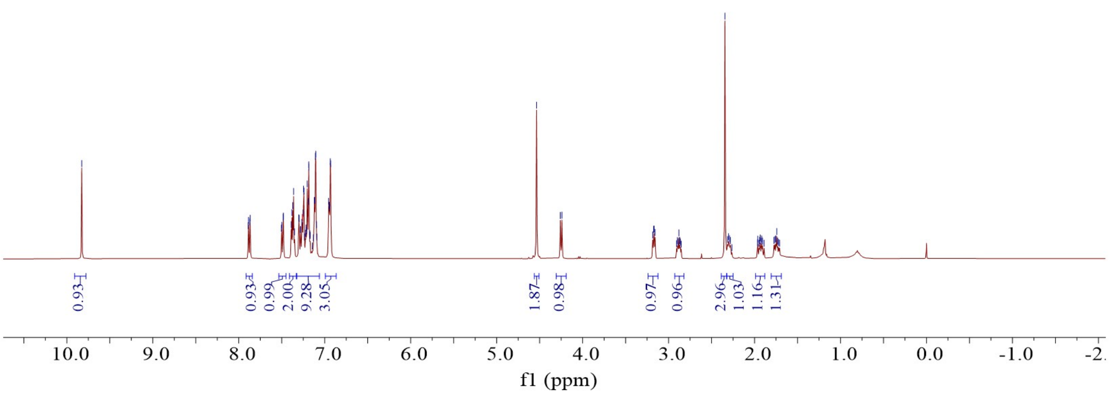
9.83 7.89 7.89 7.87 7.87 7.50 7.50 7.48 7.48 7.39 7.38 7.37 7.36 7.35 7.30 7.30 7.28 7.28 7.27 7.26 CDCl3 7.25 7.25 7.24 7.22 7.22 7.21 7.20 7.19 7.19 7.18 7.17 7.12 7.12 7.11 7.10 7.10 6.96 6.95 6.94 6.93 4.54 4.26 4.24 3.17 3.17 3.16 2.88 2.34 1.74
0.93 0.93 0.99 2.00 9.28 3.05 1.87 0.98 0.97 0.96 2.96 1.03 1.16 1.31
10.0 9.0 8.0 7.0 6.0 5.0 4.0 3.0 2.0 1.0 0.0 -1.0
f1 (ppm)

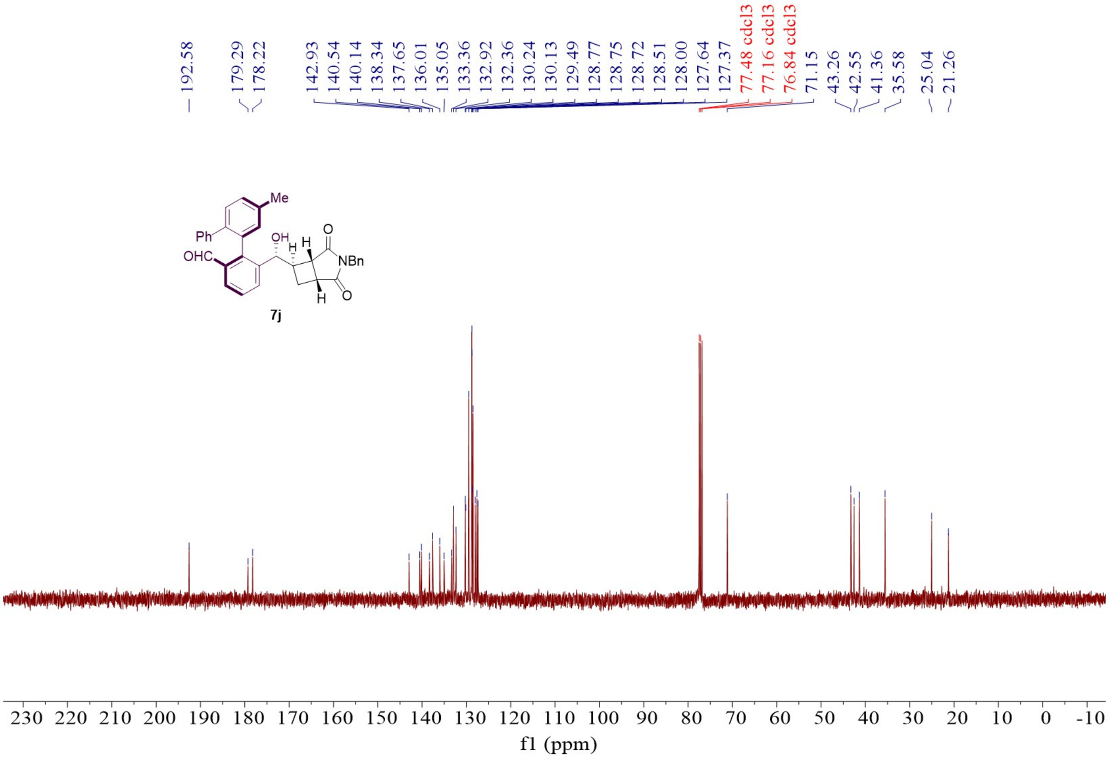
192.58
179.29
178.22
142.93
140.54
140.14
138.34
137.65
136.01
135.05
133.36
132.92
132.36
130.24
130.13
129.49
128.77
128.75
128.72
128.51
128.00
127.64
127.37
77.48 cdcl3
77.16 cdcl3
76.84 cdcl3
71.15
43.26
42.55
41.36
35.58
25.04
21.26
Me
Ph
OHC
OH
H
O
NBn
H
O
7j
230 220 210 200 190 180 170 160 150 140 130 120 110 100 90 80 70 60 50 40 30 20 10 0 -10
f1 (ppm)

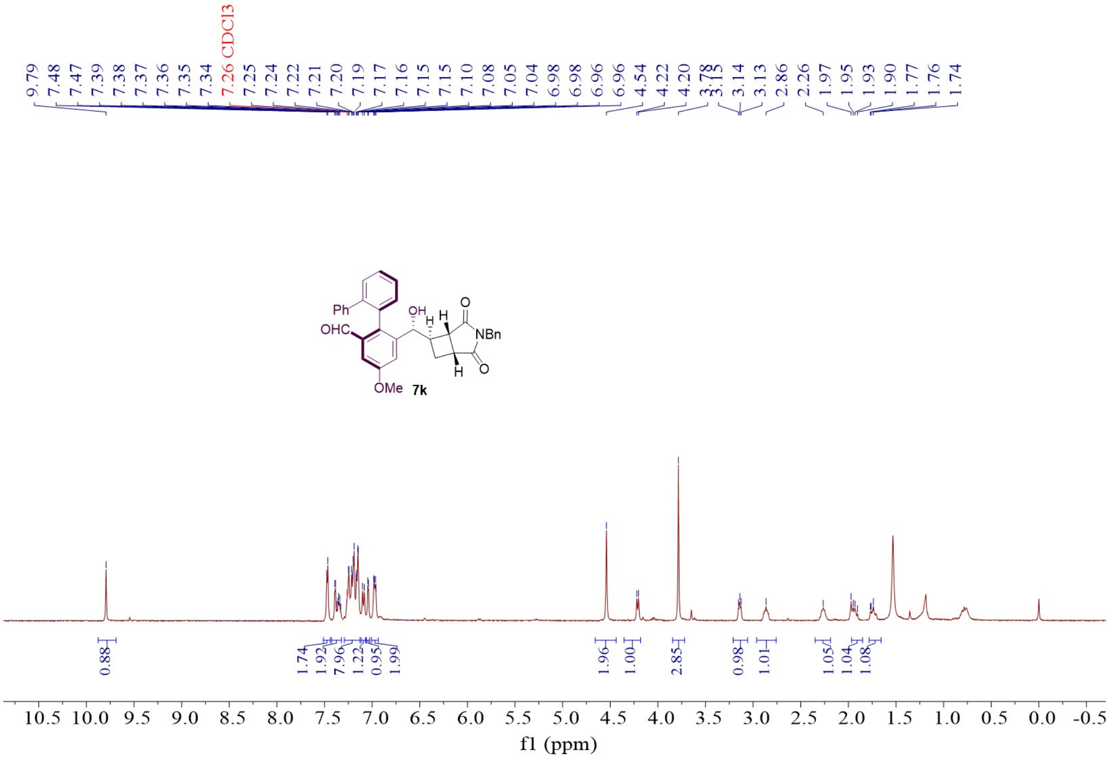
9.79
7.48
7.47
7.39
7.38
7.37
7.36
7.35
7.34
7.26 CDCl3
7.25
7.24
7.22
7.21
7.20
7.19
7.17
7.16
7.15
7.15
7.10
7.08
7.05
7.04
6.98
6.96
6.96
4.54
4.22
4.20
3.78
3.15
3.14
3.13
2.86
2.26
1.97
1.95
1.93
1.90
1.77
1.76
1.74
Ph
OHC
OH
H
O
NBn
H
O
OMe
7k
0.88
1.74
1.92
7.96
1.22
0.95
1.99
1.96
1.00
2.85
0.98
1.01
1.05
1.04
1.08
10.5 10.0 9.5 9.0 8.5 8.0 7.5 7.0 6.5 6.0 5.5 5.0 4.5 4.0 3.5 3.0 2.5 2.0 1.5 1.0 0.5 0.0 -0.5
f1 (ppm)

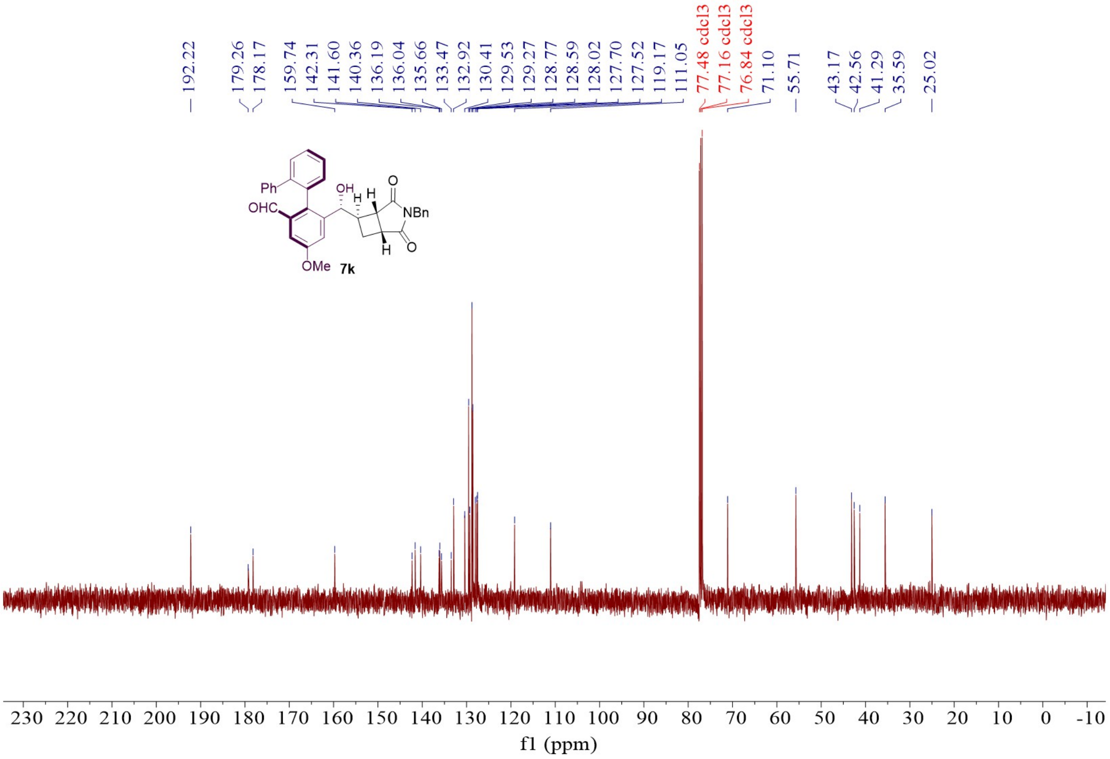
192.22
179.26
178.17
159.74
142.31
141.60
140.36
136.19
136.04
135.66
133.47
132.92
130.41
129.53
129.27
128.77
128.59
128.02
127.70
127.52
119.17
111.05
77.48 cdcl3
77.16 cdcl3
76.84 cdcl3
71.10
55.71
43.17
42.56
41.29
35.59
25.02
Ph
OHC
OH
H
O
NBn
H
O
OMe
7k
230 220 210 200 190 180 170 160 150 140 130 120 110 100 90 80 70 60 50 40 30 20 10 0 -10
f1 (ppm)

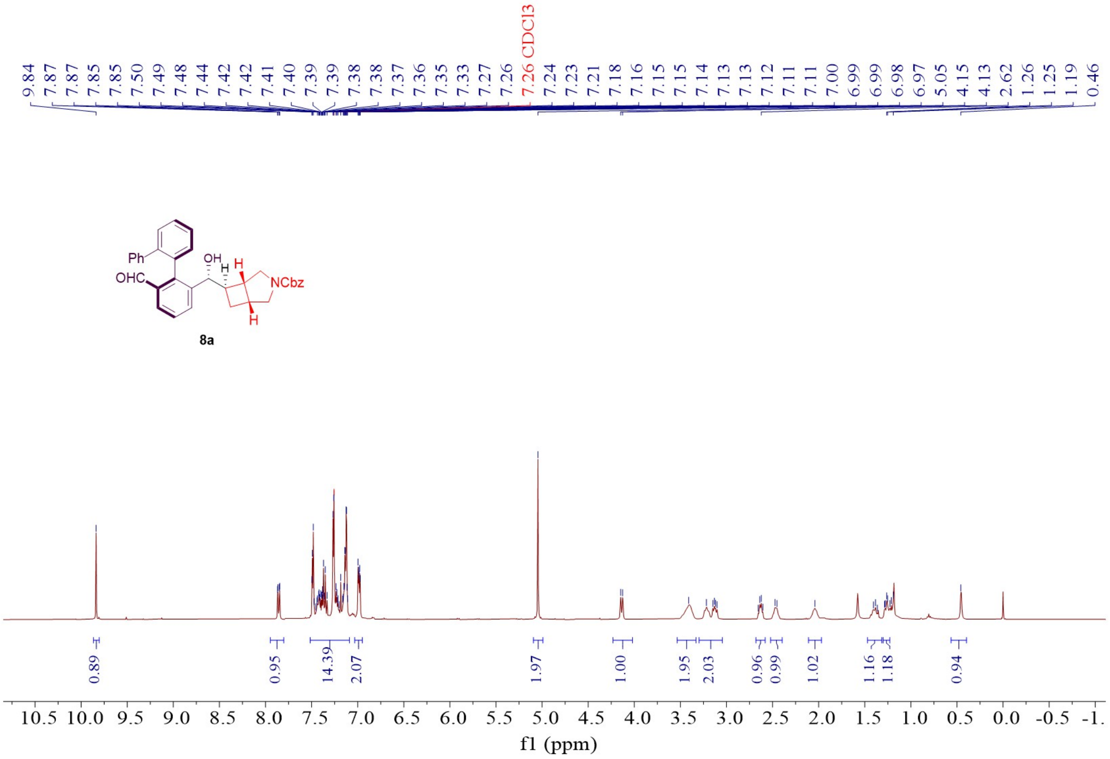
9.84
7.87
7.87
7.85
7.85
7.50
7.49
7.48
7.44
7.42
7.42
7.41
7.40
7.39
7.39
7.38
7.38
7.37
7.36
7.35
7.33
7.27
7.26
7.26 CDCl3
7.24
7.23
7.21
7.18
7.16
7.15
7.15
7.14
7.13
7.13
7.12
7.11
7.11
7.00
6.99
6.99
6.98
6.97
5.05
4.15
4.13
2.62
1.26
1.25
1.19
0.46
Ph
OHC
OH
H
H
NCbz
H
8a
0.89
0.95
14.39
2.07
1.97
1.00
1.95
2.03
0.96
0.99
1.02
1.16
1.18
0.94
10.5 10.0 9.5 9.0 8.5 8.0 7.5 7.0 6.5 6.0 5.5 5.0 4.5 4.0 3.5 3.0 2.5 2.0 1.5 1.0 0.5 0.0 -0.5 -1.
f1 (ppm)

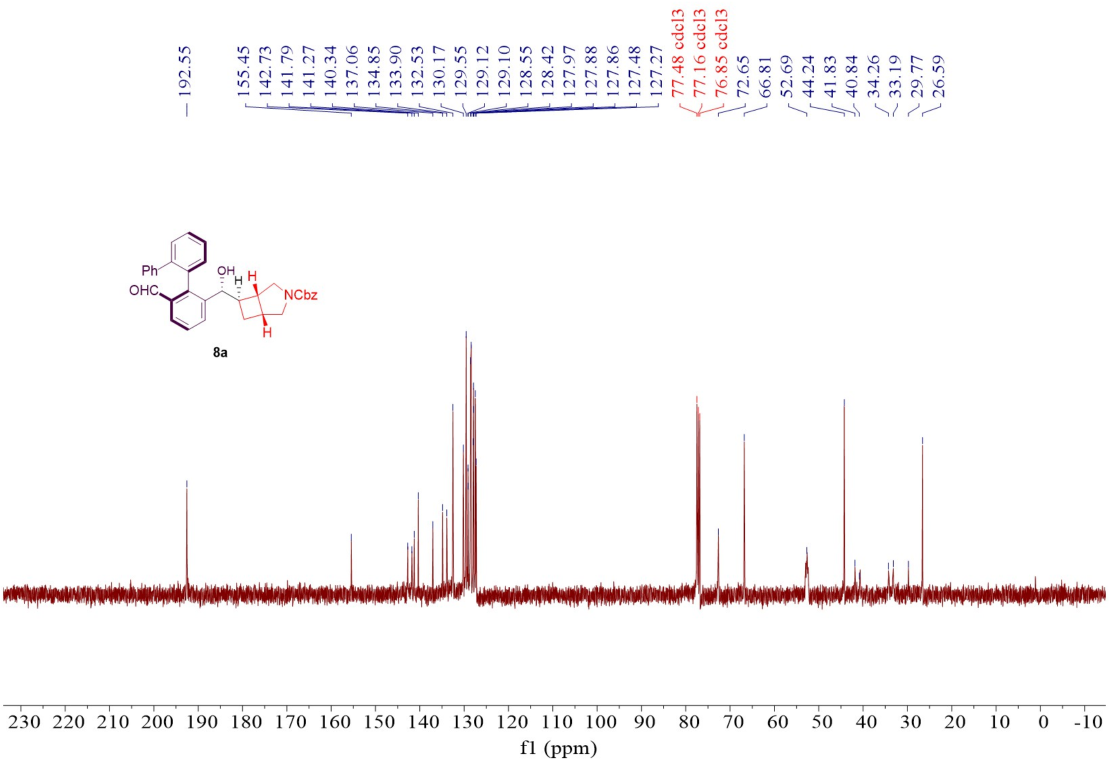
192.55
155.45
142.73
141.79
141.27
140.34
137.06
134.85
133.90
132.53
130.17
129.55
129.12
129.10
128.55
128.42
127.97
127.88
127.86
127.48
127.27
77.48 cdcl3
77.16 cdcl3
76.85 cdcl3
72.65
66.81
52.69
44.24
41.83
40.84
34.26
33.19
29.77
26.59
Ph
OHC
OH
H
H
H
NCbz
8a
230 220 210 200 190 180 170 160 150 140 130 120 110 100 90 80 70 60 50 40 30 20 10 0 -10
f1 (ppm)

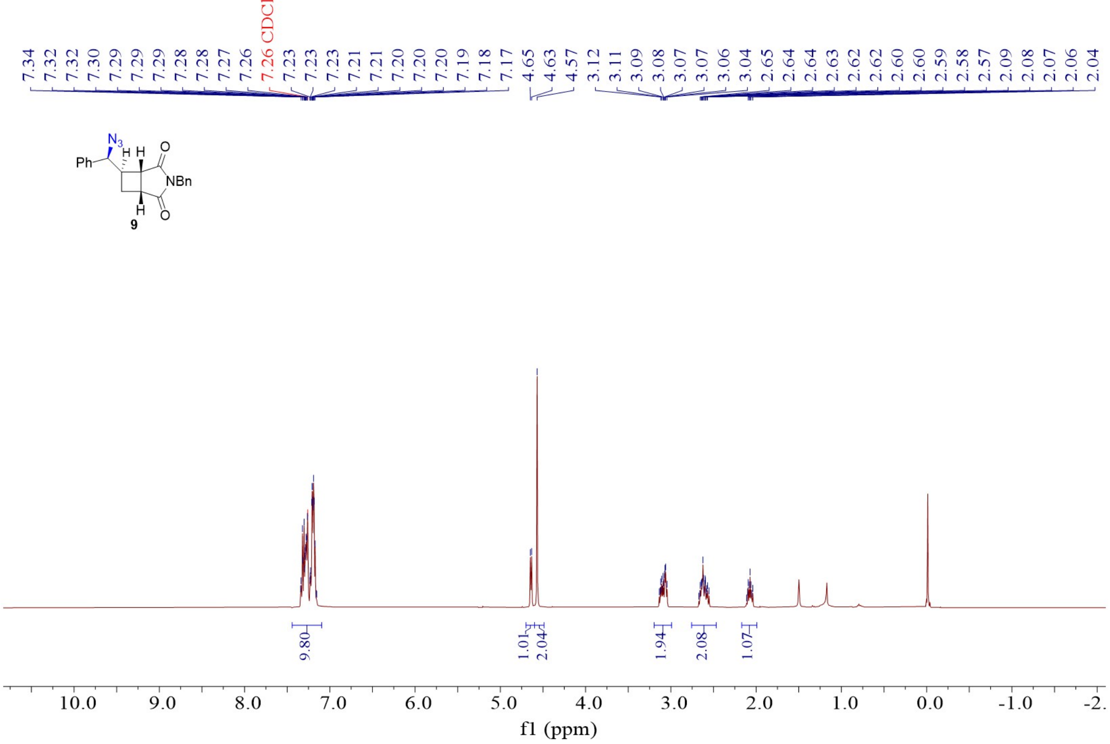
7.34
7.32
7.32
7.30
7.29
7.29
7.28
7.28
7.27
7.26
7.26 CDCl3
7.23
7.23
7.21
7.21
7.20
7.20
7.19
7.18
7.17
4.65
4.63
4.57
3.12
3.11
3.09
3.08
3.07
3.07
3.06
3.04
2.65
2.64
2.64
2.63
2.62
2.60
2.60
2.59
2.58
2.57
2.09
2.08
2.07
2.06
2.04
N_3
Ph
H
H
O
O
NBn
9
9.80
1.01
2.04
1.94
2.08
1.07
10.0 9.0 8.0 7.0 6.0 5.0 4.0 3.0 2.0 1.0 0.0 -1.0 -2.
f1 (ppm)

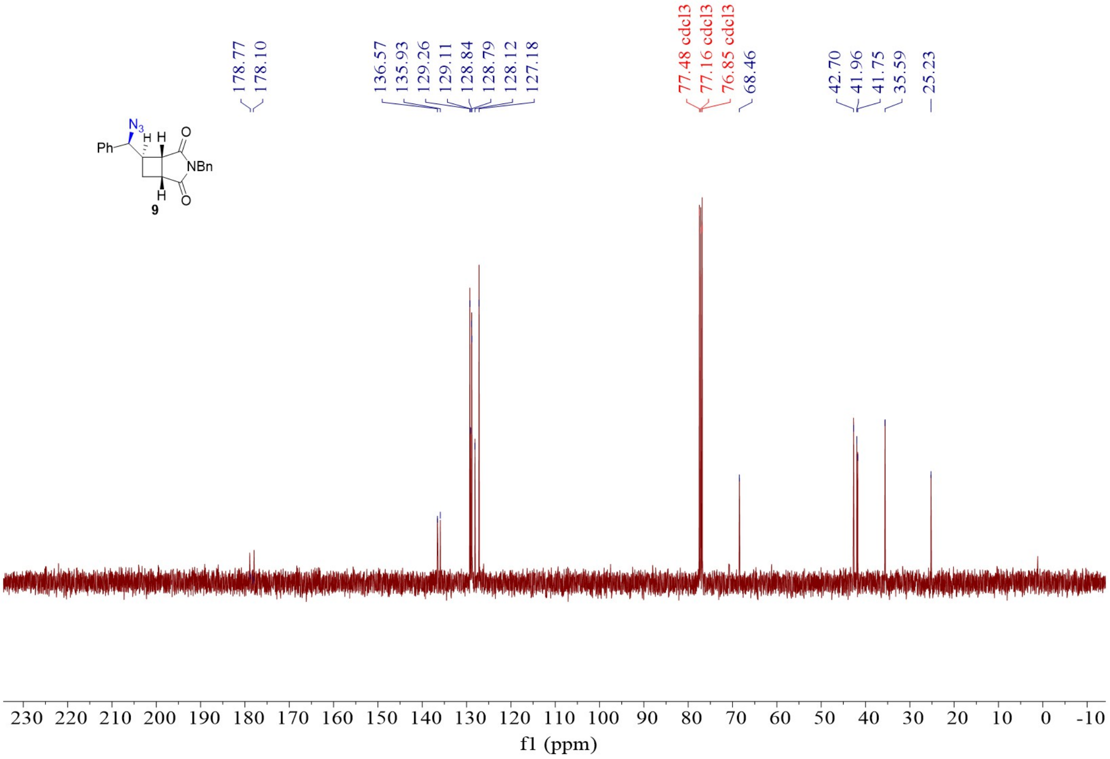

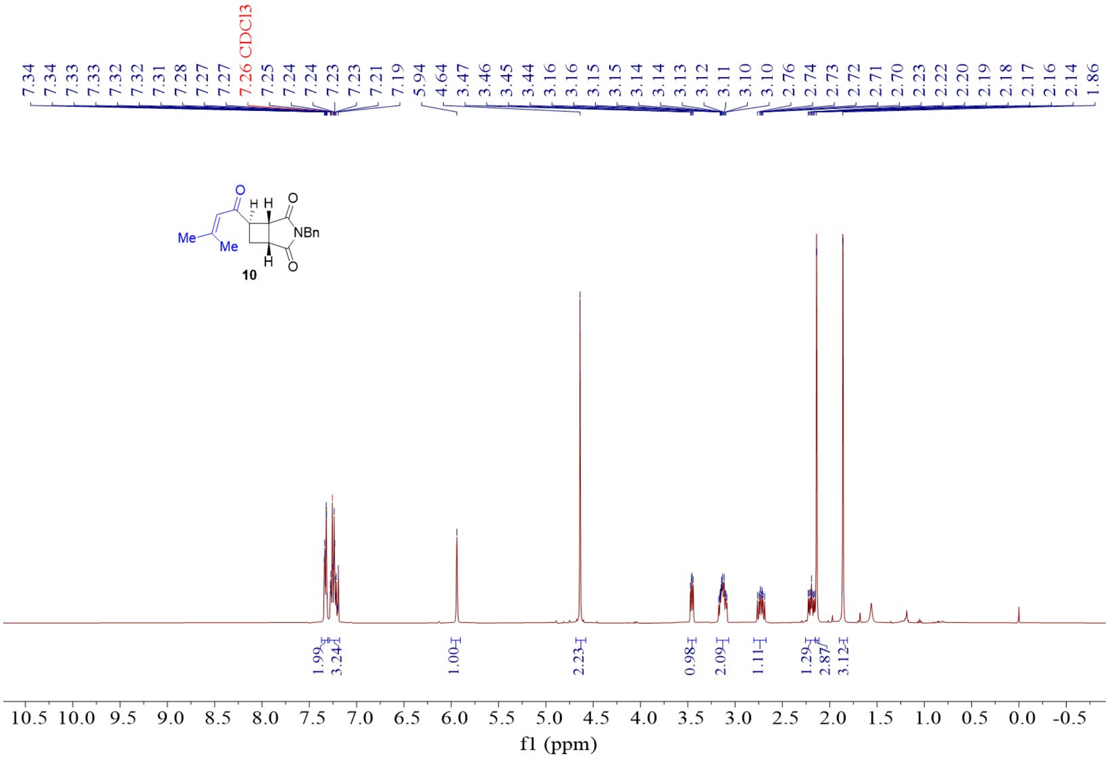

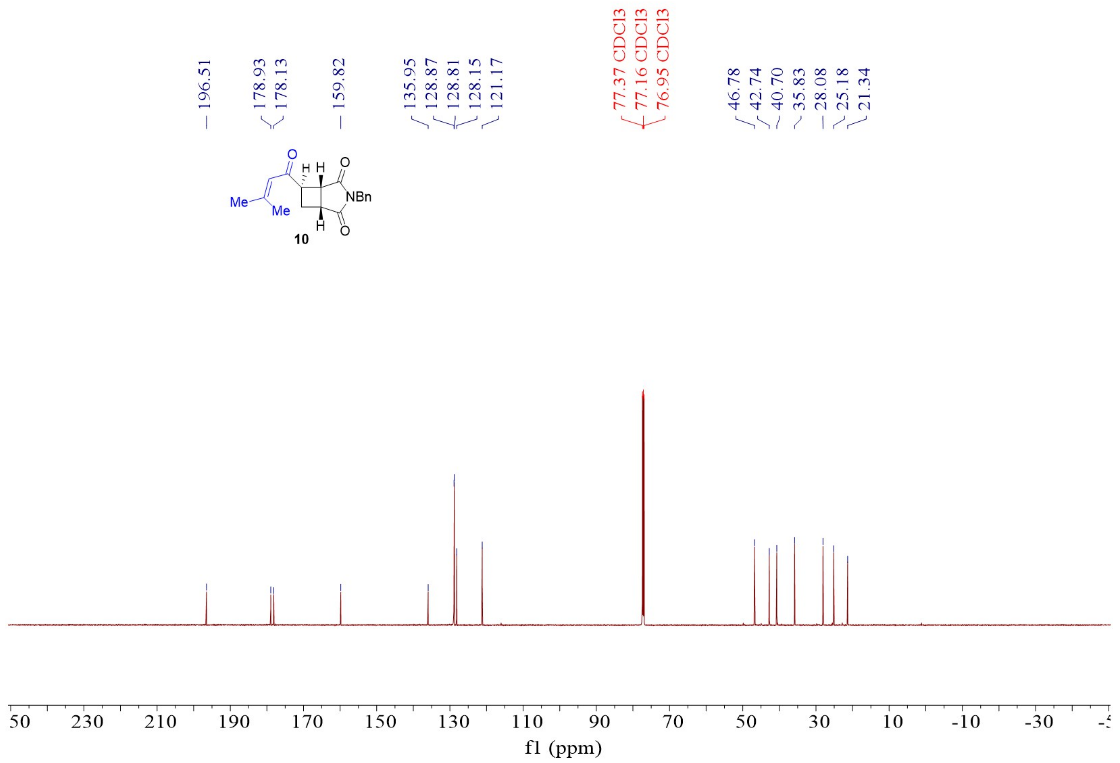

196.51
178.93
178.13
159.82
135.95
128.87
128.81
128.15
121.17
77.37 CDCl3
77.16 CDCl3
76.95 CDCl3
46.78
42.74
40.70
35.83
28.08
25.18
21.34
Me
Me
NBn
10
f1 (ppm)

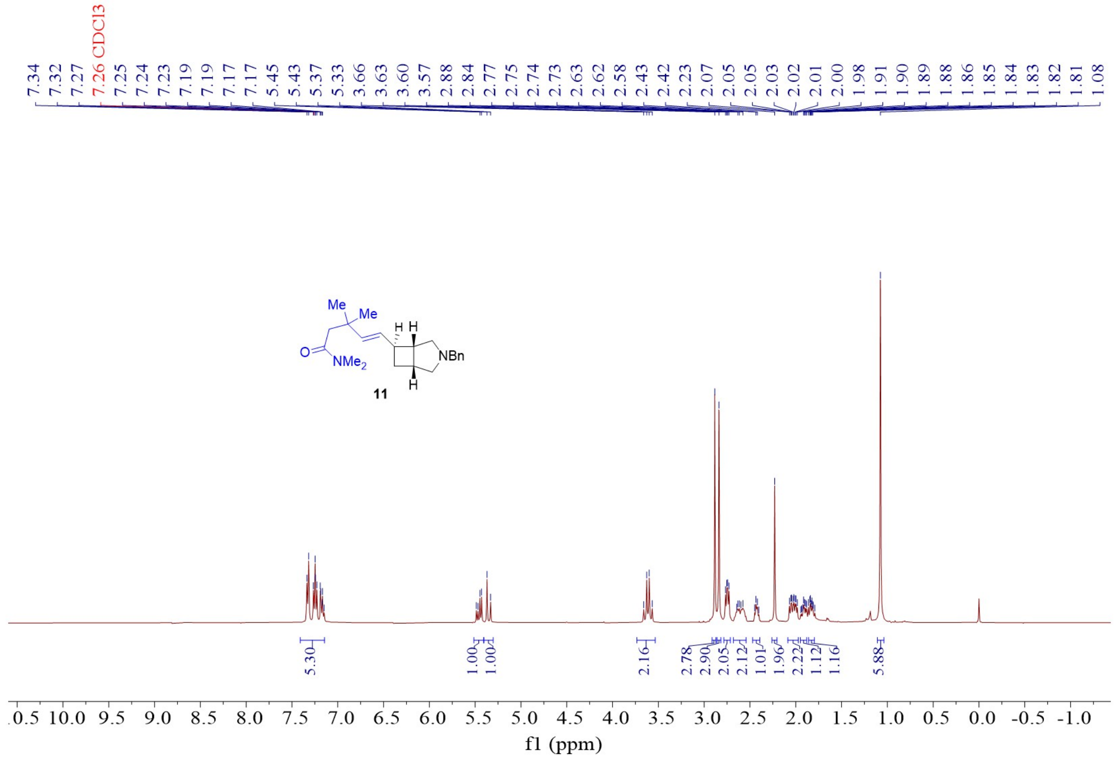

7.34
7.32
7.27
7.26 CDCl3
7.25
7.24
7.23
7.19
7.19
7.17
7.17
5.45
5.43
5.37
5.33
3.66
3.63
3.60
3.57
2.88
2.84
2.77
2.75
2.74
2.73
2.63
2.62
2.58
2.43
2.42
2.23
2.07
2.05
2.05
2.03
2.02
2.01
2.00
1.98
1.91
1.90
1.89
1.88
1.86
1.85
1.84
1.83
1.82
1.81
1.08
Me
Me
NMe2
NBn
11
5.30
1.00
1.00
2.16
2.78
2.90
2.05
2.12
1.01
1.96
2.22
1.12
1.16
5.88
f1 (ppm)

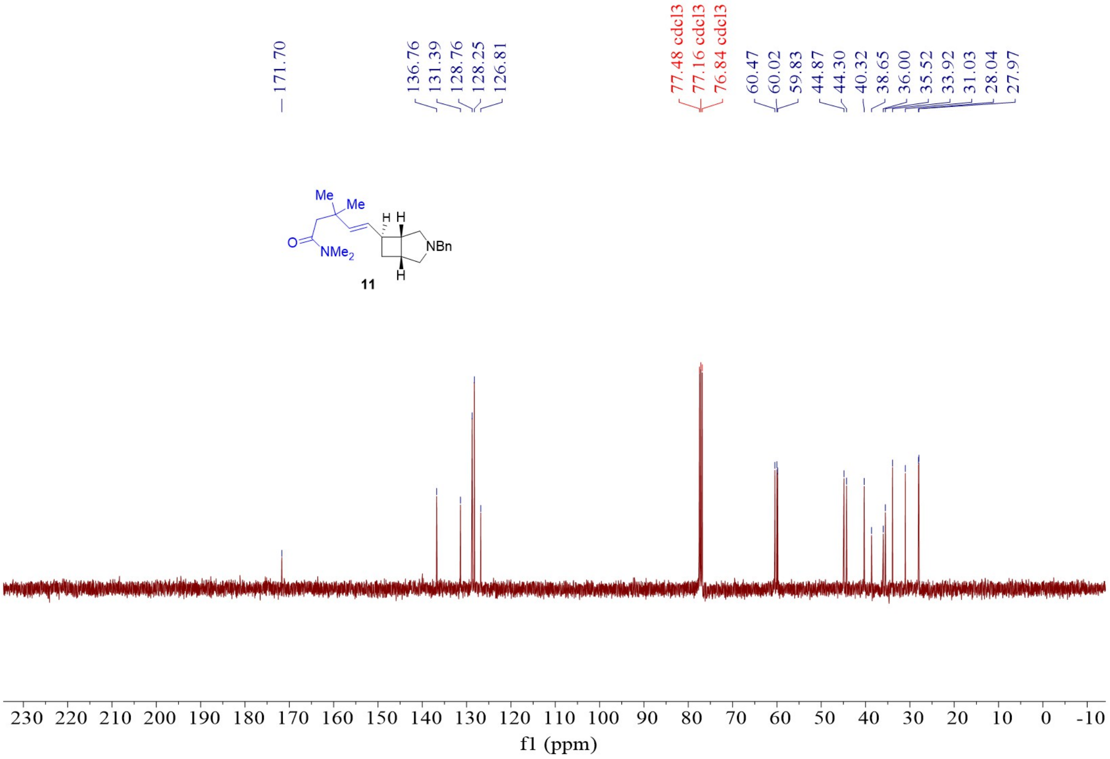
171.70
136.76
131.39
128.76
128.25
126.81
77.48 cdcl3
77.16 cdcl3
76.84 cdcl3
60.47
60.02
59.83
44.87
44.30
40.32
38.65
36.00
35.52
33.92
31.03
28.04
27.97
Me
Me
O
NMe2
H
H
H
NBn
11
230 220 210 200 190 180 170 160 150 140 130 120 110 100 90 80 70 60 50 40 30 20 10 0 -10
f1 (ppm)

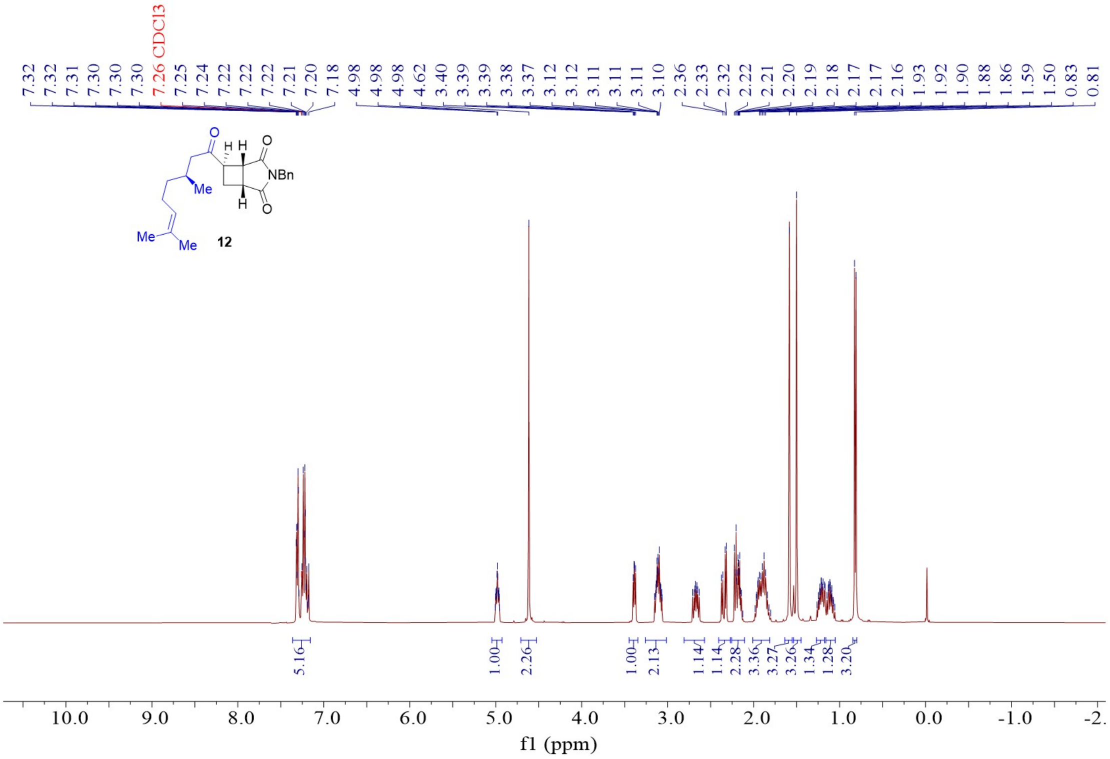
7.32
7.32
7.31
7.30
7.30
7.30
7.26 CDCl3
7.25
7.24
7.22
7.22
7.22
7.21
7.20
7.18
4.98
4.98
4.62
3.40
3.39
3.39
3.38
3.37
3.12
3.12
3.11
3.11
3.11
3.10
2.36
2.33
2.32
2.22
2.21
2.20
2.19
2.18
2.17
2.16
1.93
1.92
1.90
1.88
1.86
1.59
1.50
0.83
0.81
O
H
H
O
NBn
Me
H
O
Me
Me
12
5.16
1.00
2.26
1.00
2.13
1.14
1.14
2.28
3.36
3.27
3.26
1.34
1.28
3.20
10.0 9.0 8.0 7.0 6.0 5.0 4.0 3.0 2.0 1.0 0.0 -1.0 -2.
f1 (ppm)

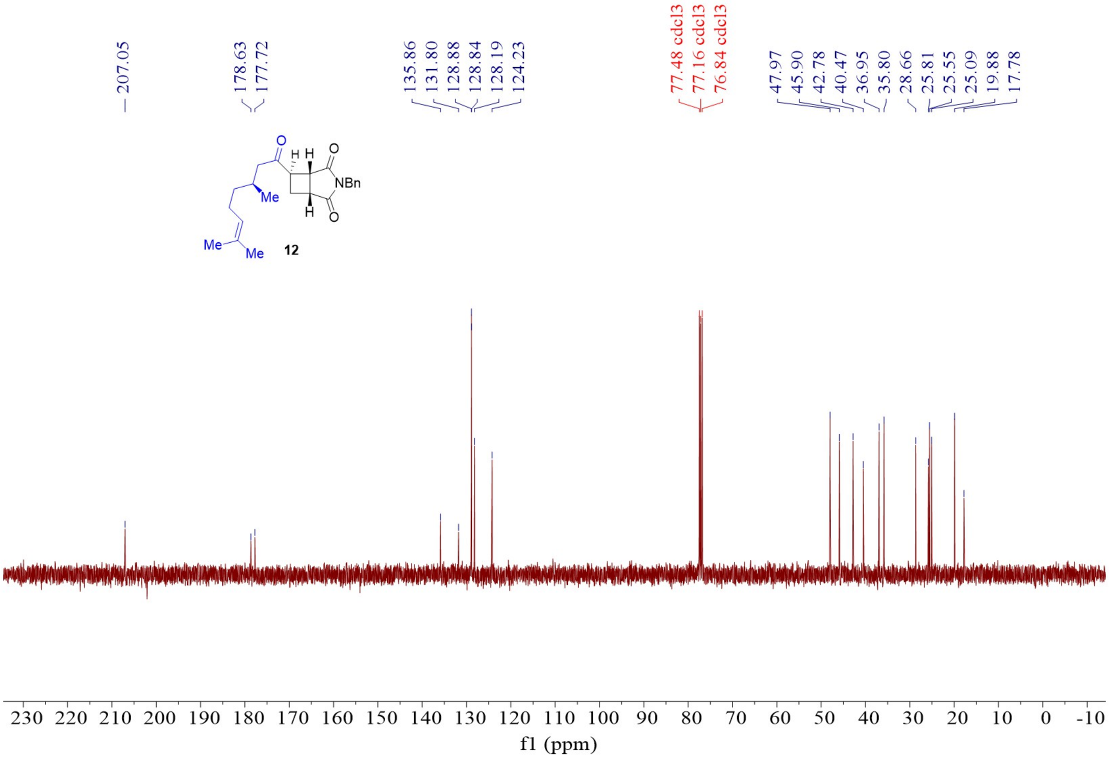
207.05
178.63
177.72
135.86
131.80
128.88
128.84
128.19
124.23
77.48 cdcl3
77.16 cdcl3
76.84 cdcl3
47.97
45.90
42.78
40.47
36.95
35.80
28.66
25.81
25.55
25.09
19.88
17.78
O
H H
O
NBn
Me
H
O
Me
Me
12
230 220 210 200 190 180 170 160 150 140 130 120 110 100 90 80 70 60 50 40 30 20 10 0 -10
f1 (ppm)

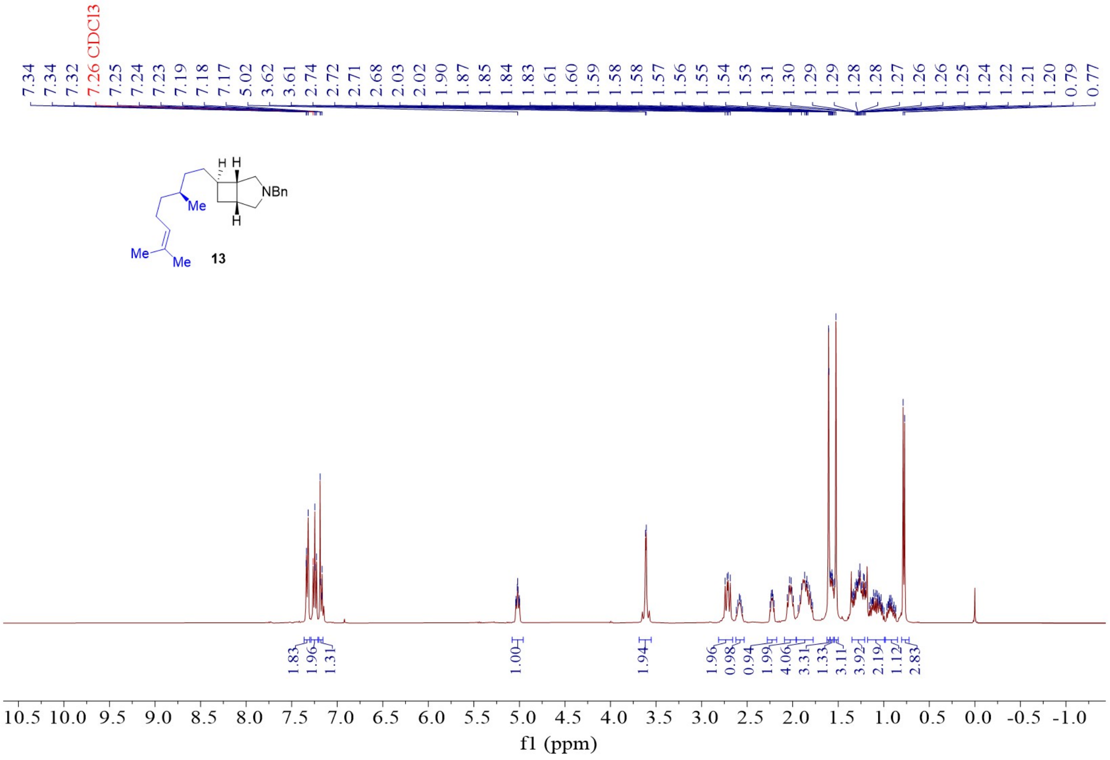
7.34
7.34
7.32
7.26 CDCl3
7.25
7.24
7.23
7.19
7.18
7.17
5.02
3.62
3.61
2.74
2.72
2.71
2.68
2.03
2.02
1.90
1.87
1.85
1.84
1.83
1.61
1.60
1.59
1.58
1.58
1.57
1.56
1.55
1.54
1.53
1.31
1.30
1.29
1.29
1.28
1.28
1.27
1.26
1.26
1.25
1.24
1.22
1.21
1.20
0.79
0.77
H H
NBn
Me
H
Me
Me
13
1.83
1.96
1.31
1.00
1.94
1.96
0.98
0.94
1.99
4.06
3.31
1.33
3.11
3.92
2.19
1.12
2.83
10.5 10.0 9.5 9.0 8.5 8.0 7.5 7.0 6.5 6.0 5.5 5.0 4.5 4.0 3.5 3.0 2.5 2.0 1.5 1.0 0.5 0.0 -0.5 -1.0
f1 (ppm)

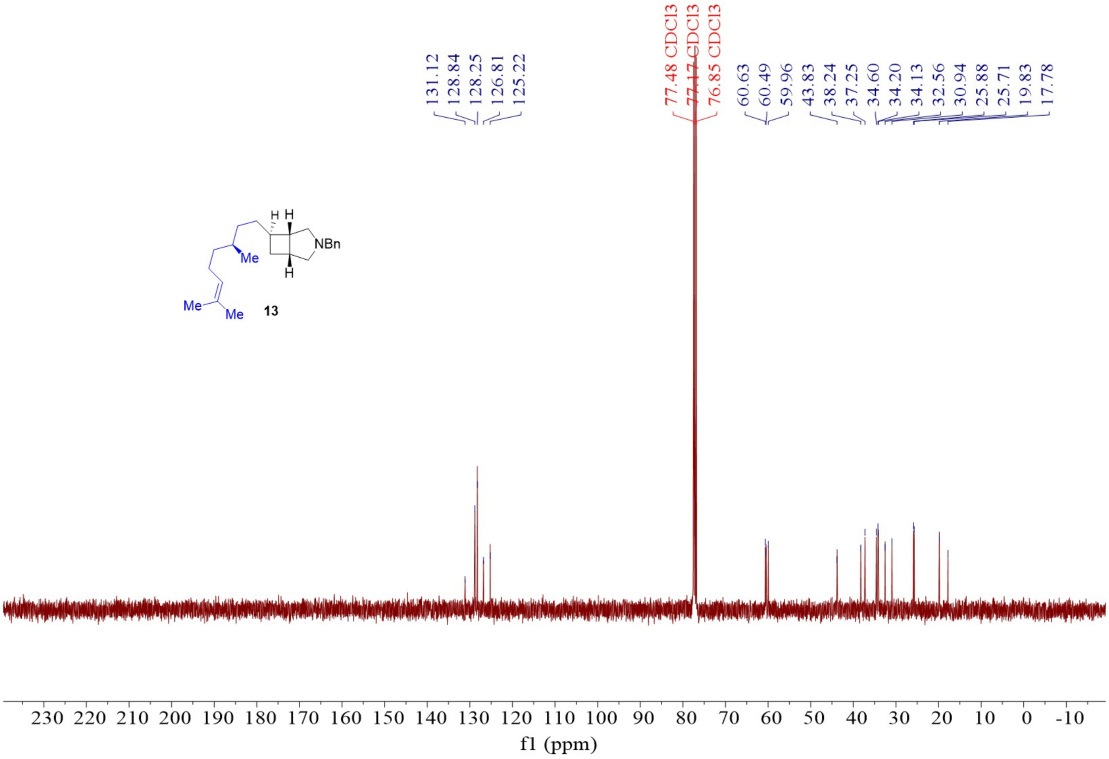
131.12
128.84
128.25
126.81
125.22
77.48 CDCl3
77.17 CDCl3
76.85 CDCl3
60.63
60.49
59.96
43.83
38.24
37.25
34.60
34.20
34.13
32.56
30.94
25.88
25.71
19.83
17.78
H
H
NBn
H
Me
Me
Me
13
230 220 210 200 190 180 170 160 150 140 130 120 110 100 90 80 70 60 50 40 30 20 10 0 -10
f1 (ppm)

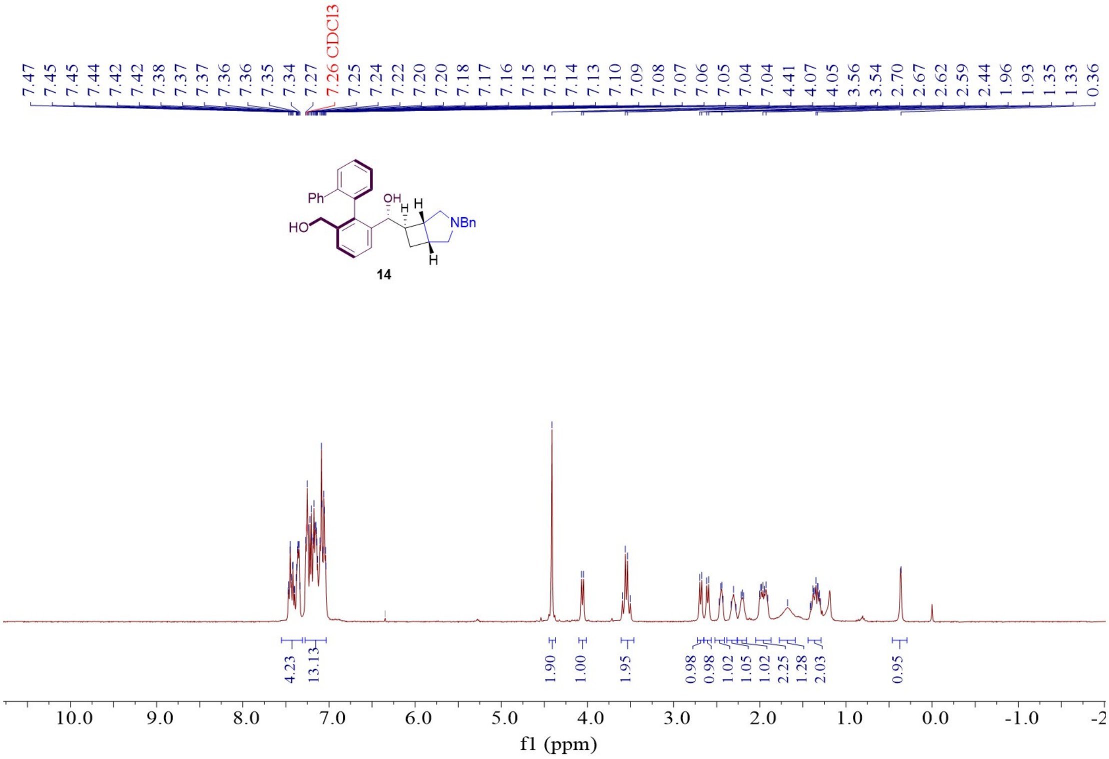
7.47
7.45
7.45
7.44
7.42
7.42
7.38
7.37
7.37
7.36
7.36
7.35
7.34
7.27
7.26 CDCl3
7.25
7.24
7.22
7.20
7.20
7.18
7.17
7.16
7.15
7.15
7.14
7.13
7.10
7.09
7.08
7.07
7.06
7.05
7.04
7.04
4.41
4.07
4.05
3.56
3.54
2.70
2.67
2.62
2.59
2.44
1.96
1.93
1.35
1.33
0.36
Ph
OH
H
H
HO
NBn
H
14
4.23
13.13
1.90
1.00
1.95
0.98
0.98
1.02
1.05
1.02
2.25
1.28
2.03
0.95
10.0 9.0 8.0 7.0 6.0 5.0 4.0 3.0 2.0 1.0 0.0 -1.0 -2
f1 (ppm)

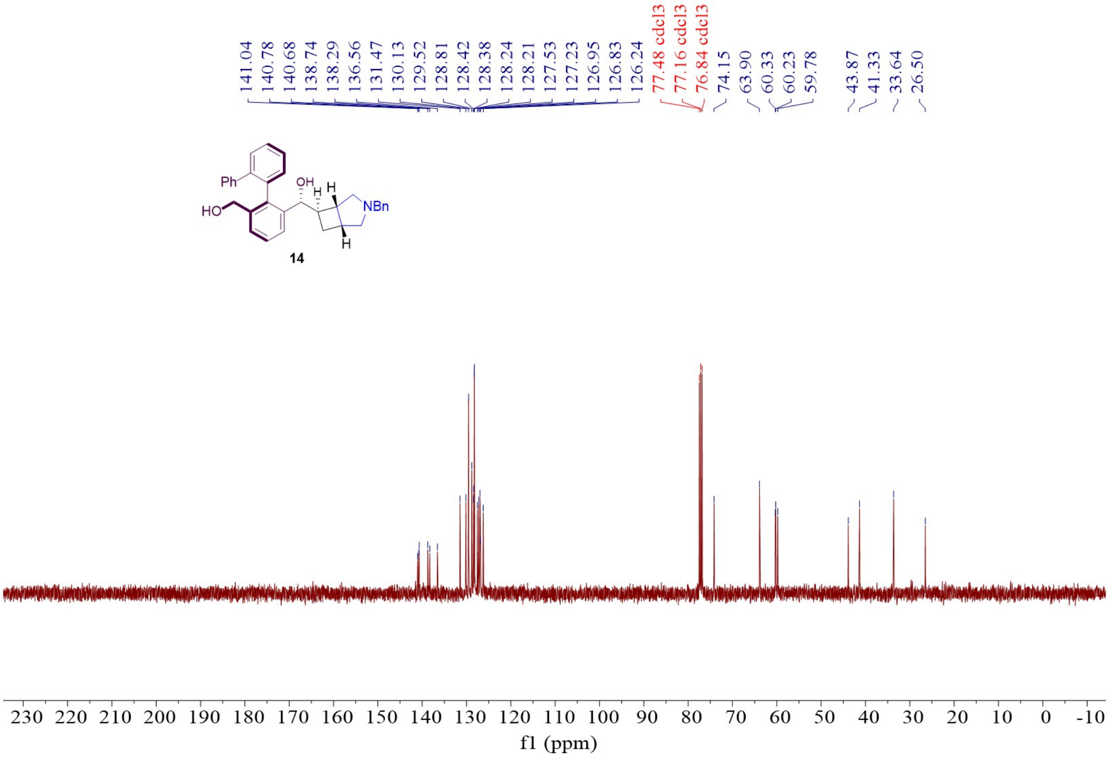
141.04
140.78
140.68
138.74
138.29
136.56
131.47
130.13
129.52
128.81
128.42
128.38
128.24
128.21
127.53
127.23
126.95
126.83
126.24
77.48 cdcl3
77.16 cdcl3
76.84 cdcl3
74.15
63.90
60.33
60.23
59.78
43.87
41.33
33.64
26.50
Ph
HO
OH
H
H
NBn
H
14
230 220 210 200 190 180 170 160 150 140 130 120 110 100 90 80 70 60 50 40 30 20 10 0 -10
f1 (ppm)

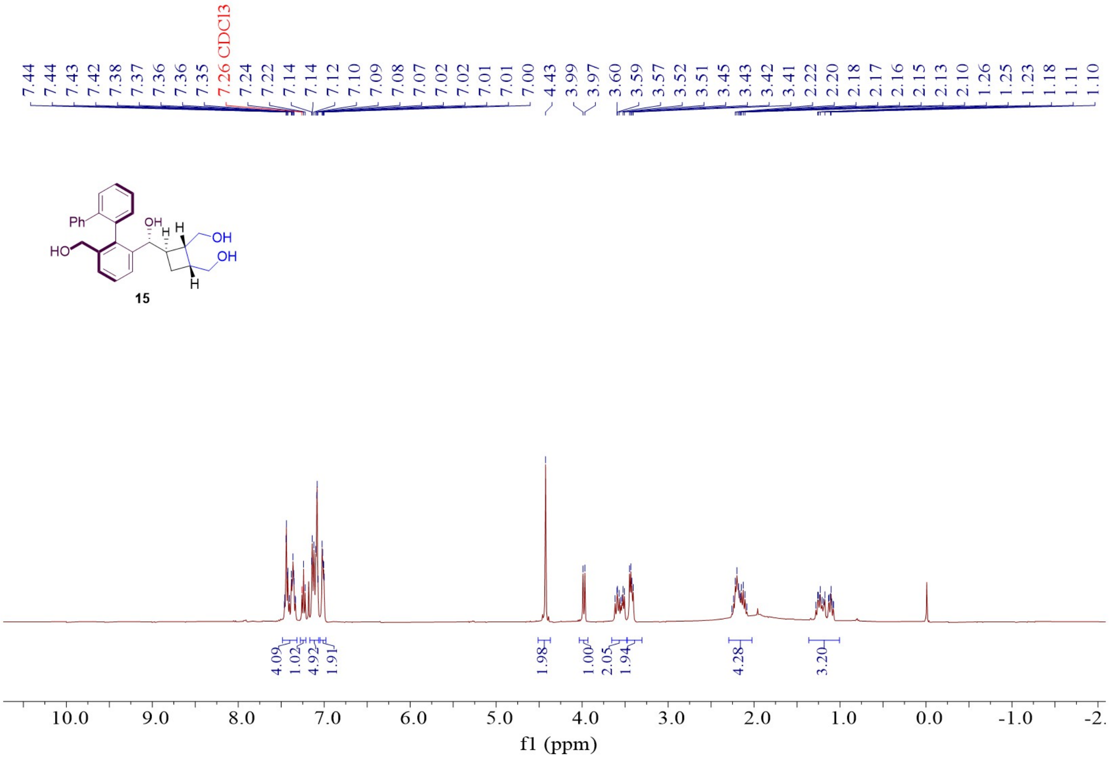
7.44
7.44
7.43
7.42
7.38
7.37
7.36
7.36
7.35
7.26 CDCl3
7.24
7.22
7.14
7.14
7.12
7.10
7.09
7.08
7.07
7.02
7.02
7.01
7.01
7.00
4.43
3.99
3.97
3.60
3.59
3.57
3.52
3.51
3.45
3.43
3.42
3.41
2.22
2.20
2.18
2.17
2.16
2.15
2.13
2.10
1.26
1.25
1.23
1.18
1.11
1.10
Ph
HO
OH
H
H
OH
OH
H
15
4.09
1.02
4.92
1.91
1.98
1.00
2.05
1.94
4.28
3.20
10.0 9.0 8.0 7.0 6.0 5.0 4.0 3.0 2.0 1.0 0.0 -1.0 -2.
f1 (ppm)

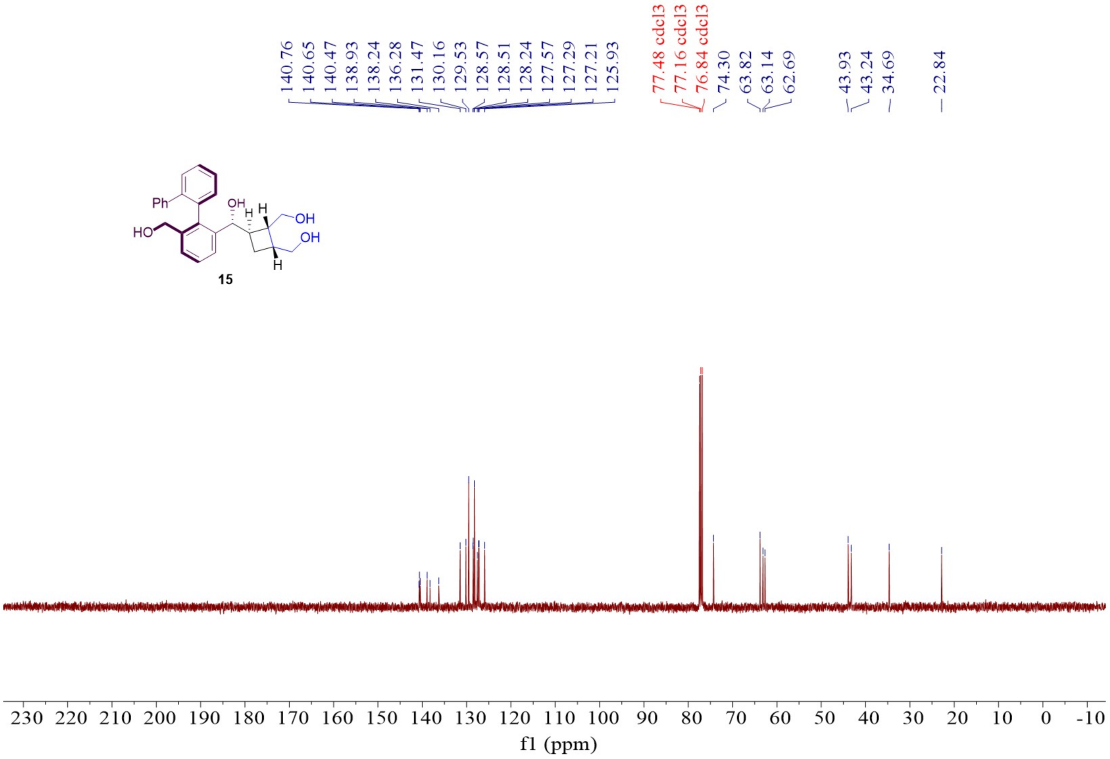
140.76
140.65
140.47
138.93
138.24
136.28
131.47
130.16
129.53
128.57
128.51
128.24
127.57
127.29
127.21
125.93
77.48 cdcl3
77.16 cdcl3
76.84 cdcl3
74.30
63.82
63.14
62.69
43.93
43.24
34.69
22.84
Ph
HO
OH
H
15
f1 (ppm)

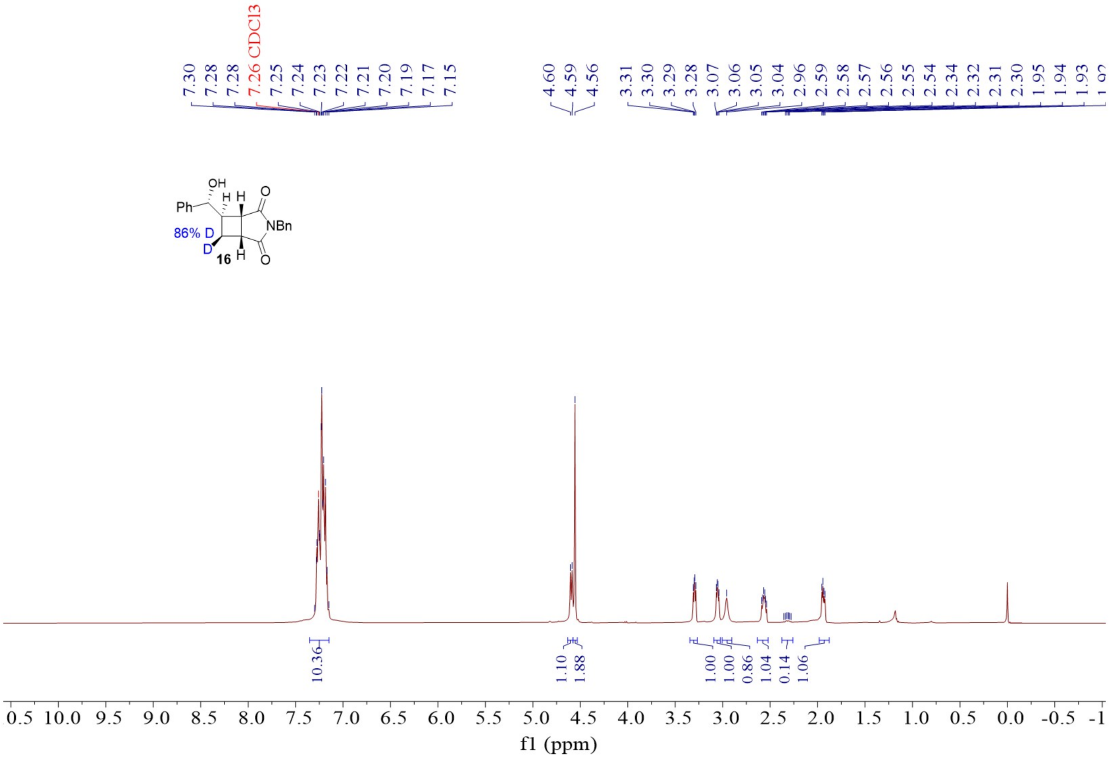
7.30
7.28
7.28
7.26 CDCl3
7.25
7.24
7.23
7.22
7.21
7.20
7.19
7.17
7.15
4.60
4.59
4.56
3.31
3.30
3.29
3.28
3.07
3.06
3.05
3.04
2.96
2.59
2.58
2.57
2.56
2.55
2.54
2.34
2.32
2.31
2.30
1.95
1.94
1.93
OH
H
O
Ph
NBn
86% D
D
16
10.36
1.10
1.88
1.00
1.00
0.86
1.04
0.14
1.06
f1 (ppm)

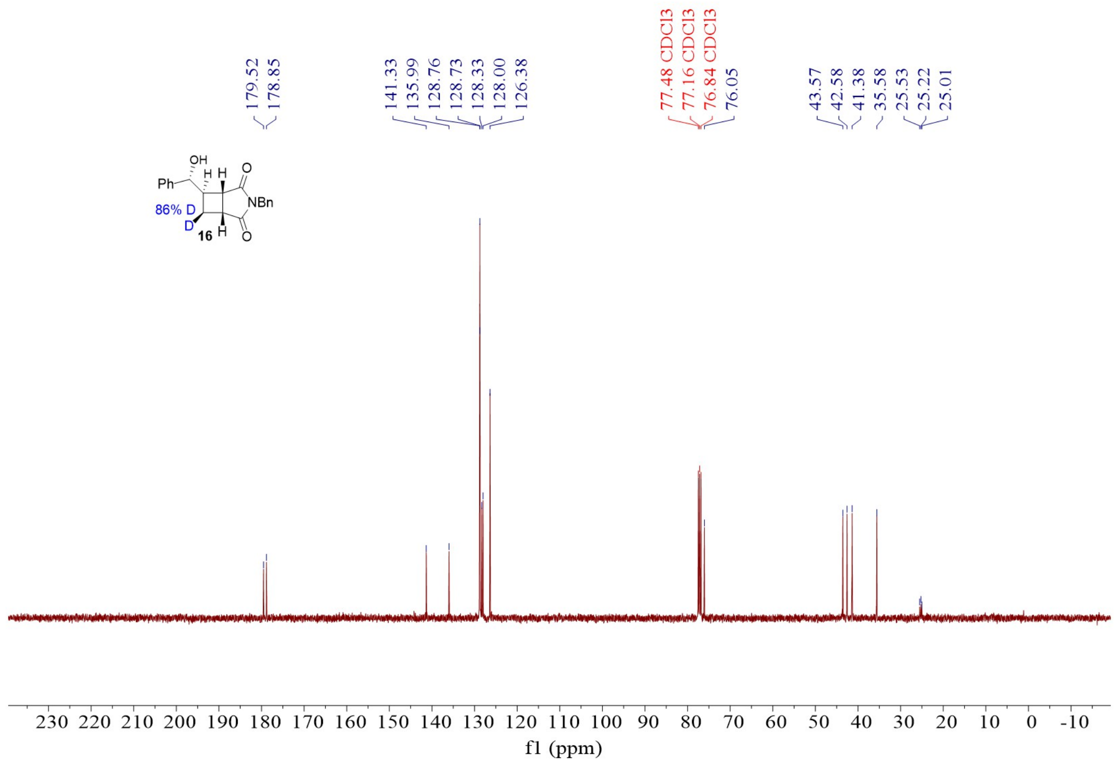
179.52
178.85
141.33
135.99
128.76
128.73
128.33
128.00
126.38
77.48 CDCl3
77.16 CDCl3
76.84 CDCl3
76.05
43.57
42.58
41.38
35.58
25.53
25.22
25.01
OH
Ph
H
H
O
NBn
86% D
D
16
H
O
230 220 210 200 190 180 170 160 150 140 130 120 110 100 90 80 70 60 50 40 30 20 10 0 -10
f1 (ppm)

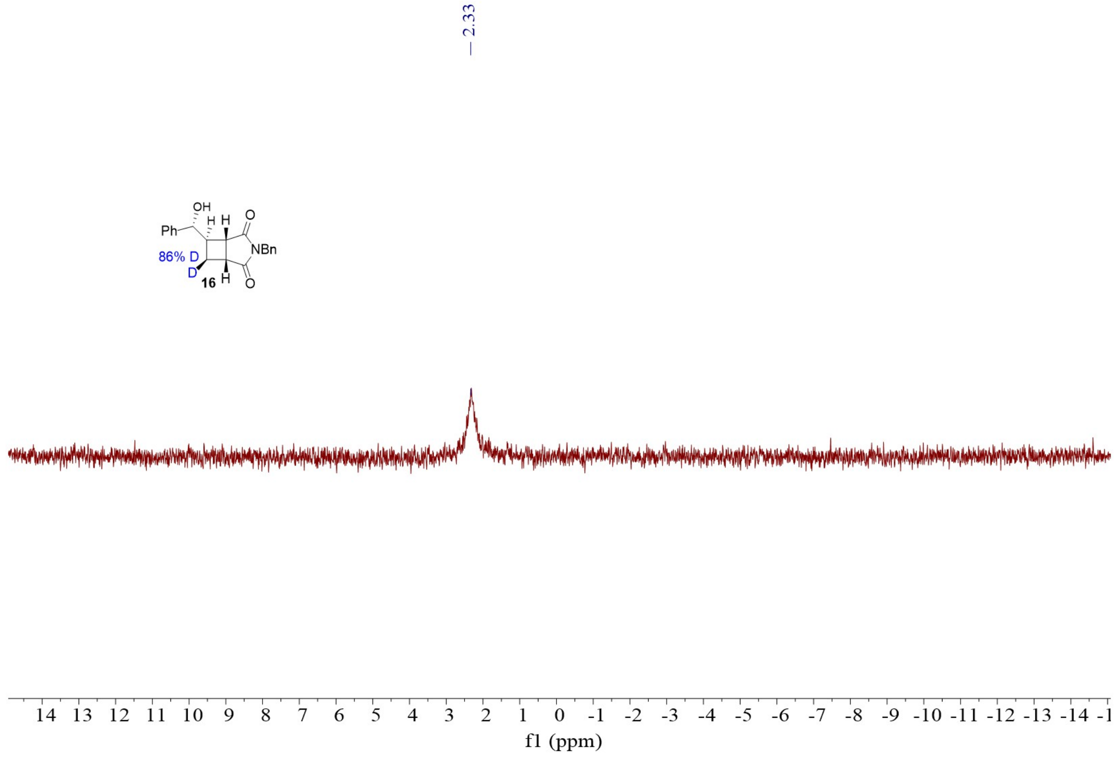
2.33
OH
Ph
H
H
O
NBn
86% D
D
16
H
O
14 13 12 11 10 9 8 7 6 5 4 3 2 1 0 -1 -2 -3 -4 -5 -6 -7 -8 -9 -10 -11 -12 -13 -14
f1 (ppm)

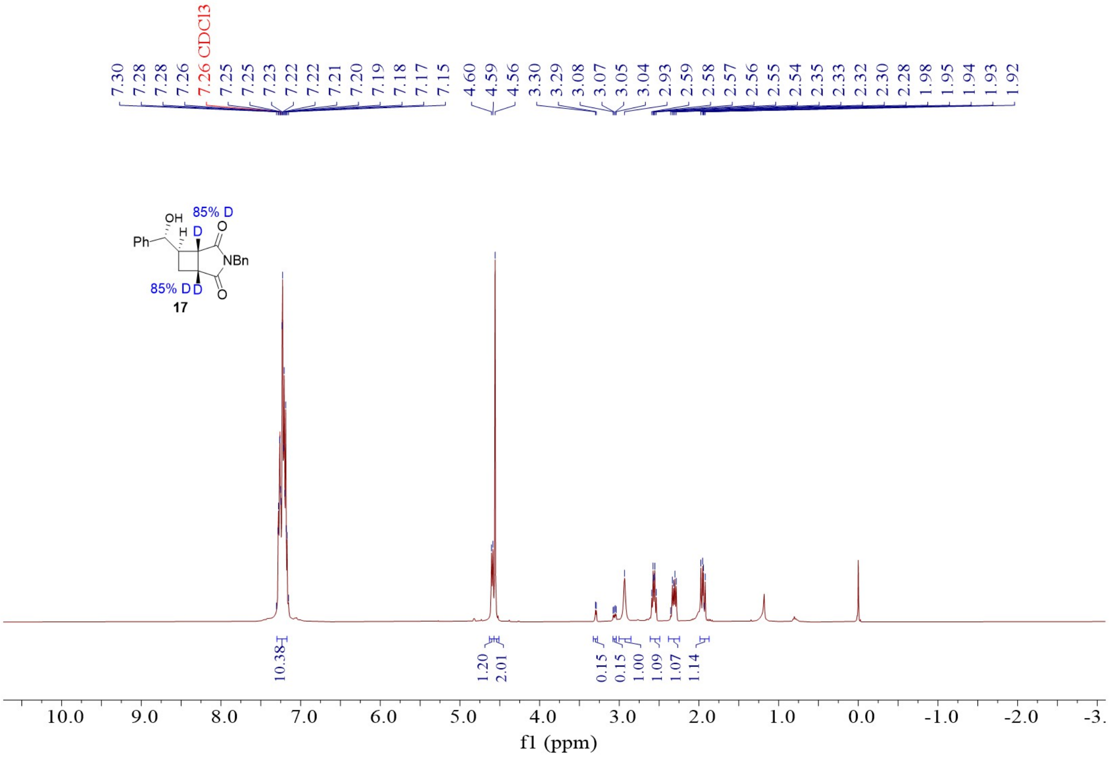
7.30 7.28 7.28 7.26 7.26 CDCl3 7.25 7.25 7.23 7.22 7.22 7.21 7.20 7.19 7.18 7.17 7.15 4.60 4.59 4.56 3.30 3.29 3.08 3.07 3.05 3.04 2.93 2.59 2.58 2.57 2.56 2.55 2.54 2.35 2.33 2.32 2.30 2.28 1.98 1.95 1.94 1.93 1.92
OH
85% D
Ph
H
D
O
NBn
85% D
D
O
17
10.38
1.20
2.01
0.15
0.15
1.00
1.09
1.07
1.14
10.0 9.0 8.0 7.0 6.0 5.0 4.0 3.0 2.0 1.0 0.0 -1.0 -2.0 -3.
f1 (ppm)

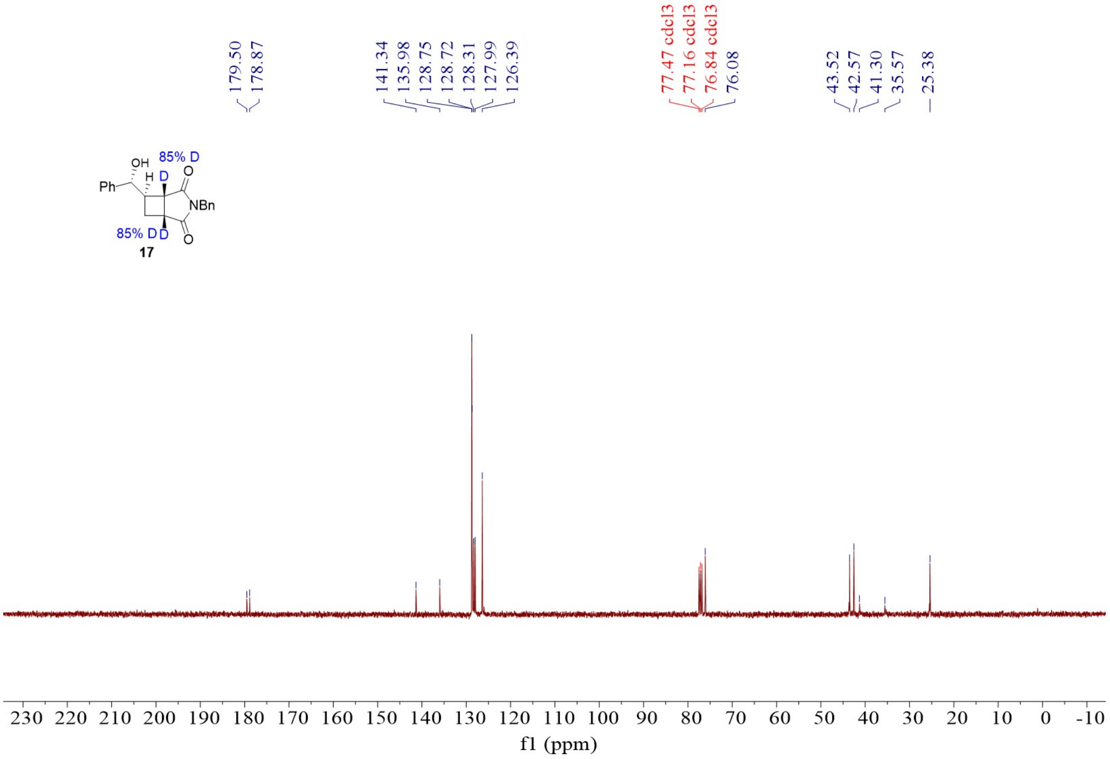
179.50 178.87
141.34 135.98 128.75 128.72 128.31 127.99 126.39
77.47 cdcl3 77.16 cdcl3 76.84 cdcl3 76.08
43.52 42.57 41.30 35.57 25.38
OH
85% D
Ph
H
D
O
NBn
85% D
D
O
17
230 220 210 200 190 180 170 160 150 140 130 120 110 100 90 80 70 60 50 40 30 20 10 0 -10
f1 (ppm)

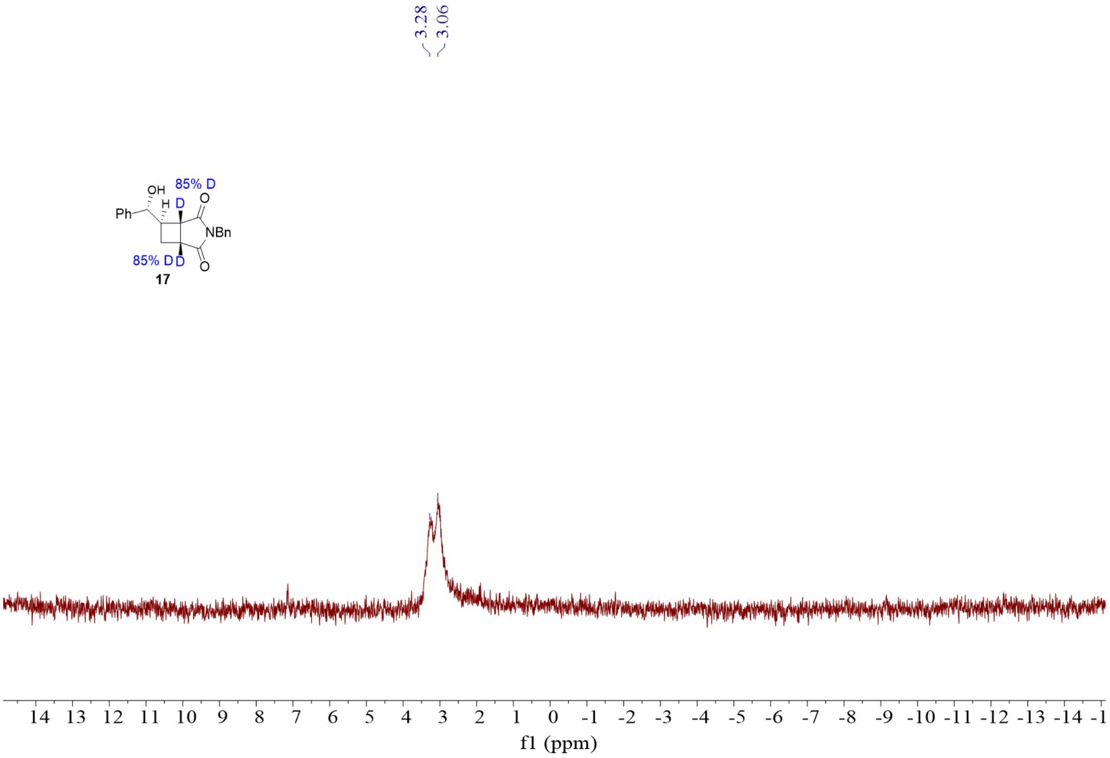
3.28
3.06
OH
85% D
Ph
H
D
NBn
85% D
D
17
f1 (ppm)

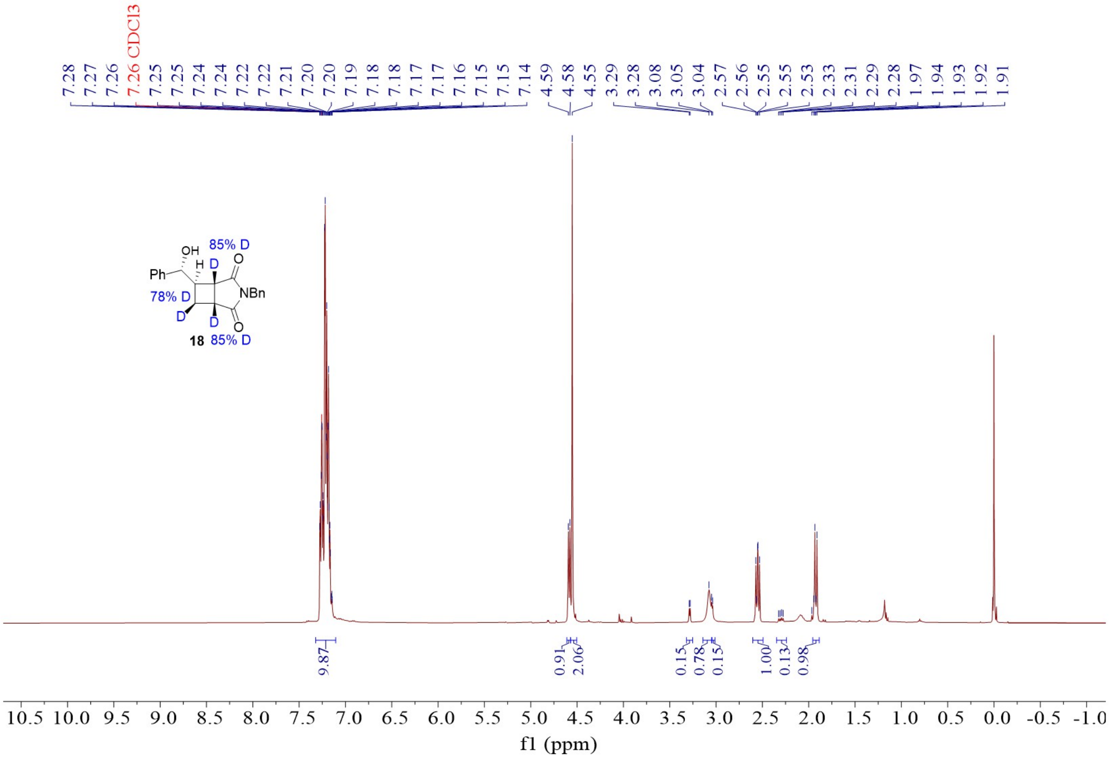
7.28
7.27
7.26
7.26 CDCl3
7.25
7.25
7.24
7.24
7.22
7.22
7.21
7.20
7.19
7.18
7.18
7.17
7.17
7.16
7.15
7.15
7.14
4.59
4.58
4.55
3.29
3.28
3.08
3.05
3.04
2.57
2.56
2.55
2.55
2.53
2.33
2.31
2.29
2.28
1.97
1.94
1.93
1.92
1.91
OH
85% D
Ph
H
D
78% D
NBn
D
D
18
85% D
9.87
0.91
2.06
0.15
0.78
0.15
1.00
0.13
0.98
f1 (ppm)

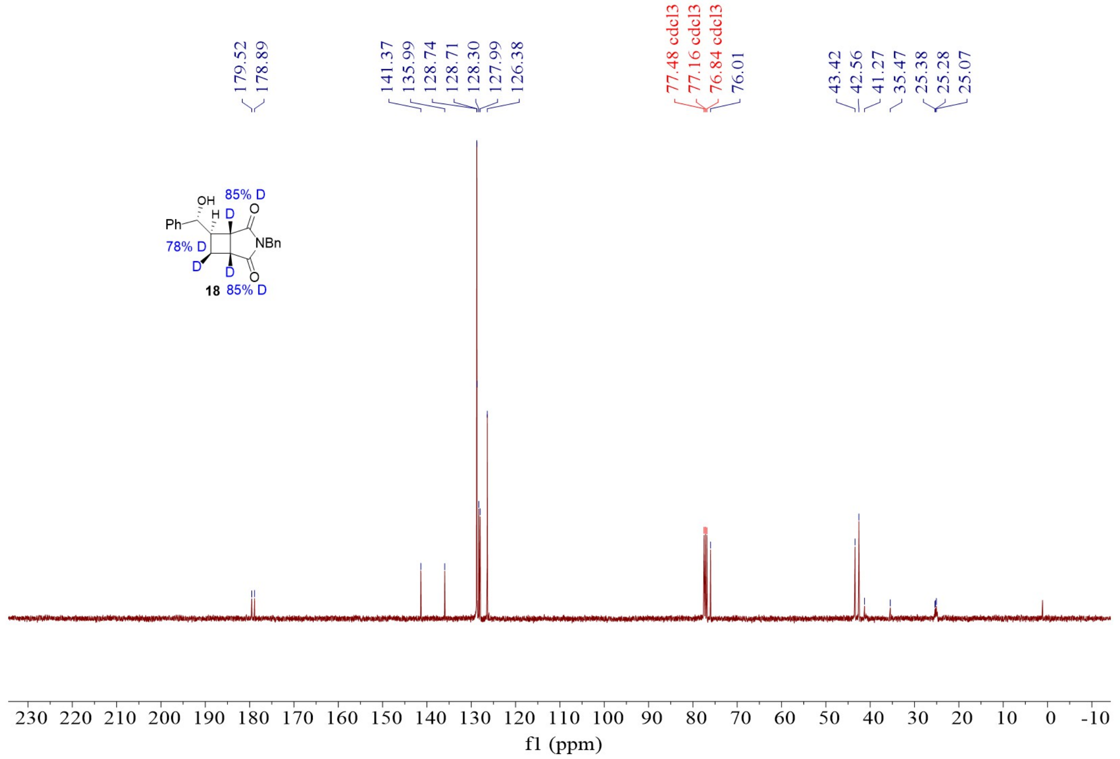
179.52
178.89
141.37
135.99
128.74
128.71
128.30
127.99
126.38
77.48 cdcl3
77.16 cdcl3
76.84 cdcl3
76.01
43.42
42.56
41.27
35.47
25.38
25.28
25.07
OH
85% D
H D
O
Ph
NBn
78% D
D
D
O
18 85% D
230 220 210 200 190 180 170 160 150 140 130 120 110 100 90 80 70 60 50 40 30 20 10 0 -10
f1 (ppm)

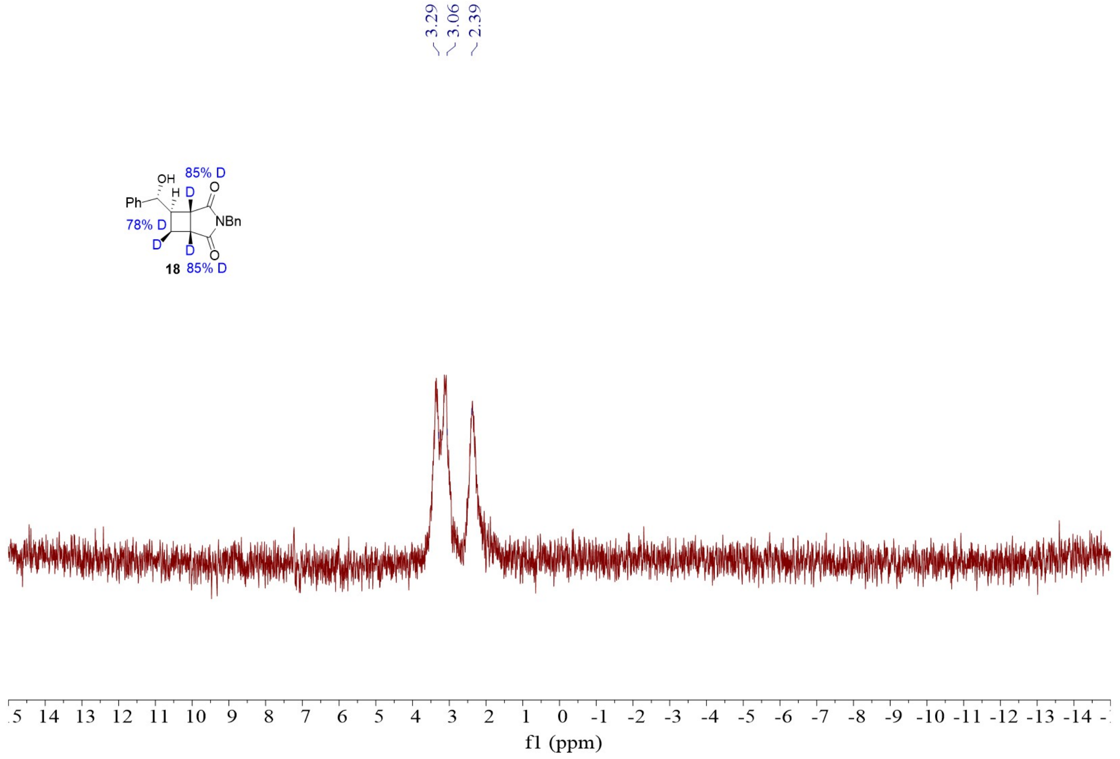
3.29
3.06
2.39
OH
85% D
H D
O
Ph
NBn
78% D
D
D
O
18 85% D
14 13 12 11 10 9 8 7 6 5 4 3 2 1 0 -1 -2 -3 -4 -5 -6 -7 -8 -9 -10 -11 -12 -13 -14
f1 (ppm)
